# Supplementary material for: Enantioselective synthesis of α-aminoboronates by NiH-catalysed asymmetric hydroamidation of alkenyl boronates
Source: Nat Commun. 2022 Sep 26;13:5630. doi: 10.1038/s41467-022-33411-9 (PMC9512809; doi:10.1038/s41467-022-33411-9)
Supplement: Supplementary file 1 — Supplementary Information [file 41467_2022_33411_MOESM1_ESM.pdf]

Supplementary Information

Enantioselective synthesis of α-aminoboronates by NiH-catalysed asymmetric hydroamidation of alkenyl boronates

Yao Zhang[1], Deyong Qiao[1], Mei Duan[1], You Wang[1,]*, and Shaolin Zhu[1,2,]*

[1]*State Key Laboratory of Coordination Chemistry, Jiangsu Key Laboratory of Advanced Organic Materials, Chemistry and Biomedicine Innovation Center (ChemBIC), School of Chemistry and Chemical Engineering, Nanjing University, Nanjing, 210093, China*

[2]*School of Chemistry and Chemical Engineering, Henan Normal University, Xinxiang, 453007, China*

*email: wangyou@nju.edu.cn; shaolinzhu@nju.edu.cn

I. Supplementary Methods

1. General Information

Solvents were either purified and dried by passage through alumina and Q5 reactant-packed columns on a solvent purification system or bought from the commercial sources and transferred to the glovebox without exposure to air. Other commercial reagents were purchased from Sigma-Aldrich, Acros, Alfa Aesar, TCI, Aladdin, J&K, Energy Chemical, Bide Pharmatech Ltd. and were used as received. Deionized water was used after degassing. Flash chromatography was performed using glass columns with silica gel (*SiliaFlash*® P60, particle size 40-63 μm, Silicycle).

$NiCl_2·6H_2O$ (CAS 7791-20-0, nickel(II) chloride hexahydrate, *ReagentPlus*®) was purchased from Sigma-Aldrich and stored under nitrogen in glovebox;

$(EtO)_3SiH$ (CAS 2031-62-1, triethoxysilane) was purchased from TCI and stored under nitrogen at –20 °C in glove box;

LiI (CAS 10377-51-2) was purchased from Aladdin or Energy Chemical (99.9% metals basis) and stored under nitrogen in glovebox;

DMA (CAS 127-19-5, *N,N*-dimethylacetamide) was purchased from Acros (99.5%, Extra Dry, *AcroSeal*®) and stored under nitrogen in glovebox.

Safety note: MSDS indicates that $(EtO)_3SiH$ is a corrosive and flammable liquid. According to the literatures[1], it may form pyrophoric gas (possibly SiH_4) during the storage or reaction. Although during our reactions, we used $(EtO)_3SiH$ without incident and SiH_4 was not observed, we urge the users of these procedures to be alert to the possibility of SiH_4 formation and possible exotherms and to take suitable precautions (suitable eye protection is also required). $(MeO)_2MeSiH$ could be an alternative hydride source in case of safety consideration.

General analytical information.

All compounds (starting materials and products) were characterized by ^{1}H NMR, ^{13}C NMR, IR spectroscopy and high-resolution mass spectrometry. ^{1}H NMR spectra were recorded on Bruker 500 MHz spectrometer and are referenced relative to residual

$CDCl_3$ proton signals at δ 7.26 ppm. ^{19}F NMR spectra were recorded on a Bruker 500 MHz spectrometer and are referenced to $CFCl_3$ (δ 0.0 ppm). Data for ^{1}H and ^{19}F NMR are reported as follows: chemical shift (δ ppm), multiplicity (s = singlet, d = doublet, t = triplet, q = quartet, m = multiplet, br = broad), integration, and coupling constant (Hz). ^{13}C NMR spectra were recorded on a Bruker 500 MHz spectrometer and are referenced to $CDCl_3$ at δ 77.16 ppm. The ^{13}C NMR spectra were obtained with ^{1}H decoupling. Data for ^{13}C NMR are reported in terms of chemical shift and multiplicity where appropriate. ^{11}B NMR spectra were recorded on a Bruker 500 MHz spectrometer and are referenced to $BF_3 \cdot Et_2O$ (δ 0.0 ppm), and the broad peaks around -3 ppm were ascribed to NMR tubes. IR spectra were obtained on a Bruker Alpha or Thermo Scientific Nicolet iS10 FT-IR and was reported in terms of frequency of absorption (cm^{-1}). GC analyses were performed on Agilent 7890 or 8890 gas chromatograph with an FID detector using a J&W DB-1 column (10 m, 0.1 mm I.D.). Low Resolution Mass spectra were obtained from on an Agilent 5977A GC-MS. High Resolution Mass spectra were obtained on a Thermo Fisher Q Exactive instrument (ESI). Melting points (m.p.) were obtained on a Mel-Temp capillary melting point apparatus. High pressure liquid chromatography (HPLC) was performed on Agilent 1260 Series chromatographs using Daicel Chiralcel & Chiralpak columns (250 mm). Optical rotations were measured on a Rudolph Research Analytical Autopol VI automatic polarimeter using a 50 mm pathlength cell at 589 nm with $[\alpha]_D$ values reported in degrees; concentration (c) is in g/100 mL.

Medium-sized screw-cap test tubes (8 mL) were used for all 0.20 mmol scale reactions: Fisher 13 x 100 mm tubes (Cat. No. 14-959-35C)

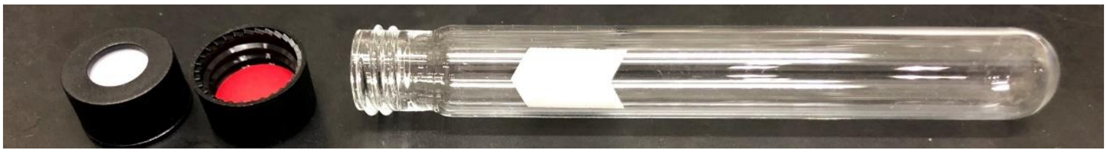

Cap with Septa: Thermo Scientific ASM PHN CAP w/PTFE/SIL (Cat. No. 03378316)

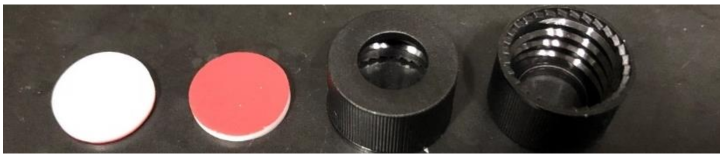

2. NiH-Catalyzed Asymmetric Hydroamidation of Alkenyl Boronates

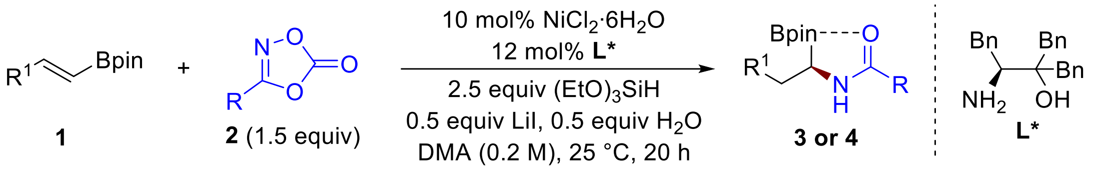

General procedure A for NiH-catalyzed asymmetric hydroamidation of alkenyl boronates. In a nitrogen-filled glove box, to an oven-dried 8 mL screw-cap vial equipped with a magnetic stir bar was added $NiCl_2{\cdot}6H_2O$ (4.8 mg, 10 mol%), **L*** (8.0 mg, 12 mol%), LiI (13.4 mg, 0.10 mmol, 0.50 equiv), 1,4,2-dioxazol-5-one (0.30 mmol, 1.5 equiv) (if the olefin is a solid, it was also added at this time) and anhydrous DMA (1.0 mL, 0.20 M). The mixture was stirred for 10 min at room temperature, at which time alkenyl boronate (0.20 mmol, 1.0 equiv) (if the 1,4,2-dioxazol-5-one is a liquid, it was added at this time), H_2O (1.8 μL, 0.10 mmol, 0.50 equiv) and $(EtO)_3SiH$ (92 μL, 0.50 mmol, 2.5 equiv) were added to the resulting mixture in this order. The tube was sealed with a teflon-lined screw cap, removed from the glove box and the reaction was stirred at 25 °C water bath for up to 20 h (the mixture was stirred at 800 rpm). After the reaction was complete, the reaction was quenched upon the addition of H_2O, and the mixture was extracted with Et_2O. The organic layer was concentrated to give the crude product. *n*-Dodecane (20 μL) was added as an internal standard for GC analysis. The product was purified by flash column chromatography (petroleum ether/EtOAc) for each substrate. The yields reported are the average of at least two experiments, unless otherwise indicated. The enantiomeric excesses (% *ee*) were determined by HPLC analysis using chiral stationary phases.

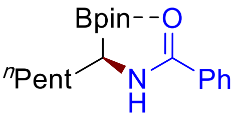

(*R*)-*N*-(1-(4,4,5,5-Tetramethyl-1,3,2-dioxaborolan-2-yl)hexyl)benzamide (Figure 3, **3a**). From (*E*)-2-(hex-1-en-1-yl)-4,4,5,5-tetramethyl-1,3,2-dioxaborolane (**1a**) (42.0 mg, 0.20 mmol, 1.0 equiv) and 3-phenyl-1,4,2-dioxazol-5-one (**2a**) (48.9 mg, 0.30 mmol, 1.5 equiv), the title compound was prepared following the general procedure **A**

using $NiCl_2 \cdot 6H_2O$ (4.8 mg, 10 mol%), **L*** (8.0 mg, 12 mol%), LiI (13.4 mg, 0.10 mmol, 0.50 equiv), H_2O (1.8 μL, 0.10 mmol, 0.50 equiv), $(EtO)_3SiH$ (92 μL, 0.50 mmol, 2.5 equiv), anhydrous DMA (1.0 mL). The reaction mixture was stirred for 20 h at 25 °C. The crude material was purified by flash column chromatography (petroleum ether/EtOAc = 2:1) to provide the title compound as a white solid in 71% yield (46.8 mg).

^{1}H NMR (500 MHz, $CDCl_3$) δ 8.47 (s, 1H), 7.80 (d, *J* = 7.4 Hz, 2H), 7.45 (t, *J* = 7.4 Hz, 1H), 7.32 (t, *J* = 7.7 Hz, 2H), 2.79 (t, *J* = 6.3 Hz, 1H), 1.75 – 1.65 (m, 1H), 1.61 – 1.52 (m, 1H), 1.49 – 1.37 (m, 2H), 1.34 – 1.28 (m, 4H), 1.26 (s, 12H), 0.88 (t, *J* = 7.0 Hz, 3H);

^{13}C NMR (126 MHz, $CDCl_3$) δ 170.8, 133.1, 128.6, 128.2, 128.1, 81.2, 32.1, 31.3, 27.7, 25.4, 25.2, 22.7, 14.2;

^{11}B NMR (160 MHz, $CDCl_3$) δ 17.8;

HRMS (ESI) calcd. for $C_{19}H_{30}BNNaO_3$ $[M+Na]^+$ m/z 354.2211, found 354.2202;

IR (neat, cm^{-1}) 3079, 2925, 1608, 1530, 1127, 1099, 709;

m.p. 130 – 132 °C;

$[\alpha]_D^{25}$ = –35.8 (c = 1.06, $CHCl_3$);

HPLC analysis: the *ee* (95%) was determined using a CHIRALPAK® IE-3 column, 5% EtOH in hexane, 1.0 mL/min, 240 nm UV detector, t_R (major) = 6.8 min, t_R (minor) = 7.4 min.

From (*Z*)-**1a** (Figure 2, entry 15): (*Z*)-2-(hex-1-en-1-yl)-4,4,5,5-tetramethyl-1,3,2-dioxaborolane ((*Z*)-**1a**) (42.0 mg, 0.20 mmol, 1.0 equiv) and 3-phenyl-1,4,2-dioxazol-5-one (**2a**) (48.9 mg, 0.30 mmol, 1.5 equiv) were used. The title compound was prepared following the general procedure A. The crude material was purified by flash column chromatography (petroleum ether/EtOAc = 2:1) to provide the title compound as a white solid in 64% yield (42.6 mg).

HPLC analysis: the *ee* (85%) was determined using a CHIRALPAK® IE-3 column, 5% EtOH in hexane, 1.0 mL/min, 240 nm UV detector, t_R (major) = 6.9 min, t_R (minor) =

7.5 min.

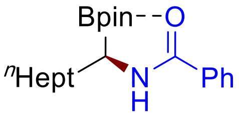

(*R*)-*N*-(1-(4,4,5,5-Tetramethyl-1,3,2-dioxaborolan-2-yl)octyl)benzamide (Figure 3, **3b**). From (*E*)-4,4,5,5-tetramethyl-2-(oct-1-en-1-yl)-1,3,2-dioxaborolane (**1b**) (47.6 mg, 0.20 mmol, 1.0 equiv) and 3-phenyl-1,4,2-dioxazol-5-one (**2a**) (48.9 mg, 0.30 mmol, 1.5 equiv), the title compound was prepared following the general procedure **A** using $NiCl_2 \cdot 6H_2O$ (4.8 mg, 10 mol%), **L*** (8.0 mg, 12 mol%), LiI (13.4 mg, 0.10 mmol, 0.50 equiv), H_2O (1.8 μL, 0.10 mmol, 0.50 equiv), $(EtO)_3SiH$ (92 μL, 0.50 mmol, 2.5 equiv), anhydrous DMA (1.0 mL). The reaction mixture was stirred for 20 h at 25 °C. The crude material was purified by flash column chromatography (petroleum ether/EtOAc = 2:1) to provide the title compound as a colorless oil in 73% yield (52.5 mg).

^{1}H NMR (500 MHz, $CDCl_3$) δ 8.16 (s, 1H), 7.80 (d, *J* = 7.2 Hz, 2H), 7.47 (t, *J* = 7.5 Hz, 1H), 7.34 (t, *J* = 7.8 Hz, 2H), 2.86 – 2.77 (m, 1H), 1.74 – 1.66 (m, 1H), 1.60 – 1.53 (m, 1H), 1.47 – 1.35 (m, 2H), 1.33 – 1.20 (m, 20H), 0.88 (t, *J* = 6.9 Hz, 3H);

^{13}C NMR (126 MHz, $CDCl_3$) δ 170.9, 133.1, 128.6, 128.2, 81.2, 32.0, 31.4, 29.9, 29.4, 28.0, 25.4, 25.3, 22.8, 14.3;

^{11}B NMR (160 MHz, $CDCl_3$) δ 18.3;

HRMS (ESI) calcd. for $C_{21}H_{35}BNO_3$ $[M+H]^+$ m/z 360.2705, found 360.2699;

IR (neat, cm^{-1}) 3196, 2925, 2855, 1610, 1112, 705;

$[\alpha]_D^{25}$ = –36.4 (c = 1.10, $CHCl_3$);

HPLC analysis: the *ee* (95%) was determined using a CHIRALCEL® OD-H column, 8% *i*PrOH in hexane, 0.5 mL/min, 254 nm UV detector, t_R (minor) = 7.3 min, t_R (major) = 8.0 min.

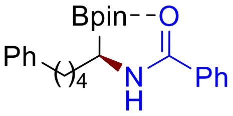

(*R*)-*N*-(5-Phenyl-1-(4,4,5,5-tetramethyl-1,3,2-dioxaborolan-2-yl)pentyl)

benzamide (Figure 3, **3c**). From (*E*)-4,4,5,5-tetramethyl-2-(5-phenylpent-1-en-1-yl)-1,3,2-dioxaborolane (**1c**) (54.4 mg, 0.20 mmol, 1.0 equiv) and 3-phenyl-1,4,2-dioxazol-5-one (**2a**) (48.9 mg, 0.30 mmol, 1.5 equiv), the title compound was prepared following the general procedure **A** using $NiCl_2·6H_2O$ (4.8 mg, 10 mol%), **L*** (8.0 mg, 12 mol%), LiI (13.4 mg, 0.10 mmol, 0.50 equiv), H_2O (1.8 μL, 0.10 mmol, 0.50 equiv), $(EtO)_3SiH$ (92 μL, 0.50 mmol, 2.5 equiv), anhydrous DMA (1.0 mL). The reaction mixture was stirred for 20 h at 25 °C. The crude material was purified by flash column chromatography (petroleum ether/EtOAc = 2:1) to provide the title compound as a colorless oil in 56% yield (44.2 mg).

^{1}H NMR (500 MHz, $CDCl_3$) δ 7.97 (s, 1H), 7.78 (d, *J* = 7.4 Hz, 2H), 7.49 (t, *J* = 7.5 Hz, 1H), 7.35 (t, *J* = 7.8 Hz, 2H), 7.26 (t, *J* = 7.5 Hz, 2H), 7.20 – 7.14 (m, 3H), 2.87 – 2.78 (m, 1H), 2.69 – 2.55 (m, 2H), 1.78 – 1.69 (m, 1H), 1.69 – 1.57 (m, 3H), 1.51 – 1.40 (m, 2H), 1.25 (s, 12H);

^{13}C NMR (126 MHz, $CDCl_3$) δ 171.0, 142.8, 133.2, 128.7, 128.6, 128.4, 128.2, 125.7, 81.3, 35.9, 31.6, 31.2, 27.5, 25.4, 25.3;

^{11}B NMR (160 MHz, $CDCl_3$) δ 18.2;

HRMS (ESI) calcd. for $C_{24}H_{32}BNNaO_3$ $[M+Na]^+$ m/z 416.2367, found 416.2358;

IR (neat, cm^{-1}) 3193, 2970, 2927, 1610, 1576, 1113, 698, 580;

$[α]_D^{25}$ = –32.4 (c = 0.89, $CHCl_3$);

HPLC analysis: the *ee* (96%) was determined using a CHIRALPAK® IE-3 column, 10% EtOH in hexane, 0.8 mL/min, 240 nm UV detector, t_R (major) = 7.4 min, t_R (minor) = 7.8 min.

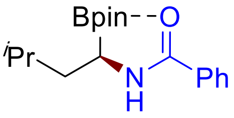

(*R*)-*N*-(3-Methyl-1-(4,4,5,5-tetramethyl-1,3,2-dioxaborolan-2-yl)butyl)benzamide (Figure 3, **3d**). From (*E*)-4,4,5,5-tetramethyl-2-(3-methylbut-1-en-1-yl)-1,3,2-dioxaborolane (**1d**) (39.2 mg, 0.20 mmol, 1.0 equiv) and 3-phenyl-1,4,2-dioxazol-5-one (**2a**) (48.9 mg, 0.30 mmol, 1.5 equiv), the title compound was prepared following

the general procedure **A** using $NiCl_2 \cdot 6H_2O$ (4.8 mg, 10 mol%), **L*** (8.0 mg, 12 mol%), LiI (13.4 mg, 0.10 mmol, 0.50 equiv), H_2O (1.8 μL, 0.10 mmol, 0.50 equiv), $(EtO)_3SiH$ (92 μL, 0.50 mmol, 2.5 equiv), anhydrous DMA (1.0 mL). The reaction mixture was stirred for 20 h at 25 °C. The crude material was purified by flash column chromatography (petroleum ether/EtOAc = 2:1) to provide the title compound as a white solid in 68% yield (43.0 mg).

^{1}H NMR (500 MHz, $CDCl_3$) δ 7.77 (d, *J* = 7.4 Hz, 2H), 7.64 (s, 1H), 7.50 (t, *J* = 7.4 Hz, 1H), 7.38 (t, *J* = 7.7 Hz, 2H), 2.95 (t, *J* = 6.8 Hz, 1H), 1.79 – 1.67 (m, 1H), 1.53 – 1.47 (m, 2H), 1.27 (s, 12H), 0.96 (d, *J* = 6.5 Hz, 6H);

^{13}C NMR (126 MHz, $CDCl_3$) δ 170.9, 133.1, 128.7, 128.4, 128.0, 81.2, 40.6, 26.3, 25.3, 25.3, 23.6, 22.2;

^{11}B NMR (160 MHz, $CDCl_3$) δ 18.6;

HRMS (ESI) calcd. for $C_{18}H_{29}BNO_3$ $[M+H]^+$ m/z 318.2235, found 318.2231;

IR (neat, cm^{-1}) 2958, 1609, 1528, 1113, 1098, 707;

$[\alpha]_D^{25}$ = –37.3 (c = 0.96, $CHCl_3$);

HPLC analysis: the *ee* (92%) was determined using a CHIRALPAK® AD-H column, 5% *i*PrOH in hexane, 1.0 mL/min, 240 nm UV detector, t_R (minor) = 4.8 min, t_R (major) = 5.5 min.

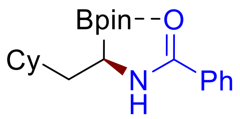

(*R*)-*N*-(2-Cyclohexyl-1-(4,4,5,5-tetramethyl-1,3,2-dioxaborolan-2-yl)ethyl)benzamide (Figure 3, **3e**). From (*E*)-2-(2-cyclohexylvinyl)-4,4,5,5-tetramethyl-1,3,2-dioxaborolane (**1e**) (47.2 mg, 0.20 mmol, 1.0 equiv) and 3-phenyl-1,4,2-dioxazol-5-one (**2a**) (48.9 mg, 0.30 mmol, 1.5 equiv), the title compound was prepared following the general procedure **A** using $NiCl_2 \cdot 6H_2O$ (4.8 mg, 10 mol%), **L*** (8.0 mg, 12 mol%), LiI (13.4 mg, 0.10 mmol, 0.50 equiv), H_2O (1.8 μL, 0.10 mmol, 0.50 equiv), $(EtO)_3SiH$ (92 μL, 0.50 mmol, 2.5 equiv), anhydrous DMA (1.0 mL). The reaction mixture was stirred for 20 h at 25 °C. The crude material was purified by flash

column chromatography (petroleum ether/EtOAc = 2:1) to provide the title compound as a colorless oil in 70% yield (50.0 mg).

^{1}H NMR (500 MHz, $CDCl_3$) δ 7.82 – 7.76 (m, 2H), 7.71 (s, 1H), 7.50 (t, J = 7.5 Hz, 1H), 7.38 (t, J = 7.8 Hz, 2H), 3.00 – 2.92 (m, 1H), 1.91 – 1.81 (m, 1H), 1.76 – 1.63 (m, 4H), 1.60 – 1.51 (m, 1H), 1.51 – 1.45 (m, 1H), 1.43 – 1.36 (m, 1H), 1.27 (s, 12H), 1.22 – 1.11 (m, 3H), 1.01 – 0.84 (m, 2H);

^{13}C NMR (126 MHz, $CDCl_3$) δ 170.8, 133.2, 128.7, 128.3, 128.1, 81.2, 39.1, 35.8, 34.2, 33.0, 26.8, 26.5, 26.5, 25.3, 25.3;

^{11}B NMR (160 MHz, $CDCl_3$) δ 18.6;

HRMS (ESI) calcd. for $C_{21}H_{33}BNO_3$ $[M+H]^+$ m/z 358.2548, found 358.2541;

IR (neat, cm^{-1}) 3066, 2921, 1611, 1122, 705;

$[\alpha]_D^{25}$ = –41.2 (c = 1.08, $CHCl_3$);

HPLC analysis: the *ee* (93%) was determined using a CHIRALPAK® AD-H column, 5% *i*PrOH in hexane, 1.0 mL/min, 254 nm UV detector, t_R (minor) = 6.0 min, t_R (major) = 6.8 min.

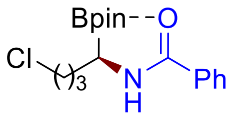

(*R*)-*N*-(4-chloro-1-(4,4,5,5-tetramethyl-1,3,2-dioxaborolan-2-yl)butyl)benzamide (Figure 3, **3f**). From (*E*)-2-(4-chlorobut-1-en-1-yl)-4,4,5,5-tetramethyl-1,3,2-dioxaborolane (**1f**) (43.3 mg, 0.20 mmol, 1.0 equiv) and 3-phenyl-1,4,2-dioxazol-5-one (**2a**) (48.9 mg, 0.30 mmol, 1.5 equiv), the title compound was prepared following the general procedure **A** using $NiCl_2·6H_2O$ (4.8 mg, 10 mol%), **L*** (8.0 mg, 12 mol%), LiI (13.4 mg, 0.10 mmol, 0.50 equiv), H_2O (1.8 μL, 0.10 mmol, 0.50 equiv), $(EtO)_3SiH$ (92 μL, 0.50 mmol, 2.5 equiv), anhydrous DMA (1.0 mL). The reaction mixture was stirred for 20 h at 25 °C. The crude material was purified by flash column chromatography (petroleum ether/EtOAc = 3:2) to provide the title compound as a white solid in 59% yield (37.9 mg).

^{1}H NMR (500 MHz, $CDCl_3$) δ 8.86 (s, 1H), 7.80 (d, J = 7.4 Hz, 2H), 7.46 (t, J = 7.5

Hz, 1H), 7.32 (t, J = 7.7 Hz, 2H), 3.60 – 3.49 (m, 2H), 2.79 (t, J = 6.5 Hz, 1H), 2.02 – 1.88 (m, 2H), 1.86 – 1.77 (m, 1H), 1.77 – 1.68 (m, 1H), 1.27 (s, 12H);

^{13}C NMR (126 MHz, $CDCl_3$) δ 171.3, 133.5, 128.7, 128.4, 127.2, 81.3, 45.3, 30.7, 28.9, 25.4, 25.3;

^{11}B NMR (160 MHz, $CDCl_3$) δ 16.7;

HRMS (ESI) calcd. for $C_{17}H_{26}BClNO_3$ $[M+H]^+$ m/z 338.1689, found 338.1682;

IR (neat, cm^{-1}) 3078, 2966, 1603, 1569, 1532, 1109, 712;

m.p. 53 – 55 °C;

$[\alpha]_D^{25}$ = –53.3 (c = 0.51, $CHCl_3$);

HPLC analysis: the *ee* (95%) was determined using a CHIRALPAK® ID-3 column, 5% EtOH in hexane, 1.0 mL/min, 240 nm UV detector, t_R (major) = 5.1 min, t_R (minor) = 5.5 min.

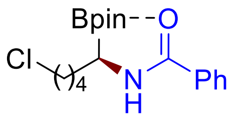

(*R*)-*N*-(5-Chloro-1-(4,4,5,5-tetramethyl-1,3,2-dioxaborolan-2-yl)pentyl)benzamide (Figure 3, **3g**). From (*E*)-2-(5-chloropent-1-en-1-yl)-4,4,5,5-tetramethyl-1,3,2-dioxaborolane (**1g**) (46.1 mg, 0.20 mmol, 1.0 equiv) and 3-phenyl-1,4,2-dioxazol-5-one (**2a**) (48.9 mg, 0.30 mmol, 1.5 equiv), the title compound was prepared following the general procedure **A** using $NiCl_2\cdot6H_2O$ (4.8 mg, 10 mol%), **L*** (8.0 mg, 12 mol%), LiI (13.4 mg, 0.10 mmol, 0.50 equiv), H_2O (1.8 μL, 0.10 mmol, 0.50 equiv), $(EtO)_3SiH$ (92 μL, 0.50 mmol, 2.5 equiv), anhydrous DMA (1.0 mL). The reaction mixture was stirred for 20 h at 25 °C. The crude material was purified by flash column chromatography (petroleum ether/EtOAc = 3:2) to provide the title compound as a colorless oil in 55% yield (38.4 mg).

^{1}H NMR (500 MHz, $CDCl_3$) δ 8.52 (s, 1H), 7.80 (d, J = 7.3 Hz, 2H), 7.47 (t, J = 7.5 Hz, 1H), 7.34 (t, J = 7.8 Hz, 2H), 3.59 – 3.48 (m, 2H), 2.83 – 2.75 (m, 1H), 1.83 – 1.74 (m, 2H), 1.74 – 1.66 (s, 1H), 1.64 – 1.51 (m, 3H), 1.26 (s, 12H);

^{13}C NMR (126 MHz, $CDCl_3$) δ 171.1, 133.3, 128.7, 128.3, 127.7, 81.2, 45.2, 32.8, 30.7,

25.5, 25.3, 25.1;

^{11}B NMR (160 MHz, $CDCl_3$) δ 17.6;

HRMS (ESI) calcd. for $C_{18}H_{27}BClNNaO_3$ $[M+Na]^+$ m/z 374.1665, found 374.1656;

IR (neat, cm^{-1}) 3193, 2971, 2929, 1610, 1576, 1111, 734;

$[\alpha]_D^{25}$ = –40.0 (c = 1.08, $CHCl_3$);

HPLC analysis: the *ee* (96%) was determined using a CHIRALPAK® AD-H column, 5% *i*PrOH in hexane, 1.0 mL/min, 240 nm UV detector, t_R (minor) = 7.9 min, t_R (major) = 9.2 min.

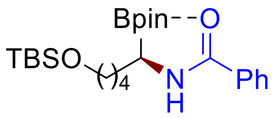

(*R*)-*N*-(5-((*tert*-Butyldimethylsilyl)oxy)-1-(4,4,5,5-tetramethyl-1,3,2-dioxaborolan-2-yl)pentyl)benzamide (Figure 3, **3h**). From (*E*)-*tert*-butyldimethyl((5-(4,4,5,5-tetramethyl-1,3,2-dioxaborolan-2-yl)pent-4-en-1-yl)oxy)silane (**1h**) (65.3 mg, 0.20 mmol, 1.0 equiv) and 3-phenyl-1,4,2-dioxazol-5-one (**2a**) (48.9 mg, 0.30 mmol, 1.5 equiv), the title compound was prepared following the general procedure **A** using $NiCl_2 \cdot 6H_2O$ (4.8 mg, 10 mol%), **L*** (8.0 mg, 12 mol%), LiI (13.4 mg, 0.10 mmol, 0.50 equiv), H_2O (1.8 μL, 0.10 mmol, 0.50 equiv), $(EtO)_3SiH$ (92 μL, 0.50 mmol, 2.5 equiv), anhydrous DMA (1.0 mL). The reaction mixture was stirred for 20 h at 25 °C. The crude material was purified by flash column chromatography (petroleum ether/EtOAc = 2:1) to provide the title compound as a colorless oil in 76% yield (68.2 mg).

^{1}H NMR (500 MHz, $CDCl_3$) δ 7.82 – 7.77 (m, 2H), 7.68 (s, 1H), 7.53 (t, *J* = 7.5 Hz, 1H), 7.41 (t, *J* = 7.8 Hz, 2H), 3.64 (t, *J* = 6.2 Hz, 2H), 2.92 – 2.83 (m, 1H), 1.78 – 1.67 (m, 1H), 1.68 – 1.44 (m, 5H), 1.27 (s, 6H), 1.26 (s, 6H), 0.88 (s, 9H), 0.04 (s, 6H);

^{13}C NMR (126 MHz, $CDCl_3$) δ 170.9, 133.2, 128.8, 128.5, 128.0, 81.3, 63.3, 32.9, 31.0, 26.2, 25.4, 25.2, 24.2, 18.5, -5.1;

^{11}B NMR (160 MHz, $CDCl_3$) δ 18.9;

HRMS (ESI) calcd. for $C_{24}H_{42}BNNaO_4Si$ $[M+Na]^+$ m/z 470.2868, found 470.2858;

IR (neat, cm^{-1}) 3070, 2928, 2857, 1611, 1096, 706;

[α]$_D^{25}$ = –41.6 (c = 0.98, $CHCl_3$);

HPLC analysis: the *ee* (95%) was determined using a CHIRALPAK® IG-3 column, 5% EtOH in hexane, 0.8 mL/min, 240 nm UV detector, t_R (major) = 4.9 min, t_R (minor) = 5.7 min.

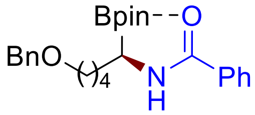

(*R*)-*N*-(5-(Benzyloxy)-1-(4,4,5,5-tetramethyl-1,3,2-dioxaborolan-2-yl)pentyl)benzamide (Figure 3, **3i**). From (*E*)-2-(5-(benzyloxy)pent-1-en-1-yl)-4,4,5,5-tetramethyl-1,3,2-dioxaborolane (**1i**) (60.4 mg, 0.20 mmol, 1.0 equiv) and 3-phenyl-1,4,2-dioxazol-5-one (**2a**) (48.9 mg, 0.30 mmol, 1.5 equiv), the title compound was prepared following the general procedure **A** using $NiCl_2{\cdot}6H_2O$ (4.8 mg, 10 mol%), **L*** (8.0 mg, 12 mol%), LiI (13.4 mg, 0.10 mmol, 0.50 equiv), H_2O (1.8 µL, 0.10 mmol, 0.50 equiv), $(EtO)_3SiH$ (92 µL, 0.50 mmol, 2.5 equiv), anhydrous DMA (1.0 mL). The reaction mixture was stirred for 20 h at 25 °C. The crude material was purified by flash column chromatography (petroleum ether/EtOAc = 1:1) to provide the title compound as a colorless oil in 66% yield (55.9 mg).

^{1}H NMR (500 MHz, $CDCl_3$) δ 7.93 (s, 1H), 7.77 (d, *J* = 7.5 Hz, 2H), 7.50 (t, *J* = 7.5 Hz, 1H), 7.36 (t, *J* = 7.8 Hz, 2H), 7.33 – 7.29 (m, 4H), 7.28 – 7.24 (m, 1H), 4.50 (s, 2H), 3.51 (t, *J* = 6.3 Hz, 2H), 2.90 – 2.80 (m, 1H), 1.76 – 1.57 (m, 4H), 1.57 – 1.51 (m, 2H), 1.26 (s, 12H);

^{13}C NMR (126 MHz, $CDCl_3$) δ 171.0, 138.6, 133.2, 128.7, 128.5, 128.1, 127.8, 127.7, 81.1, 73.1, 70.6, 30.9, 29.6, 25.4, 25.2, 24.6;

^{11}B NMR (160 MHz, $CDCl_3$) δ 18.3;

HRMS (ESI) calcd. for $C_{25}H_{34}BNNaO_4$ $[M+Na]^+$ m/z 446.2473, found 446.2462;

IR (neat, cm^{-1}) 3195, 2927, 2856, 1610, 1098, 707;

[α]$_D^{25}$ = –41.6 (c = 1.06, $CHCl_3$);

HPLC analysis: the *ee* (96%) was determined using a CHIRALPAK® IG-3 column, 5% EtOH in hexane, 1.0 mL/min, 254 nm UV detector, t_R (major) = 8.3 min, t_R (minor) =

11.6 min.

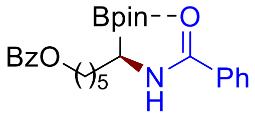

(*R*)-6-Benzamido-6-(4,4,5,5-tetramethyl-1,3,2-dioxaborolan-2-yl)hexyl benzoate (Figure 3, **3j**). From (*E*)-6-(4,4,5,5-tetramethyl-1,3,2-dioxaborolan-2-yl)hex-5-en-1-yl benzoate (**1j**) (66.0 mg, 0.20 mmol, 1.0 equiv) and 3-phenyl-1,4,2-dioxazol-5-one (**2a**) (48.9 mg, 0.30 mmol, 1.5 equiv), the title compound was prepared following the general procedure **A** using $NiCl_2\cdot 6H_2O$ (4.8 mg, 10 mol%), **L*** (8.0 mg, 12 mol%), LiI (13.4 mg, 0.10 mmol, 0.50 equiv), H_2O (1.8 μL, 0.10 mmol, 0.50 equiv), $(EtO)_3SiH$ (92 μL, 0.50 mmol, 2.5 equiv), anhydrous DMA (1.0 mL). The reaction mixture was stirred for 20 h at 25 °C. The crude material was purified by flash column chromatography (petroleum ether/EtOAc = 3:2) to provide the title compound as a colorless oil in 66% yield (59.7 mg).

^{1}H NMR (500 MHz, $CDCl_3$) δ 8.06 – 7.92 (m, 3H), 7.87 (d, *J* = 7.3 Hz, 2H), 7.57 – 7.48 (m, 2H), 7.44 – 7.37 (m, 4H), 4.48 – 4.39 (m, 1H), 4.32 – 4.22 (m, 1H), 2.83 (t, *J* = 5.9 Hz, 1H), 1.87 – 1.78 (m, 1H), 1.78 – 1.67 (m, 2H), 1.66 – 1.57 (m, 1H), 1.56 – 1.39 (m, 4H), 1.26 (s, 6H), 1.25 (s, 6H);

^{13}C NMR (126 MHz, $CDCl_3$) δ 171.0, 167.2, 133.3, 133.1, 130.5, 129.7, 128.8, 128.5, 128.2, 81.1, 64.7, 31.2, 29.0, 27.1, 25.7, 25.5, 25.2;

^{11}B NMR (160 MHz, $CDCl_3$) δ 18.2;

HRMS (ESI) calcd. for $C_{26}H_{34}BNNaO_5$ $[M+Na]^+$ m/z 474.2422, found 474.2413;

IR (neat, cm^{-1}) 3050, 2970, 2930, 1716, 1610, 1265, 1117, 734;

$[\alpha]_D^{25}$ = –58.1 (c = 0.98, $CHCl_3$);

HPLC analysis: the *ee* (95%) was determined using a CHIRALPAK® IF-3 column, 10% EtOH in hexane, 1.0 mL/min, 254 nm UV detector, t_R (major) = 6.8 min, t_R (minor) = 7.6 min.

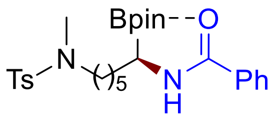

(*R*)-*N*-(6-((*N*,4-Dimethylphenyl)sulfonamido)-1-(4,4,5,5-tetramethyl-1,3,2-dioxaborolan-2-yl)hexyl)benzamide (Figure 3, **3k**). From (*E*)-*N*,4-dimethyl-*N*-(6-(4,4,5,5-tetramethyl-1,3,2-dioxaborolan-2-yl)hex-5-en-1-yl)benzenesulfonamide (**1k**) (78.7 mg, 0.20 mmol, 1.0 equiv) and 3-phenyl-1,4,2-dioxazol-5-one (**2a**) (48.9 mg, 0.30 mmol, 1.5 equiv), the title compound was prepared following the general procedure **A** using $NiCl_2 \cdot 6H_2O$ (4.8 mg, 10 mol%), **L*** (8.0 mg, 12 mol%), LiI (13.4 mg, 0.10 mmol, 0.50 equiv), H_2O (1.8 µL, 0.10 mmol, 0.50 equiv), $(EtO)_3SiH$ (92 µL, 0.50 mmol, 2.5 equiv), anhydrous DMA (1.0 mL). The reaction mixture was stirred for 20 h at 25 °C. The crude material was purified by flash column chromatography (petroleum ether/EtOAc = 3:2) to provide the title compound as a white solid in 57% yield (58.7 mg).

^{1}H NMR (500 MHz, $CDCl_3$) δ 8.10 (s, 1H), 7.92 (d, *J* = 7.5 Hz, 2H), 7.63 (d, *J* = 8.2 Hz, 2H), 7.51 (t, *J* = 7.4 Hz, 1H), 7.39 (t, *J* = 7.7 Hz, 2H), 7.30 (d, *J* = 8.1 Hz, 2H), 3.21 – 3.11 (m, 1H), 2.93 – 2.85 (m, 1H), 2.82 – 2.73 (m, 1H), 2.68 (s, 3H), 2.42 (s, 3H), 1.72 – 1.43 (m, 7H), 1.38 – 1.31 (m, 1H), 1.27 (s, 6H), 1.26 (s, 6H);

^{13}C NMR (126 MHz, $CDCl_3$) δ 171.3, 143.5, 134.4, 133.2, 129.8, 128.7, 128.4, 127.9, 127.4, 80.8, 49.0, 34.5, 30.9, 26.4, 26.0, 25.5, 25.2, 24.8, 21.6;

^{11}B NMR (160 MHz, $CDCl_3$) δ 17.3;

HRMS (ESI) calcd. for $C_{27}H_{39}BN_2NaO_5S$ $[M+Na]^+$ m/z 537.2565, found 537.2556;

IR (neat, cm^{-1}) 3050, 2929, 1610, 1156, 732;

m.p. 123 – 125 °C;

$[\alpha]_D^{25}$ = –25.5 (c = 1.05, $CHCl_3$);

HPLC analysis: the *ee* (97%) was determined using a CHIRALPAK® IG-3 column, 20% EtOH in hexane, 1.0 mL/min, 254 nm UV detector, t_R (major) = 9.7 min, t_R (minor) = 12.3 min.

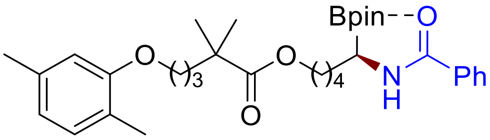

(*R*)-5-Benzamido-5-(4,4,5,5-tetramethyl-1,3,2-dioxaborolan-2-yl)pentyl 5-(2,5-dimethylphenoxy)-2,2-dimethylpentanoate (Figure 3, **3l**). From (*E*)-5-(4,4,5,5-tetramethyl-1,3,2-dioxaborolan-2-yl)pent-4-en-1-yl 5-(2,5-dimethylphenoxy)-2,2-dimethylpentanoate (**1l**) (88.9 mg, 0.20 mmol, 1.0 equiv) and 3-phenyl-1,4,2-dioxazol-5-one (**2a**) (48.9 mg, 0.30 mmol, 1.5 equiv), the title compound was prepared following the general procedure **A** using $NiCl_2·6H_2O$ (4.8 mg, 10 mol%), **L*** (8.0 mg, 12 mol%), LiI (13.4 mg, 0.10 mmol, 0.50 equiv), H_2O (1.8 μL, 0.10 mmol, 0.50 equiv), $(EtO)_3SiH$ (92 μL, 0.50 mmol, 2.5 equiv), anhydrous DMA (1.0 mL). The reaction mixture was stirred for 20 h at 25 °C. The crude material was purified by flash column chromatography (petroleum ether/EtOAc = 3:2) to provide the title compound as a colorless oil in 60% yield (67.9 mg).

^{1}H NMR (500 MHz, $CDCl_3$) δ 8.19 (s, 1H), 7.82 (d, *J* = 7.5 Hz, 2H), 7.50 (t, *J* = 7.4 Hz, 1H), 7.37 (t, *J* = 7.7 Hz, 2H), 6.99 (d, *J* = 7.5 Hz, 1H), 6.65 (d, *J* = 7.5 Hz, 1H), 6.59 (s, 1H), 4.07 (t, *J* = 6.1 Hz, 2H), 3.90 (t, *J* = 5.5 Hz, 2H), 2.83 (t, *J* = 6.0 Hz, 1H), 2.29 (s, 3H), 2.16 (s, 3H), 1.79 – 1.61 (m, 7H), 1.61 – 1.53 (m, 1H), 1.53 – 1.41 (m, 2H), 1.28 (s, 6H), 1.27 (s, 6H), 1.20 (s, 6H);

^{13}C NMR (126 MHz, $CDCl_3$) δ 178.2, 171.1, 157.0, 136.6, 133.3, 130.4, 128.6, 128.3, 127.7, 123.7, 120.8, 112.1, 81.1, 68.1, 64.5, 42.2, 37.2, 31.0, 28.9, 25.4, 25.3, 25.2, 24.1, 21.5, 15.9;

^{11}B NMR (160 MHz, $CDCl_3$) δ 17.9;

HRMS (ESI) calcd. for $C_{33}H_{48}BNNaO_6$ $[M+Na]^+$ m/z 588.3467, found 588.3453;

IR (neat, cm^{-1}) 2925, 1725, 1610, 1151, 1127, 707;

$[\alpha]_D^{25}$ = –30.3 (c = 0.98, $CHCl_3$);

HPLC analysis: the *ee* (96%) was determined using a CHIRALPAK® AD-H column, 8% *i*PrOH in hexane, 0.8 mL/min, 240 nm UV detector, t_R (minor) = 7.0 min, t_R (major) = 8.1 min.

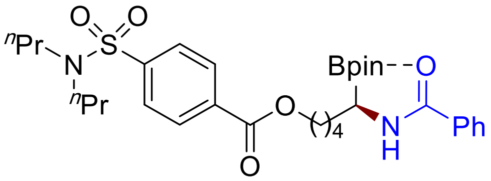

(*R*)-5-Benzamido-5-(4,4,5,5-tetramethyl-1,3,2-dioxaborolan-2-yl)pentyl 4-(*N*,*N*-dipropylsulfamoyl)benzoate (Figure 3, **3m**). From (*E*)-5-(4,4,5,5-tetramethyl-1,3,2-dioxaborolan-2-yl)pent-4-en-1-yl 4-(*N*,*N*-dipropylsulfamoyl)benzoate (**1m**) (95.9 mg, 0.20 mmol, 1.0 equiv) and 3-phenyl-1,4,2-dioxazol-5-one (**2a**) (48.9 mg, 0.30 mmol, 1.5 equiv), the title compound was prepared following the general procedure **A** using $NiCl_2 \cdot 6H_2O$ (4.8 mg, 10 mol%), **L*** (8.0 mg, 12 mol%), LiI (13.4 mg, 0.10 mmol, 0.50 equiv), H_2O (1.8 μL, 0.10 mmol, 0.50 equiv), $(EtO)_3SiH$ (92 μL, 0.50 mmol, 2.5 equiv), anhydrous DMA (1.0 mL). The reaction mixture was stirred for 20 h at 25 °C. The crude material was purified by flash column chromatography (petroleum ether/EtOAc = 2:3) to provide the title compound as a colorless oil in 60% yield (72.2 mg).

^{1}H NMR (500 MHz, $CDCl_3$) δ 8.13 (d, *J* = 8.4 Hz, 2H), 7.84 (d, *J* = 8.4 Hz, 2H), 7.82 (d, *J* = 7.4 Hz, 2H), 7.55 (t, *J* = 7.4 Hz, 1H), 7.52 (s, 1H), 7.42 (t, *J* = 7.7 Hz, 2H), 4.37 (t, *J* = 6.5 Hz, 2H), 3.13 – 3.04 (m, 4H), 2.98 – 2.88 (m, 1H), 1.89 – 1.75 (m, 3H), 1.69 – 1.49 (m, 7H), 1.26 (s, 6H), 1.26 (s, 6H), 0.86 (t, *J* = 7.4 Hz, 6H);

^{13}C NMR (126 MHz, $CDCl_3$) δ 170.9, 165.6, 144.3, 133.8, 133.3, 130.3, 128.8, 128.0, 127.1, 81.5, 65.7, 50.1, 31.1, 28.9, 25.4, 25.2, 24.2, 22.1, 11.3;

^{11}B NMR (160 MHz, $CDCl_3$) δ 18.0;

HRMS (ESI) calcd. for $C_{31}H_{45}BN_2NaO_7S$ $[M+Na]^+$ m/z 623.2933, found 623.2923;

IR (neat, cm^{-1}) 2967, 2932, 1721, 1609, 1272, 1155, 1087, 601;

$[\alpha]_D^{25}$ = –30.7 (c = 1.04, $CHCl_3$);

HPLC analysis: the *ee* (96%) was determined using a CHIRALPAK® ID-3 column, 15% EtOH in hexane, 1.0 mL/min, 254 nm UV detector, t_R (major) = 9.2 min, t_R (minor) = 13.5 min.

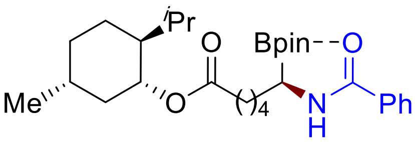

(1*R*,2*S*,5*R*)-2-isopropyl-5-methylcyclohexyl (*R*)-6-benzamido-6-(4,4,5,5-tetramethyl-1,3,2-dioxaborolan-2-yl)hexanoate (Figure 3, **3n**). From (1*R*,2*S*,5*R*)-2-isopropyl-5-methylcyclohexyl (*E*)-6-(4,4,5,5-tetramethyl-1,3,2-dioxaborolan-2-yl)hex-5-enoate (**1n**) (75.7 mg, 0.20 mmol, 1.0 equiv) and 3-phenyl-1,4,2-dioxazol-5-one (**2a**) (48.9 mg, 0.30 mmol, 1.5 equiv), the title compound was prepared following the general procedure **A** using $NiCl_2 \cdot 6H_2O$ (4.8 mg, 10 mol%), **L*** (8.0 mg, 12 mol%), LiI (13.4 mg, 0.10 mmol, 0.50 equiv), H_2O (1.8 μL, 0.10 mmol, 0.50 equiv), $(EtO)_3SiH$ (92 μL, 0.50 mmol, 2.5 equiv), anhydrous DMA (1.0 mL). The reaction mixture was stirred for 20 h at 25 °C. The crude material was purified by flash column chromatography (petroleum ether/EtOAc = 2:1) to provide the title compound as a colorless oil in 58% yield (58.1 mg).

^{1}H NMR (500 MHz, $CDCl_3$) δ 8.44 (s, 1H), 7.90 (d, *J* = 7.5 Hz, 2H), 7.53 (t, *J* = 7.4 Hz, 1H), 7.40 (t, *J* = 7.7 Hz, 2H), 4.69 (td, *J* = 10.9, 4.3 Hz, 1H), 2.93 – 2.84 (m, 1H), 2.43 – 2.22 (m, 2H), 2.00 – 1.92 (m, 1H), 1.90 – 1.80 (m, 1H), 1.78 – 1.62 (m, 5H), 1.62 – 1.54 (m, 1H), 1.54 – 1.41 (m, 3H), 1.41 – 1.32 (m, 1H), 1.27 (s, 6H), 1.27 (s, 6H), 1.10 – 0.92 (m, 2H), 0.89 (d, *J* = 3.9 Hz, 3H), 0.89 – 0.81 (m, 4H), 0.76 (d, *J* = 7.0 Hz, 3H);

^{13}C NMR (126 MHz, $CDCl_3$) δ 174.4, 171.2, 133.3, 128.7, 128.3, 127.8, 81.0, 74.5, 47.1, 41.1, 34.4, 34.3, 31.6, 30.0, 27.1, 26.4, 25.4, 25.2, 24.0, 23.6, 22.2, 20.8, 16.4;

^{11}B NMR (160 MHz, $CDCl_3$) δ 17.2;

HRMS (ESI) calcd. for $C_{29}H_{46}BNNaO_5$ $[M+Na]^+$ m/z 500.3542, found 500.3531;

IR (neat, cm^{-1}) 2957, 2928, 1724, 1610, 1113;

$[\alpha]_D^{25}$ = –59.2 (c = 1.01, $CHCl_3$);

HPLC analysis: the *dr* (98:2) was determined using a CHIRALPAK® AD-H column, 5% *i*PrOH in hexane, 1.0 mL/min, 240 nm UV detector, t_R (major) = 5.7 min, t_R (minor) = 8.6 min.

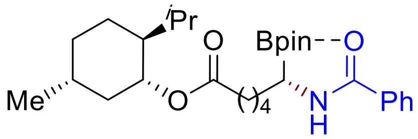

(1*R*,2*S*,5*R*)-2-isopropyl-5-methylcyclohexyl (*S*)-6-benzamido-6-(4,4,5,5-tetramethyl-1,3,2-dioxaborolan-2-yl)hexanoate (Figure 3, **3n'**). From (1*R*,2*S*,5*R*)-2-isopropyl-5-methylcyclohexyl (*E*)-6-(4,4,5,5-tetramethyl-1,3,2-dioxaborolan-2-yl)hex-5-enoate (**1n**) (75.7 mg, 0.20 mmol, 1.0 equiv) and 3-phenyl-1,4,2-dioxazol-5-one (**2a**) (48.9 mg, 0.30 mmol, 1.5 equiv), the title compound was prepared following the general procedure **A** using $NiCl_2 \cdot 6H_2O$ (4.8 mg, 10 mol%), *ent*-**L*** (8.0 mg, 12 mol%), LiI (13.4 mg, 0.10 mmol, 0.50 equiv), H_2O (1.8 μL, 0.10 mmol, 0.50 equiv), $(EtO)_3SiH$ (92 μL, 0.50 mmol, 2.5 equiv), anhydrous DMA (1.0 mL). The reaction mixture was stirred for 20 h at 25 °C. The crude material was purified by flash column chromatography (petroleum ether/EtOAc = 2:1) to provide the title compound as a colorless oil in 55% yield (55.0 mg).

^{1}H NMR (500 MHz, $CDCl_3$) δ 8.41 (s, 1H), 7.89 (d, *J* = 7.6 Hz, 2H), 7.53 (t, *J* = 7.4 Hz, 1H), 7.40 (t, *J* = 7.7 Hz, 2H), 4.71 (td, *J* = 10.9, 4.4 Hz, 1H), 2.94 – 2.86 (m, 1H), 2.43 – 2.22 (m, 2H), 2.02 – 1.92 (m, 1H), 1.87 – 1.78 (m, 1H), 1.79 – 1.62 (m, 5H), 1.62 – 1.54 (m, 1H), 1.54 – 1.40 (m, 3H), 1.40 – 1.32 (m, 1H), 1.27 (s, 6H), 1.26 (s, 6H), 1.11 – 0.93 (m, 2H), 0.90 (d, *J* = 6.5 Hz, 3H), 0.88 – 0.81 (m, 4H), 0.73 (d, *J* = 6.9 Hz, 3H);

^{13}C NMR (126 MHz, $CDCl_3$) δ 174.4, 171.2, 133.3, 128.7, 128.3, 127.8, 81.0, 74.6, 47.1, 41.1, 34.4, 34.3, 31.6, 30.0, 27.1, 26.4, 25.4, 25.1, 23.9, 23.6, 22.2, 20.9, 16.5;

^{11}B NMR (160 MHz, $CDCl_3$) δ 17.2;

HRMS (ESI) calcd. for $C_{29}H_{46}BNNaO_5$ $[M+Na]^+$ m/z 500.3542, found 500.3532;

IR (neat, cm^{-1}) 2954, 2926, 1711, 1603, 1106;

$[\alpha]_D^{25}$ = +7.1 (c = 0.98, $CHCl_3$);

HPLC analysis: the *dr* (2:98) was determined using a CHIRALPAK® AD-H column, 5% *i*PrOH in hexane, 1.0 mL/min, 240 nm UV detector, t_R (minor) = 5.7 min, t_R (major) = 8.6 min.

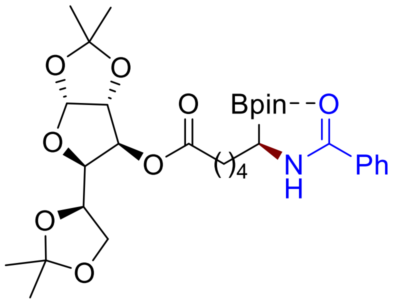

(3a*R*,5*R*,6*S*,6a*R*)-5-((*R*)-2,2-dimethyl-1,3-dioxolan-4-yl)-2,2-dimethyltetrahydrofuro[2,3-*d*][1,3]dioxol-6-yl (*R*)-6-benzamido-6-(4,4,5,5-tetramethyl-1,3,2-dioxaborolan-2-yl)hexanoate (Figure 3, **3o**). From (3a*R*,5*R*,6*S*,6a*R*)-5-((*R*)-2,2-dimethyl-1,3-dioxolan-4-yl)-2,2-dimethyltetrahydrofuro[2,3-*d*][1,3]dioxol-6-yl (*E*)-6-(4,4,5,5-tetramethyl-1,3,2-dioxaborolan-2-yl)hex-5-enoate (**1o**) (96.5 mg, 0.20 mmol, 1.0 equiv) and 3-phenyl-1,4,2-dioxazol-5-one (**2a**) (48.9 mg, 0.30 mmol, 1.5 equiv), the title compound was prepared following the general procedure **A** using $NiCl_2·6H_2O$ (4.8 mg, 10 mol%), **L*** (8.0 mg, 12 mol%), LiI (13.4 mg, 0.10 mmol, 0.50 equiv), H_2O (1.8 μL, 0.10 mmol, 0.50 equiv), $(EtO)_3SiH$ (92 μL, 0.50 mmol, 2.5 equiv), anhydrous DMA (1.0 mL). The reaction mixture was stirred for 20 h at 25 °C. The crude material was purified by flash column chromatography (petroleum ether/EtOAc = 1:1) to provide the title compound as a colorless oil in 58% yield (70.3 mg).

^{1}H NMR (500 MHz, $CDCl_3$) δ 8.22 (s, 1H), 7.87 (d, *J* = 7.3 Hz, 2H), 7.53 (t, *J* = 7.4 Hz, 1H), 7.41 (t, *J* = 7.8 Hz, 2H), 5.87 (d, *J* = 3.6 Hz, 1H), 5.30 (d, *J* = 2.7 Hz, 1H), 4.49 (d, *J* = 3.7 Hz, 1H), 4.23 – 4.14 (m, 2H), 4.10 – 4.04 (m, 1H), 4.02 – 3.96 (m, 1H), 2.92 – 2.84 (m, 1H), 2.47 – 2.32 (m, 2H), 1.80 – 1.56 (m, 4H), 1.56 – 1.41 (m, 5H), 1.34 (s, 3H), 1.30 (s, 3H), 1.29 – 1.24 (m, 15H);

^{13}C NMR (126 MHz, $CDCl_3$) δ 173.1, 171.2, 133.4, 128.8, 128.3, 127.7, 112.4, 109.5, 105.2, 83.5, 81.1, 80.1, 76.2, 72.6, 67.4, 34.0, 30.3, 26.9, 26.9, 26.4, 25.4, 25.4, 25.1, 23.9;

^{11}B NMR (160 MHz, $CDCl_3$) δ 17.7;

HRMS (ESI) calcd. for $C_{31}H_{46}BNNaO_{10}$ $[M+Na]^+$ m/z 626.3107, found 626.3105;

IR (neat, cm^{-1}) 2986, 2933, 1745, 1610, 1373, 1155;

[α]$_D^{25}$ = –46.6 (c = 1.00, $CHCl_3$);

HPLC analysis: the *dr* (97:3) was determined using a CHIRALPAK® IF-3 column, 12% EtOH in hexane, 1.0 mL/min, 240 nm UV detector, t_R (major) = 6.8 min, t_R (minor) = 7.5 min.

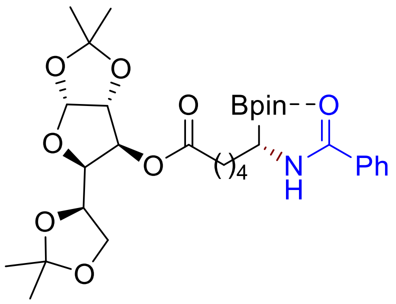

(3a*R*,5*R*,6*S*,6a*R*)-5-((*R*)-2,2-dimethyl-1,3-dioxolan-4-yl)-2,2-dimethyltetrahydrofuro[2,3-*d*][1,3]dioxol-6-yl (*S*)-6-benzamido-6-(4,4,5,5-tetramethyl-1,3,2-dioxaborolan-2-yl)hexanoate (Figure 3, **3o'**). From (3a*R*,5*R*,6*S*,6a*R*)-5-((*R*)-2,2-dimethyl-1,3-dioxolan-4-yl)-2,2-dimethyltetrahydrofuro[2,3-*d*][1,3]dioxol-6-yl (*E*)-6-(4,4,5,5-tetramethyl-1,3,2-dioxaborolan-2-yl)hex-5-enoate (**1o**) (96.5 mg, 0.20 mmol, 1.0 equiv) and 3-phenyl-1,4,2-dioxazol-5-one (**2a**) (48.9 mg, 0.30 mmol, 1.5 equiv), the title compound was prepared following the general procedure **A** using $NiCl_2·6H_2O$ (4.8 mg, 10 mol%), *ent*-**L*** (8.0 mg, 12 mol%), LiI (13.4 mg, 0.10 mmol, 0.50 equiv), H_2O (1.8 μL, 0.10 mmol, 0.50 equiv), $(EtO)_3SiH$ (92 μL, 0.50 mmol, 2.5 equiv), anhydrous DMA (1.0 mL). The reaction mixture was stirred for 20 h at 25 °C. The crude material was purified by flash column chromatography (petroleum ether/EtOAc = 1:1) to provide the title compound as a colorless oil in 61% yield (73.4 mg).

^{1}H NMR (500 MHz, $CDCl_3$) δ 8.24 (s, 1H), 7.87 (d, *J* = 7.3 Hz, 2H), 7.53 (t, *J* = 7.5 Hz, 1H), 7.42 (t, *J* = 7.8 Hz, 2H), 5.87 (d, *J* = 3.7 Hz, 1H), 5.28 (d, *J* = 2.0 Hz, 1H), 4.47 (d, *J* = 3.7 Hz, 1H), 4.24 – 4.17 (m, 2H), 4.11 – 4.05 (m, 1H), 4.04 – 3.98 (m, 1H), 2.91 – 2.82 (m, 1H), 2.49 – 2.30 (m, 2H), 1.80 – 1.56 (m, 4H), 1.55 – 1.42 (m, 5H), 1.39 (s, 3H), 1.30 (s, 3H), 1.29 (s, 3H), 1.27 (s, 6H), 1.26 (s, 6H);

^{13}C NMR (126 MHz, $CDCl_3$) δ 173.2, 171.2, 133.4, 128.8, 128.3, 127.8, 112.4, 109.5,

105.2, 83.6, 81.1, 79.9, 76.3, 72.5, 67.4, 33.9, 30.2, 27.0, 27.0, 26.9, 26.4, 25.4, 25.4, 25.1, 24.0;

^{11}B NMR (160 MHz, $CDCl_3$) δ 17.5;

HRMS (ESI) calcd. for $C_{31}H_{46}BNNaO_{10}$ $[M+Na]^+$ m/z 626.3107, found 626.3109;

IR (neat, cm^{-1}) 2987, 2934, 1746, 1610, 1373, 1157;

$[\alpha]_D^{25}$ = +14.1 (c = 1.00, $CHCl_3$);

HPLC analysis: the *dr* (3:97) was determined using a CHIRALPAK® IF-3 column, 12% EtOH in hexane, 1.0 mL/min, 240 nm UV detector, t_R (minor) = 6.7 min, t_R (major) = 7.5 min.

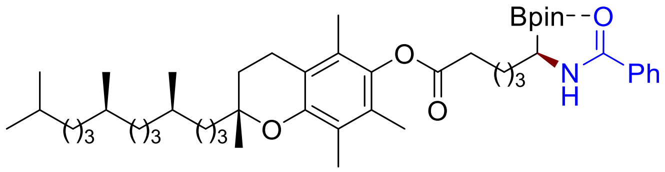

(*R*)-2,5,7,8-tetramethyl-2-((4*R*,8*R*)-4,8,12-trimethyltridecyl)chroman-6-yl (*R*)-6-benzamido-6-(4,4,5,5-tetramethyl-1,3,2-dioxaborolan-2-yl)hexanoate (Figure 3, **3p**). From (*R*)-2,5,7,8-tetramethyl-2-((4*R*,8*R*)-4,8,12-trimethyltridecyl)chroman-6-yl (*E*)-6-(4,4,5,5-tetramethyl-1,3,2-dioxaborolan-2-yl)hex-5-enoate (**1p**) (130.6 mg, 0.20 mmol, 1.0 equiv) and 3-phenyl-1,4,2-dioxazol-5-one (**2a**) (48.9 mg, 0.30 mmol, 1.5 equiv), the title compound was prepared following the general procedure **A** using $NiCl_2 \cdot 6H_2O$ (4.8 mg, 10 mol%), **L*** (8.0 mg, 12 mol%), LiI (13.4 mg, 0.10 mmol, 0.50 equiv), H_2O (1.8 μL, 0.10 mmol, 0.50 equiv), $(EtO)_3SiH$ (92 μL, 0.50 mmol, 2.5 equiv), anhydrous DMA (1.0 mL). The reaction mixture was stirred for 20 h at 25 °C. The crude material was purified by flash column chromatography (petroleum ether/EtOAc = 5:2) to provide the title compound as a colorless oil in 57% yield (88.7 mg).

^{1}H NMR (500 MHz, $CDCl_3$) δ 8.44 (s, 1H), 7.81 (d, *J* = 7.6 Hz, 2H), 7.49 (t, *J* = 7.4 Hz, 1H), 7.32 (t, *J* = 7.6 Hz, 2H), 2.91 – 2.82 (m, 1H), 2.73 – 2.51 (m, 4H), 2.08 (s, 3H), 2.02 – 1.84 (m, 7H), 1.84 – 1.65 (m, 5H), 1.64 – 1.47 (m, 5H), 1.45 – 1.33 (m, 4H), 1.32 – 1.18 (m, 23H), 1.17 – 1.01 (m, 6H), 0.90 – 0.79 (m, 12H);

^{13}C NMR (126 MHz, $CDCl_3$) δ 173.4, 171.2, 149.5, 140.6, 133.3, 128.7, 128.3, 127.6,

126.7, 124.9, 123.1, 117.5, 80.9, 75.2, 39.5, 37.6, 37.6, 37.4, 33.9, 32.9, 32.8, 31.2, 30.3, 28.1, 27.2, 25.4, 25.2, 24.9, 24.6, 24.2, 22.9, 22.8, 21.2, 20.7, 19.9, 19.8, 13.2, 12.3, 12.0;

^{11}B NMR (160 MHz, $CDCl_3$) δ 17.2;

HRMS (ESI) calcd. for $C_{48}H_{76}BNNaO_6$ $[M+Na]^+$ m/z 796.5658, found 796.5652;

IR (neat, cm^{-1}) 2925, 2866, 1751, 1610, 1110;

$[\alpha]_D^{25}$ = –19.2 (c = 1.01, $CHCl_3$);

HPLC analysis: the *dr* (98:2) was determined using a CHIRALPAK® ID-3 column, 10% EtOH in hexane, 1.0 mL/min, 240 nm UV detector, t_R (major) = 4.9 min, t_R (minor) = 5.7 min.

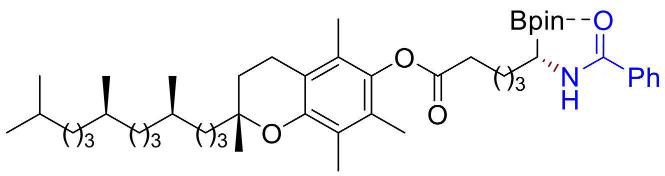

(*R*)-2,5,7,8-tetramethyl-2-((4*R*,8*R*)-4,8,12-trimethyltridecyl)chroman-6-yl (*S*)-6-benzamido-6-(4,4,5,5-tetramethyl-1,3,2-dioxaborolan-2-yl)hexanoate (Figure 3, **3p'**). From (*R*)-2,5,7,8-tetramethyl-2-((4*R*,8*R*)-4,8,12-trimethyltridecyl)chroman-6-yl (*E*)-6-(4,4,5,5-tetramethyl-1,3,2-dioxaborolan-2-yl)hex-5-enoate (**1p**) (130.6 mg, 0.20 mmol, 1.0 equiv) and 3-phenyl-1,4,2-dioxazol-5-one (**2a**) (48.9 mg, 0.30 mmol, 1.5 equiv), the title compound was prepared following the general procedure **A** using $NiCl_2{\cdot}6H_2O$ (4.8 mg, 10 mol%), *ent*-**L*** (8.0 mg, 12 mol%), LiI (13.4 mg, 0.10 mmol, 0.50 equiv), H_2O (1.8 μL, 0.10 mmol, 0.50 equiv), $(EtO)_3SiH$ (92 μL, 0.50 mmol, 2.5 equiv), anhydrous DMA (1.0 mL). The reaction mixture was stirred for 20 h at 25 °C. The crude material was purified by flash column chromatography (petroleum ether/EtOAc = 5:2) to provide the title compound as a colorless oil in 58% yield (90.5 mg).

^{1}H NMR (500 MHz, $CDCl_3$) δ 8.52 (s, 1H), 7.81 (d, *J* = 7.7 Hz, 2H), 7.48 (t, *J* = 7.4 Hz, 1H), 7.31 (t, *J* = 7.6 Hz, 2H), 2.90 – 2.82 (m, 1H), 2.72 – 2.51 (m, 4H), 2.08 (s, 3H), 2.03 – 1.84 (m, 7H), 1.84 – 1.65 (m, 5H), 1.63 –1.46 (m, 5H), 1.44 – 1.32 (m, 4H),

1.31 – 1.20 (m, 23H), 1.16 – 1.03 (m, 6H), 0.89 – 0.81 (m, 12H);

^{13}C NMR (126 MHz, $CDCl_3$) δ 173.4, 171.2, 149.5, 140.6, 133.3, 128.6, 128.3, 127.5, 126.7, 124.9, 123.1, 117.5, 80.9, 75.2, 39.5, 37.6, 37.6, 37.4, 33.9, 32.9, 32.8, 31.2, 30.3, 28.1, 27.2, 25.4, 25.2, 24.9, 24.6, 24.2, 22.9, 22.8, 21.2, 20.7, 19.9, 19.8, 13.2, 12.3, 12.0;

^{11}B NMR (160 MHz, $CDCl_3$) δ 17.1;

HRMS (ESI) calcd. for $C_{48}H_{76}BNNaO_6$ $[M+Na]^+$ m/z 796.5658, found 796.5654;

IR (neat, cm^{-1}) 2926, 2867, 1750, 1610, 1110;

$[\alpha]_D^{25}$ = +26.7 (c = 1.07, $CHCl_3$);

HPLC analysis: the *dr* (2:98) was determined using a CHIRALPAK® ID-3 column, 10% EtOH in hexane, 1.0 mL/min, 240 nm UV detector, t_R (major) = 4.9 min, t_R (minor) = 5.7 min.

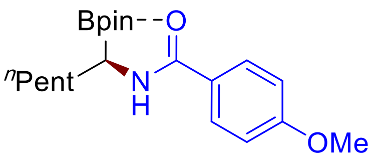

(*R*)-4-Methoxy-*N*-(1-(4,4,5,5-tetramethyl-1,3,2-dioxaborolan-2-yl)hexyl) benzamide (Figure 4, **4b**). From (*E*)-2-(hex-1-en-1-yl)-4,4,5,5-tetramethyl-1,3,2-dioxaborolane (**1a**) (42.0 mg, 0.20 mmol, 1.0 equiv) and 3-(4-methoxyphenyl)-1,4,2-dioxazol-5-one (**2b**) (57.9 mg, 0.30 mmol, 1.5 equiv), the title compound was prepared following the general procedure **A** using $NiCl_2 \cdot 6H_2O$ (4.8 mg, 10 mol%), **L*** (8.0 mg, 12 mol%), LiI (13.4 mg, 0.10 mmol, 0.50 equiv), H_2O (1.8 μL, 0.10 mmol, 0.50 equiv), $(EtO)_3SiH$ (92 μL, 0.50 mmol, 2.5 equiv), anhydrous DMA (1.0 mL). The reaction mixture was stirred for 20 h at 25 °C. The crude material was purified by flash column chromatography (petroleum ether/EtOAc = 1:1) to provide the title compound as a white solid in 72% yield (51.8 mg).

^{1}H NMR (500 MHz, $CDCl_3$) δ 8.52 (s, 1H), 7.78 (d, *J* = 8.9 Hz, 2H), 6.79 (d, *J* = 8.9 Hz, 2H), 3.80 (s, 3H), 2.69 (t, *J* = 6.6 Hz, 1H), 1.72 – 1.63 (m, 1H), 1.58 – 1.48 (m, 1H), 1.47 – 1.36 (m, 2H), 1.35 – 1.27 (m, 4H), 1.26 (s, 6H), 1.25 (s, 6H), 0.88 (t, *J* = 7.0 Hz, 3H);

^{13}**C NMR** (126 MHz, $CDCl_3$) δ 170.7, 163.6, 130.5, 119.4, 113.8, 80.7, 55.5, 32.2, 31.4, 27.7, 25.5, 25.3, 22.7, 14.3;

^{11}**B NMR** (160 MHz, $CDCl_3$) δ 15.8;

HRMS (ESI) calcd. for $C_{20}H_{33}BNO_4$ $[M+H]^+$ m/z 362.2497, found 362.2489;

IR (neat, cm^{-1}) 3064, 2925, 2854, 1609, 1497, 1260, 1108;

m.p. 166 – 168 °C;

$[\alpha]_D^{25}$ = –59.2 (c = 0.98, $CHCl_3$);

HPLC analysis: the *ee* (95%) was determined using a CHIRALPAK® IG-3 column, 5% EtOH in hexane, 1.0 mL/min, 254 nm UV detector, t_R (major) = 7.2 min, t_R (minor) = 8.1 min.

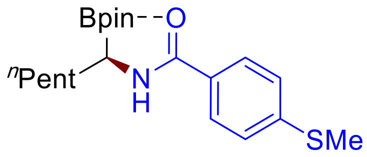

(*R*)-4-(Methylthio)-*N*-(1-(4,4,5,5-tetramethyl-1,3,2-dioxaborolan-2-yl)hexyl)benzamide (Figure 4, **4c**). From (*E*)-2-(hex-1-en-1-yl)-4,4,5,5-tetramethyl-1,3,2-dioxaborolane (**1a**) (42.0 mg, 0.20 mmol, 1.0 equiv) and 3-(4-(methylthio)phenyl)-1,4,2-dioxazol-5-one (**2c**) (62.8 mg, 0.30 mmol, 1.5 equiv), the title compound was prepared following the general procedure **A** using $NiCl_2 \cdot 6H_2O$ (4.8 mg, 10 mol%), **L*** (8.0 mg, 12 mol%), LiI (13.4 mg, 0.10 mmol, 0.50 equiv), H_2O (1.8 μL, 0.10 mmol, 0.50 equiv), $(EtO)_3SiH$ (92 μL, 0.50 mmol, 2.5 equiv), anhydrous DMA (1.0 mL). The reaction mixture was stirred for 20 h at 25 °C. The crude material was purified by flash column chromatography (petroleum ether/EtOAc = 2:1) to provide the title compound as a white solid in 61% yield (46.0 mg).

^{1}**H NMR** (500 MHz, $CDCl_3$) δ 8.68 (s, 1H), 7.70 (d, *J* = 8.5 Hz, 2H), 7.10 (d, *J* = 8.5 Hz, 2H), 2.73 (t, *J* = 6.5 Hz, 1H), 2.47 (s, 3H), 1.74 – 1.63 (m, 1H), 1.59 – 1.49 (m, 1H), 1.48 – 1.37 (m, 2H), 1.35 – 1.27 (m, 4H), 1.27 (s, 12H), 0.88 (t, *J* = 7.0 Hz, 3H);

^{13}**C NMR** (126 MHz, $CDCl_3$) δ 170.5, 146.2, 128.7, 124.9, 123.2, 81.0, 32.2, 31.4, 27.6, 25.4, 25.3, 22.7, 14.8, 14.2;

^{11}**B NMR** (160 MHz, $CDCl_3$) δ 16.6;

HRMS (ESI) calcd. for $C_{20}H_{33}BNO_3S$ $[M+H]^+$ m/z 378.2269, found 378.2260;

IR (neat, cm^{-1}) 3205, 2969, 2929, 1602, 1547, 1115, 733;

m.p. 166 – 167 °C;

$[\alpha]_D^{25}$ = –59.5 (c = 1.16, $CHCl_3$);

HPLC analysis: the *ee* (93%) was determined using a CHIRALPAK® IE-3 column, 5% EtOH in hexane, 1.0 mL/min, 220 nm UV detector, t_R (major) = 9.7 min, t_R (minor) = 10.8 min.

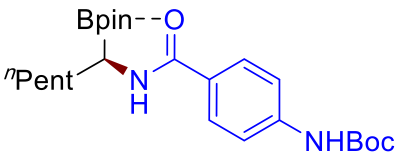

tert*-Butyl (*R*)-(4-((1-(4,4,5,5-tetramethyl-1,3,2-dioxaborolan-2-yl)hexyl)carbamoyl)phenyl)carbamate** (Figure 4, **4d**). From (*E*)-2-(hex-1-en-1-yl)-4,4,5,5-tetramethyl-1,3,2-dioxaborolane (**1a**) (42.0 mg, 0.20 mmol, 1.0 equiv) and *tert*-butyl (4-(5-oxo-1,4,2-dioxazol-3-yl)phenyl)carbamate (**2d**) (83.5 mg, 0.30 mmol, 1.5 equiv), the title compound was prepared following the general procedure **A** using $NiCl_2 \cdot 6H_2O$ (4.8 mg, 10 mol%), **L (8.0 mg, 12 mol%), LiI (13.4 mg, 0.10 mmol, 0.50 equiv), H_2O (1.8 μL, 0.10 mmol, 0.50 equiv), $(EtO)_3SiH$ (92 μL, 0.50 mmol, 2.5 equiv), anhydrous DMA (1.0 mL). The reaction mixture was stirred for 20 h at 25 °C. The crude material was purified by flash column chromatography (petroleum ether/EtOAc = 2:1) to provide the title compound as a white solid in 67% yield (59.8 mg).

^{1}H NMR (500 MHz, $CDCl_3$) δ 8.14 (s, 1H), 7.57 (d, *J* = 8.4 Hz, 2H), 7.40 (d, *J* = 8.6 Hz, 2H), 2.77 – 2.67 (m, 1H), 1.76 – 1.65 (m, 1H), 1.56 – 1.38 (m, 12H), 1.36 – 1.24 (m, 16H), 0.85 (t, *J* = 6.4 Hz, 3H);

^{13}C NMR (126 MHz, $CDCl_3$) δ 170.5, 153.2, 143.7, 129.4, 120.3, 117.7, 81.2, 80.6, 32.1, 31.4, 28.5, 28.1, 25.5, 25.4, 22.7, 14.2;

^{11}B NMR (160 MHz, $CDCl_3$) δ 15.5;

HRMS (ESI) calcd. for $C_{24}H_{39}BN_2NaO_5$ $[M+Na]^+$ m/z 469.2844, found 469.2834;

IR (neat, cm^{-1}) 3224, 2929, 1733, 1605, 1520, 1154, 1096, 737;

m.p. 204 – 205 °C;

$[\alpha]_D^{25}$ = –37.7 (c = 1.10, $CHCl_3$);

HPLC analysis: the *ee* (94%) was determined using a CHIRALPAK® IF-3 column, 5% EtOH in hexane, 1.0 mL/min, 220 nm UV detector, t_R (major) = 5.7 min, t_R (minor) = 6.3 min.

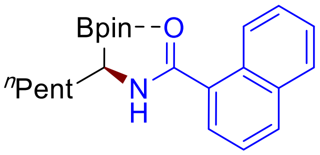

(*R*)-*N*-(1-(4,4,5,5-tetramethyl-1,3,2-dioxaborolan-2-yl)hexyl)-1-naphthamide

(Figure 4, **4e**). From (*E*)-2-(hex-1-en-1-yl)-4,4,5,5-tetramethyl-1,3,2-dioxaborolane (**1a**) (42.0 mg, 0.20 mmol, 1.0 equiv) and 3-(naphthalen-1-yl)-1,4,2-dioxazol-5-one (**2e**) (64.0 mg, 0.30 mmol, 1.5 equiv), the title compound was prepared following the general procedure **A** using $NiCl_2·6H_2O$ (4.8 mg, 10 mol%), **L*** (8.0 mg, 12 mol%), LiI (13.4 mg, 0.10 mmol, 0.50 equiv), H_2O (1.8 μL, 0.10 mmol, 0.50 equiv), $(EtO)_3SiH$ (92 μL, 0.50 mmol, 2.5 equiv), anhydrous DMA (1.0 mL). The reaction mixture was stirred for 20 h at 25 °C. The crude material was purified by flash column chromatography (petroleum ether/EtOAc = 4:1) to provide the title compound as a white solid in 60% yield (45.7 mg).

^{1}H NMR (500 MHz, $CDCl_3$) δ 8.39 (d, *J* = 8.0 Hz, 1H), 7.93 (d, *J* = 8.2 Hz, 1H), 7.88 – 7.84 (m, 1H), 7.67 (d, *J* = 6.9 Hz, 1H), 7.58 – 7.49 (m, 2H), 7.46 – 7.41 (m, 1H), 6.88 (s, 1H), 3.17 – 3.09 (m, 1H), 1.82 – 1.73 (m, 1H), 1.70 – 1.60 (m, 1H), 1.50 – 1.39 (m, 2H), 1.39 – 1.32 (m, 4H), 1.30 (s, 12H), 0.89 (t, *J* = 7.0 Hz, 3H);

^{13}C NMR (126 MHz, $CDCl_3$) δ 172.1, 133.7, 132.0, 130.4, 130.0, 128.5, 127.6, 126.7, 126.4, 125.5, 124.7, 82.4, 32.0, 31.3, 27.6, 25.3, 25.2, 22.7, 14.2;

^{11}B NMR (160 MHz, $CDCl_3$) δ 23.9;

HRMS (ESI) calcd. for $C_{23}H_{33}BNO_3$ $[M+H]^+$ m/z 382.2548, found 382.2542;

IR (neat, cm^{-1}) 3176, 3065, 2931, 1579, 1532, 1191, 1127, 779;

m.p. 53 – 55 ° Ç

$[\alpha]_D^{25}$ = –34.4 (c = 0.62, $CHCl_3$);

HPLC analysis: the *ee* (84%) was determined using a CHIRALPAK® ID-3 column, 5%

EtOH in hexane, 1.0 mL/min, 240 nm UV detector, t_R (major) = 4.7 min, t_R (minor) = 5.1 min.

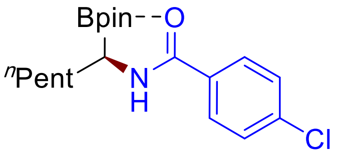

(*R*)-4-Chloro-*N*-(1-(4,4,5,5-tetramethyl-1,3,2-dioxaborolan-2-yl)hexyl)benzamide (Figure 4, **4f**). From (*E*)-2-(hex-1-en-1-yl)-4,4,5,5-tetramethyl-1,3,2-dioxaborolane (**1a**) (42.0 mg, 0.20 mmol, 1.0 equiv) and 3-(4-chlorophenyl)-1,4,2-dioxazol-5-one (**2f**) (59.3 mg, 0.30 mmol, 1.5 equiv), the title compound was prepared following the general procedure **A** using $NiCl_2·6H_2O$ (4.8 mg, 10 mol%), **L*** (8.0 mg, 12 mol%), LiI (13.4 mg, 0.10 mmol, 0.50 equiv), H_2O (1.8 μL, 0.10 mmol, 0.50 equiv), $(EtO)_3SiH$ (92 μL, 0.50 mmol, 2.5 equiv), anhydrous DMA (1.0 mL). The reaction mixture was stirred for 20 h at 25 °C. The crude material was purified by flash column chromatography (petroleum ether/EtOAc = 2:1) to provide the title compound as a colorless oil in 62% yield (45.7 mg).

^{1}H NMR (500 MHz, $CDCl_3$) δ 8.57 (s, 1H), 7.74 (d, *J* = 8.6 Hz, 2H), 7.29 (d, *J* = 8.6 Hz, 2H), 2.85 – 2.78 (m, 1H), 1.77 – 1.64 (m, 1H), 1.62 – 1.51 (m, 1H), 1.46 – 1.36 (m, 2H), 1.35 – 1.28 (m, 4H), 1.26 (s, 12H), 0.88 (t, *J* = 7.0 Hz, 3H);

^{13}C NMR (126 MHz, $CDCl_3$) δ 169.5, 139.6, 129.7, 128.9, 126.6, 81.7, 32.1, 31.3, 27.5, 25.3, 25.2, 22.7, 14.2;

^{11}B NMR (160 MHz, $CDCl_3$) δ 19.1;

HRMS (ESI) calcd. for $C_{19}H_{29}BClNNaO_3$ $[M+Na]^+$ m/z 388.1821, found 388.1811;

IR (neat, cm^{-1}) 3066, 2969, 2927, 1606, 1485, 1092;

$[\alpha]_D^{25}$ = –47.6 (c = 1.09, $CHCl_3$);

HPLC analysis: the *ee* (96%) was determined using a CHIRALPAK® AS-H column, 3% *i*PrOH in hexane, 0.4 mL/min, 254 nm UV detector, t_R (minor) = 9.3 min, t_R (major) = 10.3 min.

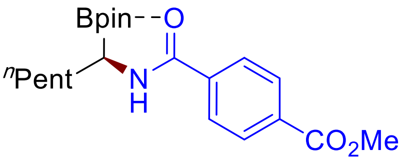

Methyl (*R*)-4-((1-(4,4,5,5-tetramethyl-1,3,2-dioxaborolan-2-yl)hexyl)carbamoyl)benzoate (Figure 4, **4g**). From (*E*)-2-(hex-1-en-1-yl)-4,4,5,5-tetramethyl-1,3,2-dioxaborolane (**1a**) (42.0 mg, 0.20 mmol, 1.0 equiv) and methyl 4-(5-oxo-1,4,2-dioxazol-3-yl)benzoate (**2g**) (66.4 mg, 0.30 mmol, 1.5 equiv), the title compound was prepared following the general procedure **A** using $NiCl_2·6H_2O$ (4.8 mg, 10 mol%), **L*** (8.0 mg, 12 mol%), LiI (13.4 mg, 0.10 mmol, 0.50 equiv), MeOH (4.0 μL, 0.10 mmol, 0.50 equiv, instead of H_2O), $(EtO)_3SiH$ (92 μL, 0.50 mmol, 2.5 equiv), anhydrous DMA (1.0 mL). The reaction mixture was stirred for 20 h at 25 °C. The crude material was purified by flash column chromatography (petroleum ether/EtOAc = 2:1) to provide the title compound as a white solid in 62% yield (48.4 mg).

^{1}H NMR (500 MHz, $CDCl_3$) δ 8.18 (s, 1H), 7.98 (d, *J* = 8.4 Hz, 2H), 7.85 (d, *J* = 8.5 Hz, 2H), 3.93 (s, 3H), 3.00 – 2.91 (m, 1H), 1.77 – 1.68 (m, 1H), 1.65 – 1.53 (m, 1H), 1.48 – 1.37 (m, 2H), 1.35 – 1.24 (m, 16H), 0.88 (t, *J* = 7.0 Hz, 3H);

^{13}C NMR (126 MHz, $CDCl_3$) δ 169.2, 166.1, 133.8, 133.3, 129.8, 128.1, 82.1, 52.6, 32.1, 31.2, 27.4, 25.3, 25.2, 22.7, 14.2;

^{11}B NMR (160 MHz, $CDCl_3$) δ 21.8;

HRMS (ESI) calcd. for $C_{21}H_{33}BNO_5$ $[M+H]^+$ m/z 390.2446, found 390.2436;

IR (neat, cm^{-1}) 2925, 2856, 1730, 1603, 1278, 1107, 725;

m.p. 116 – 118 °C;

$[\alpha]_D^{25}$ = –27.8 (c = 1.01, $CHCl_3$);

HPLC analysis: the *ee* (93%) was determined using two connected CHIRALCEL® OD-H columns, 3% *i*PrOH in hexane, 0.8 mL/min, 254 nm UV detector, t_R (major) = 15.7 min, t_R (minor) = 18.7 min.

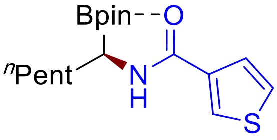

(*R*)-*N*-(1-(4,4,5,5-Tetramethyl-1,3,2-dioxaborolan-2-yl)hexyl)thiophene-3-carboxamide (Figure 4, **4h**). From (*E*)-2-(hex-1-en-1-yl)-4,4,5,5-tetramethyl-1,3,2-dioxaborolane (**1a**) (42.0 mg, 0.20 mmol, 1.0 equiv) and 3-(thiophen-3-yl)-1,4,2-dioxazol-5-one (**2h**) (50.7 mg, 0.30 mmol, 1.5 equiv), the title compound was prepared following the general procedure **A** using $NiCl_2·6H_2O$ (4.8 mg, 10 mol%), **L*** (8.0 mg, 12 mol%), LiI (13.4 mg, 0.10 mmol, 0.50 equiv), H_2O (1.8 μL, 0.10 mmol, 0.50 equiv), $(EtO)_3SiH$ (92 μL, 0.50 mmol, 2.5 equiv), anhydrous DMA (1.0 mL). The reaction mixture was stirred for 20 h at 25 °C. The crude material was purified by flash column chromatography (petroleum ether/EtOAc = 3:2) to provide the title compound as a colorless oil in 74% yield (49.7 mg).

^{1}H NMR (500 MHz, $CDCl_3$) δ 8.65 (s, 1H), 8.09 (dd, *J* = 2.9, 1.0 Hz, 1H), 7.44 (dd, *J* = 5.1, 1.1 Hz, 1H), 7.21 (dd, *J* = 5.1, 3.0 Hz, 1H), 2.76 (t, *J* = 5.9 Hz, 1H), 1.74 – 1.63 (m, 1H), 1.60 – 1.48 (m, 1H), 1.48 – 1.36 (m, 2H), 1.35 – 1.27 (m, 4H), 1.26 (s, 12H), 0.88 (t, *J* = 7.0 Hz, 3H);

^{13}C NMR (126 MHz, $CDCl_3$) δ 166.4, 132.3, 130.5, 126.6, 126.6, 81.3, 32.1, 31.3, 27.5, 25.3, 25.2, 22.7, 14.2;

^{11}B NMR (160 MHz, $CDCl_3$) δ 17.3;

HRMS (ESI) calcd. for $C_{17}H_{28}BNNaO_3S$ $[M+Na]^+$ m/z 360.1775, found 360.1765;

IR (neat, cm^{-1}) 3119, 2965, 2925, 1594, 1098;

$[\alpha]_D^{25}$ = –51.0 (c = 1.01, $CHCl_3$);

HPLC analysis: the *ee* (98%) was determined using a CHIRALCEL® OD-H column, 2% *i*PrOH in hexane, 1.0 mL/min, 254 nm UV detector, t_R (minor) = 6.1 min, t_R (major) = 8.4 min.

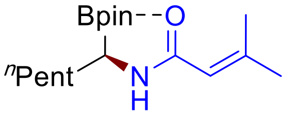

(*R*)-3-Methyl-*N*-(1-(4,4,5,5-tetramethyl-1,3,2-dioxaborolan-2-yl)hexyl)but-2-enamide (Figure 4, **4i**). From (*E*)-2-(hex-1-en-1-yl)-4,4,5,5-tetramethyl-1,3,2-dioxaborolane (**1a**) (42.0 mg, 0.20 mmol, 1.0 equiv) and 3-(2-methylprop-1-en-1-yl)-

1,4,2-dioxazol-5-one (**2i**) (42.3 mg, 0.30 mmol, 1.5 equiv), the title compound was prepared following the general procedure **A** using $NiCl_2 \cdot 6H_2O$ (4.8 mg, 10 mol%), **L*** (8.0 mg, 12 mol%), LiI (13.4 mg, 0.10 mmol, 0.50 equiv), H_2O (1.8 μL, 0.10 mmol, 0.50 equiv), $(EtO)_3SiH$ (92 μL, 0.50 mmol, 2.5 equiv), anhydrous DMA (1.0 mL). The reaction mixture was stirred for 20 h at 25 °C. The crude material was purified by flash column chromatography (petroleum ether/EtOAc = 3:2) to provide the title compound as a colorless oil in 63% yield (39.2 mg).

^{1}H NMR (500 MHz, $CDCl_3$) δ 8.65 (s, 1H), 5.74 (s, 1H), 2.40 – 2.33 (m, 1H), 2.18 (s, 3H), 1.87 (s, 3H), 1.60 – 1.51 (m, 1H), 1.47 – 1.39 (m, 1H), 1.36 – 1.24 (m, 6H), 1.21 (s, 6H), 1.19 (s, 6H), 0.87 (t, J = 6.9 Hz, 3H);

^{13}C NMR (126 MHz, $CDCl_3$) δ 170.8, 158.2, 112.1, 80.2, 32.2, 31.5, 28.3, 28.0, 25.5, 25.1, 22.8, 21.0, 14.3;

^{11}B NMR (160 MHz, $CDCl_3$) δ 14.1;

HRMS (ESI) calcd. for $C_{17}H_{33}BNO_3$ $[M+H]^+$ m/z 310.2548, found 310.2543;

IR (neat, cm^{-1}) 3176, 2965, 2927, 1665, 1573, 1156, 1107;

$[\alpha]_D^{25}$ = –79.0 (c = 1.00, $CHCl_3$);

HPLC analysis: the *ee* (97%) was determined using a CHIRALPAK® AD-H column, 5% *i*PrOH in hexane, 1.0 mL/min, 240 nm UV detector, t_R (major) = 5.2 min, t_R (minor) = 7.2 min.

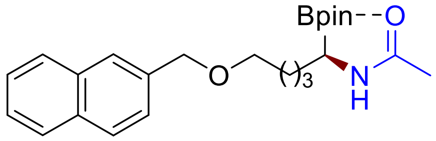

(*R*)-*N*-(5-(Naphthalen-2-ylmethoxy)-1-(4,4,5,5-tetramethyl-1,3,2-dioxaborolan-2-yl)pentyl)acetamide (Figure 4, **4j**). From (*E*)-4,4,5,5-tetramethyl-2-(5-(naphthalen-2-ylmethoxy)pent-1-en-1-yl)-1,3,2-dioxaborolane (**1t**) (70.5 mg, 0.20 mmol, 1.0 equiv) and 3-methyl-1,4,2-dioxazol-5-one (**2j**) (30.3 mg, 0.30 mmol, 1.5 equiv), the title compound was prepared following the general procedure **A** using $NiCl_2 \cdot 6H_2O$ (4.8 mg, 10 mol%), **L*** (8.0 mg, 12 mol%), LiI (13.4 mg, 0.10 mmol, 0.50 equiv), H_2O (1.8 μL, 0.10 mmol, 0.50 equiv), $(EtO)_3SiH$ (92 μL, 0.50 mmol, 2.5 equiv), anhydrous DMA

(1.0 mL). The reaction mixture was stirred for 20 h at 25 °C. The crude material was purified by flash column chromatography (CH_2Cl_2/MeOH = 10:1) to provide the title compound as a colorless oil in 47% yield (38.3 mg).

^{1}H NMR (500 MHz, $CDCl_3$) δ 8.01 (s, 1H), 7.84 – 7.78 (m, 3H), 7.75 (s, 1H), 7.49 – 7.41 (m, 3H), 4.63 (s, 2H), 3.56 – 3.47 (m, 2H), 2.55 – 2.46 (m, 1H), 1.94 (s, 3H), 1.74 – 1.56 (m 3H), 1.56 – 1.37 (m, 3H), 1.17 (s, 12H);

^{13}C NMR (126 MHz, $CDCl_3$) δ 175.0, 136.1, 133.4, 133.1, 128.3, 128.0, 127.8, 126.6, 126.3, 126.0, 126.0, 80.7, 73.2, 70.6, 30.8, 29.6, 25.4, 25.2, 24.8, 18.1;

^{11}B NMR (160 MHz, $CDCl_3$) δ 16.6;

HRMS (ESI) calcd. for $C_{24}H_{34}BNNaO_4$ $[M+Na]^+$ m/z 434.2473, found 434.2461;

IR (neat, cm^{-1}) 3053, 2969, 2927, 1609, 1557, 1154, 1097;

$[\alpha]_D^{25}$ = –59.4 (c = 1.04, $CHCl_3$);

HPLC analysis: the *ee* (97%) was determined using a CHIRALCEL® OD-H column, 5% *i*PrOH in hexane, 1.0 mL/min, 254 nm UV detector, t_R (minor) = 10.0 min, t_R (major) = 12.1 min.

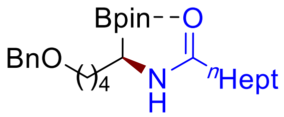

(*R*)-*N*-(5-(Benzyloxy)-1-(4,4,5,5-tetramethyl-1,3,2-dioxaborolan-2-yl)pentyl)octanamide (Figure 4, **4k**). From (*E*)-2-(5-(benzyloxy)pent-1-en-1-yl)-4,4,5,5-tetramethyl-1,3,2-dioxaborolane (**1i**) (60.4 mg, 0.20 mmol, 1.0 equiv) and 3-heptyl-1,4,2-dioxazol-5-one (**2k**) (55.6 mg, 0.30 mmol, 1.5 equiv), the title compound was prepared following the general procedure **A** using $NiCl_2·6H_2O$ (4.8 mg, 10 mol%), **L*** (8.0 mg, 12 mol%), LiI (13.4 mg, 0.10 mmol, 0.50 equiv), H_2O (1.8 µL, 0.10 mmol, 0.50 equiv), $(EtO)_3SiH$ (92 µL, 0.50 mmol, 2.5 equiv), anhydrous DMA (1.0 mL). The reaction mixture was stirred for 20 h at 25 °C. The crude material was purified by flash column chromatography (petroleum ether/EtOAc = 1:1) to provide the title compound as a colorless oil in 63% yield (56.4 mg).

^{1}H NMR (500 MHz, $CDCl_3$) δ 7.38 (s, 1H), 7.35 – 7.30 (m, 4H), 7.30 – 7.26 (m, 1H),

4.48 (s, 2H), 3.53 – 3.43 (m, 2H), 2.60 – 2.52 (m, 1H), 2.27 – 2.14 (m, 2H), 1.71 – 1.52 (m, 5H), 1.52 – 1.38 (m, 3H), 1.32 – 1.22 (m, 8H), 1.20 (s, 12H), 0.87 (t, *J* = 7.0 Hz, 3H);

^{13}C NMR (126 MHz, $CDCl_3$) δ 178.1, 138.6, 128.5, 127.8, 127.7, 80.8, 73.1, 70.6, 31.9, 31.7, 30.8, 29.6, 29.2, 28.9, 25.3, 25.1, 25.1, 24.8, 22.7, 14.2;

^{11}B NMR (160 MHz, $CDCl_3$) δ 16.6;

HRMS (ESI) calcd. for $C_{26}H_{44}BNNaO_4$ $[M+Na]^+$ m/z 468.3256, found 468.3246;

IR (neat, cm^{-1}) 2963, 2925, 1603, 1155, 1099;

$[\alpha]_D^{25}$ = –50.0 (c = 1.08, $CHCl_3$);

HPLC analysis: the *ee* (97%) was determined using a CHIRALCEL® OD-H column, 8% *i*PrOH in hexane, 0.5 mL/min, 210 nm UV detector, t_R (major) = 9.6 min, t_R (minor) = 10.6 min.

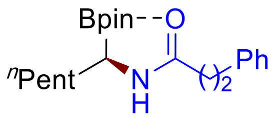

(*R*)-3-Phenyl-*N*-(1-(4,4,5,5-tetramethyl-1,3,2-dioxaborolan-2-yl)hexyl)propanamide (Figure 4, **4l**). From (*E*)-2-(hex-1-en-1-yl)-4,4,5,5-tetramethyl-1,3,2-dioxaborolane (**1a**) (42.0 mg, 0.20 mmol, 1.0 equiv) and 3-phenethyl-1,4,2-dioxazol-5-one (**2l**) (57.4 mg, 0.30 mmol, 1.5 equiv), the title compound was prepared following the general procedure **A** using $NiCl_2 \cdot 6H_2O$ (4.8 mg, 10 mol%), **L*** (8.0 mg, 12 mol%), LiI (13.4 mg, 0.10 mmol, 0.50 equiv), H_2O (1.8 μL, 0.10 mmol, 0.50 equiv), $(EtO)_3SiH$ (92 μL, 0.50 mmol, 2.5 equiv), anhydrous DMA (1.0 mL). The reaction mixture was stirred for 20 h at 25 °C. The crude material was purified by flash column chromatography (petroleum ether/EtOAc = 2:3) to provide the title compound as a colorless oil in 61% yield (43.9 mg).

^{1}H NMR (500 MHz, $CDCl_3$) δ 7.43 (s, 1H), 7.30 – 7.24 (m, 2H), 7.20 (t, *J* = 7.3 Hz, 1H), 7.15 (d, *J* = 7.4 Hz, 2H), 2.91 (t, *J* = 7.8 Hz, 2H), 2.64 – 2.51 (m, 3H), 1.61 – 1.51 (m, 1H), 1.39 – 1.32 (m, 1H), 1.31 – 1.22 (m, 6H), 1.21 (s, 12H), 0.87 (t, *J* = 6.8 Hz, 3H);

^{13}C NMR (126 MHz, $CDCl_3$) δ 176.3, 139.8, 128.8, 128.4, 126.7, 81.1, 34.1, 32.0, 31.2, 31.2, 27.8, 25.4, 25.2, 22.7, 14.2;

^{11}B NMR (160 MHz, $CDCl_3$) δ 19.2;

HRMS (ESI) calcd. for $C_{21}H_{35}BNO_3$ $[M+H]^+$ m/z 360.2705, found 360.2696;

IR (neat, cm^{-1}) 3168, 2961, 2926, 1604, 1550, 1155, 1109;

$[\alpha]_D^{25}$ = –43.0 (c = 0.91, $CHCl_3$);

HPLC analysis: the *ee* (97%) was determined using a CHIRALCEL® OD-H column, 5% *i*PrOH in hexane, 1.0 mL/min, 210 nm UV detector, t_R (minor) = 5.7 min, t_R (major) = 6.5 min.

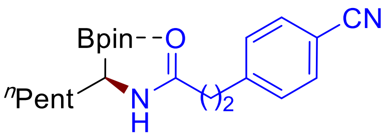

(*R*)-3-(4-Cyanophenyl)-*N*-(1-(4,4,5,5-tetramethyl-1,3,2-dioxaborolan-2-yl)hexyl)propanamide (Figure 4, **4m**). From (*E*)-2-(hex-1-en-1-yl)-4,4,5,5-tetramethyl-1,3,2-dioxaborolane (**1a**) (42.0 mg, 0.20 mmol, 1.0 equiv) and 4-(2-(5-oxo-1,4,2-dioxazol-3-yl)ethyl)benzonitrile (**2m**) (64.9 mg, 0.30 mmol, 1.5 equiv), the title compound was prepared following the general procedure **A** using $NiCl_2{\cdot}6H_2O$ (4.8 mg, 10 mol%), **L*** (8.0 mg, 12 mol%), LiI (13.4 mg, 0.10 mmol, 0.50 equiv), H_2O (1.8 μL, 0.10 mmol, 0.50 equiv), $(EtO)_3SiH$ (92 μL, 0.50 mmol, 2.5 equiv), anhydrous DMA (1.0 mL). The reaction mixture was stirred for 20 h at 25 °C. The crude material was purified by flash column chromatography (petroleum ether/EtOAc = 2:3) to provide the title compound as a colorless oil in 48% yield (36.8 mg).

^{1}H NMR (500 MHz, $CDCl_3$) δ 7.57 (d, *J* = 8.2 Hz, 2H), 7.31 (d, *J* = 8.2 Hz, 2H), 6.41 (s, 1H), 3.02 (t, *J* = 7.5 Hz, 2H), 2.83 – 2.74 (m, 1H), 2.62 – 2.48 (m, 2H), 1.61 – 1.51 (m, 1H), 1.44 – 1.35 (m, 1H), 1.31 – 1.24 (m, 6H), 1.23 (s, 12H), 0.87 (t, *J* = 6.8 Hz, 3H);

^{13}C NMR (126 MHz, $CDCl_3$) δ 174.0, 145.9, 132.5, 129.4, 119.0, 110.6, 82.2, 34.9, 31.9, 31.4, 31.1, 27.4, 25.2, 25.1, 22.7, 14.2;

^{11}B NMR (160 MHz, $CDCl_3$) δ 23.7;

HRMS (ESI) calcd. for $C_{22}H_{34}BN_2O_3$ $[M+H]^+$ m/z 385.2657, found 385.2648;

IR (neat, cm^{-1}) 3173, 2964, 2927, 2229, 1606, 1155, 1109;

$[\alpha]_D^{25}$ = –19.1 (c = 0.58, $CHCl_3$);

HPLC analysis: the *ee* (95%) was determined using a CHIRALPAK® AD-H column, 10% *i*PrOH in hexane, 1.0 mL/min, 220 nm UV detector, t_R (major) = 4.1 min, t_R (minor) = 5.0 min.

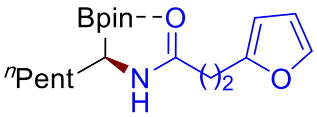

(*R*)-3-(Furan-2-yl)-*N*-(1-(4,4,5,5-tetramethyl-1,3,2-dioxaborolan-2-yl)hexyl)propanamide (Figure 4, **4n**). From (*E*)-2-(hex-1-en-1-yl)-4,4,5,5-tetramethyl-1,3,2-dioxaborolane (**1a**) (42.0 mg, 0.20 mmol, 1.0 equiv) and 3-(2-(furan-2-yl)ethyl)-1,4,2-dioxazol-5-one (**2n**) (54.3 mg, 0.30 mmol, 1.5 equiv), the title compound was prepared following the general procedure **A** using $NiCl_2 \cdot 6H_2O$ (4.8 mg, 10 mol%), **L*** (8.0 mg, 12 mol%), LiI (13.4 mg, 0.10 mmol, 0.50 equiv), H_2O (1.8 μL, 0.10 mmol, 0.50 equiv), $(EtO)_3SiH$ (92 μL, 0.50 mmol, 2.5 equiv), anhydrous DMA (1.0 mL). The reaction mixture was stirred for 20 h at 25 °C. The crude material was purified by flash column chromatography (petroleum ether/EtOAc = 2:1) to provide the title compound as a yellow oil in 58% yield (40.6 mg).

^{1}H NMR (500 MHz, $CDCl_3$) δ 7.34 – 7.26 (m, 2H), 6.26 (dd, *J* = 3.1, 1.9 Hz, 1H), 6.04 (d, *J* = 2.7 Hz, 1H), 2.95 (t, *J* = 7.6 Hz, 2H), 2.69 – 2.55 (m, 3H), 1.62 – 1.52 (m, 1H), 1.43 – 1.35 (m, 1H), 1.33 – 1.23 (m, 6H), 1.21 (s, 12H), 0.87 (t, *J* = 6.8 Hz, 3H);

^{13}C NMR (126 MHz, $CDCl_3$) δ 175.6, 153.3, 141.6, 110.5, 106.2, 81.3, 32.0, 31.3, 31.2, 27.7, 25.3, 25.1, 23.7, 22.7, 14.2;

^{11}B NMR (160 MHz, $CDCl_3$) δ 19.9;

HRMS (ESI) calcd. for $C_{19}H_{33}BNO_4$ $[M+H]^+$ m/z 350.2497, found 350.2491;

IR (neat, cm^{-1}) 3168, 2964, 2926, 1605, 1552, 1154, 1109, 729;

$[\alpha]_D^{25}$ = –37.7 (c = 1.04, $CHCl_3$);

HPLC analysis: the *ee* (96%) was determined using a CHIRALPAK® AD-H column,

5% *i*PrOH in hexane, 0.8 mL/min, 220 nm UV detector, t_R (major) = 5.6 min, t_R (minor) = 7.5 min.

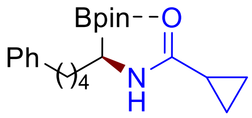

(*R*)-*N*-(5-Phenyl-1-(4,4,5,5-tetramethyl-1,3,2-dioxaborolan-2-yl)pentyl)cyclopropanecarboxamide (Figure 4, **4o**). From (*E*)-4,4,5,5-tetramethyl-2-(5-phenylpent-1-en-1-yl)-1,3,2-dioxaborolane (**1c**) (54.4 mg, 0.20 mmol, 1.0 equiv) and 3-cyclopropyl-1,4,2-dioxazol-5-one (**2o**) (38.1 mg, 0.30 mmol, 1.5 equiv), the title compound was prepared following the general procedure **A** using $NiCl_2 \cdot 6H_2O$ (4.8 mg, 10 mol%), **L*** (8.0 mg, 12 mol%), LiI (13.4 mg, 0.10 mmol, 0.50 equiv), H_2O (1.8 μL, 0.10 mmol, 0.50 equiv), $(EtO)_3SiH$ (92 μL, 0.50 mmol, 2.5 equiv), anhydrous DMA (1.0 mL). The reaction mixture was stirred for 20 h at 25 °C. The crude material was purified by flash column chromatography (petroleum ether/EtOAc = 1:1) to provide the title compound as a colorless oil in 55% yield (39.3 mg).

^{1}H NMR (500 MHz, $CDCl_3$) δ 8.32 (s, 1H), 7.28 – 7.22 (m, 2H), 7.19 – 7.12 (m, 3H), 2.67 – 2.53 (m, 2H), 2.52 – 2.45 (m, 1H), 1.70 – 1.52 (m, 4H), 1.52 – 1.32 (m, 3H), 1.31 – 1.21 (m, 1H), 1.16 (s, 6H), 1.15 (s, 6H), 1.12 – 1.06 (m, 1H), 0.95 – 0.85 (m, 2H);

^{13}C NMR (126 MHz, $CDCl_3$) δ 178.6, 142.8, 128.5, 128.4, 125.8, 80.5, 36.0, 31.7, 31.3, 27.9, 25.5, 25.1, 10.9, 8.6;

^{11}B NMR (160 MHz, $CDCl_3$) δ 16.2;

HRMS (ESI) calcd. for $C_{21}H_{33}BNO_3$ $[M+H]^+$ m/z 358.2548, found 358.2538;

IR (neat, cm^{-1}) 3206, 3062, 2929, 1603, 1548, 1154, 1115, 734;

$[\alpha]_D^{25}$ = –61.3 (c = 0.97, $CHCl_3$);

HPLC analysis: the *ee* (98%) was determined using a CHIRALPAK® AD-H column, 5% *i*PrOH in hexane, 1.0 mL/min, 220 nm UV detector, t_R (major) = 4.3 min, t_R (minor) = 6.4 min.

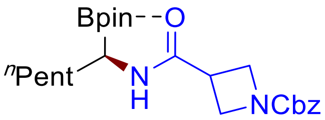

Benzyl (*R*)-3-((1-(4,4,5,5-tetramethyl-1,3,2-dioxaborolan-2-yl)hexyl)carbamoyl)azetidine-1-carboxylate (Figure 4, **4p**). From (*E*)-2-(hex-1-en-1-yl)-4,4,5,5-tetramethyl-1,3,2-dioxaborolane (**1a**) (42.0 mg, 0.20 mmol, 1.0 equiv) and benzyl 3-(5-oxo-1,4,2-dioxazol-3-yl)azetidine-1-carboxylate (**2p**) (82.9 mg, 0.30 mmol, 1.5 equiv), the title compound was prepared following the general procedure **A** using $NiCl_2·6H_2O$ (4.8 mg, 10 mol%), **L*** (8.0 mg, 12 mol%), LiI (13.4 mg, 0.10 mmol, 0.50 equiv), H_2O (1.8 μL, 0.10 mmol, 0.50 equiv), $(EtO)_3SiH$ (92 μL, 0.50 mmol, 2.5 equiv), anhydrous DMA (1.0 mL). The reaction mixture was stirred for 20 h at 25 °C. The crude material was purified by flash column chromatography (CH_2Cl_2/MeOH = 30:1) to provide the title compound as a colorless oil in 47% yield (42.0 mg).

^{1}H NMR (500 MHz, $CDCl_3$) δ 7.36 – 7.29 (m, 5H), 6.14 (s, 1H), 5.08 (s, 2H), 4.24 – 4.14 (m, 2H), 4.11 (t, *J* = 8.5 Hz, 2H), 3.29 – 3.20 (m, 1H), 3.06 – 2.96 (m, 1H), 1.67 – 1.56 (m, 1H), 1.54 – 1.42 (m, 1H), 1.33 – 1.26 (m, 6H), 1.24 (s, 6H), 1.24 (s, 6H), 0.87 (t, *J* = 6.6 Hz, 3H);

^{13}C NMR (126 MHz, $CDCl_3$) δ 172.9, 156.4, 136.6, 128.6, 128.2, 128.1, 83.2, 66.9, 52.0, 32.5, 31.9, 31.0, 27.1, 25.1, 25.1, 22.6, 14.1;

^{11}B NMR (160 MHz, $CDCl_3$) δ 27.9;

HRMS (ESI) calcd. for $C_{24}H_{37}BN_2NaO_5$ $[M+Na]^+$ m/z 467.2688, found 467.2678;

IR (neat, cm^{-1}) 3210, 2959, 2829, 1711, 1605, 1352, 1132;

$[\alpha]_D^{25}$ = –13.4 (c = 0.95, $CHCl_3$);

HPLC analysis: the *ee* (96%) was determined using a CHIRALCEL® OD-H column, 5% *i*PrOH in hexane, 1.0 mL/min, 210 nm UV detector, t_R (minor) = 9.9 min, t_R (major) = 11.7 min.

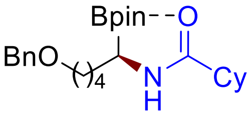

(*R*)-*N*-(5-(Benzyloxy)-1-(4,4,5,5-tetramethyl-1,3,2-dioxaborolan-2-

yl)pentyl)cyclohexanecarboxamide (Figure 4, **4q**). From (*E*)-2-(5-(benzyloxy)pent-1-en-1-yl)-4,4,5,5-tetramethyl-1,3,2-dioxaborolane (**1i**) (60.4 mg, 0.20 mmol, 1.0 equiv) and 3-cyclohexyl-1,4,2-dioxazol-5-one (**2q**) (50.8 mg, 0.30 mmol, 1.5 equiv), the title compound was prepared following the general procedure **A** using $NiCl_2 \cdot 6H_2O$ (4.8 mg, 10 mol%), **L*** (8.0 mg, 12 mol%), LiI (13.4 mg, 0.10 mmol, 0.50 equiv), H_2O (1.8 μL, 0.10 mmol, 0.50 equiv), $(EtO)_3SiH$ (92 μL, 0.50 mmol, 2.5 equiv), anhydrous DMA (1.0 mL). The reaction mixture was stirred for 20 h at 25 °C. The crude material was purified by flash column chromatography (petroleum ether/EtOAc = 1:1) to provide the title compound as a colorless oil in 65% yield (55.8 mg).

^{1}H NMR (500 MHz, $CDCl_3$) δ 7.35 – 7.30 (m, 4H), 7.30 – 7.26 (m, 1H), 7.10 (s, 1H), 4.48 (s, 2H), 3.49 (t, *J* = 6.3 Hz, 2H), 2.59 – 2.50 (m, 1H), 2.25 – 2.15 (m, 1H), 1.89 – 1.81 (m, 2H), 1.81 – 1.71 (m, 2H), 1.70 – 1.56 (m, 4H), 1.50 –1.39 (m, 3H), 1.39 – 1.29 (m, 2H), 1.29 – 1.21 (m, 3H), 1.19 (s, 12H);

^{13}C NMR (126 MHz, $CDCl_3$) δ 180.9, 138.6, 128.5, 127.8, 127.7, 80.6, 73.1, 70.6, 40.5, 30.8, 29.6, 28.9, 28.8, 25.6, 25.4, 25.4, 25.4, 25.1, 24.8;

^{11}B NMR (160 MHz, $CDCl_3$) δ 16.0;

HRMS (ESI) calcd. for $C_{25}H_{40}BNNaO_4$ $[M+Na]^+$ m/z 452.2943, found 452.2933;

IR (neat, cm^{-1}) 3198, 2930, 2855, 1599, 1098, 733;

$[\alpha]_D^{25}$ = –55.7 (c = 1.05, $CHCl_3$);

HPLC analysis: the *ee* (94%) was determined using a CHIRALPAK® AD-H column, 5% *i*PrOH in hexane, 1.0 mL/min, 220 nm UV detector, t_R (major) = 4.6 min, t_R (minor) = 8.4 min.

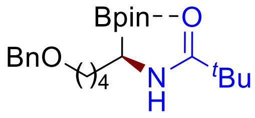

(*R*)-*N*-(5-(Benzyloxy)-1-(4,4,5,5-tetramethyl-1,3,2-dioxaborolan-2-yl)pentyl)pivalamide (Figure 4, **4r**). From (*E*)-2-(5-(benzyloxy)pent-1-en-1-yl)-4,4,5,5-tetramethyl-1,3,2-dioxaborolane (**1i**) (60.4 mg, 0.20 mmol, 1.0 equiv) and 3-(*tert*-butyl)-1,4,2-dioxazol-5-one (**2r**) (42.9 mg, 0.30 mmol, 1.5 equiv), the title

compound was prepared following the general procedure **A** using $NiCl_2 \cdot 6H_2O$ (4.8 mg, 10 mol%), **L*** (8.0 mg, 12 mol%), LiI (13.4 mg, 0.10 mmol, 0.50 equiv), H_2O (1.8 μL, 0.10 mmol, 0.50 equiv), $(EtO)_3SiH$ (92 μL, 0.50 mmol, 2.5 equiv), anhydrous DMA (1.0 mL). The reaction mixture was stirred for 20 h at 25 °C. The crude material was purified by flash column chromatography (petroleum ether/EtOAc = 1:1) to provide the title compound as a colorless oil in 53% yield (42.4 mg).

^{1}H NMR (500 MHz, $CDCl_3$) δ 7.35 – 7.30 (m, 4H), 7.29 – 7.25 (m, 1H), 6.85 (s, 1H), 4.49 (s, 2H), 3.49 (t, J = 6.3 Hz, 2H), 2.59 – 2.51 (m, 1H), 1.72 – 1.56 (m, 3H), 1.51 – 1.38 (m, 3H), 1.21 – 1.16 (m, 21H);

^{13}C NMR (126 MHz, $CDCl_3$) δ 183.9, 138.6, 128.5, 127.8, 127.7, 80.4, 73.1, 70.5, 35.7, 30.9, 29.6, 26.9, 25.4, 25.2, 24.8;

^{11}B NMR (160 MHz, $CDCl_3$) δ 15.8;

HRMS (ESI) calcd. for $C_{23}H_{39}BNO_4$ $[M+H]^+$ m/z 404.2967, found 404.2956;

IR (neat, cm^{-1}) 3204, 2970, 2931, 1581, 1097, 732;

$[\alpha]_D^{25}$ = –49.9 (c = 0.99, $CHCl_3$);

HPLC analysis: the *ee* (83%) was determined using a CHIRALCEL® OD-H column, 5% *i*PrOH in hexane, 1.0 mL/min, 220 nm UV detector, t_R (major) = 5.3 min, t_R (minor) = 8.2 min.

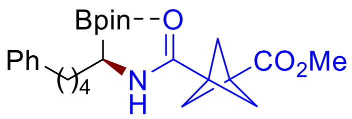

Methyl (*R*)-3-((5-phenyl-1-(4,4,5,5-tetramethyl-1,3,2-dioxaborolan-2-yl)pentyl)carbamoyl)bicyclo[1.1.1]pentane-1-carboxylate (Figure 4, **4s**). From (*E*)-4,4,5,5-tetramethyl-2-(5-phenylpent-1-en-1-yl)-1,3,2-dioxaborolane (**1c**) (54.4 mg, 0.20 mmol, 1.0 equiv) and methyl 3-(5-oxo-1,4,2-dioxazol-3-yl)bicyclo[1.1.1]pentane-1-carboxylate (**2s**) (63.4 mg, 0.30 mmol, 1.5 equiv), the title compound was prepared following the general procedure **A** using $NiCl_2 \cdot 6H_2O$ (4.8 mg, 10 mol%), **L*** (8.0 mg, 12 mol%), LiI (13.4 mg, 0.10 mmol, 0.50 equiv), H_2O (1.8 μL, 0.10 mmol, 0.50 equiv), $(EtO)_3SiH$ (92 μL, 0.50 mmol, 2.5 equiv), anhydrous DMA (1.0 mL). The reaction

mixture was stirred for 20 h at 25 °C. The crude material was purified by flash column chromatography (petroleum ether/EtOAc = 1:1) to provide the title compound as a colorless oil in 53% yield (46.6 mg).

^{1}H NMR (500 MHz, $CDCl_3$) δ 7.28 – 7.24 (m, 2H), 7.19 – 7.14 (m, 3H), 6.15 (s, 1H), 3.69 (s, 3H), 2.89 – 2.80 (m, 1H), 2.65 – 2.56 (m, 2H), 2.28 (s, 6H), 1.70 – 1.59 (m, 3H), 1.56 – 1.43 (m, 1H), 1.37 – 1.29 (m, 2H), 1.21 (s, 12H);

^{13}C NMR (126 MHz, $CDCl_3$) δ171.5, 169.6, 142.6, 128.6, 128.4, 125.8, 82.4, 52.6, 52.0, 37.6, 37.5, 35.8, 31.3, 31.0, 27.0, 25.2, 25.1;

^{11}B NMR (160 MHz, $CDCl_3$) δ 24.5;

HRMS (ESI) calcd. for $C_{25}H_{36}BNNaO_5$ $[M+Na]^+$ m/z 464.2579, found 464.2567;

IR (neat, cm^{-1}) 2929, 1732, 1598, 1202, 1141;

$[\alpha]_D^{25}$ = –29.1 (c = 0.99, $CHCl_3$);

HPLC analysis: the *ee* (97%) was determined using a CHIRALPAK® AD-H column, 5% *i*PrOH in hexane, 1.0 mL/min, 220 nm UV detector, t_R (major) = 5.2 min, t_R (minor) = 6.0 min.

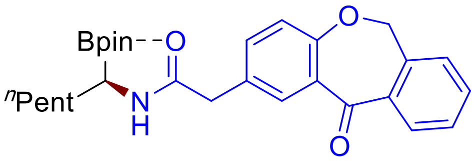

(*R*)-2-(11-Oxo-6,11-dihydrodibenzo[*b*,*e*]oxepin-2-yl)-*N*-(1-(4,4,5,5-tetramethyl-1,3,2-dioxaborolan-2-yl)hexyl)acetamide (Figure 4, **4t**). From (*E*)-2-(hex-1-en-1-yl)-4,4,5,5-tetramethyl-1,3,2-dioxaborolane (**1a**) (42.0 mg, 0.20 mmol, 1.0 equiv) and 3-((11-oxo-6,11-dihydrodibenzo[*b*,*e*]oxepin-2-yl)methyl)-1,4,2-dioxazol-5-one (**2t**) (92.8 mg, 0.30 mmol, 1.5 equiv), the title compound was prepared following the general procedure **A** using $NiCl_2·6H_2O$ (4.8 mg, 10 mol%), **L*** (8.0 mg, 12 mol%), LiI (13.4 mg, 0.10 mmol, 0.50 equiv), H_2O (1.8 μL, 0.10 mmol, 0.50 equiv), $(EtO)_3SiH$ (92 μL, 0.50 mmol, 2.5 equiv), anhydrous DMA (1.0 mL). The reaction mixture was stirred for 20 h at 25 °C. The crude material was purified by flash column chromatography (petroleum ether/EtOAc = 1:1) to provide the title compound as a colorless oil in 50% yield (47.6 mg).

^{1}H NMR (500 MHz, $CDCl_3$) δ 8.05 (d, *J* = 2.2 Hz, 1H), 7.92 – 7.82 (m, 1H), 7.59 – 7.53 (m, 1H), 7.51 – 7.44 (m, 1H), 7.40 (dd, *J* = 8.4, 2.3 Hz, 1H), 7.38 – 7.34 (m, 1H), 7.03 (d, *J* = 8.4 Hz, 1H), 6.47 (s, 1H), 5.18 (s, 2H), 3.64 (s, 2H), 2.76 – 2.68 (m, 1H), 1.61 – 1.51 (m, 1H), 1.44 – 1.34 (m, 1H), 1.29 – 1.17 (m, 18H), 0.82 (t, *J* = 6.7 Hz, 3H);

^{13}C NMR (126 MHz, $CDCl_3$) δ 190.7, 174.2, 160.9, 140.4, 136.4, 135.6, 133.1, 132.7, 129.6, 129.5, 128.0, 127.1, 125.5, 121.8, 81.8, 73.8, 39.3, 31.9, 31.1, 27.4, 25.3, 25.1, 22.6, 14.1;

^{11}B NMR (160 MHz, $CDCl_3$) δ 22.4;

HRMS (ESI) calcd. for $C_{28}H_{36}BNNaO_5$ $[M+Na]^+$ m/z 500.2579, found 500.2569;

IR (neat, cm^{-1}) 3055, 2927, 1660, 1613, 1265, 735;

$[\alpha]_D^{25}$ = –20.5 (c = 1.01, $CHCl_3$);

HPLC analysis: the *ee* (97%) was determined using a CHIRALPAK® AD-H column, 10% *i*PrOH in hexane, 1.0 mL/min, 240 nm UV detector, t_R (major) = 6.3 min, t_R (minor) = 7.7 min.

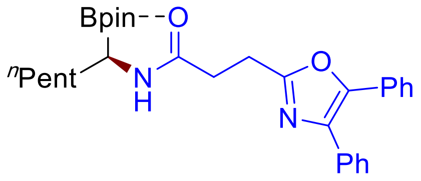

(*R*)-3-(4,5-Diphenyloxazol-2-yl)-*N*-(1-(4,4,5,5-tetramethyl-1,3,2-dioxaborolan-2-yl)hexyl)propanamide (Figure 4, **4u**). From (*E*)-2-(hex-1-en-1-yl)-4,4,5,5-tetramethyl-1,3,2-dioxaborolane (**1a**) (42.0 mg, 0.20 mmol, 1.0 equiv) and 3-(2-(4,5-diphenyloxazol-2-yl)ethyl)-1,4,2-dioxazol-5-one (**2u**) (100.3 mg, 0.30 mmol, 1.5 equiv), the title compound was prepared following the general procedure **A** using $NiCl_2·6H_2O$ (4.8 mg, 10 mol%), **L*** (8.0 mg, 12 mol%), LiI (13.4 mg, 0.10 mmol, 0.50 equiv), H_2O (1.8 μL, 0.10 mmol, 0.50 equiv), $(EtO)_3SiH$ (92 μL, 0.50 mmol, 2.5 equiv), anhydrous DMA (1.0 mL). The reaction mixture was stirred for 20 h at 25 °C. The crude material was purified by flash column chromatography (petroleum ether/EtOAc = 1:1) to provide the title compound as a colorless oil in 51% yield (51.0 mg).

^{1}H NMR (500 MHz, $CDCl_3$) δ 7.91 (s, 1H), 7.64 – 7.56 (m, 4H), 7.41 – 7.33 (m 6H),

3.28 – 3.18 (m, 2H), 2.97 – 2.88 (m, 2H), 2.81 – 2.73 (m, 1H), 1.63 – 1.54 (m, 1H), 1.50 – 1.39 (m, 1H), 1.34 – 1.25 (m, 4H), 1.25 – 1.20 (m, 14H), 0.84 (t, *J* = 6.7 Hz, 3H);

^{13}C NMR (126 MHz, $CDCl_3$) δ 175.1, 162.1, 146.0, 134.6, 131.7, 129.0, 128.9, 128.8, 128.5, 128.0, 126.9, 126.7, 81.5, 31.9, 31.0, 29.6, 27.8, 25.2, 25.1, 23.8, 22.6, 14.2;

^{11}B NMR (160 MHz, $CDCl_3$) δ 20.8;

HRMS (ESI) calcd. for $C_{30}H_{40}BN_2O_4$ $[M+H]^+$ m/z 503.3076, found 503.3068;

IR (neat, cm^{-1}) 3198, 3056, 2927, 1606, 1156, 1110, 763, 693;

$[\alpha]_D^{25}$ = –21.1 (c = 0.92, $CHCl_3$);

HPLC analysis: the *ee* (97%) was determined using a CHIRALPAK® AD-H column, 5% *i*PrOH in hexane, 0.8 mL/min, 240 nm UV detector, t_R (major) = 6.2 min, t_R (minor) = 7.5 min.

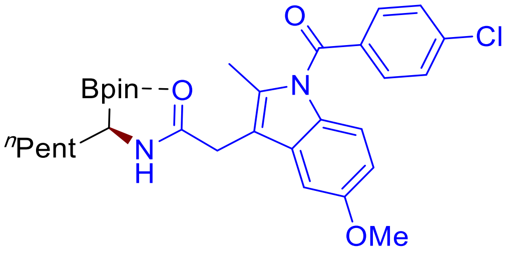

(*R*)-2-(1-(4-Chlorobenzoyl)-5-methoxy-2-methyl-1*H*-indol-3-yl)-*N*-(1-(4,4,5,5-tetramethyl-1,3,2-dioxaborolan-2-yl)hexyl)acetamide (Figure 4, **4v**). From (*E*)-2-(hex-1-en-1-yl)-4,4,5,5-tetramethyl-1,3,2-dioxaborolane (**1a**) (42.0 mg, 0.20 mmol, 1.0 equiv) and 3-((1-(4-chlorobenzoyl)-5-methoxy-2-methyl-1*H*-indol-3-yl)methyl)-1,4,2-dioxazol-5-one (**2v**) (119.6 mg, 0.30 mmol, 1.5 equiv), the title compound was prepared following the general procedure **A** using $NiCl_2 \cdot 6H_2O$ (4.8 mg, 10 mol%), **L*** (8.0 mg, 12 mol%), LiI (13.4 mg, 0.10 mmol, 0.50 equiv), H_2O (1.8 μL, 0.10 mmol, 0.50 equiv), $(EtO)_3SiH$ (92 μL, 0.50 mmol, 2.5 equiv), anhydrous DMA (1.0 mL). The reaction mixture was stirred for 20 h at 25 °C. The crude material was purified by flash column chromatography (petroleum ether/EtOAc = 2:3) to provide the title compound as a light yellow solid in 46% yield (52.6 mg).

^{1}H NMR (500 MHz, $CDCl_3$) δ 7.62 (d, *J* = 8.5 Hz, 2H), 7.47 (d, *J* = 8.5 Hz, 2H), 6.88 – 6.83 (m, 2H), 6.69 (dd, *J* = 9.0, 2.4 Hz, 1H), 6.37 (s, 1H), 3.81 (s, 3H), 3.74 (s, 2H),

2.73 – 2.63 (m, 1H), 2.34 (s, 3H), 1.60 – 1.49 (m, 1H), 1.41 – 1.31 (m, 1H), 1.22 (s, 12H), 1.20 – 1.11 (m, 6H), 0.80 (t, J = 6.7 Hz, 3H);

^{13}C NMR (126 MHz, $CDCl_3$) δ 173.7, 168.4, 156.5, 139.9, 136.9, 133.6, 131.3, 131.0, 130.0, 129.4, 115.3, 112.9, 111.0, 100.6, 81.7, 55.9, 31.9, 31.2, 29.0, 27.4, 25.3, 25.1, 22.6, 14.1, 13.5;

^{11}B NMR (160 MHz, $CDCl_3$) δ 21.9;

HRMS (ESI) calcd. for $C_{31}H_{40}BClN_2NaO_5$ $[M+Na]^+$ m/z 589.2611, found 589.2596;

IR (neat, cm^{-1}) 3249, 2928, 1678, 1603, 1323, 1146;

m.p. 150 – 151 °C;

$[\alpha]_D^{25}$ = –34.5 (c = 0.65, $CHCl_3$);

HPLC analysis: the *ee* (98%) was determined using a CHIRALPAK® AD-H column, 10% *i*PrOH in hexane, 1.0 mL/min, 240 nm UV detector, t_R (major) = 5.7 min, t_R (minor) = 6.7 min.

3. Synthetic Application

a. Gram-Scale Experiment

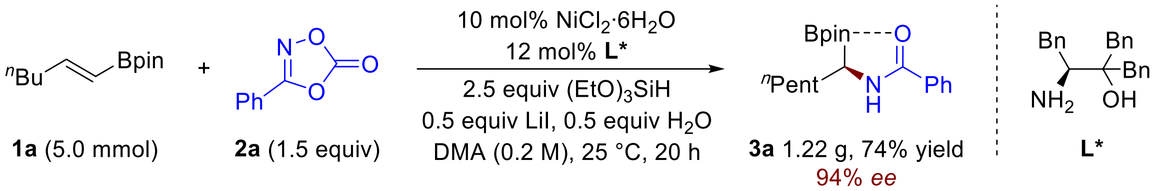

In a nitrogen-filled glove box, to an oven-dried 100 mL round bottom flask equipped with a magnetic stir bar was added $NiCl_2 \cdot 6H_2O$ (118.8 mg, 10 mol%), **L*** (198.9 mg, 12 mol%), LiI (334.6 mg, 2.5 mmol, 0.50 equiv), 3-phenyl-1,4,2-dioxazol-5-one (**2a**) (1.224 g, 7.5 mmol, 1.5 equiv). The flask was sealed with a rubber stopper, removed from the glove box and equipped with a N_2 balloon, and anhydrous DMA (25 mL, 0.20 M) was added via syringe and the mixture was stirred for 10 min at 25 °C (water bath), at which time (*E*)-2-(hex-1-en-1-yl)-4,4,5,5-tetramethyl-1,3,2-dioxaborolane (**1a**) (1.051g, 5.0 mmol, 1.0 equiv), H_2O (45 μL, 2.5 mmol, 0.50 equiv) were added via syringe and the mixture was stirred for 5 min and then $(EtO)_3SiH$ (2.053 g, 12.5 mmol, 2.5 equiv) was added to the resulting mixture. The mixture was stirred at 25 °C for up to 20 h. After the reaction was complete, *n*-dodecane (500 μL) was added as an internal standard for GC analysis, and the reaction was quenched upon the addition of H_2O (150 mL). The mixture was extracted with Et_2O. The organic layer was concentrated. The crude material was purified by flash column chromatography (petroleum ether/EtOAc = 2:1) to provide **3a** as a white solid in 74% yield (1.220 g). The *ee* (94%) was determined via chiral HPLC analysis.

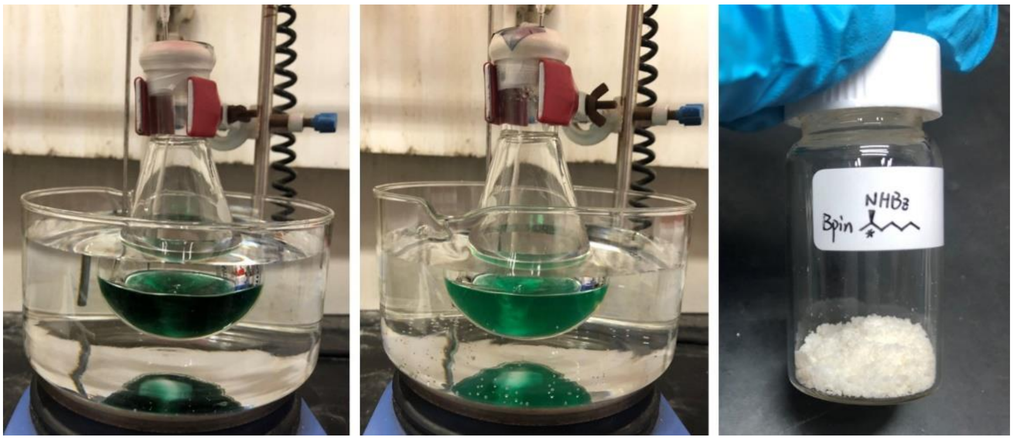

Supplementary Figure 1: (Left) All the reagents were added and stirred at 25 °C for 10 min. (a

dark green homogeneous mixture) **(Center)** The mixture was stirred at 25 °C for 20 h. (a light green homogeneous mixture) **(Right)** Purified product **3a**. (A white solid (1.220 g) was obtained after purification by column chromatography.).

b. One-pot asymmetric hydroamidation *w/o* isolation of alkenyl boronate

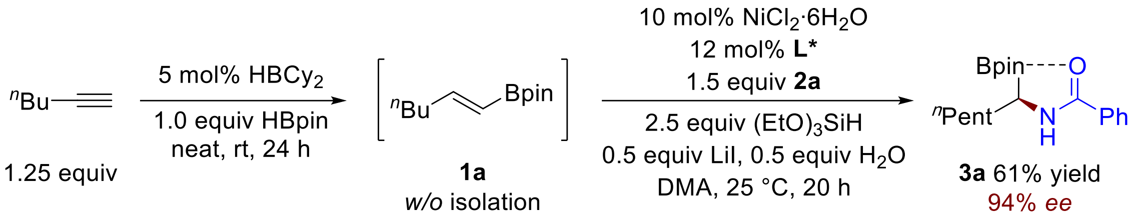

The procedure was modified from the previous literature[7]. In a nitrogen-filled glove box, to an oven-dried 8 mL screw-cap vial equipped with a magnetic stir bar was added freshly prepared dicyclohexylborane (3.6 mg, 5 mol%), 1-hexyne (41.1 mg, 0.5 mmol, 1.25 equiv) and pinacolborane (58 µL, 0.4 mmol, 1.0 equiv). The reaction mixture was stirred for 18 hours. Afterwards, the volatile materials were removed under reduced pressure at room temperature for 1 h. The vial was refilled with N_2 and transferred to the nitrogen-filled glove box. $NiCl_2 \cdot 6H_2O$ (9.6 mg, 10 mol%), **L*** (16.0 mg, 12 mol%), LiI (26.8 mg, 0.20 mmol, 0.50 equiv), 3-phenyl-1,4,2-dioxazol-5-one (**2a**) (97.8 mg, 0.60 mmol, 1.5 equiv) and anhydrous DMA (2.0 mL, 0.20 M) were added to the vial. The mixture was stirred for 10 min at room temperature, at which time H_2O (3.6 µL, 0.20 mmol, 0.50 equiv) and $(EtO)_3SiH$ (184 µL, 1.0 mmol, 2.5 equiv) were added to the resulting mixture in this order. The tube was sealed with a teflon-lined screw cap, removed from the glove box and the reaction was stirred at 25 °C water bath for up to 20 h (the mixture was stirred at 1000 rpm). After the reaction was complete, *n*-dodecane (40 µL) was added as an internal standard for GC analysis, and the reaction was quenched upon the addition of H_2O. The mixture was extracted with Et_2O. The organic layer was concentrated. The crude material was purified by flash column chromatography (petroleum ether/EtOAc = 2:1) to provide **3a** as a white solid in 61% yield (80.6 mg). The *ee* (94%) was determined via chiral HPLC analysis.

c. Concise Synthetic Route to Vaborbactam

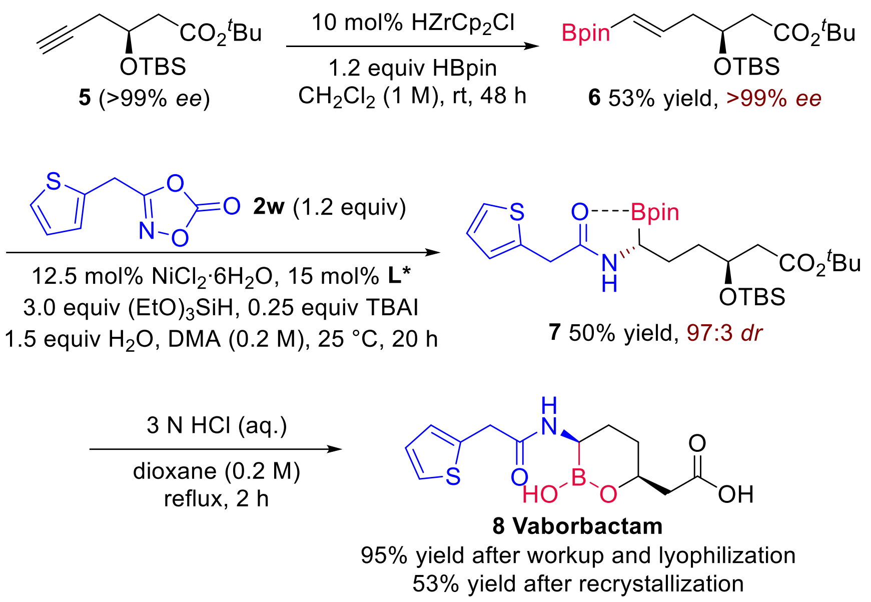

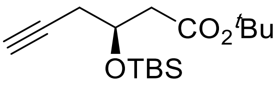

***tert*-Butyl (*S*)-3-((*tert*-butyldimethylsilyl)oxy)hex-5-ynoate** (Figure 5, **5**). The title compound was prepared according to the known literature method[2]. Spectral data match the previously reported.

^{1}H NMR (500 MHz, $CDCl_3$) δ 4.29 – 4.17 (m, 1H), 2.58 (dd, J = 15.4, 4.8 Hz, 1H), 2.45 (dd, J = 15.4, 7.1 Hz, 1H), 2.40 (dd, J = 6.1, 2.7 Hz, 2H), 1.99 (t, J = 2.7 Hz, 1H), 1.45 (s, 9H), 0.87 (s, 9H), 0.10 (s, 3H), 0.08 (s, 3H);

^{13}C NMR (126 MHz, $CDCl_3$) δ 170.7, 81.0, 80.6, 70.6, 68.1, 43.1, 28.3, 27.5, 25.9, 18.1, -4.5, -4.7;

HRMS (ESI) calcd. for $C_{16}H_{30}NaO_3Si$ $[M+Na]^+$ m/z 321.1856, found 321.1847;

$[\alpha]_D^{25}$ = +28.9 (c = 1.02, $CHCl_3$), >99% *ee*;

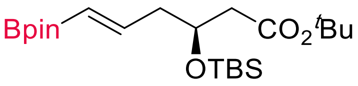

***tert*-Butyl (*S*,*E*)-3-((*tert*-butyldimethylsilyl)oxy)-6-(4,4,5,5-tetramethyl-1,3,2-dioxaborolan-2-yl)hex-5-enoate** (Figure 5, **6**). Under N_2 atmosphere, to an oven-dried round bottom flask equipped with a stir bar, Schwartz's reagent (0.351 g, 10 mol%), CH_2Cl_2 (13 mL) and *tert*-butyl (*S*)-3-((*tert*-butyldimethylsilyl)oxy)hex-5-ynoate (**5**)

(4.060 g, 13.6 mmol, 1.0 equiv) were added and stirred for 5 minutes. At 0 °C, pinacolborane (2.089 g, 16.3 mmol, 1.2 equiv) was added dropwise to the mixture. Then, the mixture was allowed to warm to rt and stirred for 48 hours. The reaction was quenched with H_2O carefully, extracted with Et_2O, and concentrated under reduced pressure. The crude mixture was purified by silica gel chromatography (petroleum ether/EtOAc = 30:1) to afford the titled (*E*)-alkenyl boronate as a colorless oil in 53% yield (3.068 g).

^{1}H NMR (500 MHz, $CDCl_3$) δ 6.57 (dt, *J* = 17.9, 7.0 Hz, 1H), 5.47 (d, *J* = 17.9 Hz, 1H), 4.22 – 4.13 (m, 1H), 2.40 – 2.32 (m, 4H), 1.43 (s, 9H), 1.25 (s, 12H), 0.86 (s, 9H), 0.06 (s, 3H), 0.05 (s, 3H);

^{13}C NMR (126 MHz, $CDCl_3$) δ 171.0, 150.1, 83.2, 80.4, 68.9, 44.4, 43.7, 28.3, 26.0, 24.9, 24.9, 18.2, -4.3, -4.6;

^{11}B NMR (160 MHz, $CDCl_3$) δ 29.9;

HRMS (ESI) calcd. for $C_{22}H_{43}BNaO_5Si$ $[M+Na]^+$ m/z 449.2865, found 449.2853;

IR (neat, cm^{-1}) 2978, 2930, 1730, 1640, 1361, 1142, 832;

$[\alpha]_D^{25}$ = +13.8 (c = 1.09, $CHCl_3$);

HPLC analysis: the *ee* (>99%) was determined using a CHIRALPAK® IF-3 column, 0.5% *i*PrOH in hexane, 1.0 mL/min, 220 nm UV detector, t_R (minor) = 6.9 min, t_R (major) = 8.8 min.

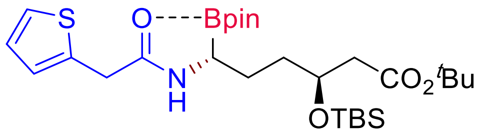

tert*-Butyl (3*S*,6*R*)-3-((*tert*-butyldimethylsilyl)oxy)-6-(4,4,5,5-tetramethyl-1,3,2-dioxaborolan-2-yl)-6-(2-(thiophen-2-yl)acetamido)hexanoate** (Figure 5, **7**). From *tert*-butyl (*S*,*E*)-3-((*tert*-butyldimethylsilyl)oxy)-6-(4,4,5,5-tetramethyl-1,3,2-dioxaborolan-2-yl)hex-5-enoate (**6**) (85.3 mg, 0.20 mmol, 1.0 equiv) and 3-(thiophen-2-ylmethyl)-1,4,2-dioxazol-5-one (**2w**) (44.0 mg, 0.24 mmol, 1.2 equiv), the title compound was prepared following the general procedure **A** using $NiCl_2·6H_2O$ (5.9 mg, 12.5 mol%), **L (12.0 mg, 15 mol%), TBAI (18.5 mg, 0.050 mmol, 0.25 equiv, instead

of LiI), H_2O (5.4 μL, 0.30 mmol, 1.5 equiv), $(EtO)_3SiH$ (110 μL, 0.60 mmol, 3.0 equiv), anhydrous DMA (1.0 mL). The reaction mixture was stirred for 20 h at 25 °C. The crude material was purified by flash column chromatography (petroleum ether/EtOAc = 2:1) to provide the title compound as a yellow oil in 50% yield (56.4 mg).

^{1}H NMR (500 MHz, $CDCl_3$) δ 7.25 (dd, J = 5.1, 0.9 Hz, 1H), 6.98 (dd, J = 5.1, 3.5 Hz, 1H), 6.93 (d, J = 3.1 Hz, 1H), 6.58 (s, 1H), 4.08 – 3.97 (m, 1H), 3.87 (s, 2H), 2.76 – 2.66 (m, 1H), 2.39 – 2.26 (m, 2H), 1.65 – 1.55 (m, 1H), 1.55 – 1.44 (m, 3H), 1.41 (s, 9H), 1.22 (s, 12H), 0.84 (s, 9H), 0.02 (s, 3H), 0.02 (s, 3H);

^{13}C NMR (126 MHz, $CDCl_3$) δ 173.9, 171.3, 133.9, 128.2, 127.7, 126.2, 81.7, 80.5, 68.9, 43.6, 35.0, 34.1, 28.3, 26.4, 26.0, 25.3, 25.1, 18.1, -4.4, -4.6;

^{11}B NMR (160 MHz, $CDCl_3$) δ 21.8;

HRMS (ESI) calcd. for $C_{28}H_{50}BNNaO_6SSi$ $[M+Na]^+$ m/z 590.3113, found 590.3103;

IR (neat, cm^{-1}) 2968, 2929, 1729, 1607, 1149, 834;

$[\alpha]_D^{25}$ = –20.2 (c = 1.00, $CHCl_3$);

HPLC analysis: the *dr* (97:3) was determined using a CHIRALPAK® AD-H column, 5% *i*PrOH in hexane, 1.0 mL/min, 240 nm UV detector, t_R (major) = 4.3 min, t_R (minor) = 6.5 min.

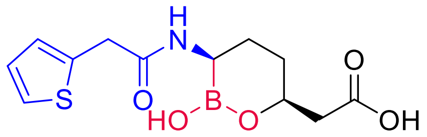

2-((3*R*,6*S*)-2-Hydroxy-3-(2-(thiophen-2-yl)acetamido)-1,2-oxaborinan-6-yl)acetic acid[3] (Figure 5, **8**). The title compound was prepared according to a known method with a similar substrate[3]. To a solution of *tert*-butyl (3*S*,6*R*)-3-((*tert*-butyldimethylsilyl)oxy)-6-(4,4,5,5-tetramethyl-1,3,2-dioxaborolan-2-yl)-6-(2-(thiophen-2-yl)acetamido)hexanoate (**7**) (398 mg, 0.70 mmol) in 1,4-dioxane (2 mL) was added 2 mL of 3 N HCl (aq.). The reaction mixture was heated at reflux for 2 h, after which the cooled reaction mixture was diluted with water (2 mL) and extracted with Et_2O (3 × 6 mL). The aqueous layer was concentrated to afford a sticky residue which was azeotroped with MeCN (3 × 8 mL), dissolved in 20% dioxane-water, and

lyophilized to afford a white powder (198 mg, 95%).

The crude product (150 mg) was suspended in EtOAc (3 mL). Water (0.5 mL) was added, and most of the compound appeared to go into the water layer. After sonicating for about 30 min, a white precipitate formed. The solid was collected by filtration, washed with Et_2O and hexane. The solid was dissolved in 20% dioxane-water, and lyophilized to provide the title compound as a white solid in 53% yield (79.2 mg).

^{1}H NMR (500 MHz, CD_3OD) δ 7.34 (dd, *J* = 5.2, 1.2 Hz, 1H), 7.09 – 7.02 (m, 1H), 7.00 (dd, *J* = 5.2, 3.5 Hz, 1H), 4.14 – 4.04 (m, 1H), 3.97 (s, 2H), 2.66 – 2.57 (m, 1H), 2.37 (dd, *J* = 15.0, 7.3 Hz, 1H), 2.25 (dd, *J* = 15.0, 5.8 Hz, 1H), 1.78 – 1.68 (m, 1H), 1.68 – 1.52 (m, 2H), 1.11 – 0.97 (m, 1H);

^{13}C NMR (126 MHz, CD_3OD) δ 177.8, 175.6, 135.2, 128.8, 128.2, 126.7, 70.5, 44.4, 32.6, 28.5, 27.6;

^{11}B NMR (160 MHz, CD_3OD) δ 11.7;

HRMS (ESI) calcd. for $C_{12}H_{15}BNO_4S$ $[M–H_2O+H]^+$ m/z 280.0809, found 280.0802;

IR (neat, cm^{-1}) 3511, 2941, 1718, 1607, 1225, 1183, 701;

$[\alpha]_D^{25}$ = –6.1 (c = 0.85, CH_3OH).

4. Mechanistic Experiments

a. Nonlinear effect study

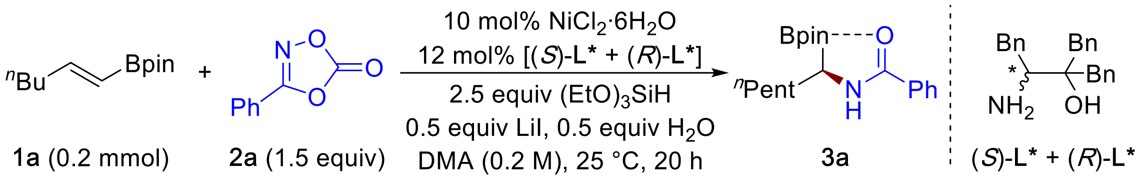

Supplementary Table 1: Nonlinear effect study

Entry	*ee* (%) of mixed L*	(*S*)-L* (mg)	(*R*)-L* (mg)	*ee* (%) of 3a
1	20	4.8	3.2	17
2	40	5.6	2.4	37
3	60	6.4	1.6	52
4	80	7.2	0.8	73
5	99	8.0	0	95

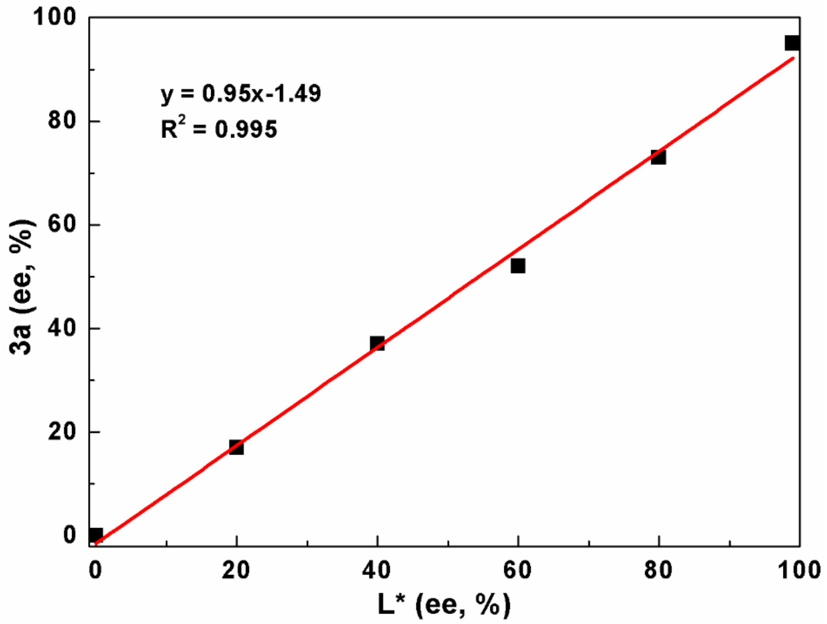

5 parallel reactions at 0.20 mmol scale were performed following general procedure **A**. In a nitrogen-filled glove box, to an oven-dried 8 mL screw-cap vial equipped with a magnetic stir bar was added $NiCl_2 \cdot 6H_2O$ (4.8 mg, 10 mol%), specified amount of (*S*)-**L*** and (*R*)-**L*** (to provide the enantiomeric composition of **L***) as listed in Supplementary Table 1, LiI (13.4 mg, 0.10 mmol, 0.50 equiv), 3-phenyl-1,4,2-dioxazol-5-one (**2a**) (48.9 mg, 0.30 mmol, 1.5 equiv) and anhydrous DMA (1.0 mL,

0.20 M). The mixture was stirred for 10 min at room temperature, at which time (*E*)-2-(hex-1-en-1-yl)-4,4,5,5-tetramethyl-1,3,2-dioxaborolane (**1a**) (42.0 mg, 0.20 mmol, 1.0 equiv), H_2O (1.8 μL, 0.10 mmol, 0.50 equiv) and $(EtO)_3SiH$ (92 μL, 0.50 mmol, 2.5 equiv) were added to the resulting mixture in this order. The tube was sealed with a teflon-lined screw cap, removed from the glove box and the reaction was stirred at 25 °C water bath (the mixture was stirred at 800 rpm). The reactions were stopped at the indicated reaction time, quenched upon the addition of H_2O and extracted with Et_2O. *n*-Dodecane (20 μL) was added as an internal standard for GC analysis. The organic layer was concentrated to give the crude product. The product was purified by flash column chromatography (petroleum ether/EtOAc = 2:1) for each substrate. The enantiomeric excesses (% *ee*) were determined by HPLC analysis using chiral stationary phases.

Note: A linear correlation between the *ee* value of the ligand **L*** and that of the product **3a** was observed, consistent with the monomeric nature of the active catalyst.

b. Isotopic labelling experiments

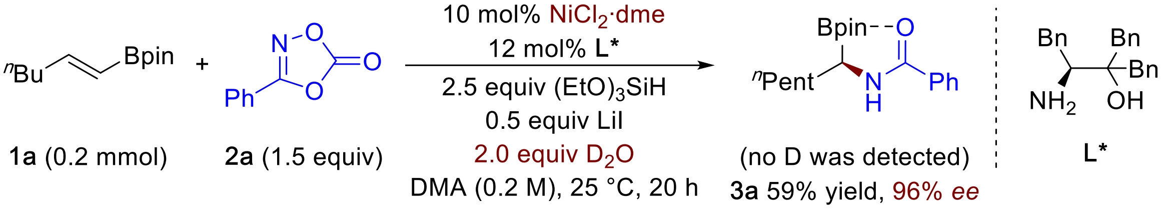

Following the general procedure **A**, in a nitrogen-filled glove box, to an oven-dried 8 mL screw-cap vial equipped with a magnetic stir bar was added $NiCl_2$·dme (4.4 mg, 10 mol%), **L*** (8.0 mg, 12 mol%), LiI (13.4 mg, 0.10 mmol, 0.50 equiv), 3-phenyl-1,4,2-dioxazol-5-one (**2a**) (48.9 mg, 0.30 mmol, 1.5 equiv) and anhydrous DMA (1.0 mL, 0.20 M) were added, and the mixture was stirred for 10 min at 25 °C, at which time (*E*)-2-(hex-1-en-1-yl)-4,4,5,5-tetramethyl-1,3,2-dioxaborolane (**1a**) (42.0 mg, 0.20 mmol, 1.0 equiv), D_2O (7.2 μL, 0.40 mmol, 2.0 equiv) and $(EtO)_3SiH$ (92 μL, 0.50 mmol, 2.5 equiv) were added to the resulting mixture in this order. The tube was sealed with a teflon-lined screw cap, removed from the glove box and the reaction was stirred at 25 °C for up to 20 h (the mixture was stirred at 800 rpm). The reaction was quenched upon the addition of H_2O, and the mixture was extracted with Et_2O. The organic layer was concentrated to give the crude product. *n*-Dodecane (20 μL) was added as an internal standard for GC analysis. The crude material was purified by flash column chromatography (petroleum ether/EtOAc = 2:1) to provide **3a** as a colorless liquid in 57% yield (37.7 mg). The *ee* (96%) of **3a** was determined via HPLC analysis.

Note: Althought 2 equiv D_2O was added, no deuterium incorporation was observed in the product (**3a**), eliminating the possibility of a protic reagent as the hydride source.

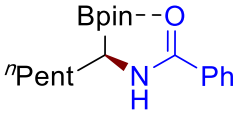

(no D was detected)

(*R*)-*N*-(1-(4,4,5,5-Tetramethyl-1,3,2-dioxaborolan-2-yl)hexyl)benzamide (Figure 6, **3a**, no D was detected).

^{1}H NMR (500 MHz, $CDCl_3$) δ 8.53 (s, 1H), 7.80 (d, *J* = 7.6 Hz, 2H), 7.45 (t, *J* = 7.4 Hz, 1H), 7.32 (t, *J* = 7.6 Hz, 2H), 2.79 (t, *J* = 6.5 Hz, 1H), 1.75 – 1.65 (m, 1H), 1.61 –

1.52 (m, 1H), 1.49 – 1.37 (m, 2H), 1.34 – 1.28 (m, 4H), 1.26 (s, 12H), 0.88 (t, J = 7.0 Hz, 3H);

^{13}C NMR (126 MHz, $CDCl_3$) δ 170.9, 133.1, 128.6, 128.3, 127.9, 81.2, 32.1, 31.3, 27.6, 25.4, 25.2, 22.7, 14.2;

^{11}B NMR (160 MHz, $CDCl_3$) δ 17.6;

^{2}H NMR (92 MHz, $CHCl_3$) no D was detected.

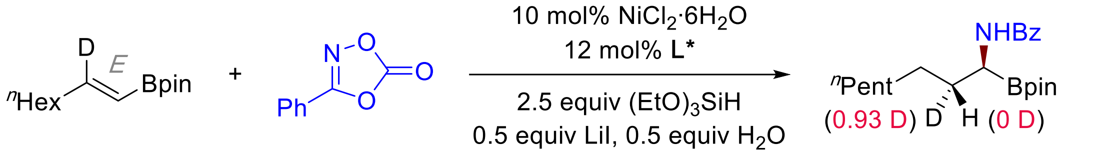

1b-D (93% D) **2a** (1.5 equiv) **3b-D** 70% yield, 96% *ee*

Following the general procedure **A**, in a nitrogen-filled glove box, to an oven-dried 8 mL screw-cap vial equipped with a magnetic stir bar was added $NiCl_2·6H_2O$ (4.8 mg, 10 mol%), **L*** (8.0 mg, 12 mol%), LiI (13.4 mg, 0.50 equiv), 3-phenyl-1,4,2-dioxazol-5-one (**2a**) (48.9 mg, 0.30 mmol, 1.5 equiv) and anhydrous DMA (1.0 mL) were added, and the mixture was stirred for 10 min at 25 °C, at which time (*E*)-4,4,5,5-tetramethyl-2-(oct-1-en-1-yl-2-d)-1,3,2-dioxaborolane (**1b-D**, 93% D) (47.8 mg, 0.20 mmol, 1.0 equiv), H_2O (1.8 µL, 0.50 equiv) and $(EtO)_3SiH$ (92 µL, 2.5 equiv) were added to the resulting mixture in this order. The tube was sealed with a teflon-lined screw cap, removed from the glove box and the reaction was stirred at 25 °C for up to 20 h (the mixture was stirred at 800 rpm). The reaction was quenched upon the addition of H_2O, and the mixture was extracted with Et_2O. The organic layer was concentrated to give the crude product. *n*-Dodecane (20 µL) was added as an internal standard for GC analysis. The crude material was purified by flash column chromatography (petroleum ether/EtOAc = 2:1) to provide **3b-D** as a colorless liquid in 70% yield (50.1 mg). The *ee* (96%) of **3b-D** was determined via HPLC analysis.

Note: Diastereomerically pure **3b-D** was obtained from this reaction, indicating that *syn*-hydronickellation is involved in the enantio-determining step.

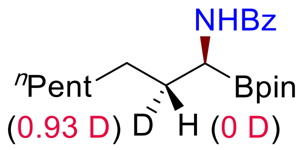

***N*-((1*S*,2*R*)-1-(4,4,5,5-Tetramethyl-1,3,2-dioxaborolan-2-yl)octyl-2-d)benzamide** (Figure 6, **3b-D**).

^{1}H NMR (500 MHz, $CDCl_3$) δ 8.45 (s, 1H), 7.80 (d, J = 7.4 Hz, 2H), 7.46 (t, J = 7.4 Hz, 1H), 7.32 (t, J = 7.7 Hz, 2H), 2.79 (d, J = 6.8 Hz, 1H), 1.74 – 1.66 (m, 0.07H), 1.58 – 1.51 (m, 1H), 1.46 – 1.36 (m, 2H), 1.33 – 1.20 (m, 20H), 0.87 (t, J = 6.9 Hz, 3H);

^{13}C NMR (126 MHz, $CDCl_3$) δ 170.9, 133.1, 128.6, 128.3, 128.0, 81.2, 32.0, 31.0 (t, J = 18.9 Hz), 29.9, 29.4, 27.9, 25.4, 25.2, 22.8, 14.3;

^{11}B NMR (160 MHz, $CDCl_3$) δ 18.0;

^{2}H NMR (92 MHz, $CHCl_3$) δ 1.68 (corresponding to the missing 0.93H);

HRMS (ESI) calcd. for $C_{21}H_{33}DBNNaO_3$ $[M+Na]^+$ m/z 383.2587, found 383.2576;

IR (neat, cm^{-1}) 3196, 2923, 2854, 1610, 1154, 1111, 706;

$[\alpha]_D^{25}$ = –38.3 (c = 1.00, $CHCl_3$);

HPLC analysis: the *ee* (96%) was determined using a CHIRALCEL® OD-H column, 8% *i*PrOH in hexane, 0.5 mL/min, 254 nm UV detector, t_R (minor) = 7.3 min, t_R (major) = 7.9 min (see Supplementary Figure 192).

c. Capture of metal-nitrenoid intermediate

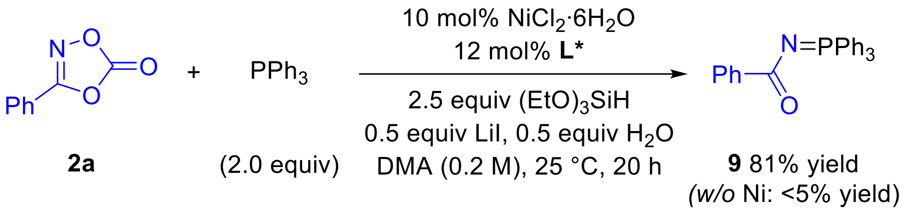

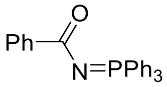

N*-(Triphenyl-λ^5-phosphaneylidene)benzamide** (Figure 6, **9**). In a nitrogen-filled glove box, to an oven-dried 8 mL screw-cap vial equipped with a magnetic stir bar was added $NiCl_2 \cdot 6H_2O$ (4.8 mg, 10 mol%), **L (8.0 mg, 12 mol%), LiI (13.4 mg, 0.10 mmol, 0.50 equiv), 1,4,2-dioxazol-5-one (32.6 mg, 0.20 mmol, 1.0 equiv), PPh_3 (104.9 mg, 0.40 mmol, 2.0 equiv), anhydrous DMA (1.0 mL, 0.20 M) were added and the mixture was stirred for 10 min at room temperature. H_2O (1.8 μL, 0.10 mmol, 0.50 equiv) and $(EtO)_3SiH$ (92 μL, 0.50 mmol, 2.5 equiv) were added to the resulting mixture in this order. The tube was sealed with a teflon-lined screw cap, removed from the glove box and the reaction was stirred at 25 °C water bath for up to 20 h (the mixture was stirred at 800 rpm). After the reaction was complete, the reaction was quenched upon the addition of H_2O, and the mixture was extracted with EtOAc. The organic layer was concentrated to give the crude product. The crude material was purified by flash column chromatography (petroleum ether/EtOAc = 4:1) to provide the title compound as a white solid in 81% yield (61.5 mg).

^{1}H NMR (400 MHz, $CDCl_3$) δ 8.37 (dd, *J* = 8.0, 1.5 Hz, 2H), 7.94 – 7.78 (m, 6H), 7.60 – 7.53 (m, 3H), 7.53 – 7.37 (m, 9H);

^{13}C NMR (101 MHz, $CDCl_3$) δ 176.2, 138.6, 133.3 (d, *J* = 9.9 Hz), 132.4, 130.8, 129.6, 128.8 (d, *J* = 12.3 Hz), 127.8;

^{31}P NMR (202 MHz, $CDCl_3$) δ 20.7;

HRMS (ESI) calcd. for $C_{25}H_{20}NNaOP$ $[M+Na]^+$ m/z 404.1175, found 404.1163;

IR (neat, cm^{-1}) 3057, 1594, 1557, 1328, 1106, 719, 690, 515;

m.p. 198 – 199 °C.

d. Monitoring of the Reaction Progress

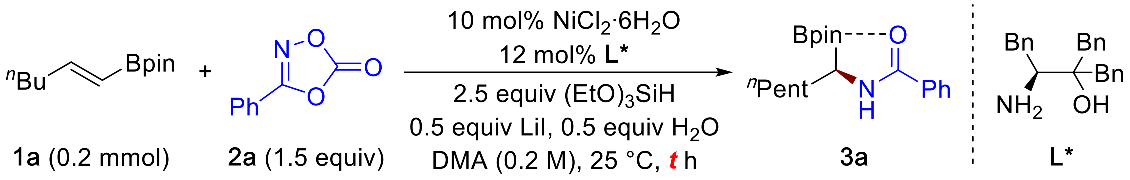

Supplementary Table 2: Yield and *ee* of **3a** as a function of time

Entry	*t* (h)	yield (%)	*ee* (%)
1	1	28	95
2	2	44	95
3	4	59	95
4	6.5	75	95
5	20	75	95
6	26	75	95
7	45	75	95

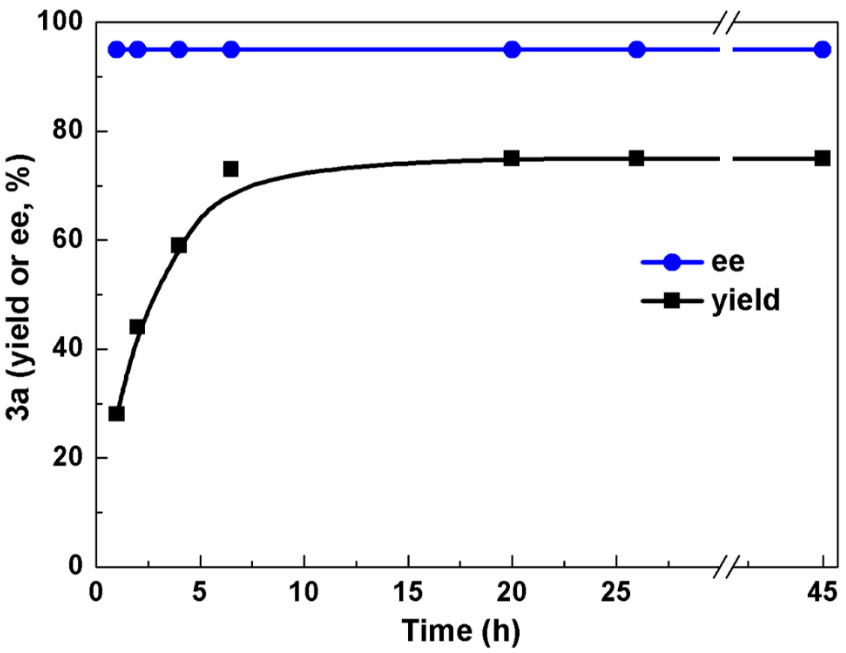

7 parallel reactions at 0.20 mmol scale were performed following general procedure **A**. In a nitrogen-filled glove box, to an oven-dried 8 mL screw-cap vial equipped with a magnetic stir bar was added $NiCl_2 \cdot 6H_2O$ (4.8 mg, 10 mol%), **L*** (8.0 mg, 12 mol%), LiI (13.4 mg, 0.10 mmol, 0.50 equiv), 3-phenyl-1,4,2-dioxazol-5-one (**2a**) (48.9 mg, 0.30 mmol, 1.5 equiv) and anhydrous DMA (1.0 mL, 0.20 M). The mixture was stirred

for 10 min at room temperature, at which time (*E*)-2-(hex-1-en-1-yl)-4,4,5,5-tetramethyl-1,3,2-dioxaborolane (**1a**) (42.0 mg, 0.20 mmol, 1.0 equiv), H_2O (1.8 μL, 0.10 mmol, 0.50 equiv) and $(EtO)_3SiH$ (92 μL, 0.50 mmol, 2.5 equiv) were added to the resulting mixture in this order. The tube was sealed with a teflon-lined screw cap, removed from the glove box and the reaction was stirred at 25 °C water bath (the mixture was stirred at 800 rpm). The reactions were stopped at the indicated reaction time, quenched upon the addition of H_2O and extracted with Et_2O. *n*-Dodecane (20 μL) was added as an internal standard for GC analysis. The organic layer was concentrated to give the crude product. The product was purified by flash column chromatography (petroleum ether/EtOAc = 2:1) for each substrate. The enantiomeric excesses (% *ee*) were determined by HPLC analysis using chiral stationary phases.

Note: During the entire reaction, the *ee* of the product remained unchanged.

5. Determination of the Absolute Configuration

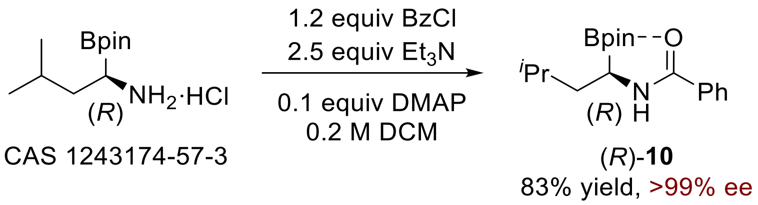

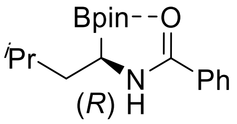

(*R*)-*N*-(3-Methyl-1-(4,4,5,5-tetramethyl-1,3,2-dioxaborolan-2-yl)butyl)benzamide ((*R*)-**10**). To a stirred solution of (*R*)-3-methyl-1-(4,4,5,5-tetramethyl-1,3,2-dioxaborolan-2-yl)butan-1-amine hydrochloride (CAS 1243174-57-3, commercial available optically pure compound) (49.9 mg, 0.20 mmol, 1.0 equiv), dimethylaminopyridine (DMAP, 2.4 mg, 0.020 mmol, 0.10 equiv) in dry CH_2Cl_2 (15 mL) at 0 °C was added Et_3N (70 μL, 0.5 mmol, 2.5 equiv). After 10 minutes, benzoyl chloride (33.7 mg, 0.24 mmol, 1.2 equiv) were added. The resulting reaction mixture was allowed to warm to rt and the stirring was continued for overnight. After the reaction was complete, the reaction was quenched upon the addition of H_2O, and the mixture was extracted with EtOAc. After removal of solvent under reduced pressure, the crude material was purified by flash column chromatography (petroleum ether/EtOAc = 2:1) to provide the title compound **10** as a white solid in 83% yield (52.4 mg), and the spectral data match **3d**.

$[\alpha]_D^{25}$ = –40.9 (c = 0.94, $CHCl_3$);

HPLC analysis: the *ee* (>99%) was determined using a CHIRALPAK® AD-H column, 5% *i*PrOH in hexane, 1.0 mL/min, 240 nm UV detector, t_R (minor) = 4.8 min, t_R (major) = 5.6 min, (*R*) configuration (see Supplementary Figure 197).

Note: The absolute configuration of **3d** was determined to be (*R*) by comparison of the optical rotation and HPLC peak of the title compound with **3d** ($[\alpha]_D^{25}$ = –37.3 (c = 0.96, $CHCl_3$); HPLC analysis: the *ee* (92%) of **3d** was determined using a CHIRALPAK® AD-H column, 5% iPrOH in hexane, 1.0 mL/min, 240 nm UV detector, t_R (minor) = 4.8 min, t_R (major) = 5.5 min.).

6. Conditions Optimization

Supplementary Table 3: Effect of reaction parameters[a].

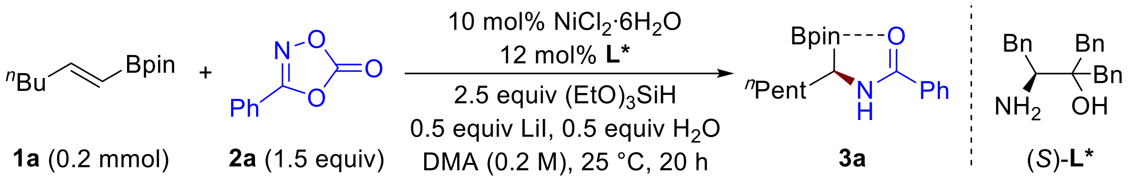

Entry	Variation from the standard conditions	Yield (%)	*ee* (%)
1	None	75 (71)	95
2	w/o Ni	0	-
3	w/o **L***	13	nd
4	$NiCl_2$·dme instead of $NiCl_2·6H_2O$	63	86
5	$NiBr_2·3H_2O$ instead of $NiCl_2·6H_2O$	58	61
6	$NiCl_2$ instead of $NiCl_2·6H_2O$	<5	nd
7	$Ni(NO_3)_2·6H_2O$ instead of $NiCl_2·6H_2O$	<5	nd
8	NiI_2 instead of $NiCl_2·6H_2O$	8	nd
9	$NiI_2·xH_2O$ instead of $NiCl_2·6H_2O$	8	nd
10	w/o LiI	46	97
11	NaI instead of LiI	67	94
12	TBAI instead of LiI	72	95
13	DMMS instead of $(EtO)_3SiH$	69	96
14	DEMS instead of $(EtO)_3SiH$	20	94
15	$(MeO)_3SiH$ instead of $(EtO)_3SiH$	67	95
16	PMHS instead of $(EtO)_3SiH$	<5	nd
17	w/o H_2O	58	81
18	MeOH instead of H_2O	73	89
19	EtOH instead of H_2O	72	88
20	^{i}PrOH instead of H_2O	72	85
21	^{t}BuOH instead of H_2O	64	80
22	DMF instead of DMAc	11	nd
23	DMPU instead of DMAc	13	nd
24	NMP instead of DMAc	49	96
25	THF instead of DMAc	12	nd
26	DCE instead of DMAc	<5	nd
27	10 °C instead of 25 °C	59	97

28	40 °C instead of 25 °C	74	85
29	under air in a closed vial	68	94
30	2 equiv H_2O	70	96

[a] Yields determined by GC using *n*-dodecane as the internal standard, the yield in parentheses is the isolated yield (0.20 mmol scale). Enantioselectivities were determined by chiral HPLC analysis.

Supplementary Table 4: Effect of Ligands[a].

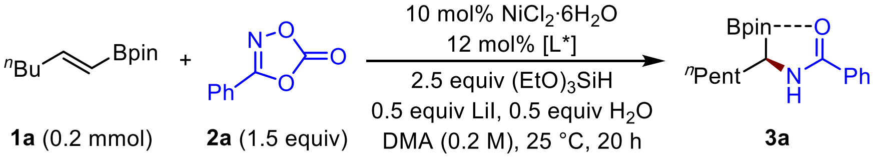

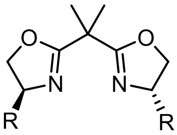

L4, R = Ph, 18% yield, 56% *ee*
L7, R = ^{i}Pr, 6% yield

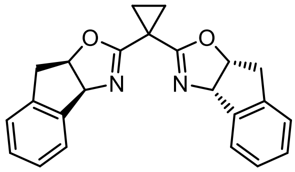

L8, 0% yield

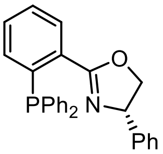

L9, 18% yield, 0% *ee*

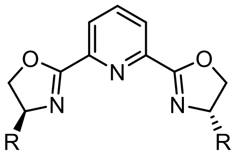

L10, R = Ph, 6% yield
L11, R = ^{i}Pr, 0% yield

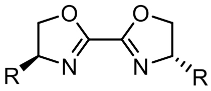

L12, R = Ph, 67% yield, 0% *ee*
L13, R = ^{i}Pr, 80% yield, 10% *ee*
L5, R = Bn, 74% yield, 44% *ee*

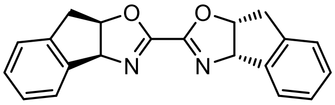

L14, 41% yield, 7% *ee*

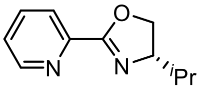

L15, 16% yield, 8% *ee*

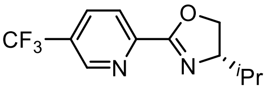

L16, 38% yield, 8% *ee*

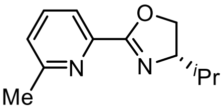

L6, 75% yield, 16% *ee*

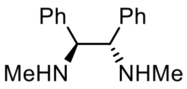

L17, 0% yield

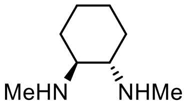

L18, 0% yield

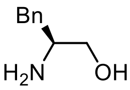

L3, 14% yield, 13% *ee*

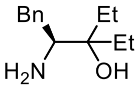

L2, 57% yield, 85% *ee*

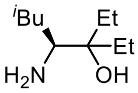

L19, 38% yield, 75% *ee*

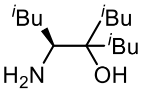

L20, 51% yield, 85% *ee*

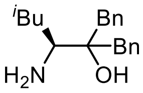

L21, 67% yield, 93% *ee*

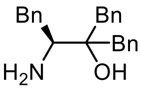

L*, 75% yield, 95% *ee*

[a] Yields determined by GC using *n*-dodecane as the internal standard, the yield in parentheses is the isolated yield (0.20 mmol scale). Enantioselectivities were determined by chiral HPLC analysis.

Supplementary Table 5: Effect of other olefins under standard conditions[a].

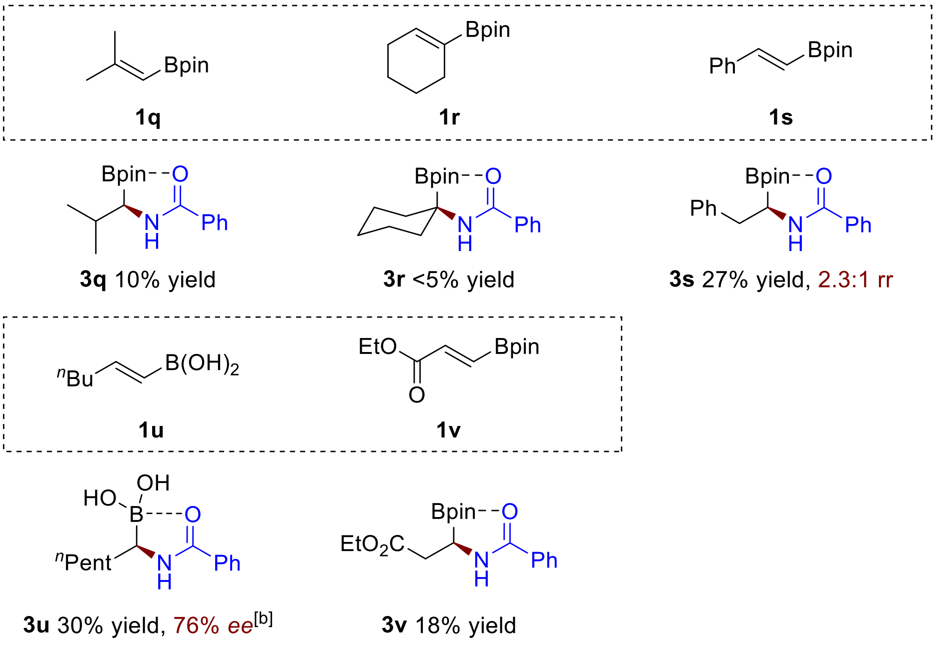

[a] reaction conditons were the same as **Figure 3**; [b] $(MeO)_2MeSiH$ was used instead of $(EtO)_3SiH$, the product was isolated as **3a** after treatment with pinacol.

7. Preparation of Substrates

a. Preparation of alkenyl boronates

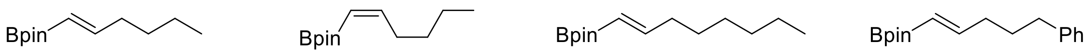

1a (Z)-1a 1b 1c

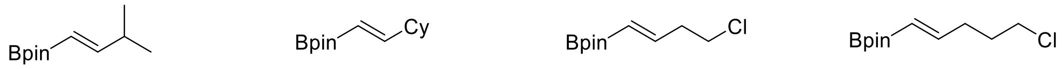

1d 1e 1f 1g

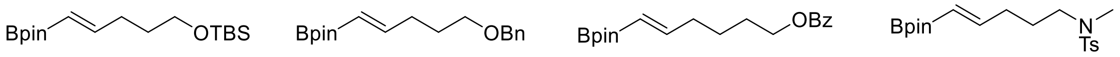

1h 1i 1j 1k

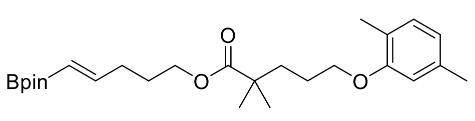

1l

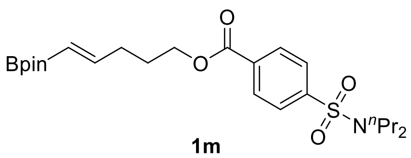

1m

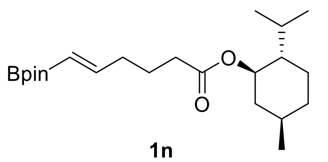

1n

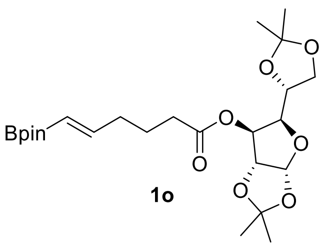

1o

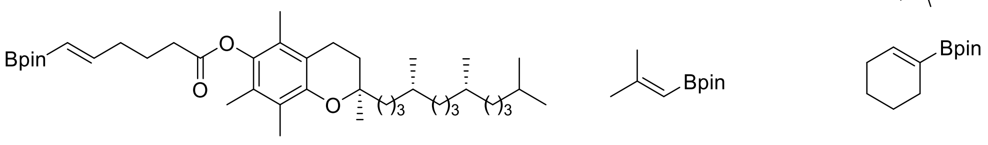

1p 1q 1r

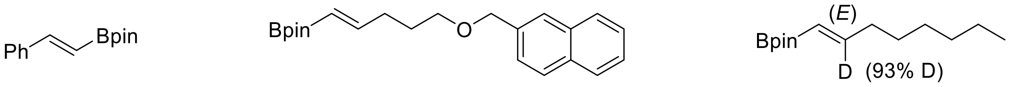

1s 1t 1b-D

Compounds **1a**, **1e**, **1q**, **1r**, **1s** are commercially available. Compounds (*Z*)-**1a**[4], **1b**[5], **1c**[7], **1d**[6], **1g**[5], **1h**[8], **1i**[9], **1j**[7],**1k**[5],and **1b-D**[10] were prepared according to the previously reported procedures.

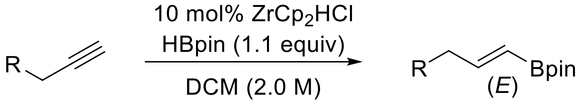

General procedure B for the synthesis of (*E*)-alkenyl boronates[11]. Under N_2 atmosphere, to an oven-dried round bottom flask equipped with a stir bar, Schwartz's reagent (10 mol%), CH_2Cl_2 (2.0 M) and alkyne (1.0 equiv) were added and stirred for 5 minutes. At 0 °C, pinacolborane (1.1 equiv) was added dropwise to the mixture. Then,

the mixture was allowed to warm to 30 °C and stirred for 24 hours. The reaction was quenched with H_2O, extracted with Et_2O, and concentrated under reduced pressure. The crude mixture was purified by silica gel chromatography (petroleum ether/EtOAc) to afford the (*E*)-alkenyl boronates.

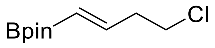

(*E*)-2-(4-chlorobut-1-en-1-yl)-4,4,5,5-tetramethyl-1,3,2-dioxaborolane (Figure 3, **1f**). From the 4-chlorobut-1-yne (0.89 g, 10.0 mmol), the title compound was prepared following the general procedure **B**. The crude material was purified by flash column chromatography (petroleum ether/EtOAc = 50:1) to provide the title compound as a colorless oil in 59% yield (1.27 g).

^{1}H NMR (500 MHz, $CDCl_3$) δ 6.57 (dt, *J* = 18.0, 6.4 Hz, 1H), 5.55 (dt, *J* = 18.0, 1.4 Hz, 1H), 3.57 (t, *J* = 7.0 Hz, 2H), 2.65 – 2.58 (m, 2H), 1.27 (s, 12H);

^{13}C NMR (126 MHz, $CDCl_3$) δ 149.0, 83.4, 43.0, 38.8, 24.9;

^{11}B NMR (160 MHz, $CDCl_3$) δ 29.7;

HRMS (ESI) calcd. for $C_{10}H_{18}BClNaO_2$ $[M+Na]^+$ m/z 239.0981, found 239.0978;

IR (neat, cm^{-1}) 2977, 1638, 1360, 1318, 1139, 1005.

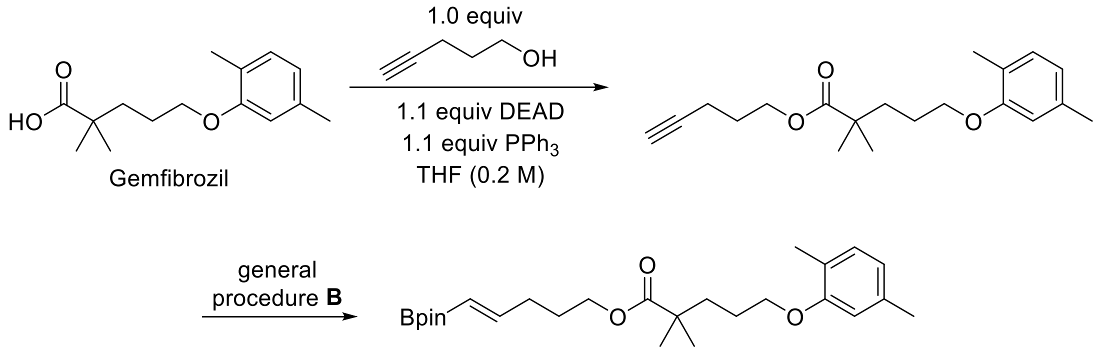

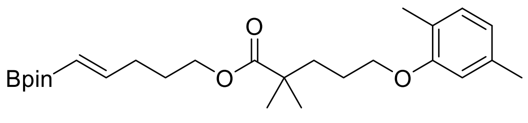

(*E*)-5-(4,4,5,5-Tetramethyl-1,3,2-dioxaborolan-2-yl)pent-4-en-1-yl 5-(2,5-dimethylphenoxy)-2,2-dimethylpentanoate (Figure 3, **1l**). To an anhydrous THF (40 mL) solution of gemfibrozil (10.0 mmol, 1.0 equiv, CAS 25812-30-0), pent-4-yn-1-ol

(0.84 g, 10 mmol, 1.0 equiv) and triphenylphosphine (PPh_3, 2.89 g, 11.0 mmol, 1.1 equiv) was slowly added a THF solution (20 mL) of diethyl azodicarboxylate (DEAD, 1.92 g, 11.0 mmol, 1.1 equiv) over 30 min at 0 °C. The resulting mixture was then stirred at rt overnight. The reaction was quenched with brine and extracted with ethyl acetate. The combined organic layers were dried over anhydrous Na_2SO_4, filtered, and concentrated. After removal of solvent under reduced pressure, the crude material was purified by flash column chromatography to provide the corresponding alkyne as a yellow oil in 68% yield (2.15 g).

From the resulting alkyne (1.90 g, 6.0 mmol), the title compound was prepared following the general procedure **B**. The crude material was purified by flash column chromatography (petroleum ether/EtOAc = 20:1) to provide the title compound as a colorless oil in 53% yield (1.41 g).

^{1}H NMR (500 MHz, $CDCl_3$) δ 7.00 (d, *J* = 7.5 Hz, 1H), 6.70 – 6.54 (m, 3H), 5.47 (dt, *J* = 17.9, 1.5 Hz, 1H), 4.07 (t, *J* = 6.5 Hz, 2H), 3.96 – 3.87 (m, 2H), 2.30 (s, 3H), 2.27 – 2.19 (m, 2H), 2.30 (s, 3H), 1.80 – 1.68 (m, 6H), 1.26 (s, 12H), 1.21 (s, 6H);

^{13}C NMR (126 MHz, $CDCl_3$) δ 177.9, 157.1, 152.9, 136.5, 130.4, 123.7, 120.8, 112.1, 83.2, 68.1, 63.9, 42.2, 37.3, 32.2, 27.4, 25.3, 24.9, 21.5, 15.9;

^{11}B NMR (160 MHz, $CDCl_3$) δ 29.9;

HRMS (ESI) calcd. for $C_{26}H_{41}BNaO_5$ $[M+Na]^+$ m/z 467.2939, found 467.2926;

IR (neat, cm^{-1}) 2977, 1726, 1639, 1363, 1142.

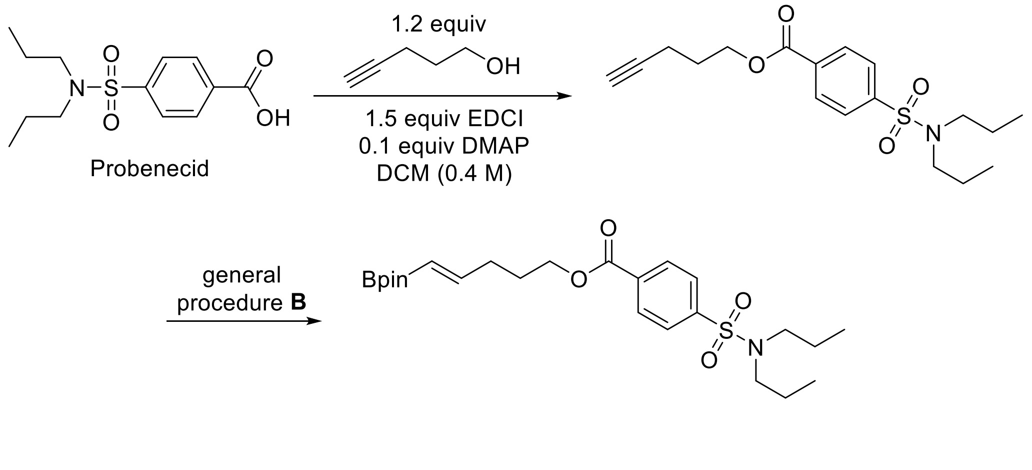

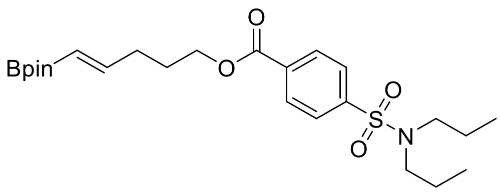

(*E*)-5-(4,4,5,5-Tetramethyl-1,3,2-dioxaborolan-2-yl)pent-4-en-1-yl 4-(*N*,*N*-dipropylsulfamoyl)benzoate (Figure 3, **1m**). The probenecid (2.85 g, 10 mmol, 1.0 equiv, CAS 57-66-9) was added to a solution of 1-(3-dimethylaminopropyl)-3-ethylcarbodiimide (EDCI, 2.88 g, 15 mmol, 1.5 equiv) and 4-dimethylaminopyridine (DMAP, 0.12 g, 1.0 mmol, 0.10 equiv) in CH_2Cl_2 (25 mL) at 0 °C. Pent-4-yn-1-ol (1.01 g, 12 mmol, 1.2 equiv) was then added. The reaction mixture was allowed to warm to rt overnight. The solution was diluted with CH_2Cl_2 and washed with 1 M HCl (aq.), saturated $NaHCO_3$ (aq.) and brine sequentially. The organic layer was dried over anhydrous Na_2SO_4. After removal of solvent under reduced pressure, the crude material was purified by flash column chromatography to provide the corresponding alkyne as a yellow oil in 84% yield (2.95 g).

From the resulting alkyne (2.81 g, 8.0 mmol), the title compound was prepared following the general procedure **B**. The crude material was purified by flash column chromatography (petroleum ether/EtOAc = 10:1) to provide the title compound as a white solid in 55% yield (2.10 g).

^{1}H NMR (500 MHz, $CDCl_3$) δ 8.14 (d, *J* = 8.6 Hz, 2H), 7.87 (d, *J* = 8.6 Hz, 2H), 6.65 (dt, *J* = 18.0, 6.4 Hz, 1H), 5.50 (dt, *J* = 17.9, 1.5 Hz, 1H), 4.36 (t, *J* = 6.5 Hz, 2H), 3.12 – 3.06 (m, 4H), 2.37 – 2.28 (m, 2H), 1.97 – 1.87 (m, 2H), 1.60 – 1.48 (m, 4H), 1.26 (s, 12H), 0.87 (t, *J* = 7.4 Hz, 6H);

^{13}C NMR (126 MHz, $CDCl_3$) δ 165.4, 152.5, 144.3, 133.8, 130.3, 127.1, 83.3, 65.2, 50.1, 32.2, 27.3, 24.9, 22.1, 11.3;

^{11}B NMR (160 MHz, $CDCl_3$) δ 30.3;

HRMS (ESI) calcd. for $C_{24}H_{38}BNNaO_6S$ $[M+Na]^+$ m/z 502.2405, found 502.2392;

IR (neat, cm^{-1}) 2975, 1720, 1643, 1275, 1142, 601;

m.p. 63 – 65 °C.

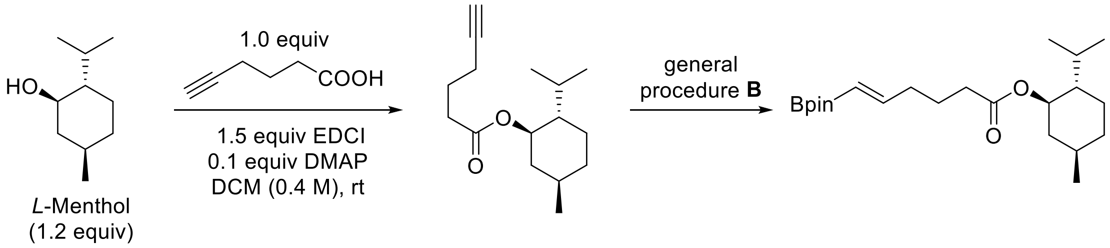

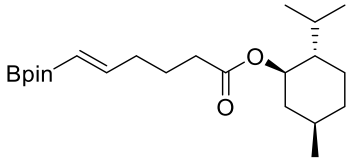

(1*R*,2*S*,5*R*)-2-Isopropyl-5-methylcyclohexyl (*E*)-6-(4,4,5,5-tetramethyl-1,3,2-dioxaborolan-2-yl)hex-5-enoate (Figure 3, **1n**). The hex-5-ynoic acid (1.12 g, 10 mmol, 1.0 equiv) was added to a solution of 1-(3-dimethylaminopropyl)-3-ethylcarbodiimide (EDCI, 2.88 g, 15 mmol, 1.5 equiv) and 4-dimethylaminopyridine (DMAP, 0.12 g, 1.0 mmol, 0.10 equiv) in CH_2Cl_2 (25 mL) at 0 °C. *L*-Menthol (1.88 g, 12 mmol, 1.2 equiv, CAS 2216-51-5) was then added. The reaction mixture was allowed to warm to rt overnight. The solution was diluted with CH_2Cl_2 and washed with 1 M HCl (aq.), saturated $NaHCO_3$ (aq.) and brine sequentially. The organic layer was dried over anhydrous Na_2SO_4. After removal of solvent under reduced pressure, the crude material was purified by flash column chromatography to provide the corresponding alkyne as a colorless oil in 80% yield (2.01 g).

From the resulting alkyne (1.88 g, 7.5 mmol), the title compound was prepared following the general procedure **B**. The crude material was purified by flash column chromatography (petroleum ether/EtOAc = 20:1) to provide the title compound as a colorless oil in 62% yield (1.76 g).

^{1}H NMR (500 MHz, $CDCl_3$) δ 6.59 (dt, *J* = 17.9, 6.4 Hz, 1H), 5.45 (dt, *J* = 17.9, 1.5 Hz, 1H), 4.67 (td, *J* = 10.9, 4.4 Hz, 1H), 2.32 – 2.24 (m, 2H), 2.22 – 2.14 (m, 2H), 2.00 – 1.93 (m, 1H), 1.90 – 1.80 (m, 1H), 1.79 – 1.71 (m, 2H), 1.71 – 1.63 (m, 2H), 1.54 – 1.42 (m, 1H), 1.39 – 1.31 (m, 1H), 1.26 (s, 12H), 1.10 – 0.99 (m, 1H), 0.99 – 0.90 (m, 1H), 0.91 – 0.81 (m, 7H), 0.75 (d, *J* = 7.0 Hz, 3H);

^{13}C NMR (126 MHz, $CDCl_3$) δ 173.2, 153.2, 83.2, 74.1, 47.2, 41.1, 35.2, 34.4, 34.3, 31.5, 26.4, 24.9, 23.8, 23.6, 22.2, 20.9, 16.4;

^{11}B NMR (160 MHz, $CDCl_3$) δ 29.9;

HRMS (ESI) calcd. for $C_{22}H_{39}BNaO_4$ [M+Na]$^+$ m/z 401.2834, found 401.2823;

IR (neat, cm^{-1}) 2955, 2929, 1728, 1639, 1362, 1143.

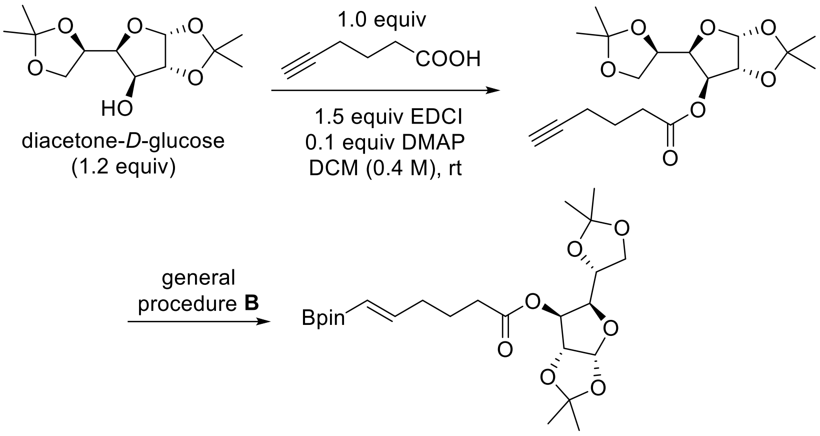

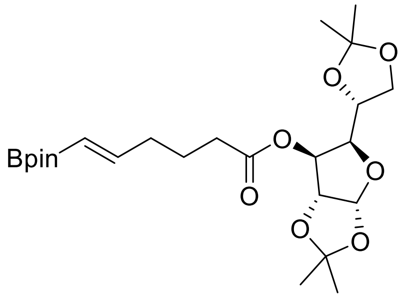

(3a*R*,5*R*,6*S*,6a*R*)-5-((*R*)-2,2-dimethyl-1,3-dioxolan-4-yl)-2,2-dimethyltetrahydro furo[2,3-*d*][1,3]dioxol-6-yl (*E*)-6-(4,4,5,5-tetramethyl-1,3,2-dioxaborolan-2-yl)hex-5-enoate (Figure 3, **1o**). The hex-5-ynoic acid (1.12 g, 10 mmol, 1.0 equiv) was added to a solution of 1-(3-dimethylaminopropyl)-3-ethylcarbodiimide (EDCI, 2.88 g, 15 mmol, 1.5 equiv) and 4-dimethylaminopyridine (DMAP, 0.12 g, 1.0 mmol, 0.10 equiv) in CH_2Cl_2 (25 mL) at 0 °C. Diacetone-*D*-glucose (3.12 g, 12 mmol, 1.2 equiv, CAS 582-52-5) was then added. The reaction mixture was allowed to warm to rt overnight. The solution was diluted with CH_2Cl_2 and washed with 1 M HCl (aq.), saturated $NaHCO_3$ (aq.) and brine sequentially. The organic layer was dried over anhydrous Na_2SO_4. After removal of solvent under reduced pressure, the crude material was purified by flash column chromatography to provide the corresponding alkyne as a colorless oil in 86% yield (3.05 g).

From the resulting alkyne (3.01 g, 8.5 mmol), the title compound was prepared following the general procedure **B**. The crude material was purified by flash column

chromatography (petroleum ether/EtOAc = 5:1) to provide the title compound as a colorless oil in 57% yield (2.33 g).

^{1}H NMR (500 MHz, $CDCl_3$) δ 6.57 (dt, *J* = 18.0, 6.4 Hz, 1H), 5.86 (d, *J* = 3.7 Hz, 1H), 5.45 (dt, *J* = 17.9, 1.5 Hz, 1H), 5.26 (d, *J* = 1.9 Hz, 1H), 4.46 (d, *J* = 3.7 Hz, 1H), 4.23 – 4.16 (m, 2H), 4.11 – 4.05 (m, 1H), 4.03 – 3.98 (m, 1H), 2.41 – 2.30 (m, 2H), 2.24 – 2.15 (m, 2H), 1.82 – 1.73 (m, 2H), 1.51 (s, 3H), 1.40 (s, 3H), 1.32 – 1.29 (m, 6H), 1.26 (s, 12H);

^{13}C NMR (126 MHz, $CDCl_3$) δ 172.1, 152.8, 112.4, 109.5, 105.2, 83.5, 83.3, 80.0, 76.1, 72.6, 67.5, 34.9, 33.7, 27.0, 26.9, 26.4, 25.4, 24.9, 23.4;

^{11}B NMR (160 MHz, $CDCl_3$) δ 30.0;

HRMS (ESI) calcd. for $C_{24}H_{39}BNaO_9$ $[M+Na]^+$ m/z 505.2579, found 505.2577;

IR (neat, cm^{-1}) 2981, 2936, 1745, 1638, 1363, 1142, 1073.

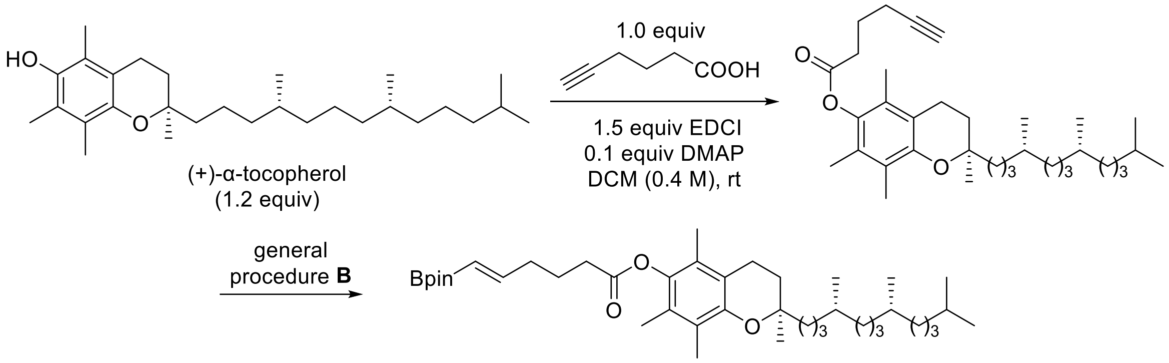

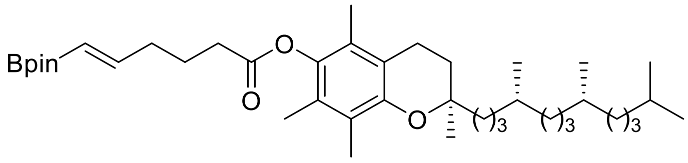

(*R*)-2,5,7,8-tetramethyl-2-((4*R*,8*R*)-4,8,12-trimethyltridecyl)chroman-6-yl (*E*)-6-(4,4,5,5-tetramethyl-1,3,2-dioxaborolan-2-yl)hex-5-enoate (Figure 3, **1p**). The hex-5-ynoic acid (1.12 g, 10 mmol, 1.0 equiv) was added to a solution of 1-(3-dimethylaminopropyl)-3-ethylcarbodiimide (EDCI, 2.88 g, 15 mmol, 1.5 equiv) and 4-dimethylaminopyridine (DMAP, 0.12 g, 1.0 mmol, 0.10 equiv) in CH_2Cl_2 (25 mL) at 0 °C. (+)-α-Tocopherol (5.16 g, 12 mmol, 1.2 equiv, CAS 59-02-9) was then added. The reaction mixture was allowed to warm to rt overnight. The solution was diluted with CH_2Cl_2 and washed with 1 M HCl (aq.), saturated $NaHCO_3$ (aq.) and brine

sequentially. The organic layer was dried over anhydrous Na_2SO_4. After removal of solvent under reduced pressure, the crude material was purified by flash column chromatography to provide the corresponding alkyne as a colorless oil in 87% yield (4.57 g).

From the resulting alkyne (4.46 g, 8.5 mmol), the title compound was prepared following the general procedure **B**. The crude material was purified by flash column chromatography (petroleum ether/EtOAc = 100:1) to provide the title compound as a colorless oil in 68% yield (3.78 g).

^{1}H NMR (500 MHz, $CDCl_3$) δ 6.64 (dt, J = 17.9, 6.5 Hz, 1H), 5.50 (dt, J = 17.9, 1.4 Hz, 1H), 2.64 – 2.55 (m, 4H), 2.35 – 2.27 (m, 2H), 2.08 (s, 3H), 2.00 (s, 3H), 1.98 – 1.89 (m, 5H), 1.85 – 1.70 (m, 2H), 1.58 – 1.48 (m, 3H), 1.46 – 1.32 (m, 4H), 1.33 – 1.21 (s, 23H), 1.17 – 1.02 (m, 6H), 0.90 – 0.82 (m, 12H);

^{13}C NMR (126 MHz, $CDCl_3$) δ 172.2, 153.0, 149.5, 140.6, 126.8, 125.0, 123.1, 117.5, 83.3, 75.2, 39.5, 37.6, 37.6, 37.4, 35.3, 33.7, 33.0, 32.9, 31.3, 28.1, 25.0, 24.9, 24.6, 23.9, 22.9, 22.8, 21.2, 20.8, 19.9, 19.8, 13.1, 12.3, 12.0;

^{11}B NMR (160 MHz, $CDCl_3$) δ 29.9;

HRMS (ESI) calcd. for $C_{41}H_{69}BNaO_5$ $[M+Na]^+$ m/z 675.5130, found 675.5128;

IR (neat, cm^{-1}) 2926, 2868, 1753, 1638, 1362, 1133.

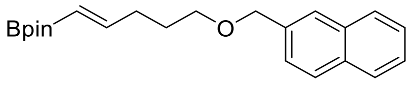

(*E*)-4,4,5,5-Tetramethyl-2-(5-(naphthalen-2-ylmethoxy)pent-1-en-1-yl)-1,3,2-dioxaborolane (Figure 4, **1t**). From 2-((pent-4-yn-1-yloxy)methyl)naphthalene (1.35 g, 6.0 mmol), the title compound was prepared following the general procedure **B**. The crude material was purified by flash column chromatography (petroleum ether/EtOAc = 20:1) to provide the title compound as a colorless oil in 64% yield (1.35 g).

^{1}H NMR (500 MHz, $CDCl_3$) δ 7.83 (d, J = 9.1 Hz, 3H), 7.77 (s, 1H), 7.49 – 7.43 (m, 3H), 6.65 (dt, J = 17.9, 6.4 Hz, 1H), 5.47 (dt, J = 17.9, 1.5 Hz, 1H), 4.66 (s, 2H), 3.53 (t, J = 6.5 Hz, 2H), 2.32 – 2.23 (m, 2H), 1.82 – 1.74 (m, 2H), 1.26 (s, 12H);

^{13}C NMR (126 MHz, $CDCl_3$) δ 153.9, 136.2, 133.4, 133.1, 128.3, 128.0, 127.8, 126.4,

126.2, 125.9, 83.2, 73.1, 69.9, 32.5, 28.4, 24.9;

^{11}B NMR (160 MHz, $CDCl_3$) δ 30.0;

HRMS (ESI) calcd. for $C_{22}H_{29}BNaO_3$ $[M+Na]^+$ m/z 375.2102, found 375.2093;

IR (neat, cm^{-1}) 2978, 2935, 2856, 1639, 1362, 1143.

b. Preparation of 1,4,2-dioxazol-5-ones

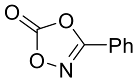
2a

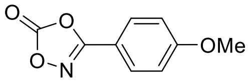
2b

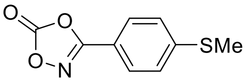
2c

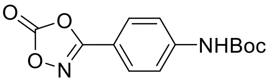
2d

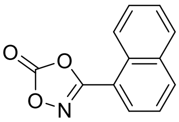
2e

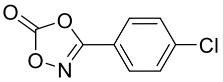
2f

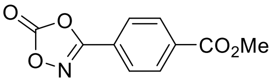
2g

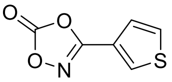
2h

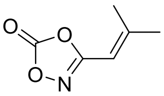
2i

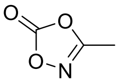
2j

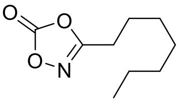
2k

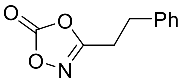
2l

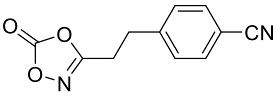
2m

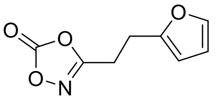
2n

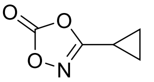
2o

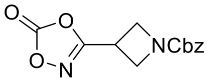
2p

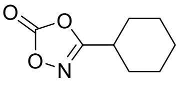
2q

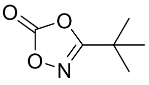
2r

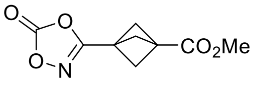
2s

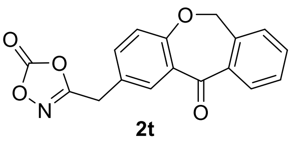
2t

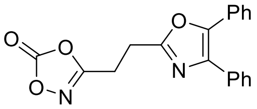
2u

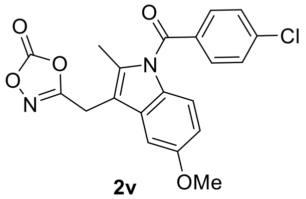
2v

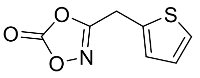
2w

Compounds **2a**, **2b**, **2f**, **2k**, **2l**, **2s**, **2t** and **2v** were prepared according to reference 12. Compounds **2g**, **2j**, **2o**, **2q** and **2r** were prepared according to reference 13.

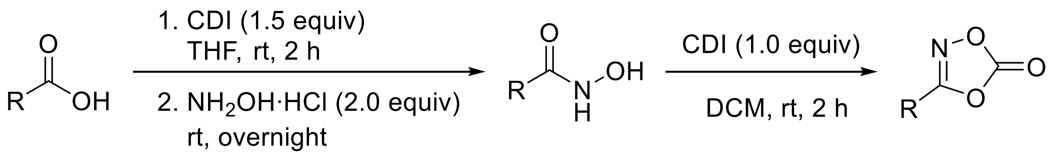

General procedure C for the synthesis of 1,4,2-dioxazol-5-ones[12-14]. 1,1'-Carbonyldiimidazole (CDI, 1.5 equiv) was added to a mixture of carboxylic acid (1.0 equiv.) in dry tetrahydrofuran (THF, 1.0 M) at rt. The reaction mixture was stirred for 2 hours. Afterward, hydroxylamine hydrochloride (2.0 equiv) was added. The resulting mixture was stirred overnight. The reaction mixture was diluted with 5% $KHSO_4$ (aq) and extracted with EtOAc. The combined organic layer was washed with water and

brine, dried over anhydrous Na_2SO_4, and concentrated in vacuo. The crude hydroxamic acid was used for next step without further purification.

To a stirred solution of hydoxamic acid (1.0 equiv) in freshly distilled dichloromethane, 1,1′-carbonyldiimidazole (1.0 equiv) was added in one portion at rt. After being stirred for 2 hours, the reaction mixture was quenched with 1 N HCl (aq.), and extracted with EtOAc. The combined organic phase was dried over anhydrous Na_2SO_4, filtered and concentrated in vacuo. The resulting residue was purified quickly by short silica pad (PE/EA = 10:1 ~ 5:1) to give the desired 1,4,2-dioxazol-5-ones.

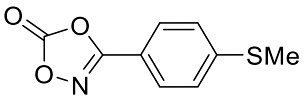

3-(4-(Methylthio)phenyl)-1,4,2-dioxazol-5-one (Figure 4, **2c**). From 4-(methylthio)benzoic acid (1.68 g, 10 mmol), the title compound was prepared following the general procedure **C**. The crude material was purified quickly by short silica pad (petroleum ether/EtOAc = 10:1) to provide the title compound as a white solid in 58% yield (1.21 g).

^{1}H NMR (400 MHz, $CDCl_3$) δ 7.73 (d, *J* = 8.8 Hz, 2H), 7.33 (d, *J* = 8.7 Hz, 2H), 2.54 (s, 3H);

^{13}C NMR (101 MHz, $CDCl_3$) δ 163.5, 154.0, 147.4, 126.8, 125.8, 115.9, 14.9;

HRMS (ESI) calcd. for C_8H_7NNaOS $[M–CO_2+Na]^+$ m/z 116.0321, found 116.0317;

IR (neat, cm^{-1}) 1824, 1605, 1360, 1097, 996, 750;

m.p. 115 – 116 °C.

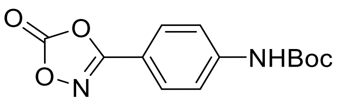

***tert*-Butyl (4-(5-oxo-1,4,2-dioxazol-3-yl)phenyl)carbamate** (Figure 4, **2d**). From 4-((*tert*-butoxycarbonyl)amino)benzoic acid (2.37 g, 10 mmol), the title compound was prepared following the general procedure **C**. The crude material was purified quickly by short silica pad (petroleum ether/EtOAc = 8:1) to provide the title compound as a

white solid in 47% yield (1.31 g).

^{1}H NMR (400 MHz, $CDCl_3$) δ 7.77 (d, *J* = 8.9 Hz, 2H), 7.55 (d, *J* = 8.9 Hz, 2H), 6.77 (s, 1H), 1.53 (s, 9H);

^{13}C NMR (101 MHz, $CDCl_3$) δ 163.4, 154.1, 152.1, 143.7, 128.0, 118.4, 114.0, 81.9, 28.4;

HRMS (ESI) calcd. for $C_{12}H_{14}N_2NaO_3$ $[M–CO_2+Na]^+$ m/z 257.0897, found 257.0893;

IR (neat, cm^{-1}) 3355, 1860, 1698, 1613, 1502, 1155, 757;

m.p. 159 – 160 °C.

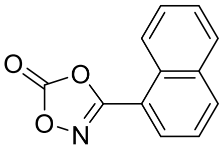

3-(naphthalen-1-yl)-1,4,2-dioxazol-5-one (Figure 4, **2e**). From 1-naphthoic acid (3.44 g, 20 mmol), the title compound was prepared following the general procedure **C**. The crude material was purified quickly by short silica pad (petroleum ether/EtOAc = 8:1) to provide the title compound as a white solid in 27% yield (1.15 g).

^{1}H NMR (500 MHz, $CDCl_3$) δ 8.79 – 8.71 (m, 1H), 8.13 (d, *J* = 8.3 Hz, 1H), 8.06 (dd, *J* = 7.3, 1.2 Hz, 1H), 7.97 (d, *J* = 8.2 Hz, 1H), 7.74 – 7.68 (m, 1H), 7.67 – 7.56 (m, 2H);

^{13}C NMR (126 MHz, $CDCl_3$) δ 164.1, 153.6, 134.9, 133.9, 129.6, 129.5, 129.3, 129.1, 127.4, 125.5, 124.9, 116.7;

HRMS (ESI) calcd. for $C_{11}H_8NO$ $[M–CO_2+H]^+$ m/z 170.0601, found 170.0596;

IR (neat, cm^{-1}) 1809, 1321, 1002, 759;

m.p. 53 – 54 °C.

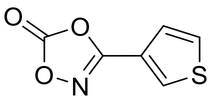

3-(Thiophen-3-yl)-1,4,2-dioxazol-5-one (Figure 4, **2h**). From thiophene-3-carboxylic acid (1.28 g, 10 mmol), the title compound was prepared following the general procedure **C**. The crude material was purified quickly by short silica pad (petroleum ether/EtOAc = 8:1) to provide the title compound as a white solid in 72% yield (1.22

g). Spectral data match those previously reported[15].

^{1}H NMR (400 MHz, $CDCl_3$) δ 8.07 – 7.99 (m, 1H), 7.56 – 7.45 (m, 2H);

^{13}C NMR (101 MHz, $CDCl_3$) δ 160.5, 153.7, 130.6, 128.7, 124.8, 121.1.

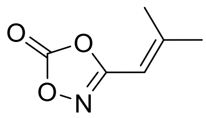

3-(2-Methylprop-1-en-1-yl)-1,4,2-dioxazol-5-one (Figure 4, **2i**). From 3-methylbut-2-enoic acid (1.00 g, 10 mmol), the title compound was prepared following the general procedure **C**. The crude material was purified quickly by short silica pad (petroleum ether/EtOAc = 10:1) to provide the title compound as a yellow liquid in 55% yield (0.78 g).

^{1}H NMR (500 MHz, $CDCl_3$) δ 5.83 – 5.74 (m, 1H), 2.13 (d, *J* = 0.9 Hz, 3H), 2.04 (d, *J* = 1.3 Hz, 3H);

^{13}C NMR (126 MHz, $CDCl_3$) δ 163.0, 156.6, 154.0, 104.8, 27.8, 21.8;

HRMS (ESI) calcd. for C_5H_8NO $[M–CO_2+H]^+$ m/z 98.0601, found 98.0602;

IR (neat, cm^{-1}) 1830, 1656, 1284, 1154, 984, 761.

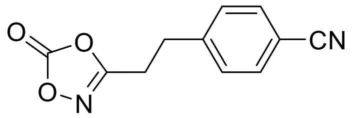

4-(2-(5-Oxo-1,4,2-dioxazol-3-yl)ethyl)benzonitrile (Figure 4, **2m**). From 3-(4-cyanophenyl)propanoic acid (1.75 g, 10 mmol), the title compound was prepared following the general procedure **C**. The crude material was purified quickly by short silica pad (petroleum ether/EtOAc = 5:1) to provide the title compound as a white solid in 79% yield (1.71 g).

^{1}H NMR (400 MHz, $CDCl_3$) δ 7.64 (d, *J* = 8.3 Hz, 2H), 7.34 (d, *J* = 8.3 Hz, 2H), 3.12 (t, *J* = 7.6 Hz, 2H), 3.02 – 2.93 (m, 2H);

^{13}C NMR (101 MHz, $CDCl_3$) δ 165.3, 153.8, 143.4, 132.9, 129.2, 118.6, 111.5, 30.4, 26.2;

HRMS (ESI) calcd. for $C_{10}H_8N_2NaO$ $[M–CO_2+Na]^+$ m/z 195.0529, found 195.0524;

IR (neat, cm^{-1}) 2231, 1826, 1637, 1148, 989, 756, 558;

m.p. 82 – 83 °C.

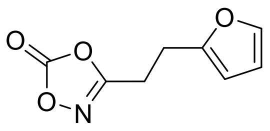

3-(2-(Furan-2-yl)ethyl)-1,4,2-dioxazol-5-one (Figure 4, **2n**). From 3-(furan-2-yl)propanoic acid (1.40 g, 10 mmol), the title compound was prepared following the general procedure **C**. The crude material was purified quickly by short silica pad (petroleum ether/EtOAc = 5:1) to provide the title compound as a white solid in 72% yield (1.30 g).

^{1}H NMR (500 MHz, $CDCl_3$) δ 7.34 (dd, *J* = 1.8, 0.6 Hz, 1H), 6.30 (dd, *J* = 3.2, 1.9 Hz, 1H), 6.15 – 6.06 (m, 1H), 3.08 (t, *J* = 7.3 Hz, 2H), 3.03 – 2.94 (m, 2H);

^{13}C NMR (126 MHz, $CDCl_3$) δ 165.7, 154.1, 151.4, 142.2, 110.6, 106.8, 24.1, 23.3;

HRMS (ESI) calcd. for $C_7H_7NNaO_2$ $[M–CO_2+Na]^+$ m/z 160.0369, found 160.0365;

IR (neat, cm^{-1}) 1819, 1641, 1152, 983, 758;

m.p. 50 – 51 °C.

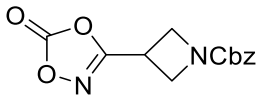

Benzyl 3-(5-oxo-1,4,2-dioxazol-3-yl)azetidine-1-carboxylate (Figure 4, **2p**). From 1-((benzyloxy)carbonyl)azetidine-3-carboxylic acid (2.35 g, 10 mmol), the title compound was prepared following the general procedure **C**. The crude material was purified quickly by short silica pad (petroleum ether/EtOAc = 3:1) to provide the title compound as a white solid in 63% yield (1.74 g).

^{1}H NMR (500 MHz, $CDCl_3$) δ 7.42 – 7.29 (m, 5H), 5.12 (s, 2H), 4.37 (t, *J* = 9.0 Hz, 2H), 4.26 (dd, *J* = 9.1, 6.0 Hz, 2H), 3.84 – 3.73 (m, 1H);

^{13}C NMR (126 MHz, $CDCl_3$) δ 165.6, 156.0, 153.6, 136.1, 128.7, 128.5, 128.3, 67.4, 51.3, 24.7;

HRMS (ESI) calcd. for $C_{12}H_{12}N_2NaO_3$ $[M–CO_2+Na]^+$ m/z 255.0740, found 255.0733;

IR (neat, cm^{-1}) 1820, 1708, 1356, 1131, 990, 760, 732;

m.p. 110 – 111 °C.

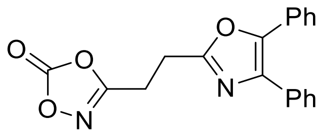

3-(2-(4,5-Diphenyloxazol-2-yl)ethyl)-1,4,2-dioxazol-5-one (Figure 4, **2u**). From 3-(4,5-diphenyloxazol-2-yl)propanoic acid (2.93 g, 10 mmol), the title compound was prepared following the general procedure **C**. The crude material was purified quickly by short silica pad (petroleum ether/EtOAc = 2:1) to provide the title compound as a white solid in 81% yield (2.71 g).

^{1}H NMR (500 MHz, $CDCl_3$) δ 7.66 – 7.59 (m, 2H), 7.59 – 7.54 (m, 2H), 7.41 – 7.31 (m, 6H), 3.35 – 3.21 (m, 4H);

^{13}C NMR (126 MHz, $CDCl_3$) δ 165.4, 159.5, 154.0, 146.2, 135.4, 132.1, 128.9, 128.9, 128.8, 128.7, 128.4, 128.0, 126.7, 23.1, 22.5;

HRMS (ESI) calcd. for $C_{18}H_{14}N_2NaO_2$ $[M–CO_2+Na]^+$ m/z 313.0948, found 313.0941;

IR (neat, cm^{-1}) 1869, 1829, 1157, 991, 757, 694;

m.p. 76 – 77 °C.

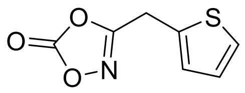

3-(Thiophen-2-ylmethyl)-1,4,2-dioxazol-5-one (Figure 5, **2w**). From 2-(thiophen-2-yl)acetic acid (1.42 g, 10 mmol), the title compound was prepared following the general procedure **C**. The crude material was purified quickly by short silica pad (petroleum ether/EtOAc = 8:1) to provide the title compound as a orange solid in 62% yield (1.14 g).

^{1}H NMR (500 MHz, $CDCl_3$) δ 7.30 (dd, J = 5.1, 1.2 Hz, 1H), 7.08 – 6.97 (m, 2H), 4.17 (s, 2H);

^{13}C NMR (126 MHz, $CDCl_3$) δ 164.5, 153.8, 131.0, 128.4, 127.7, 126.6, 25.7;

HRMS (ESI) calcd. for C_6H_6NOS $[M–CO_2+H]^+$ m/z 140.0165, found 140.0161;

IR (neat, cm^{-1}) 1817, 1632, 1341, 1150, 988, 712;

m.p. 51 – 52 °C.

c. Preparation of L*

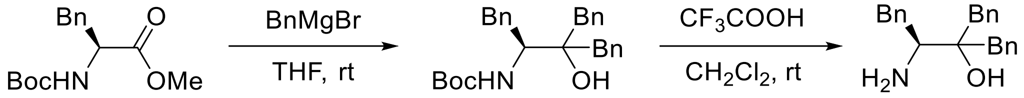

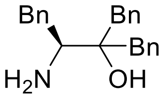

(*S*)-3-Amino-2-benzyl-1,4-diphenylbutan-2-ol (**L***).

Under N_2 atmosphere, methyl (*tert*-butoxycarbonyl)-*L*-phenylalaninate (2.23 g, 8.0 mmol, 1.0 equiv) was dissolved in THF (10 mL). The solution was cooled to 0 °C, and benzylmagnesium chloride (40 mL, 1.0 M in THF, 5.0 equiv) was added dropwise over 15 min. The resulting mixture was warmed to rt and stirred for 24 h at rt. Completion of the reaction was monitored by TLC. The solution was cooled to 0 °C again and carefully quenched with a saturated aqueous solution of NH_4Cl. The mixture was extracted with Et_2O and washed with brine, dried over Na_2SO_4, filtered, and concentrated under reduced pressure to give the crude product. The crude material was purified by flash column chromatography (petroleum ether/EtOAc = 50:1) to provide *tert*-butyl (*S*)-(3-benzyl-3-hydroxy-1,4-diphenylbutan-2-yl)carbamate as a sticky oil or white solid in 38% yield (1.31 g).

CF_3COOH (2.0 mL) was added to a solution of *tert*-butyl (*S*)-(3-benzyl-3-hydroxy-1,4-diphenylbutan-2-yl)carbamate (1.31 g, 3.0 mmol, 1.0 equiv) in CH_2Cl_2 (10 mL), and the mixture was stirred at rt. Completion of the reaction was monitored by TLC. The solution was cooled to 0 °C, and saturated aqueous $NaHCO_3$ was added carefully. The mixture was extracted with Et_2O and washed with saturated aqueous K_2CO_3, brine, dried over Na_2SO_4, filtered, and concentrated under reduced pressure to give the crude product. After recrystallization (petroleum ether/EtOAc), (*S*)-3-amino-2-benzyl-1,4-diphenylbutan-2-ol was obtained as a white solid in 85% yield (0.85 g).

^{1}H NMR (500 MHz, CD_3CN) δ 7.40 – 7.14 (m, 13H), 7.09 – 7.03 (m, 2H), 3.90 (s, 1H),

3.23 (d, J = 12.5 Hz, 1H), 2.95 (d, J = 13.3 Hz, 1H), 2.90 – 2.78 (m, 2H), 2.68 (d, J = 13.3 Hz, 1H), 2.52 – 2.38 (m, 2H), 1.04 (s, 2H);

^{13}C NMR (126 MHz, CD_3CN) δ 141.6, 139.4, 139.3, 132.2, 131.9, 130.1, 129.3, 128.8, 128.7, 127.1, 127.0, 126.9, 76.2, 59.4, 42.4, 42.3, 39.6;

HRMS (ESI) calcd. for $C_{23}H_{26}NO$ $[M+H]^+$ m/z 332.2009, found 332.2000;

IR (neat, cm^{-1}) 3377, 3317, 3026, 2937, 1493, 752, 696;

m.p. 133 – 135 °C;

$[\alpha]_D^{23}$ = +17.1 (c = 0.79, $CHCl_3$).

II. Supplementary Figures

1. NMR Spectroscopic Data

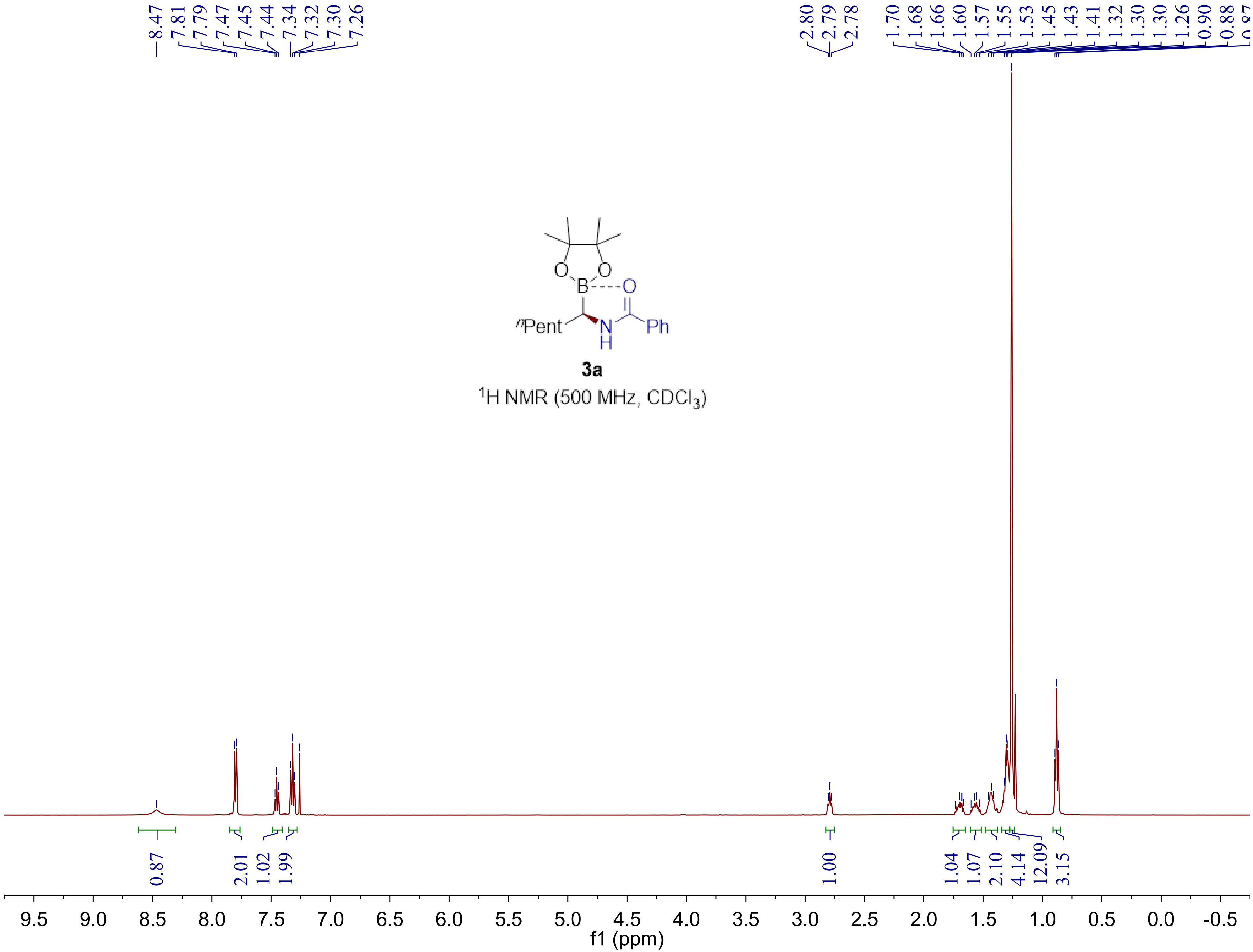

Supplementary Figure 2: ^{1}H NMR (500 MHz, $CDCl_3$) spectrum of **3a**.

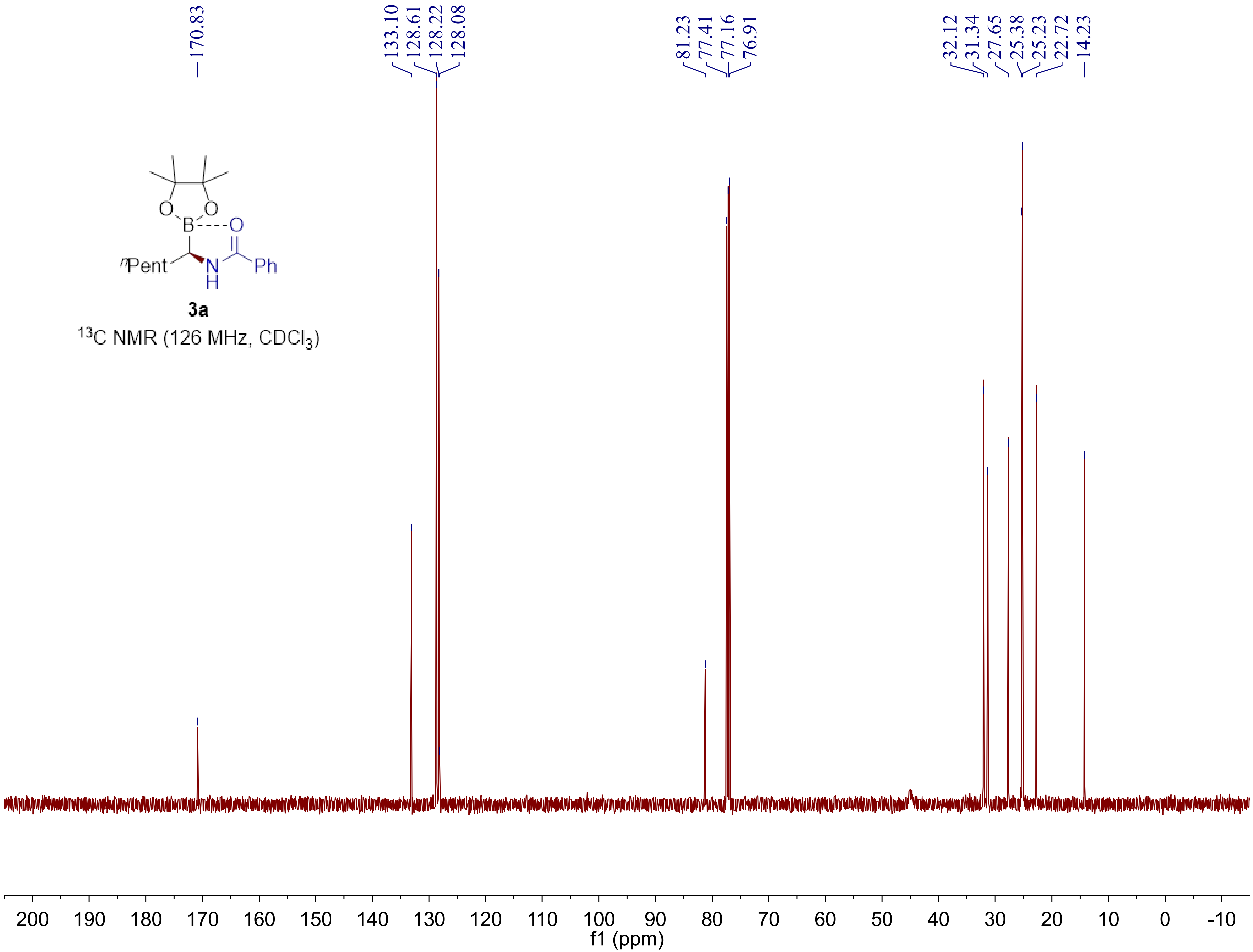

Supplementary Figure 3: ^{13}C NMR (126 MHz, $CDCl_3$) spectrum of **3a**.

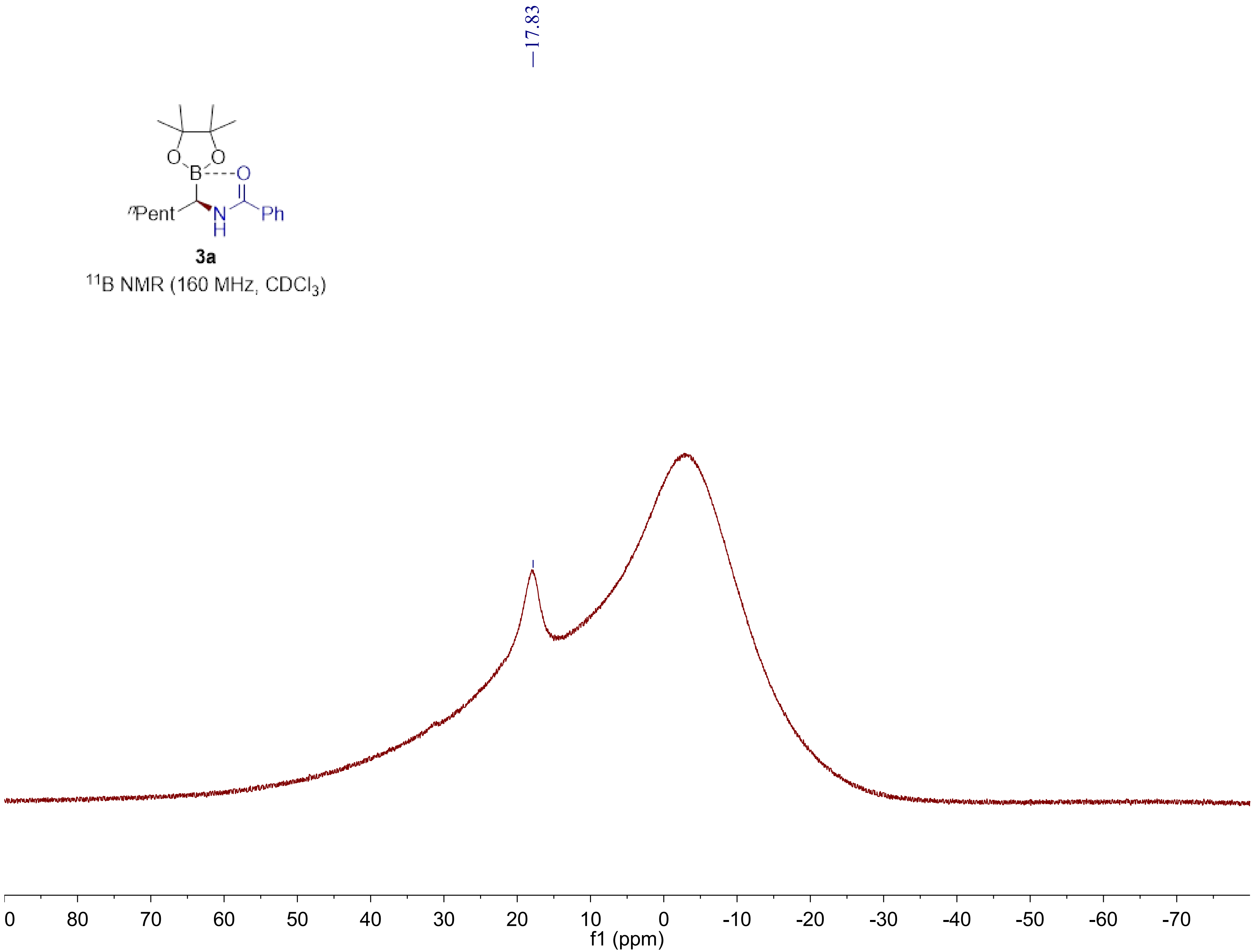

Supplementary Figure 4: ^{11}B NMR (160 MHz, $CDCl_3$) spectrum of **3a**.

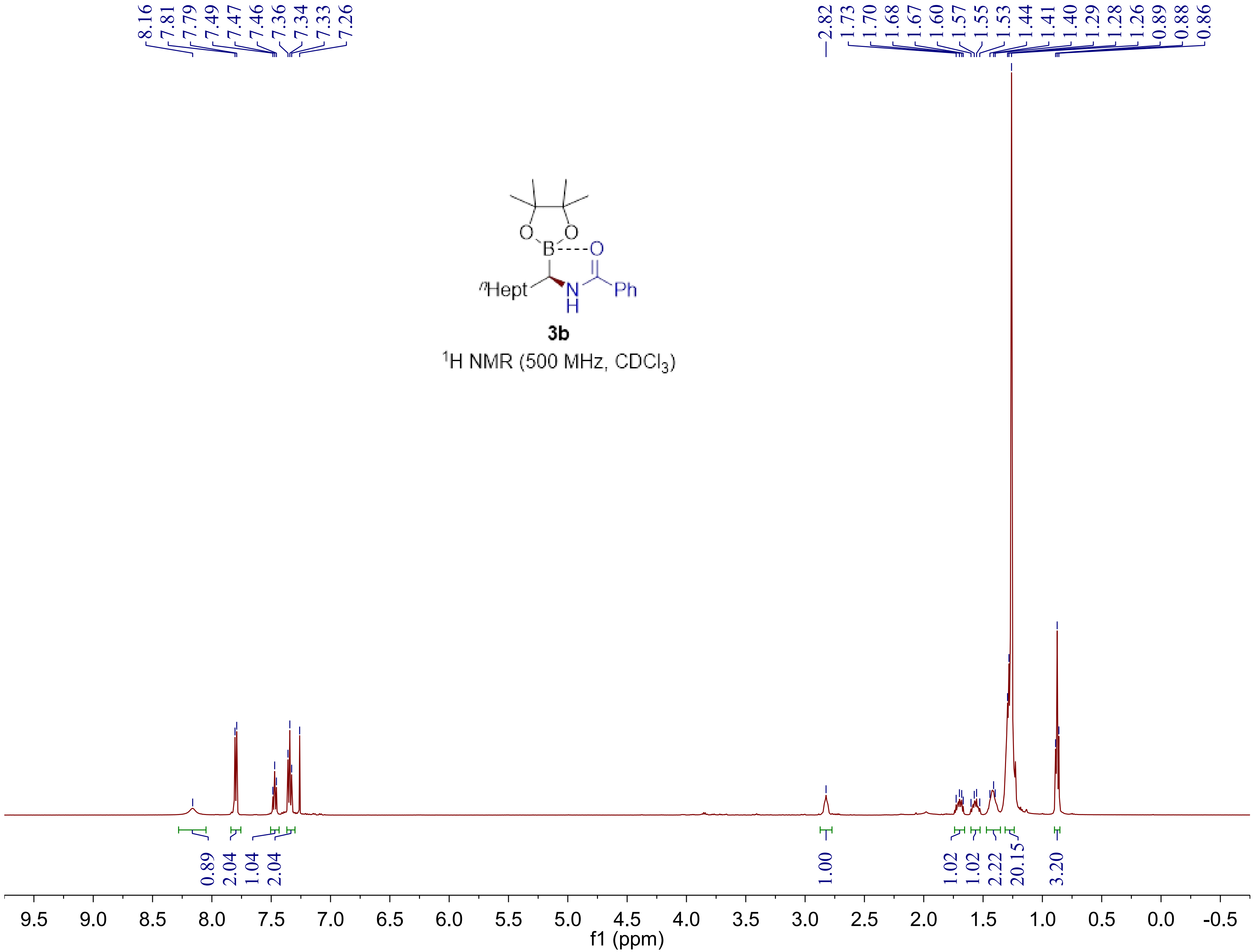

Supplementary Figure 5: ^{1}H NMR (500 MHz, $CDCl_3$) spectrum of **3b**.

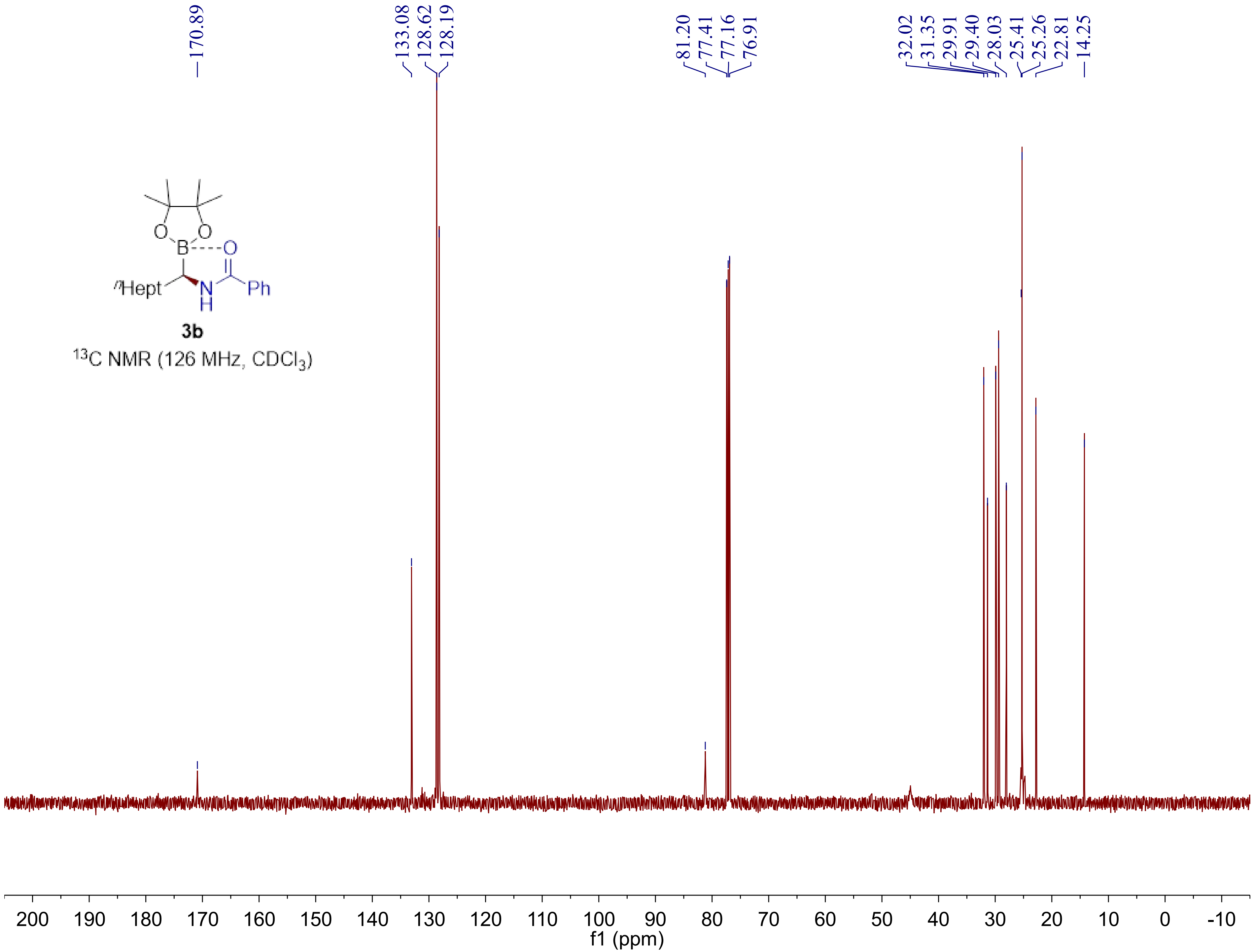

Supplementary Figure 6: ^{13}C NMR (126 MHz, $CDCl_3$) spectrum of **3b**.

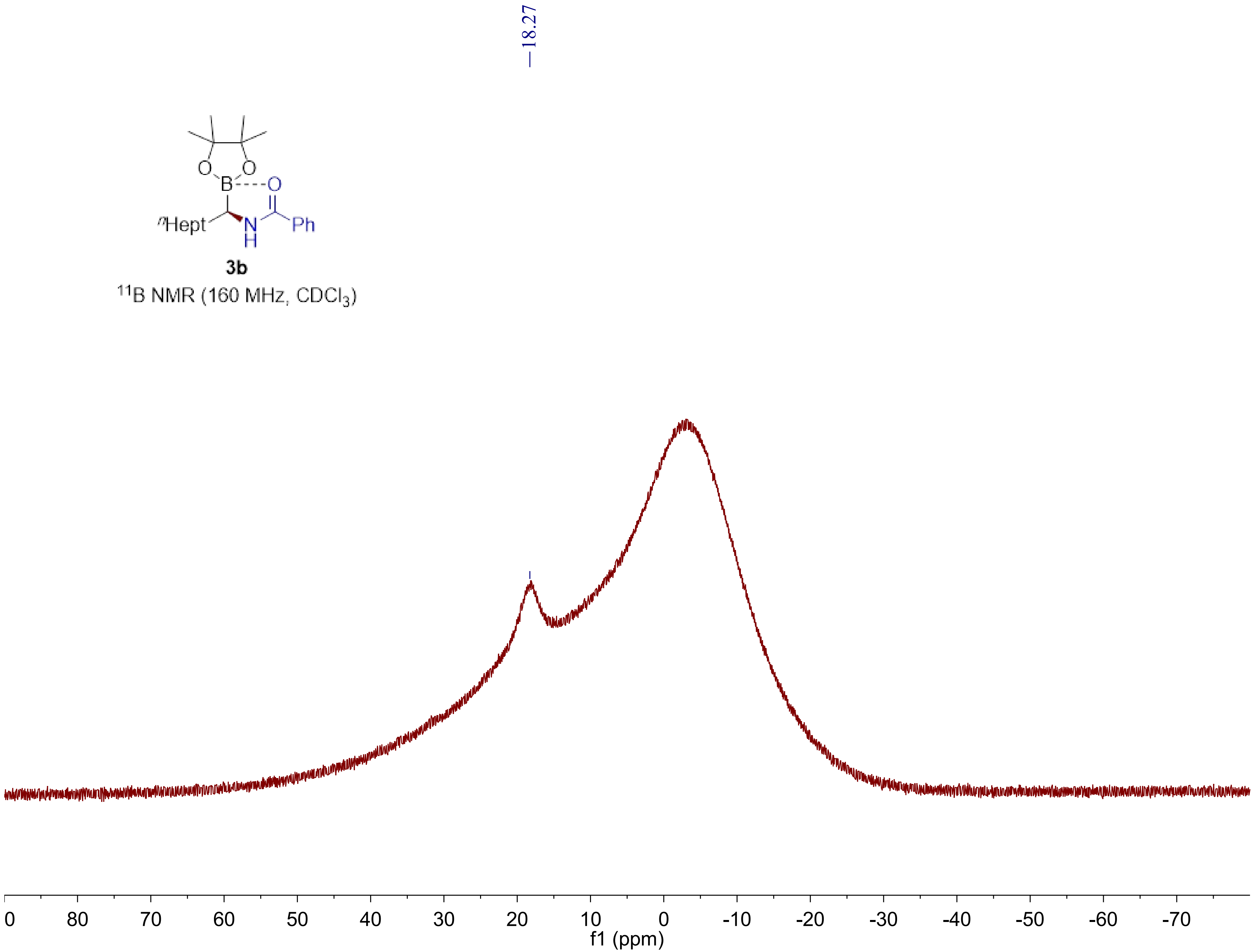

Supplementary Figure 7: ^{11}B NMR (160 MHz, $CDCl_3$) spectrum of **3b**.

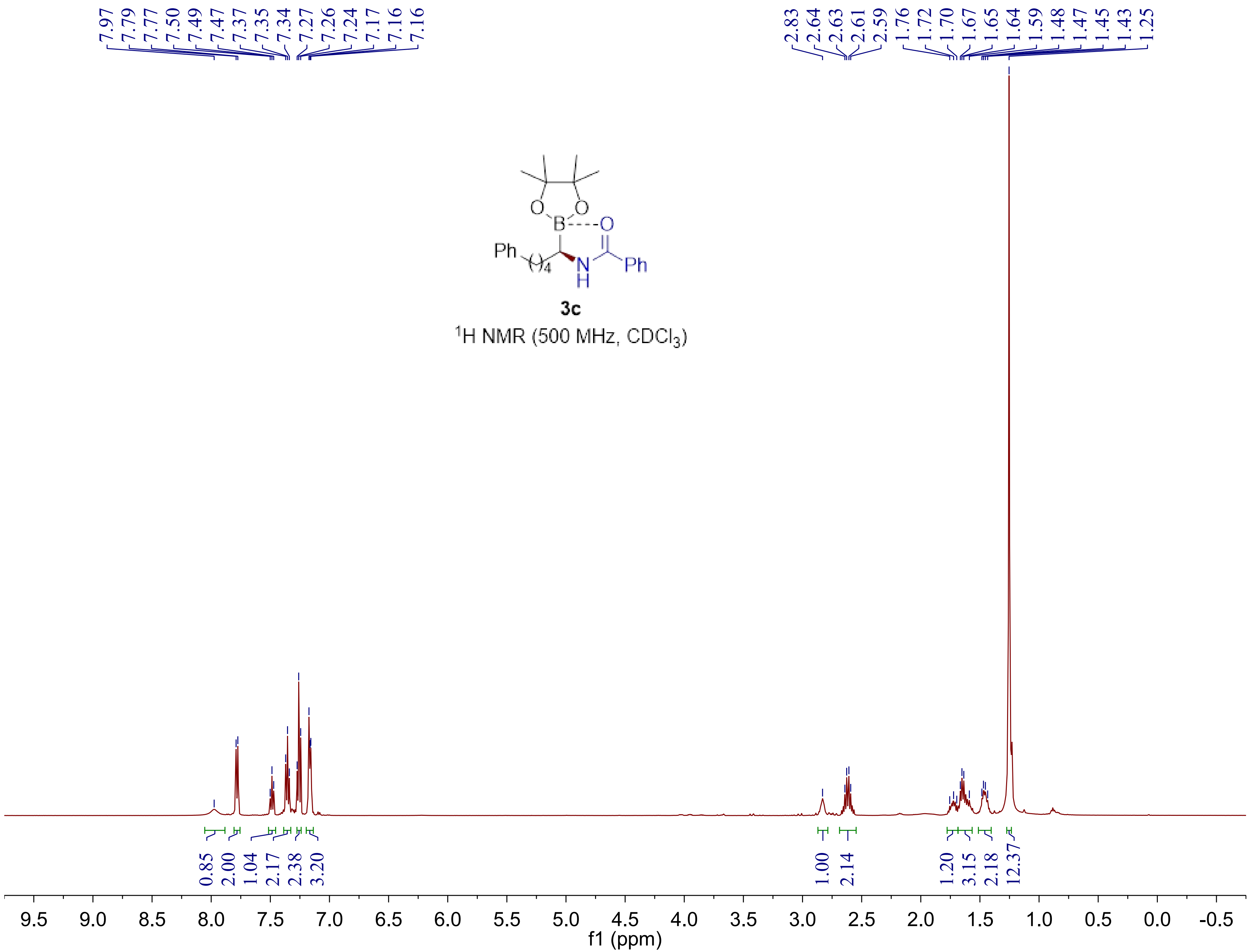

Supplementary Figure 8: ^{1}H NMR (500 MHz, CDCl$_3$) spectrum of **3c**.

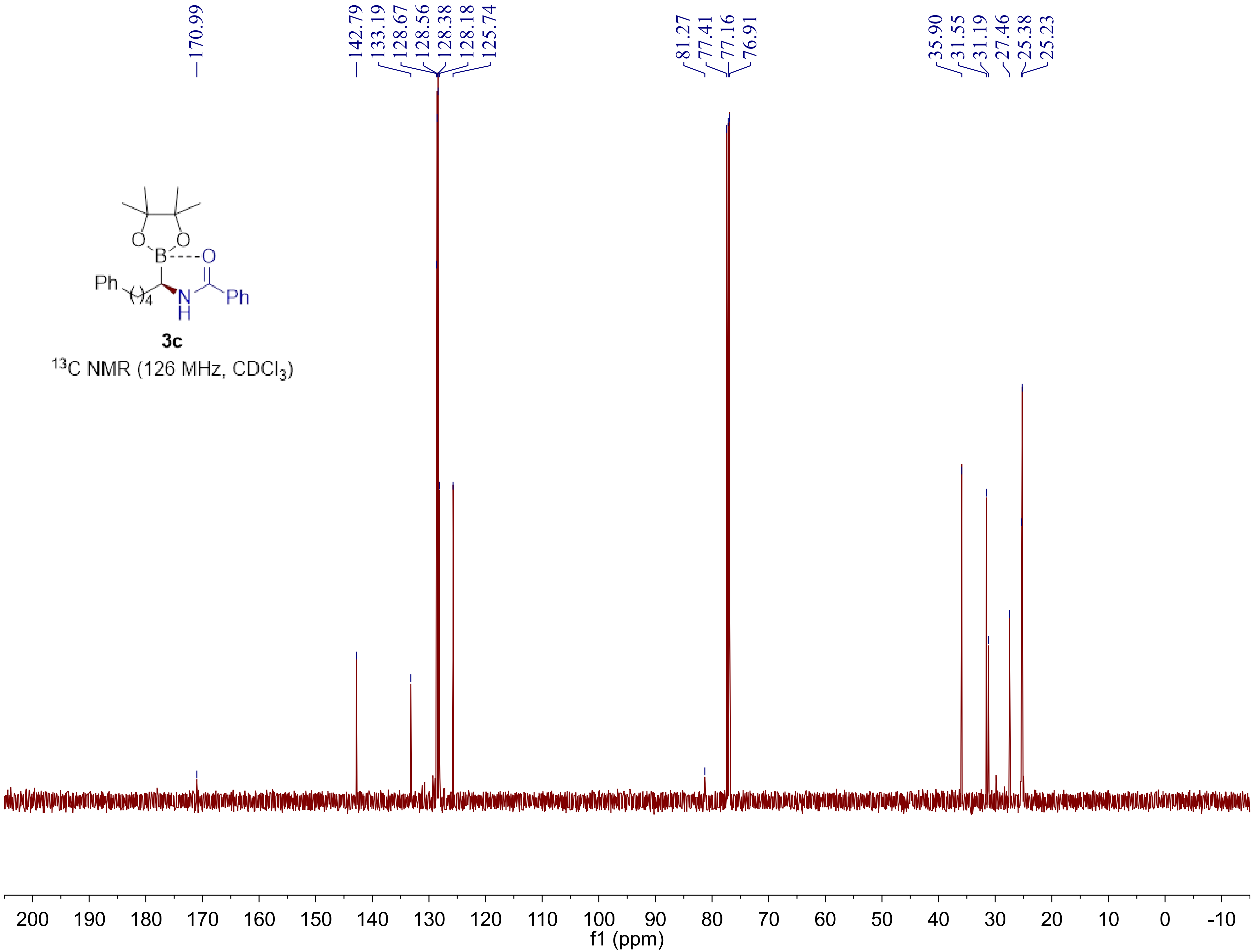

Supplementary Figure 9: ^{13}C NMR (126 MHz, $CDCl_3$) spectrum of **3c**.

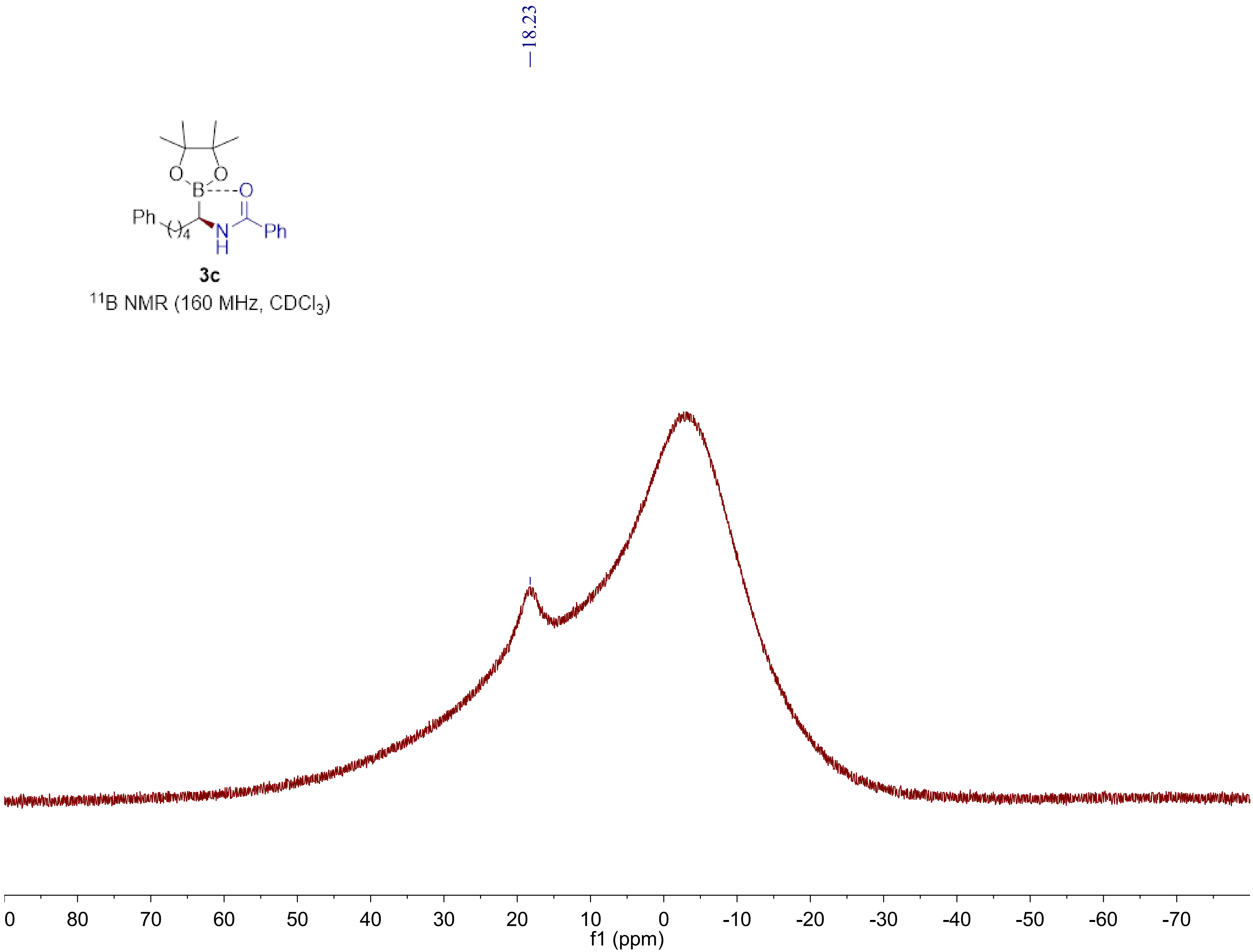

Supplementary Figure 10: ^{11}B NMR (160 MHz, $CDCl_3$) spectrum of **3c**.

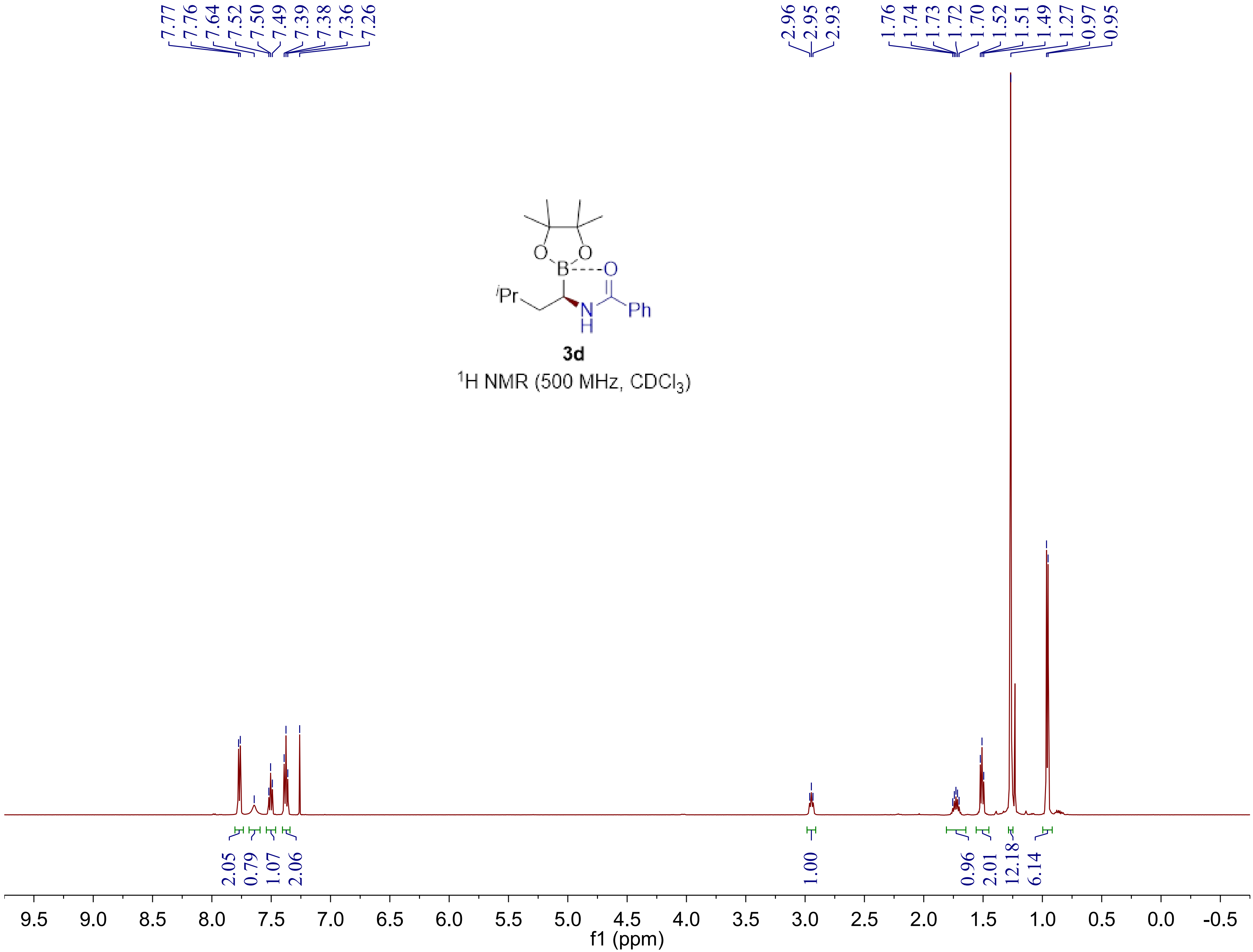

Supplementary Figure 11: ^{1}H NMR (500 MHz, $CDCl_3$) spectrum of **3d**.

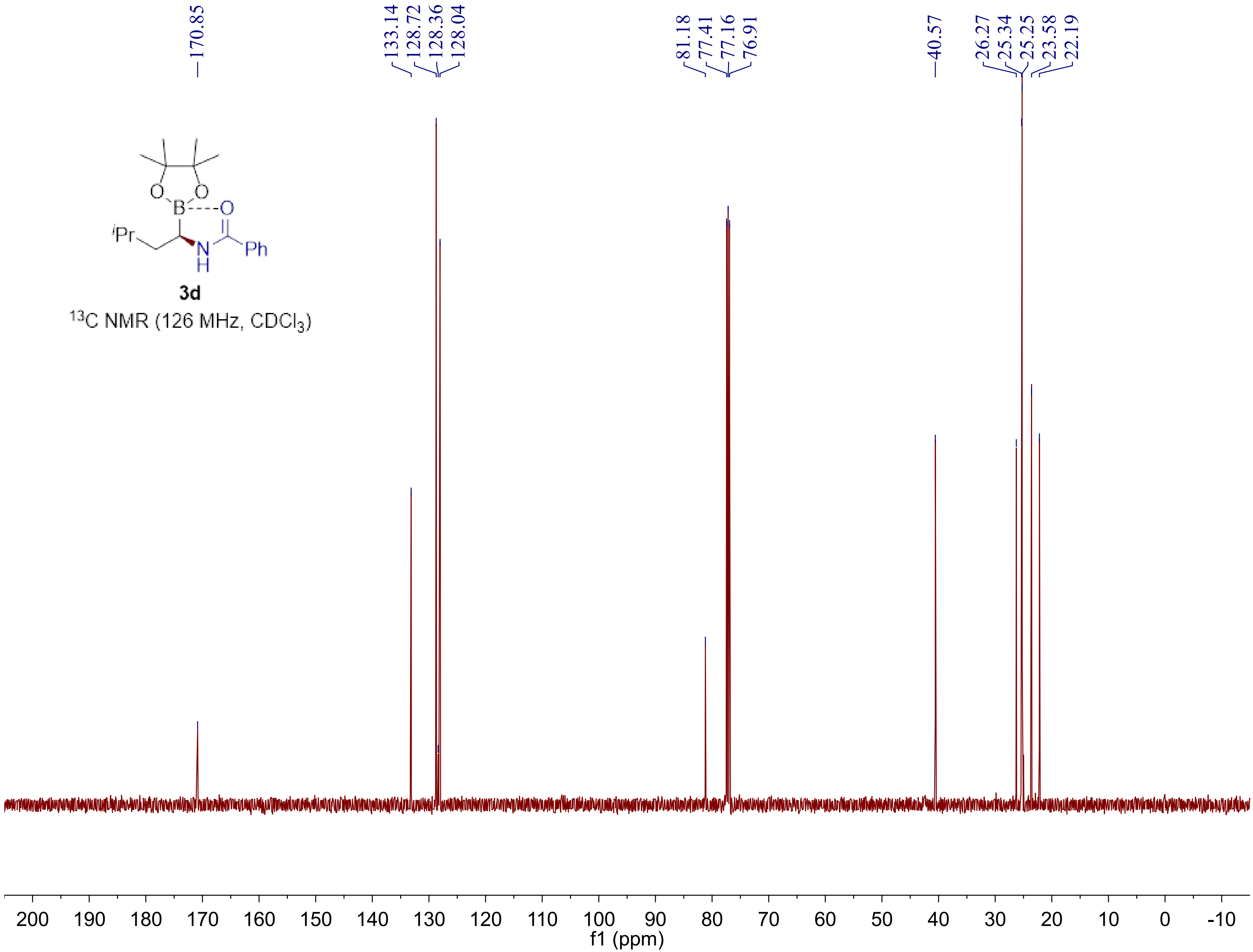

Supplementary Figure 12: ^{13}C NMR (126 MHz, $CDCl_3$) spectrum of **3d**.

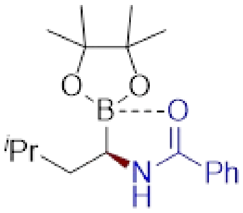

3d

^{11}B NMR (160 MHz, $CDCl_3$)

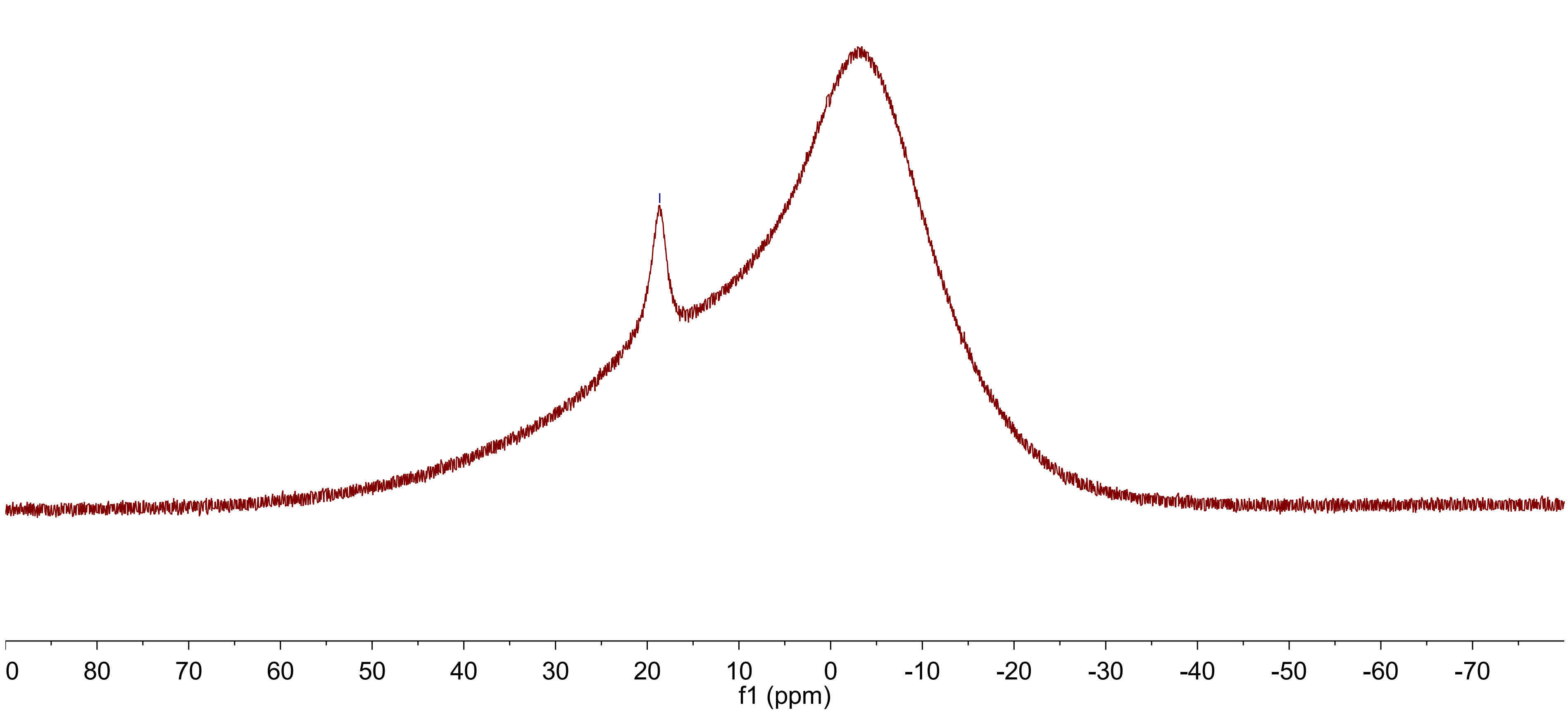

Supplementary Figure 13: ^{11}B NMR (160 MHz, $CDCl_3$) spectrum of **3d**.

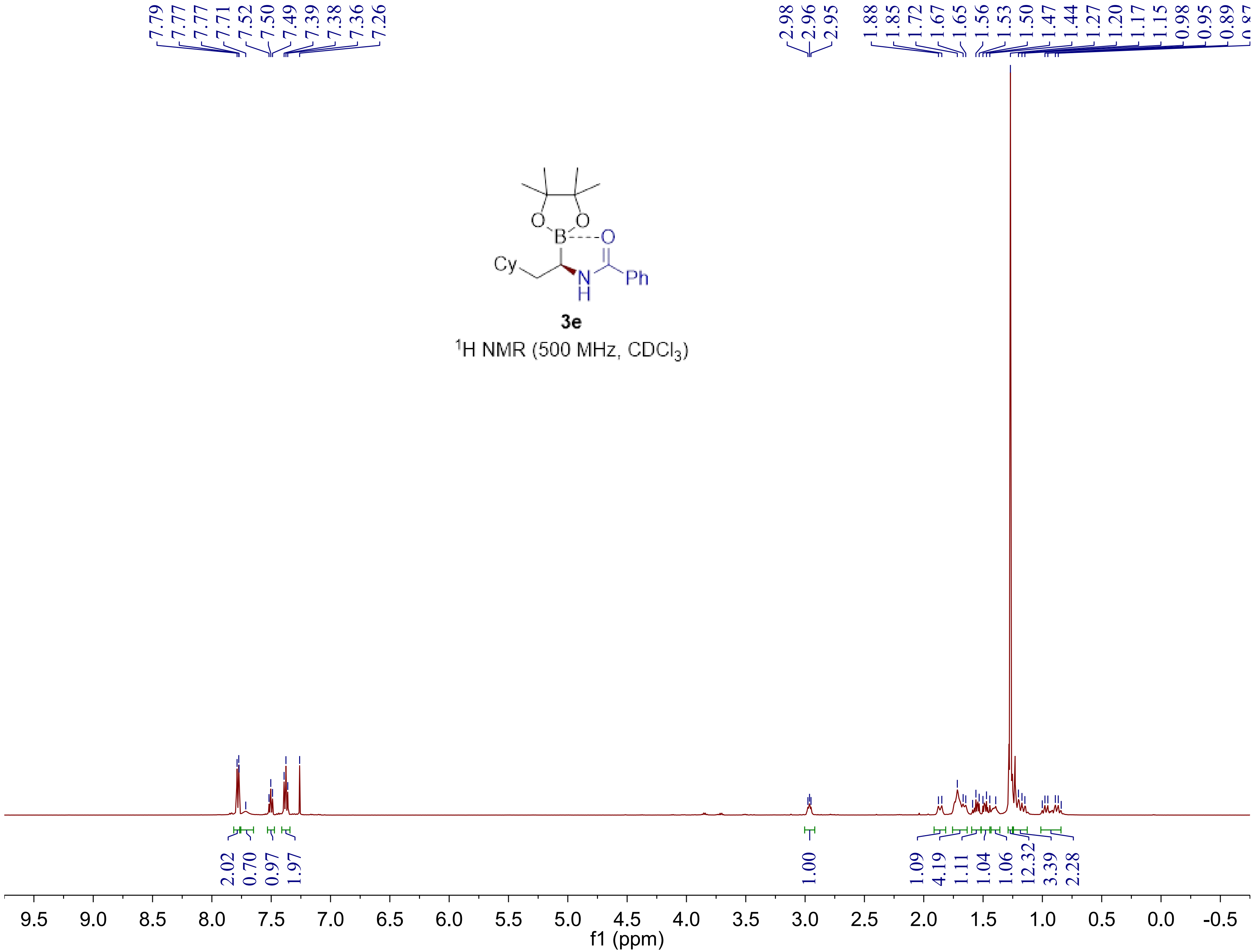

Supplementary Figure 14: ^{1}H NMR (500 MHz, $CDCl_3$) spectrum of **3e**.

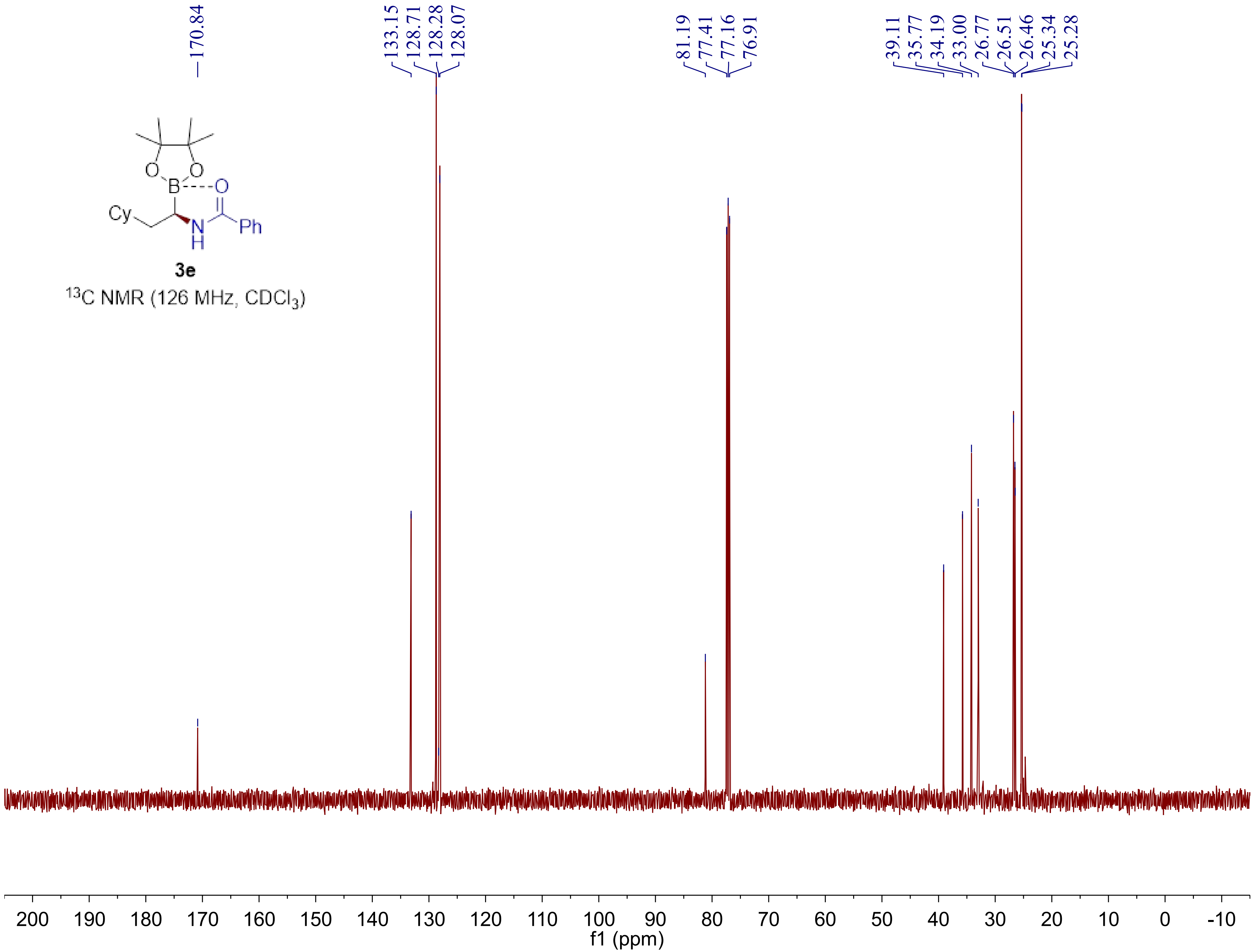

Supplementary Figure 15: ^{13}C NMR (126 MHz, $CDCl_3$) spectrum of **3e**.

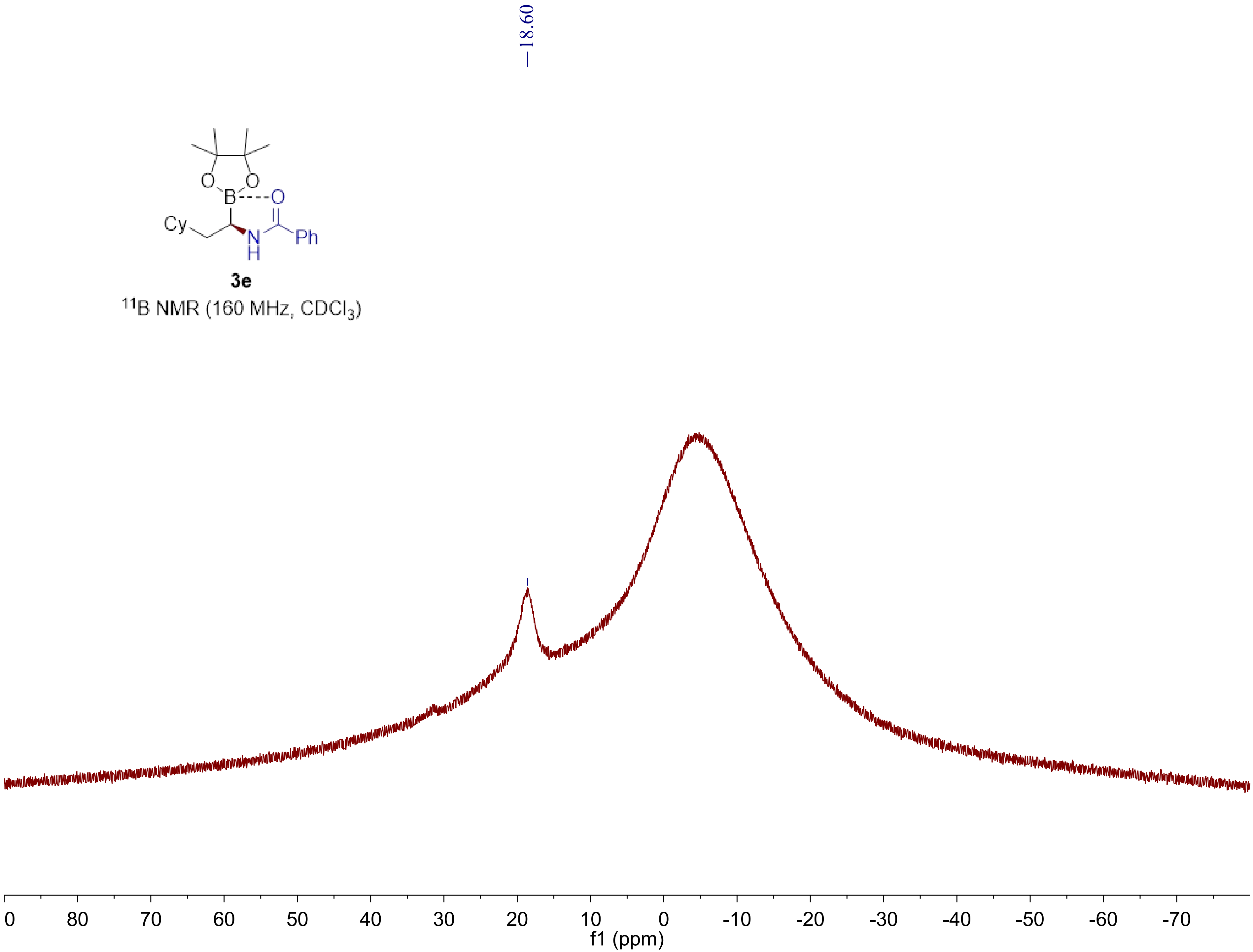

Supplementary Figure 16: ^{11}B NMR (160 MHz, $CDCl_3$) spectrum of **3e**.

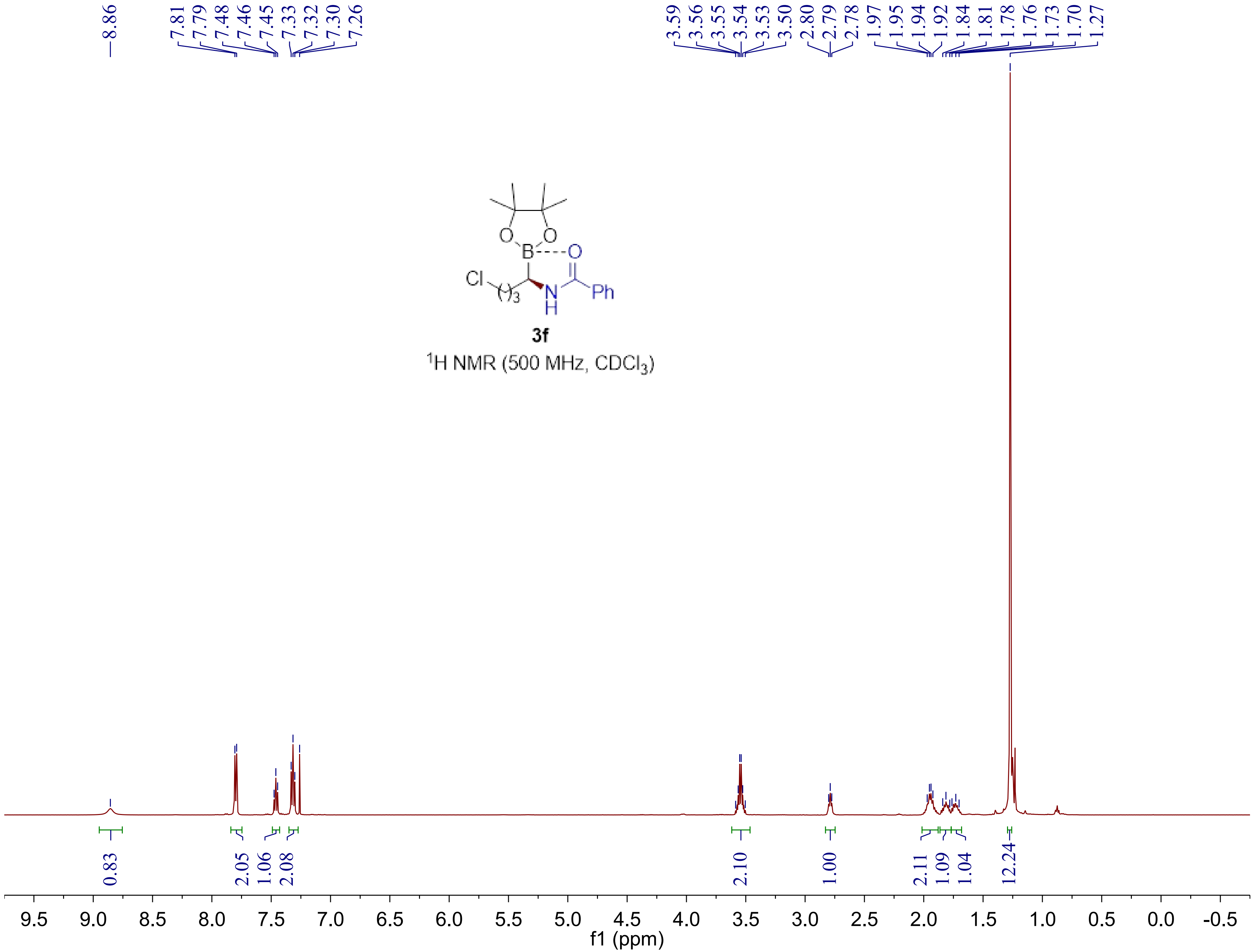

Supplementary Figure 17: ^{1}H NMR (500 MHz, $CDCl_3$) spectrum of **3f**.

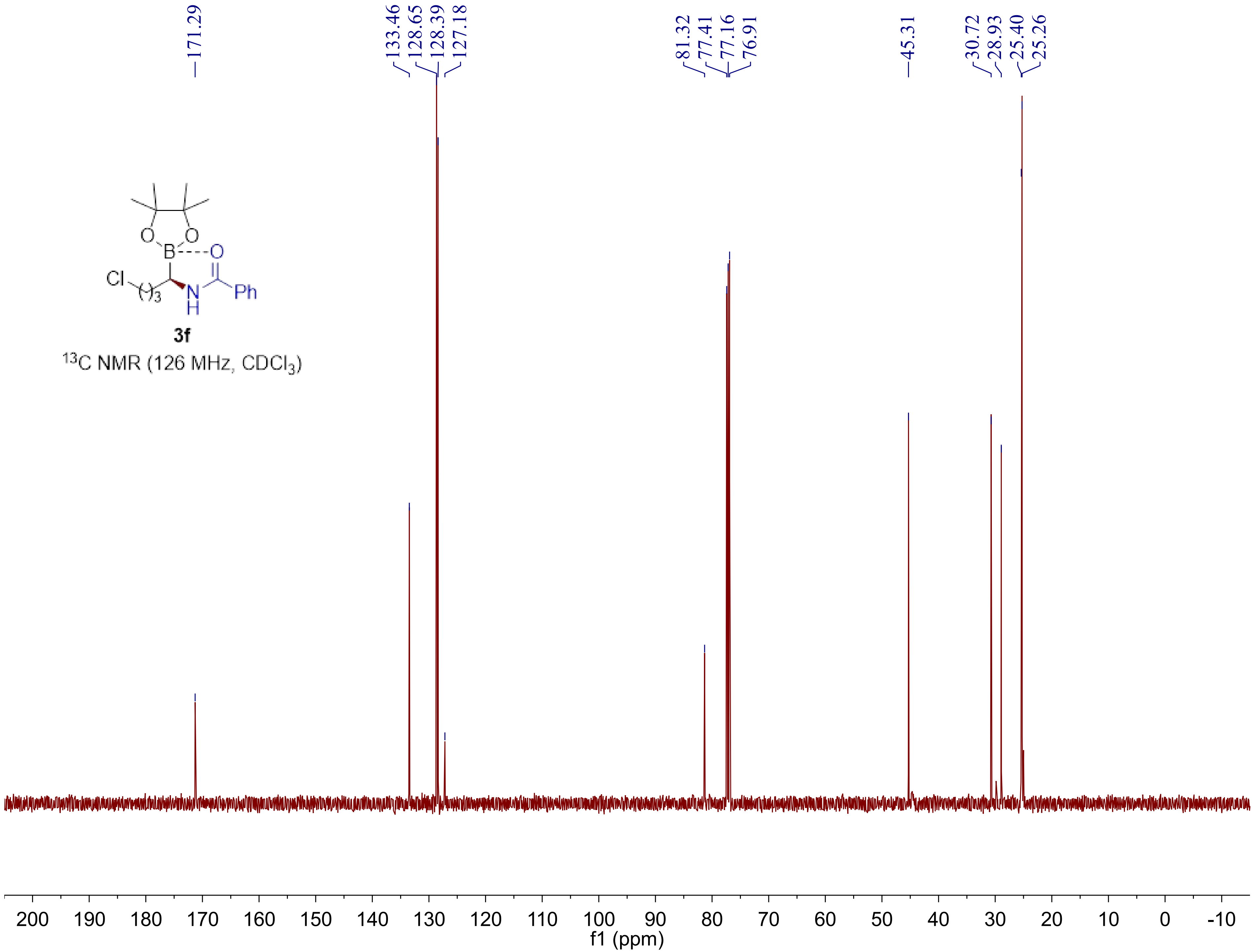

Supplementary Figure 18: ^{13}C NMR (126 MHz, $CDCl_3$) spectrum of **3f**.

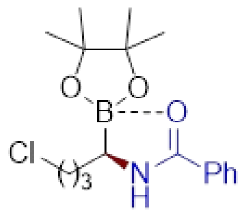

3f

^{11}B NMR (160 MHz, CDCl$_3$)

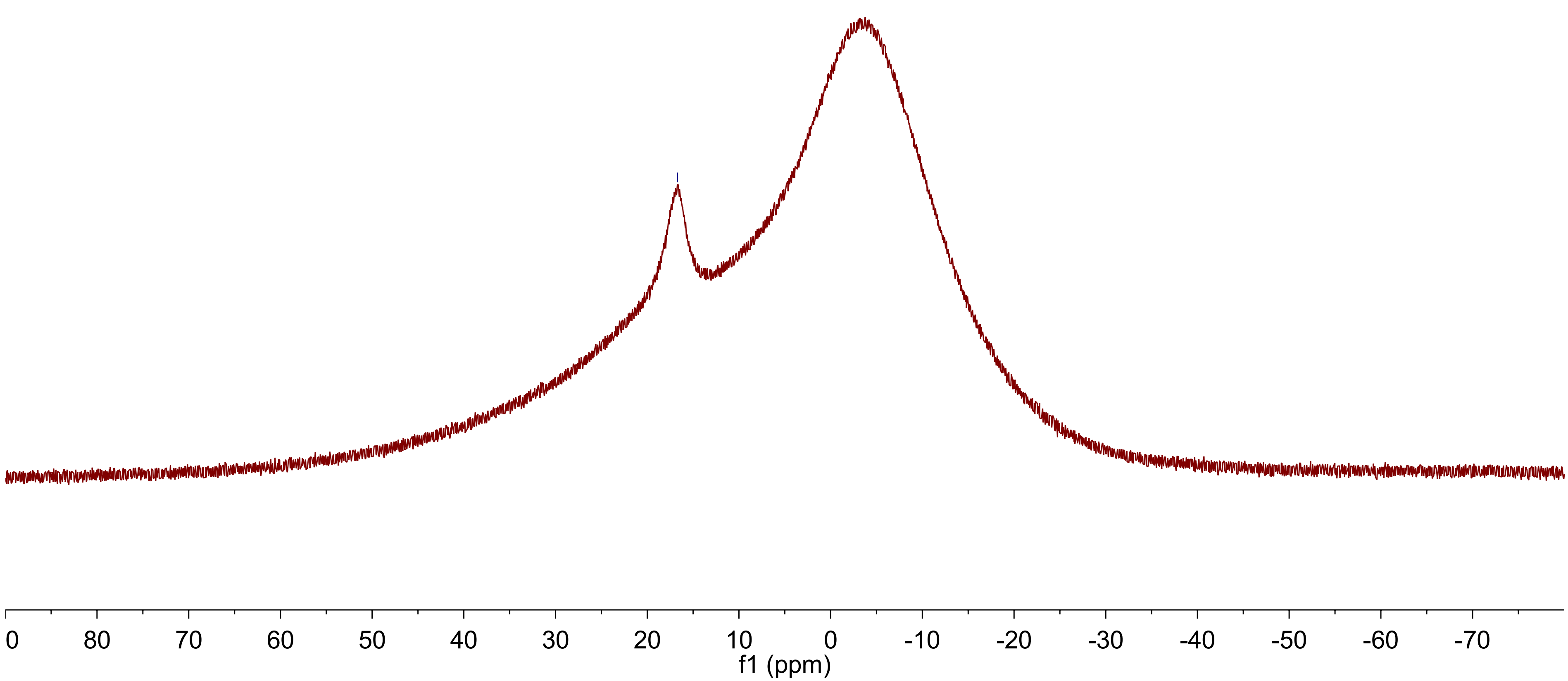

Supplementary Figure 19: ^{11}B NMR (160 MHz, CDCl$_3$) spectrum of **3f**.

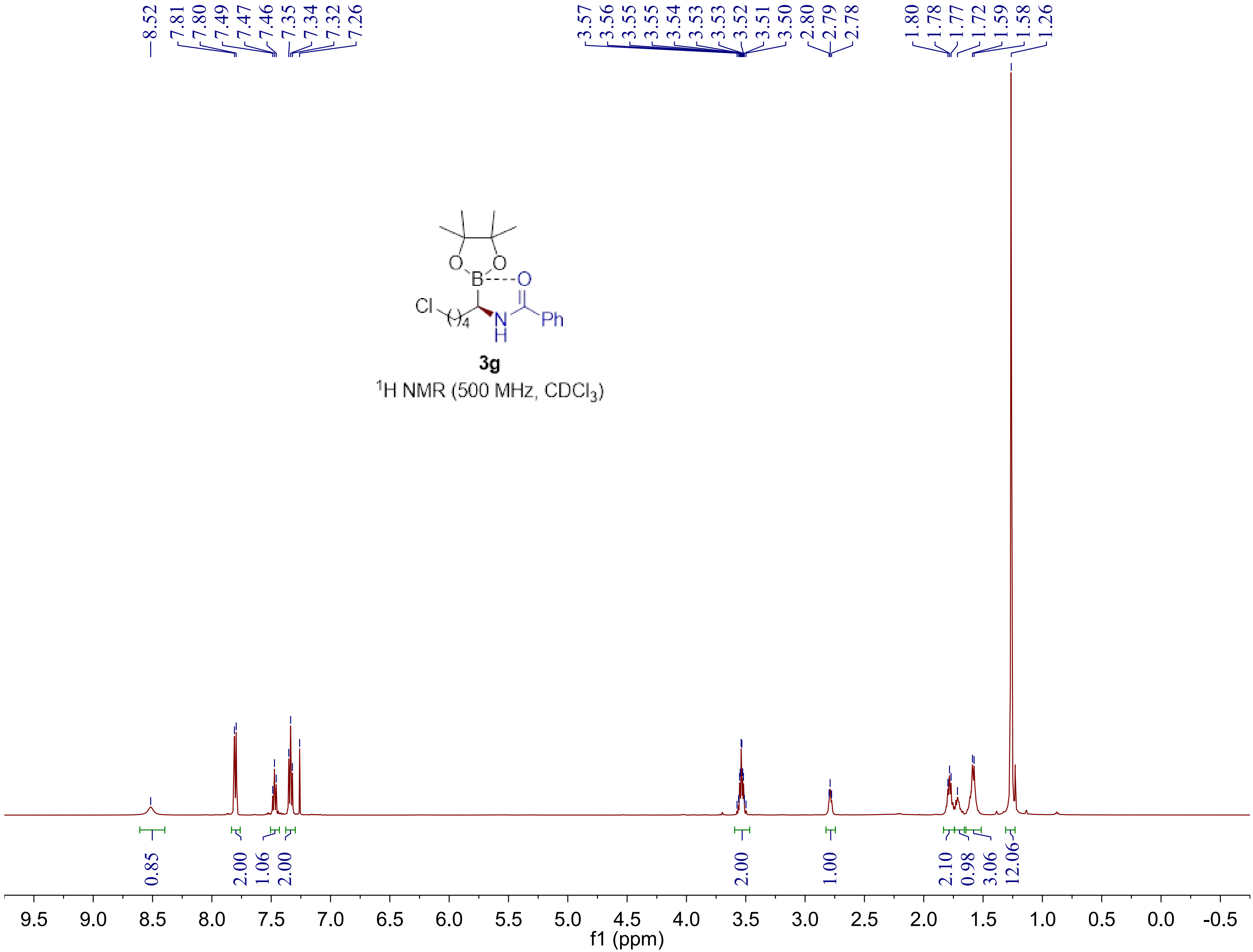

Supplementary Figure 20: ^{1}H NMR (500 MHz, $CDCl_3$) spectrum of **3g**.

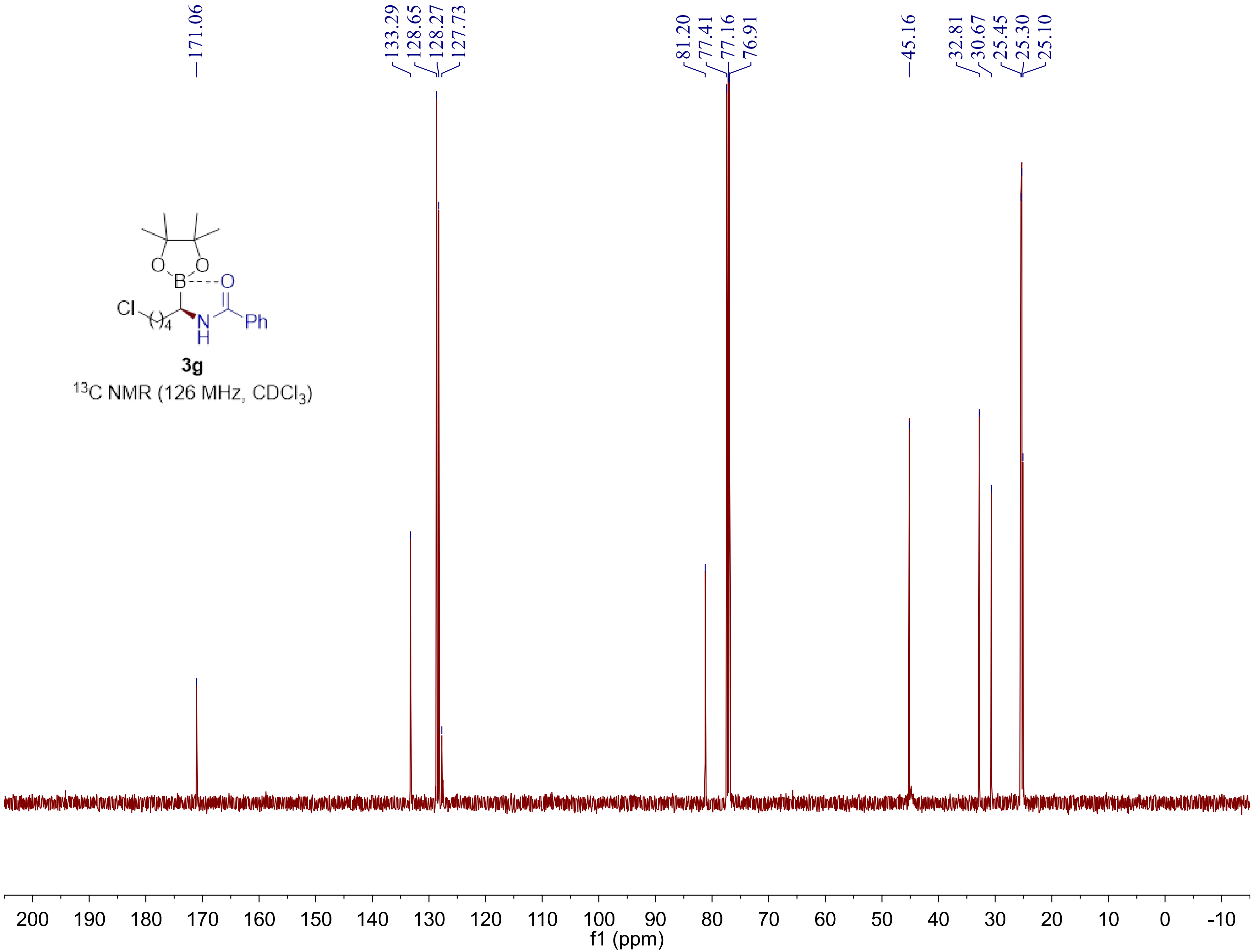

Supplementary Figure 21: ^{13}C NMR (126 MHz, $CDCl_3$) spectrum of **3g**.

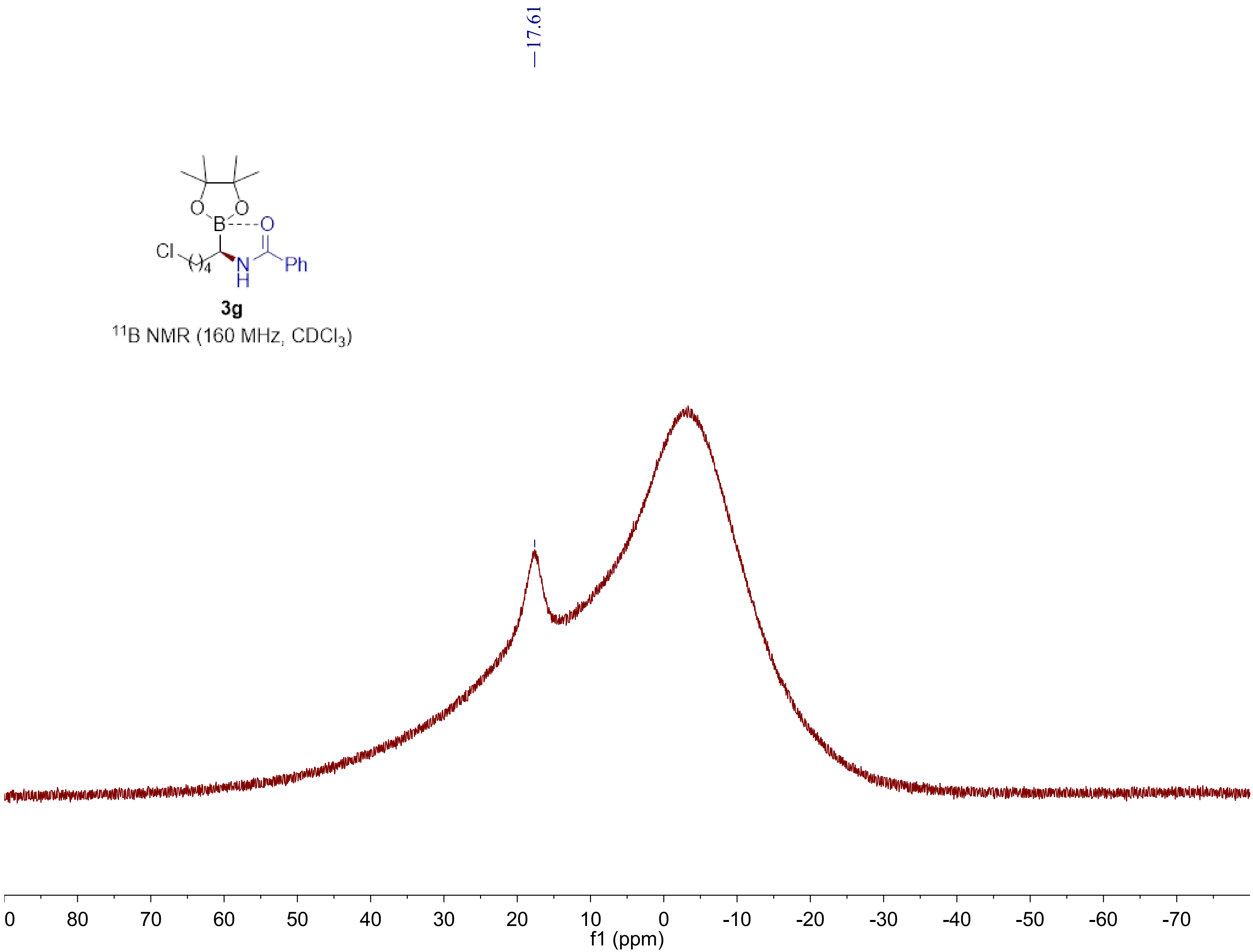

Supplementary Figure 22: ^{11}B NMR (160 MHz, $CDCl_3$) spectrum of **3g**.

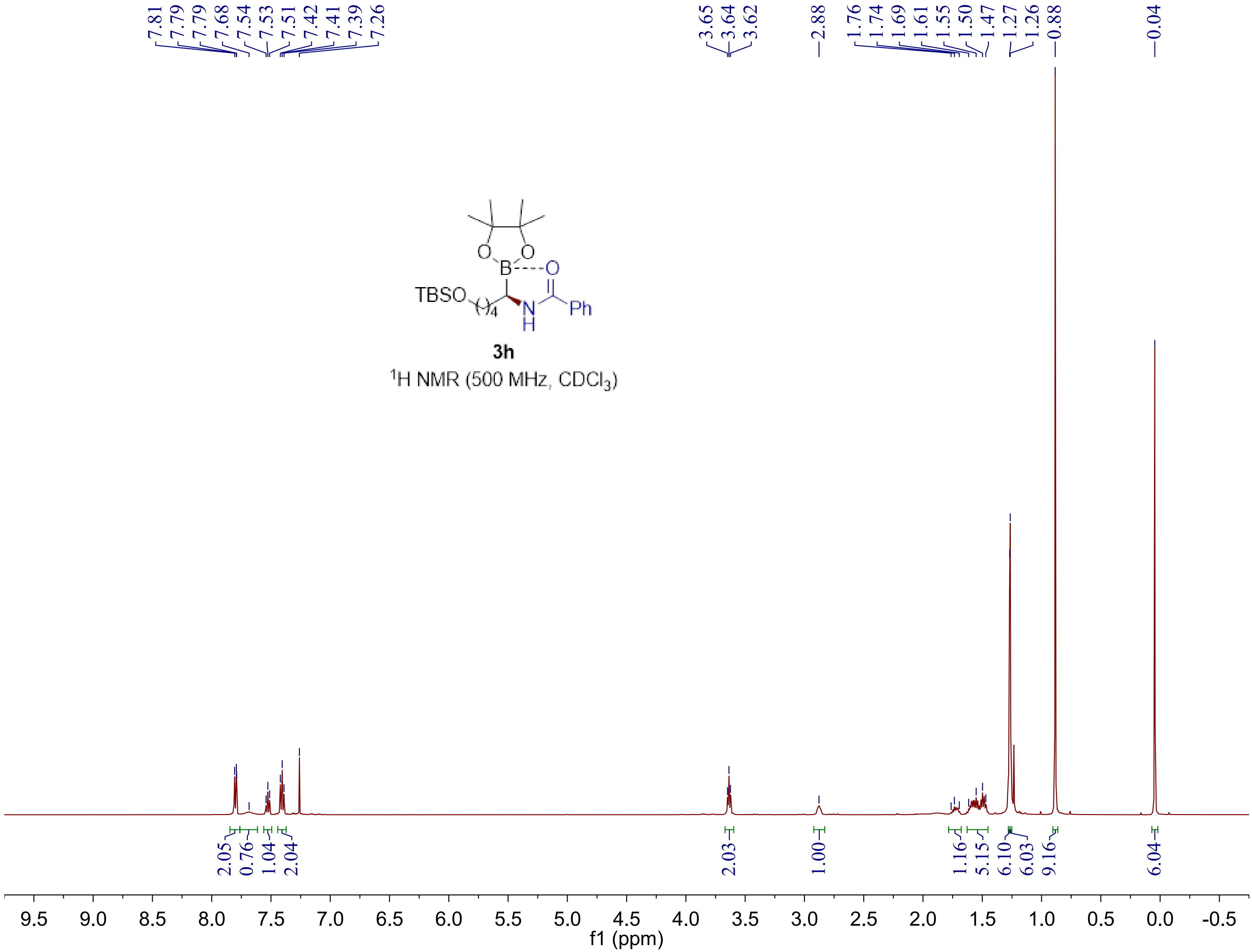

Supplementary Figure 23: ^{1}H NMR (500 MHz, $CDCl_3$) spectrum of **3h**.

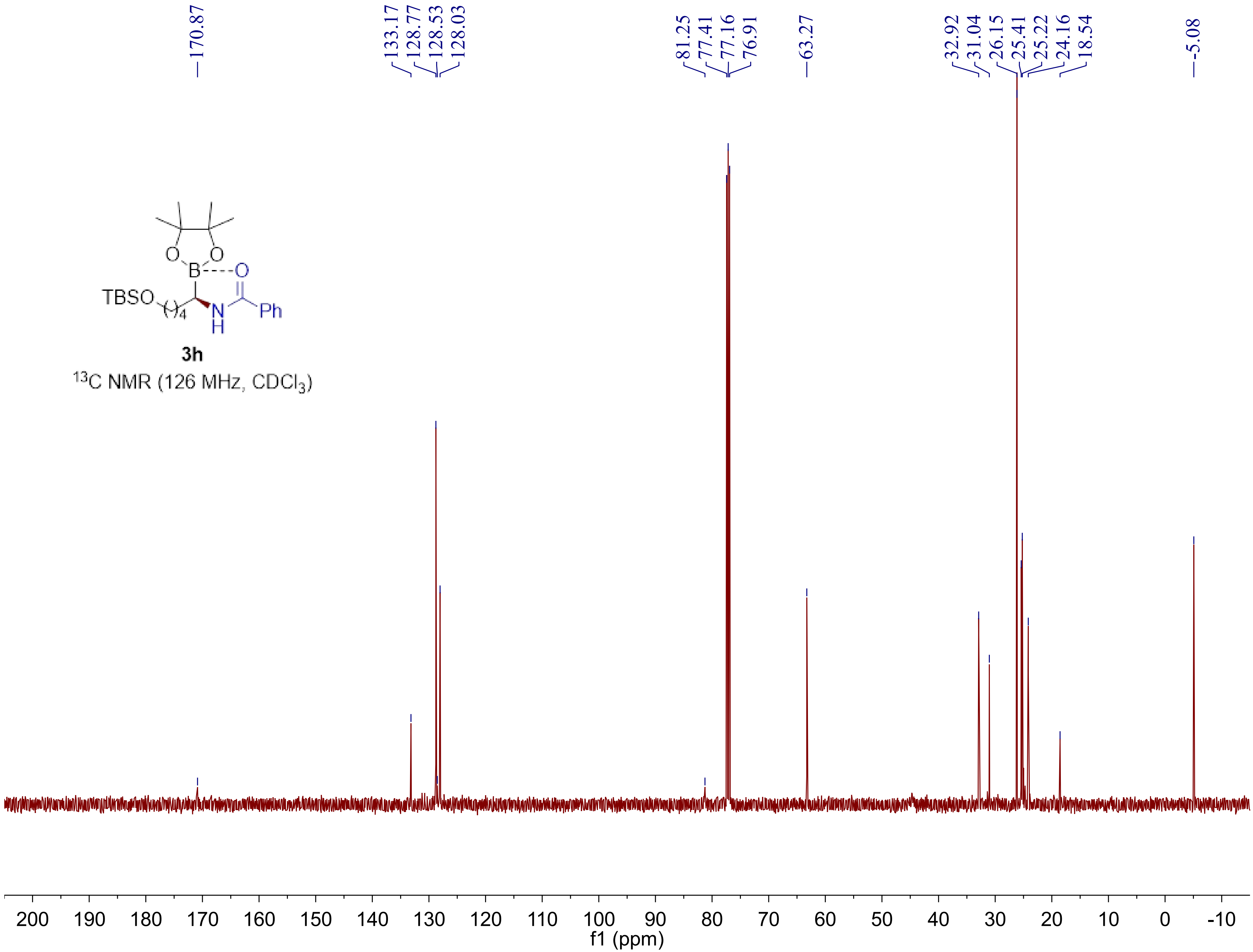

Supplementary Figure 24: ^{13}C NMR (126 MHz, $CDCl_3$) spectrum of **3h**.

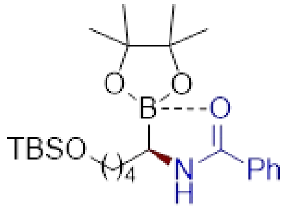

3h

^{11}B NMR (160 MHz, $CDCl_3$)

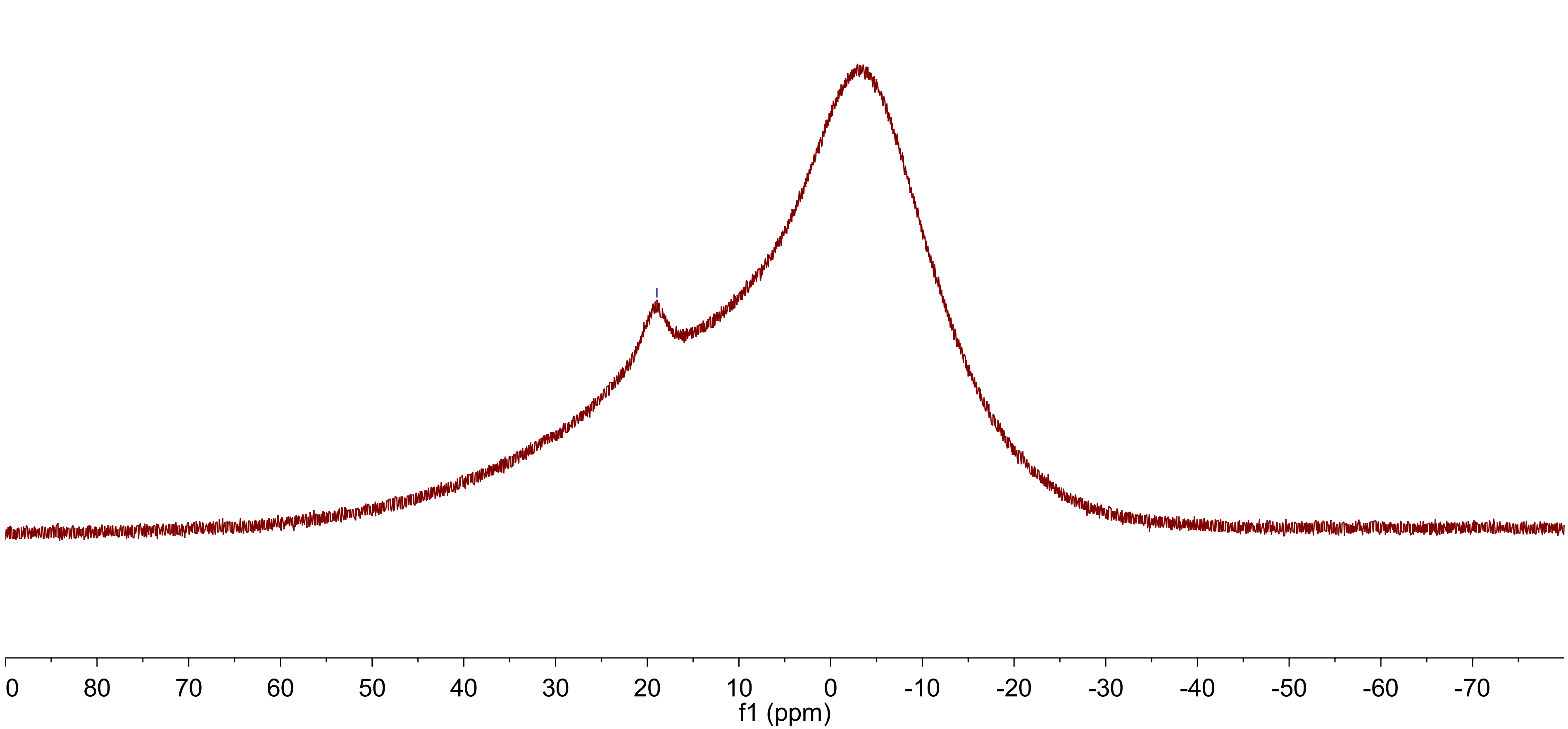

Supplementary Figure 25: ^{11}B NMR (160 MHz, $CDCl_3$) spectrum of **3h**.

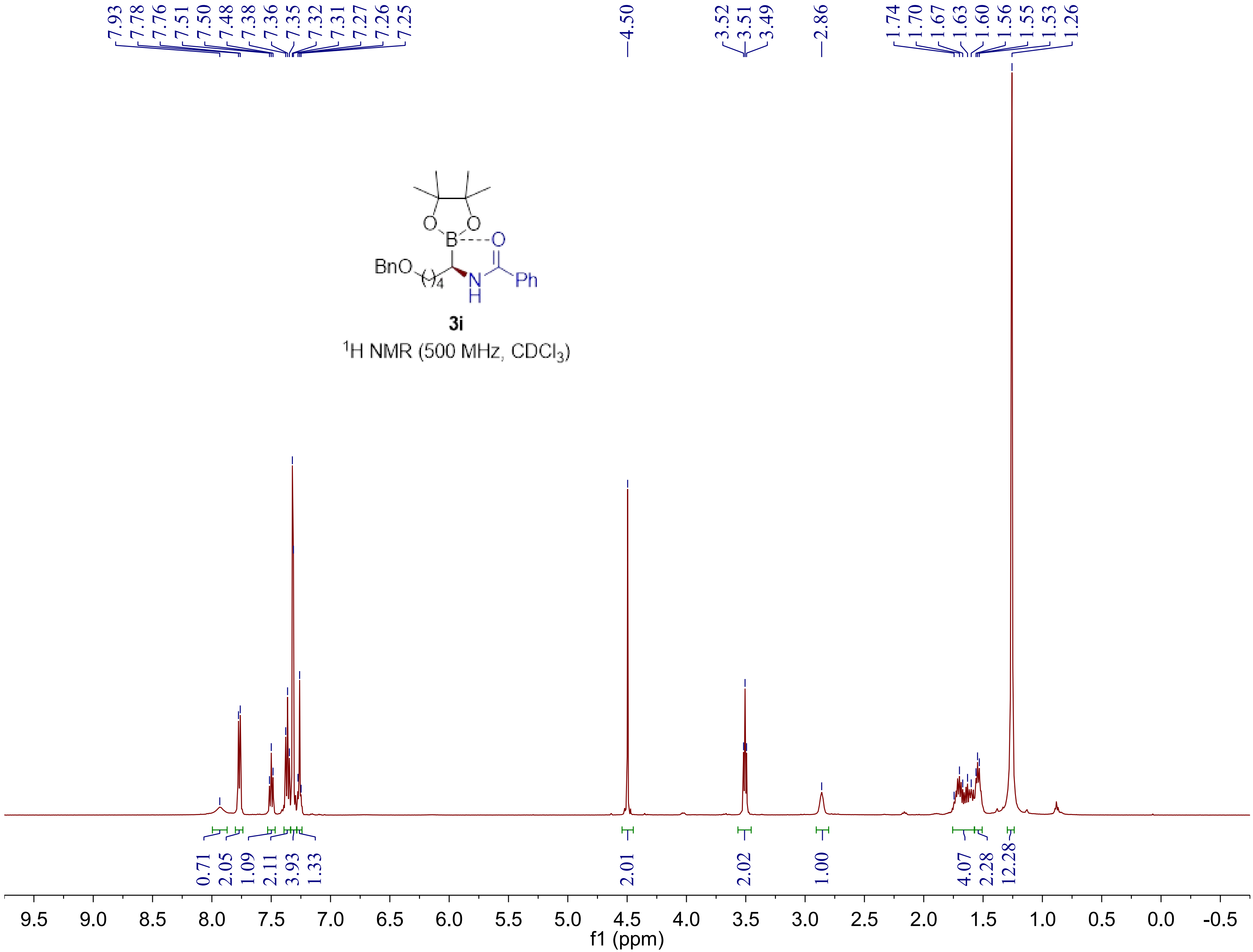

Supplementary Figure 26: ^{1}H NMR (500 MHz, $CDCl_3$) spectrum of **3i**.

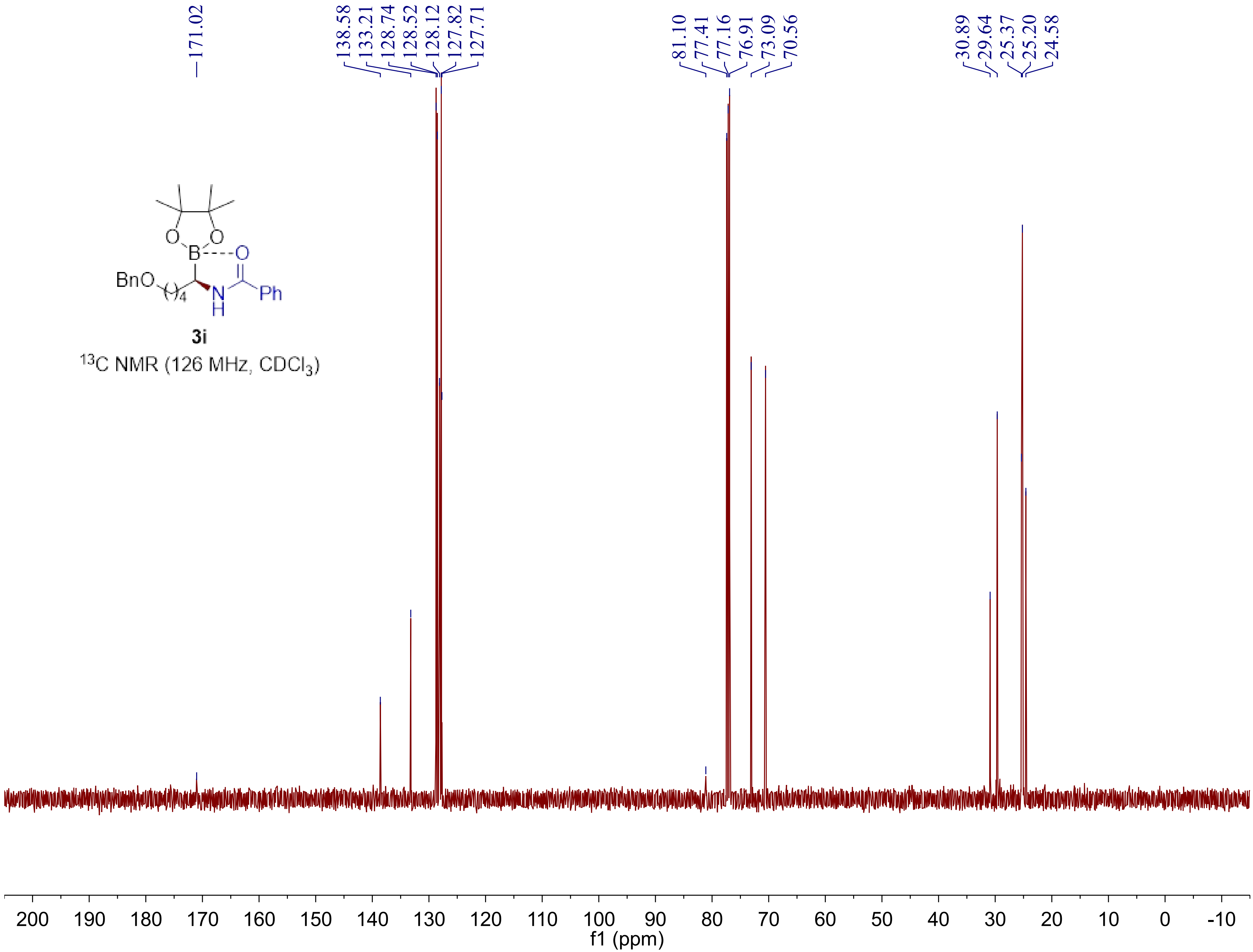

Supplementary Figure 27: ^{13}C NMR (126 MHz, CDCl$_3$) spectrum of **3i**.

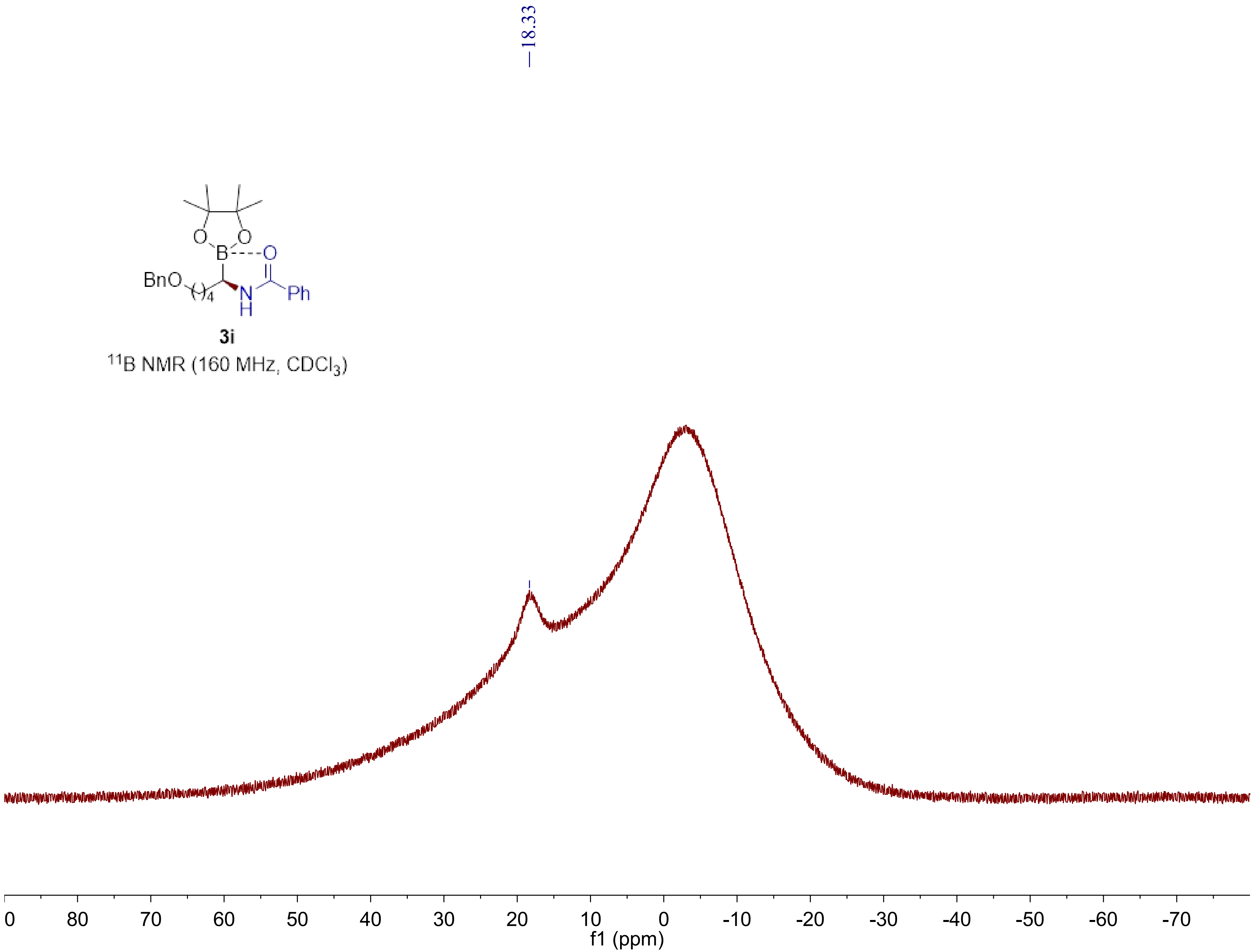

Supplementary Figure 28: ^{11}B NMR (160 MHz, $CDCl_3$) spectrum of **3i**.

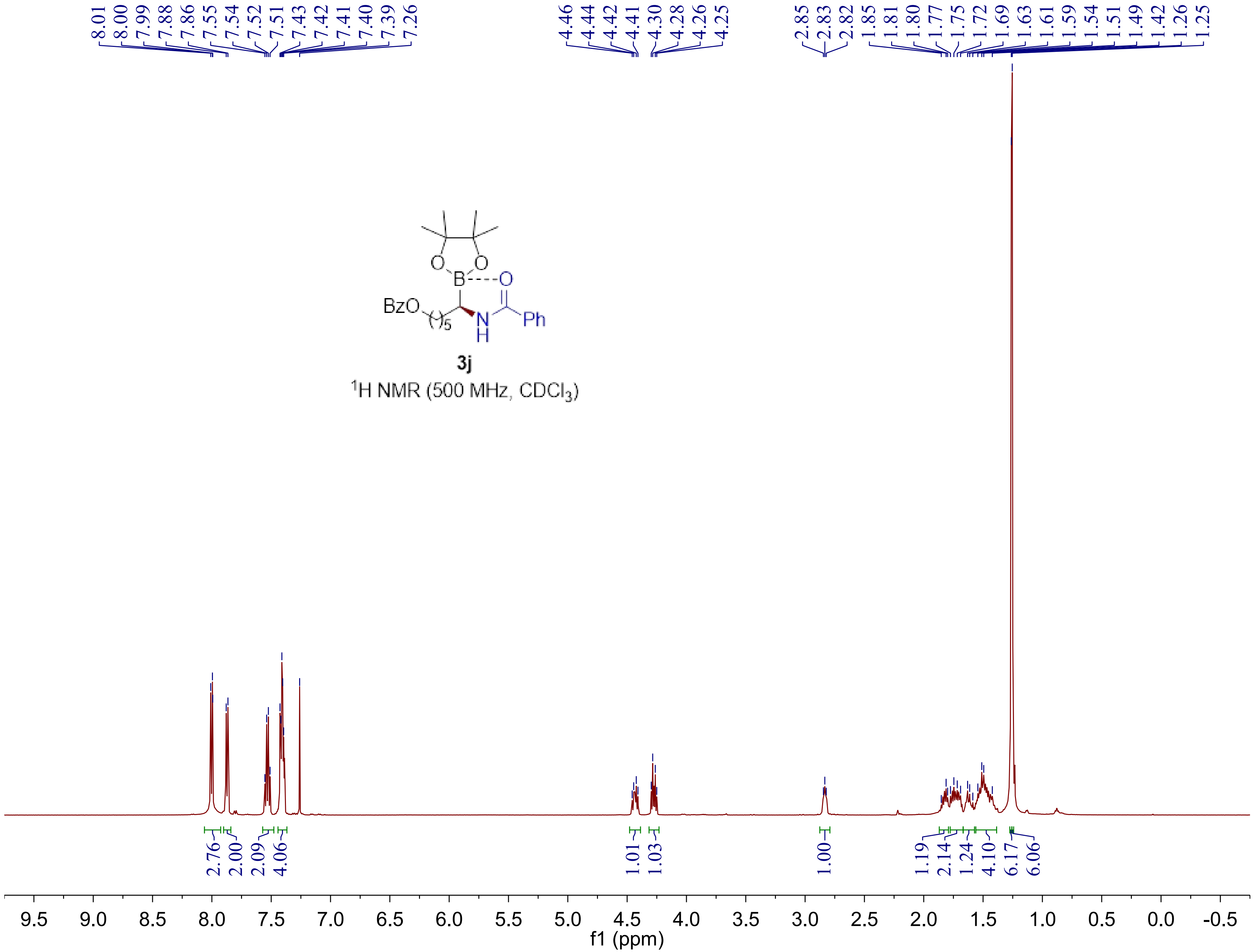

Supplementary Figure 29: ^{1}H NMR (500 MHz, $CDCl_3$) spectrum of **3j**.

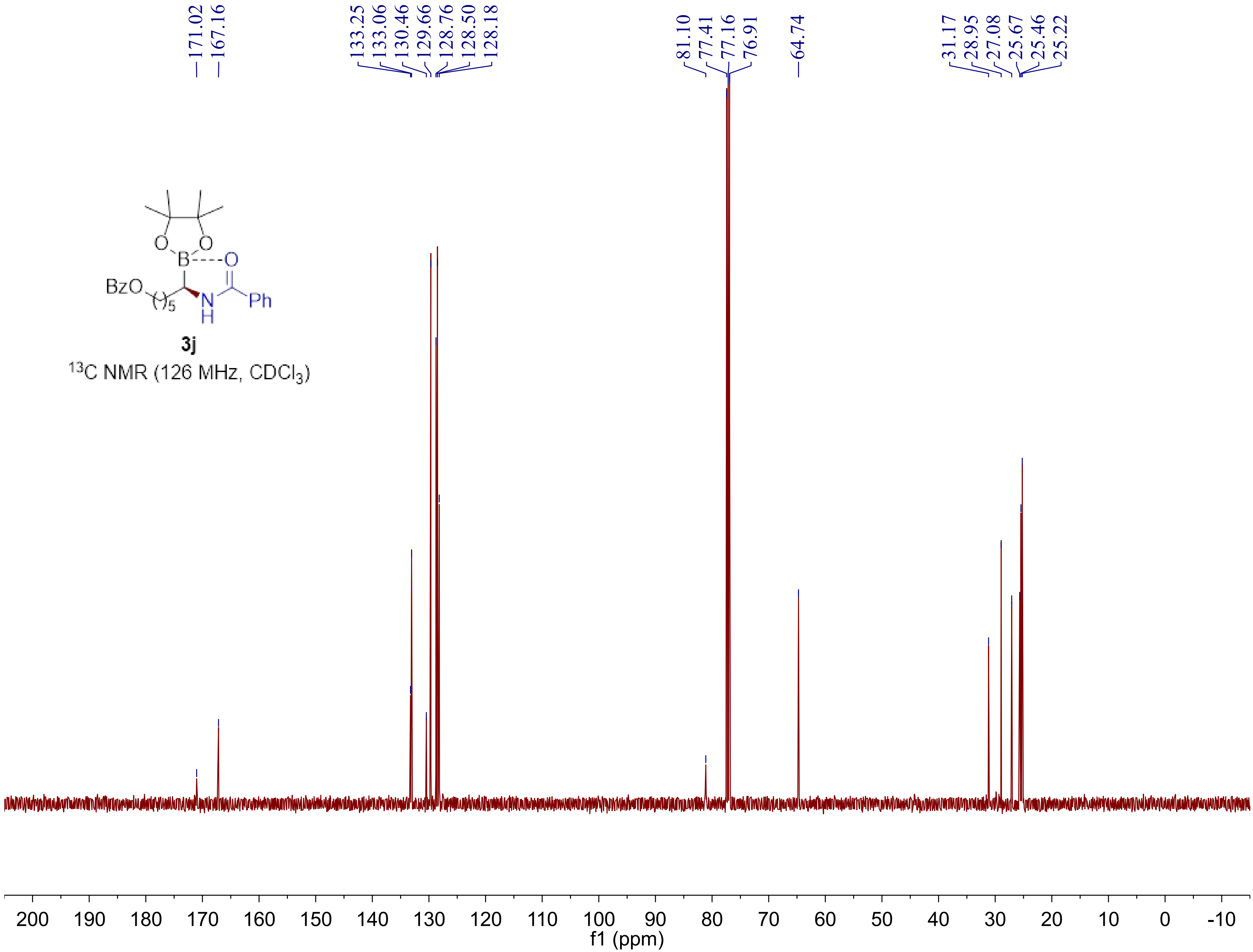

Supplementary Figure 30: ^{13}C NMR (126 MHz, $CDCl_3$) spectrum of **3j**.

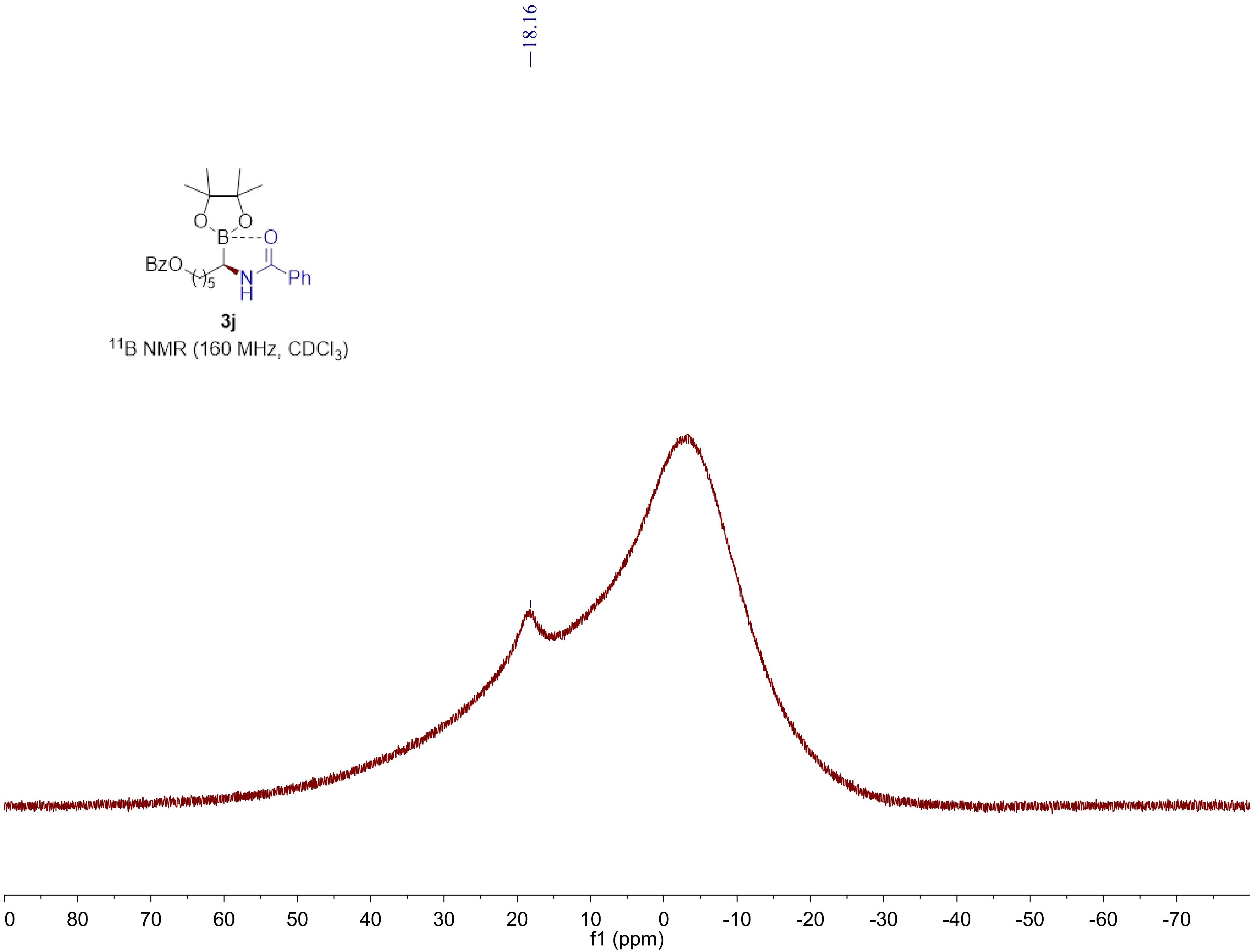

Supplementary Figure 31: ^{11}B NMR (160 MHz, $CDCl_3$) spectrum of **3j**.

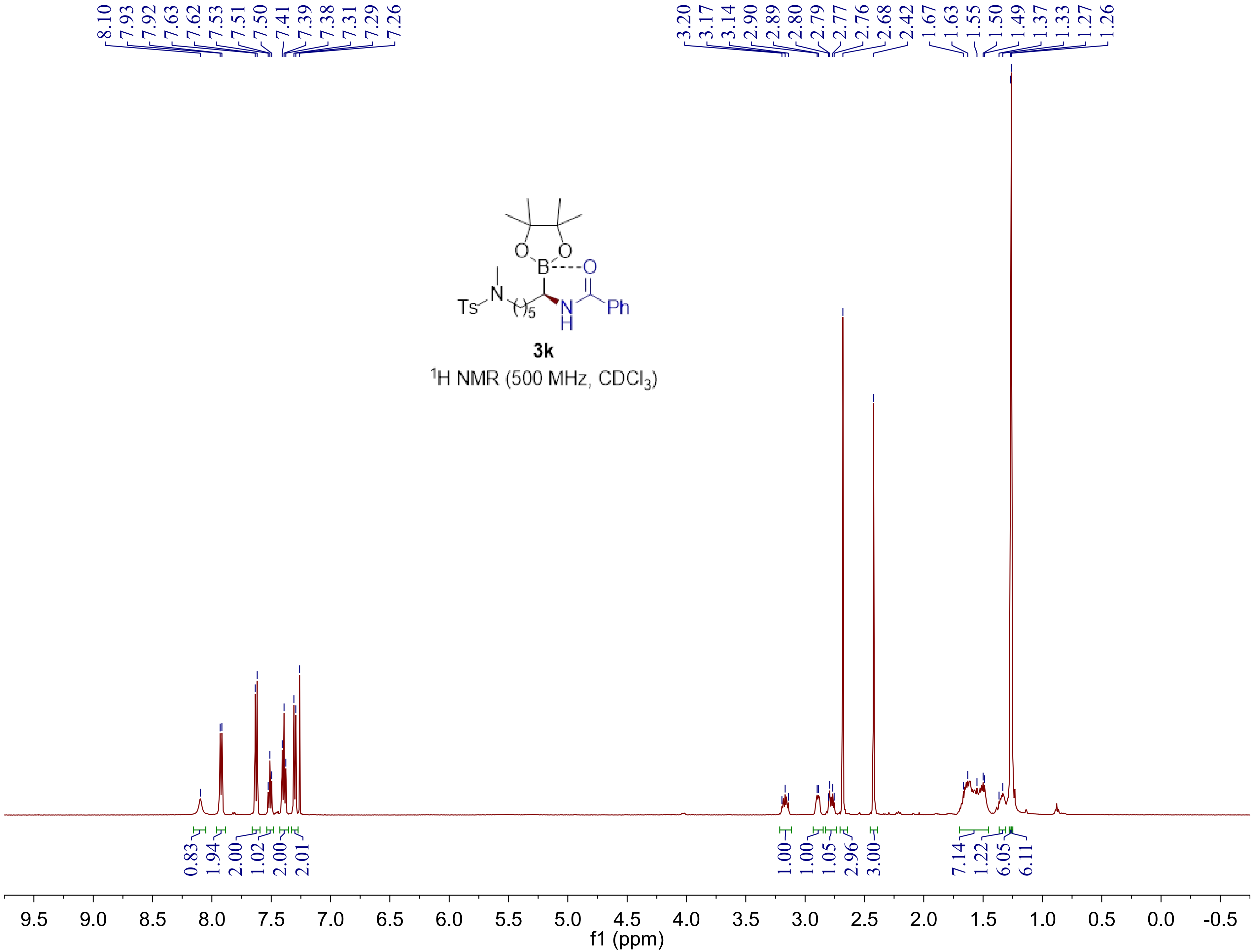

Supplementary Figure 32: ^{1}H NMR (500 MHz, $CDCl_3$) spectrum of **3k**.

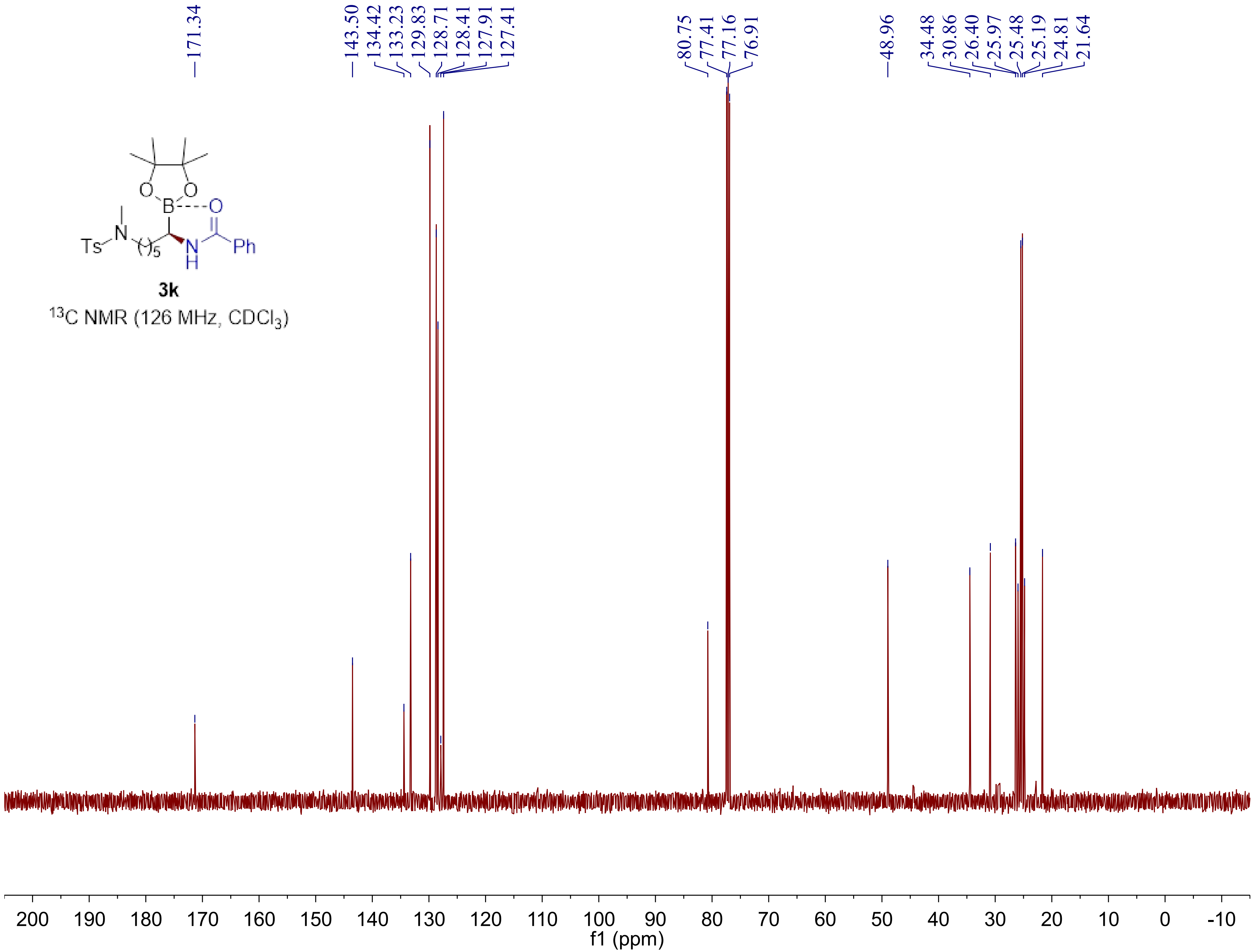

Supplementary Figure 33: ^{13}C NMR (126 MHz, $CDCl_3$) spectrum of **3k**.

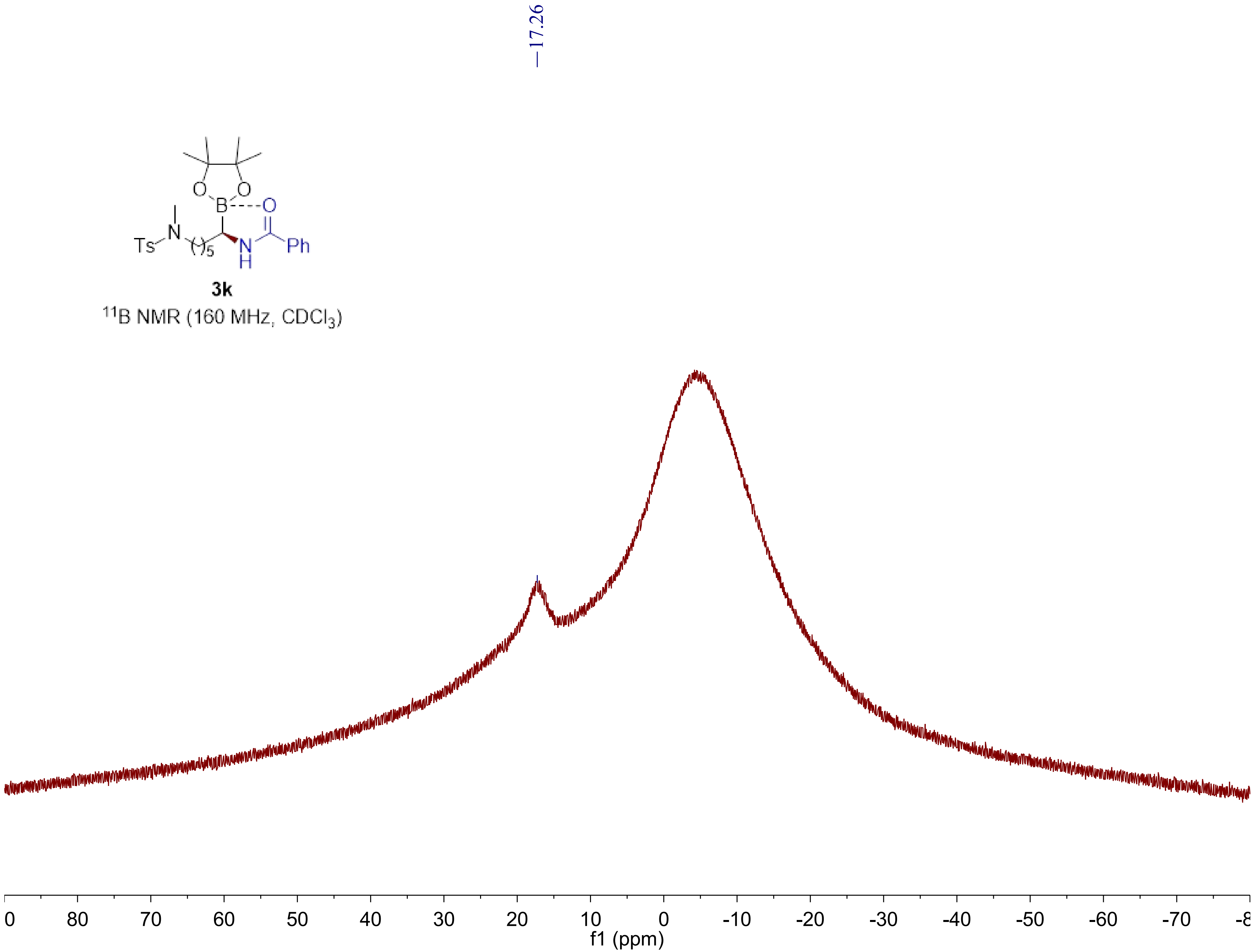

Supplementary Figure 34: ^{11}B NMR (160 MHz, $CDCl_3$) spectrum of **3k**.

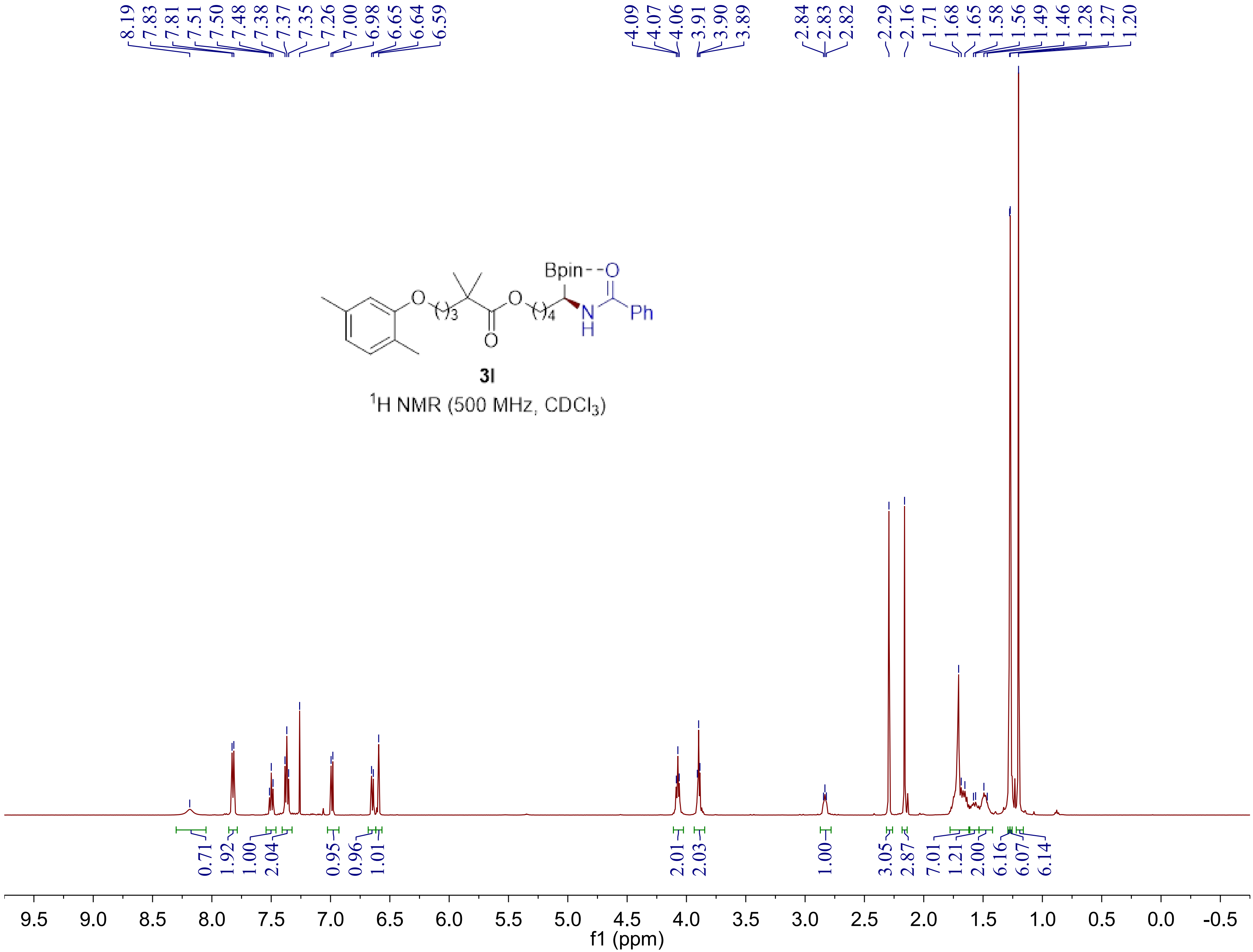

Supplementary Figure 35: ^{1}H NMR (500 MHz, $CDCl_3$) spectrum of **3l**.

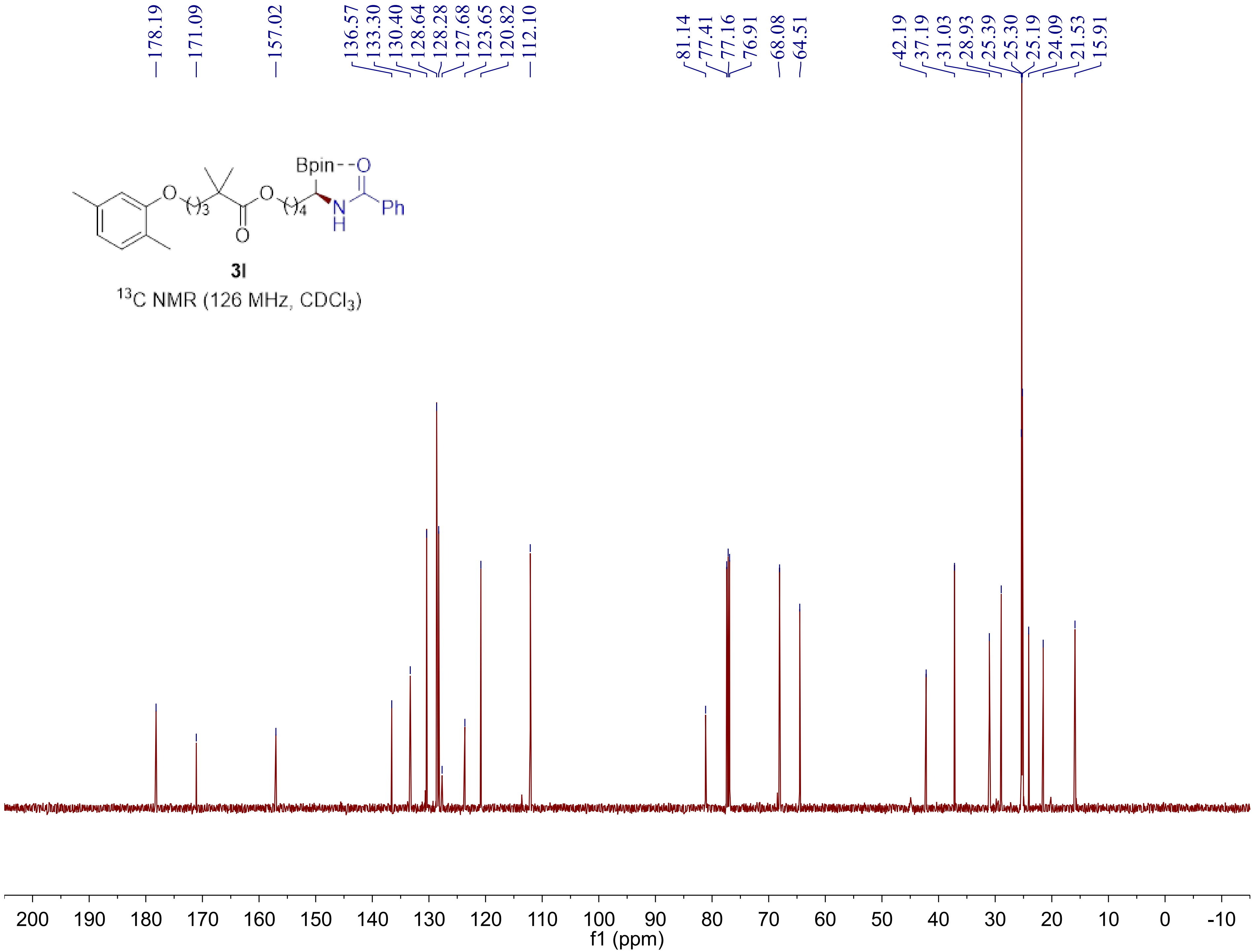

Supplementary Figure 36: ^{13}C NMR (126 MHz, $CDCl_3$) spectrum of **3l**.

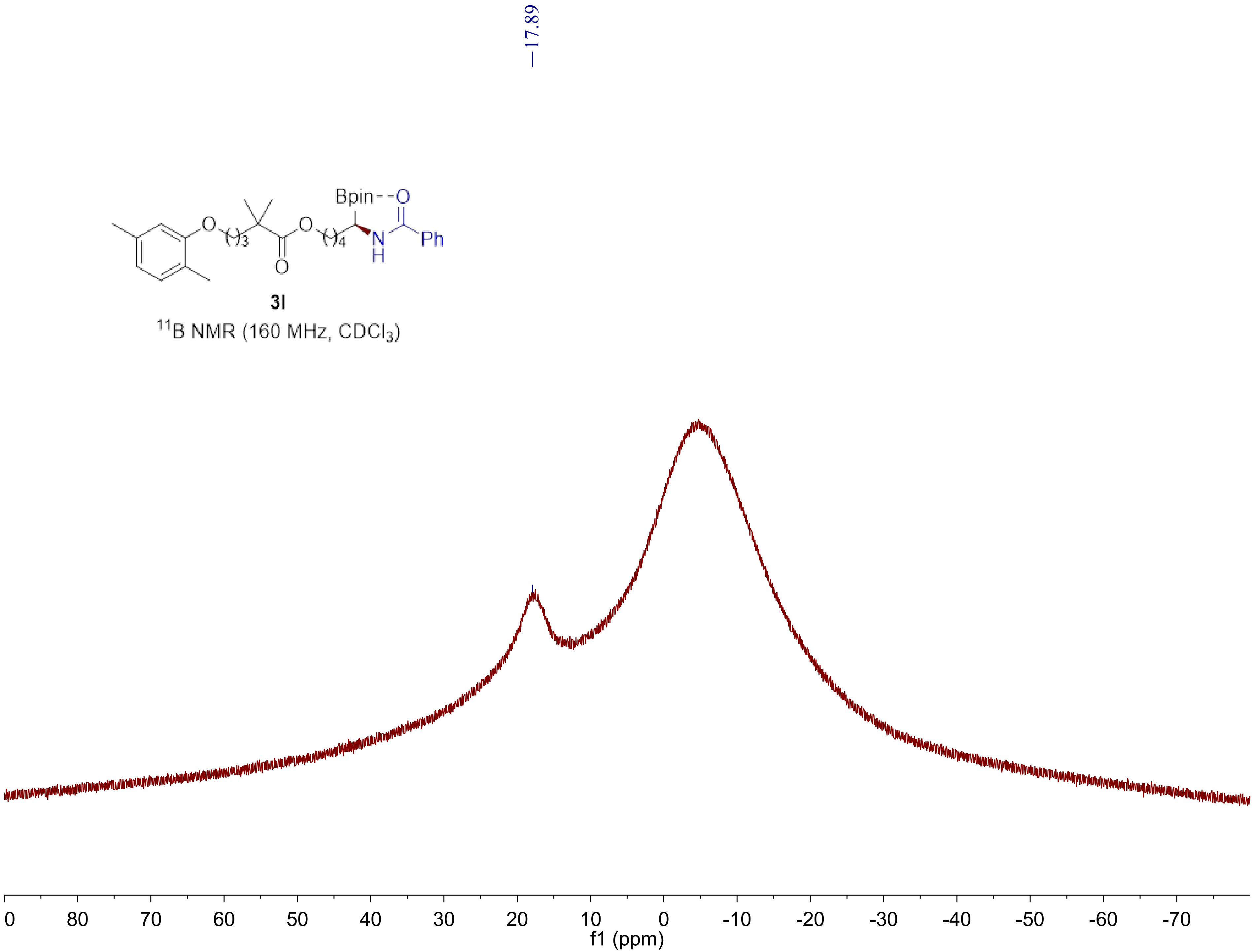

Supplementary Figure 37: ^{11}B NMR (160 MHz, $CDCl_3$) spectrum of **3l**.

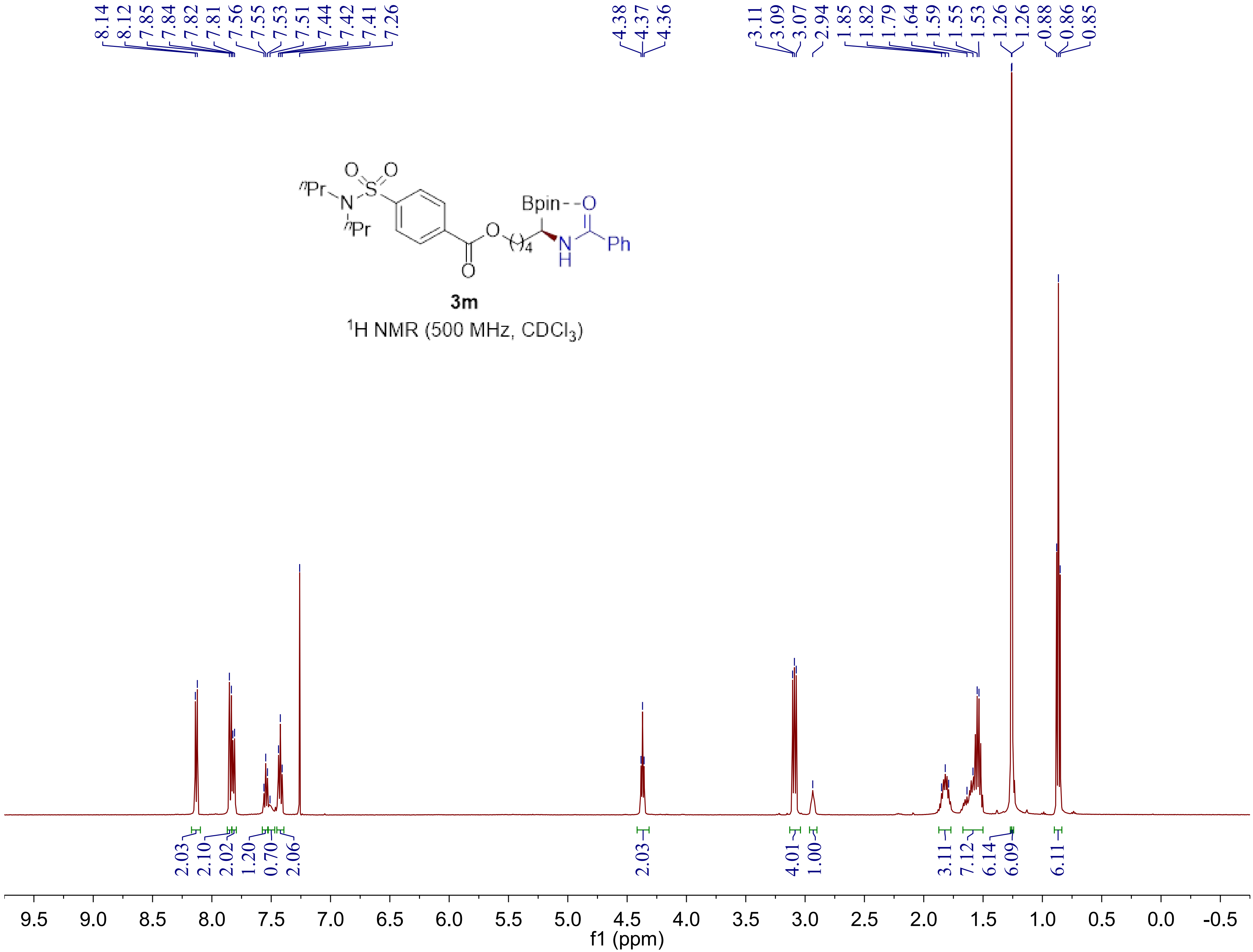

Supplementary Figure 38: ^{1}H NMR (500 MHz, $CDCl_3$) spectrum of **3m**.

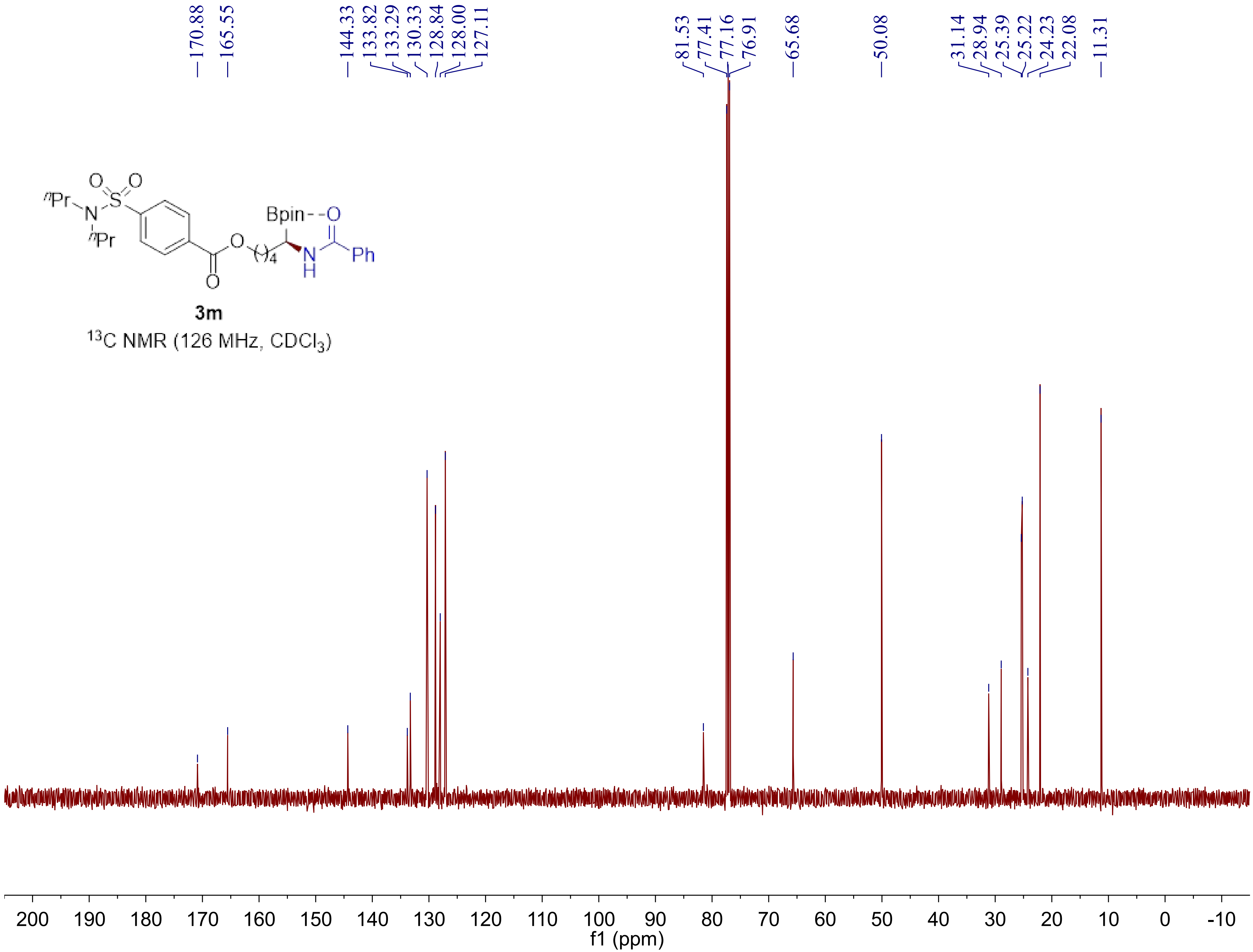

Supplementary Figure 39: ^{13}C NMR (126 MHz, $CDCl_3$) spectrum of **3m**.

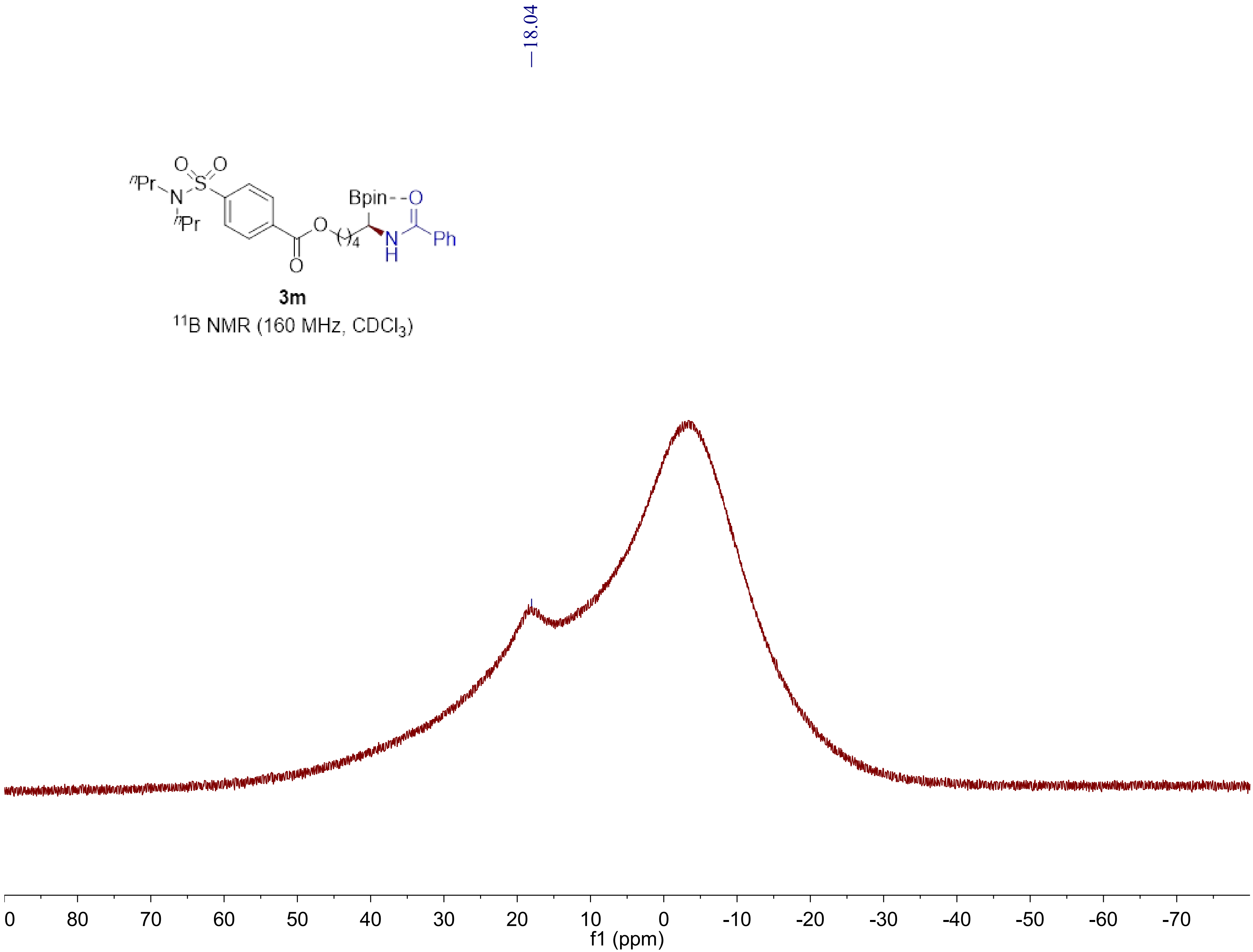

Supplementary Figure 40: ^{11}B NMR (160 MHz, CDCl$_3$) spectrum of **3m**.

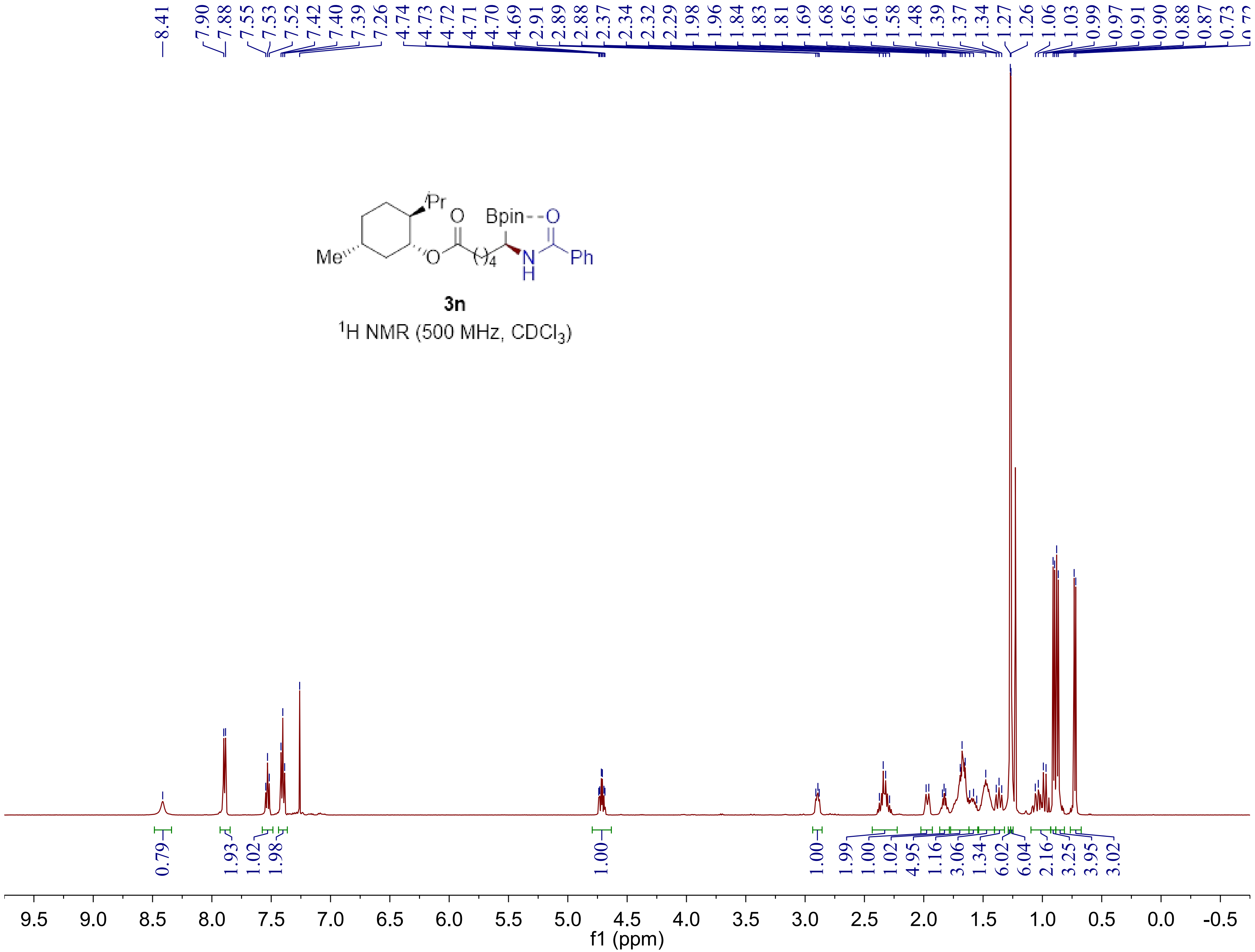

Supplementary Figure 41: ^{1}H NMR (500 MHz, $CDCl_3$) spectrum of **3n**.

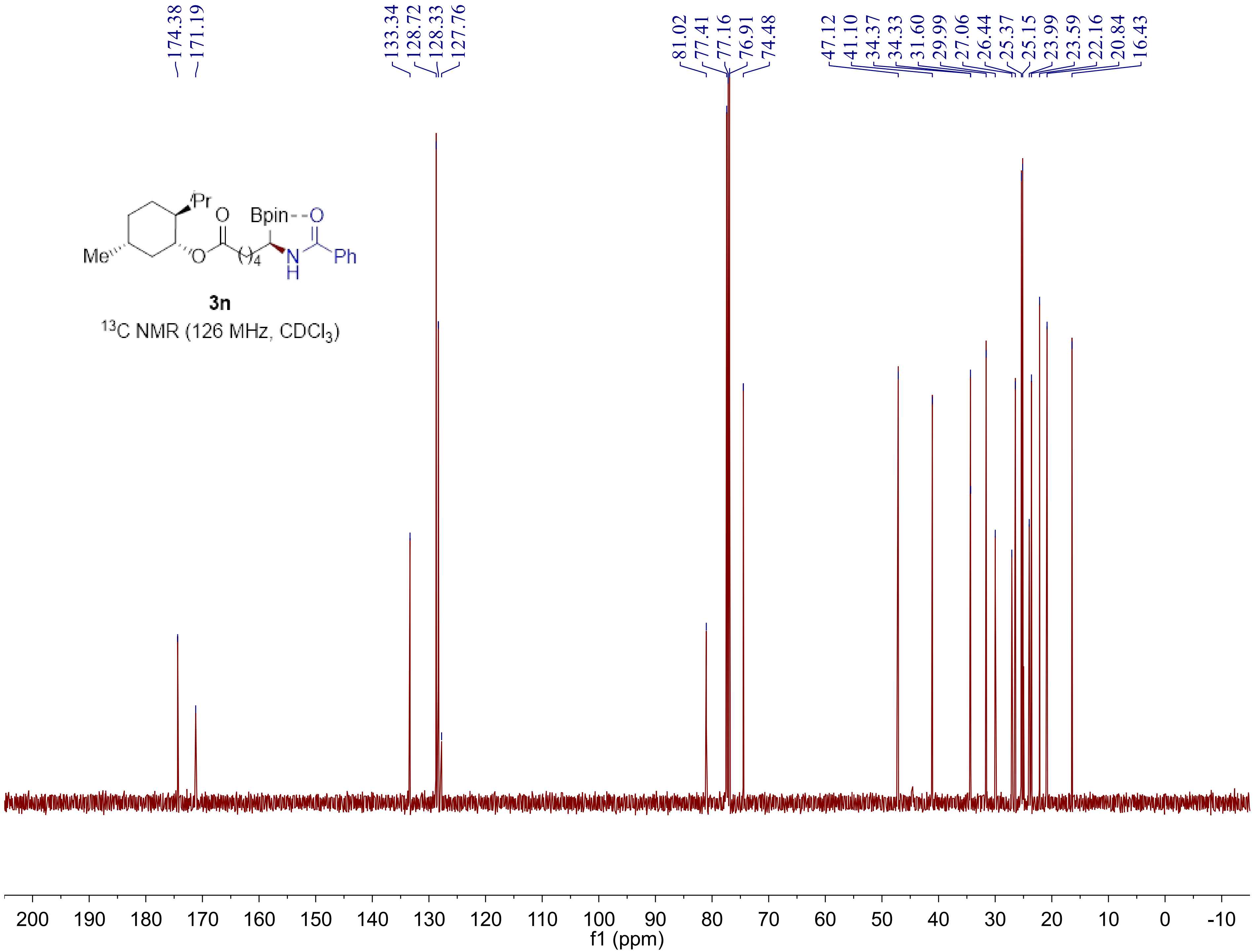

Supplementary Figure 42: ^{13}C NMR (126 MHz, $CDCl_3$) spectrum of **3n**.

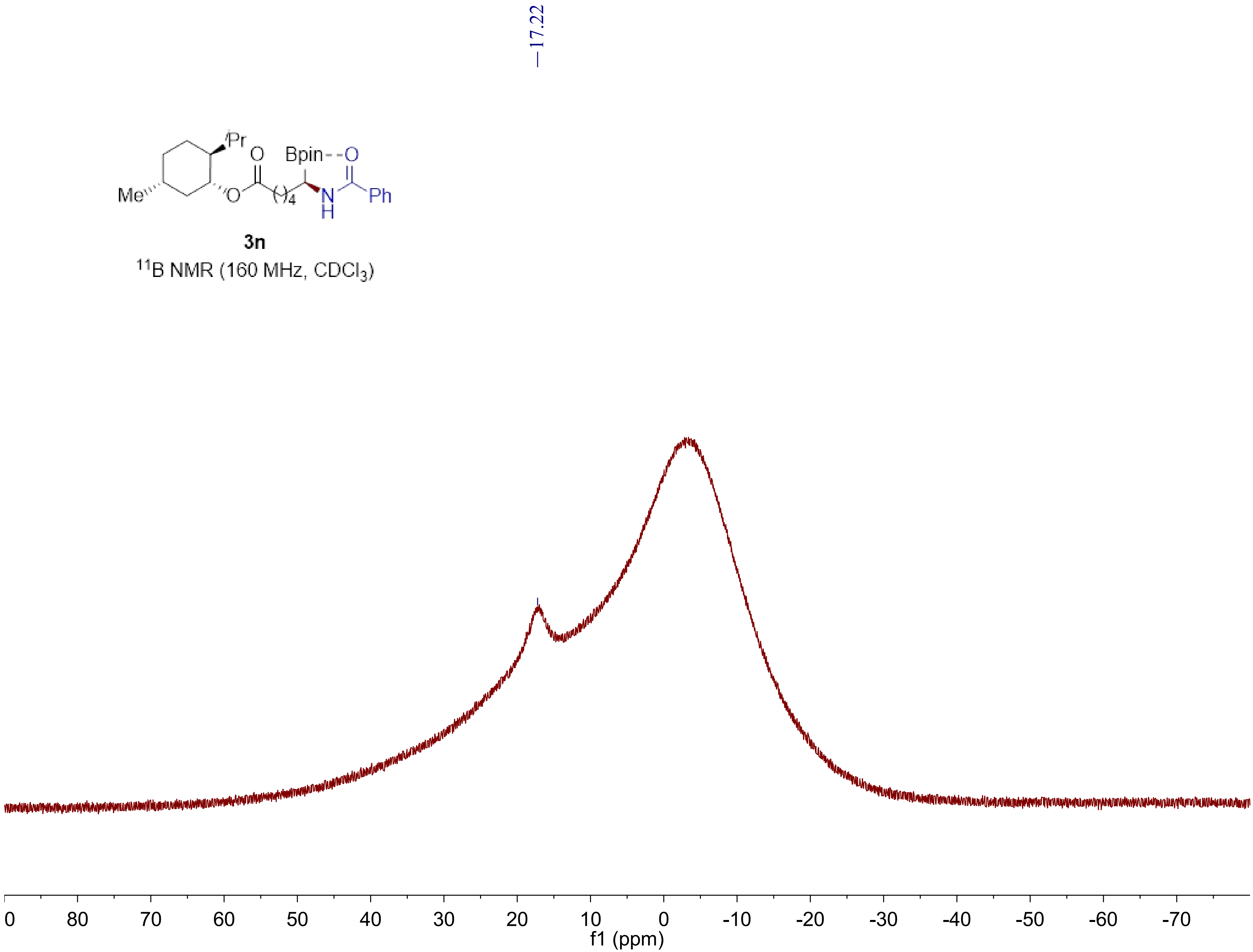

Supplementary Figure 43: ^{11}B NMR (160 MHz, $CDCl_3$) spectrum of **3n**.

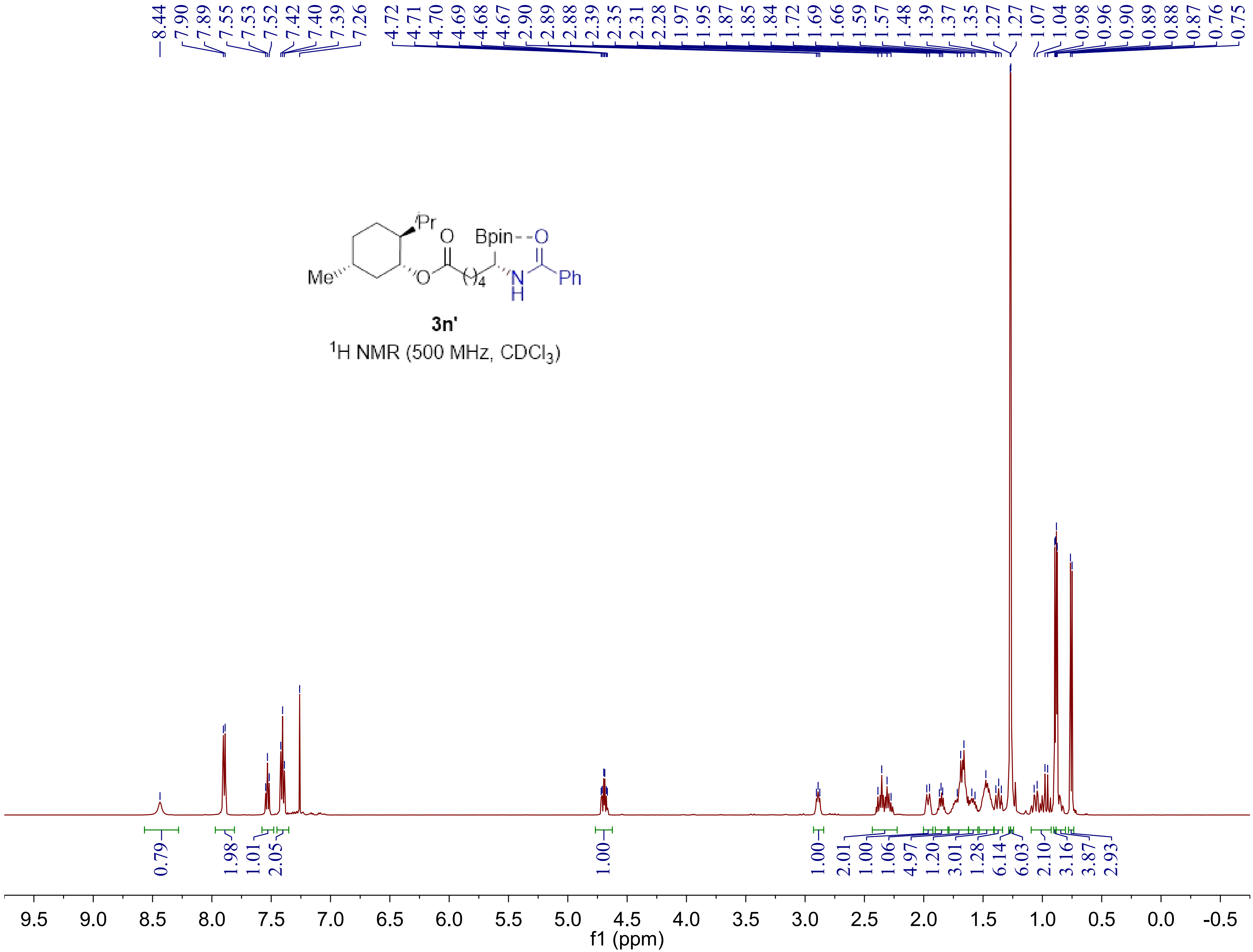

Supplementary Figure 44: ^{1}H NMR (500 MHz, $CDCl_3$) spectrum of **3n'**.

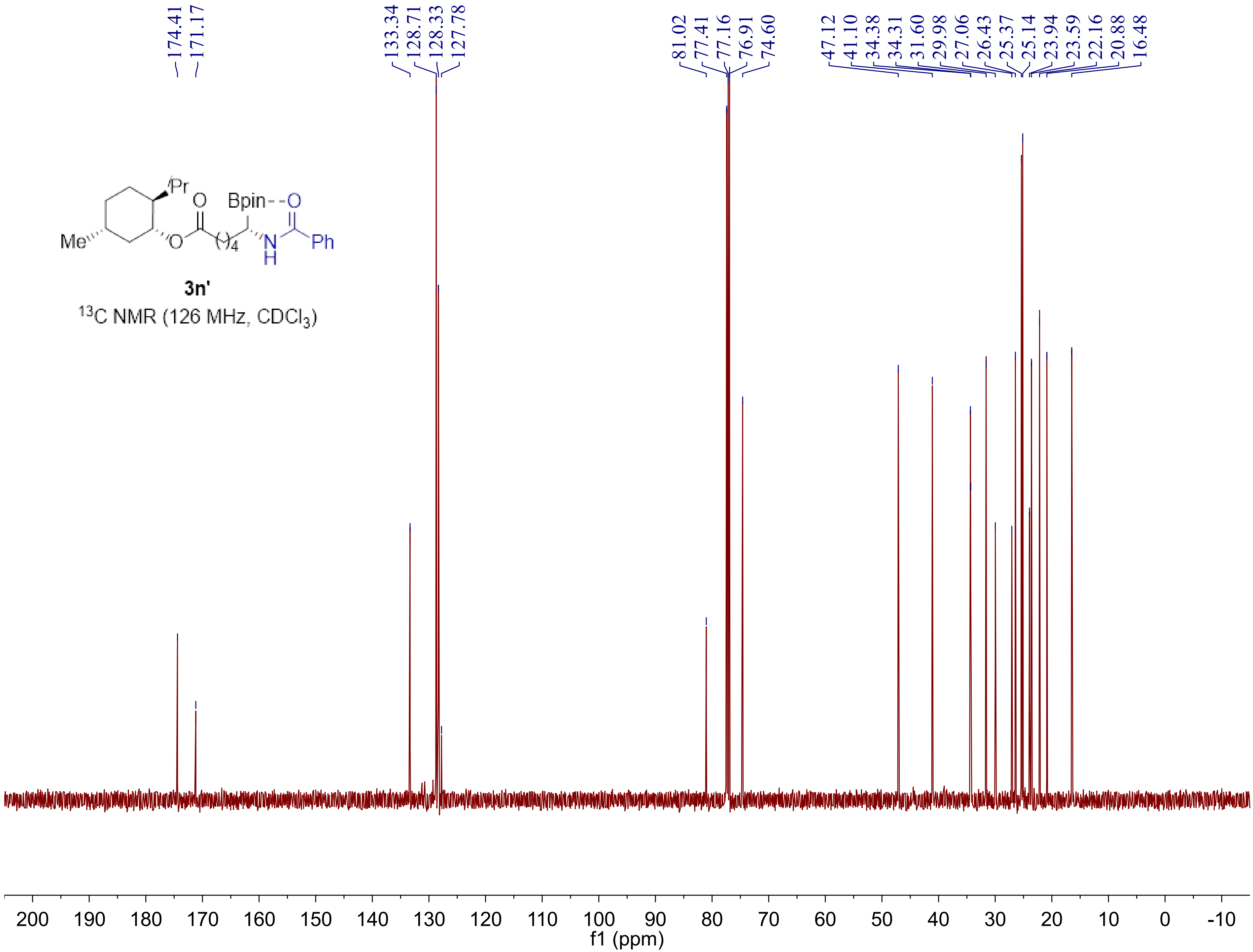

Supplementary Figure 45: ^{13}C NMR (126 MHz, $CDCl_3$) spectrum of **3n'**.

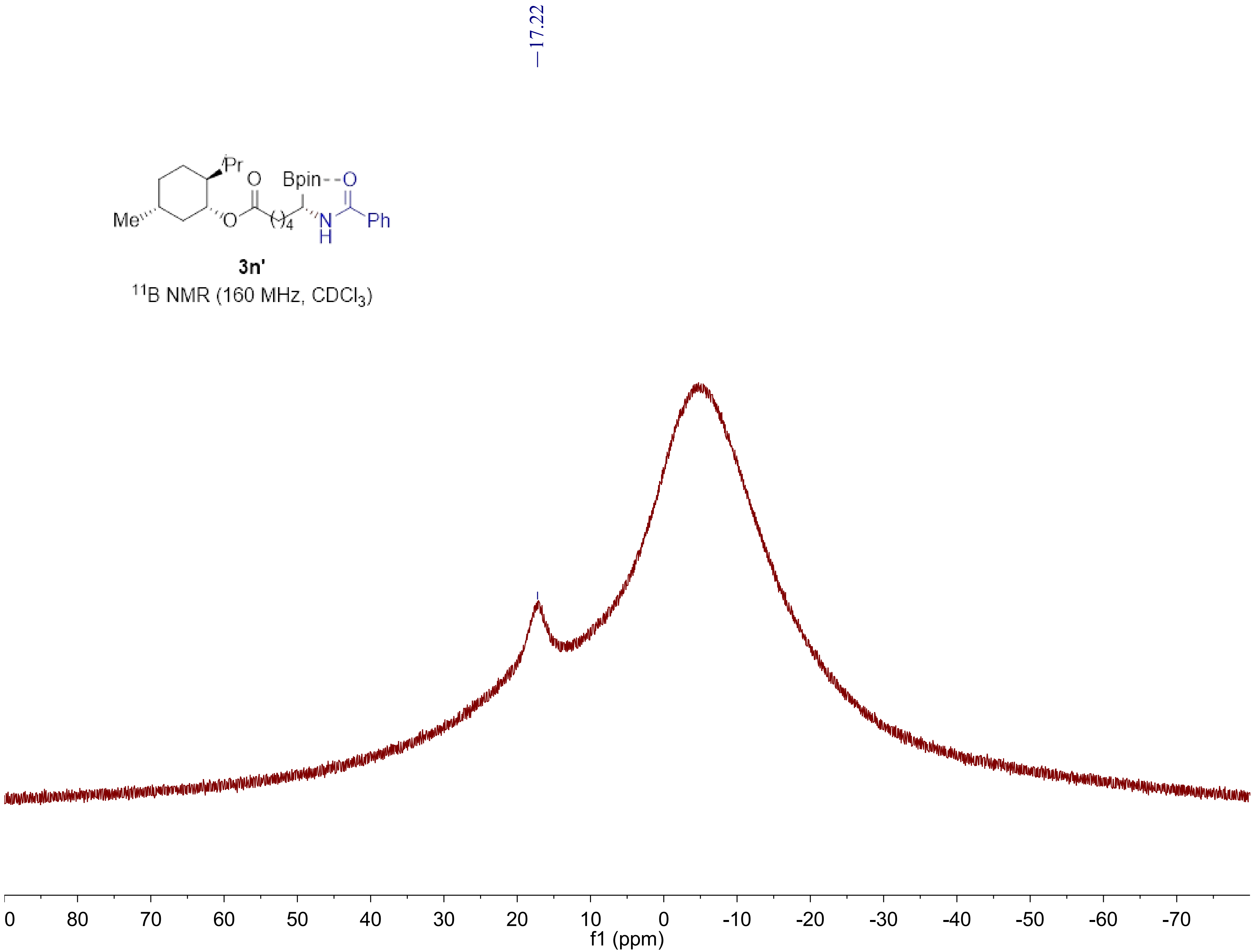

Supplementary Figure 46: ^{11}B NMR (160 MHz, $CDCl_3$) spectrum of **3n'**.

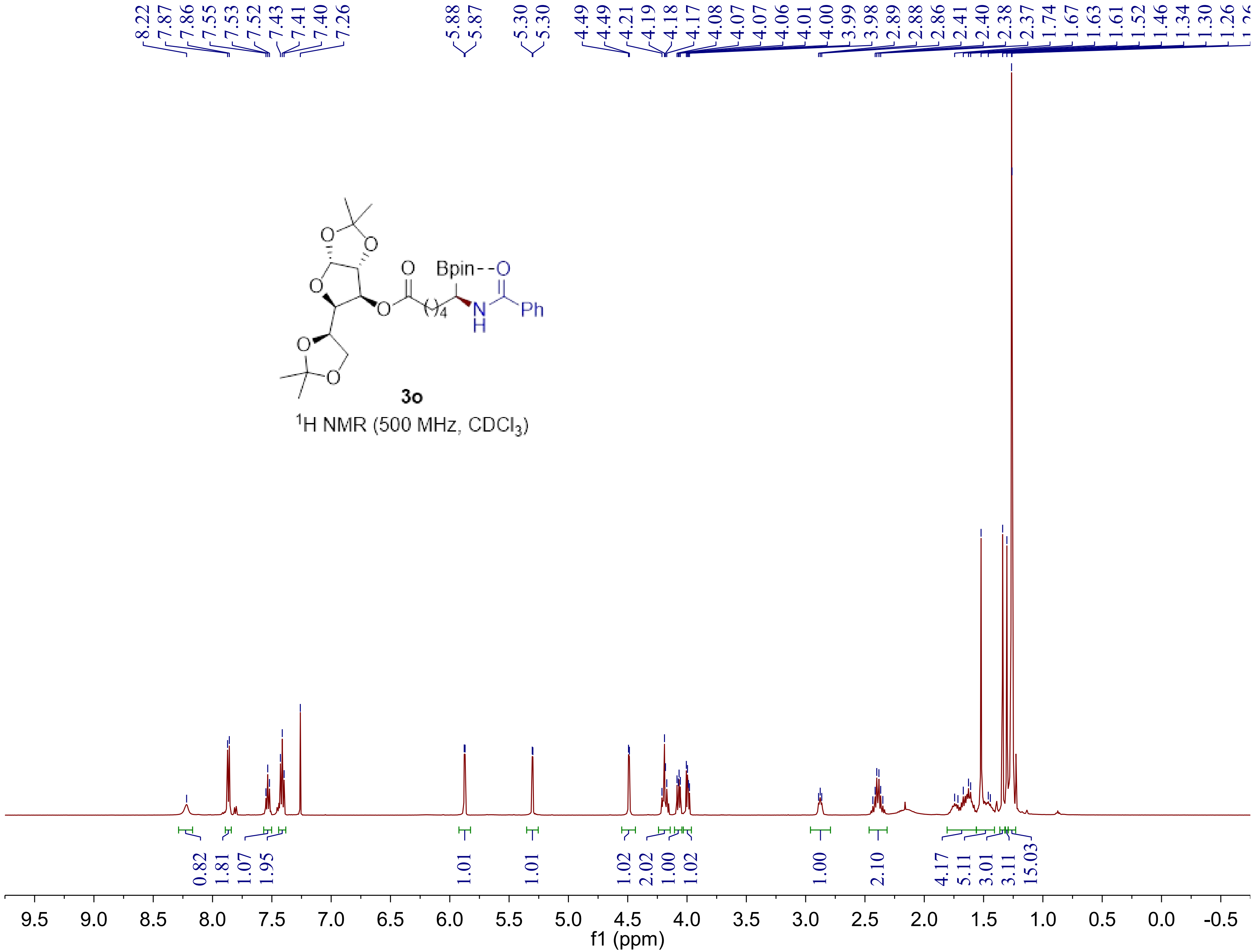

Supplementary Figure 47: ^{1}H NMR (500 MHz, $CDCl_3$) spectrum of **3o**.

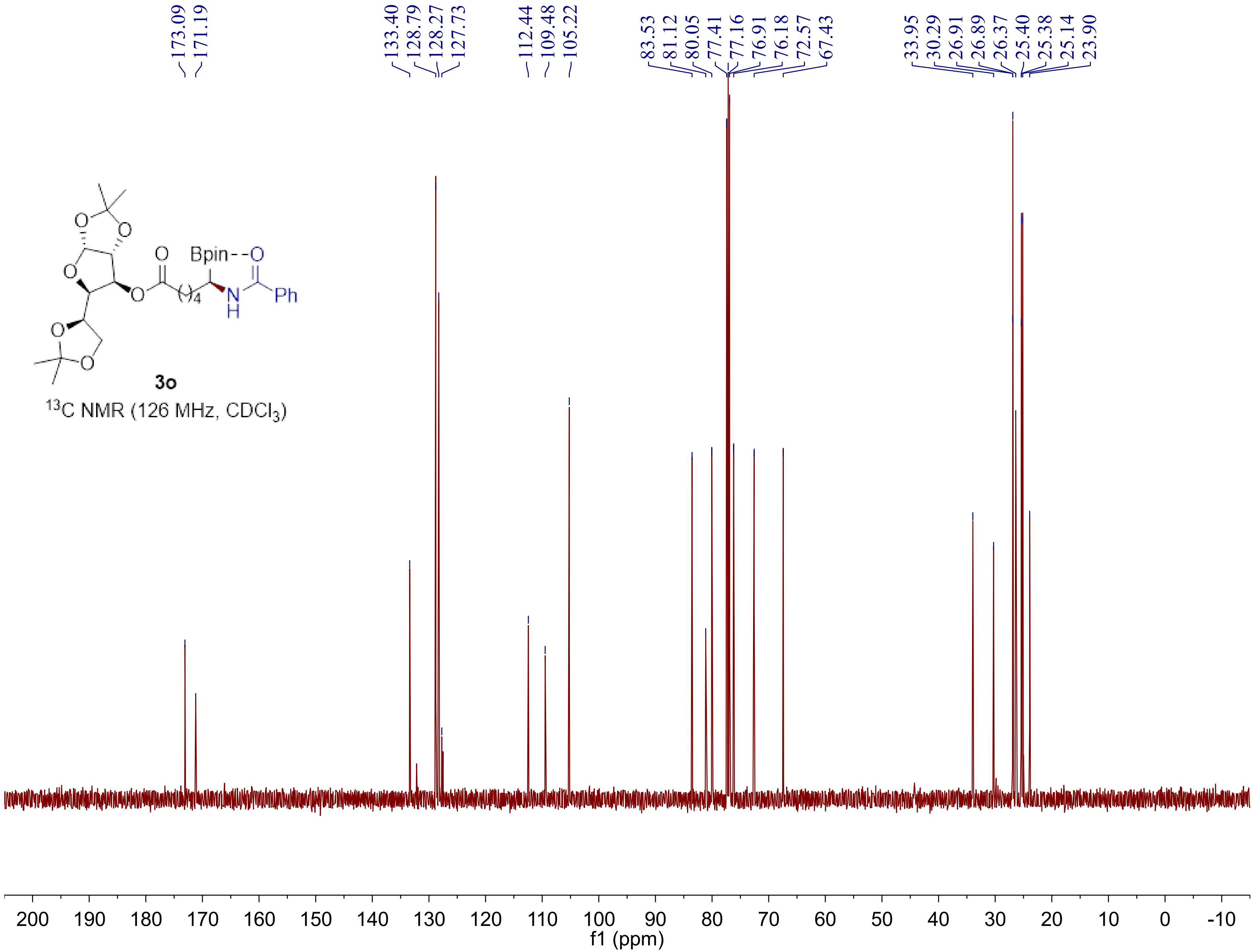

Supplementary Figure 48: ^{13}C NMR (126 MHz, $CDCl_3$) spectrum of **3o**.

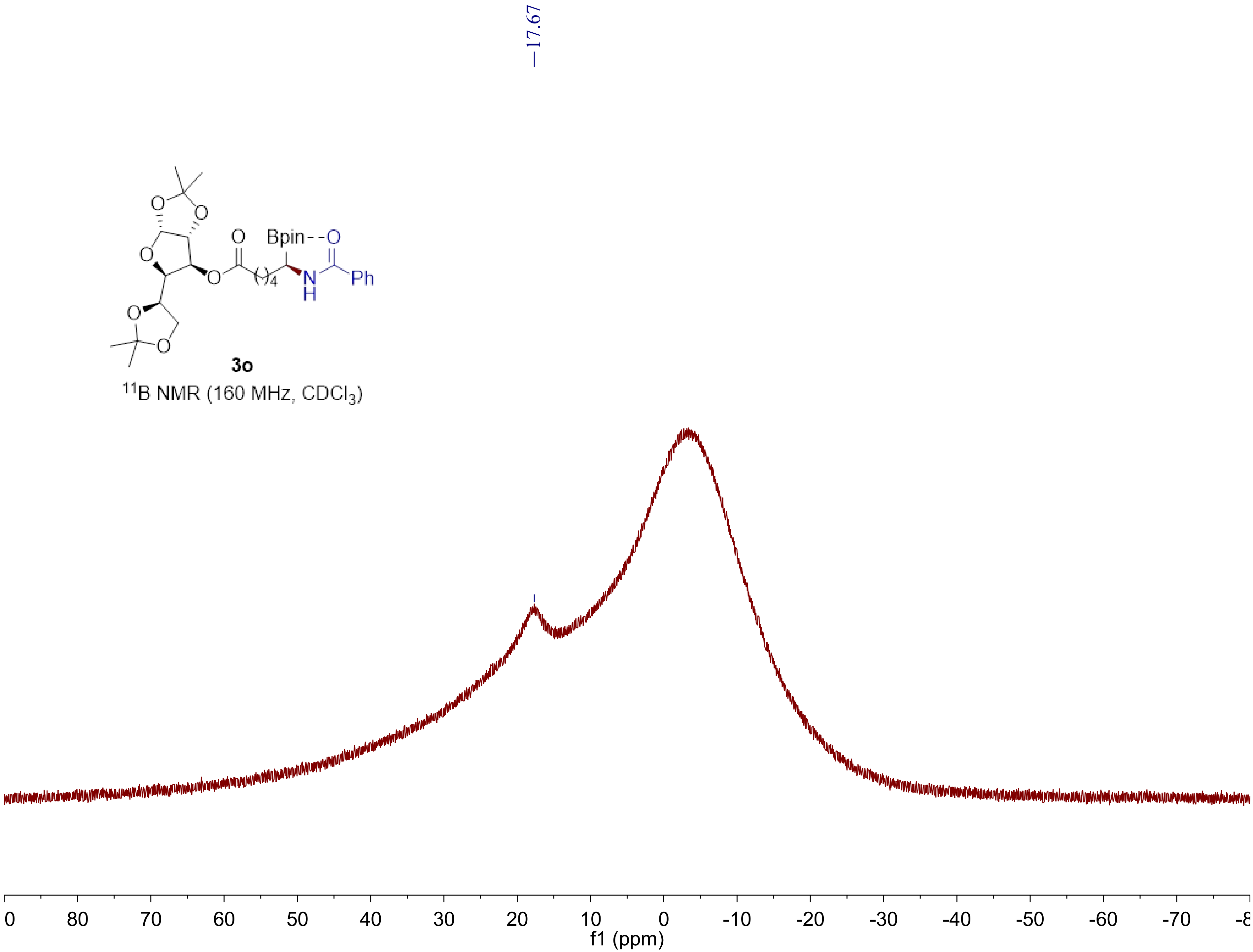

Supplementary Figure 49: ^{11}B NMR (160 MHz, CDCl$_3$) spectrum of **3o**.

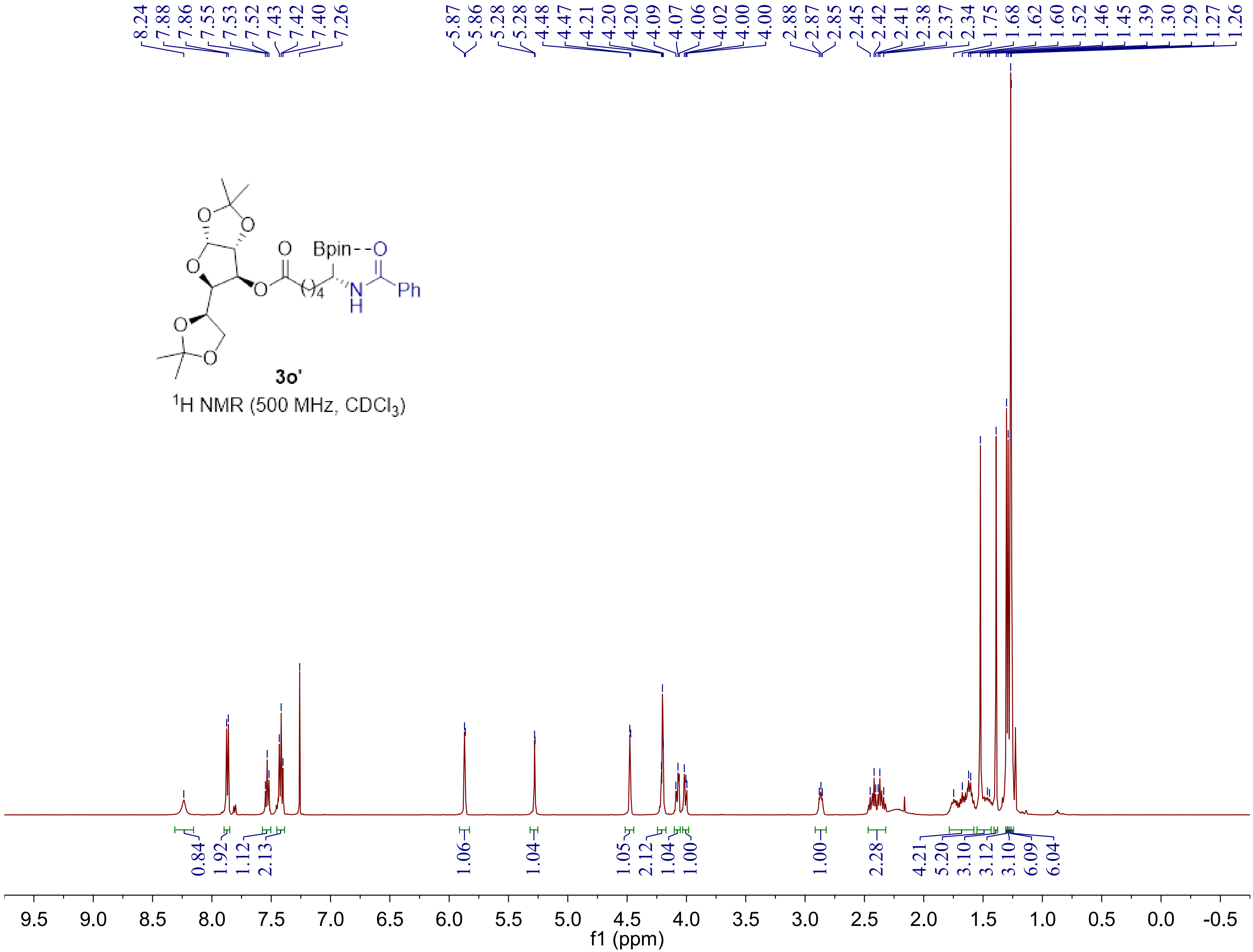

Supplementary Figure 50: ^{1}H NMR (500 MHz, $CDCl_3$) spectrum of **3o'**.

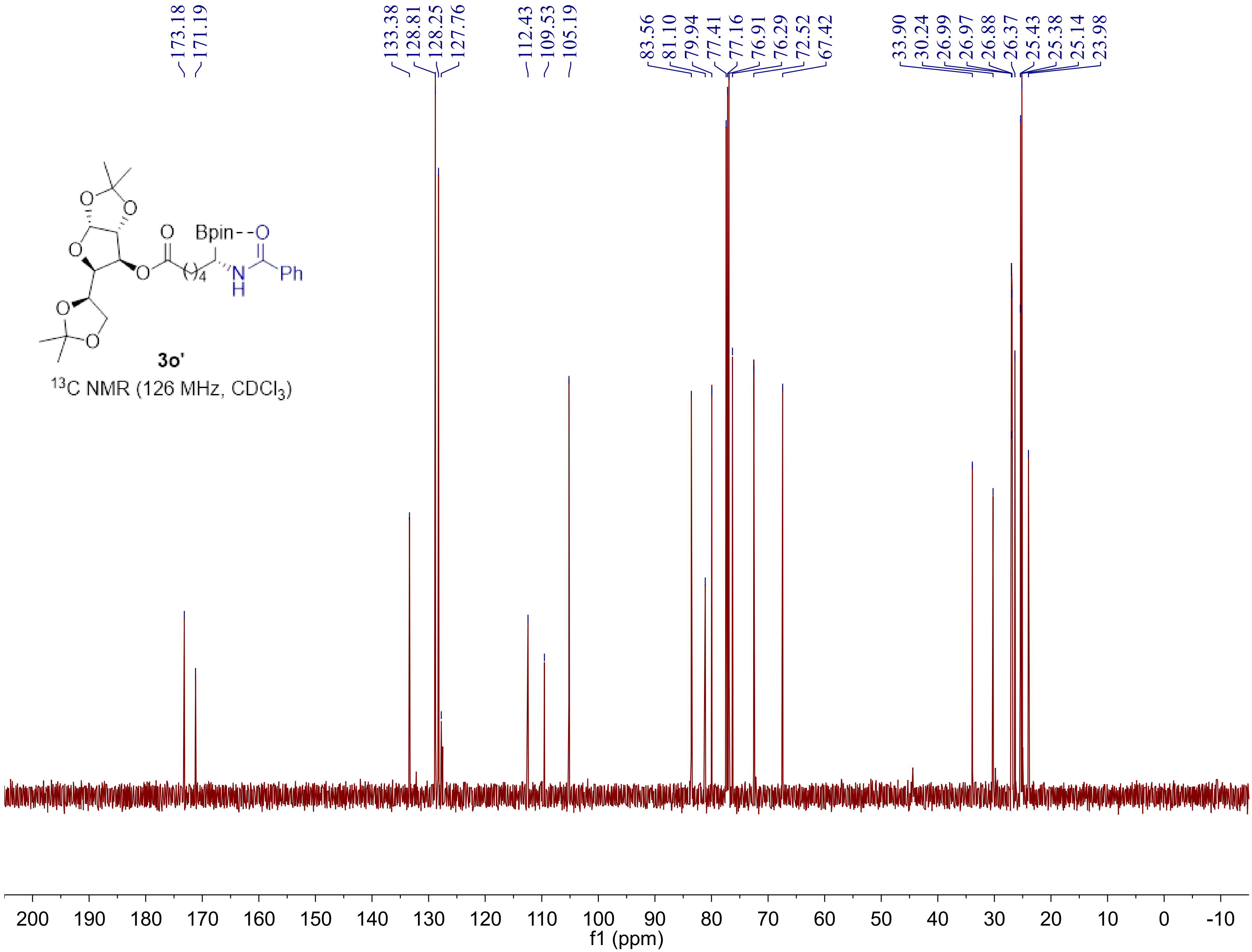

Supplementary Figure 51: ^{13}C NMR (126 MHz, $CDCl_3$) spectrum of **3o'**.

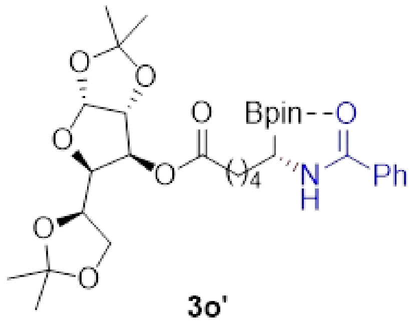

3o'

^{11}B NMR (160 MHz, $CDCl_3$)

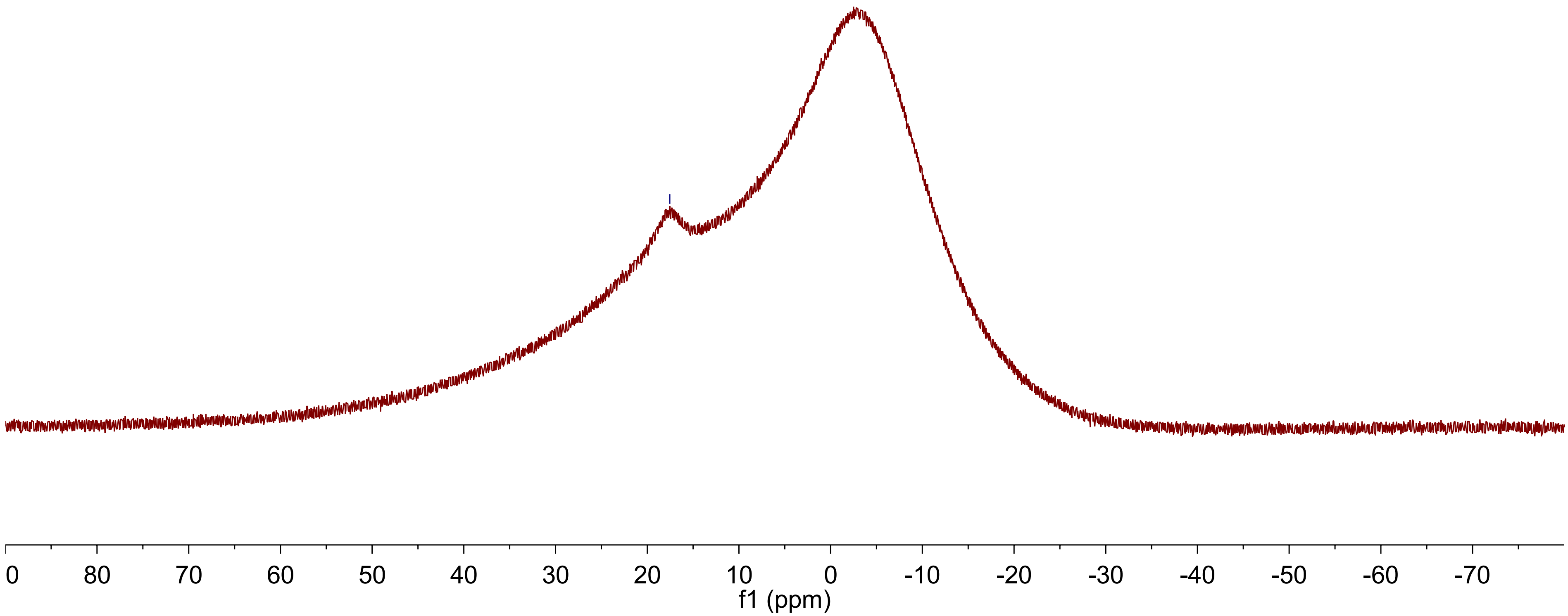

Supplementary Figure 52: ^{11}B NMR (160 MHz, $CDCl_3$) spectrum of **3o'**.

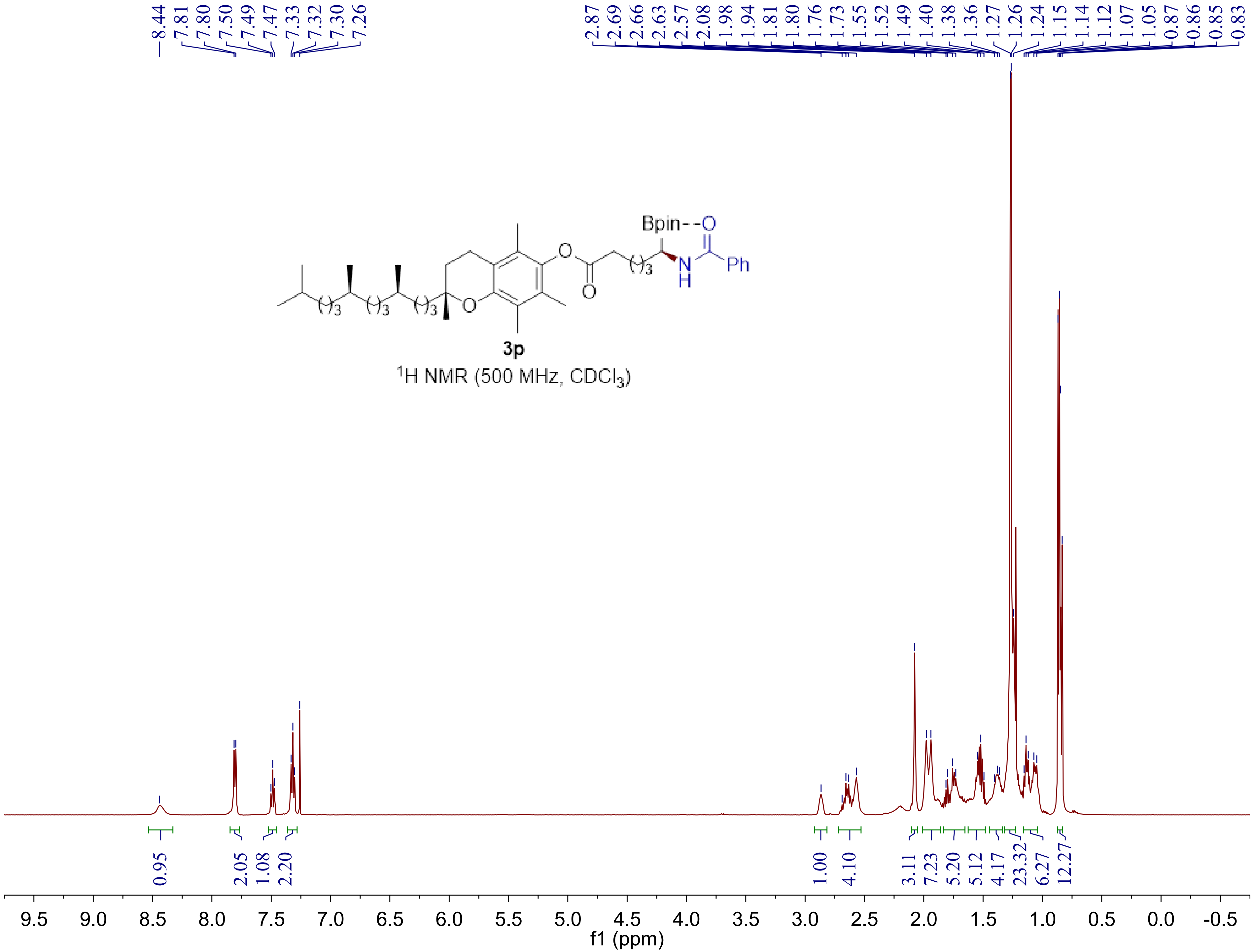

Supplementary Figure 53: ^{1}H NMR (500 MHz, $CDCl_3$) spectrum of **3p**.

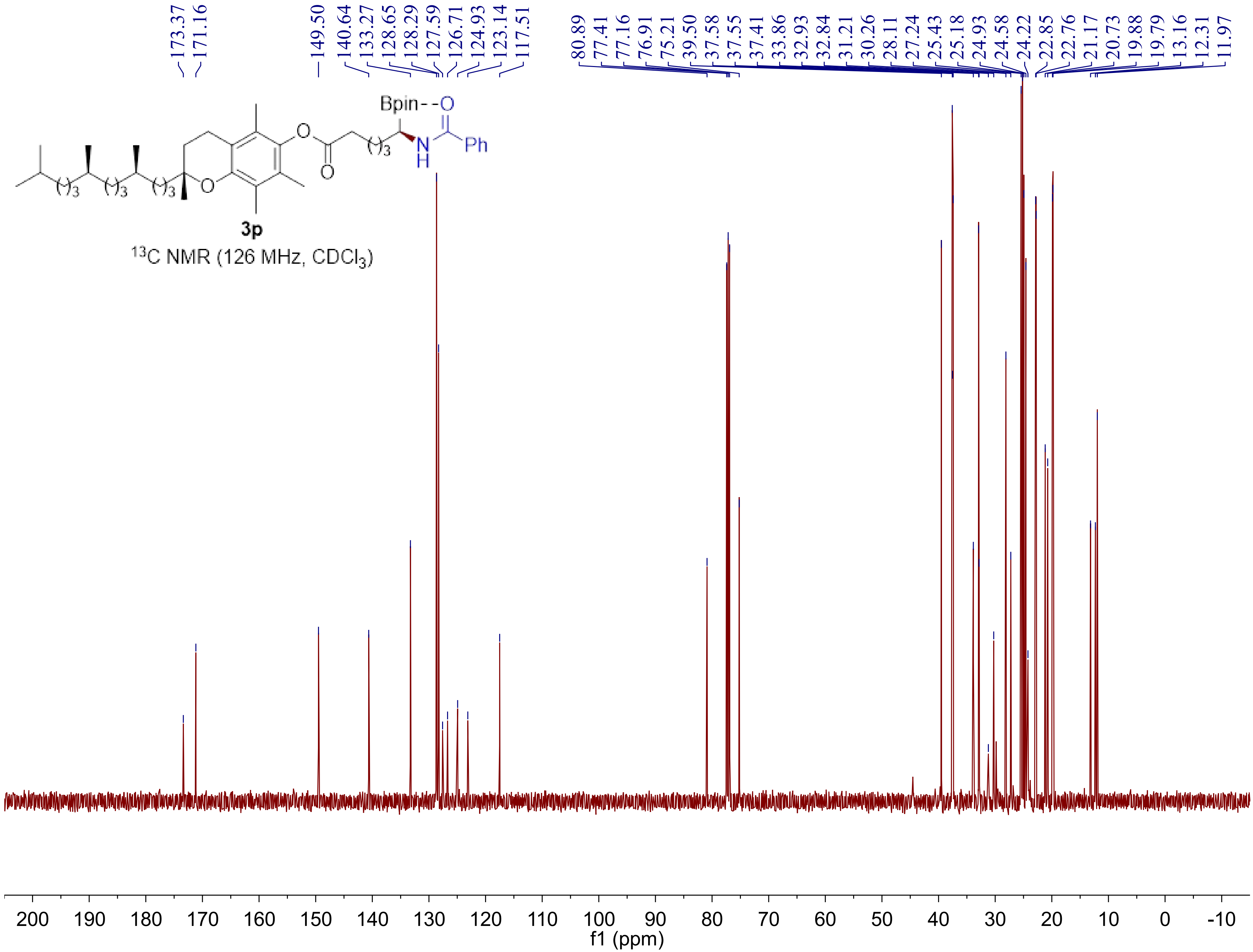

Supplementary Figure 54: ^{13}C NMR (126 MHz, CDCl$_3$) spectrum of **3p**.

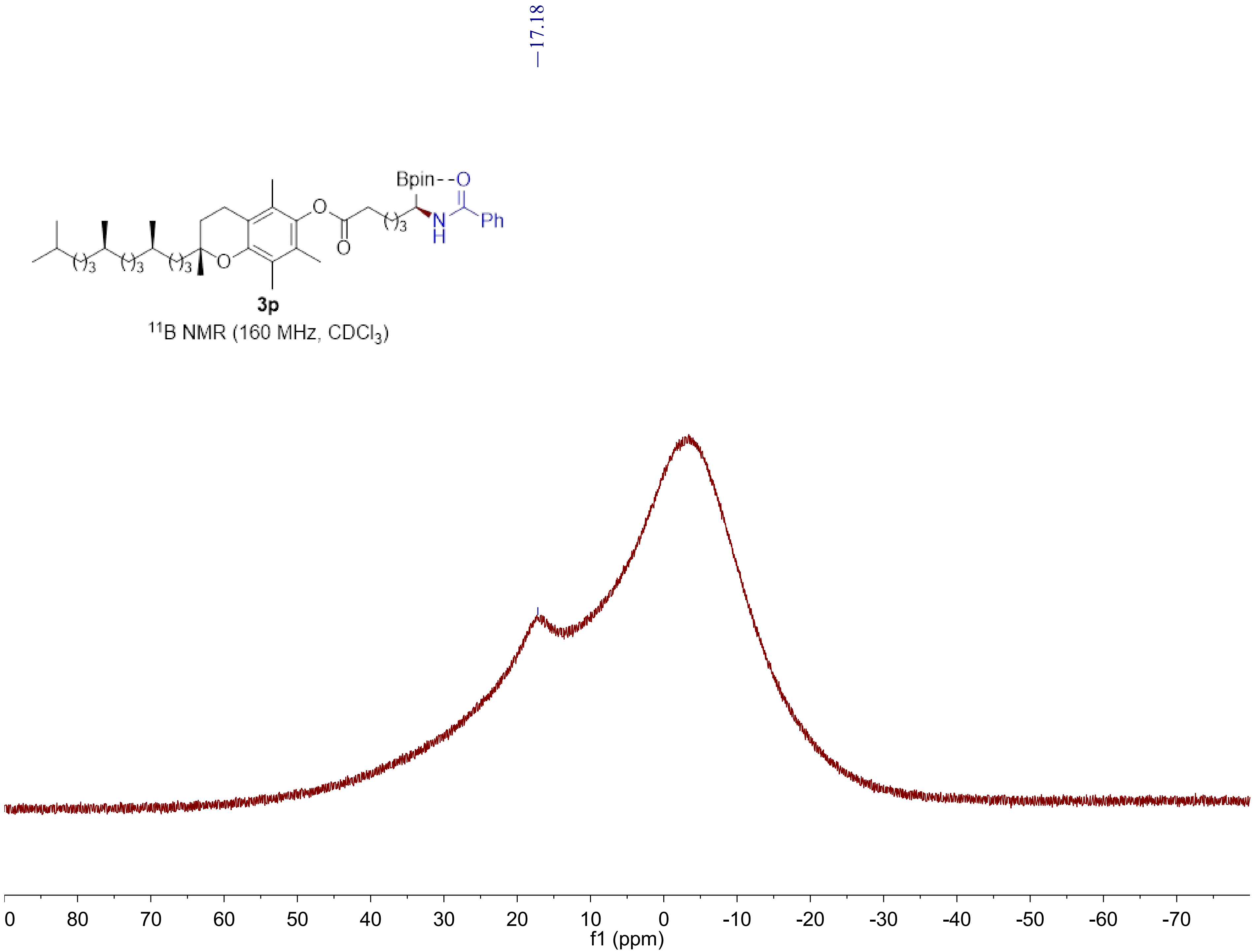

Supplementary Figure 55: ^{11}B NMR (160 MHz, $CDCl_3$) spectrum of **3p**.

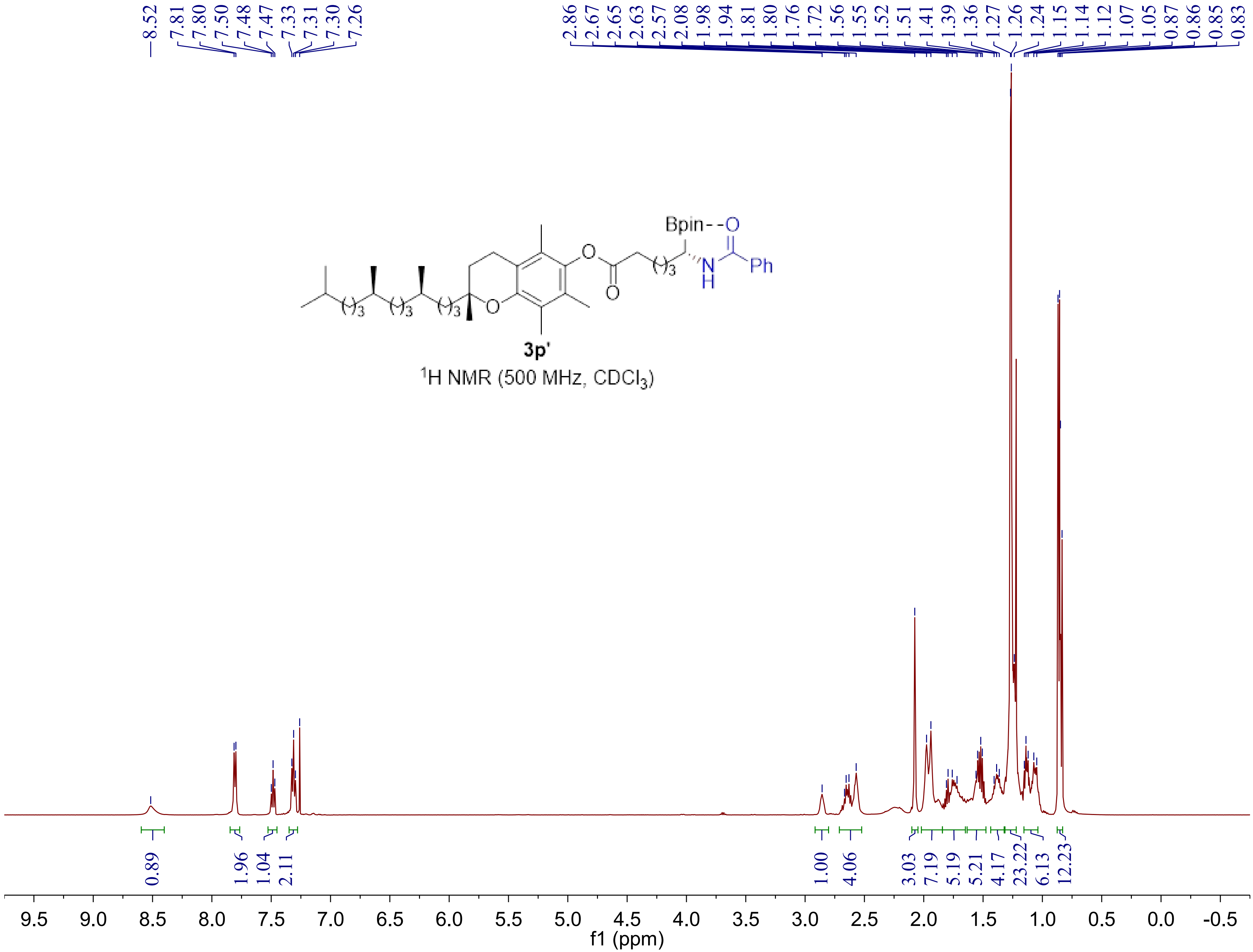

Supplementary Figure 56: ^{1}H NMR (500 MHz, $CDCl_3$) spectrum of **3p'**.

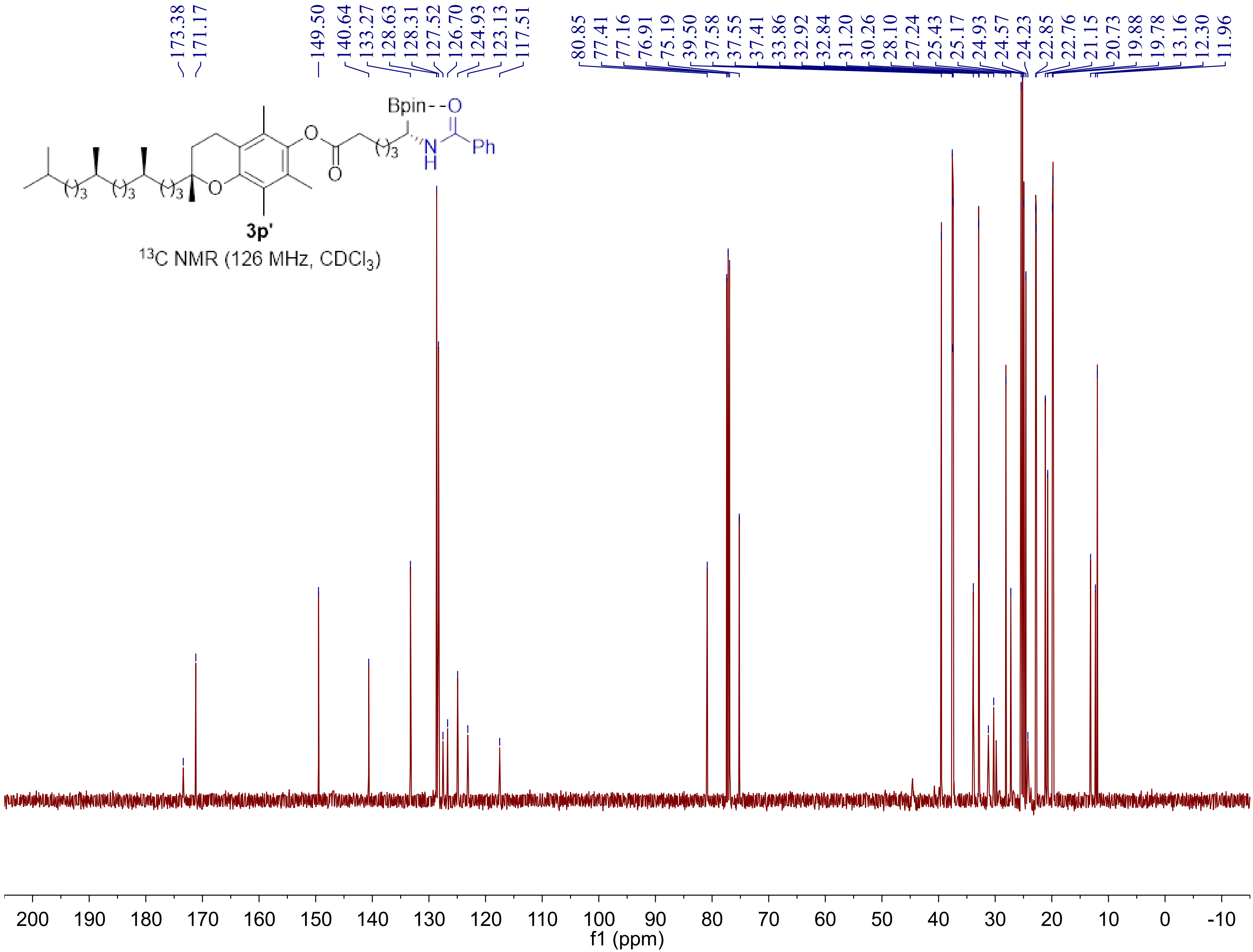

Supplementary Figure 57: ^{13}C NMR (126 MHz, CDCl$_3$) spectrum of **3p'**.

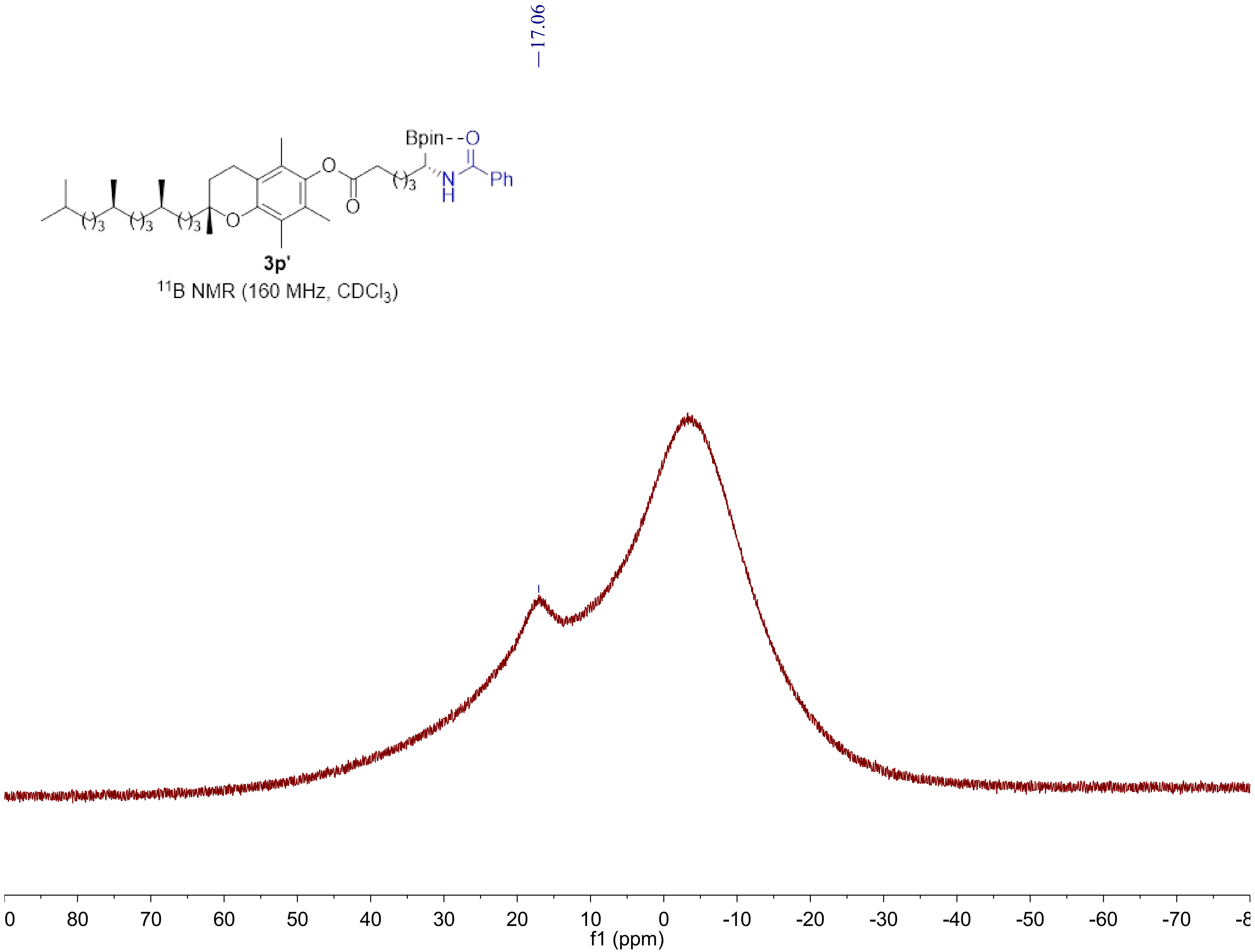

Supplementary Figure 58: ^{11}B NMR (160 MHz, $CDCl_3$) spectrum of **3p'**.

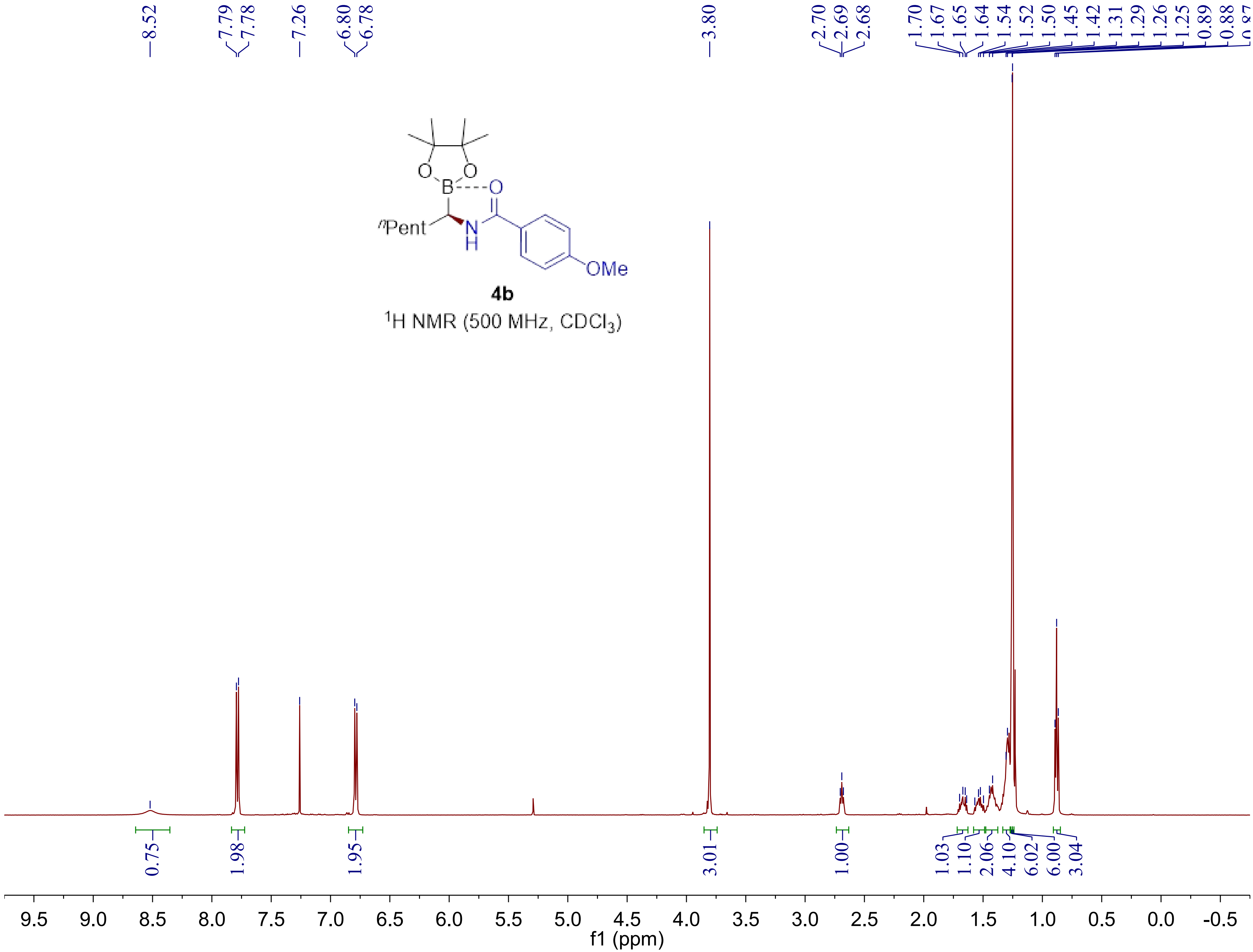

Supplementary Figure 59: ^{1}H NMR (500 MHz, $CDCl_3$) spectrum of **4b**.

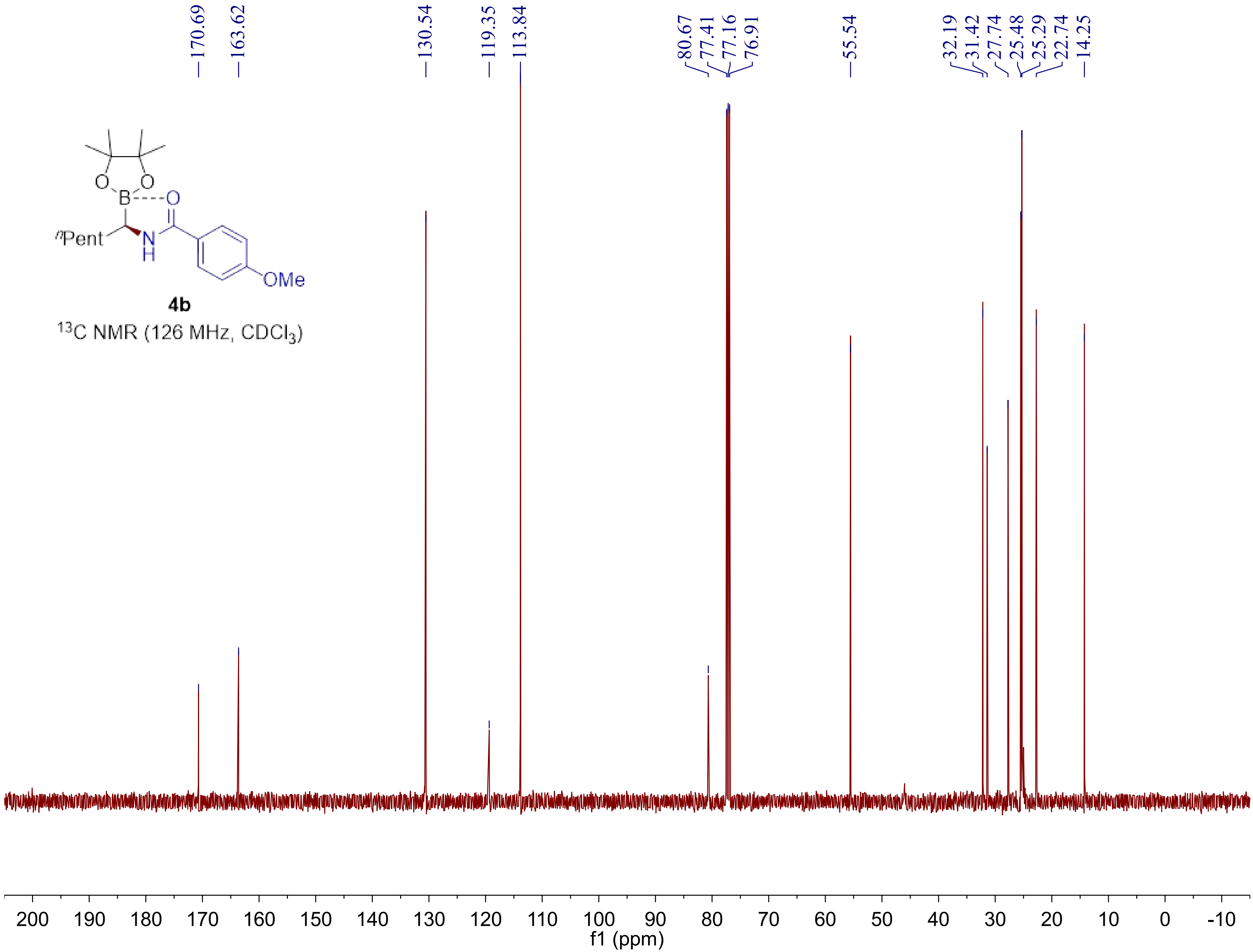

Supplementary Figure 60: ^{13}C NMR (126 MHz, $CDCl_3$) spectrum of **4b**.

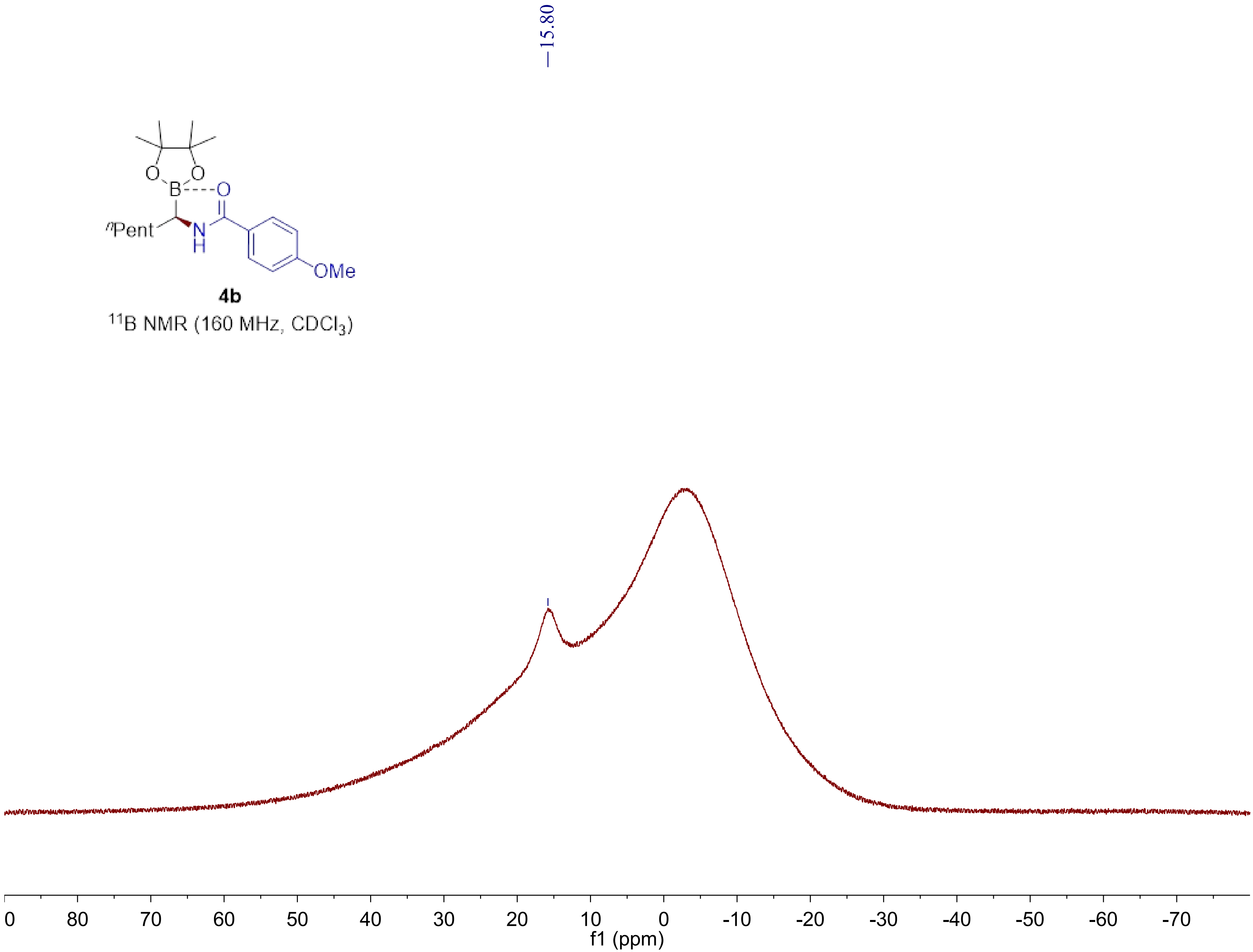

Supplementary Figure 61: ^{11}B NMR (160 MHz, CDCl$_3$) spectrum of **4b**.

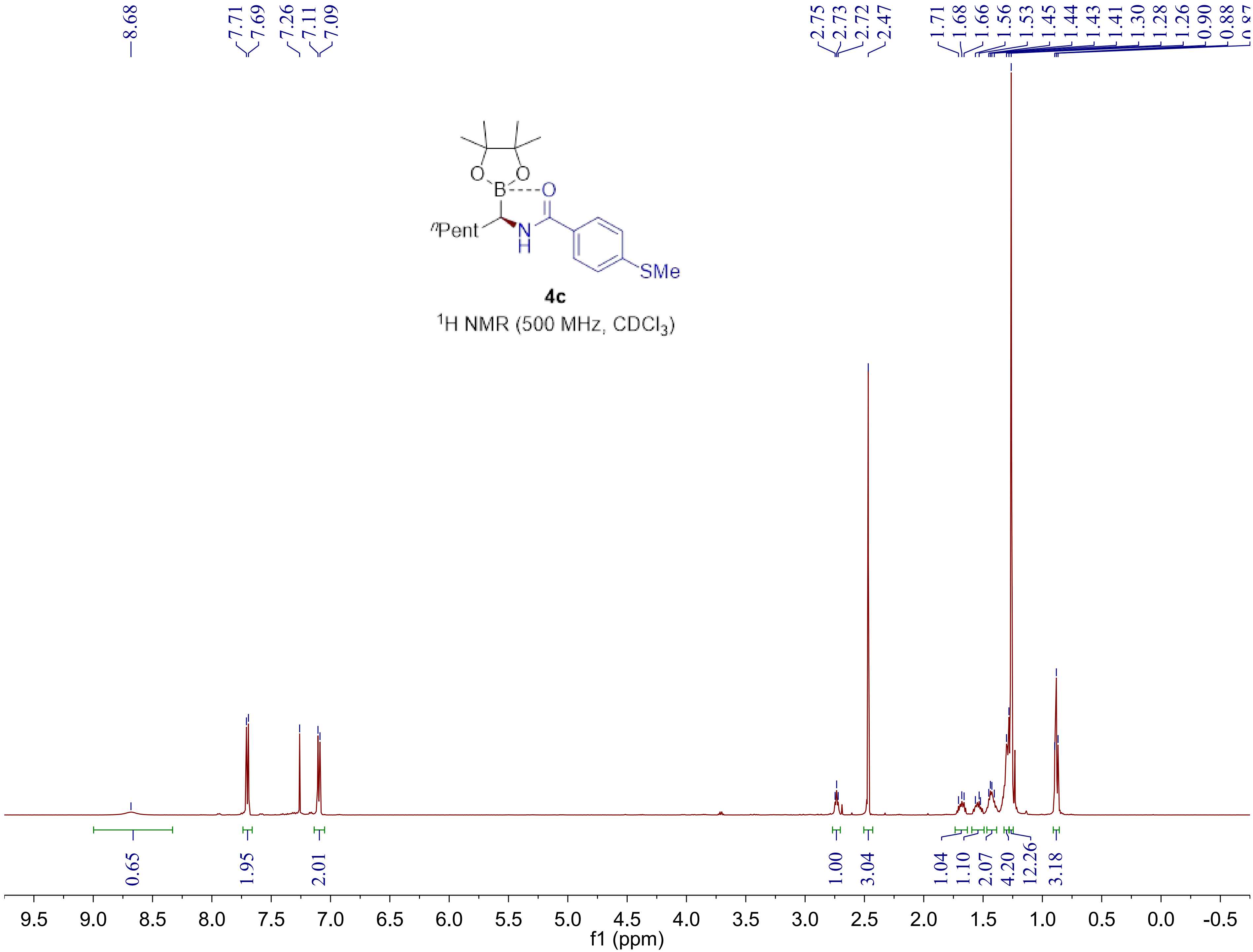

Supplementary Figure 62: ^{1}H NMR (500 MHz, $CDCl_3$) spectrum of **4c**.

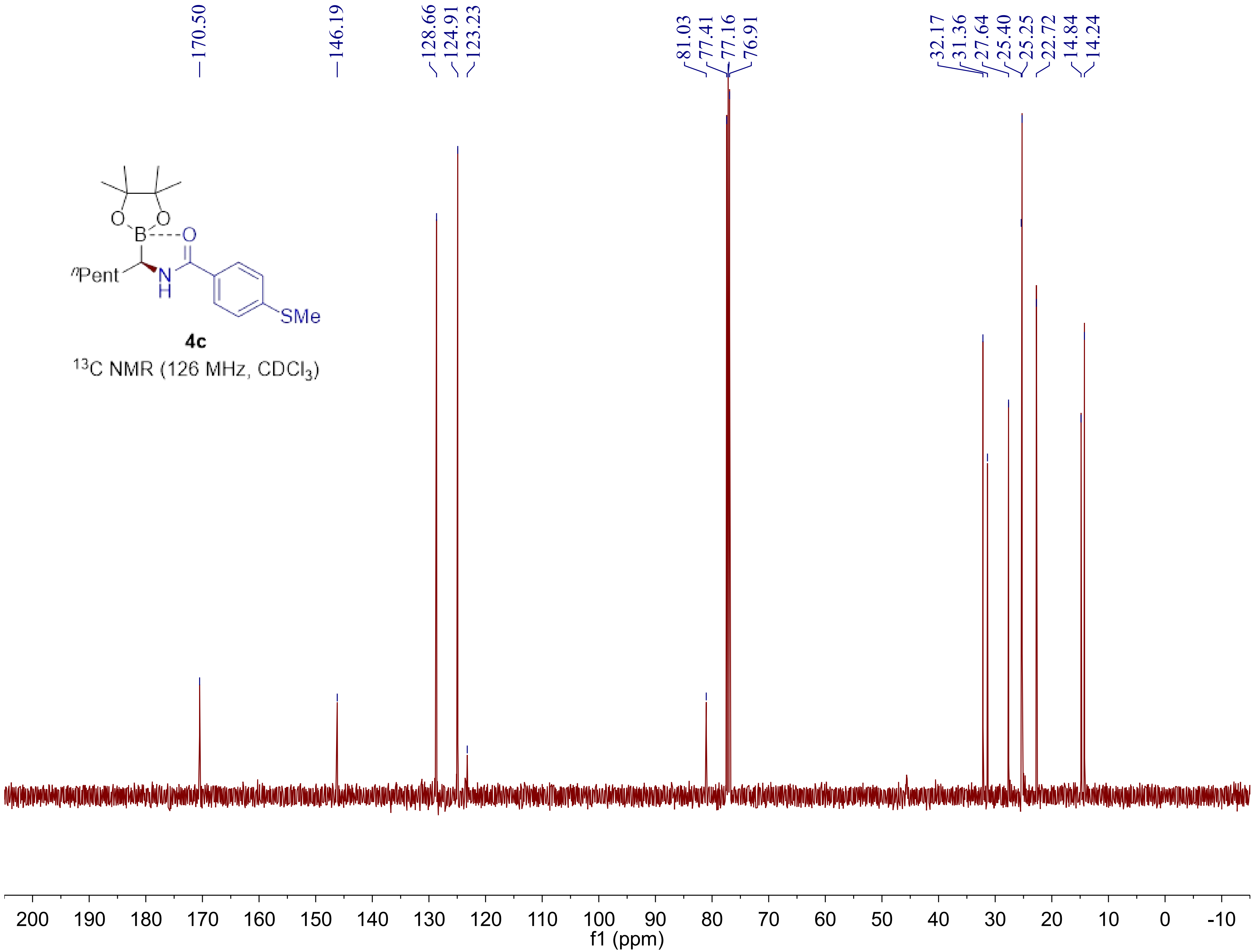

Supplementary Figure 63: ^{13}C NMR (126 MHz, $CDCl_3$) spectrum of **4c**.

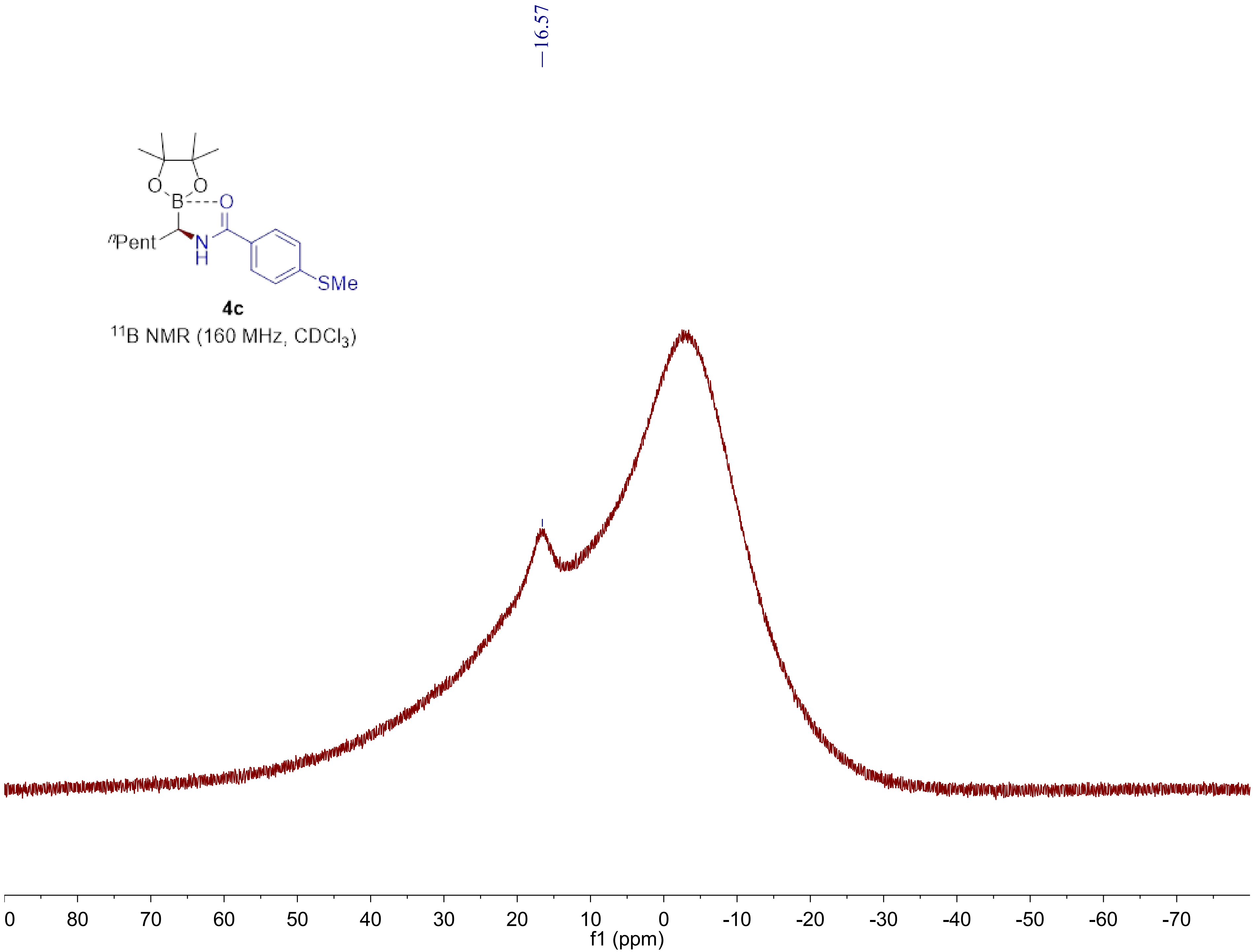

Supplementary Figure 64: ^{11}B NMR (160 MHz, $CDCl_3$) spectrum of **4c**.

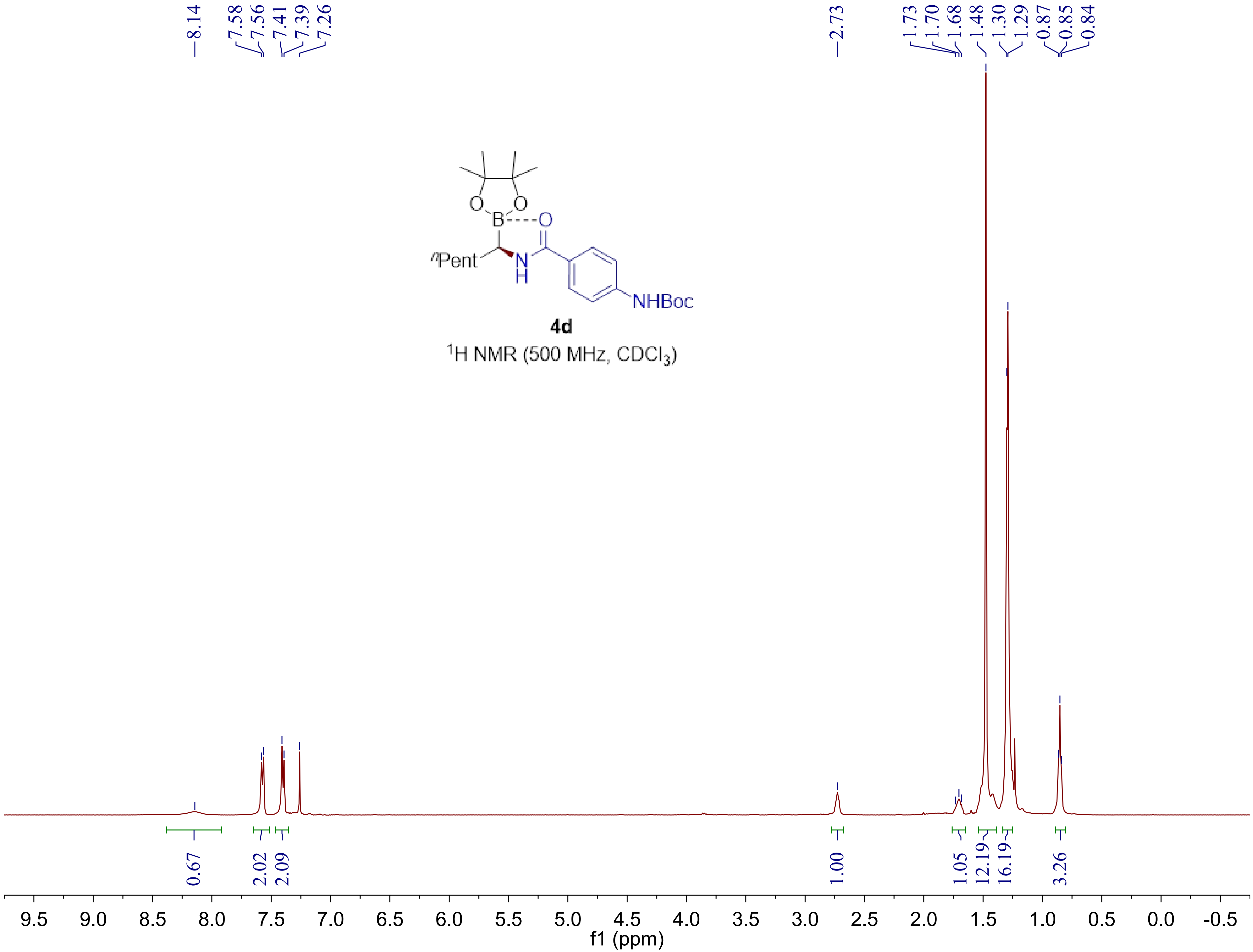

Supplementary Figure 65: ^{1}H NMR (500 MHz, $CDCl_3$) spectrum of **4d**.

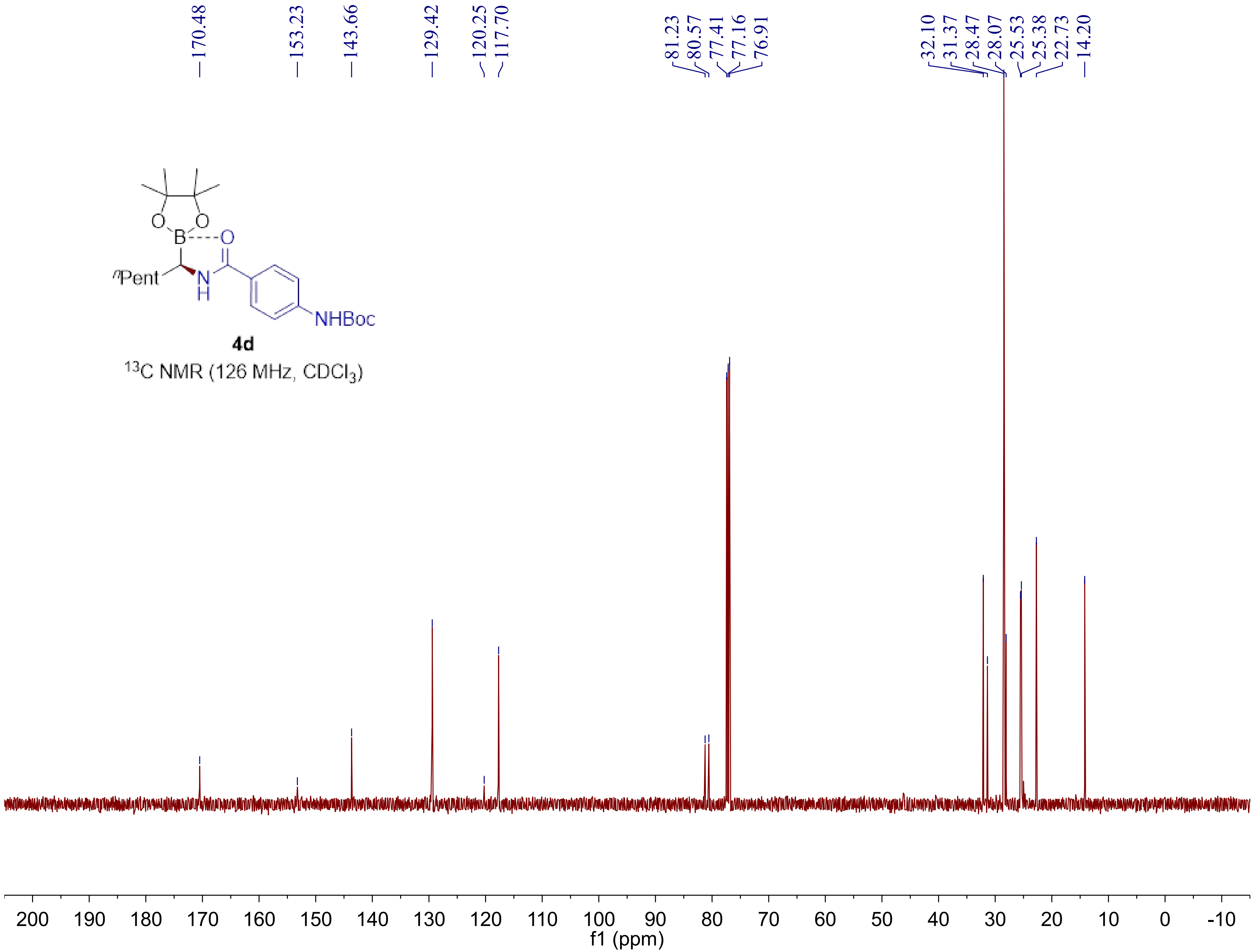

Supplementary Figure 66: ^{13}C NMR (126 MHz, $CDCl_3$) spectrum of **4d**.

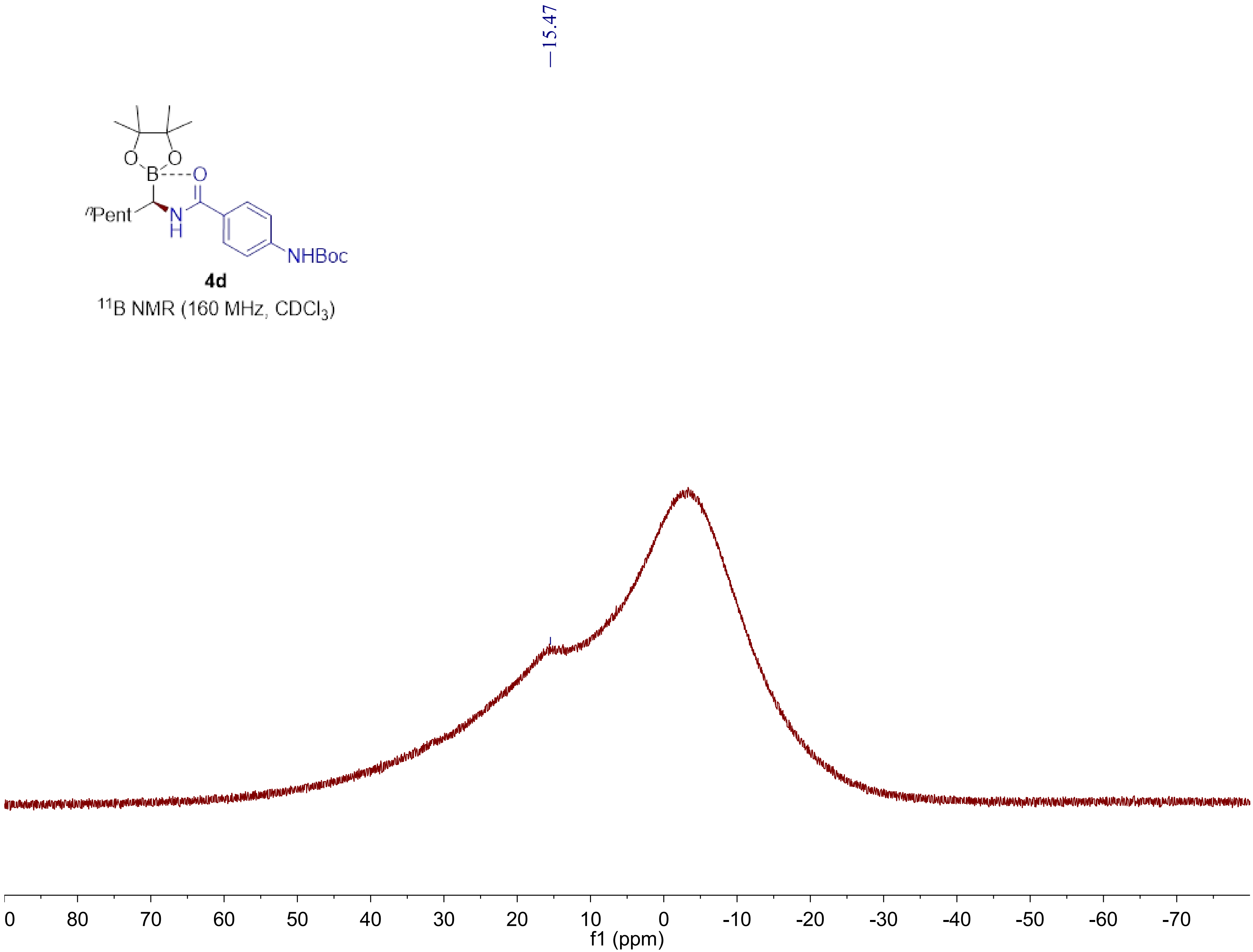

Supplementary Figure 67: ^{11}B NMR (160 MHz, $CDCl_3$) spectrum of **4d**.

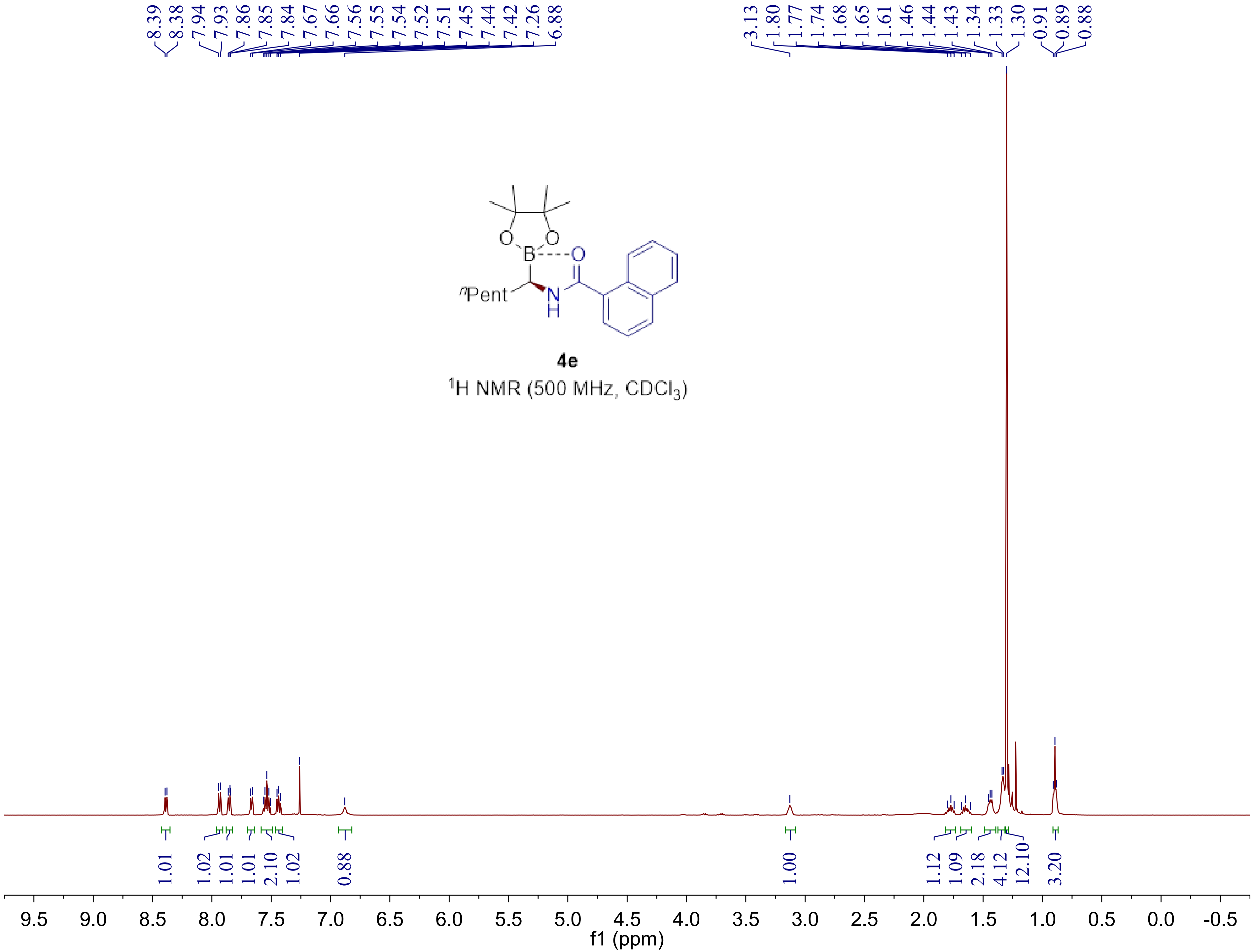

Supplementary Figure 68: ^{1}H NMR (500 MHz, $CDCl_3$) spectrum of **4e**.

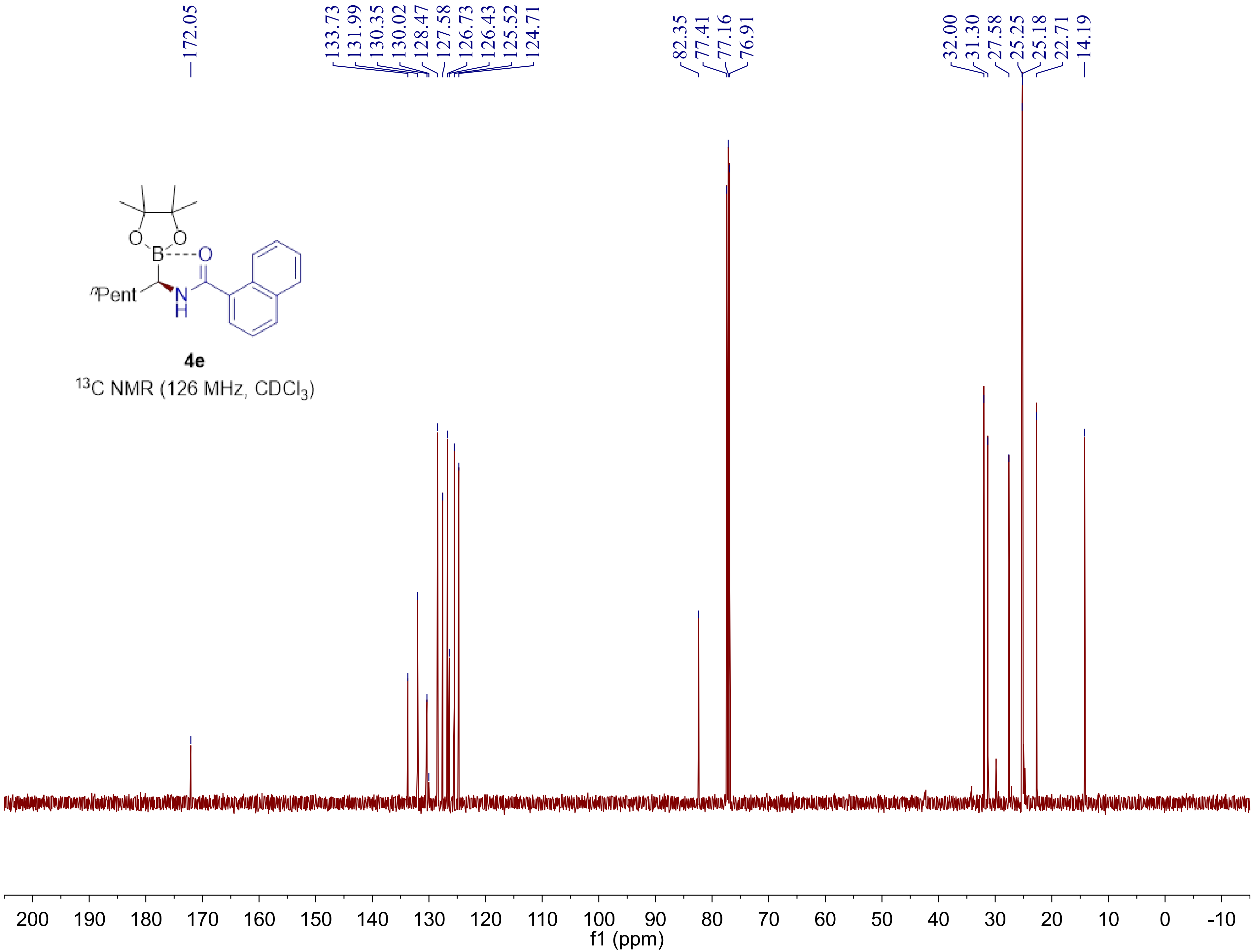

Supplementary Figure 69: ^{13}C NMR (126 MHz, $CDCl_3$) spectrum of **4e**.

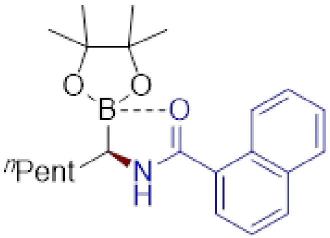

4e

^{11}B NMR (160 MHz, $CDCl_3$)

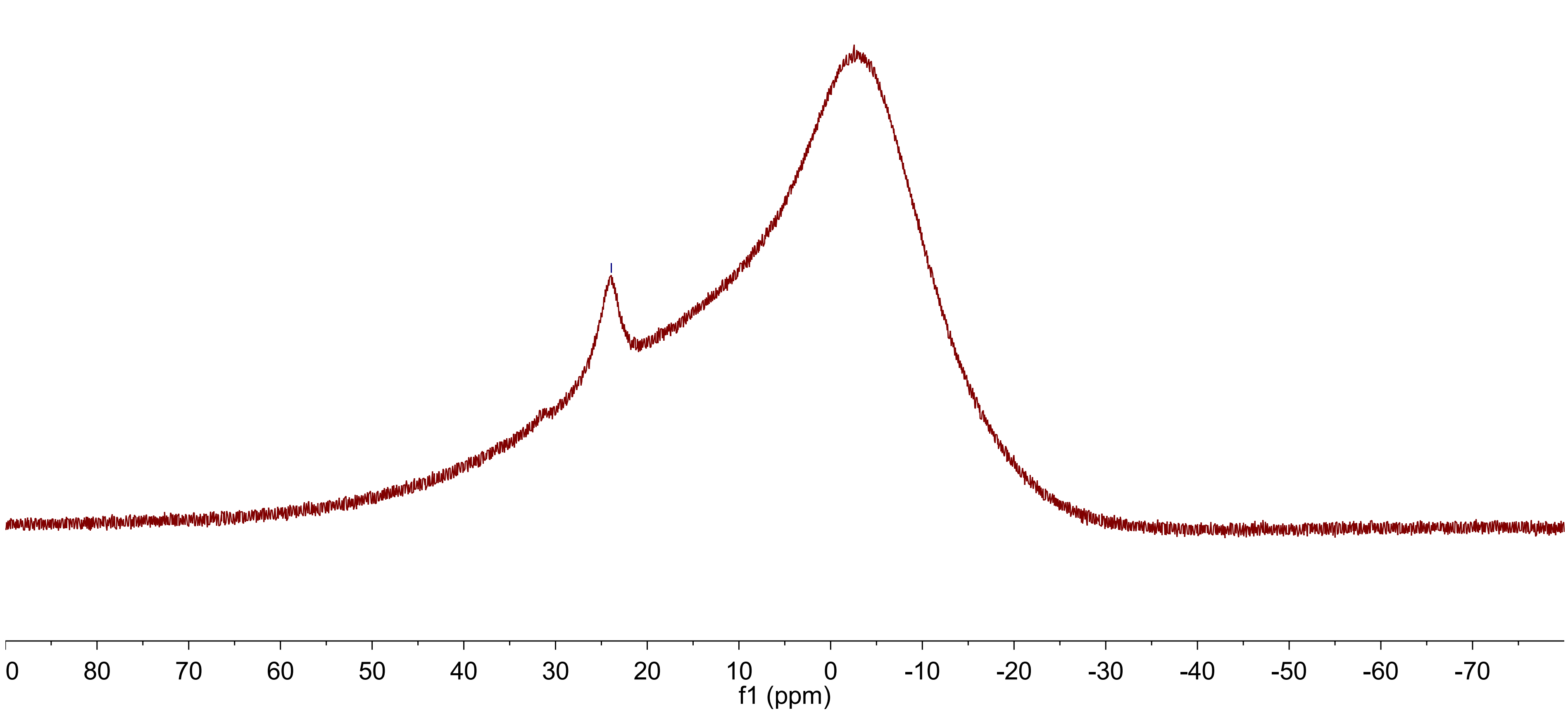

Supplementary Figure 70: ^{11}B NMR (160 MHz, $CDCl_3$) spectrum of **4e**.

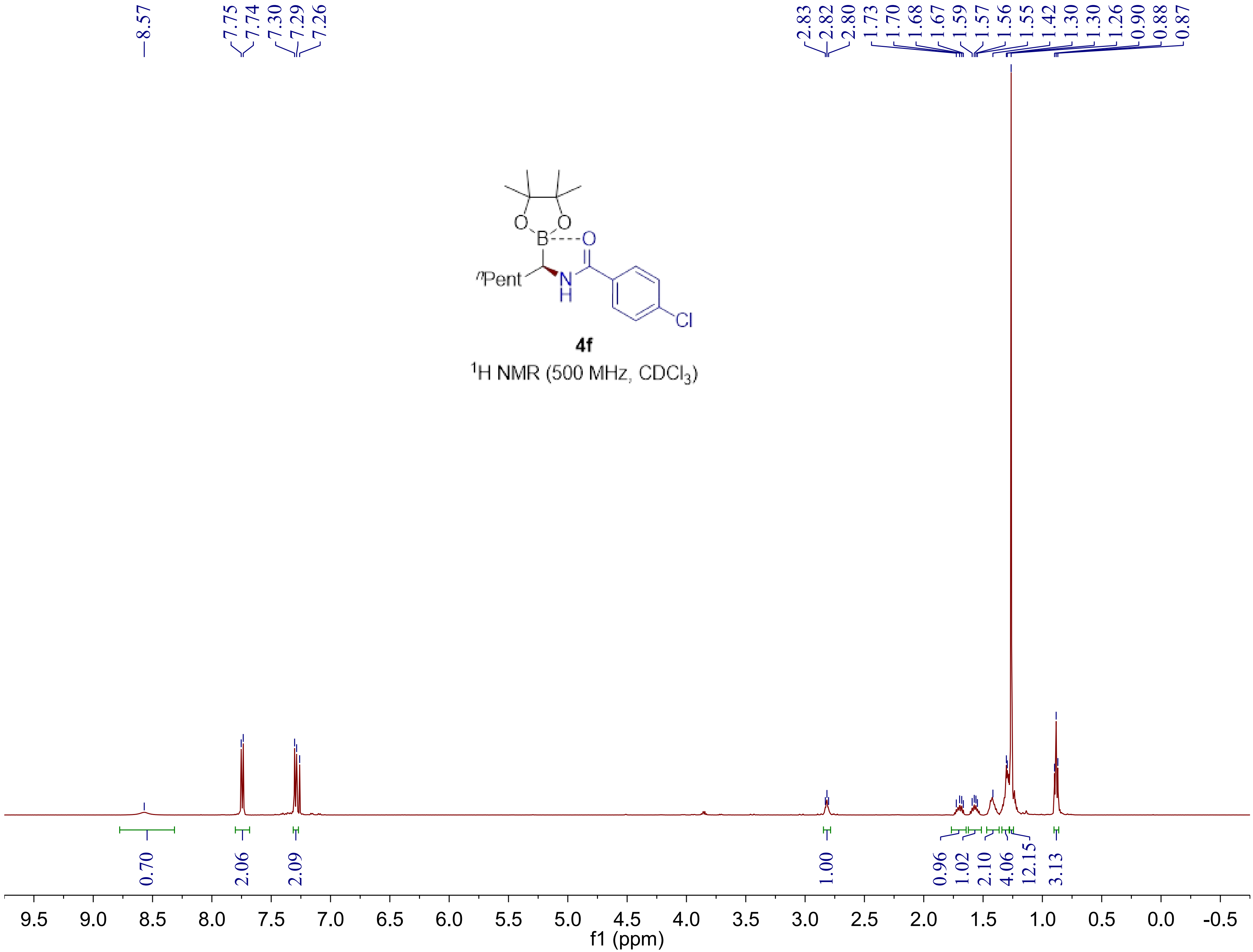

Supplementary Figure 71: ^{1}H NMR (500 MHz, $CDCl_3$) spectrum of **4f**.

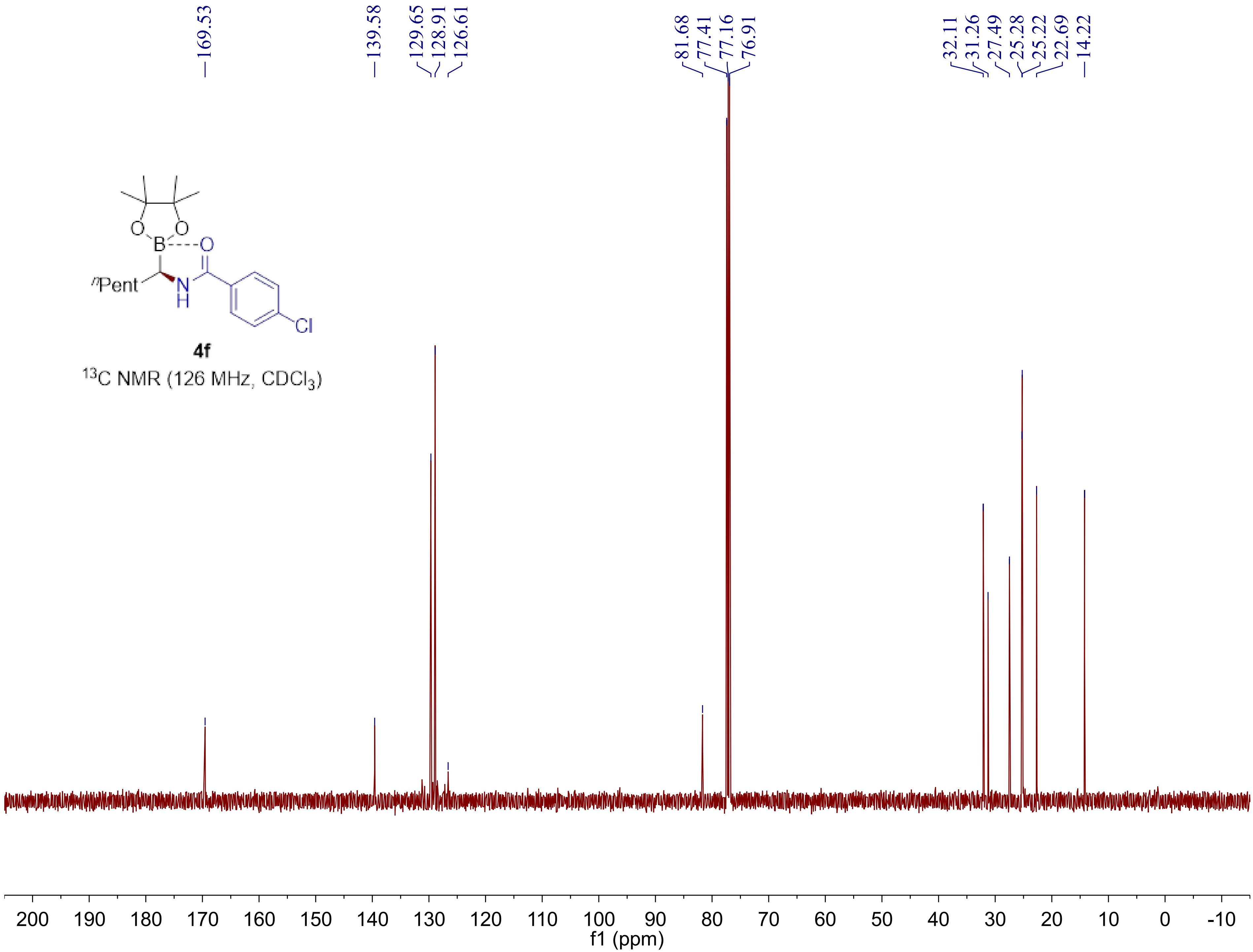

Supplementary Figure 72: ^{13}C NMR (126 MHz, $CDCl_3$) spectrum of **4f**.

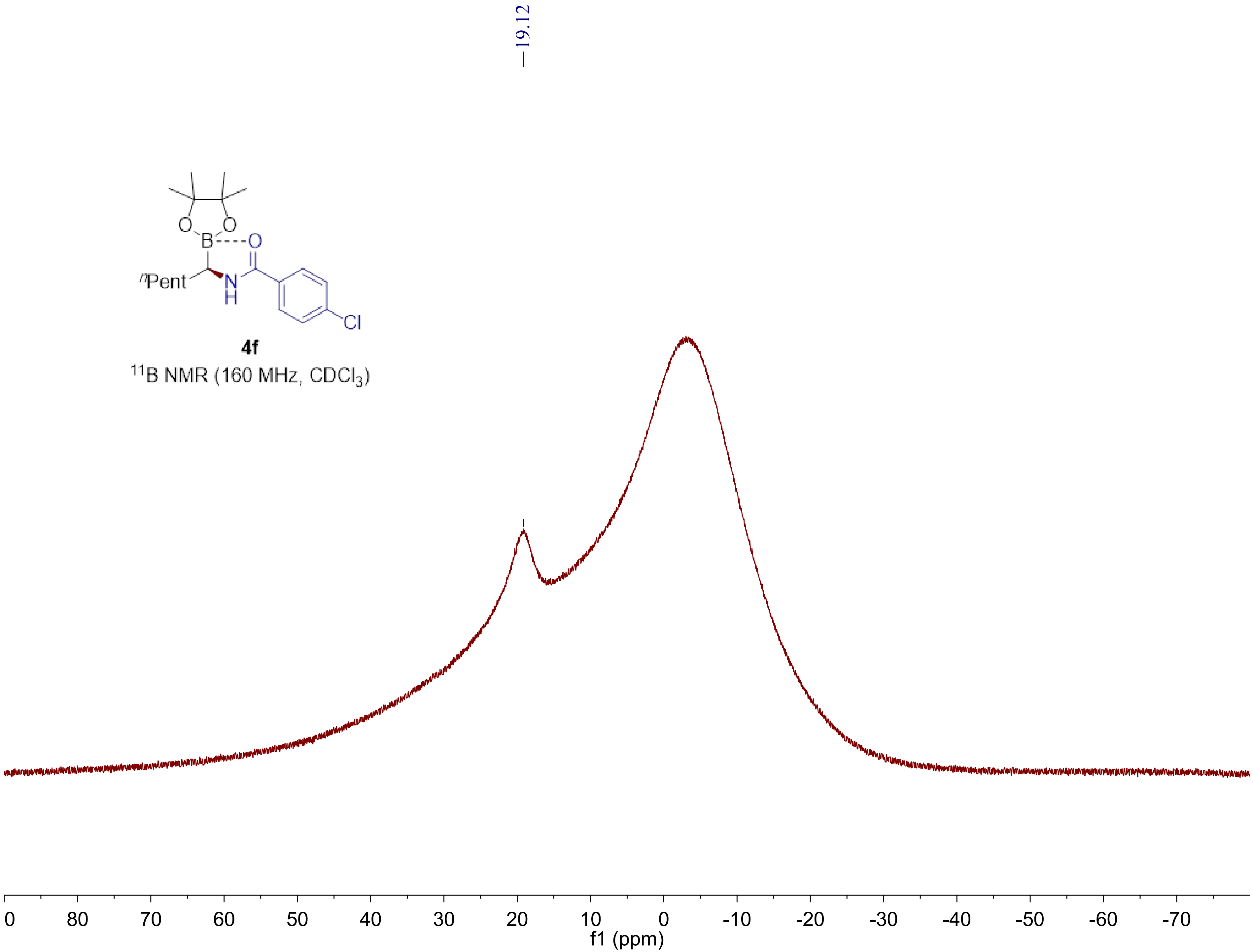

Supplementary Figure 73: ^{11}B NMR (160 MHz, $CDCl_3$) spectrum of **4f**.

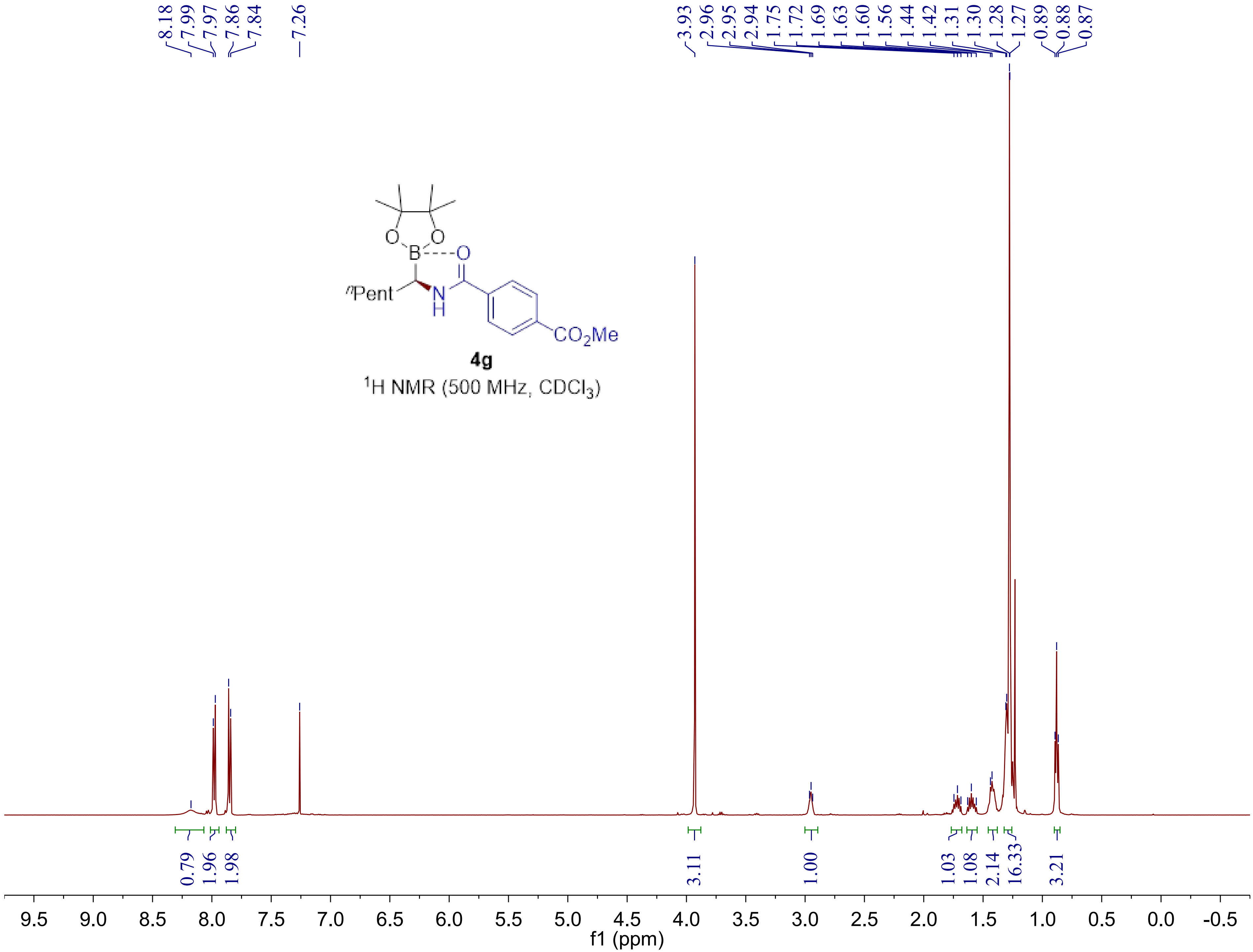

Supplementary Figure 74: ^{1}H NMR (500 MHz, $CDCl_3$) spectrum of **4g**.

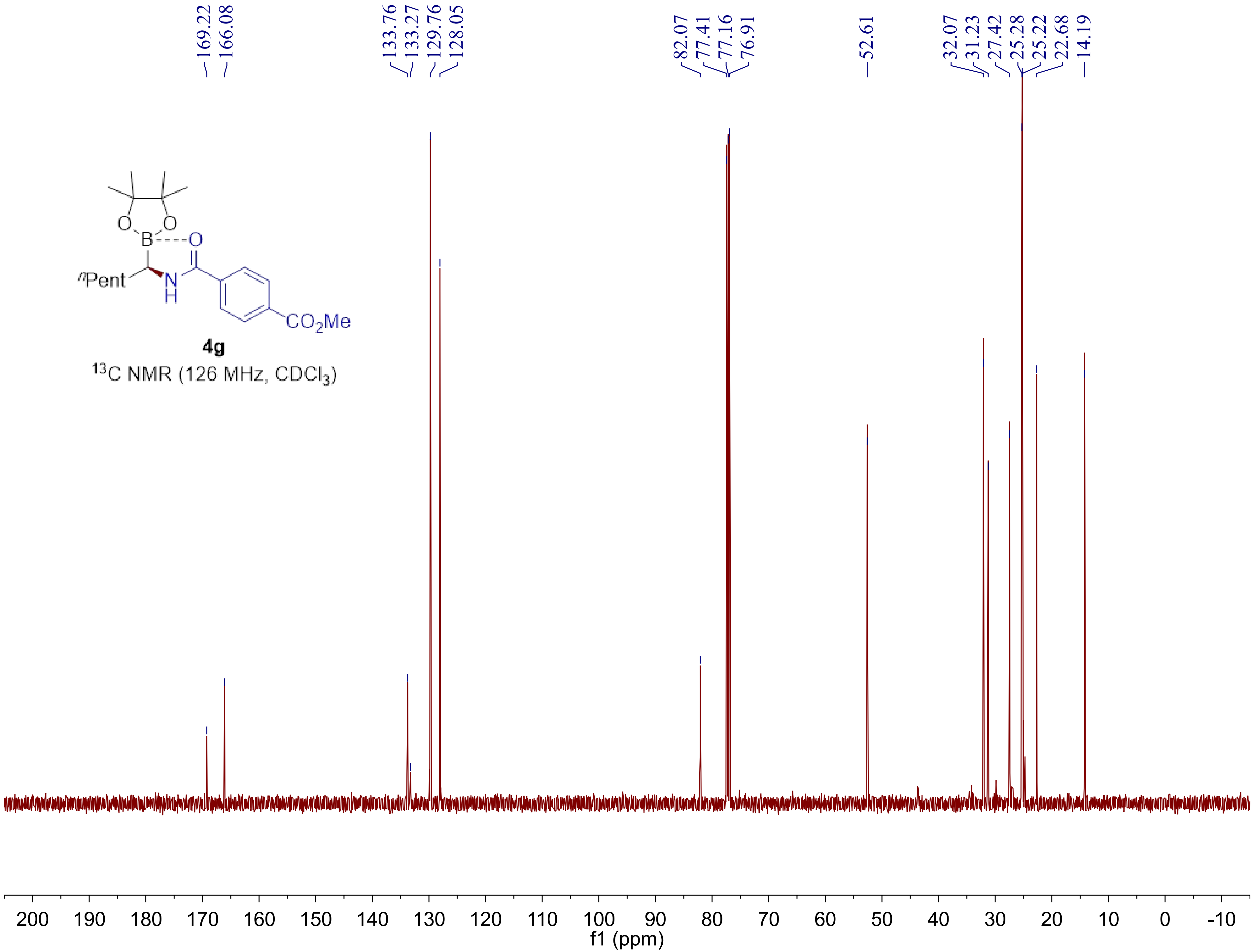

Supplementary Figure 75: ^{13}C NMR (126 MHz, $CDCl_3$) spectrum of **4g**.

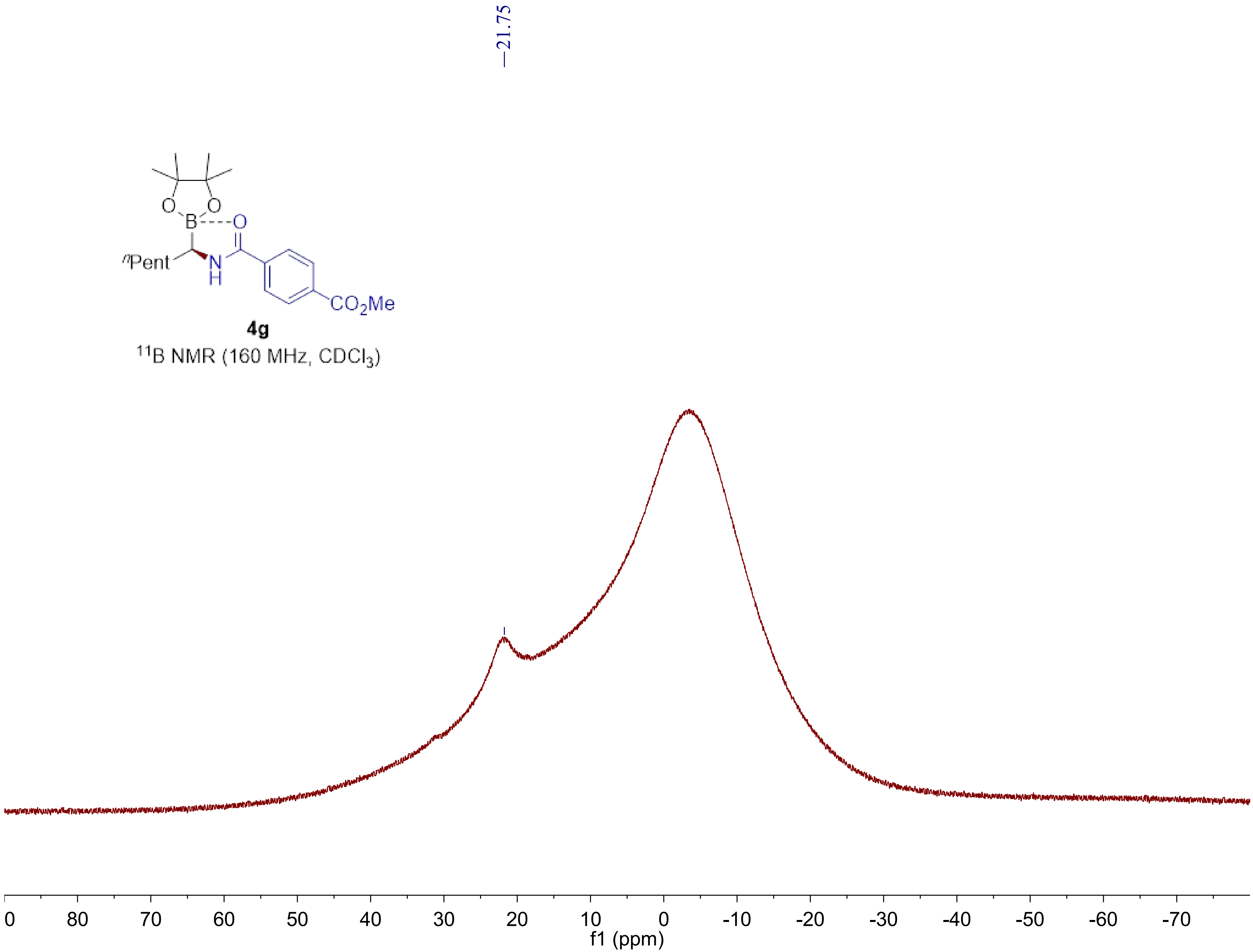

Supplementary Figure 76: ^{11}B NMR (160 MHz, CDCl$_3$) spectrum of **4g**.

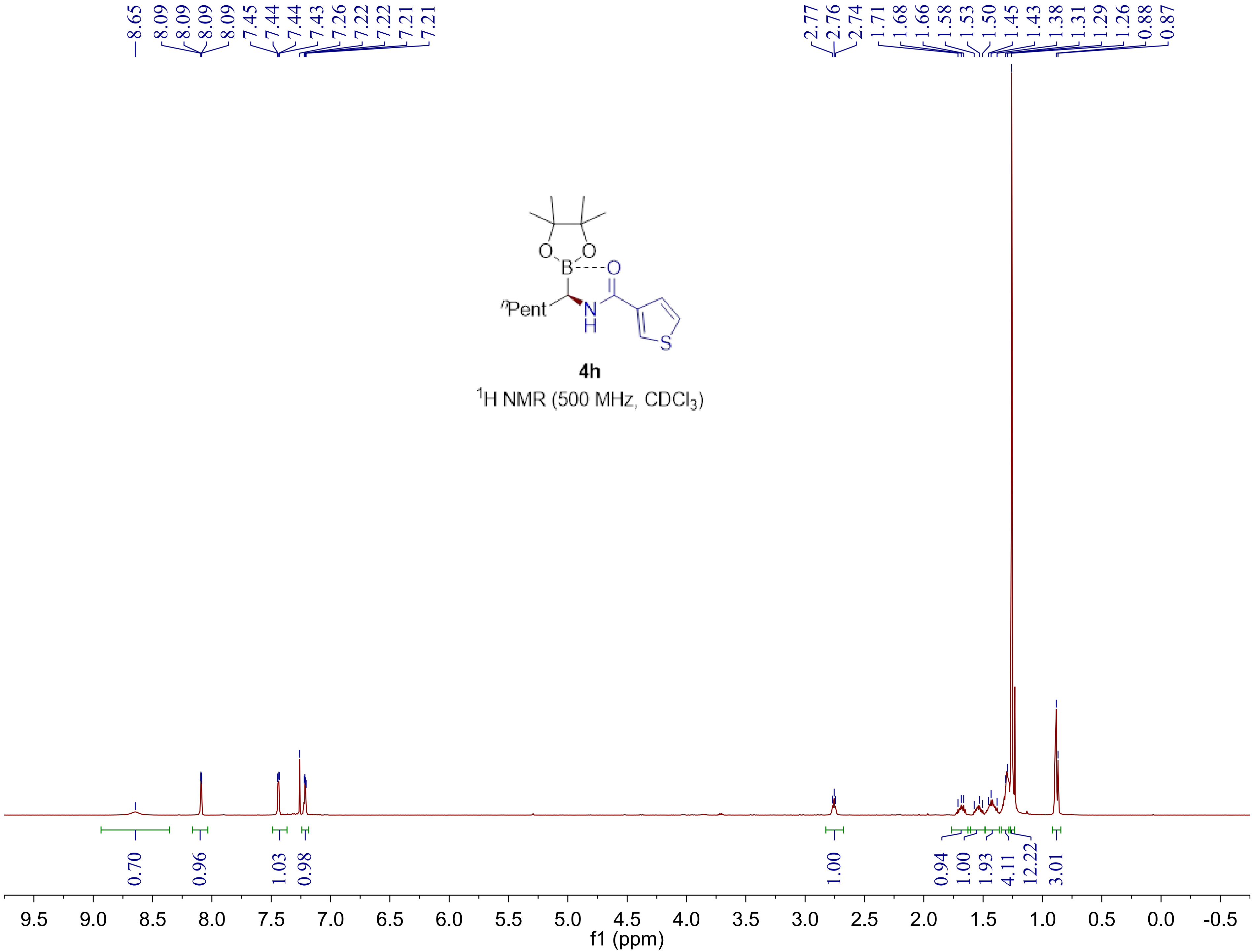

Supplementary Figure 77: ^{1}H NMR (500 MHz, $CDCl_3$) spectrum of **4h**.

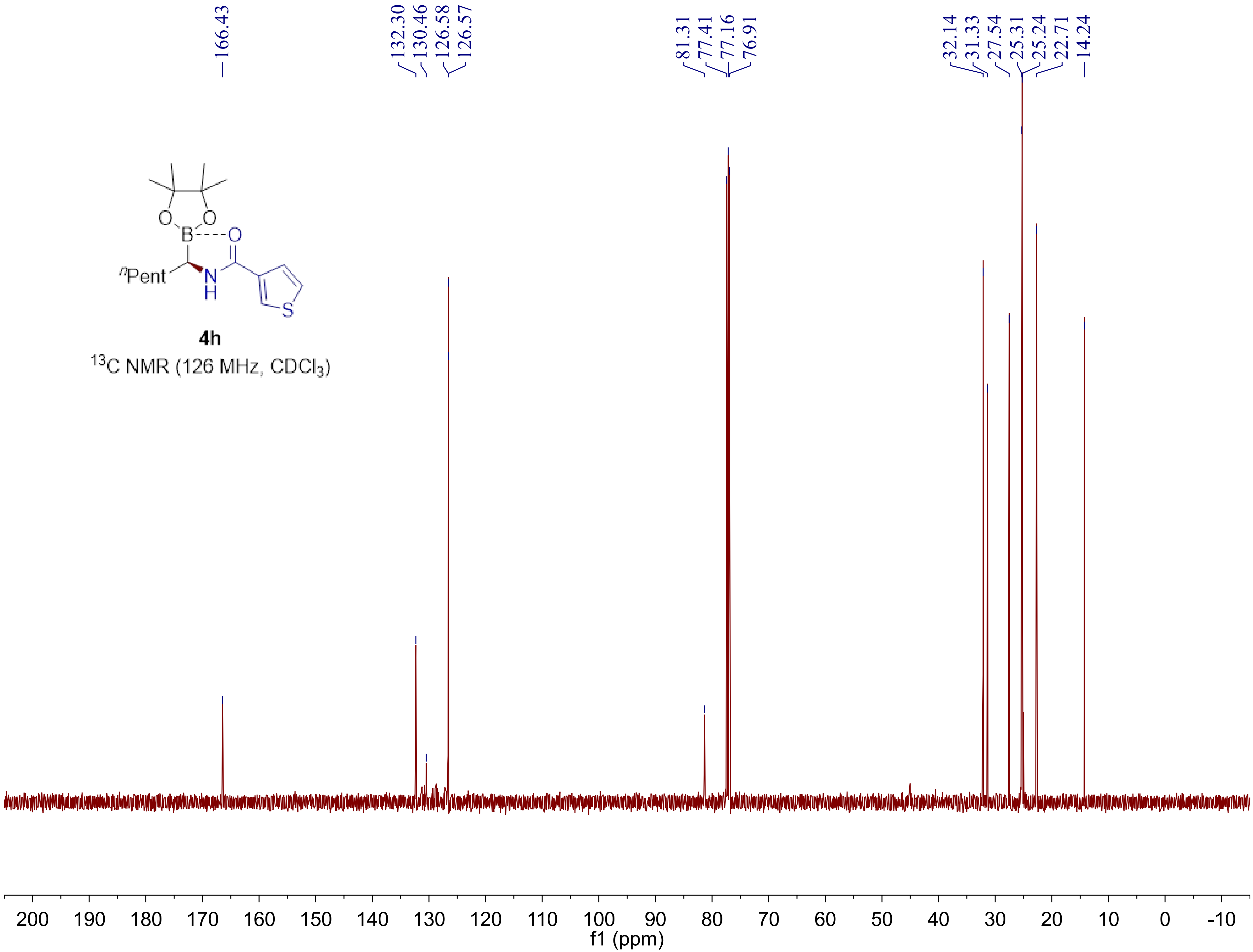

Supplementary Figure 78: ^{13}C NMR (126 MHz, $CDCl_3$) spectrum of **4h**.

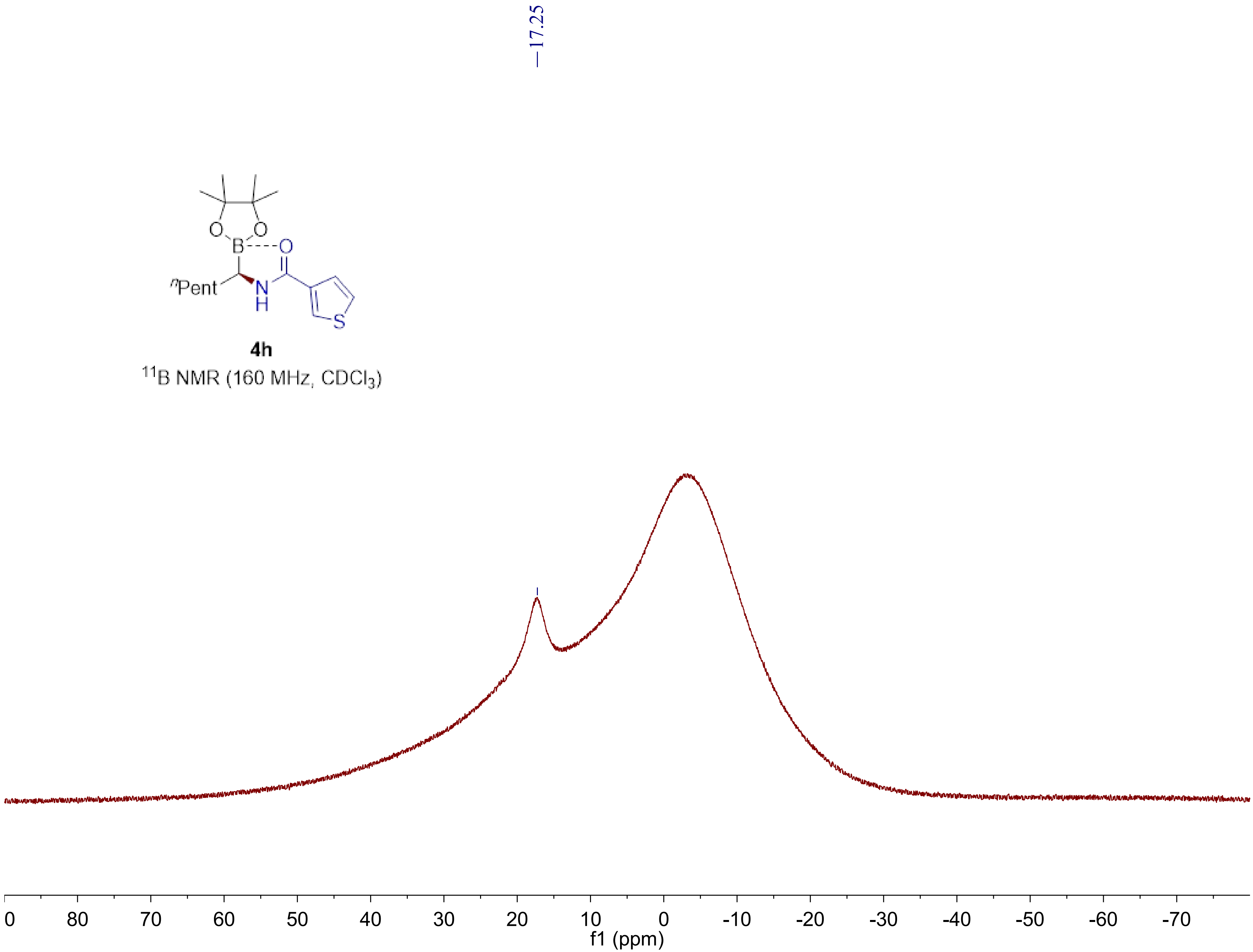

Supplementary Figure 79: ^{11}B NMR (160 MHz, $CDCl_3$) spectrum of **4h**.

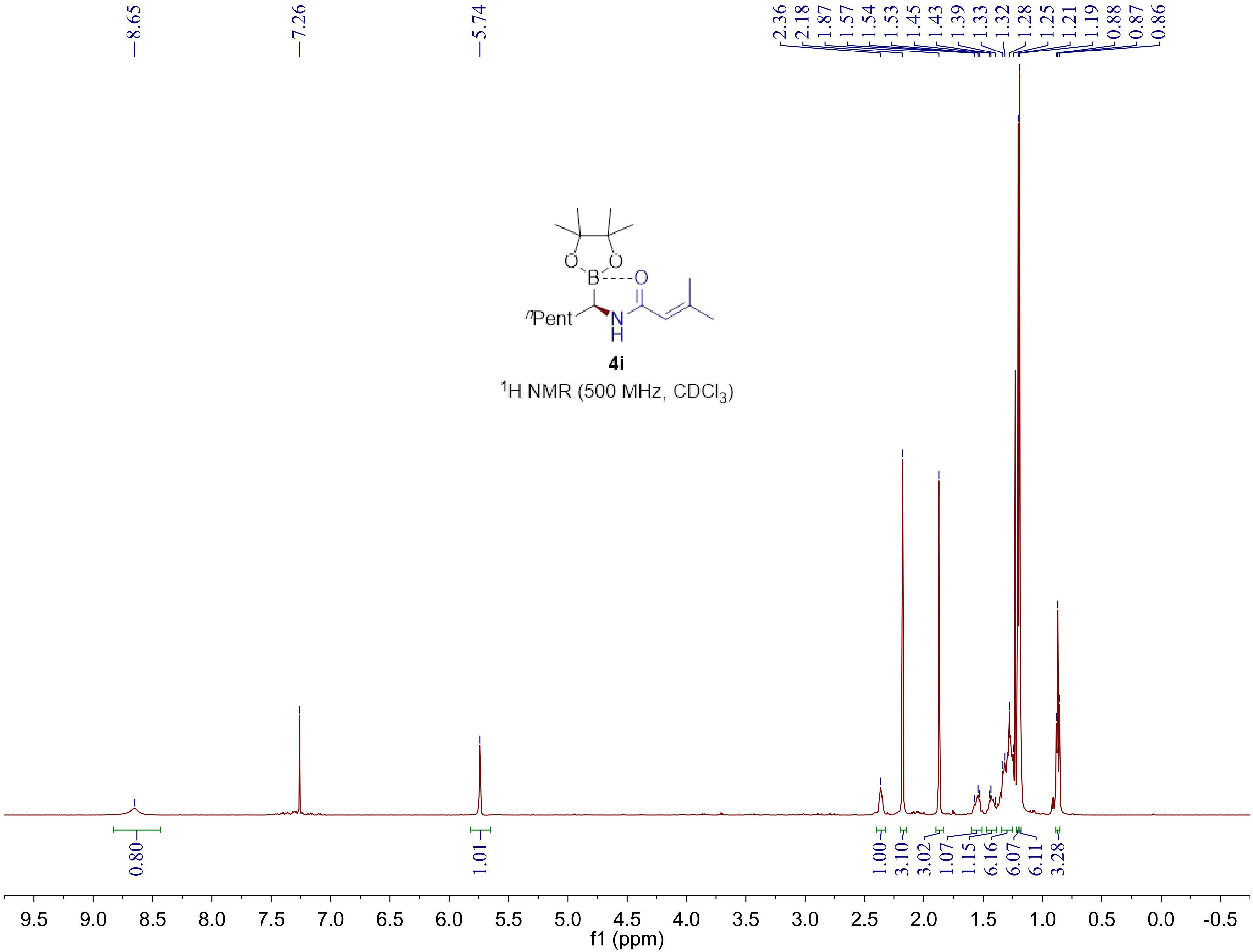

Supplementary Figure 80: ^{1}H NMR (500 MHz, $CDCl_3$) spectrum of **4i**.

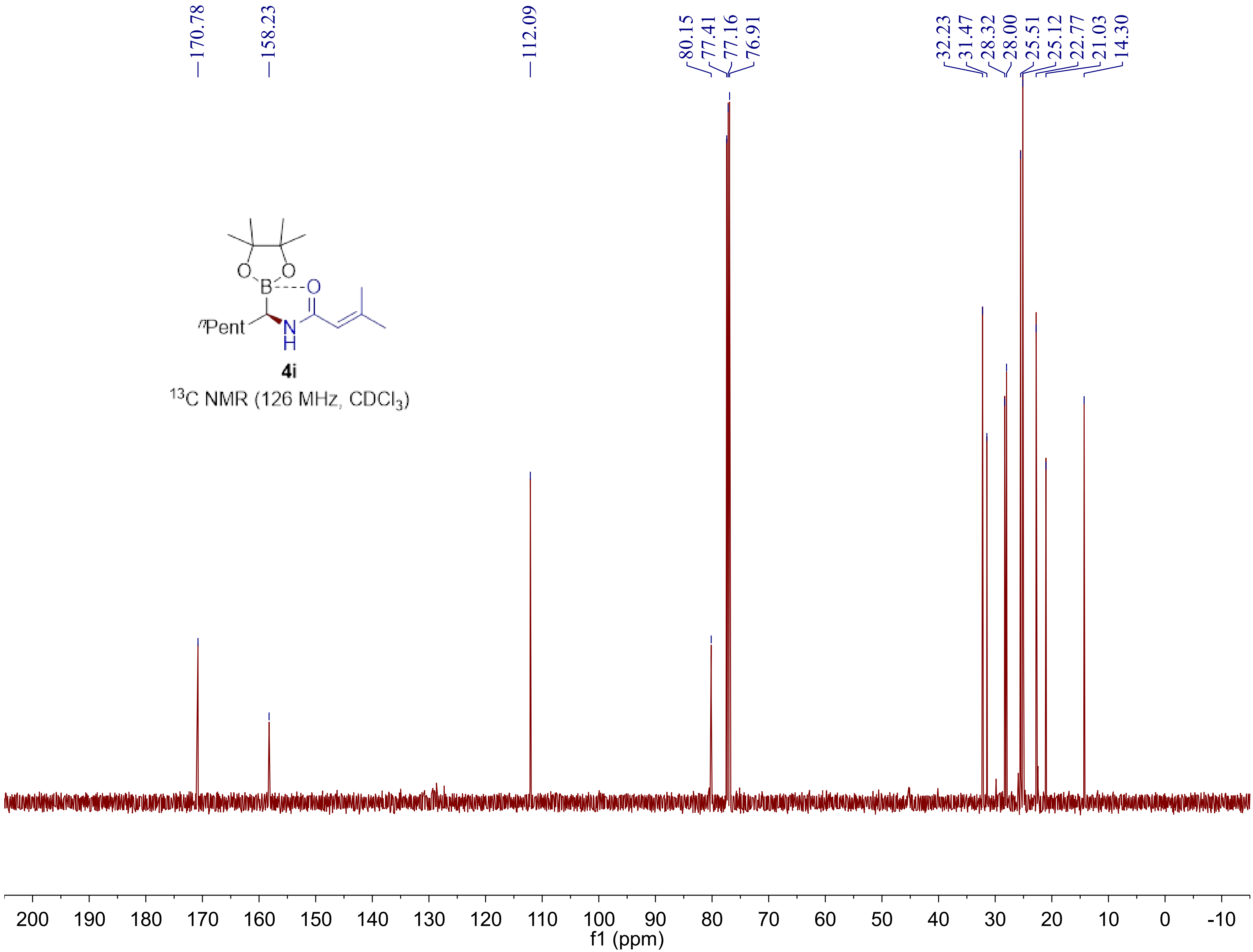

Supplementary Figure 81: ^{13}C NMR (126 MHz, $CDCl_3$) spectrum of **4i**.

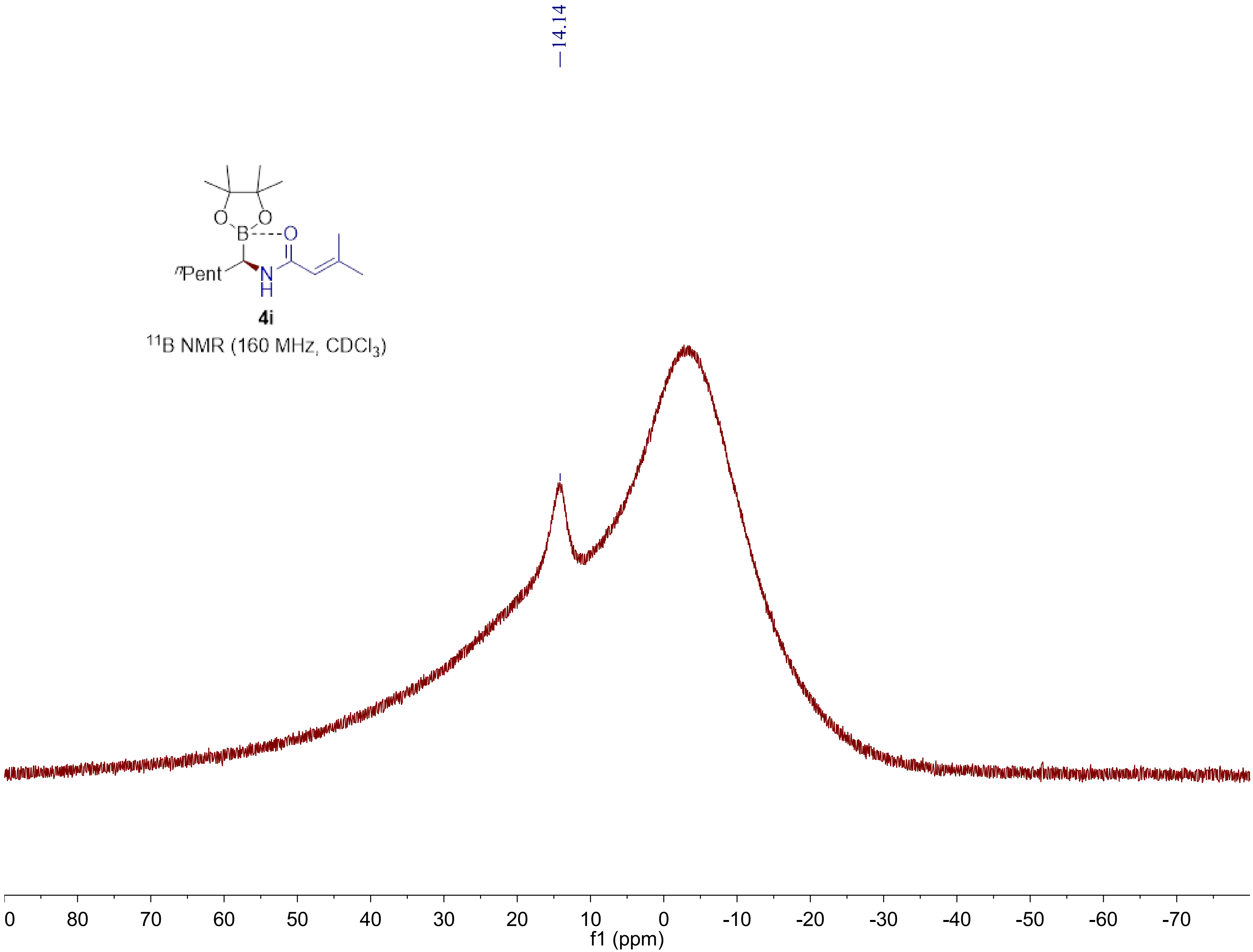

Supplementary Figure 82: ^{11}B NMR (160 MHz, $CDCl_3$) spectrum of **4i**.

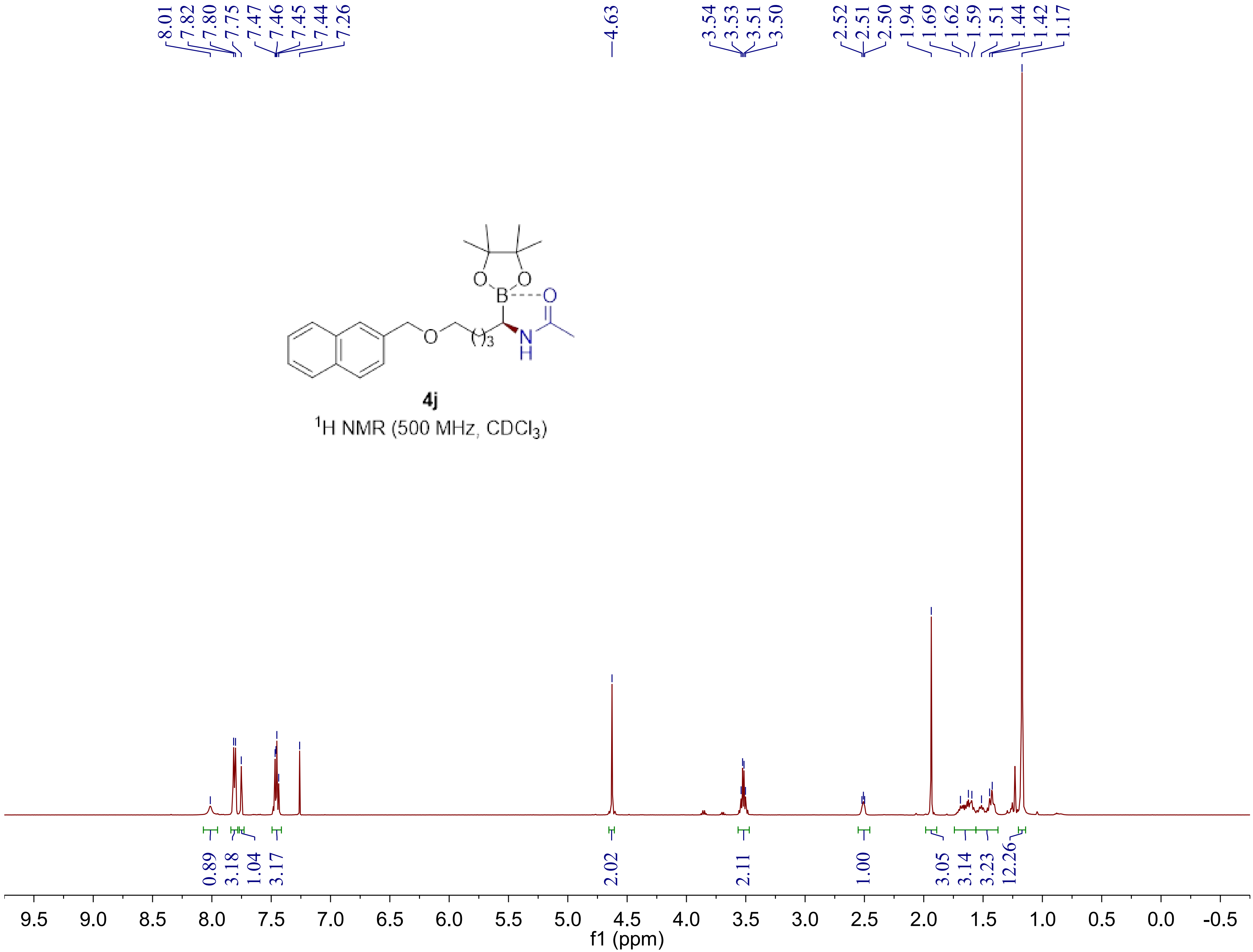

Supplementary Figure 83: ^{1}H NMR (500 MHz, $CDCl_3$) spectrum of **4j**.

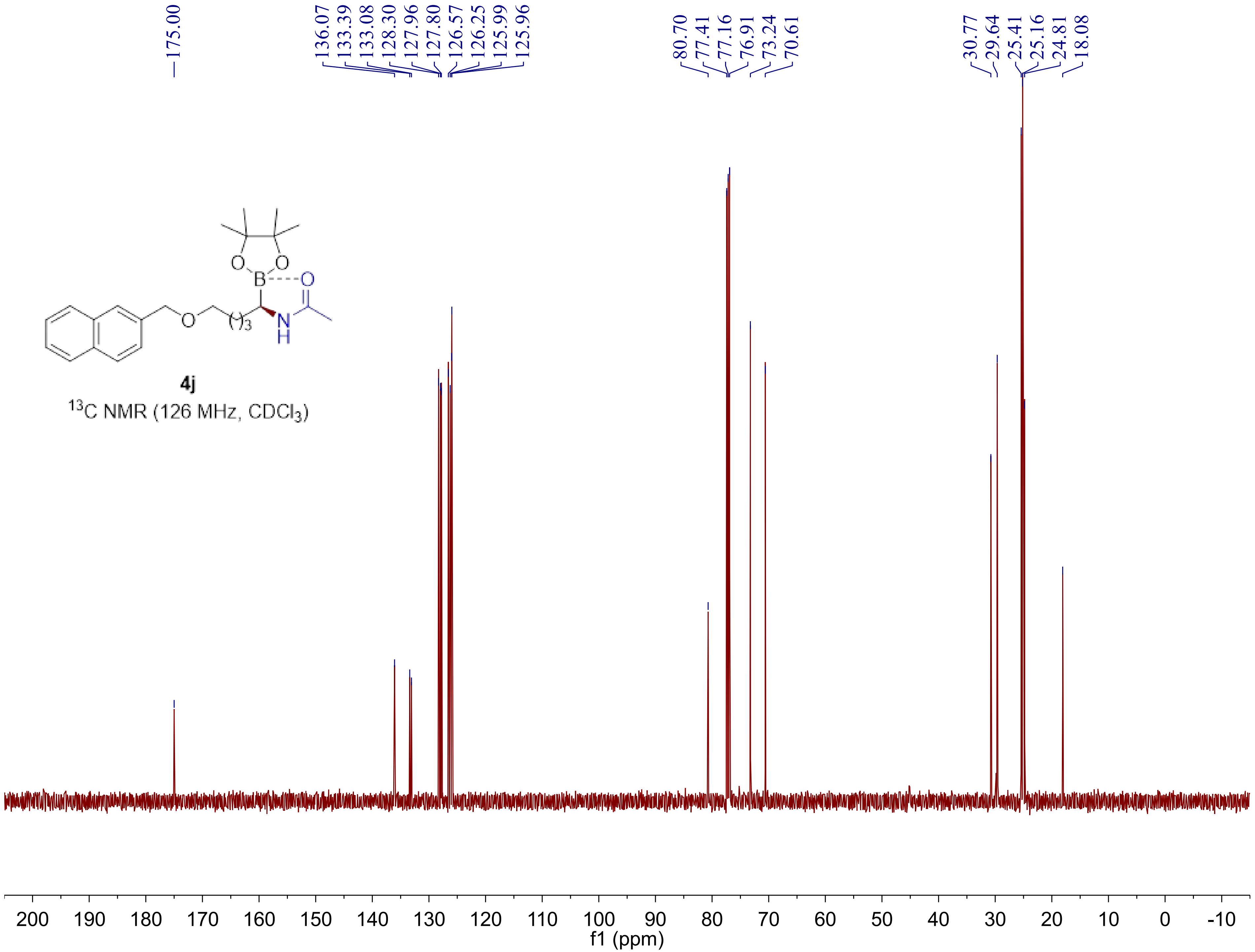

Supplementary Figure 84: ^{13}C NMR (126 MHz, $CDCl_3$) spectrum of **4j**.

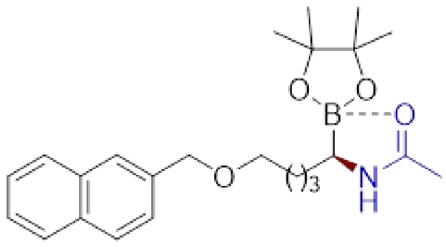

4j

^{11}B NMR (160 MHz, $CDCl_3$)

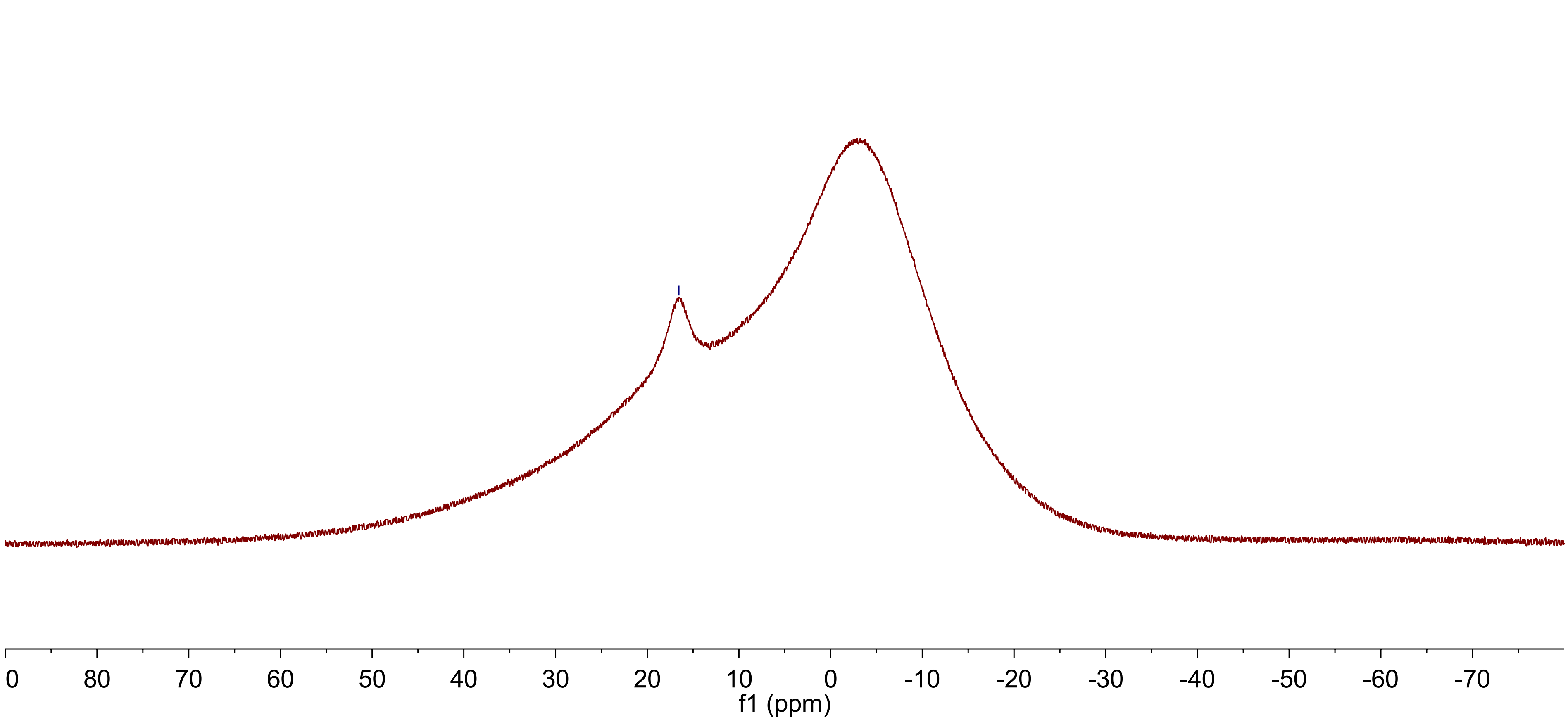

Supplementary Figure 85: ^{11}B NMR (160 MHz, $CDCl_3$) spectrum of **4j**.

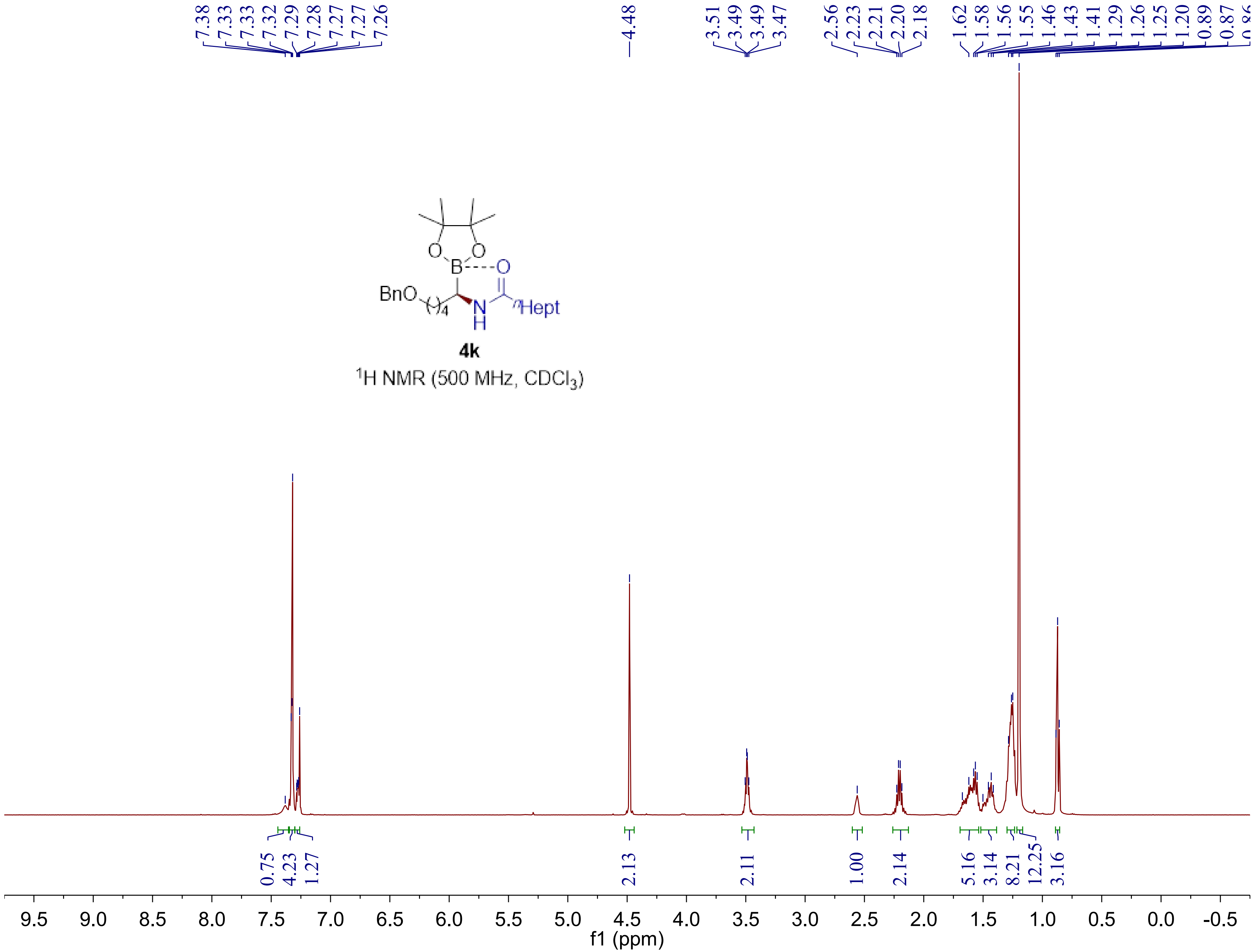

Supplementary Figure 86: ^{1}H NMR (500 MHz, $CDCl_3$) spectrum of **4k**.

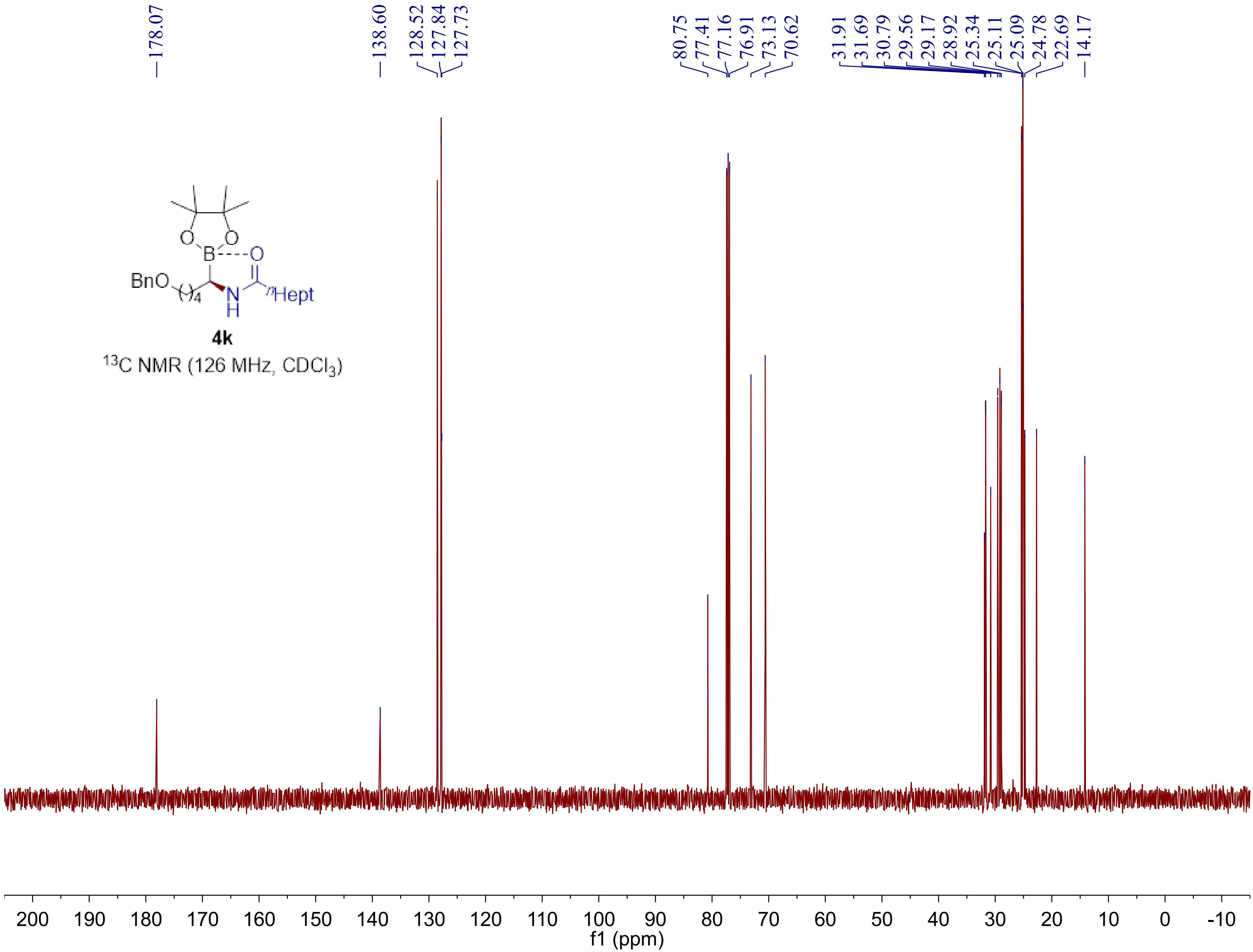

Supplementary Figure 87: ^{13}C NMR (126 MHz, $CDCl_3$) spectrum of **4k**.

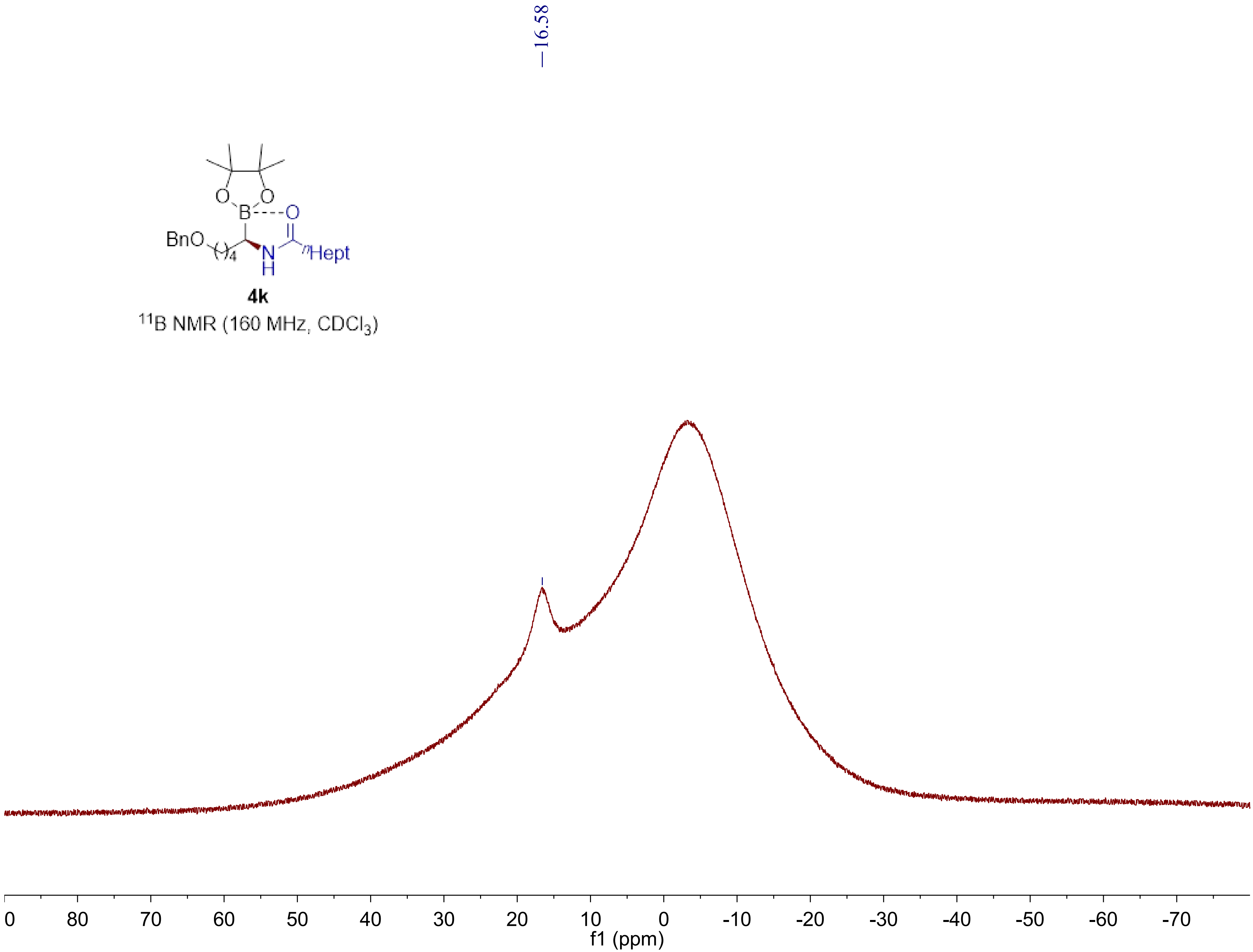

Supplementary Figure 88: ^{11}B NMR (160 MHz, $CDCl_3$) spectrum of **4k**.

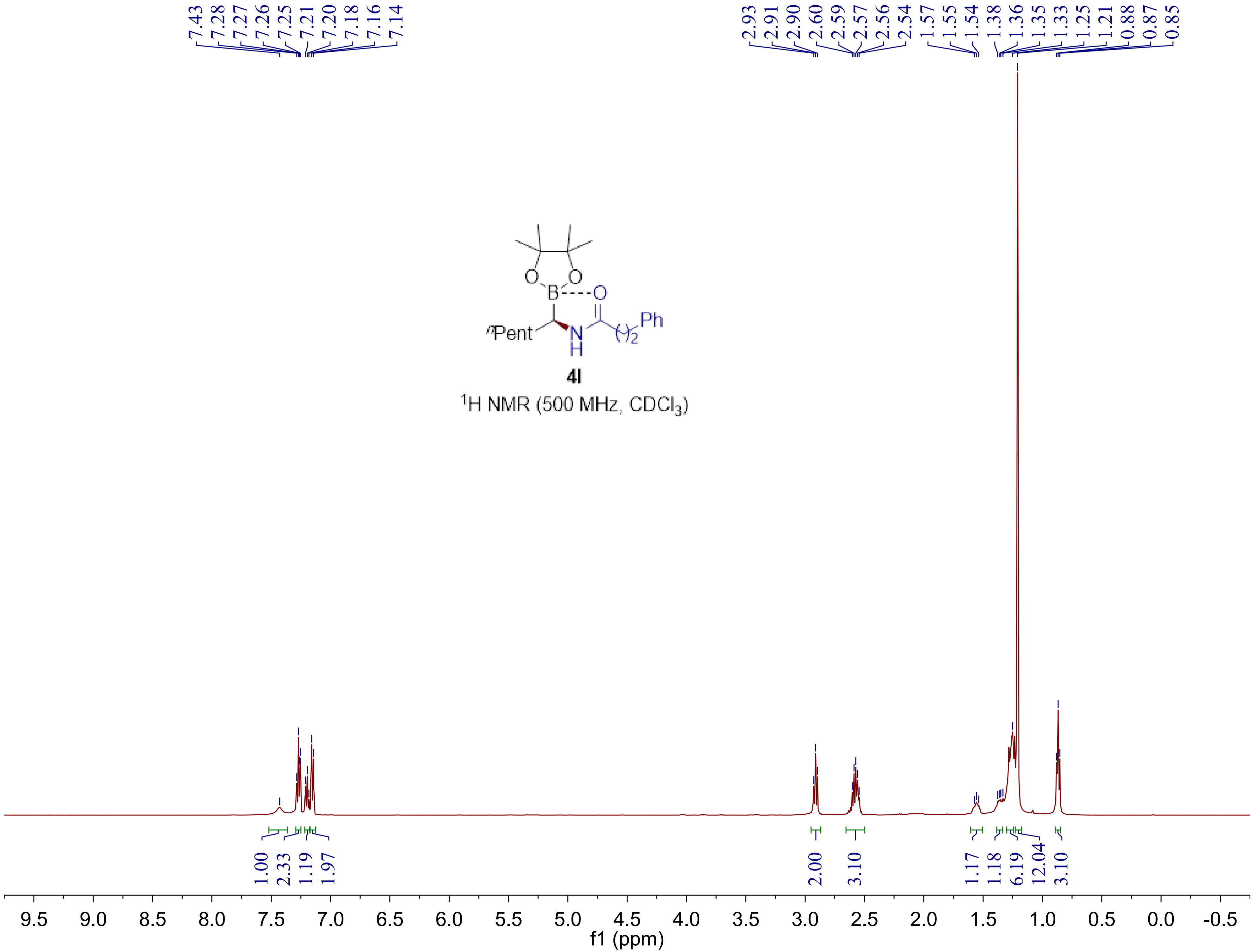

Supplementary Figure 89: ^{1}H NMR (500 MHz, $CDCl_3$) spectrum of **4l**.

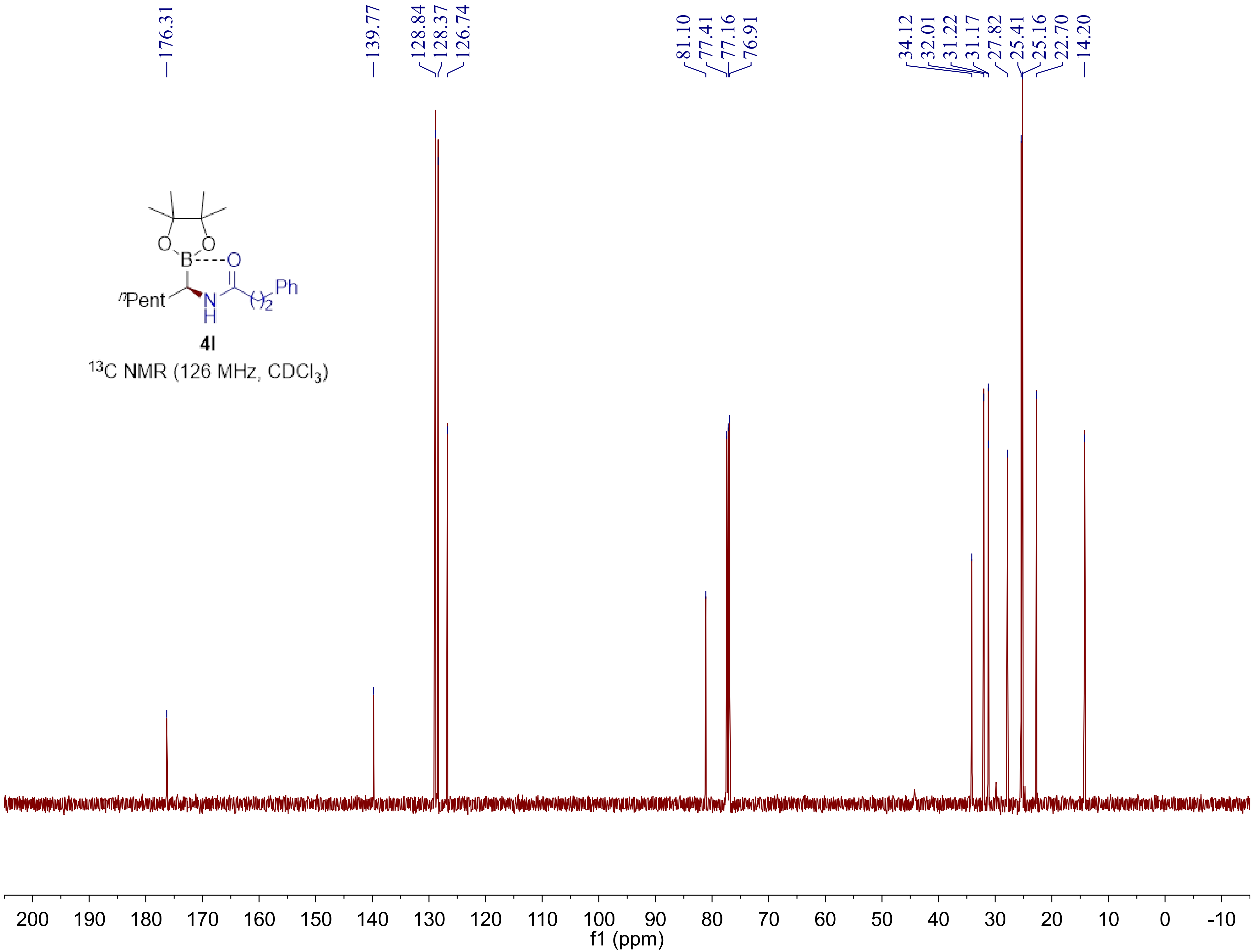

Supplementary Figure 90: ^{13}C NMR (126 MHz, $CDCl_3$) spectrum of **4l**.

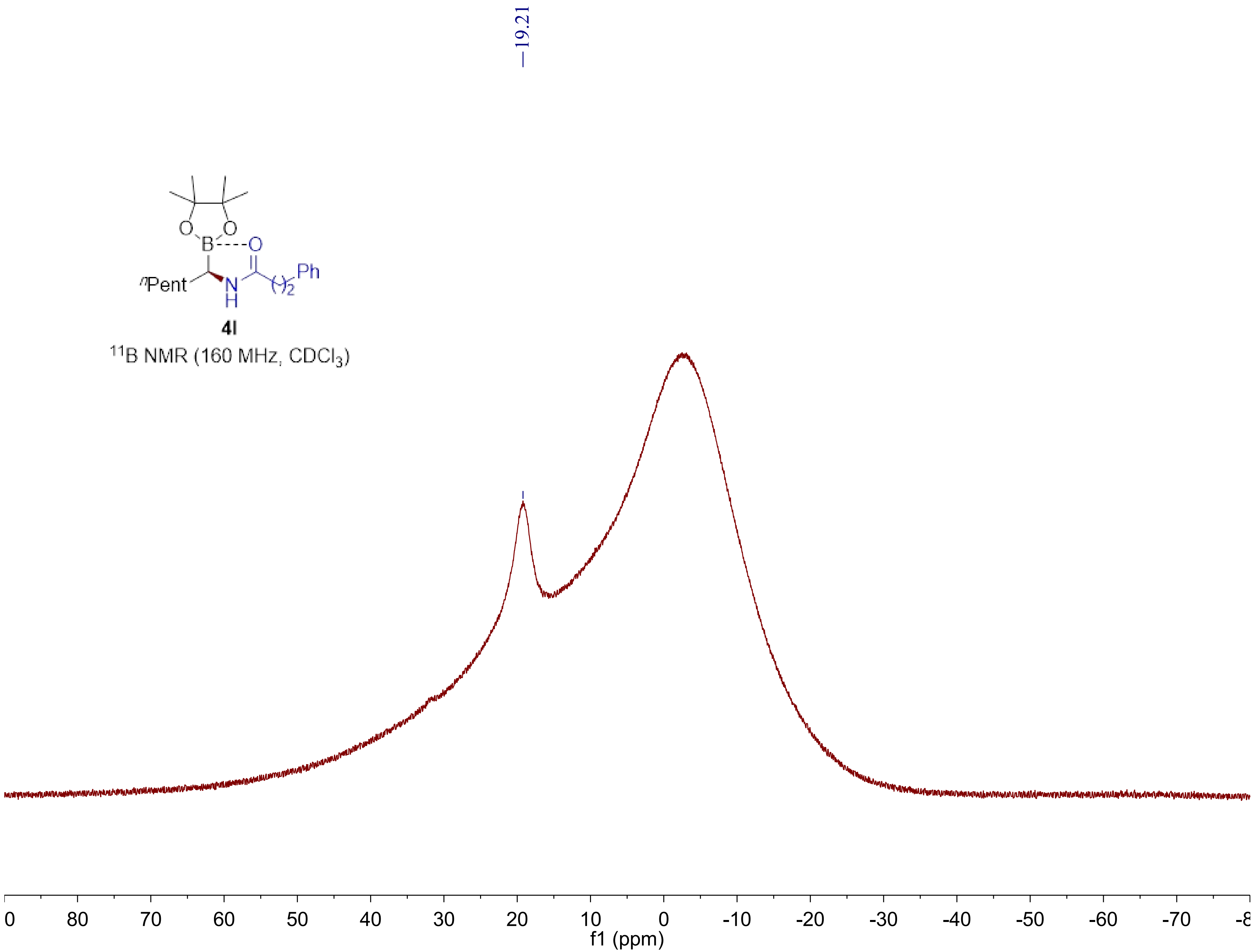

Supplementary Figure 91: ^{11}B NMR (160 MHz, $CDCl_3$) spectrum of **4l**.

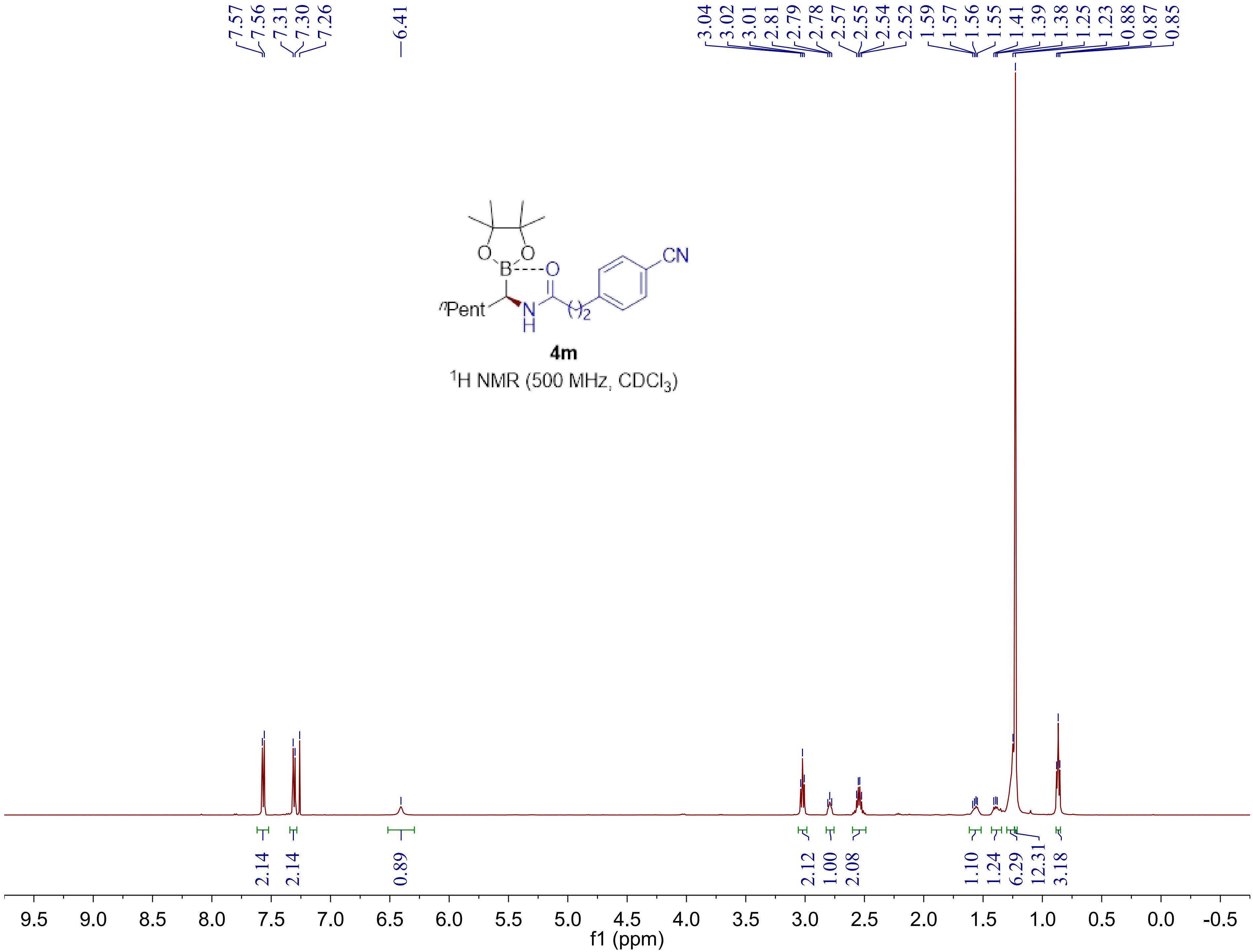

Supplementary Figure 92: ^{1}H NMR (500 MHz, $CDCl_3$) spectrum of **4m**.

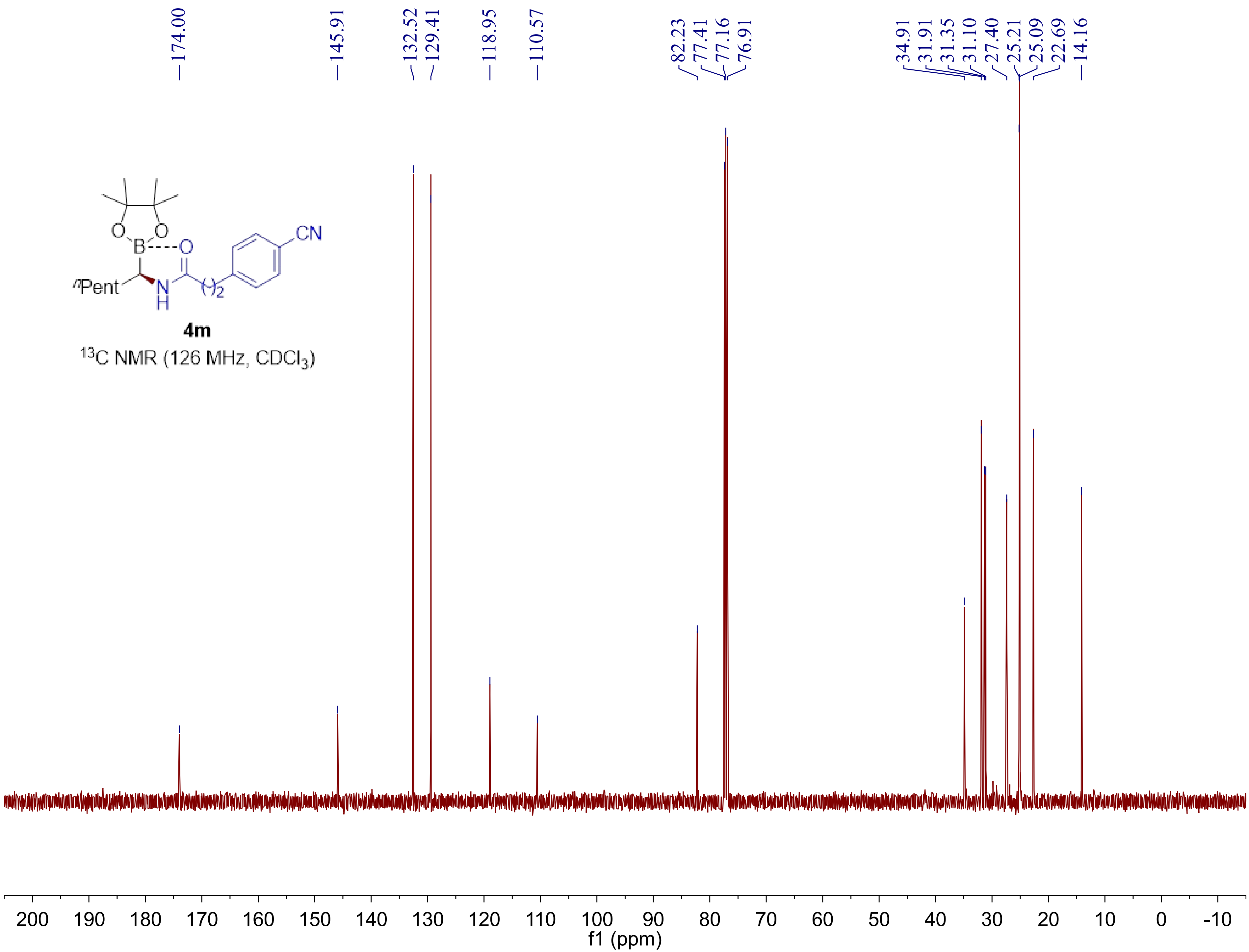

Supplementary Figure 93: ^{13}C NMR (126 MHz, $CDCl_3$) spectrum of **4m**.

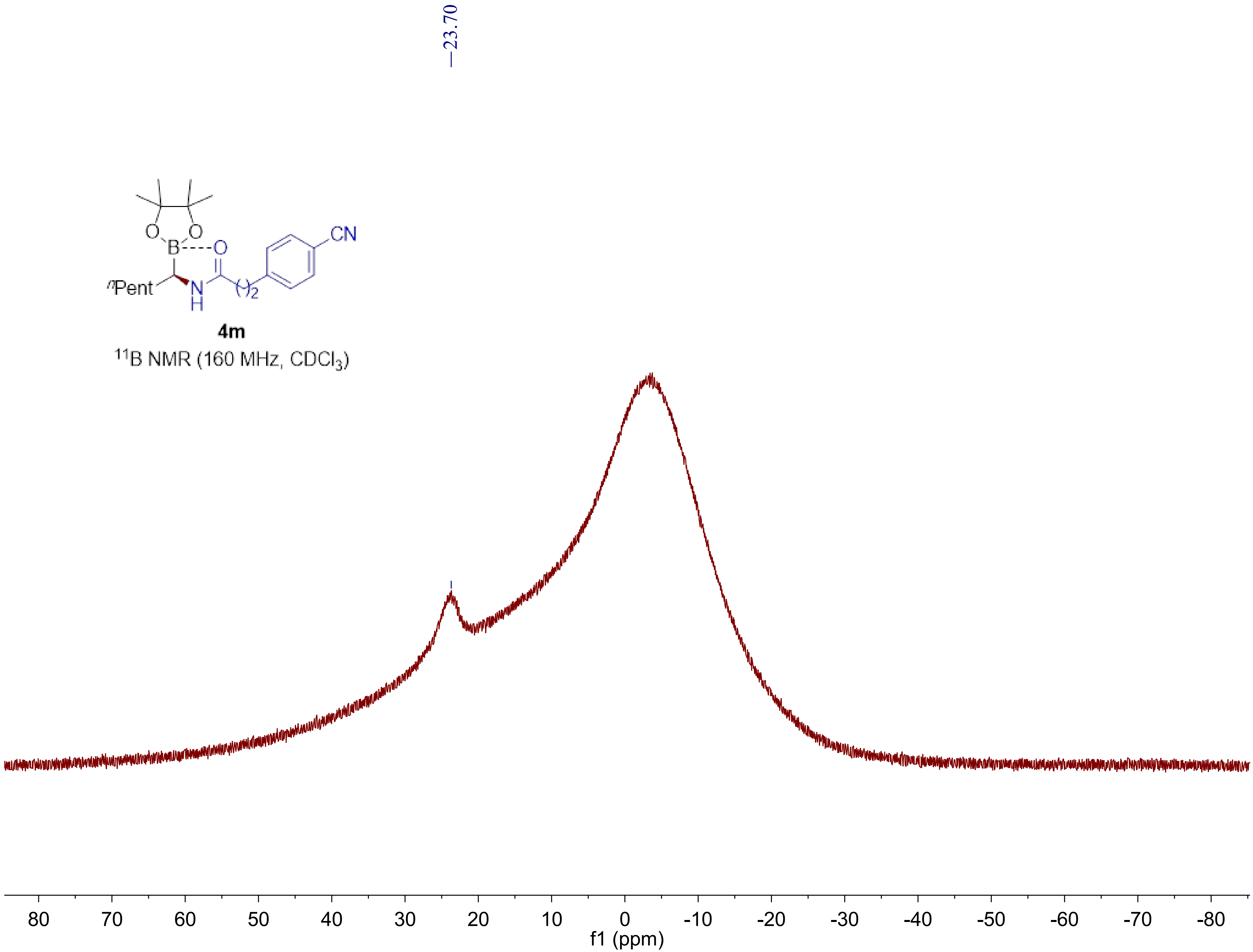

Supplementary Figure 94: ^{11}B NMR (160 MHz, $CDCl_3$) spectrum of **4m**.

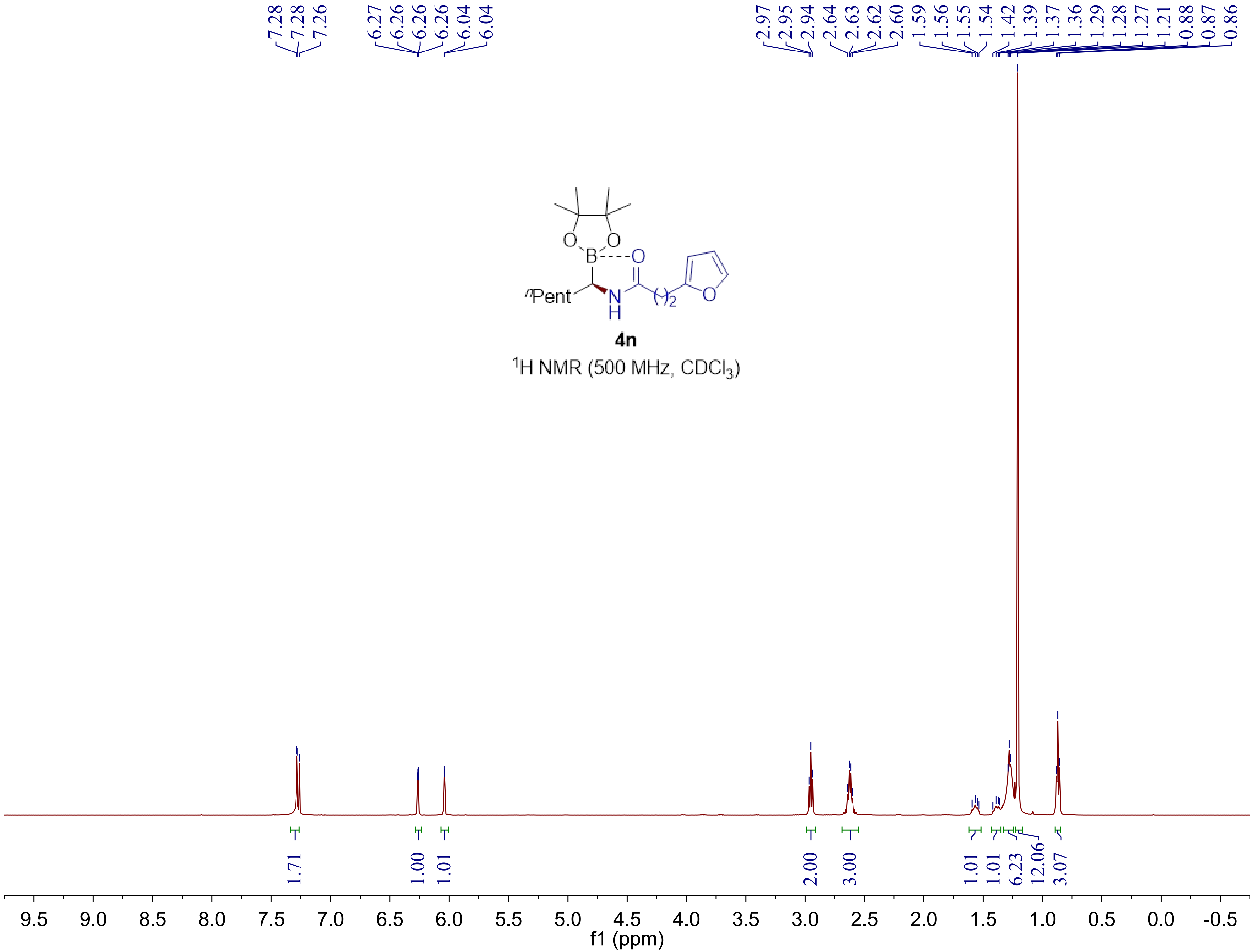

Supplementary Figure 95: ^{1}H NMR (500 MHz, $CDCl_3$) spectrum of **4n**.

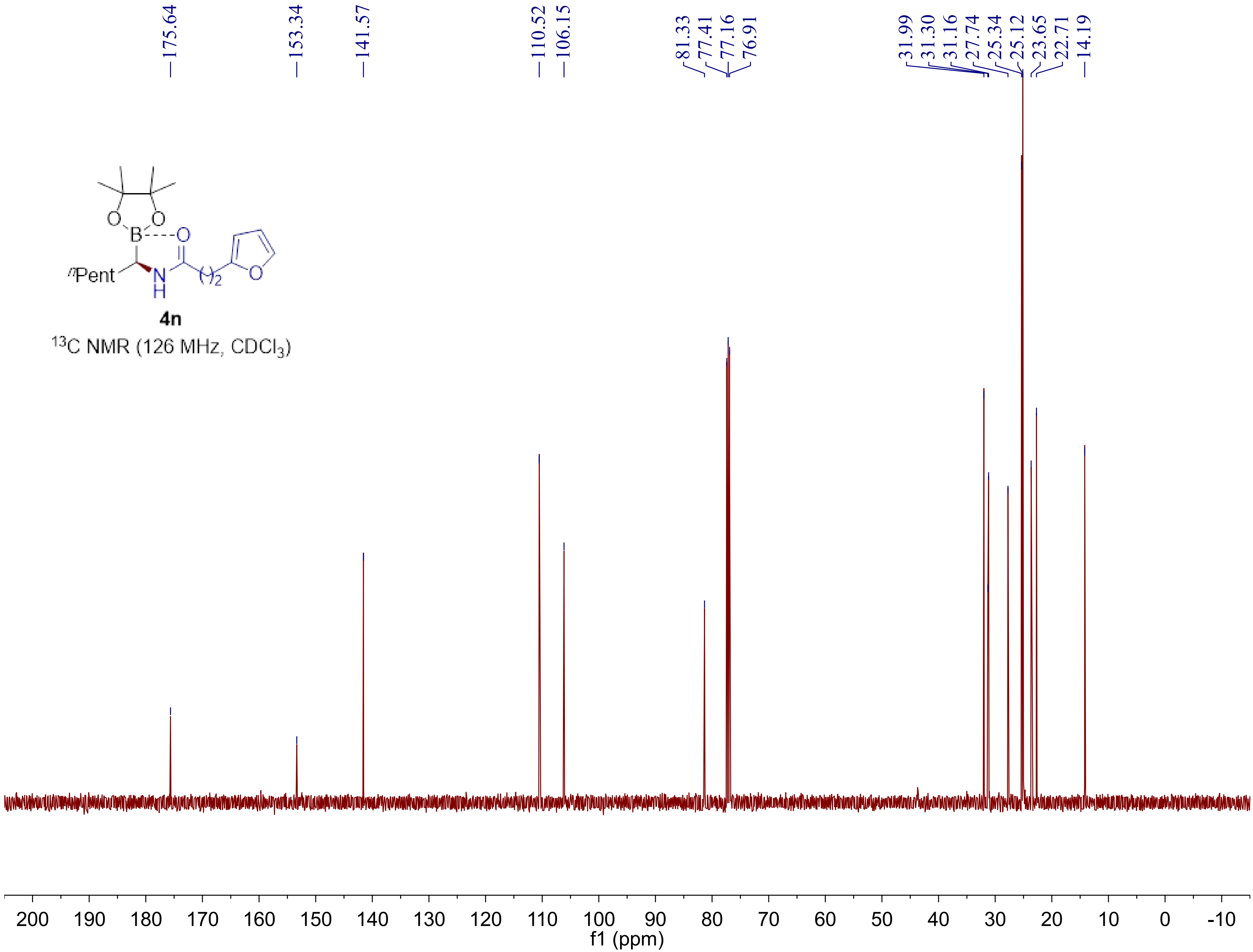

Supplementary Figure 96: ^{13}C NMR (126 MHz, $CDCl_3$) spectrum of **4n**.

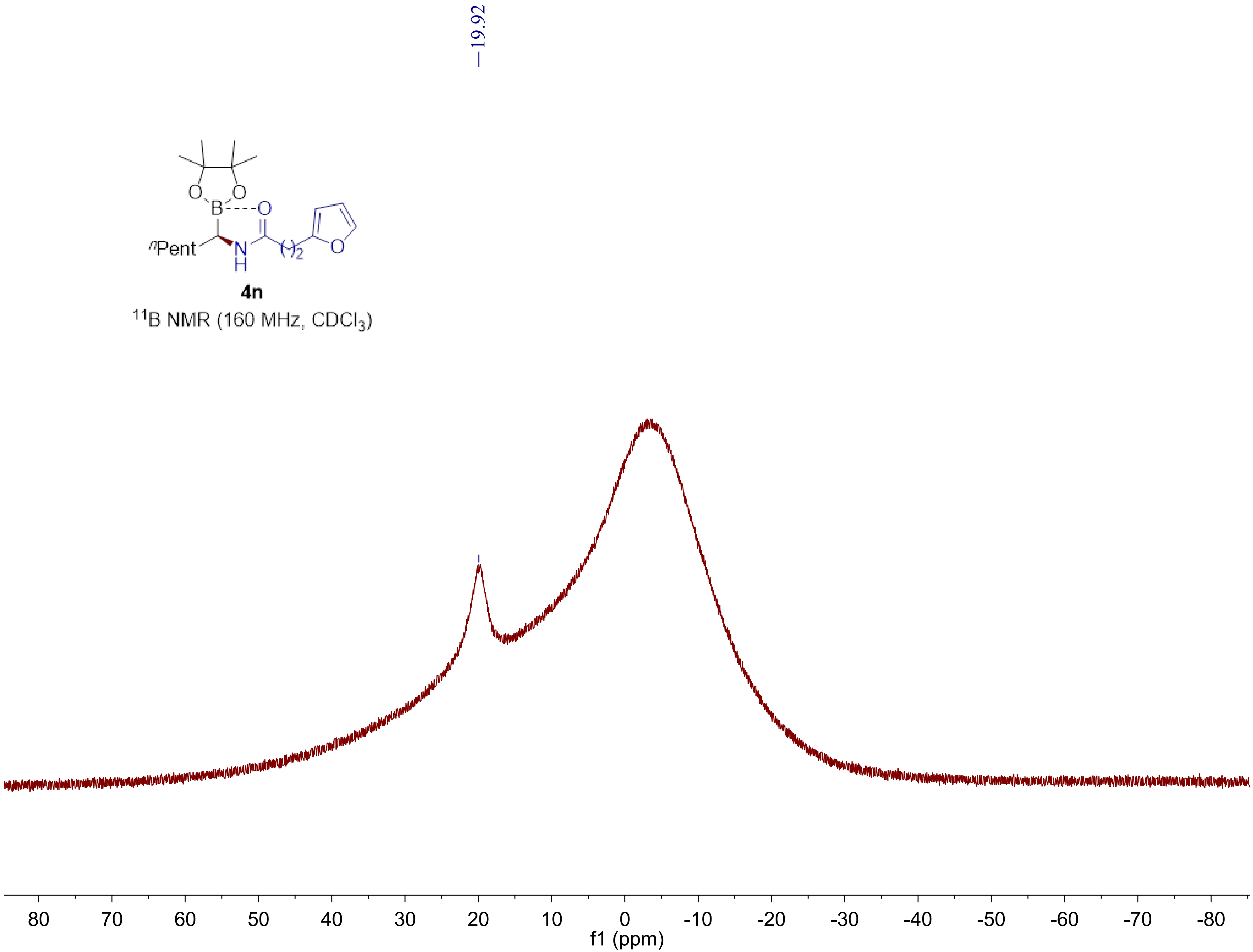

Supplementary Figure 97: ^{11}B NMR (160 MHz, $CDCl_3$) spectrum of **4n**.

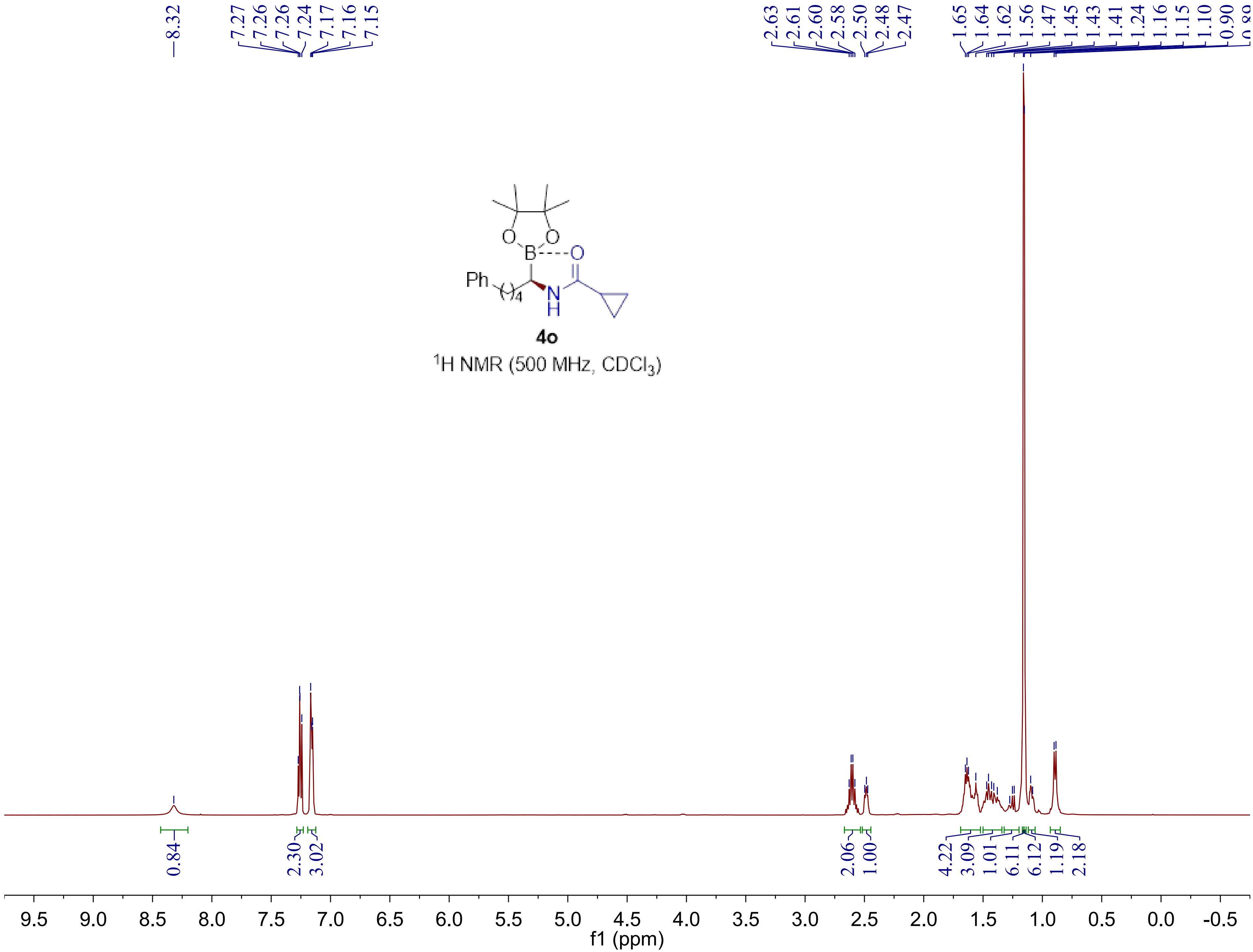

Supplementary Figure 98: ^{1}H NMR (500 MHz, $CDCl_3$) spectrum of **4o**.

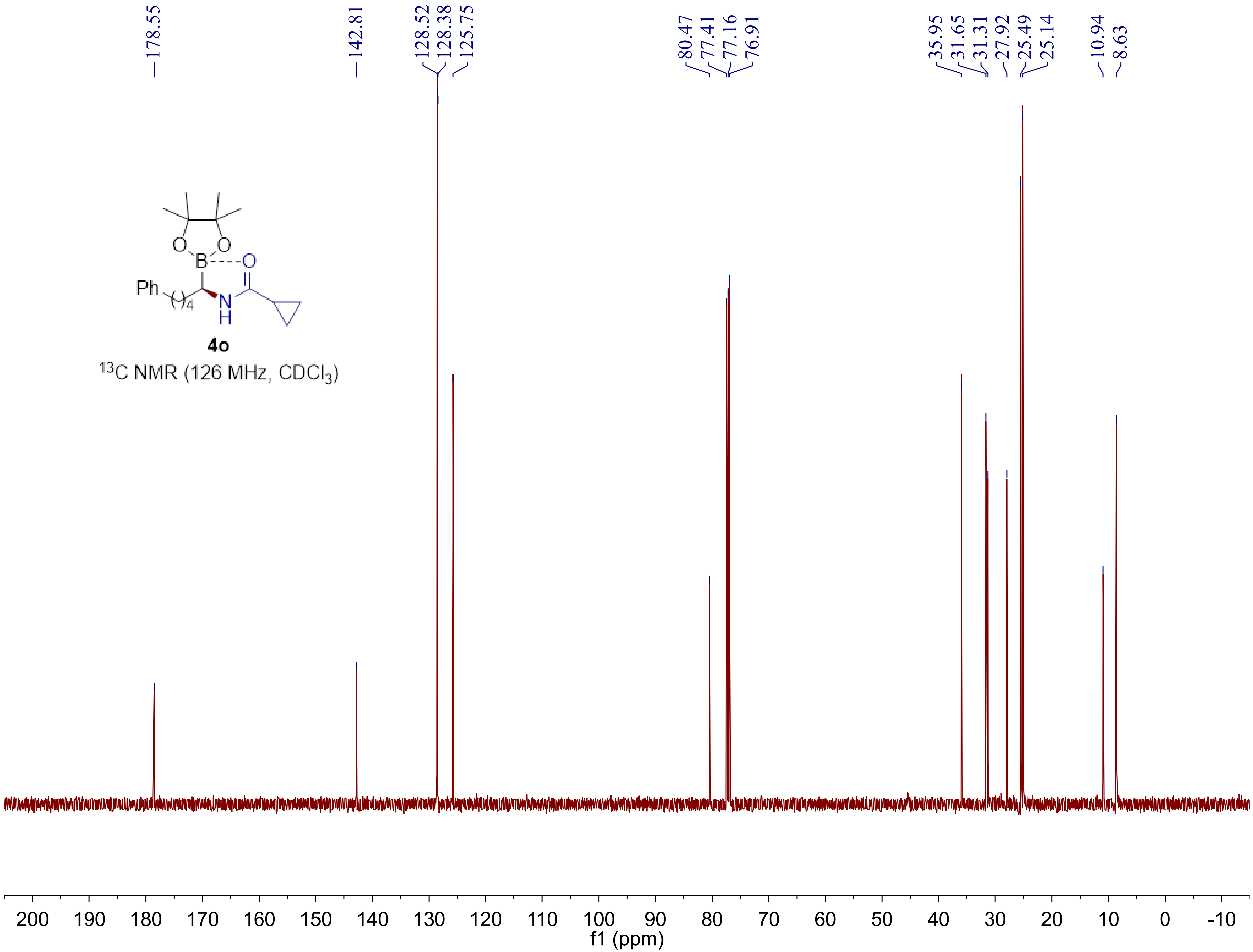

Supplementary Figure 99: ^{13}C NMR (126 MHz, $CDCl_3$) spectrum of **4o**.

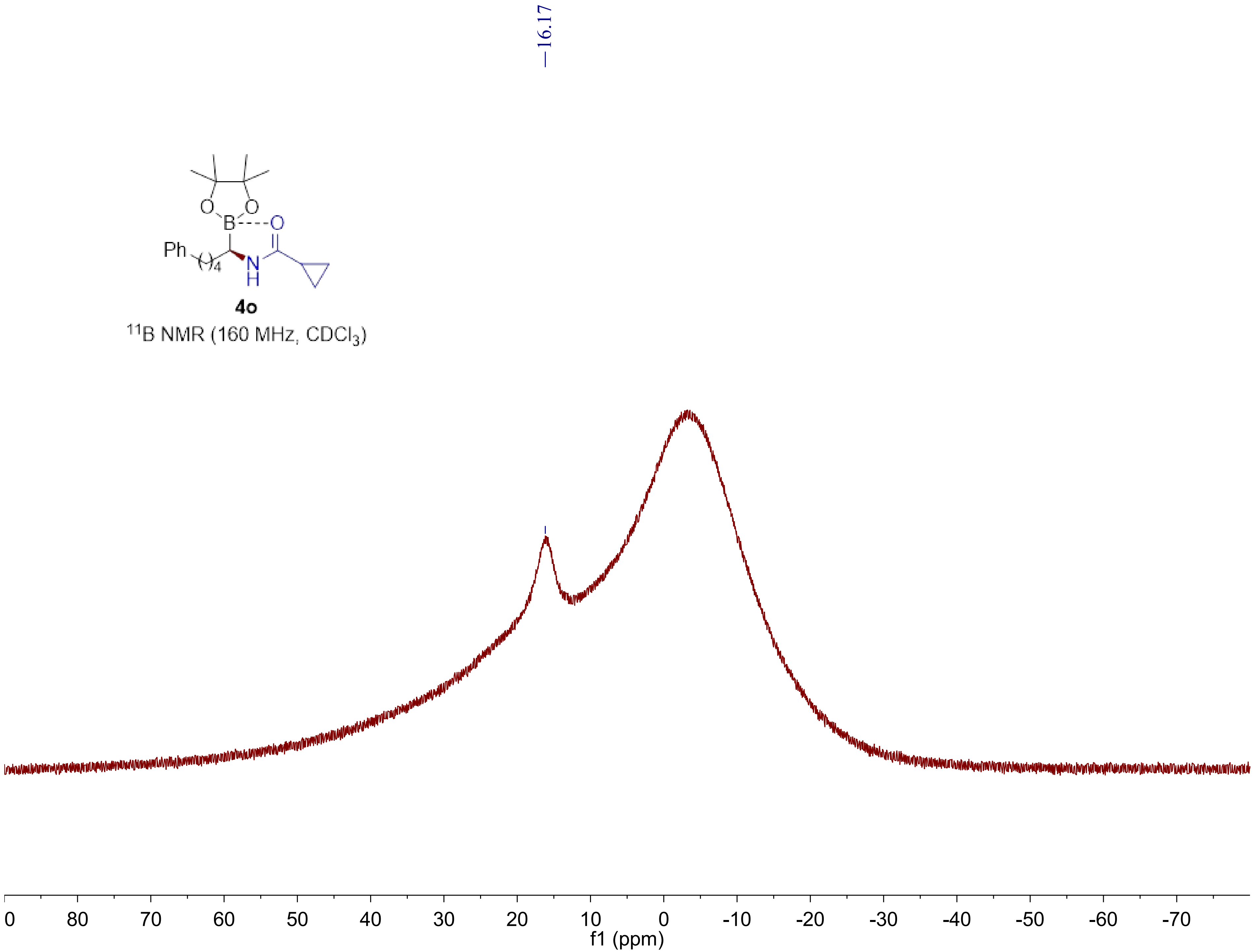

Supplementary Figure 100: ^{11}B NMR (160 MHz, $CDCl_3$) spectrum of **4o**.

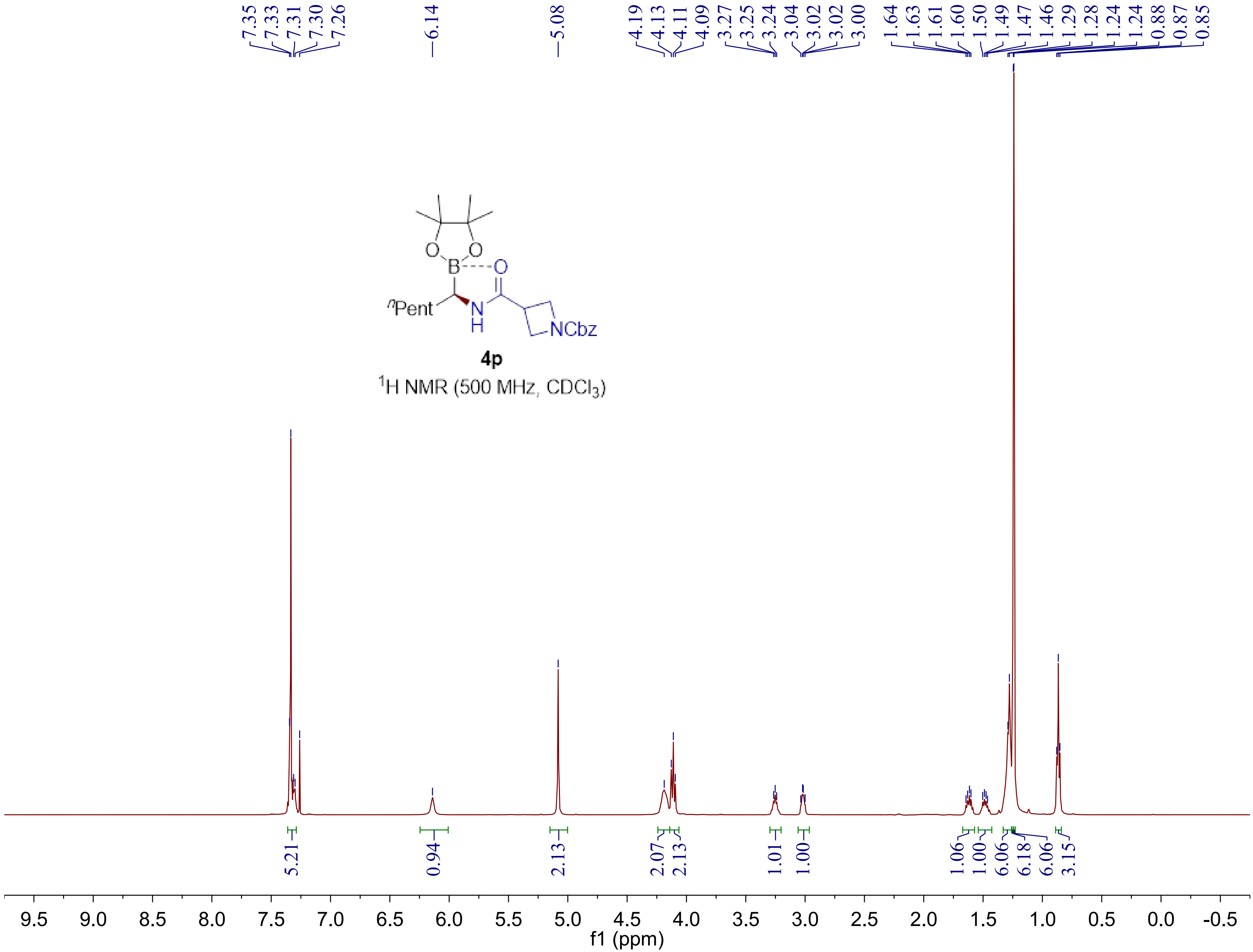

Supplementary Figure 101: ^{1}H NMR (500 MHz, $CDCl_3$) spectrum of **4p**.

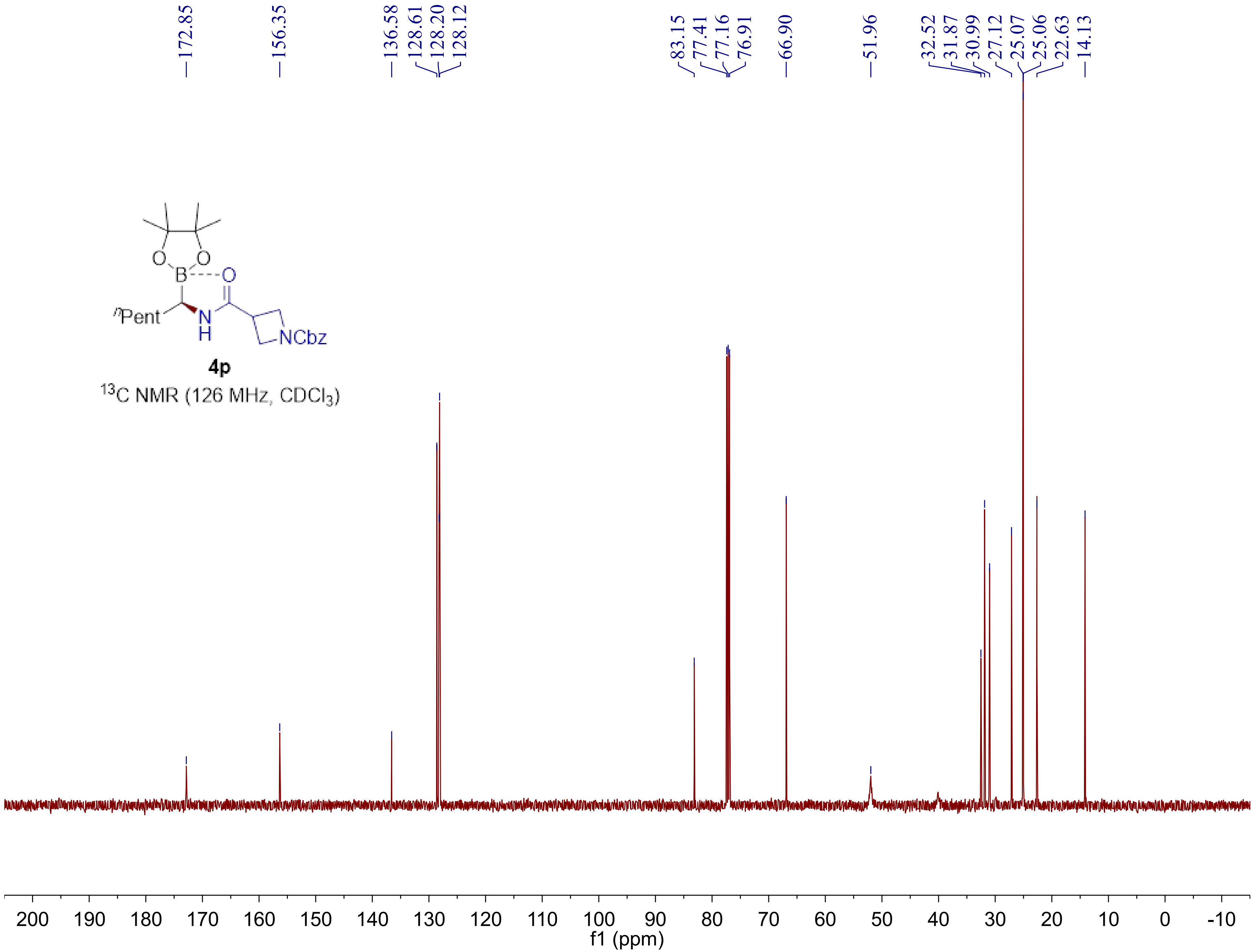

Supplementary Figure 102: ^{13}C NMR (126 MHz, $CDCl_3$) spectrum of **4p**.

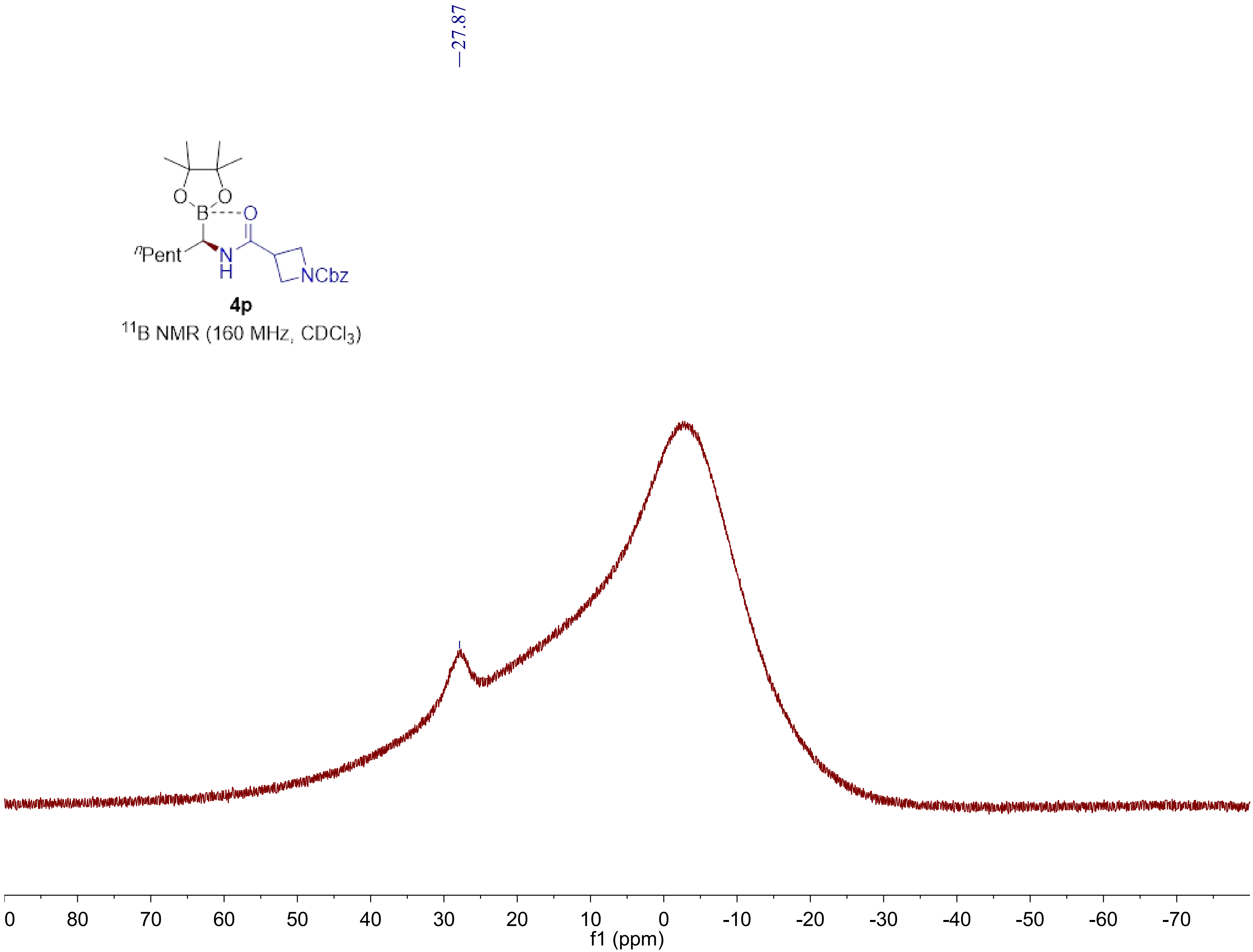

Supplementary Figure 103: ^{11}B NMR (160 MHz, $CDCl_3$) spectrum of **4p**.

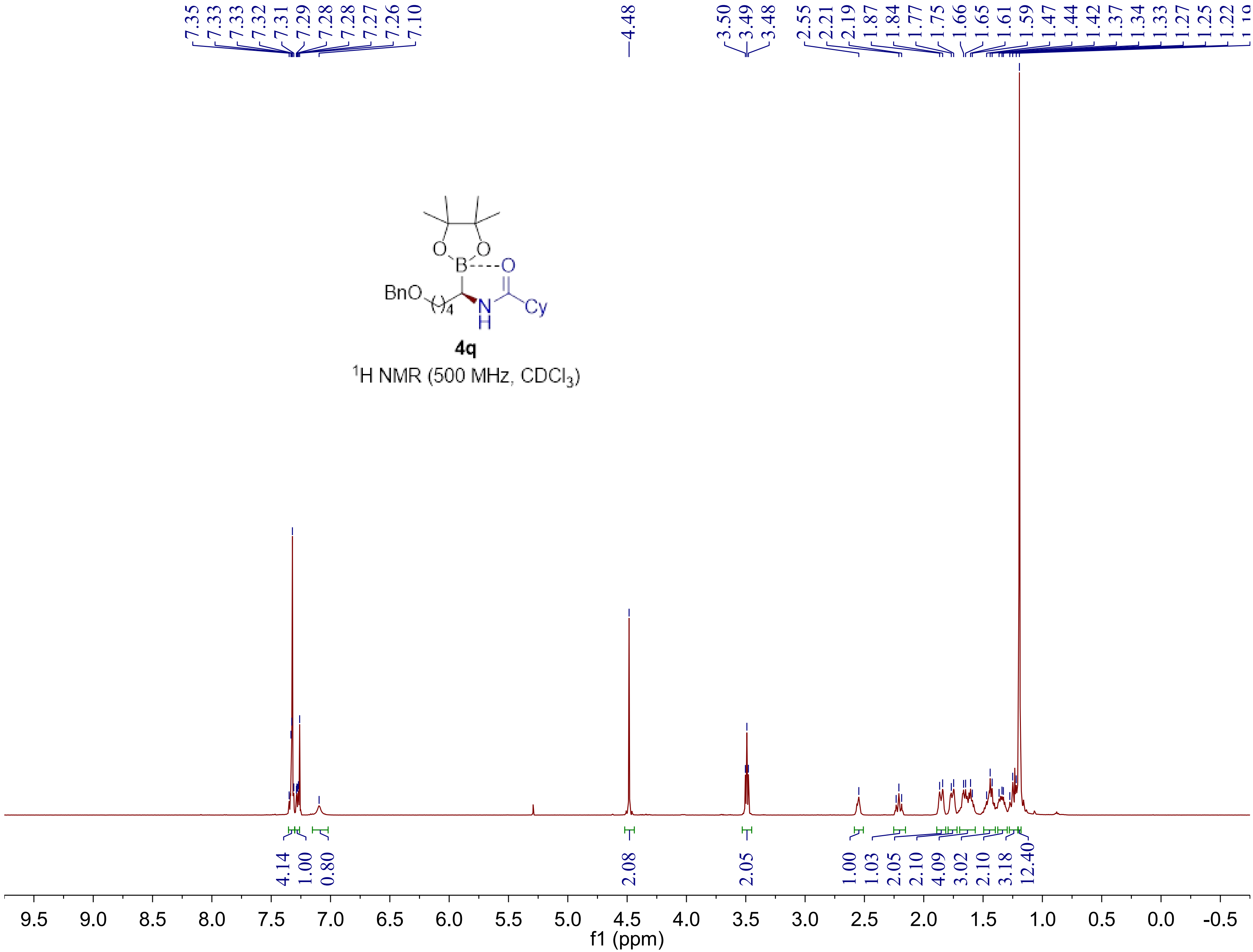

Supplementary Figure 104: ^{1}H NMR (500 MHz, $CDCl_3$) spectrum of **4q**.

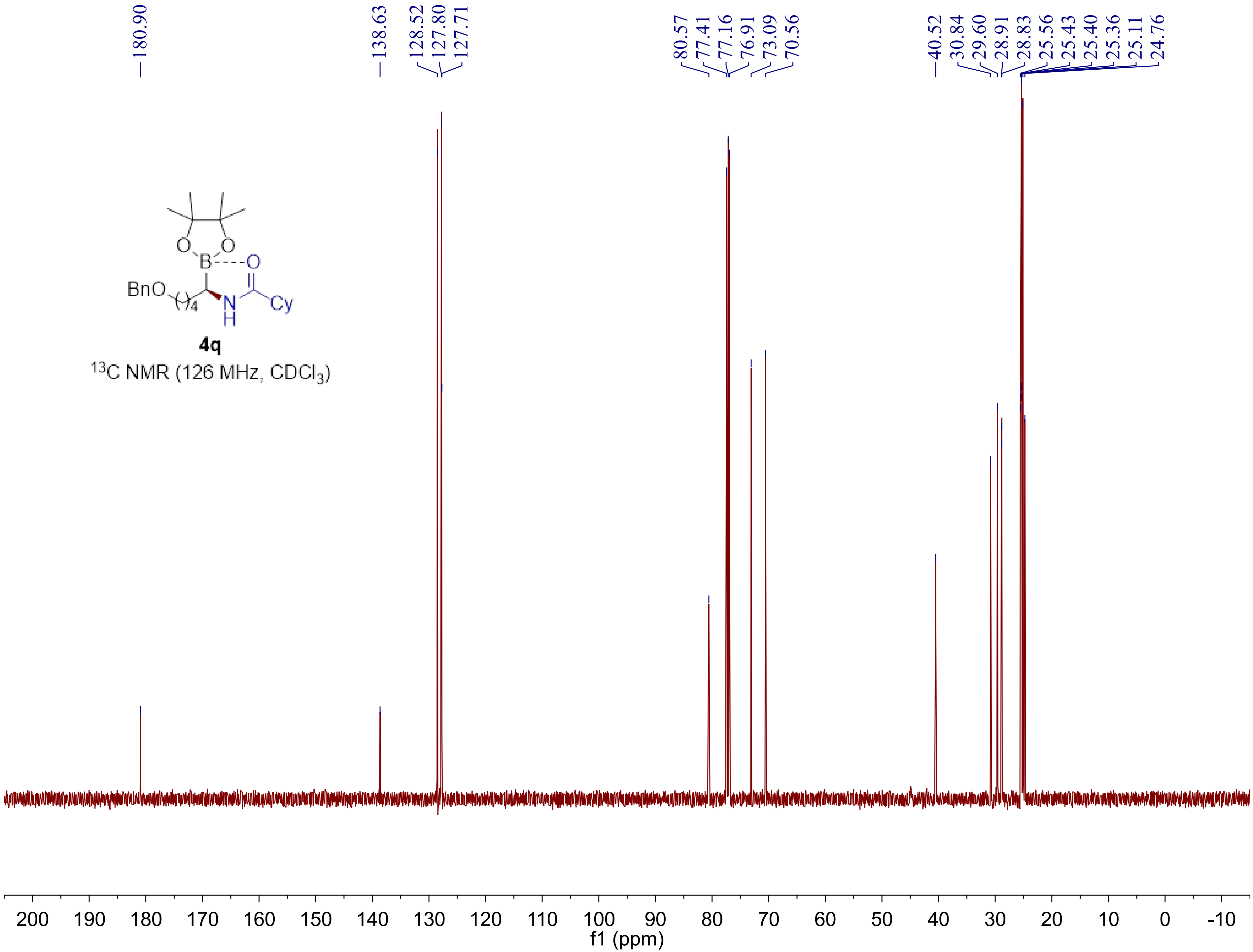

Supplementary Figure 105: ^{13}C NMR (126 MHz, $CDCl_3$) spectrum of **4q**.

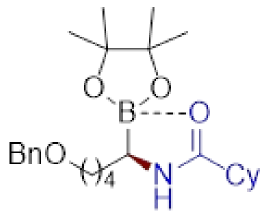

4q

^{11}B NMR (160 MHz, $CDCl_3$)

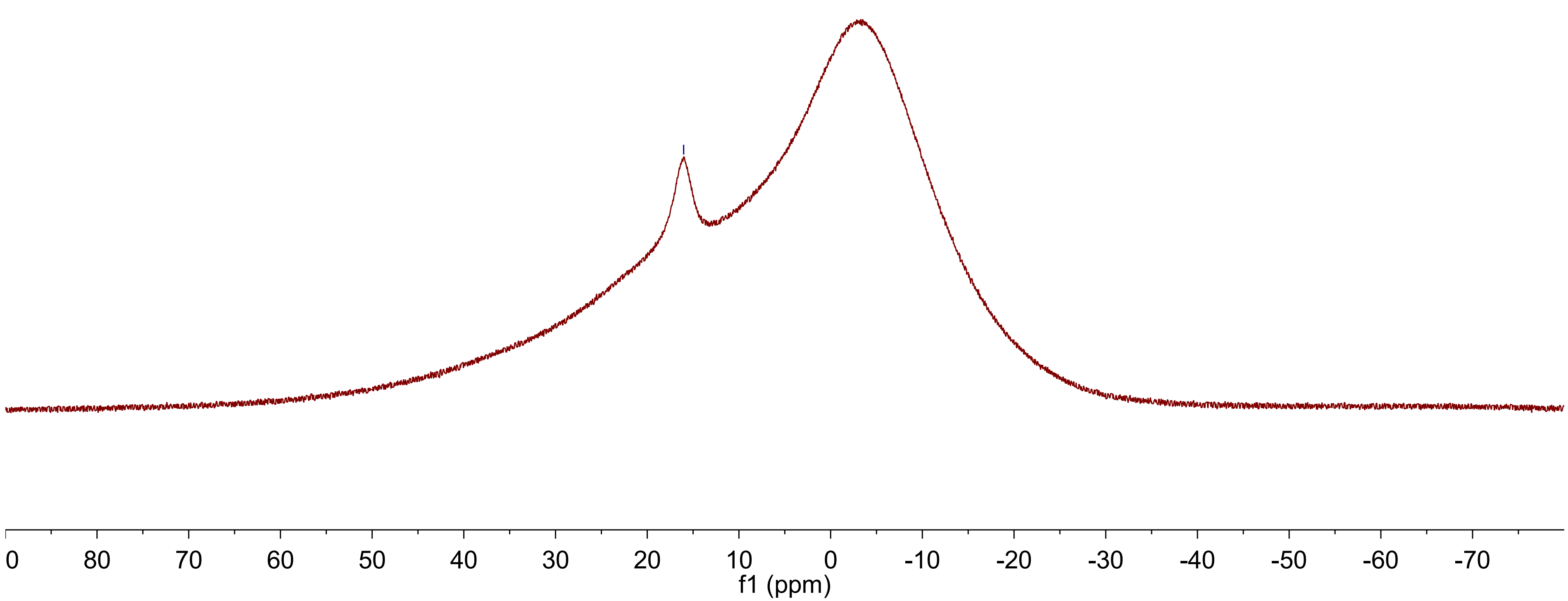

Supplementary Figure 106: ^{11}B NMR (160 MHz, $CDCl_3$) spectrum of **4q**.

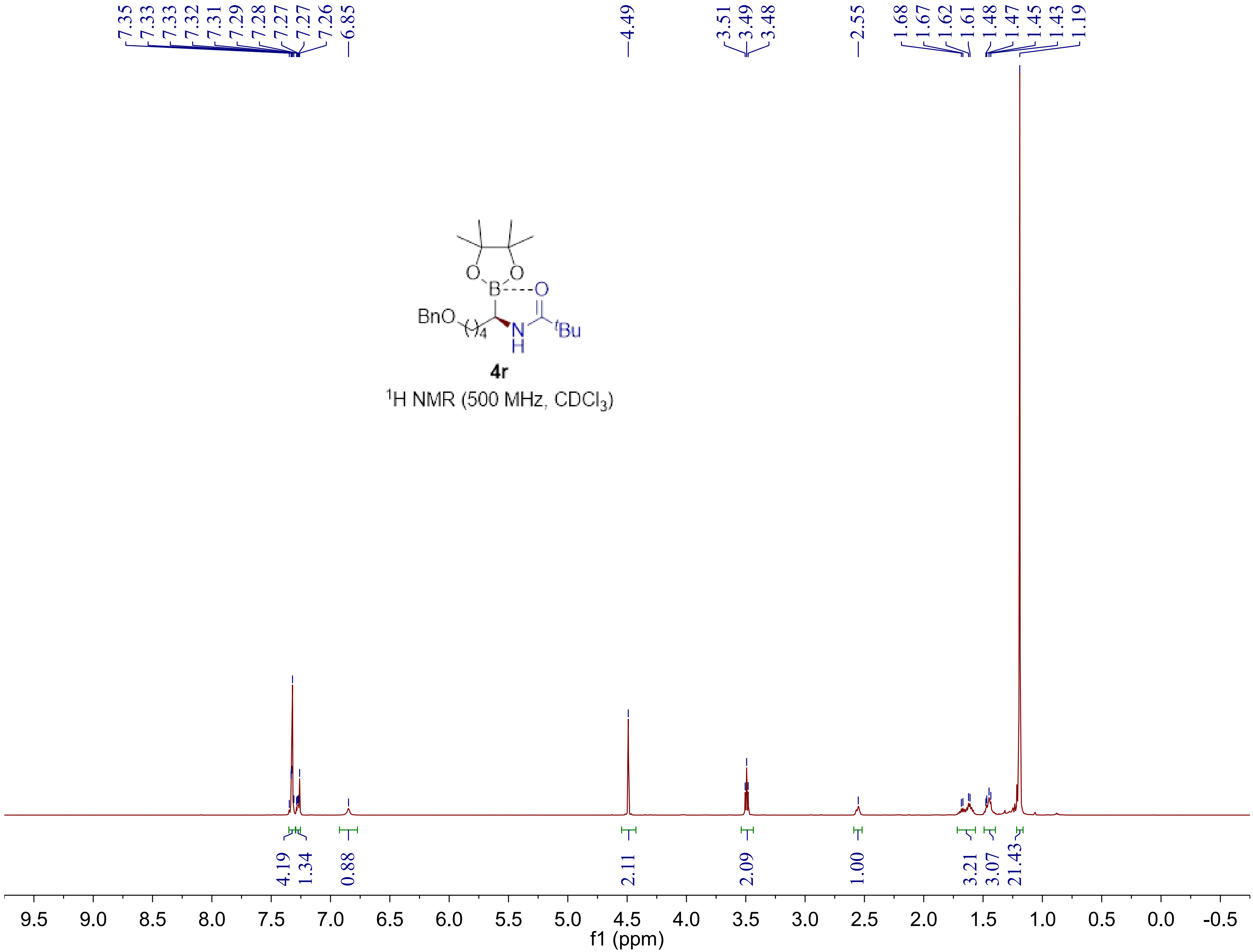

Supplementary Figure 107: ^{1}H NMR (500 MHz, $CDCl_3$) spectrum of **4r**.

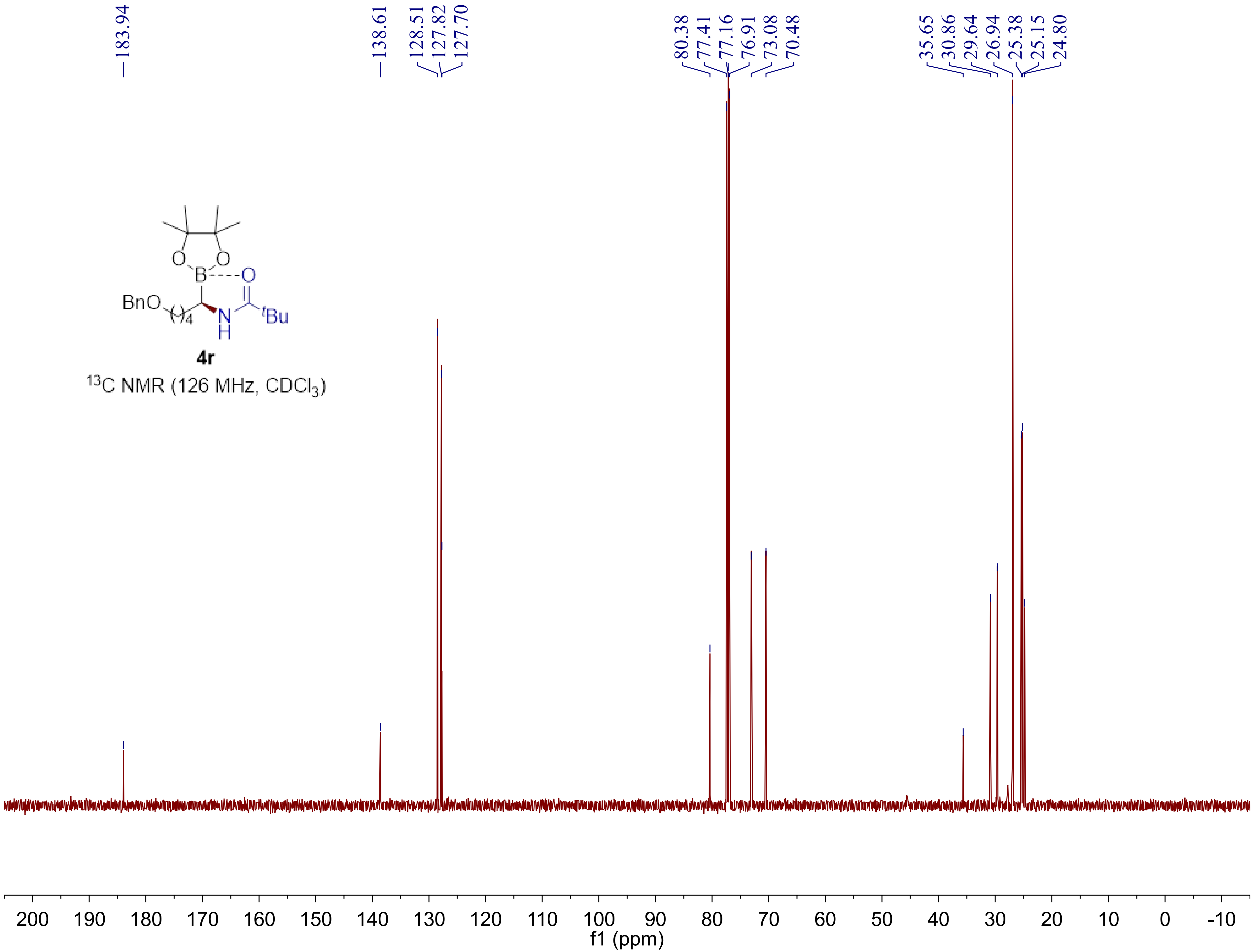

Supplementary Figure 108: ^{13}C NMR (126 MHz, $CDCl_3$) spectrum of **4r**.

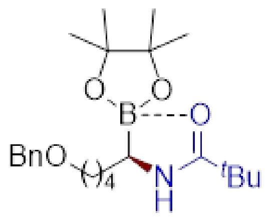

4r

^{11}B NMR (160 MHz, $CDCl_3$)

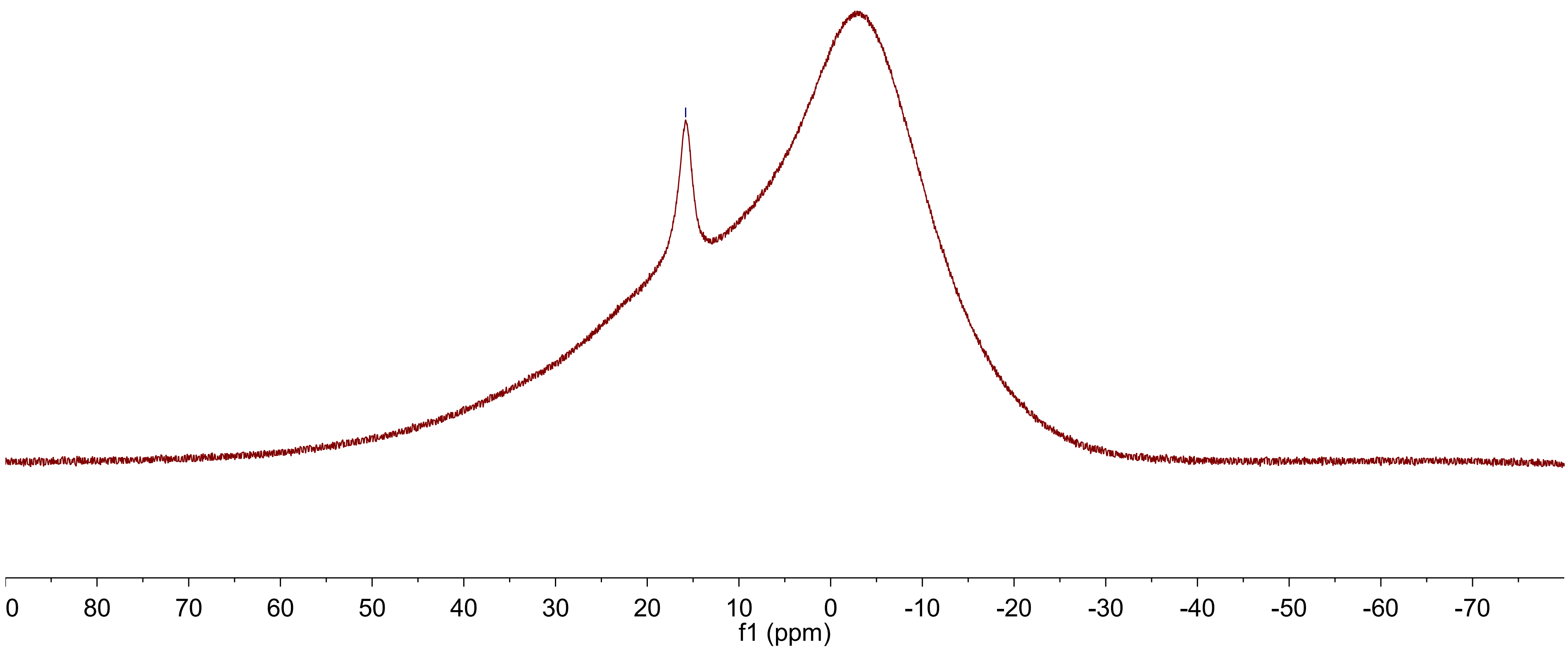

Supplementary Figure 109: ^{11}B NMR (160 MHz, $CDCl_3$) spectrum of **4r**.

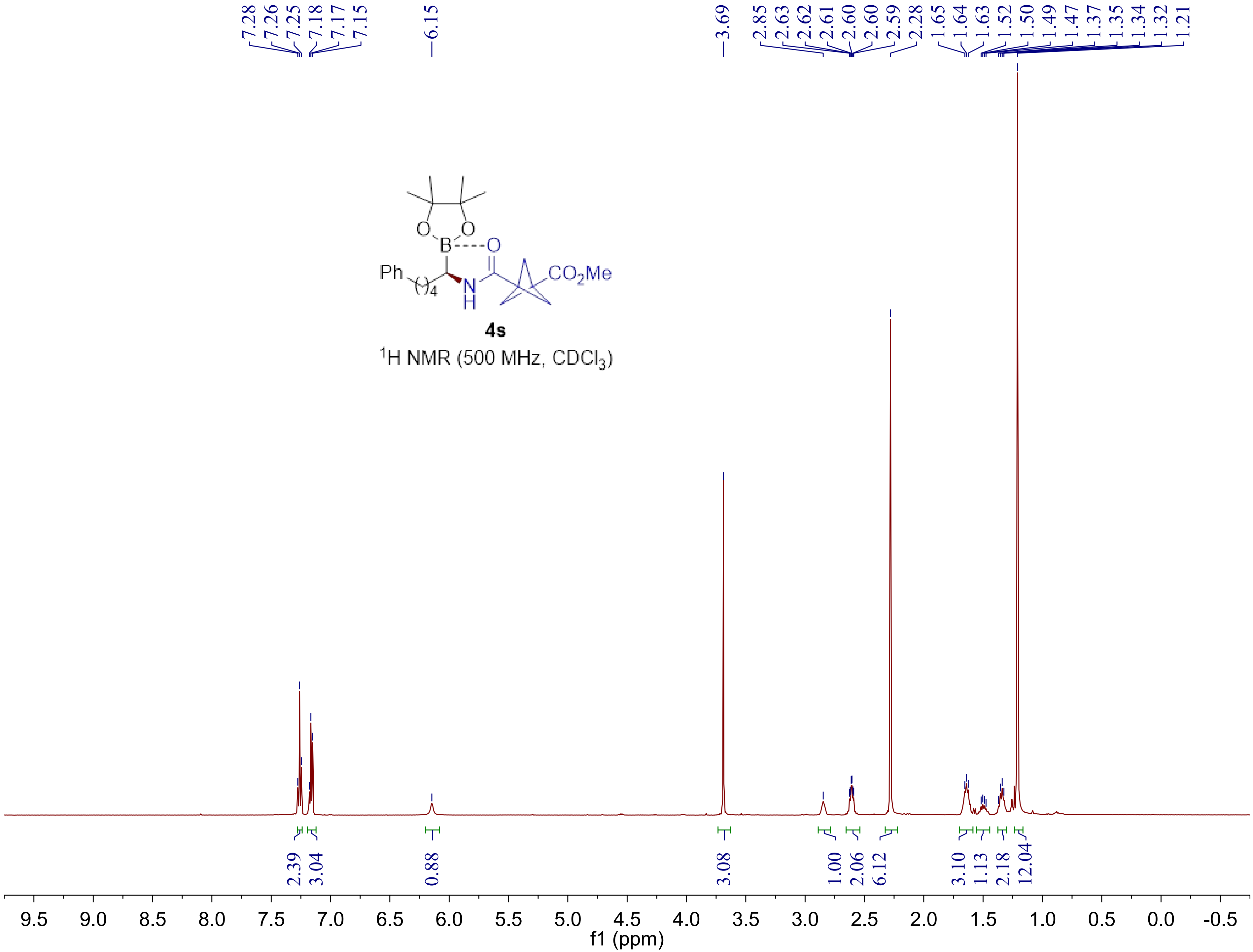

Supplementary Figure 110: ^{1}H NMR (500 MHz, $CDCl_3$) spectrum of **4s**.

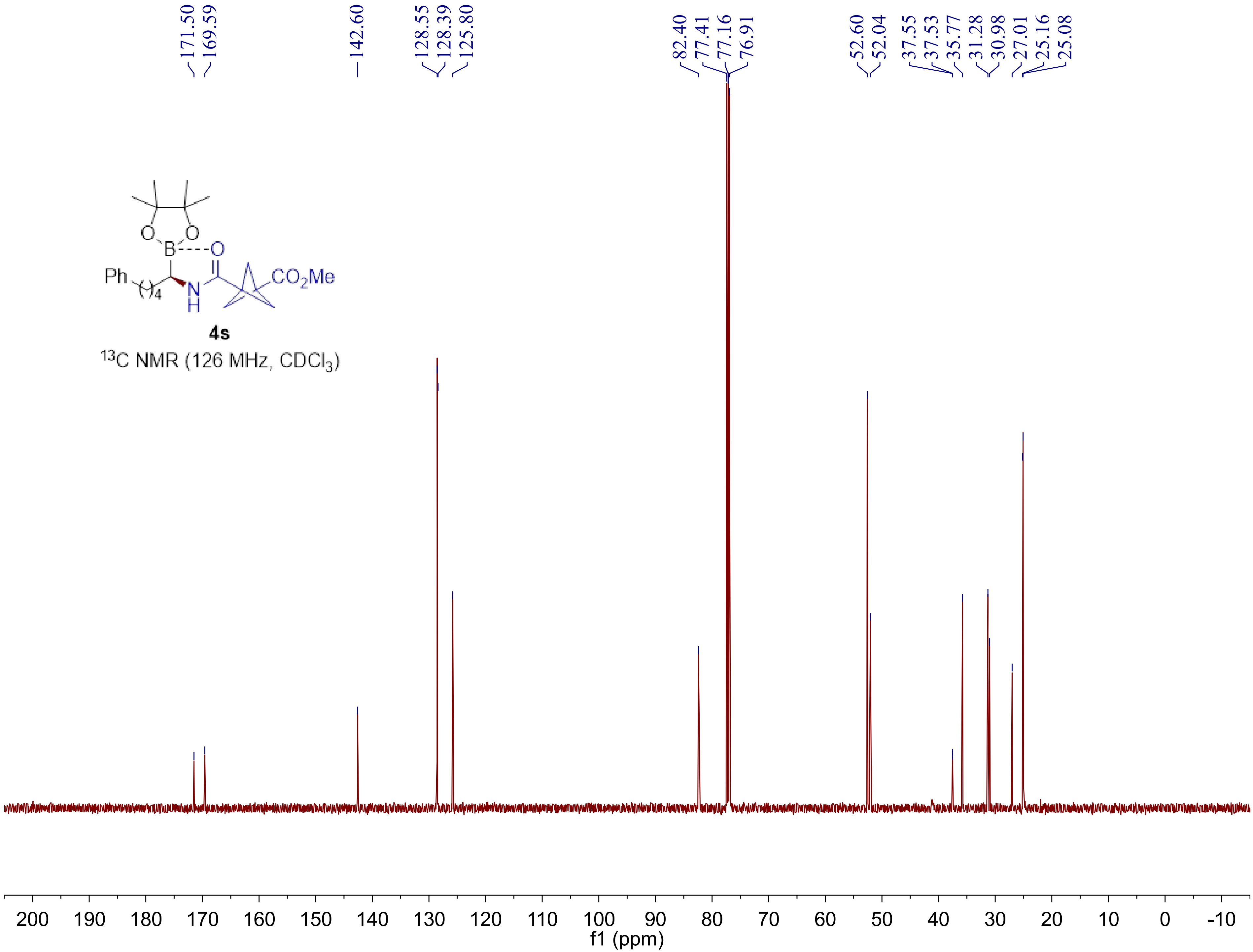

Supplementary Figure 111: ^{13}C NMR (126 MHz, $CDCl_3$) spectrum of **4s**.

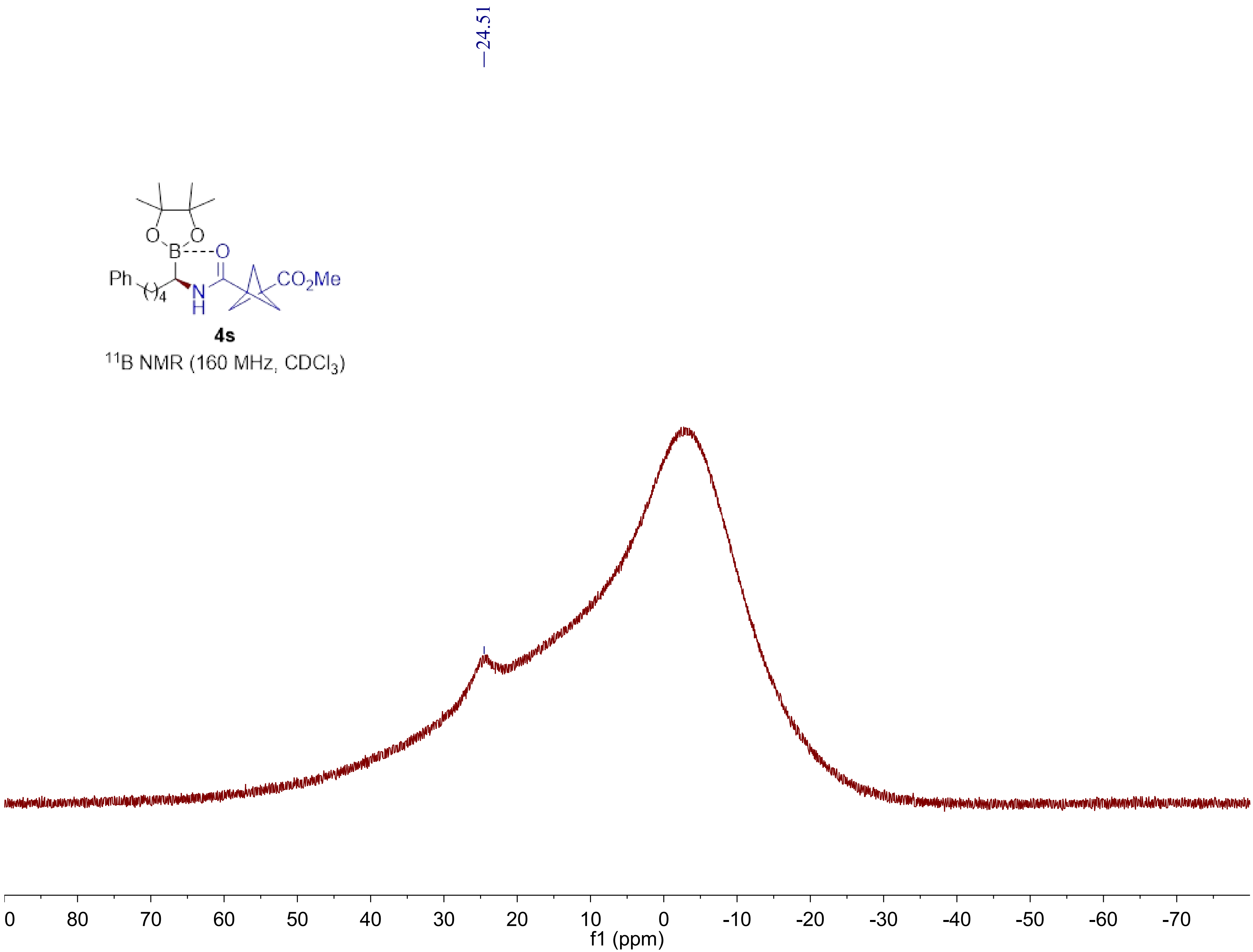

Supplementary Figure 112: ^{11}B NMR (160 MHz, $CDCl_3$) spectrum of **4s**.

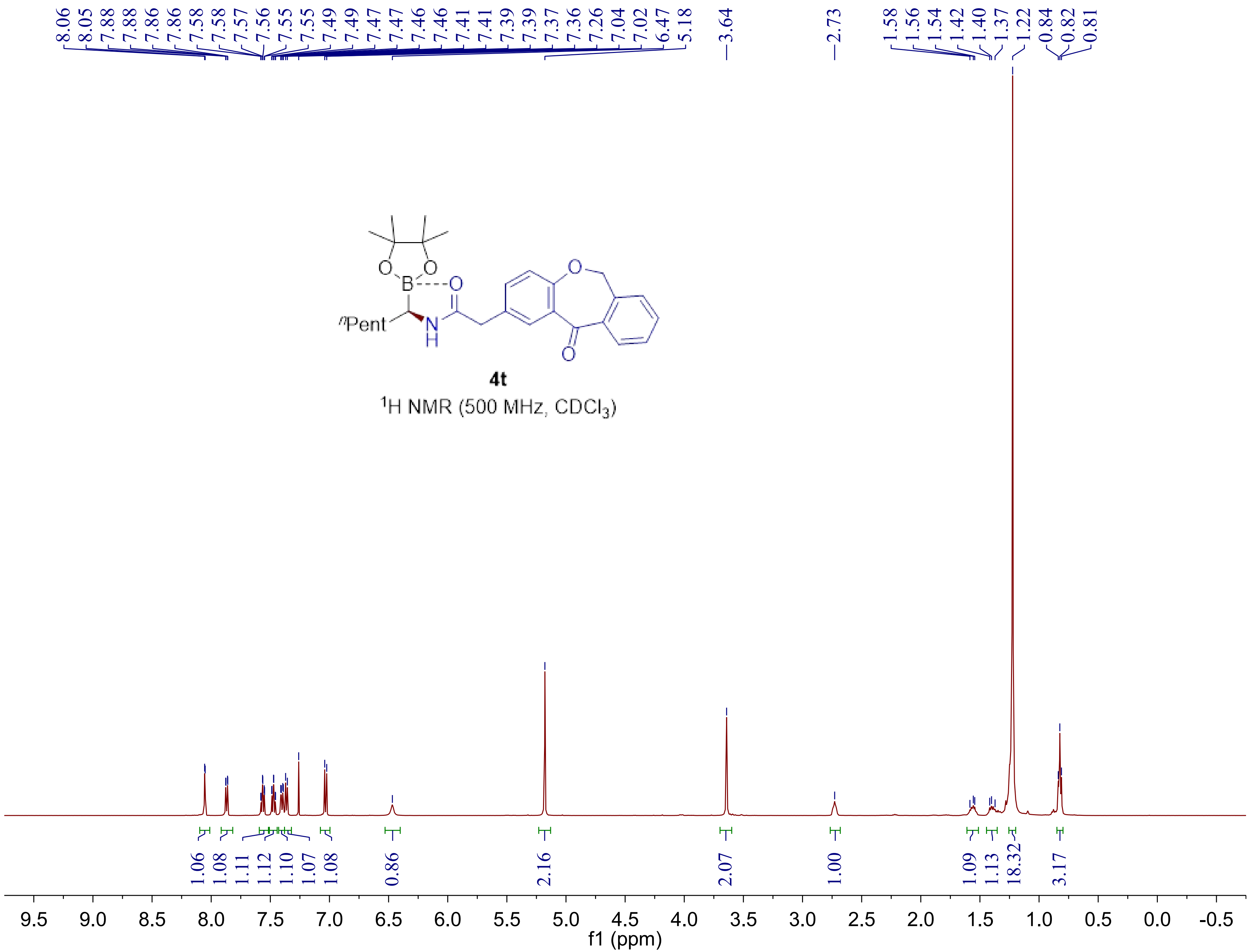

Supplementary Figure 113: ^{1}H NMR (500 MHz, $CDCl_3$) spectrum of **4t**.

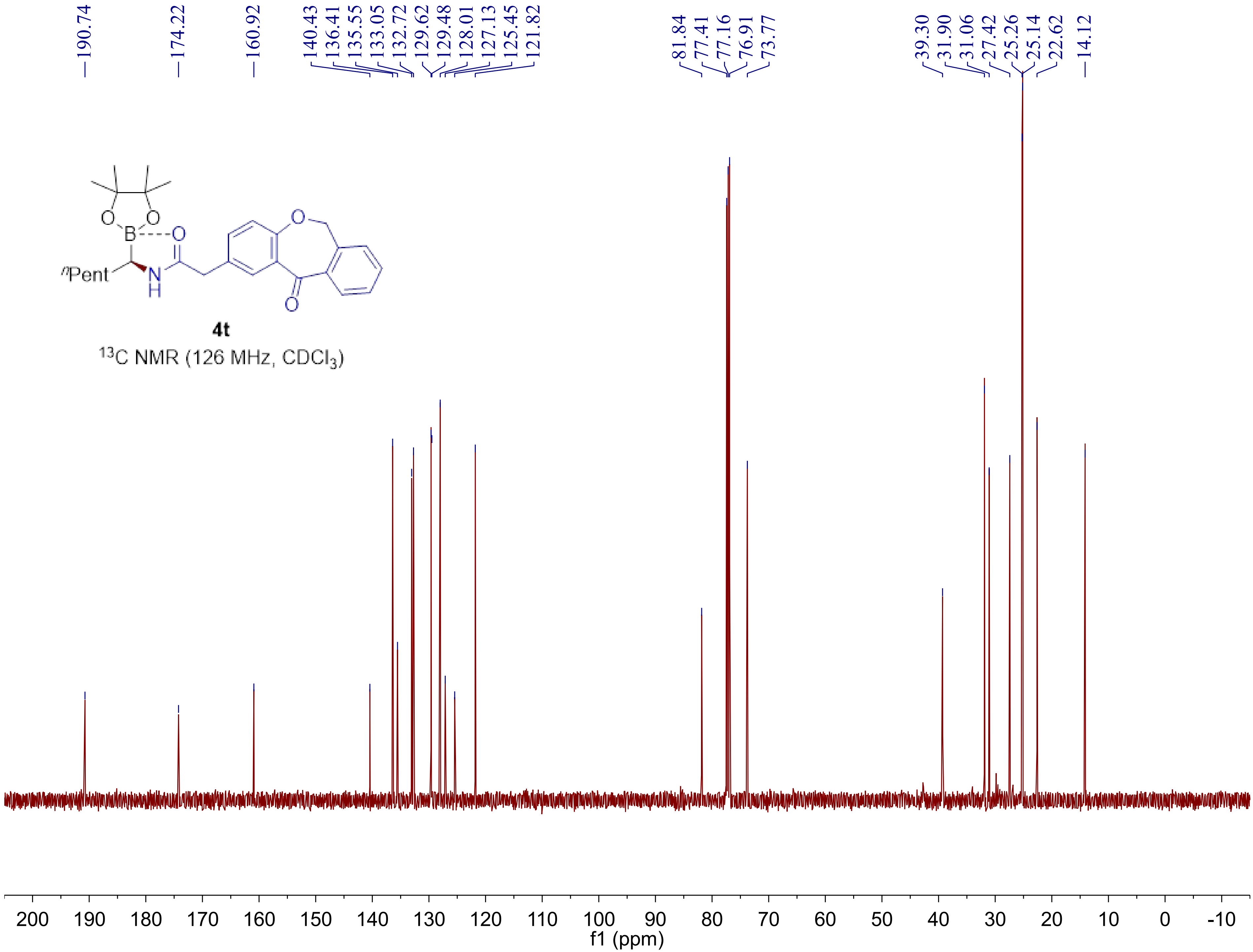

Supplementary Figure 114: ^{13}C NMR (126 MHz, $CDCl_3$) spectrum of **4t**.

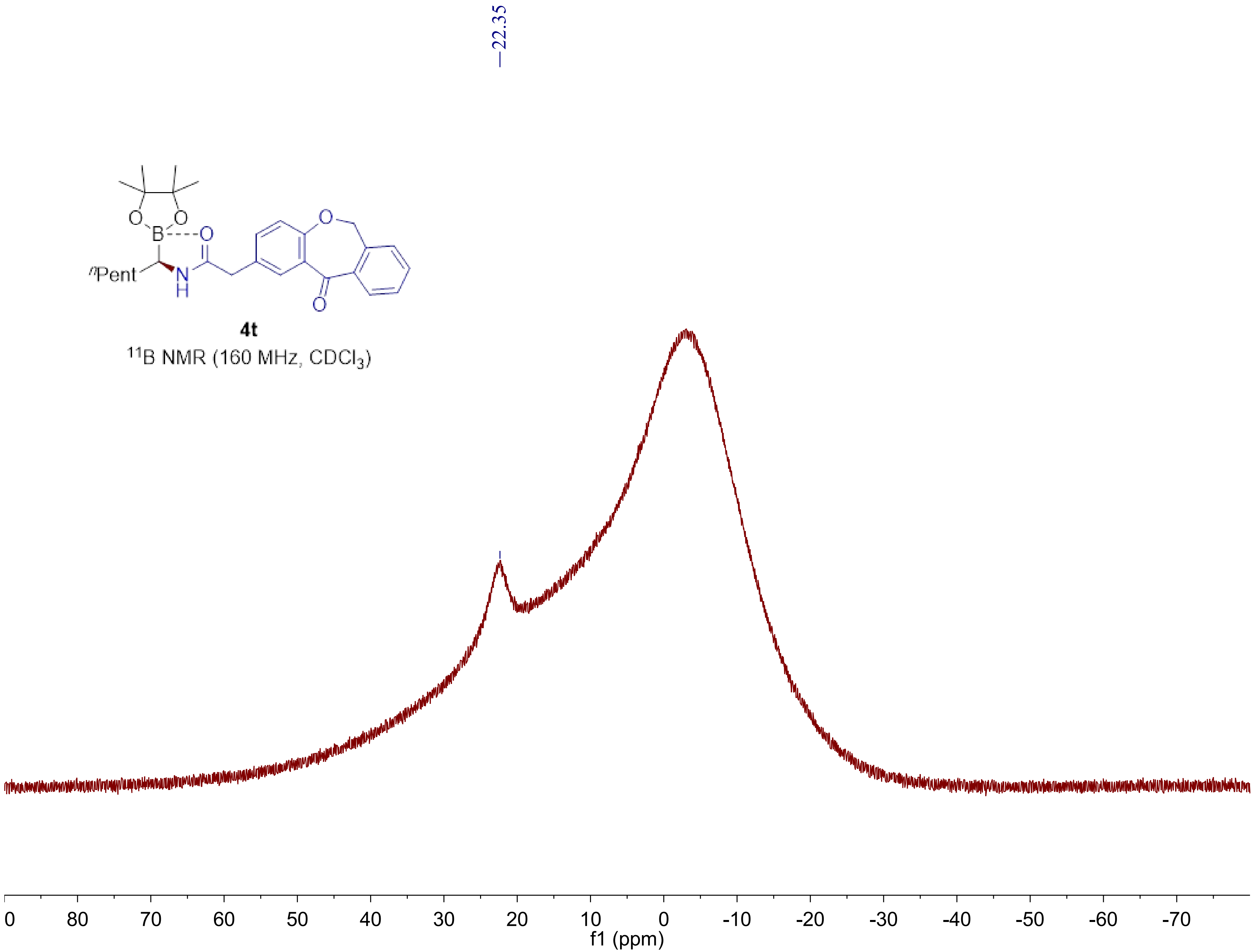

Supplementary Figure 115: ^{11}B NMR (160 MHz, $CDCl_3$) spectrum of **4t**.

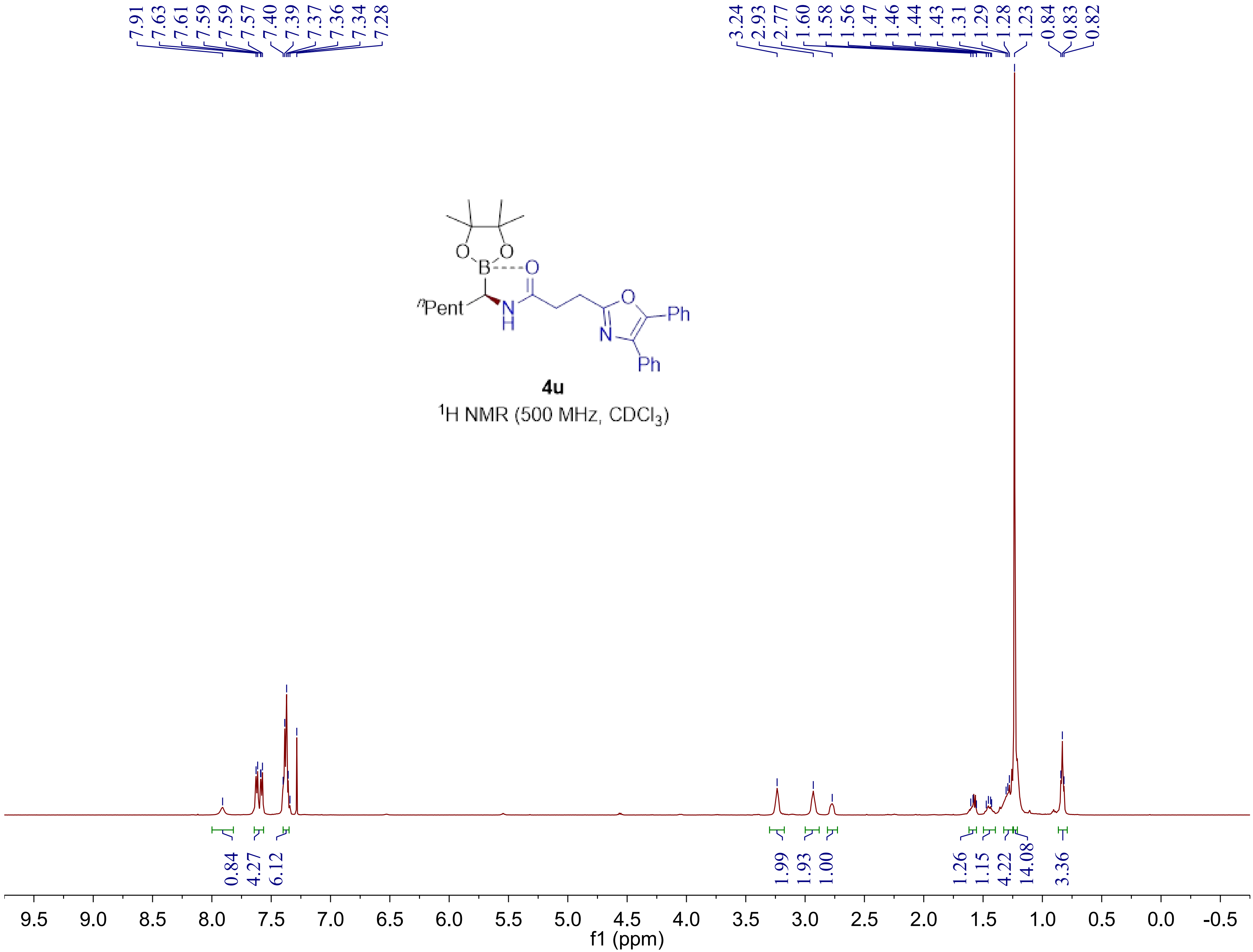

Supplementary Figure 116: ^{1}H NMR (500 MHz, $CDCl_3$) spectrum of **4u**.

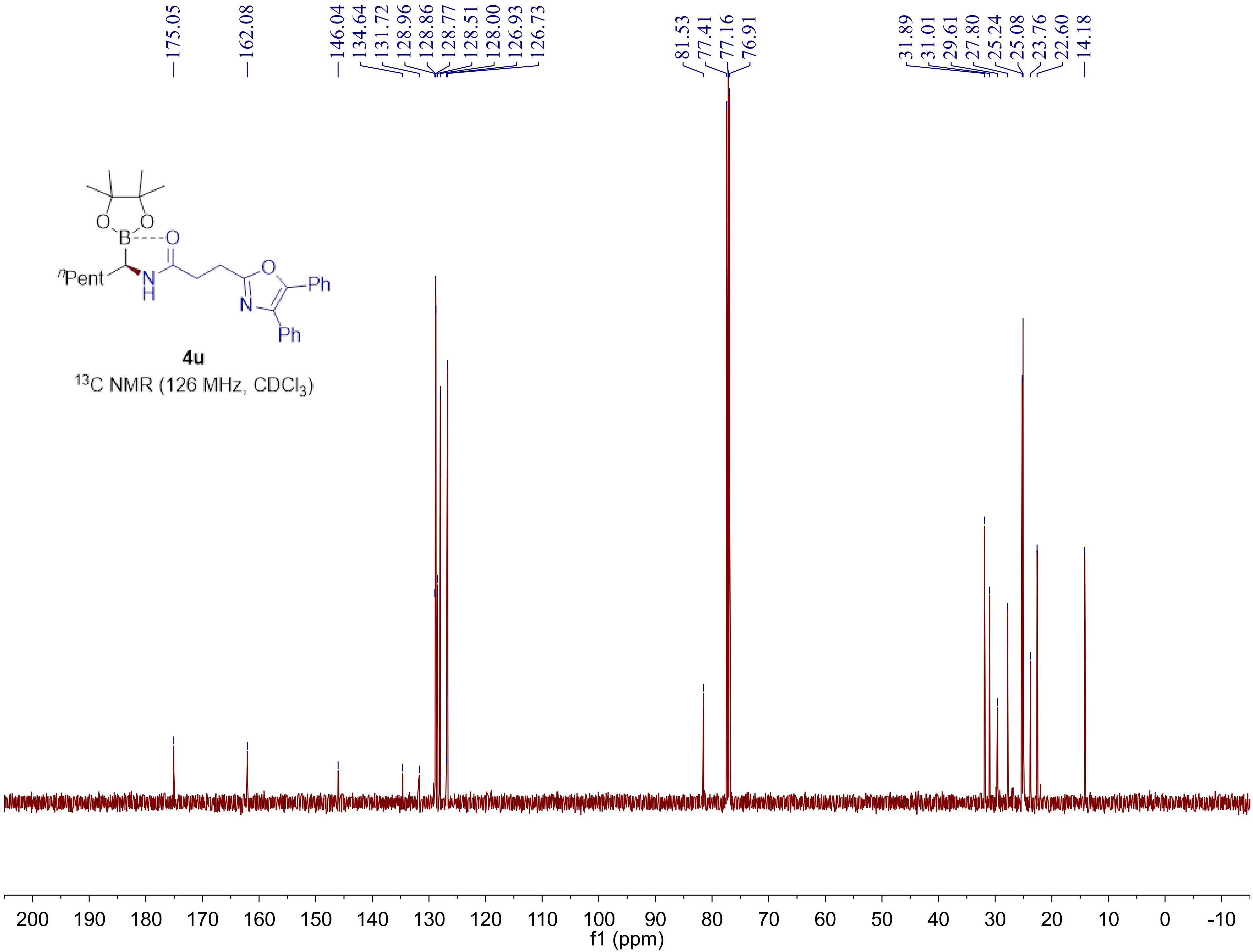

Supplementary Figure 117: ^{13}C NMR (126 MHz, CDCl$_3$) spectrum of **4u**.

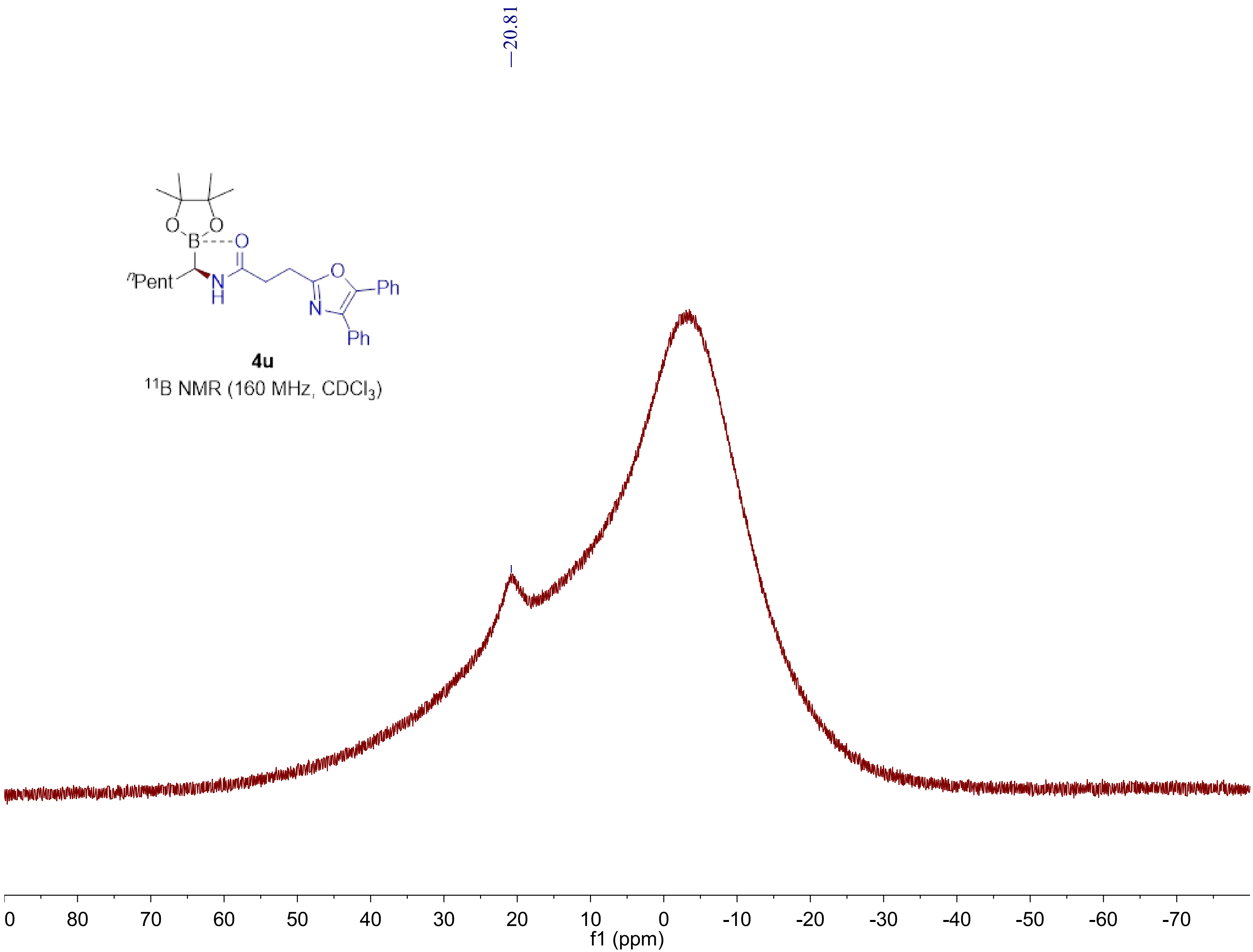

Supplementary Figure 118: ^{11}B NMR (160 MHz, $CDCl_3$) spectrum of **4u**.

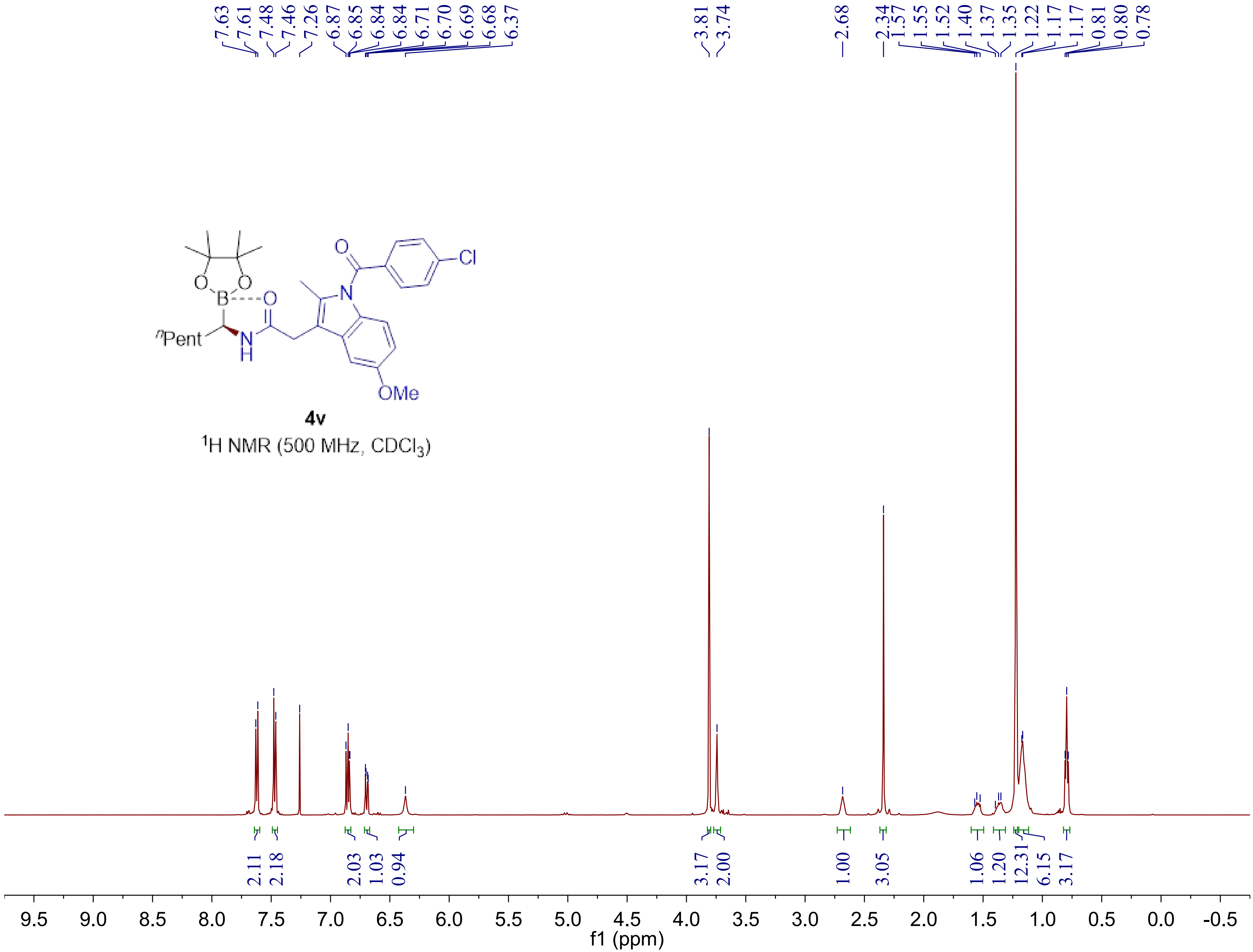

Supplementary Figure 119: ^{1}H NMR (500 MHz, $CDCl_3$) spectrum of **4v**.

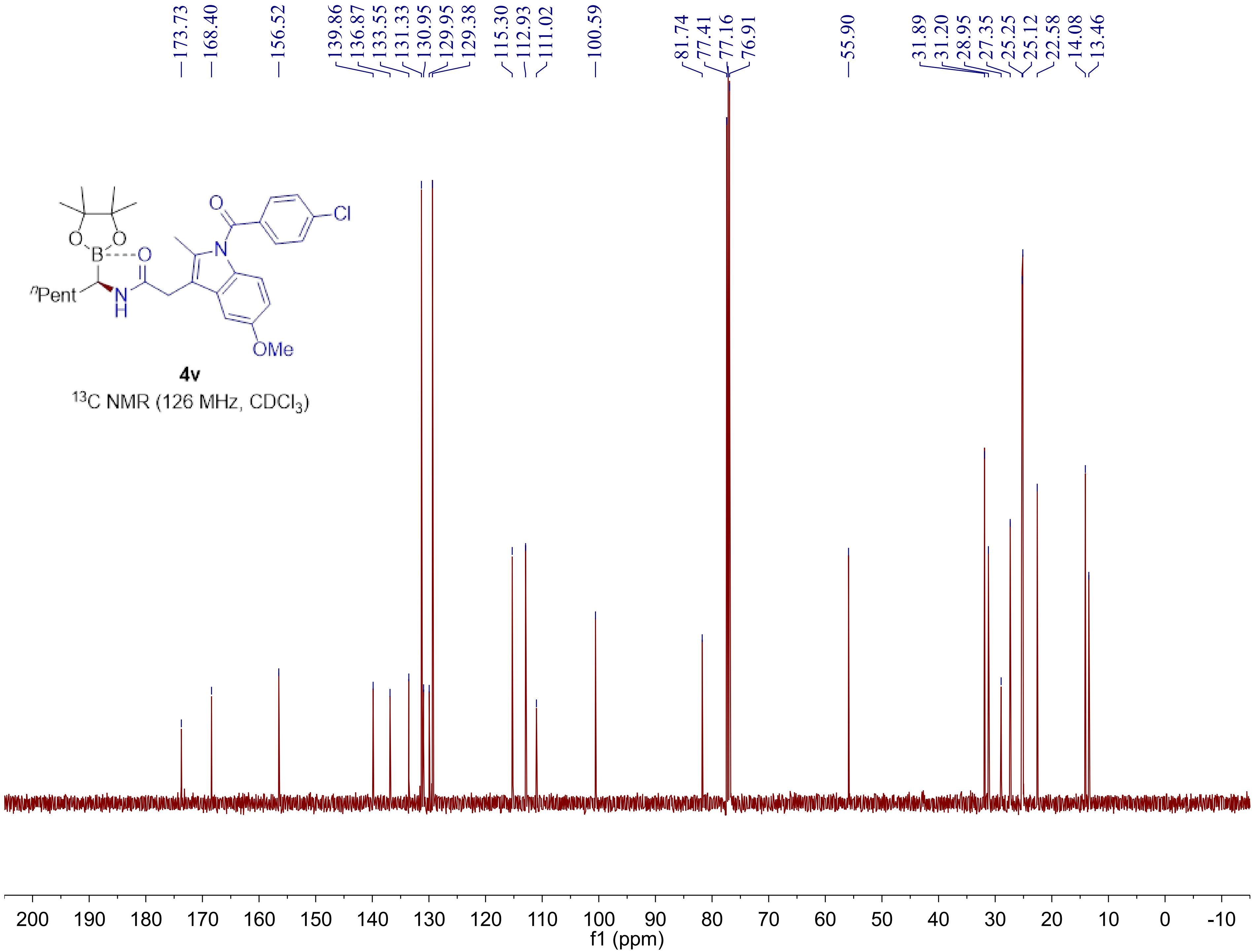

Supplementary Figure 120: ^{13}C NMR (126 MHz, $CDCl_3$) spectrum of **4v**.

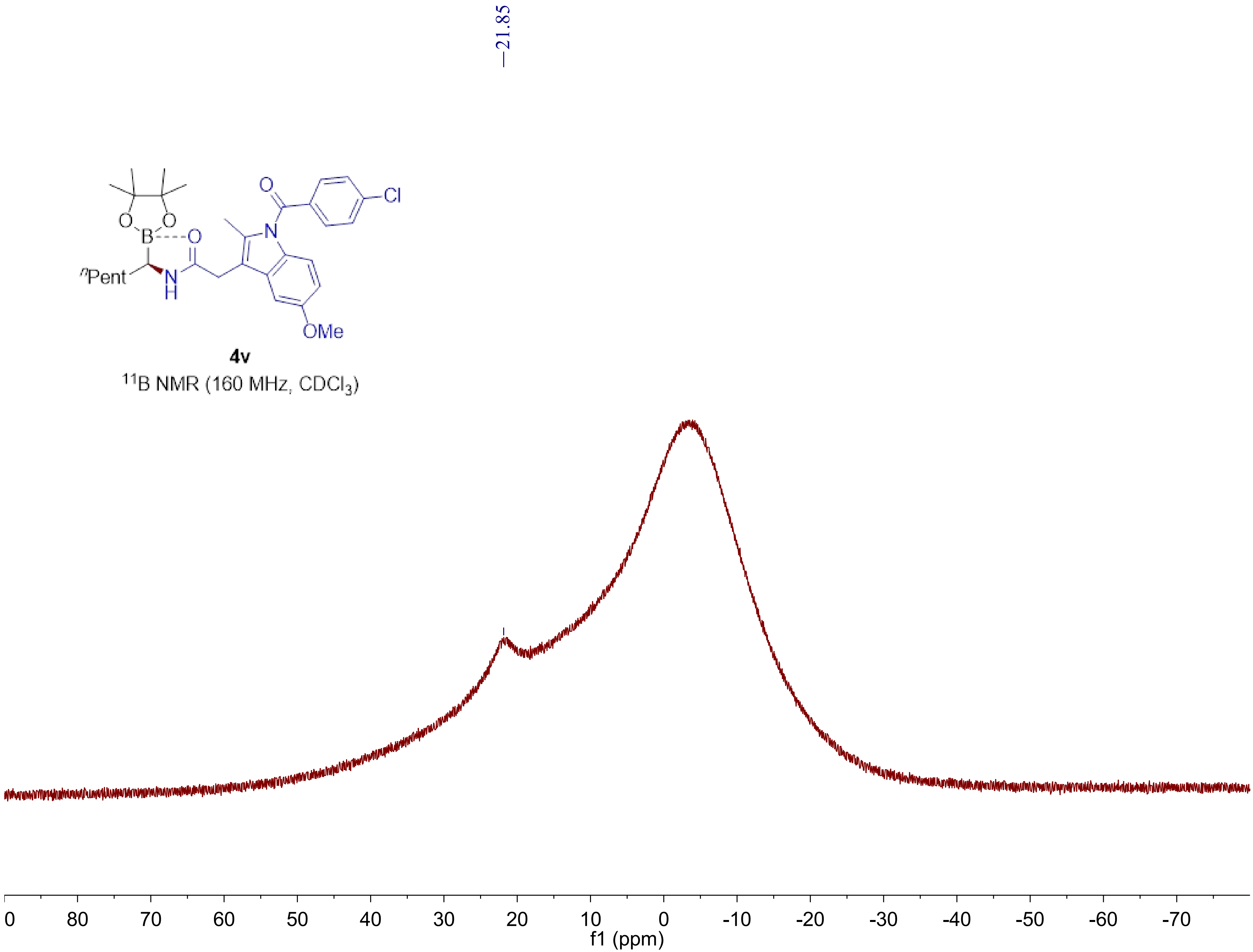

Supplementary Figure 121: ^{11}B NMR (160 MHz, $CDCl_3$) spectrum of **4v**.

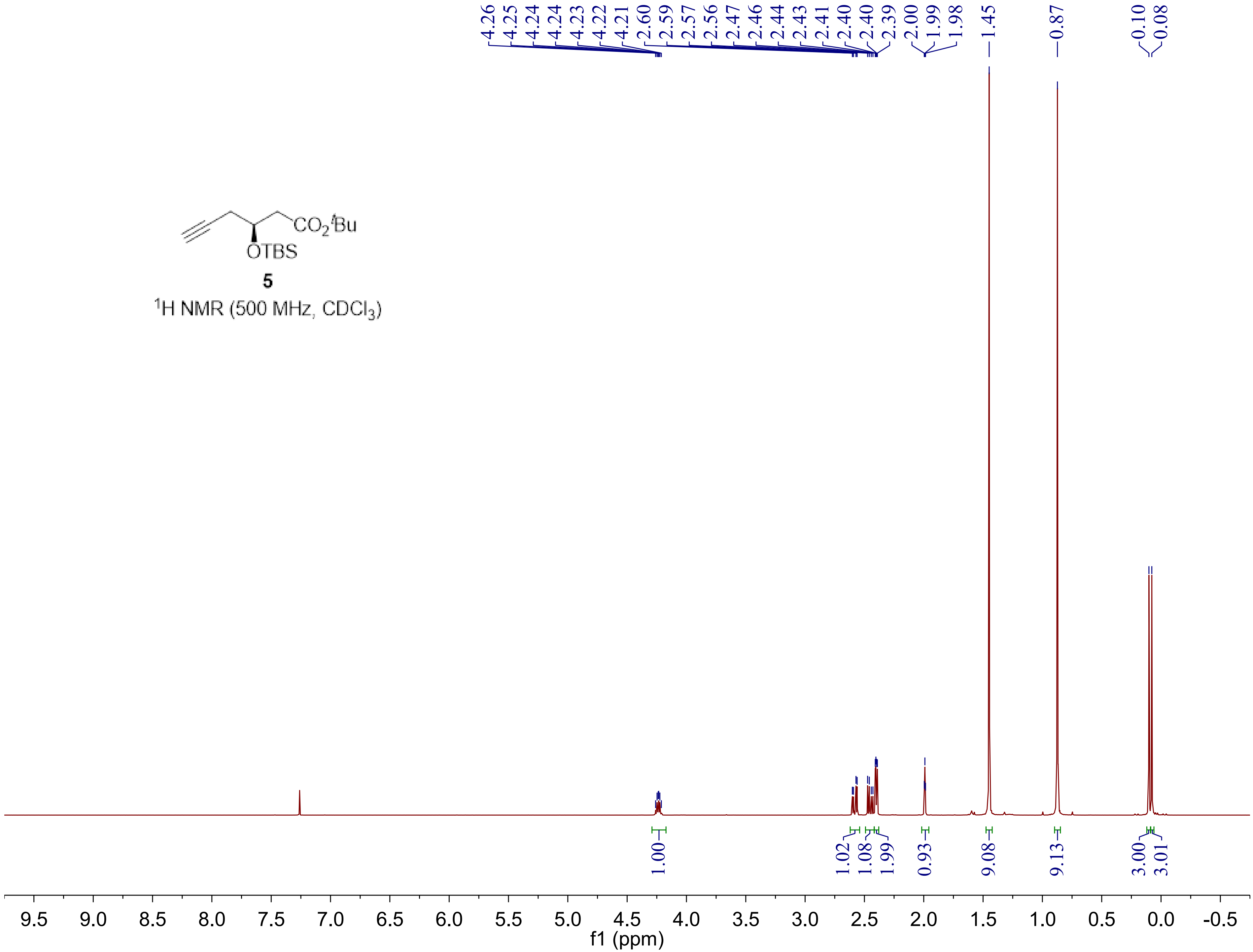

Supplementary Figure 122: ^{1}H NMR (500 MHz, $CDCl_3$) spectrum of **5**.

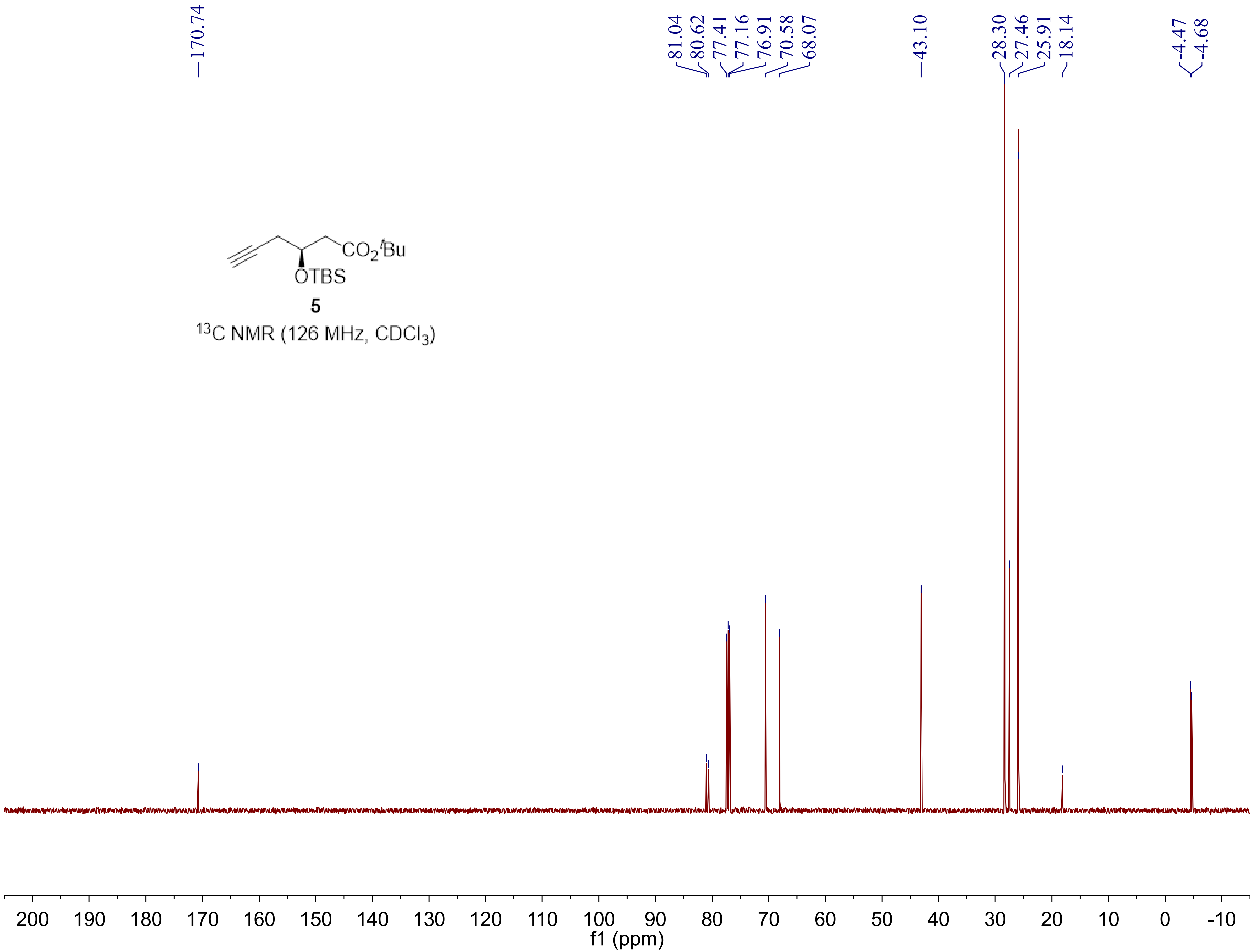

Supplementary Figure 123: ^{13}C NMR (126 MHz, $CDCl_3$) spectrum of **5**.

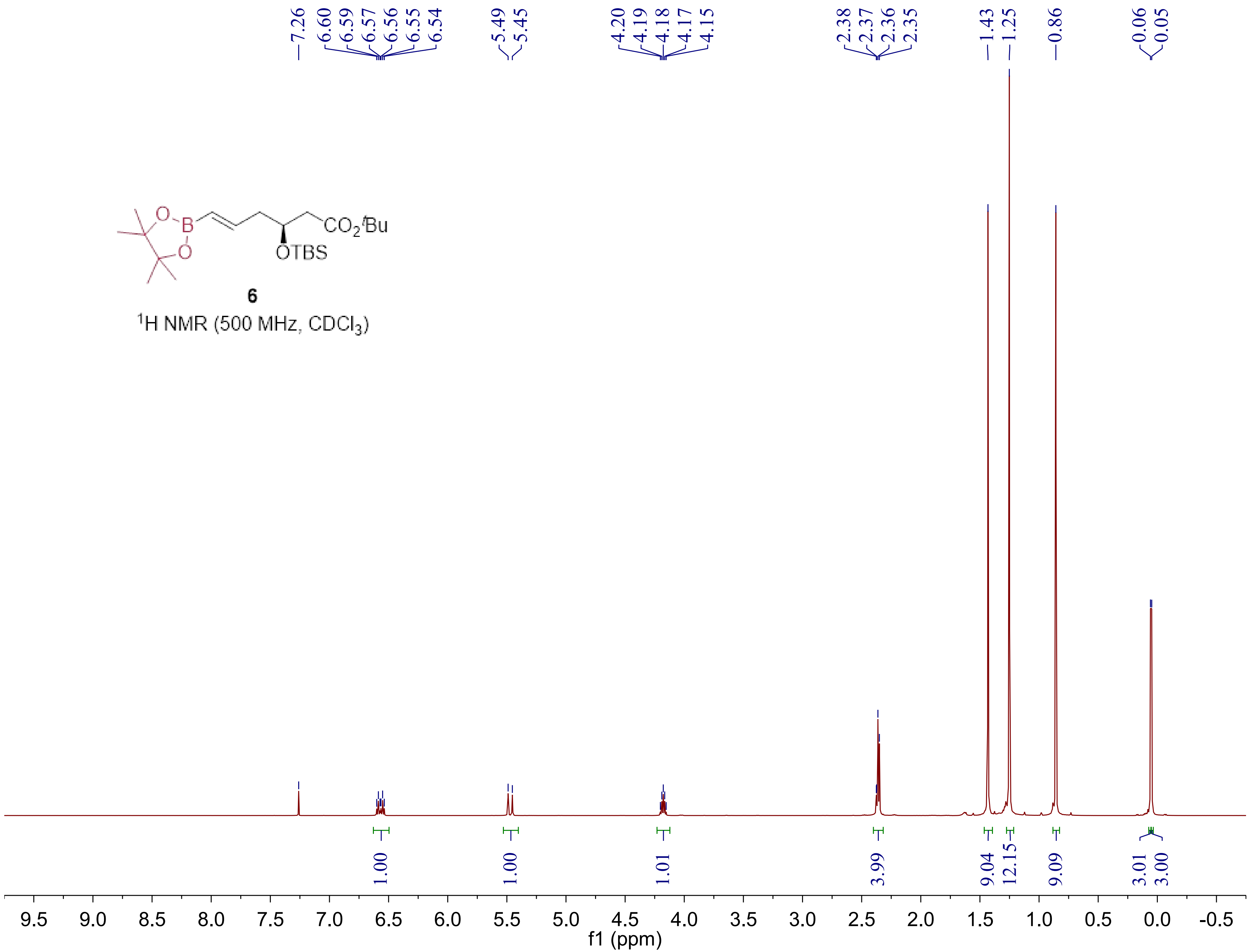

Supplementary Figure 124: ^{1}H NMR (500 MHz, $CDCl_3$) spectrum of **6**.

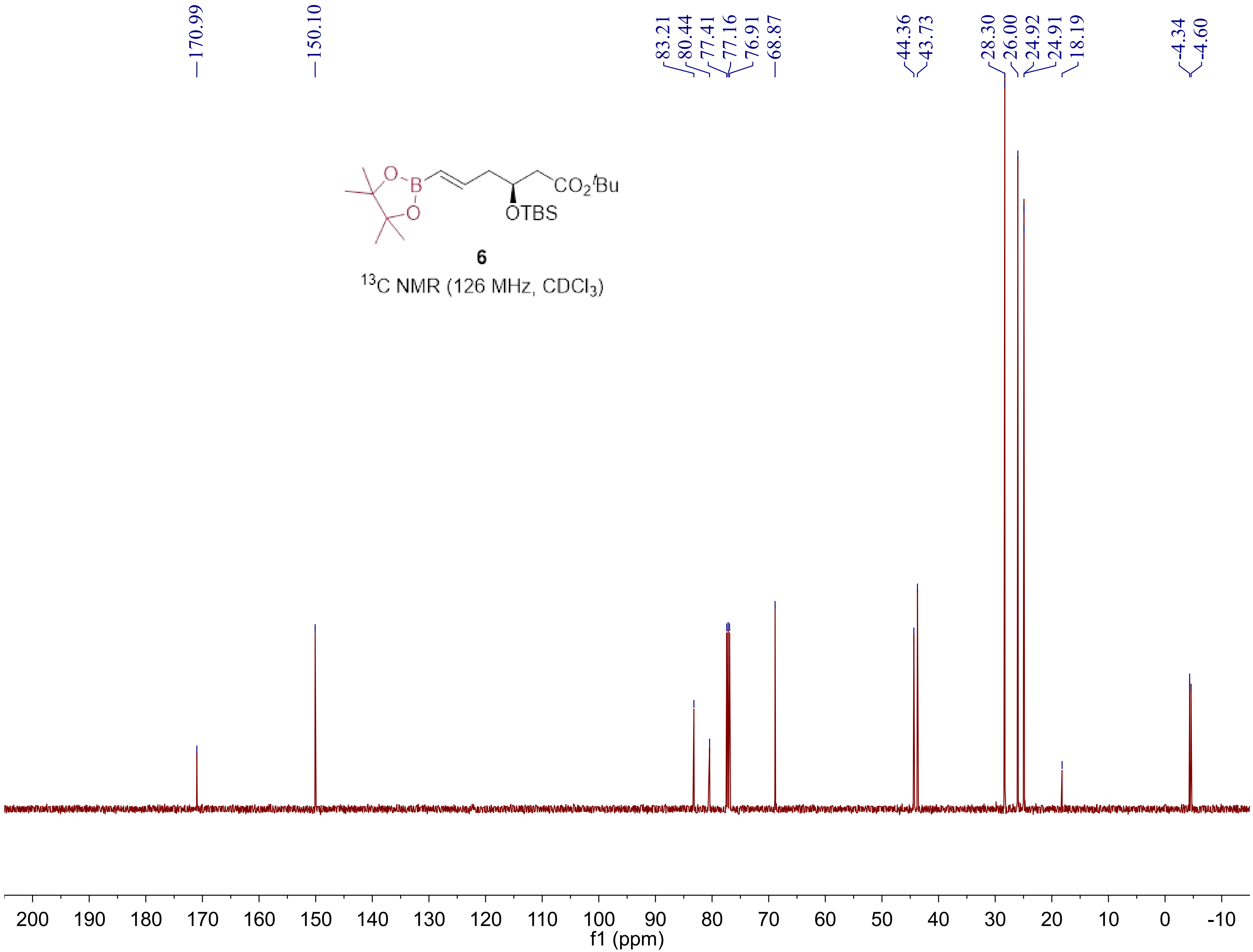

Supplementary Figure 125: ^{13}C NMR (126 MHz, $CDCl_3$) spectrum of **6**.

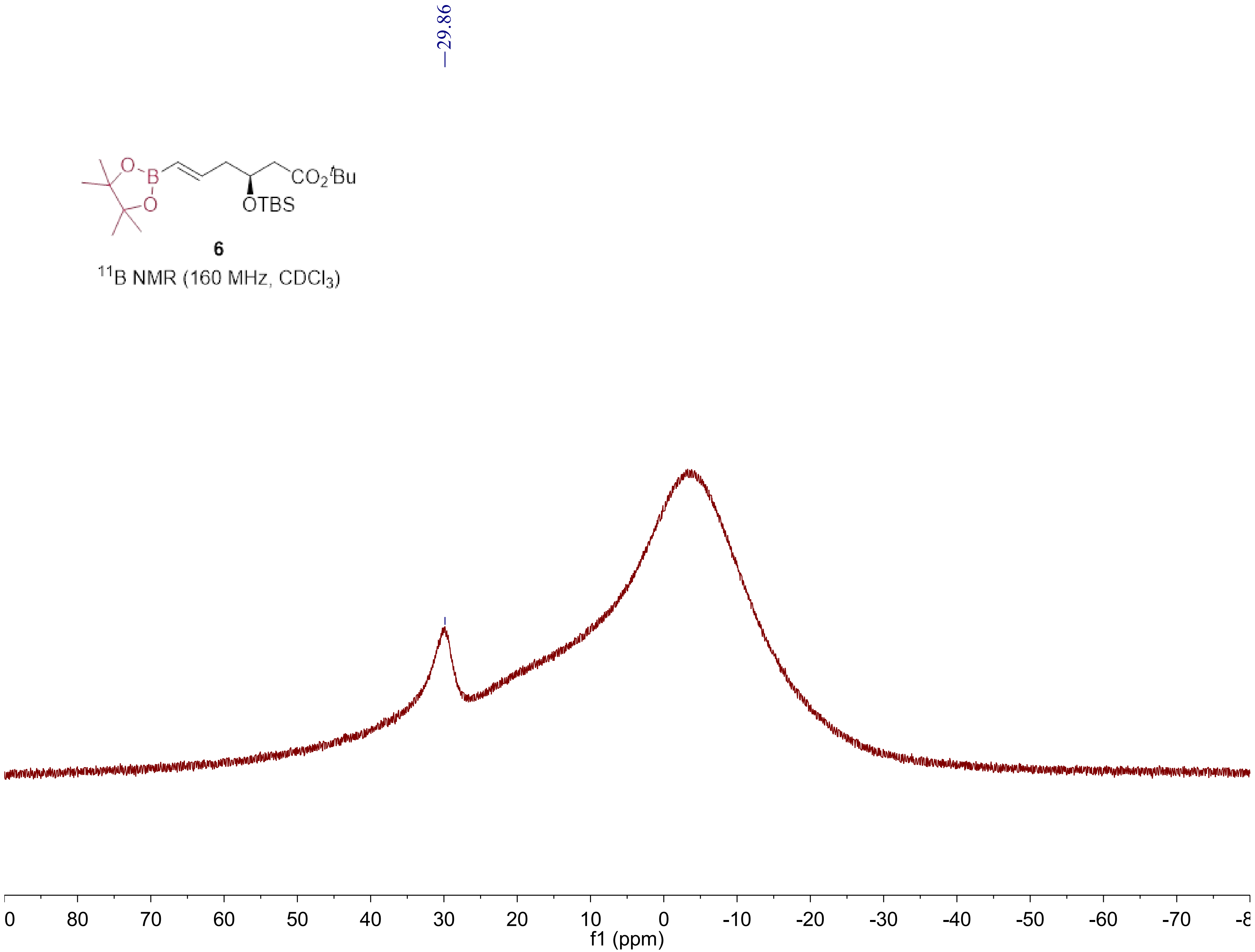

Supplementary Figure 126: ^{11}B NMR (160 MHz, $CDCl_3$) spectrum of **6**.

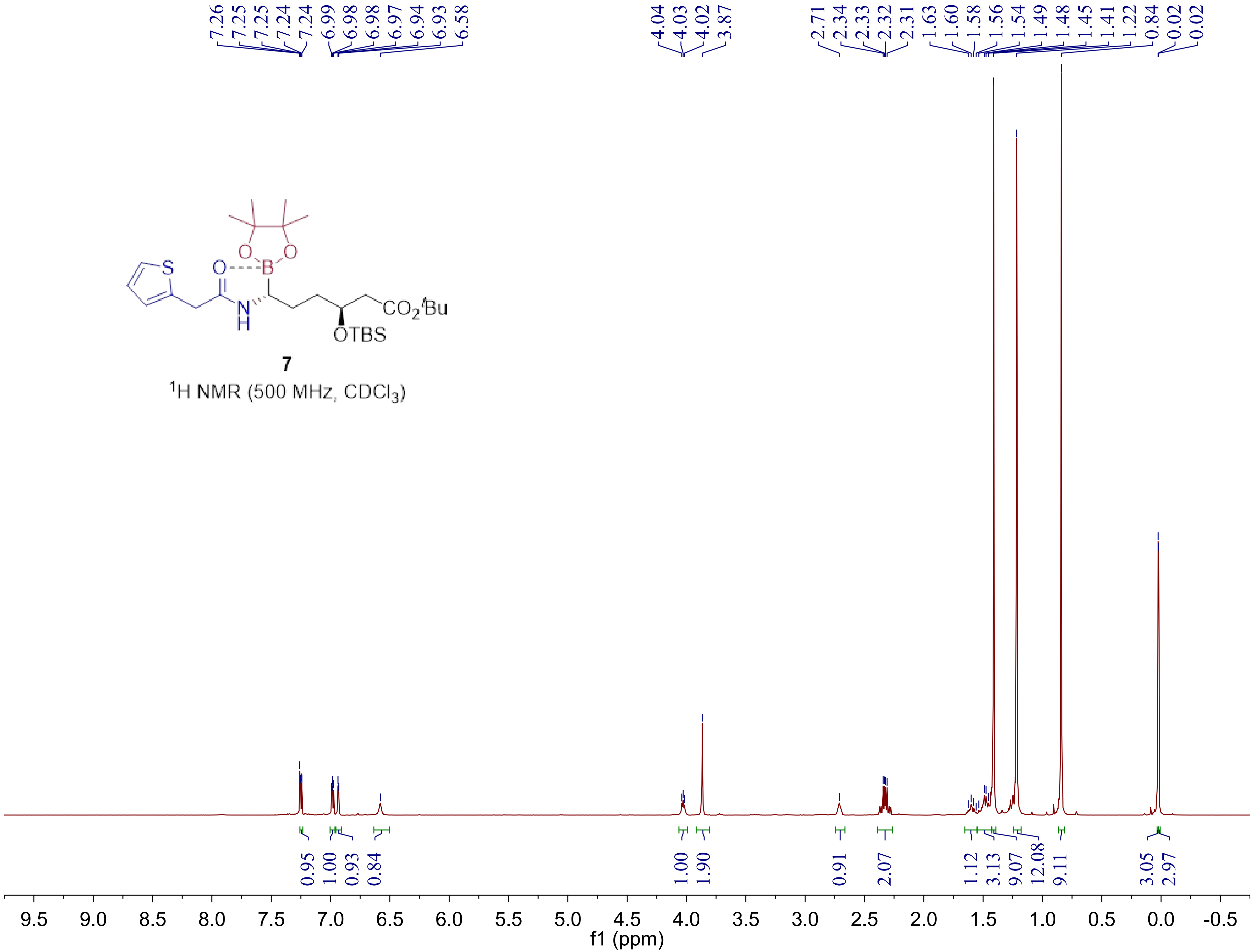

Supplementary Figure 127: ^{1}H NMR (500 MHz, $CDCl_3$) spectrum of **7**.

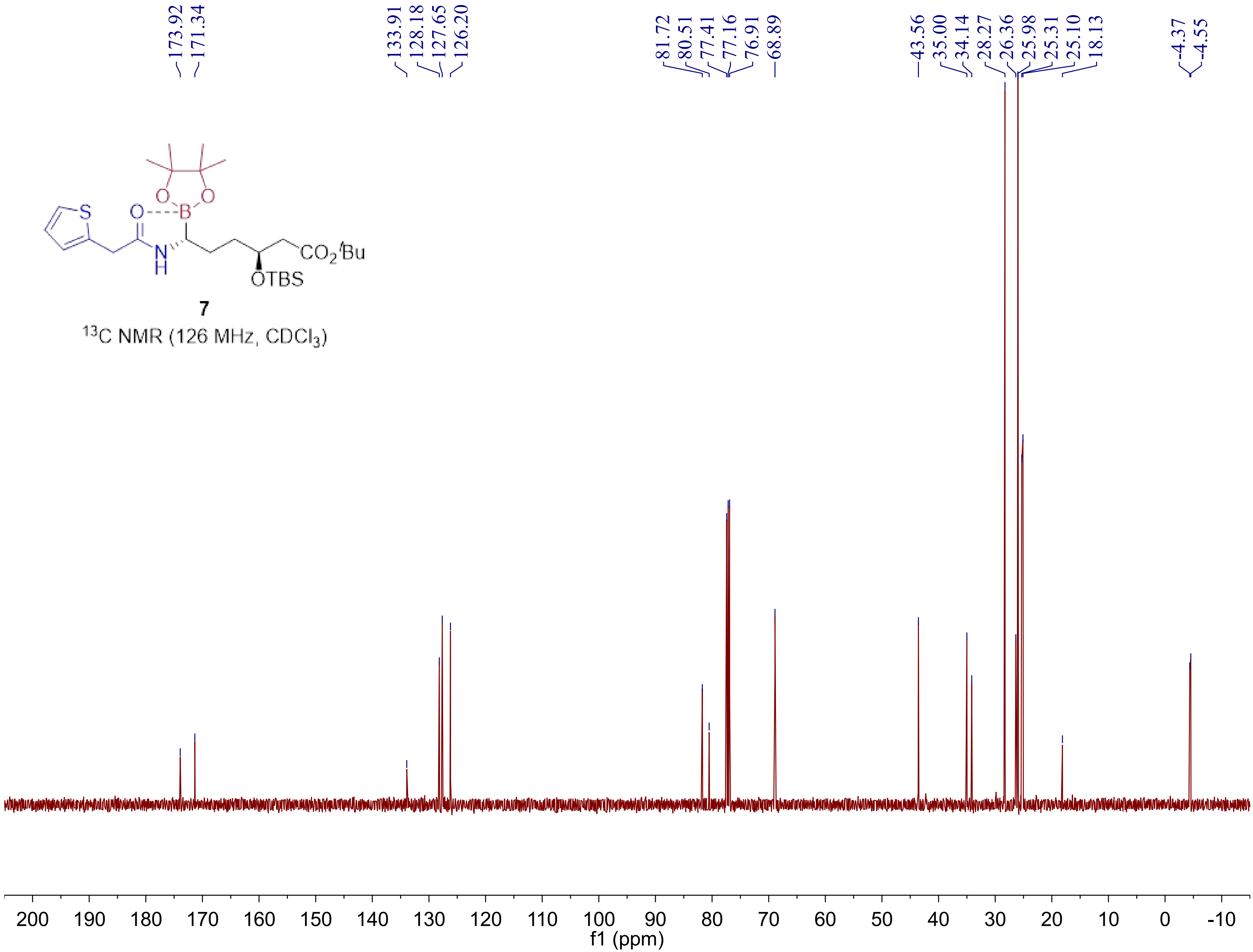

Supplementary Figure 128: ^{13}C NMR (126 MHz, $CDCl_3$) spectrum of **7**.

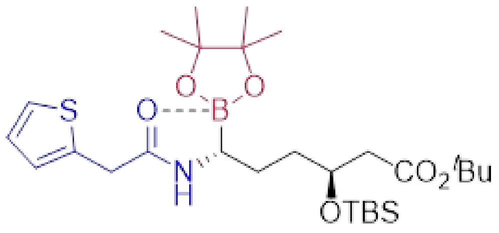

7

^{11}B NMR (160 MHz, $CDCl_3$)

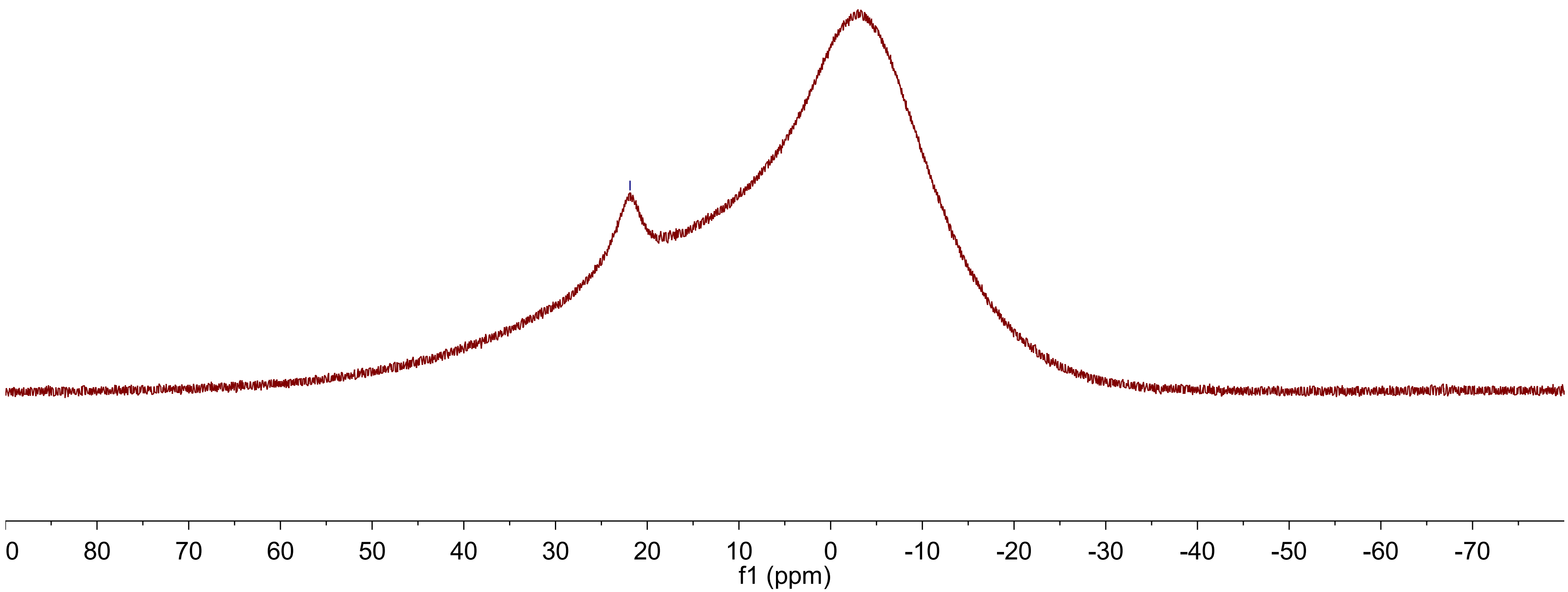

Supplementary Figure 129: ^{11}B NMR (160 MHz, $CDCl_3$) spectrum of **7**.

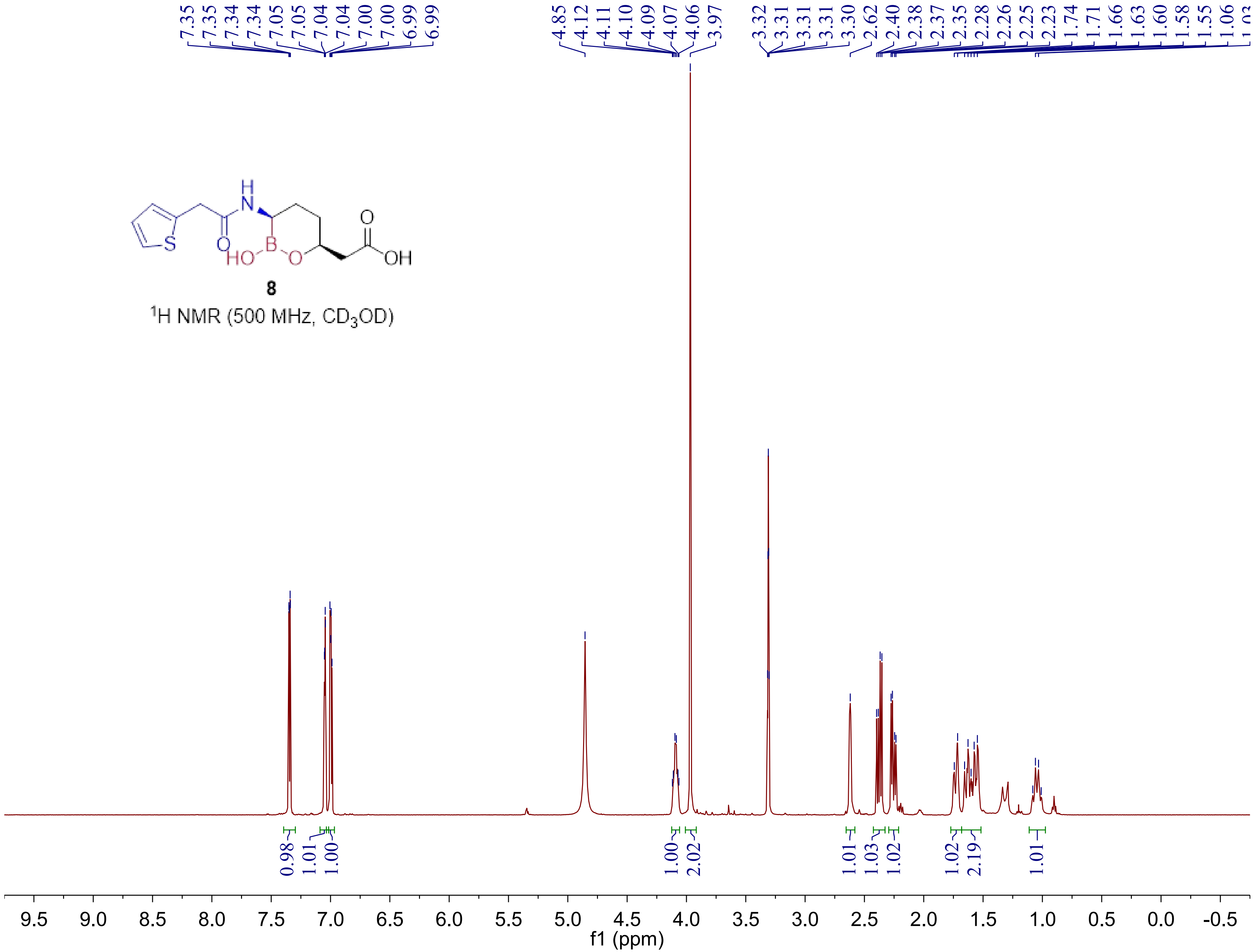

Supplementary Figure 130: ^{1}H NMR (500 MHz, CD_3OD) spectrum of **8**.

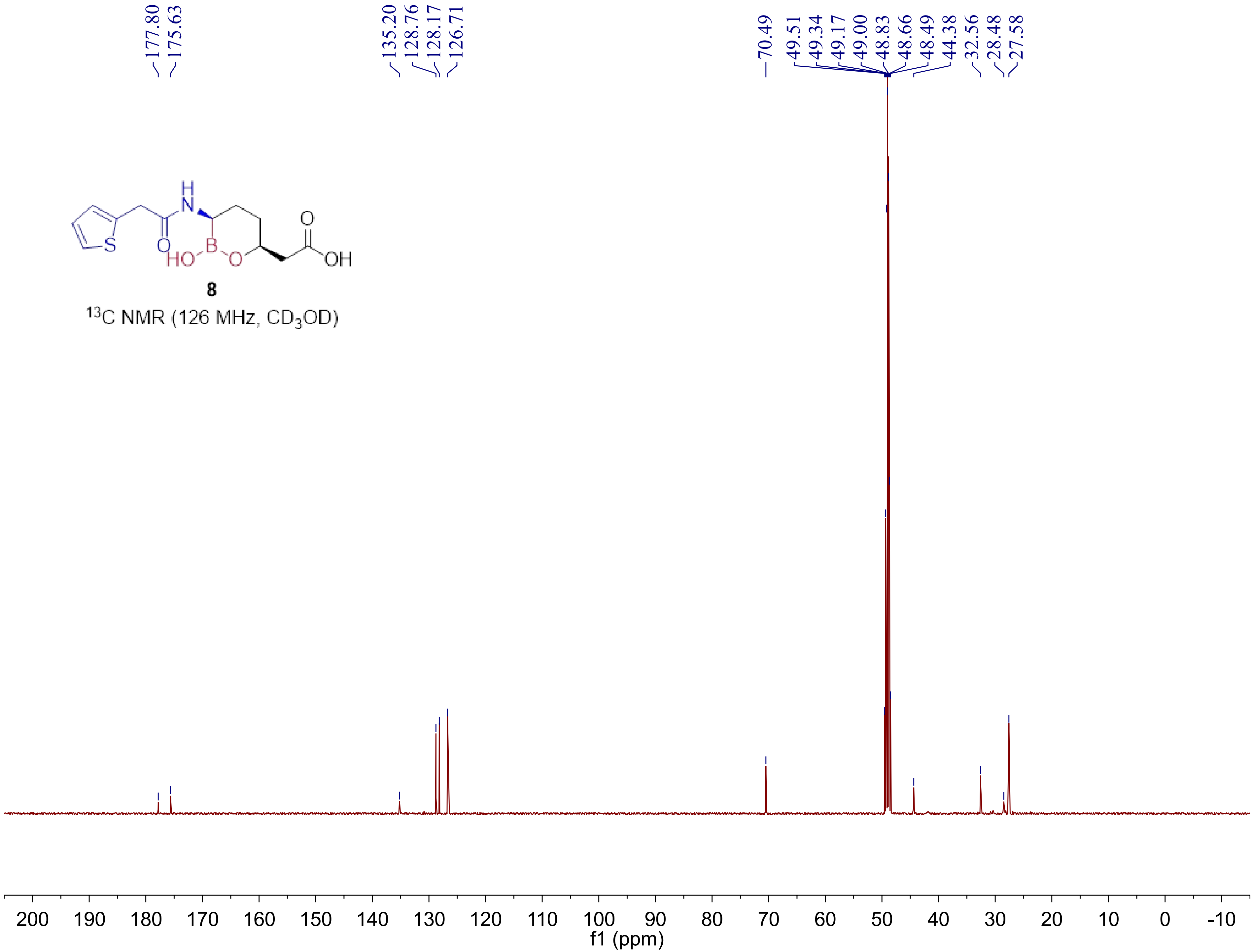

Supplementary Figure 131: ^{13}C NMR (126 MHz, CD_3OD) spectrum of **8**.

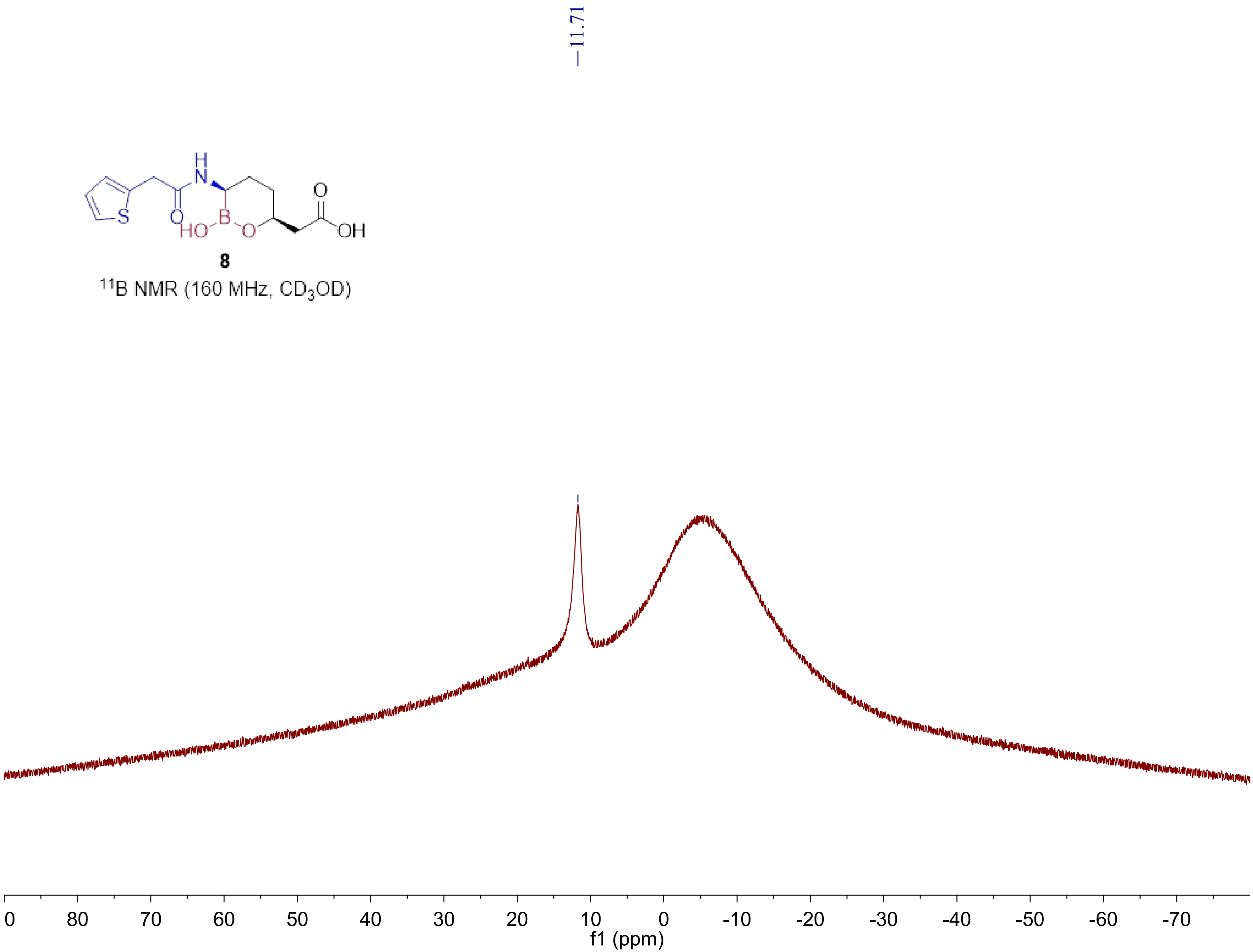

Supplementary Figure 132: ^{11}B NMR (160 MHz, CD_3OD) spectrum of **8**.

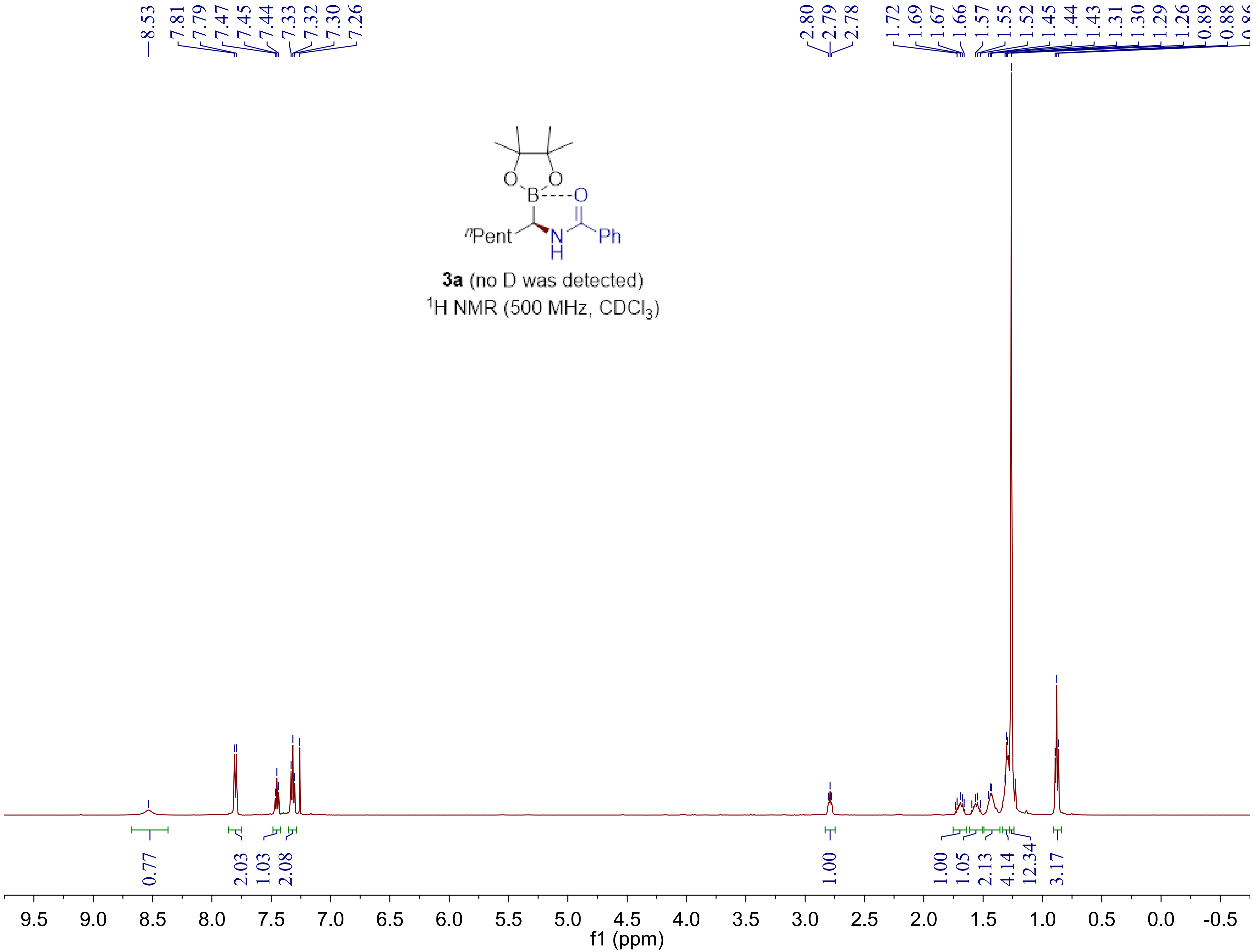

Supplementary Figure 133: ^{1}H NMR (500 MHz, $CDCl_3$) spectrum of **3a** (no D was detected).

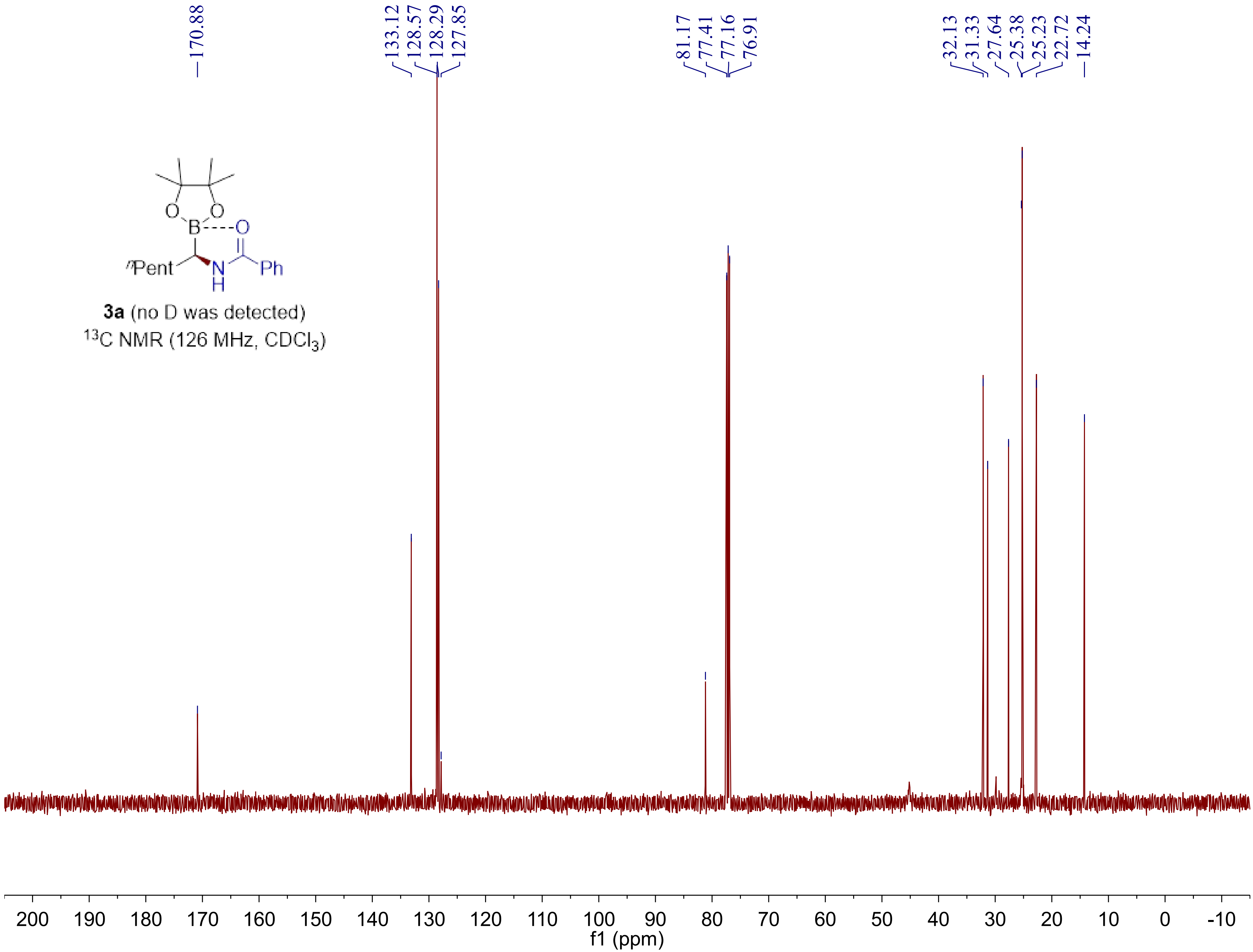

Supplementary Figure 134: ^{13}C NMR (126 MHz, $CDCl_3$) spectrum of **3a** (no D was detected).

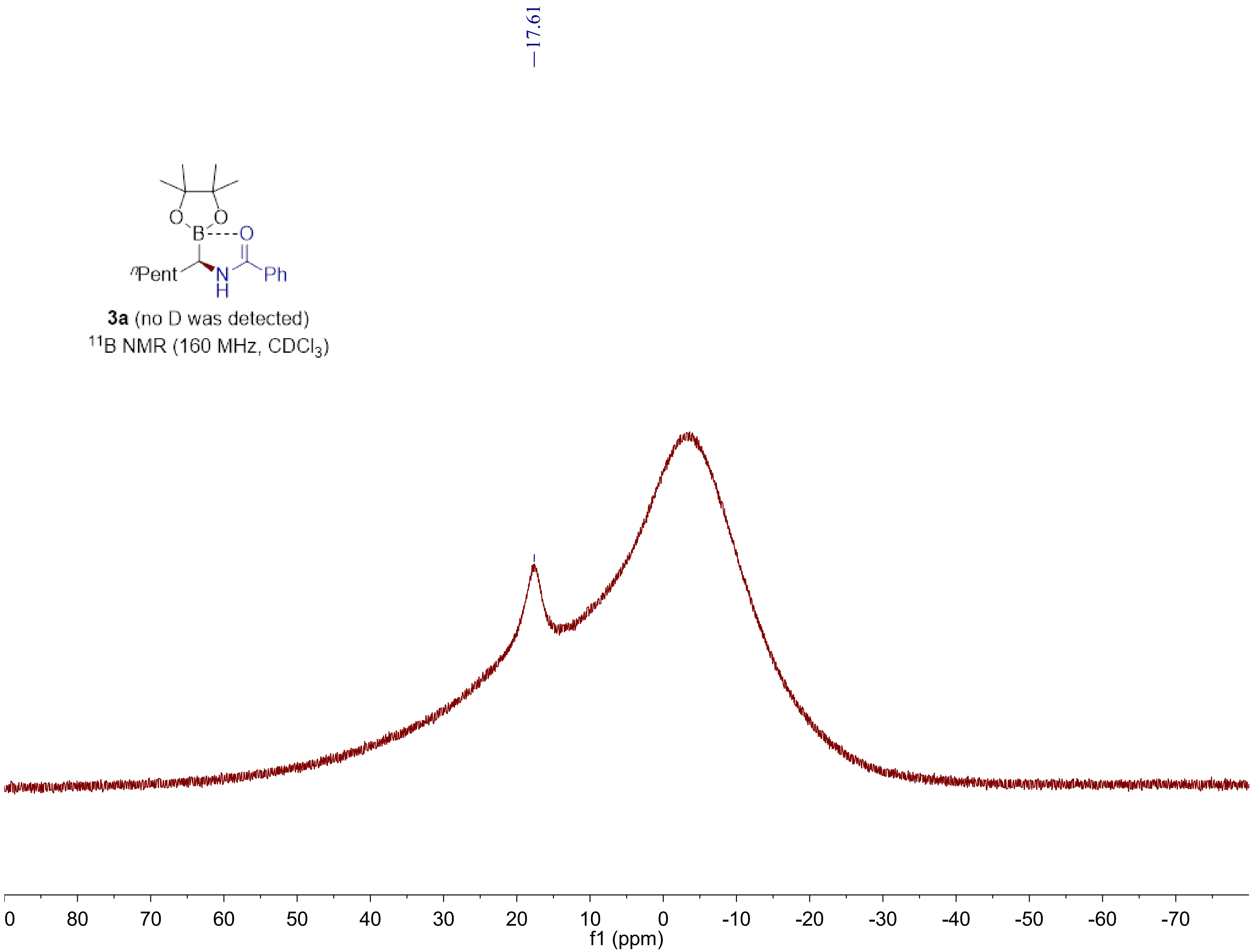

Supplementary Figure 135: ^{11}B NMR (160 MHz, $CDCl_3$) spectrum of **3a** (no D was detected).

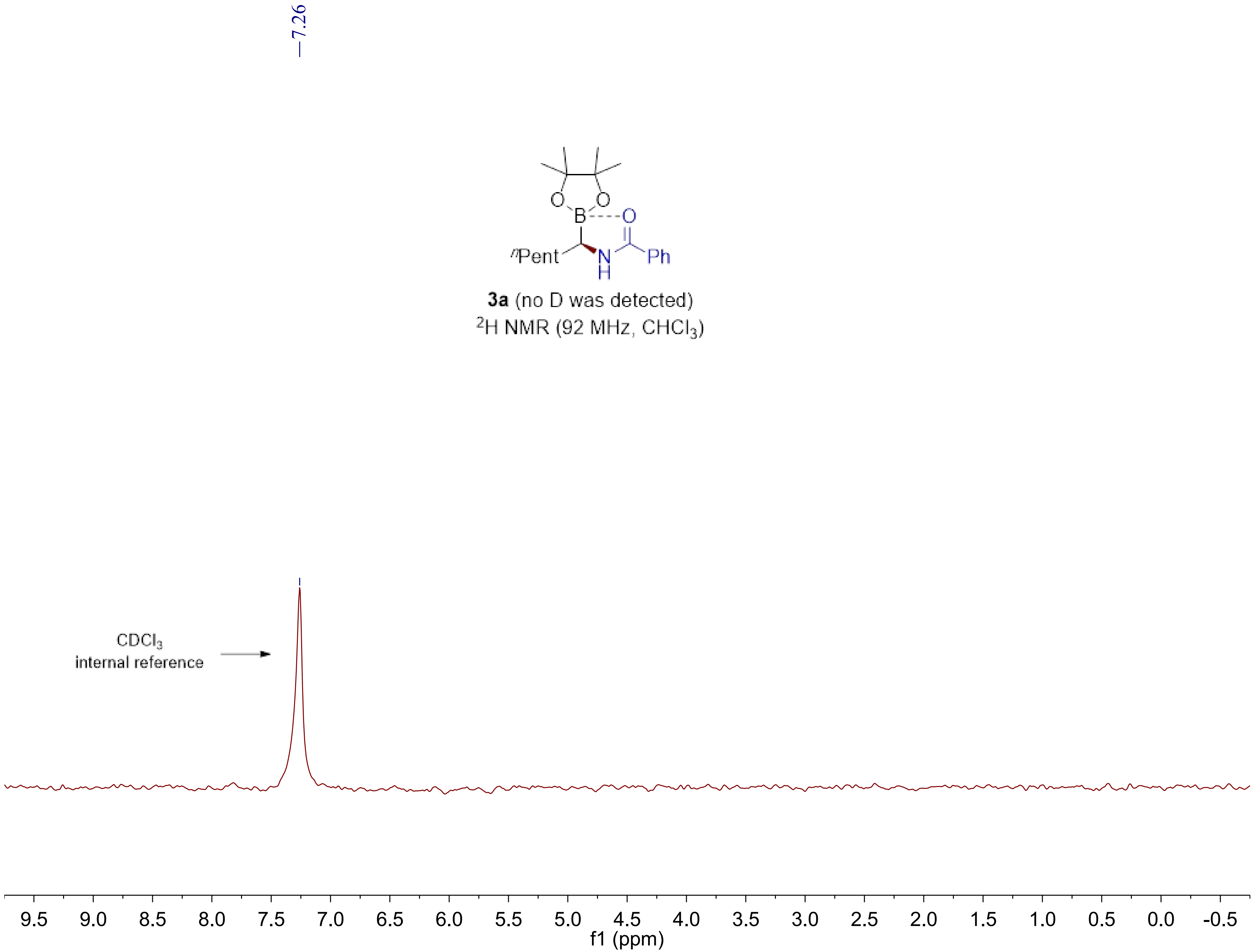

Supplementary Figure 136: ^{2}H NMR (92 MHz, $CHCl_3$) spectrum of **3a** (no D was detected).

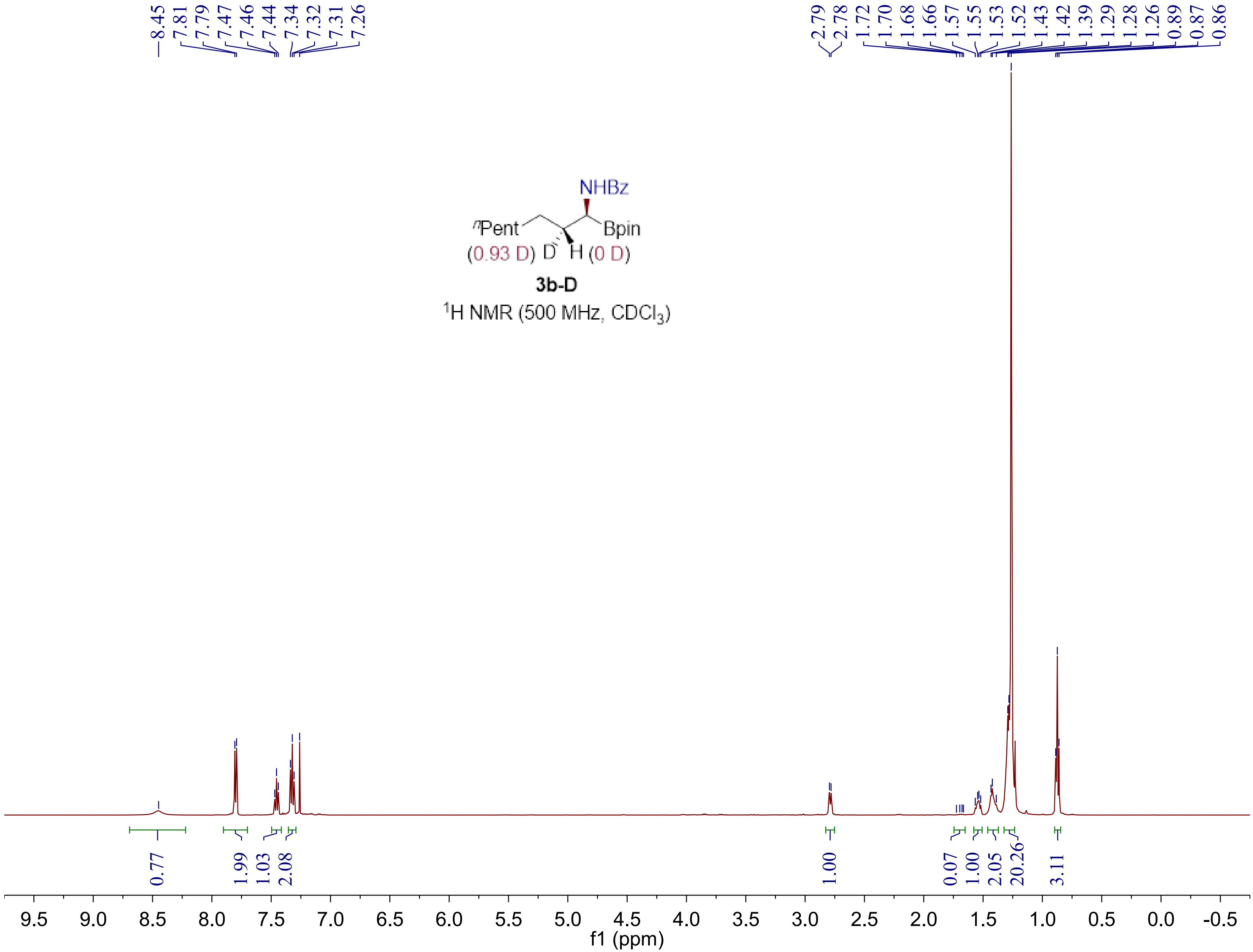

Supplementary Figure 137: ^{1}H NMR (500 MHz, $CDCl_3$) spectrum of **3b-D**.

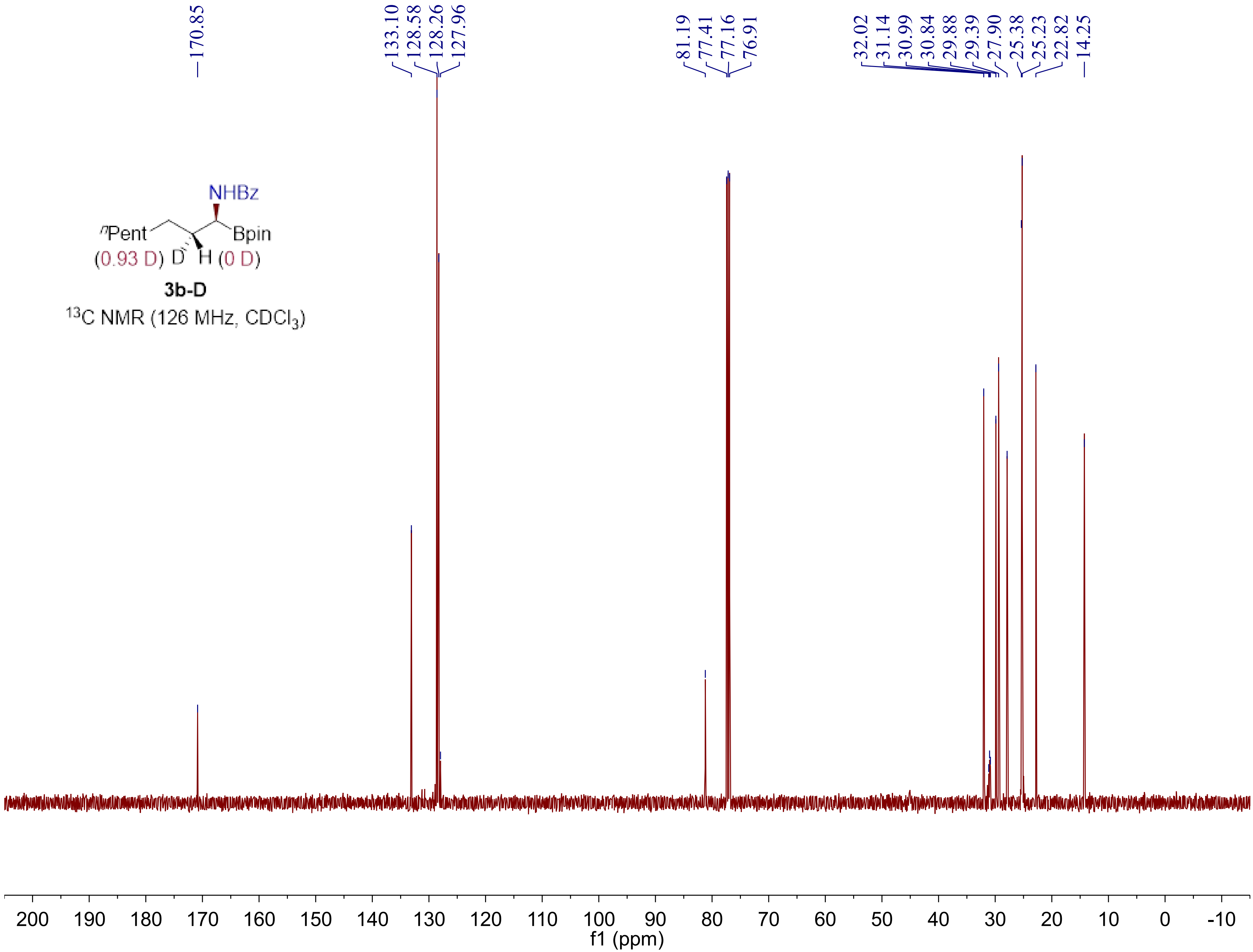

Supplementary Figure 138: ^{13}C NMR (126 MHz, $CDCl_3$) spectrum of **3b-D**.

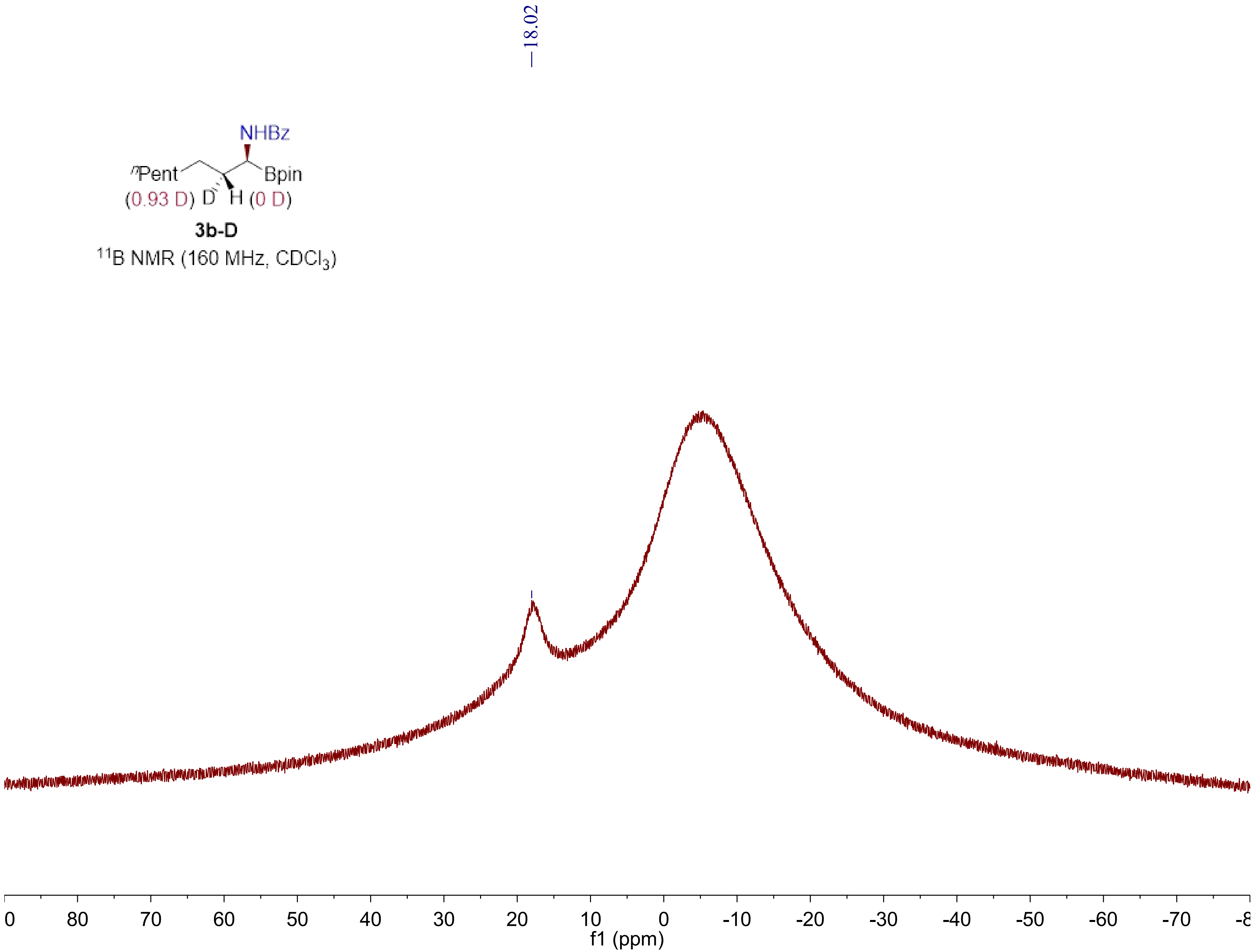

Supplementary Figure 139: ^{11}B NMR (160 MHz, $CDCl_3$) spectrum of **3b-D**.

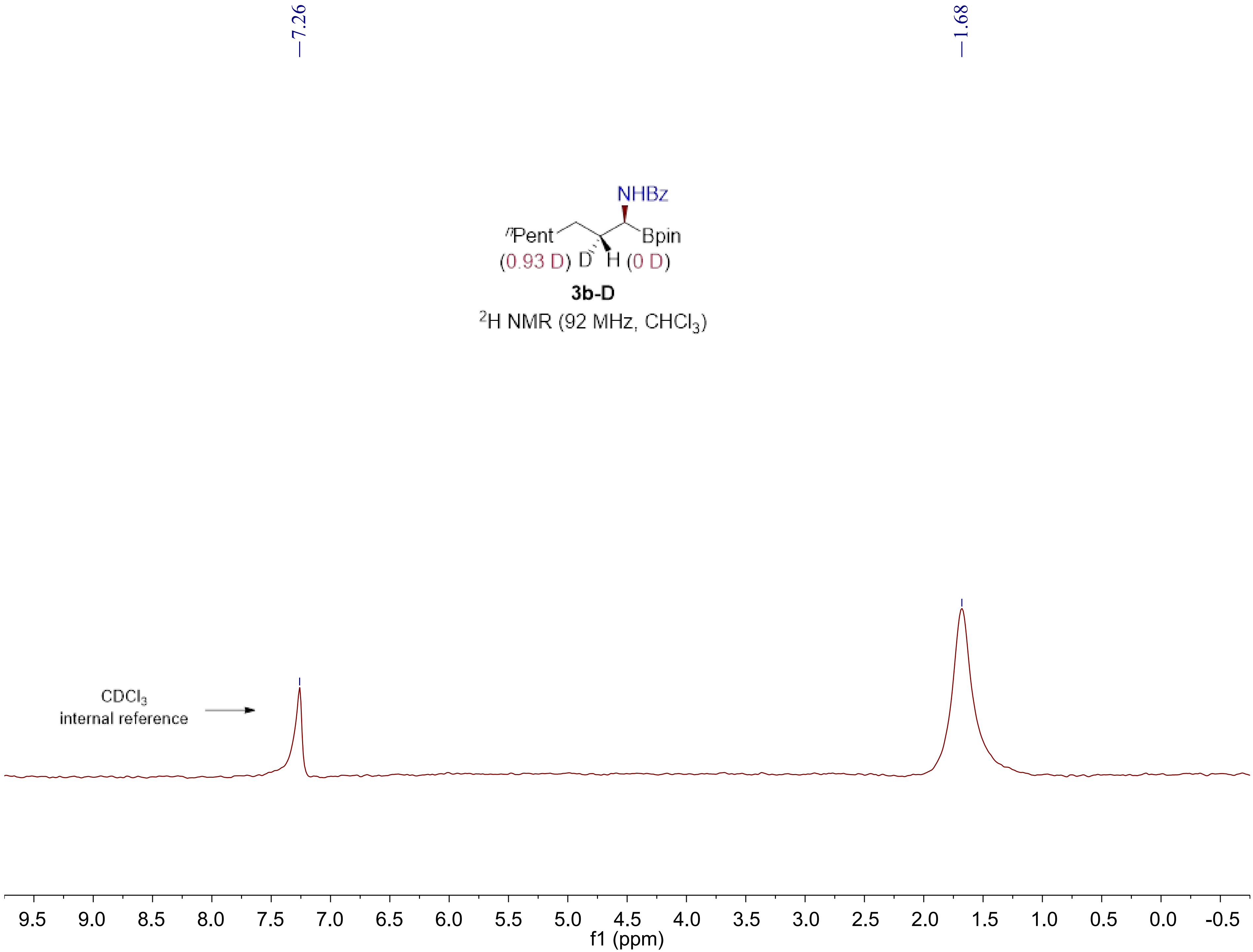

Supplementary Figure 140: ^{2}H NMR (92 MHz, $CHCl_3$) spectrum of **3b-D**.

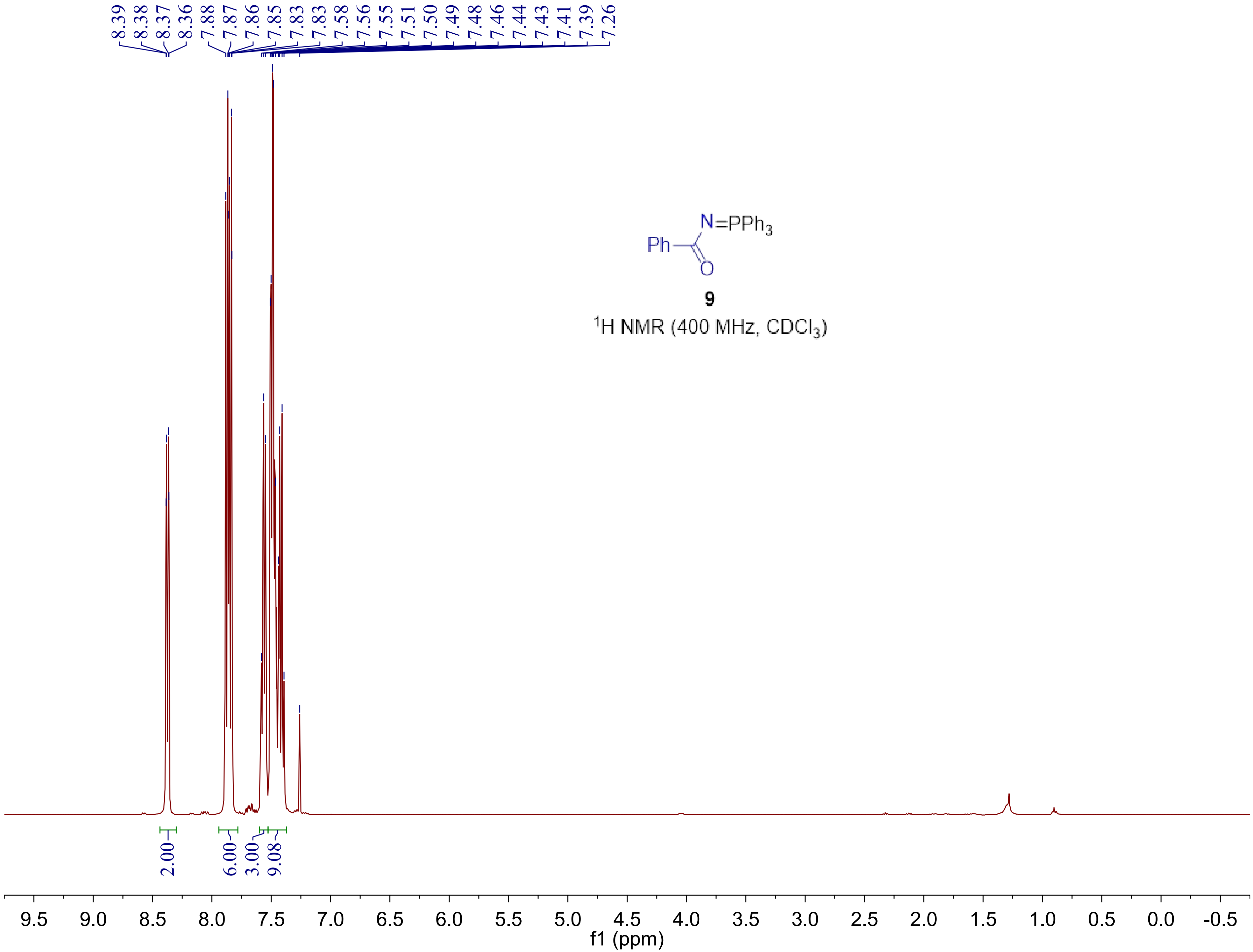

Supplementary Figure 141: ^{1}H NMR (400 MHz, $CDCl_3$) spectrum of **9**.

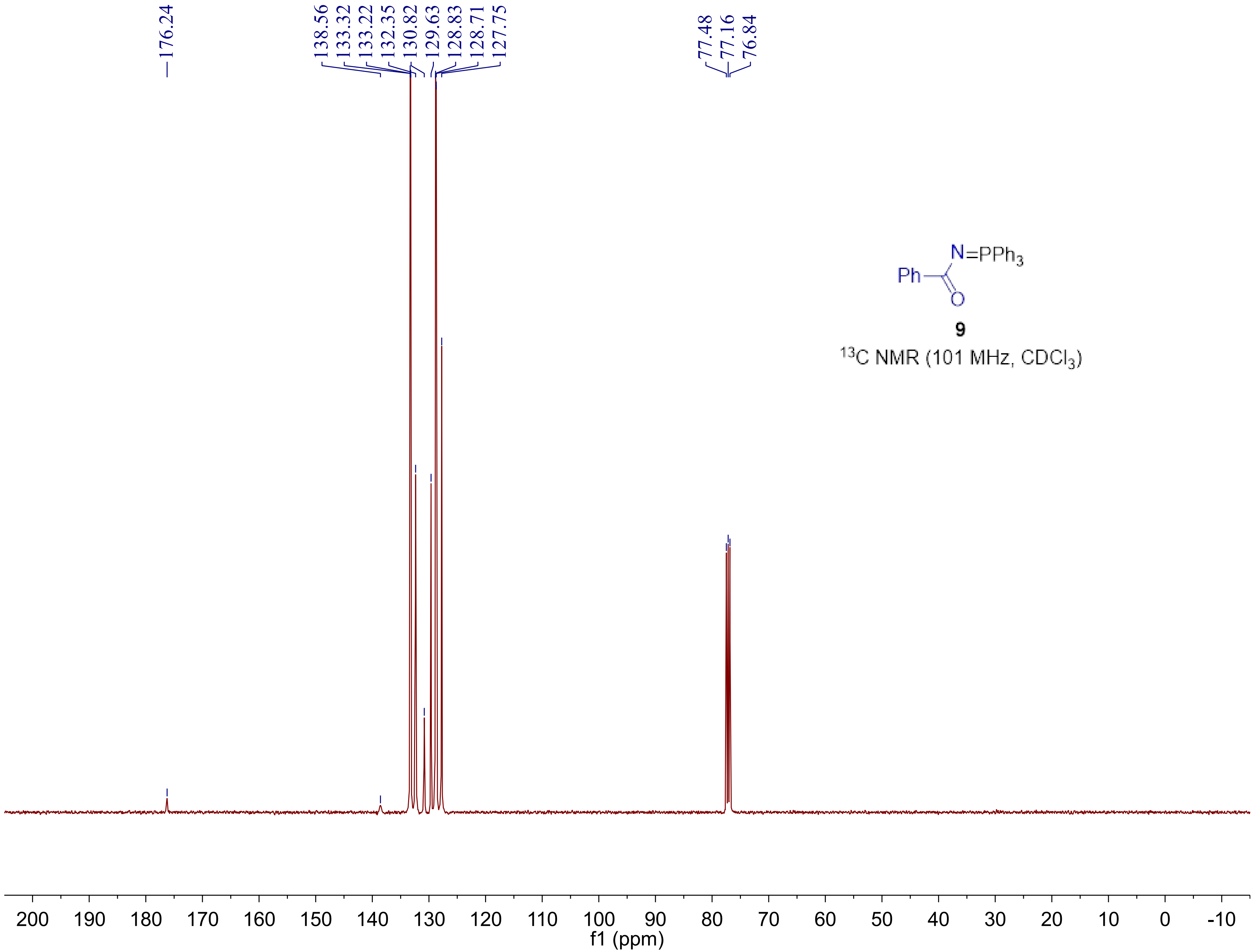

Supplementary Figure 142: ^{13}C NMR (101 MHz, $CDCl_3$) spectrum of **9**.

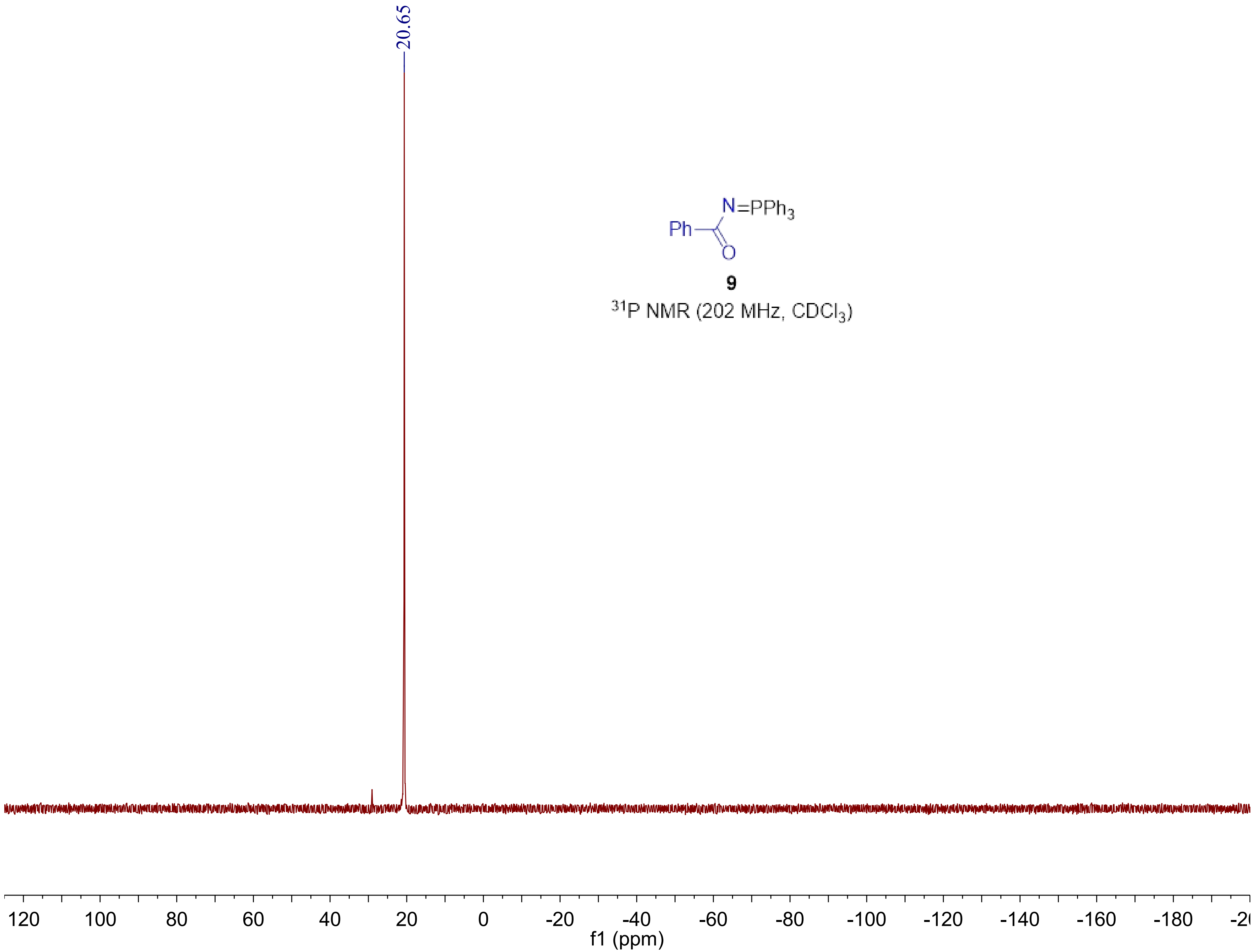

Supplementary Figure 143: ^{31}P NMR (202 MHz, $CDCl_3$) spectrum of **9**.

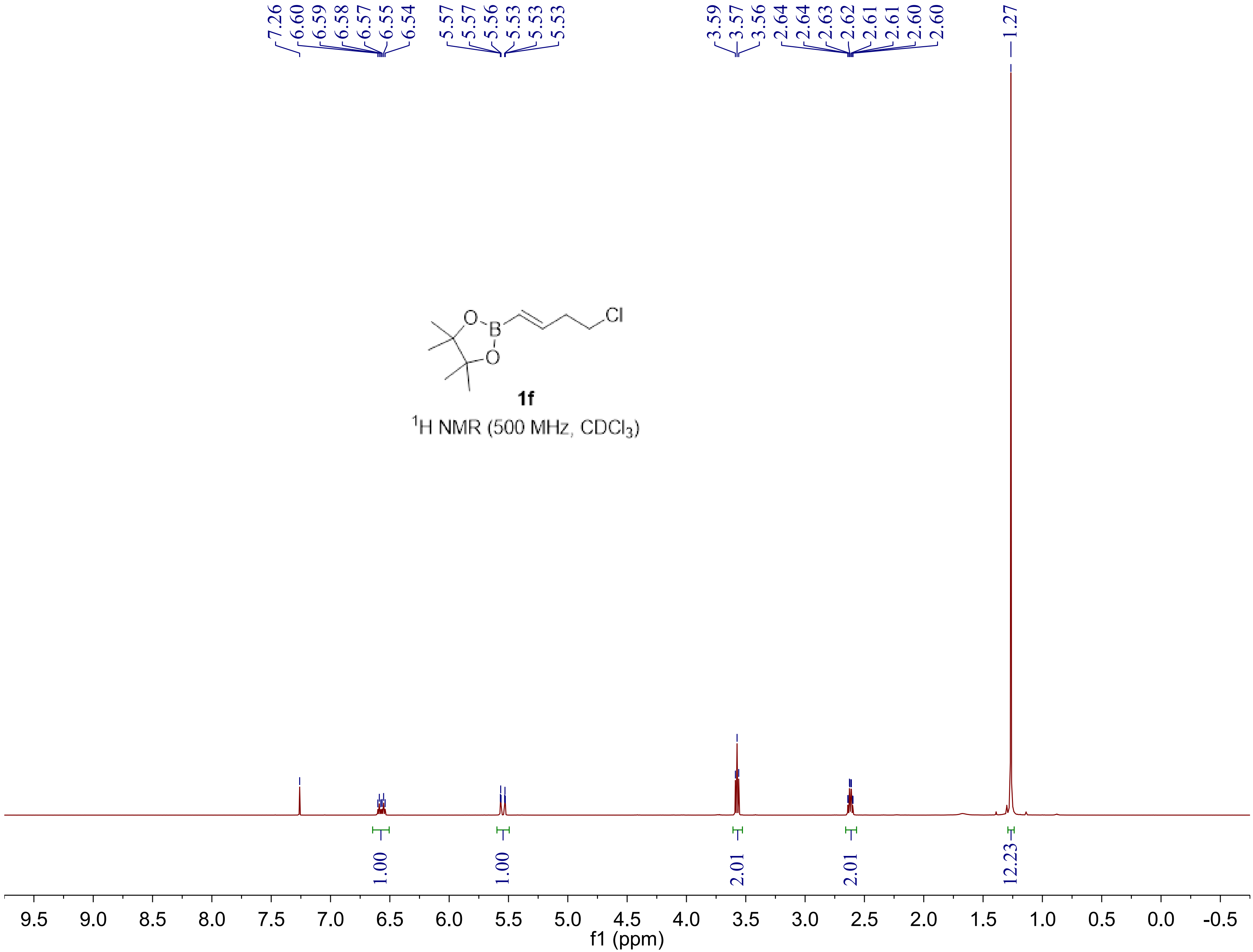

Supplementary Figure 144: ^{1}H NMR (500 MHz, $CDCl_3$) spectrum of **1f**.

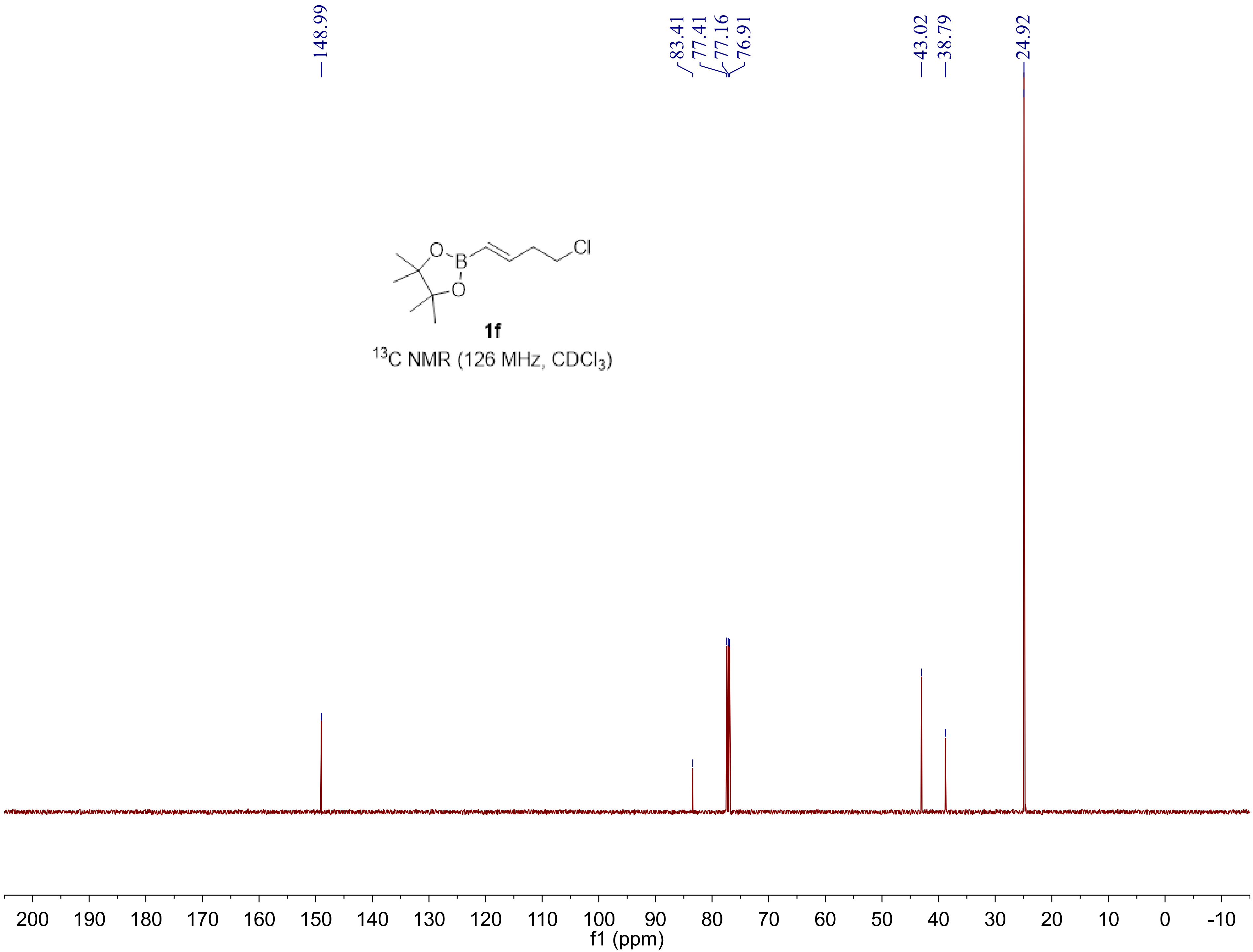

Supplementary Figure 145: ^{13}C NMR (126 MHz, $CDCl_3$) spectrum of **1f**.

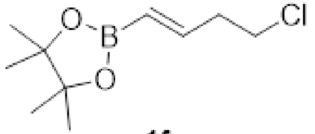

1f

^{11}B NMR (160 MHz, $CDCl_3$)

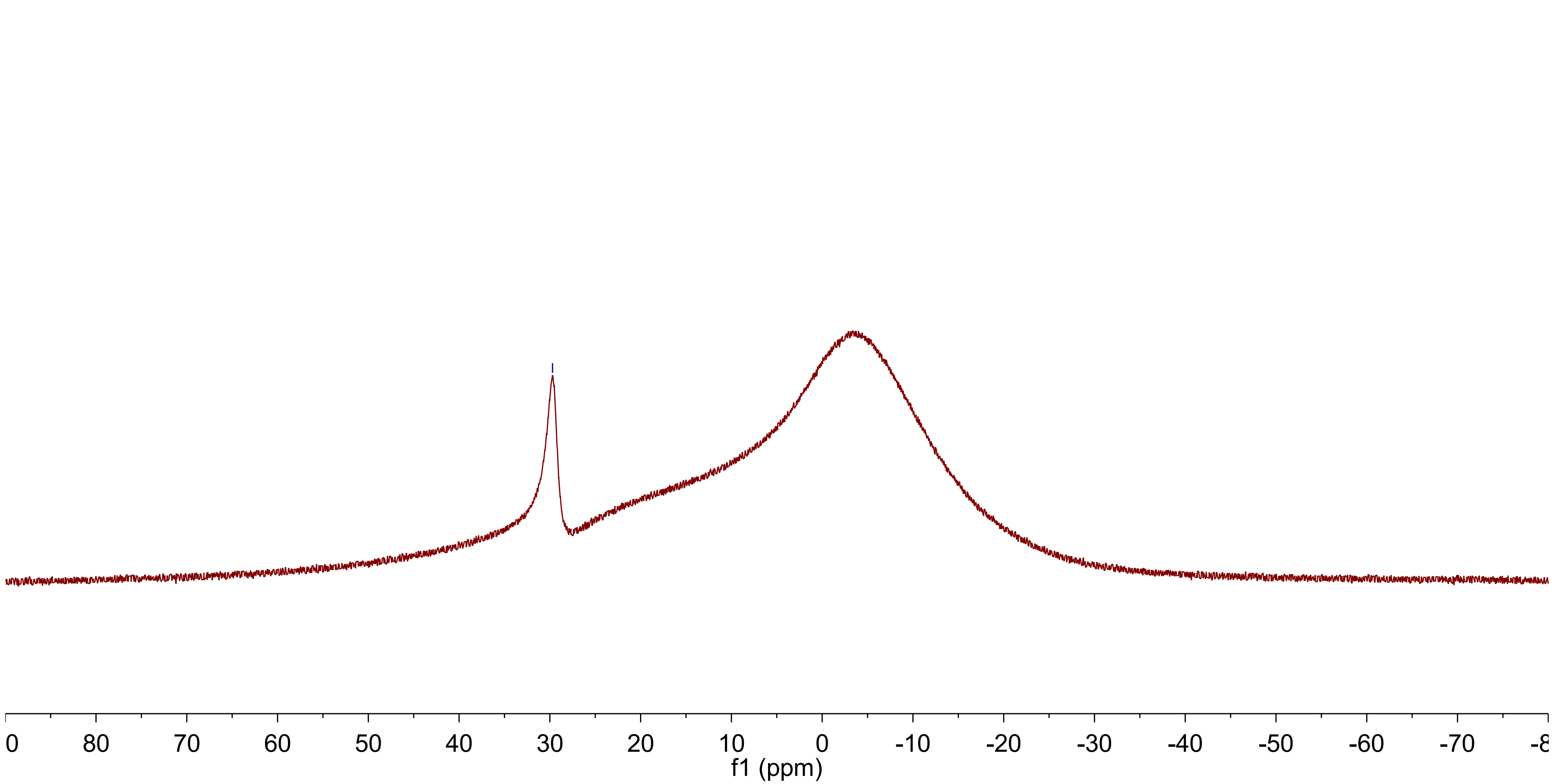

Supplementary Figure 146: ^{11}B NMR (160 MHz, $CDCl_3$) spectrum of **1f**.

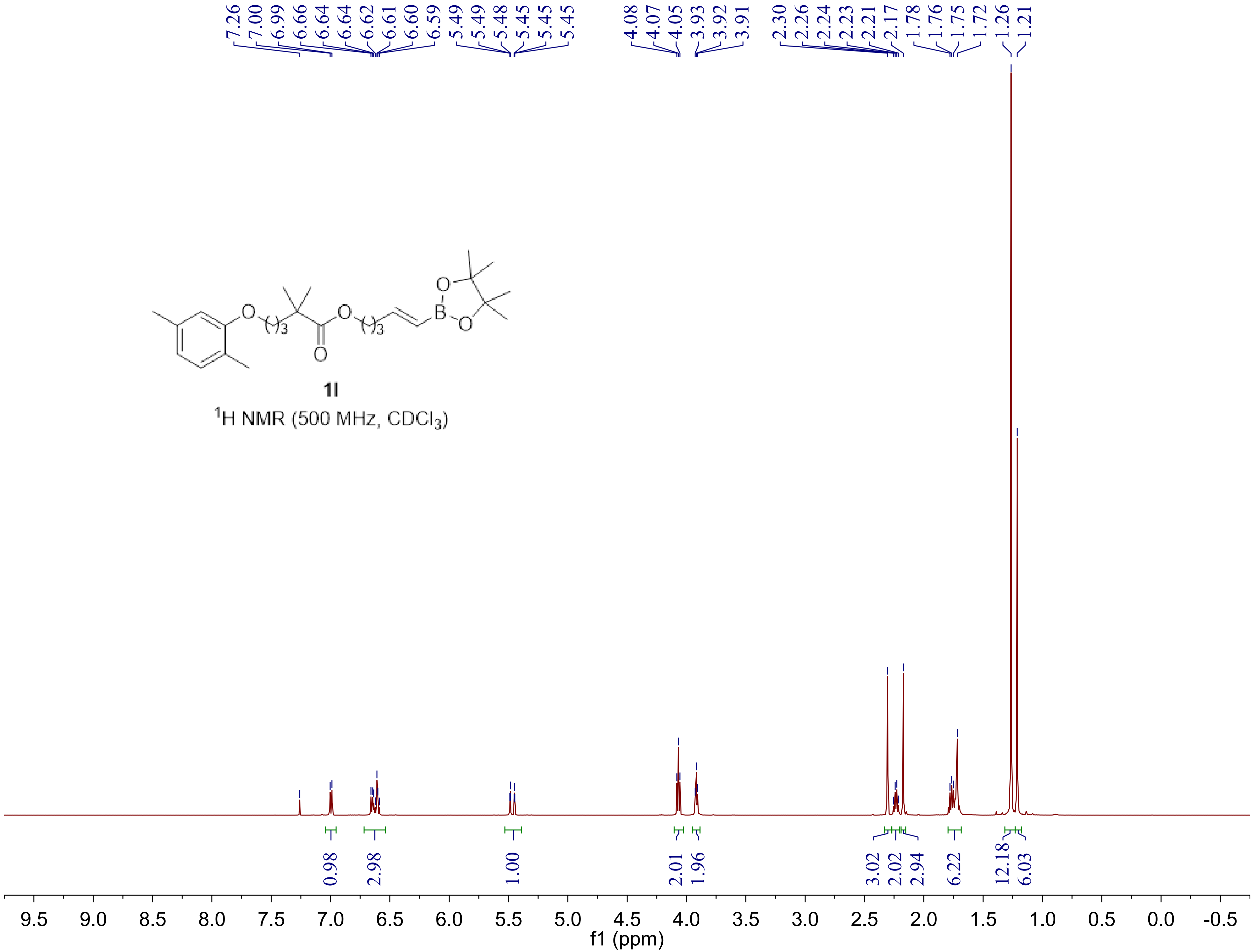

Supplementary Figure 147: ^{1}H NMR (500 MHz, CDCl$_3$) spectrum of **1l**.

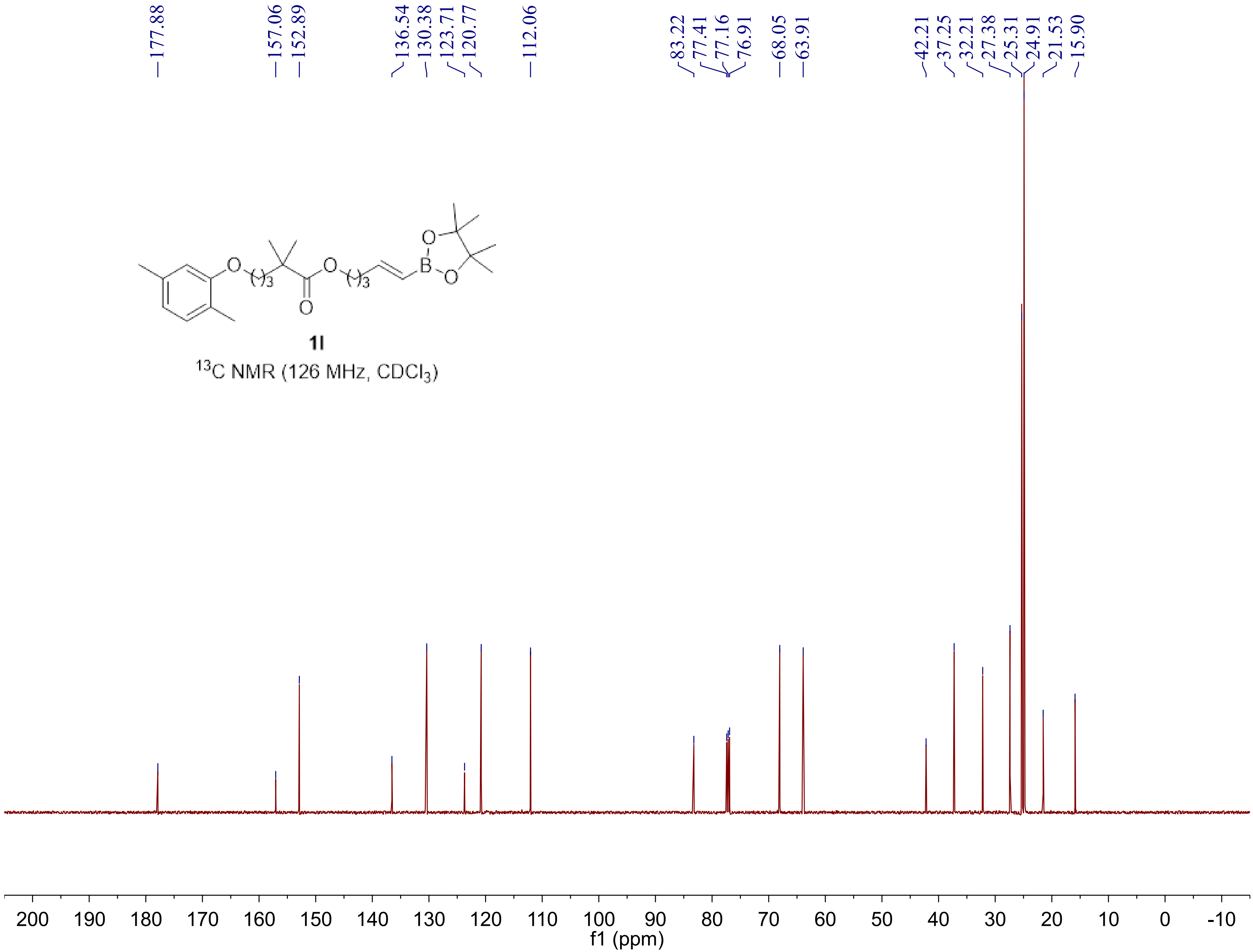

Supplementary Figure 148: ^{13}C NMR (126 MHz, $CDCl_3$) spectrum of **1l**.

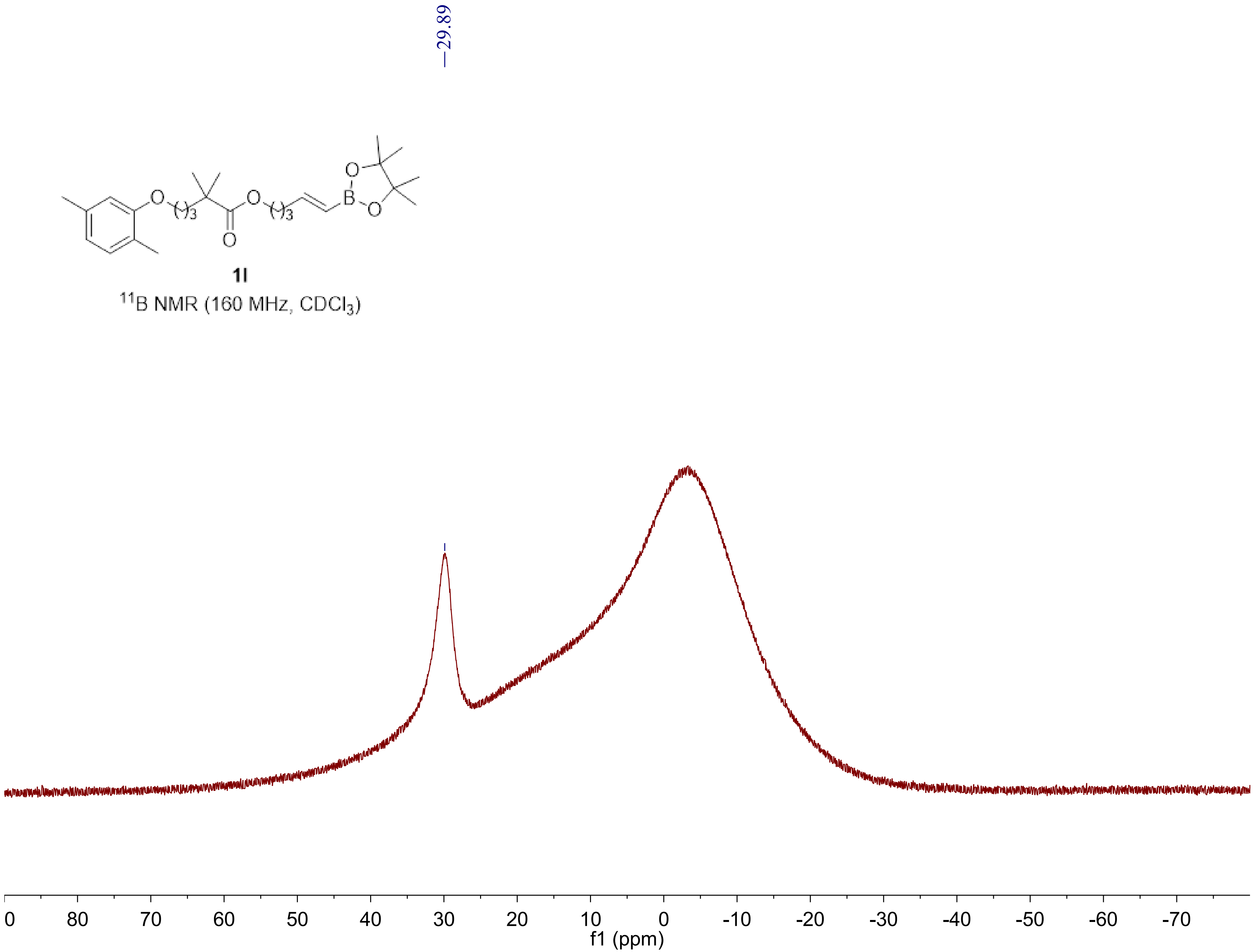

Supplementary Figure 149: ^{11}B NMR (160 MHz, CDCl$_3$) spectrum of **1l**.

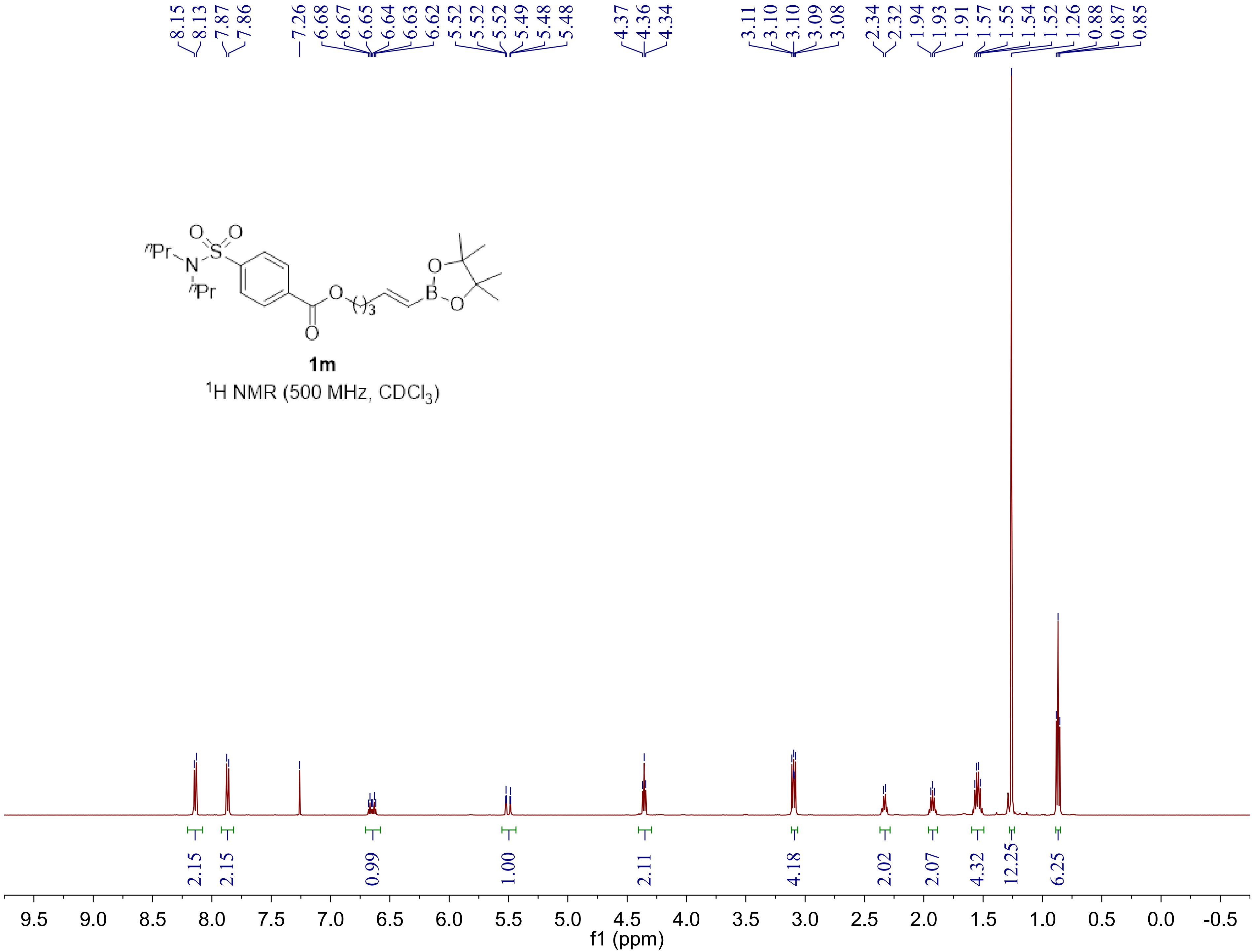

Supplementary Figure 150: ^{1}H NMR (500 MHz, $CDCl_3$) spectrum of **1m**.

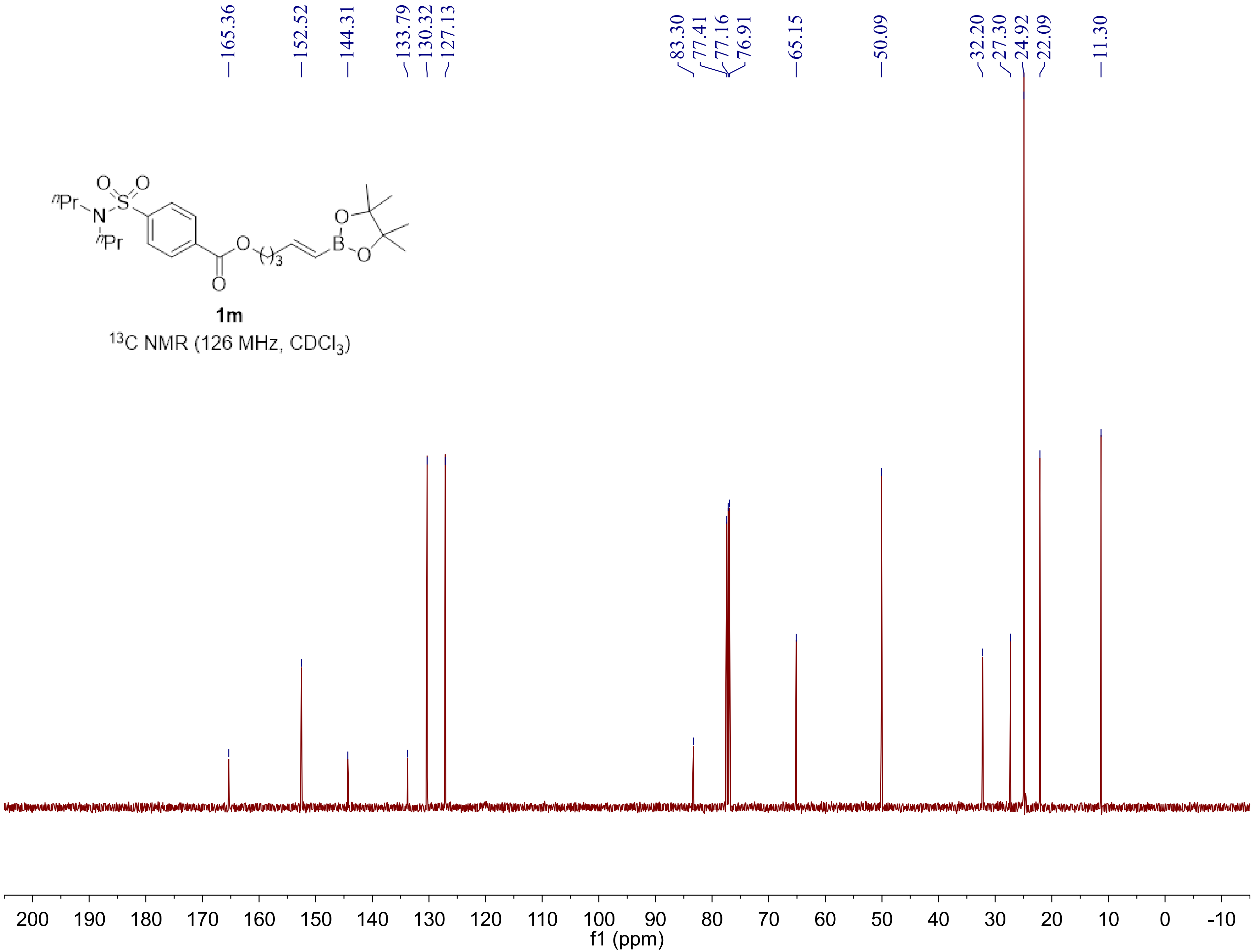

Supplementary Figure 151: ^{13}C NMR (126 MHz, $CDCl_3$) spectrum of **1m**.

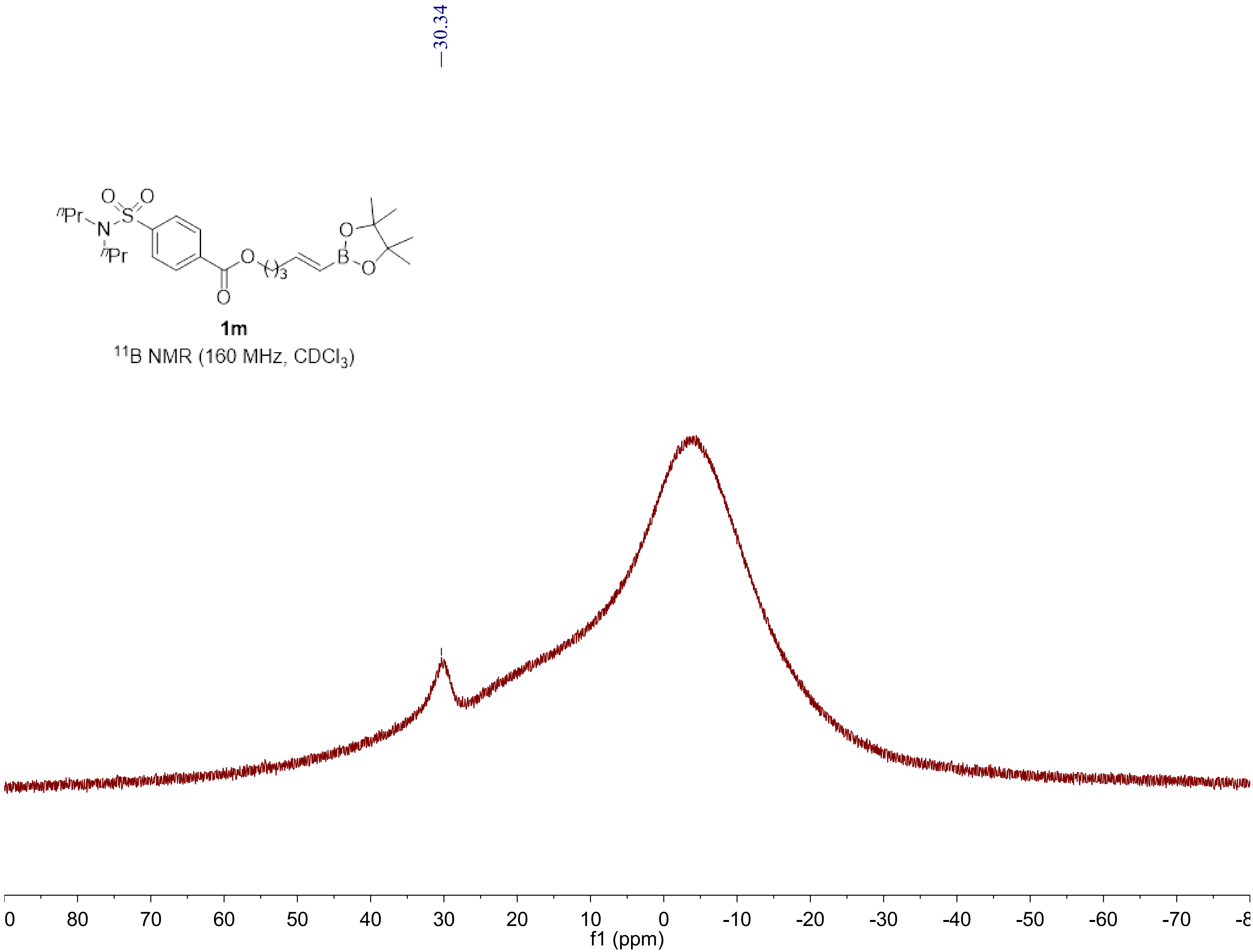

Supplementary Figure 152: ^{11}B NMR (160 MHz, $CDCl_3$) spectrum of **1m**.

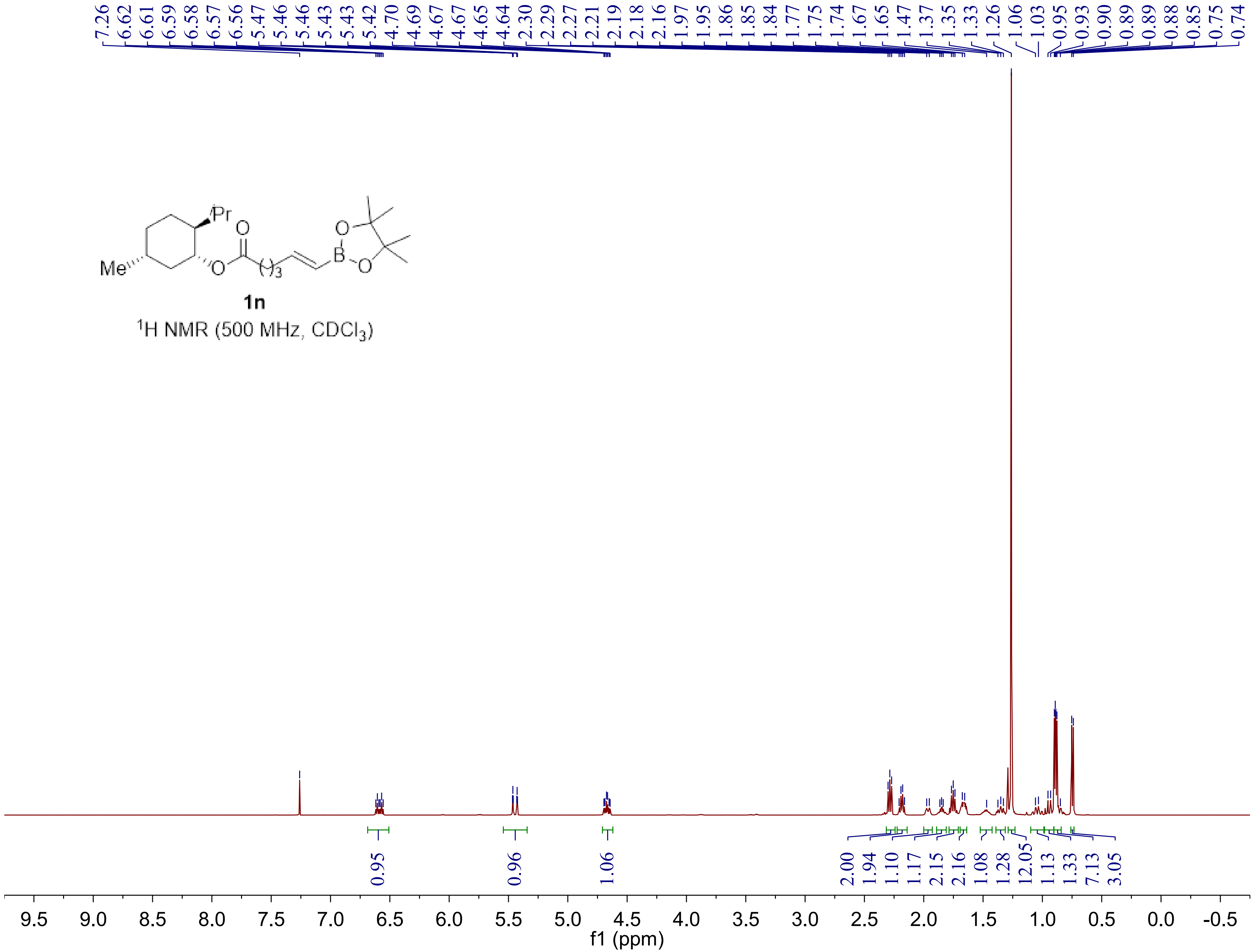

Supplementary Figure 153: ^{1}H NMR (500 MHz, $CDCl_3$) spectrum of **1n**.

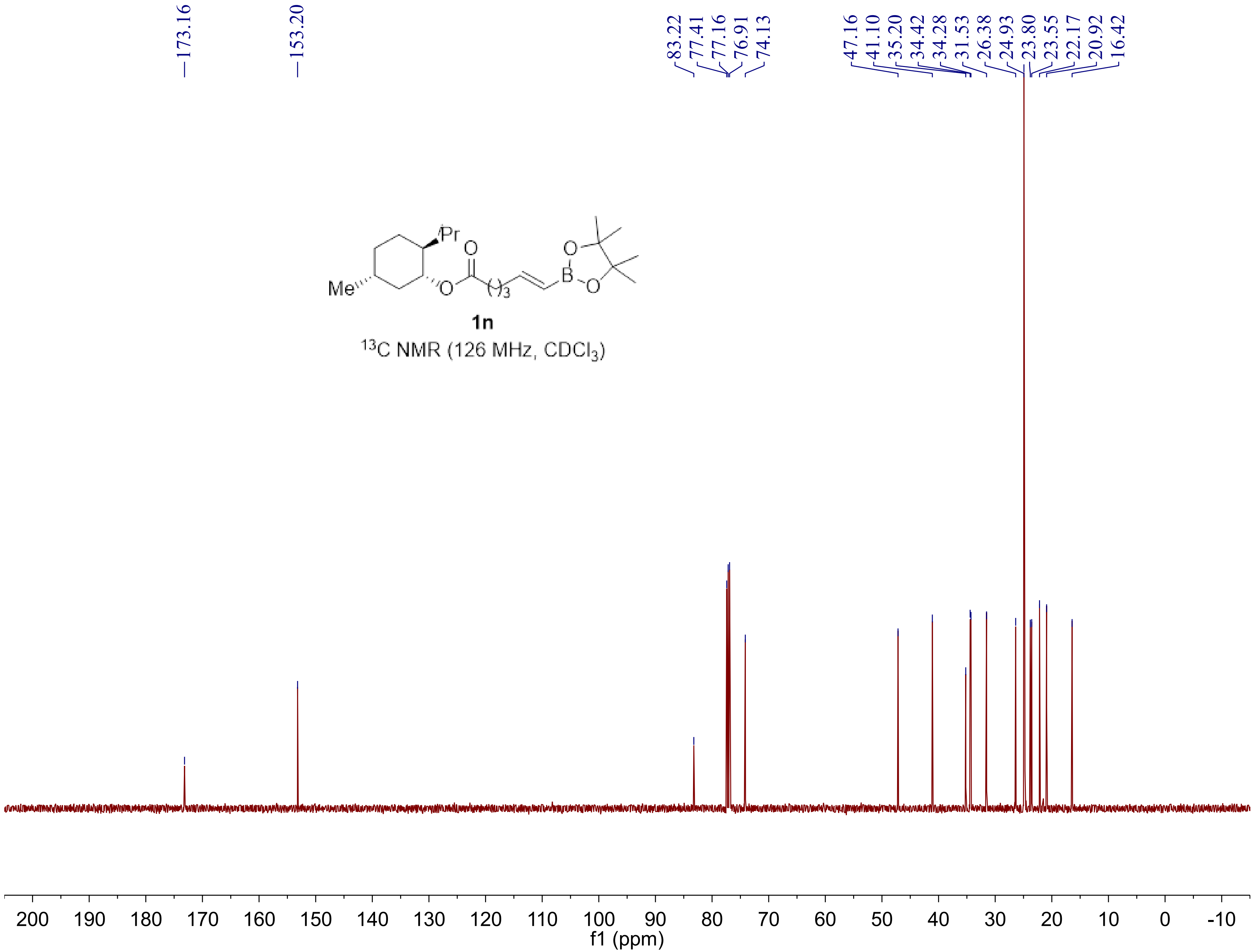

Supplementary Figure 154: ^{13}C NMR (126 MHz, $CDCl_3$) spectrum of **1n**.

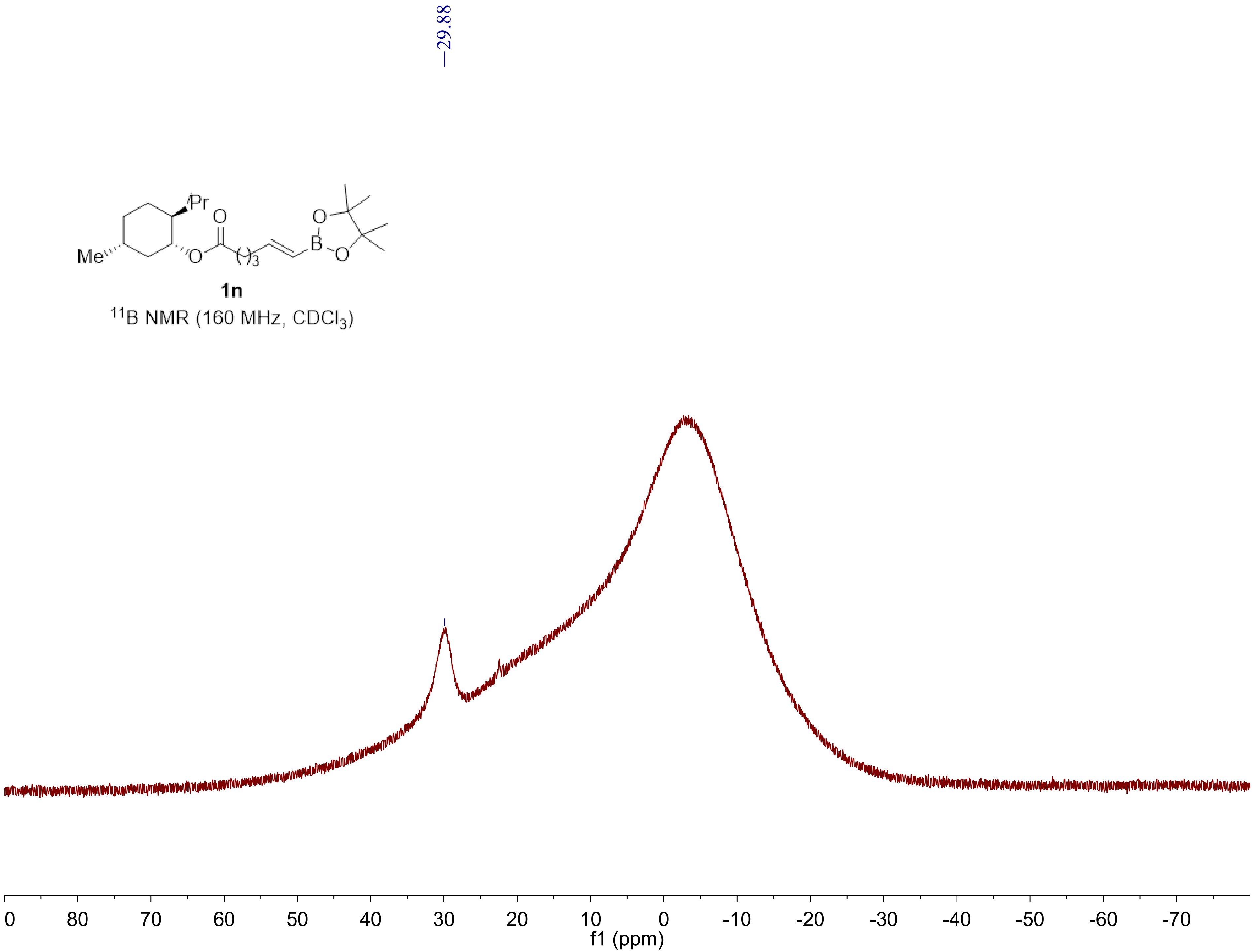

Supplementary Figure 155: ^{11}B NMR (160 MHz, $CDCl_3$) spectrum of **1n**.

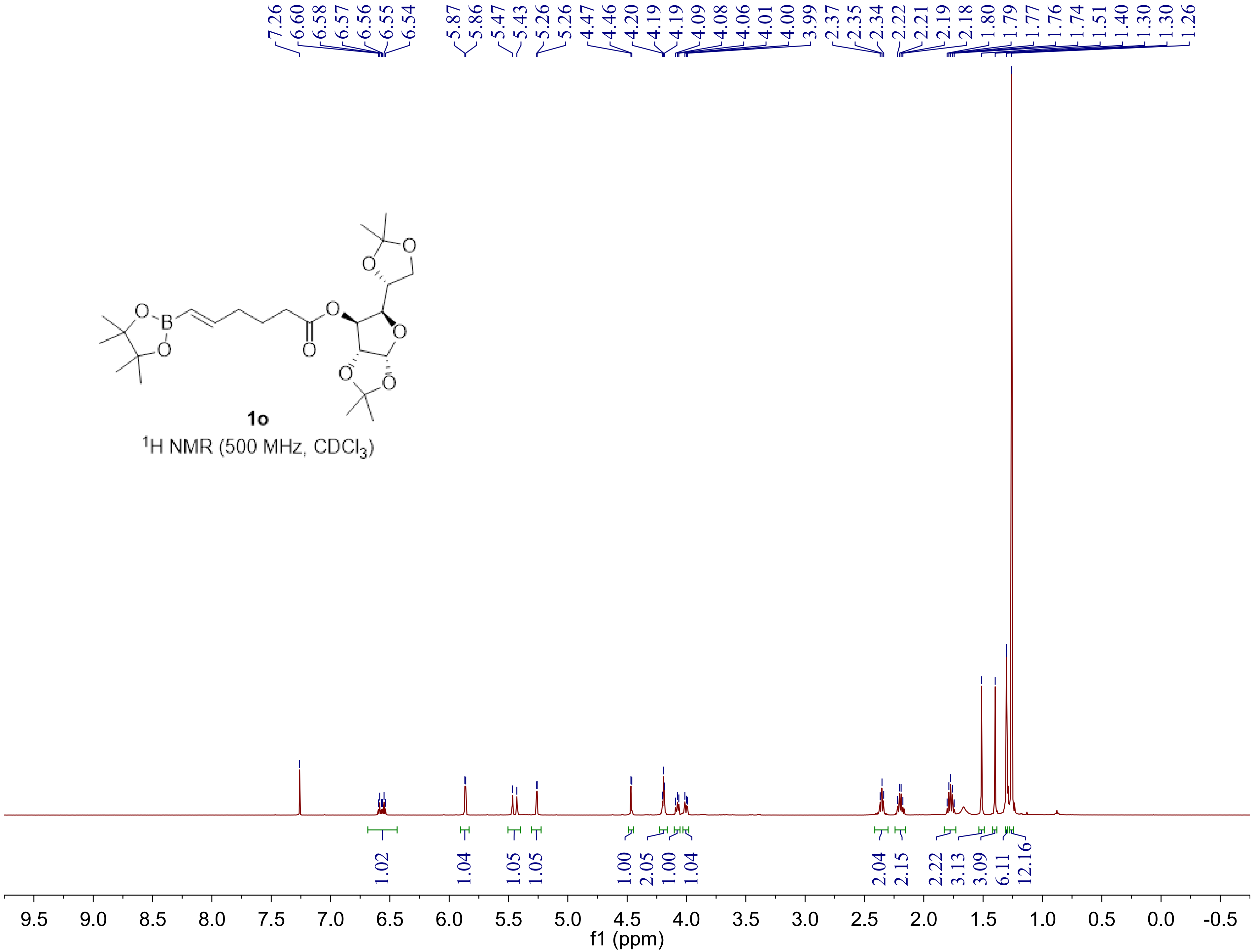

1o

^{1}H NMR (500 MHz, $CDCl_3$)

Supplementary Figure 156: ^{1}H NMR (500 MHz, $CDCl_3$) spectrum of **1o**.

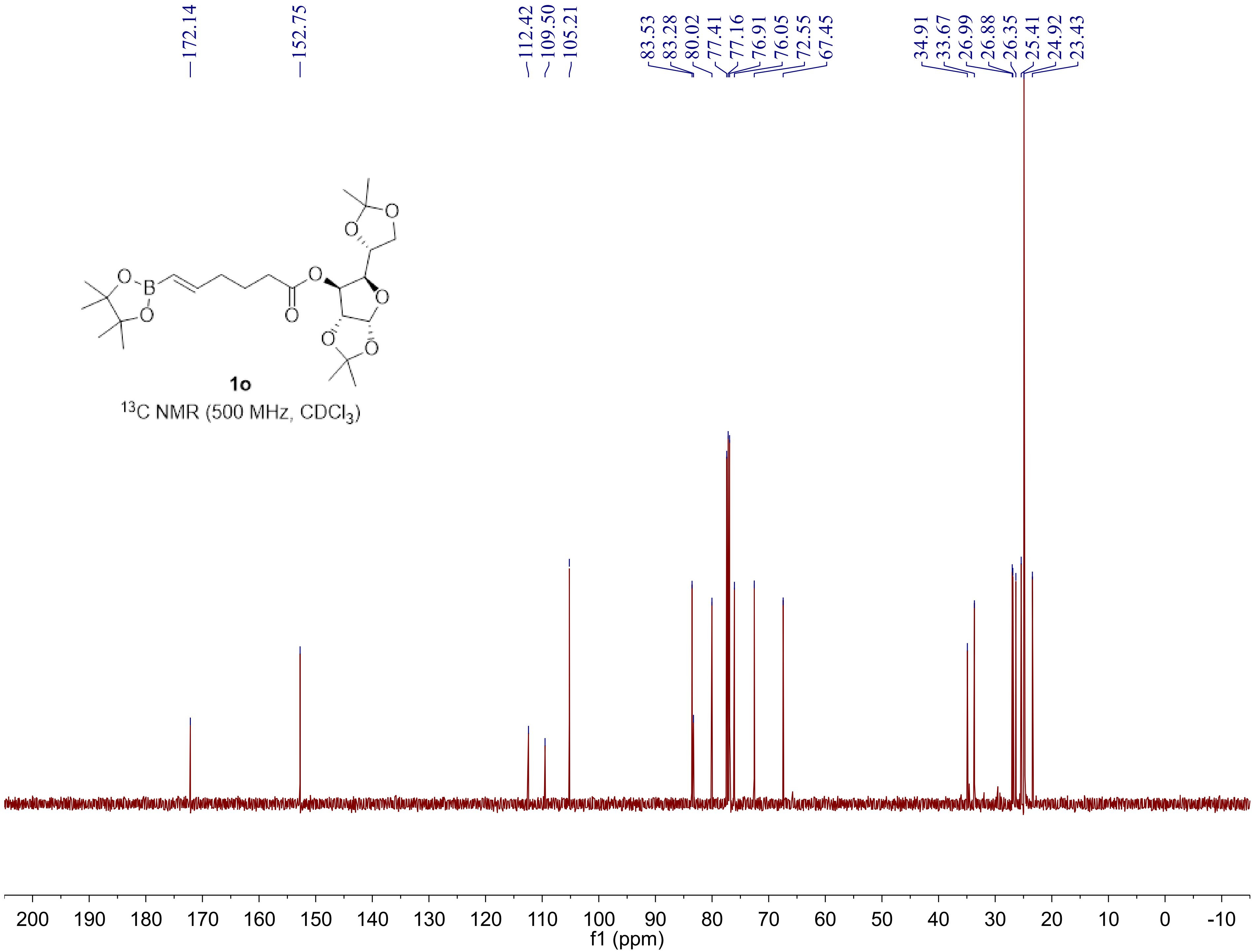

Supplementary Figure 157: ^{13}C NMR (126 MHz, $CDCl_3$) spectrum of **1o**.

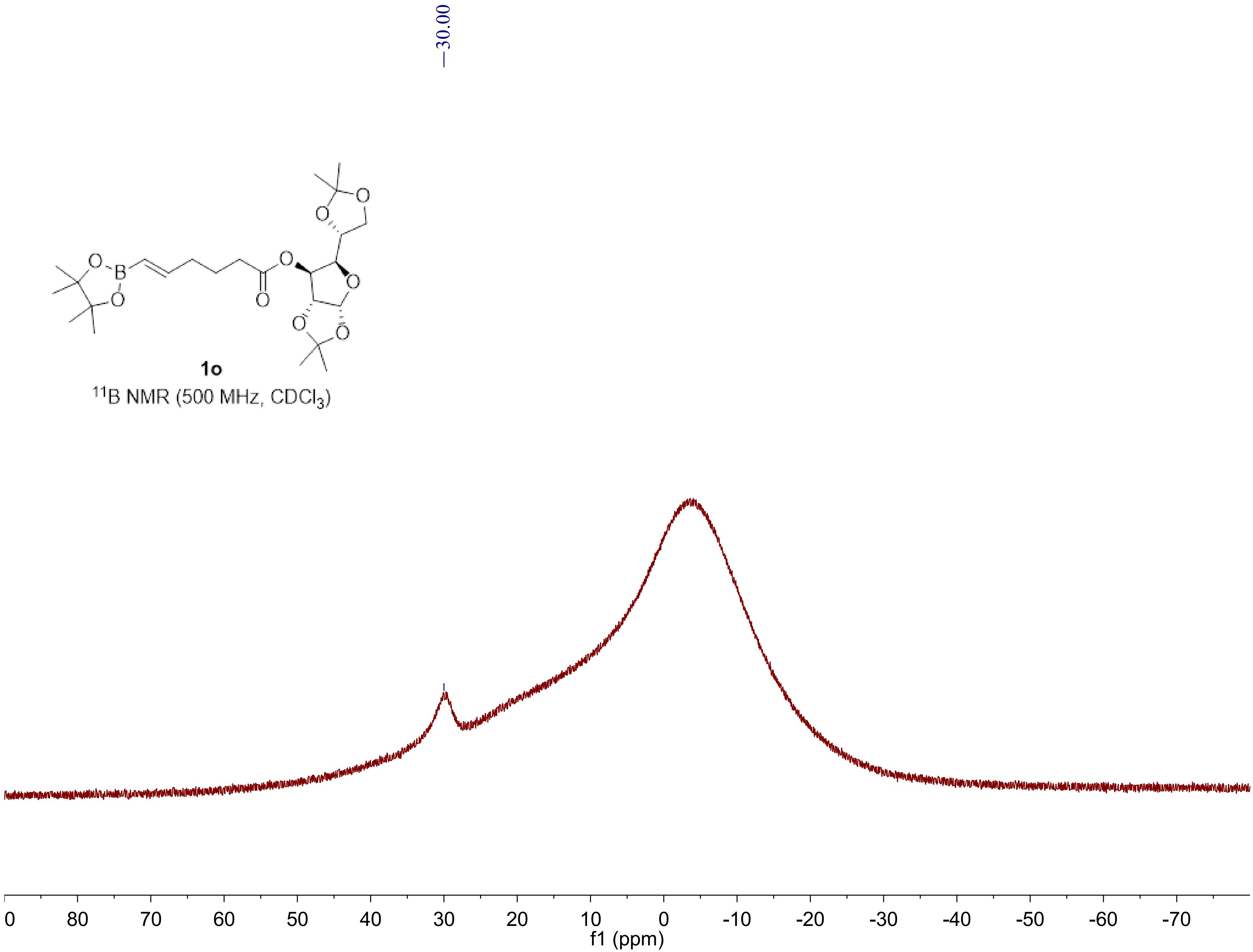

Supplementary Figure 158: ^{11}B NMR (160 MHz, $CDCl_3$) spectrum of **1o**.

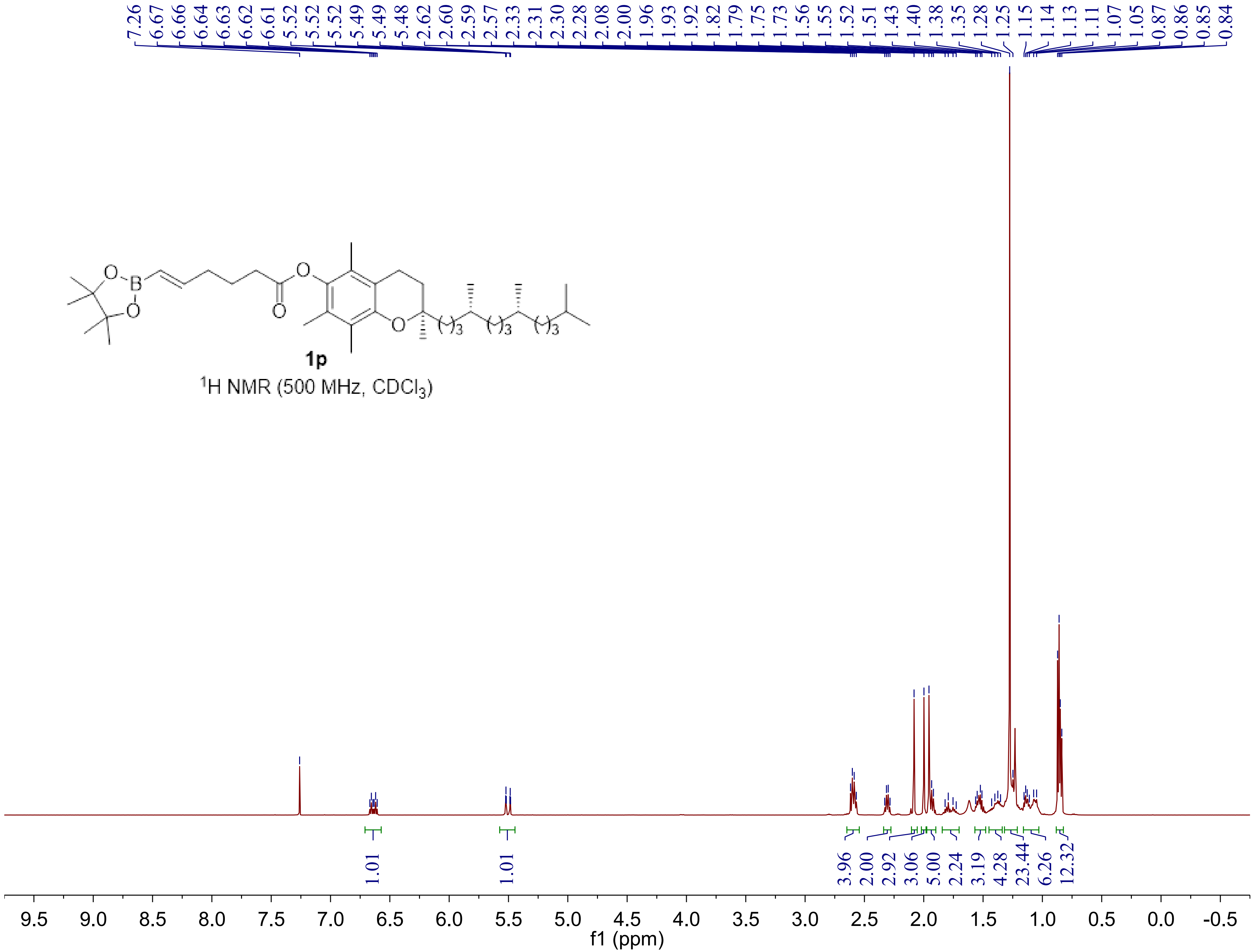

Supplementary Figure 159: ^{1}H NMR (500 MHz, $CDCl_3$) spectrum of **1p**.

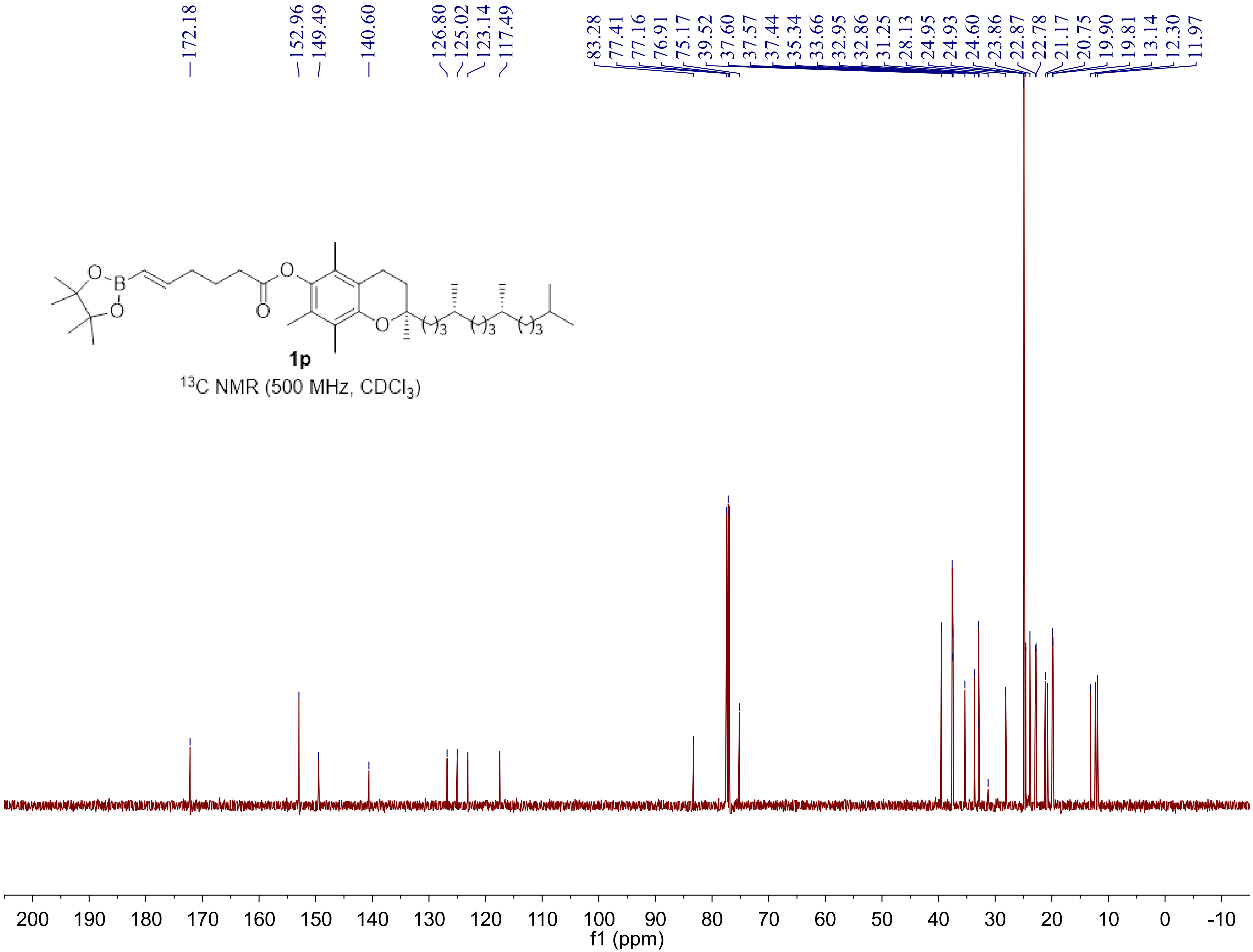

Supplementary Figure 160: ^{13}C NMR (126 MHz, $CDCl_3$) spectrum of **1p**.

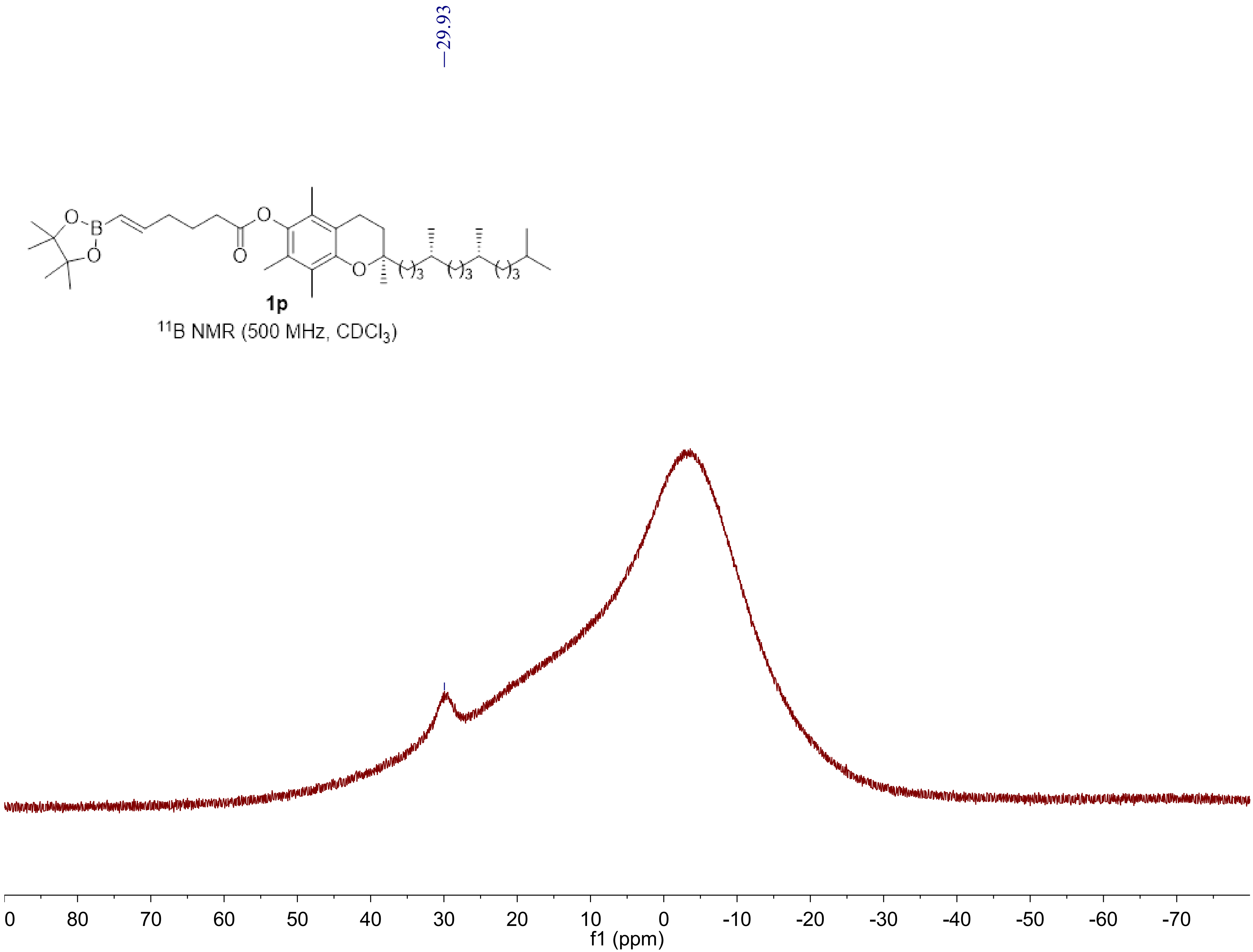

Supplementary Figure 161: ^{11}B NMR (160 MHz, $CDCl_3$) spectrum of **1p**.

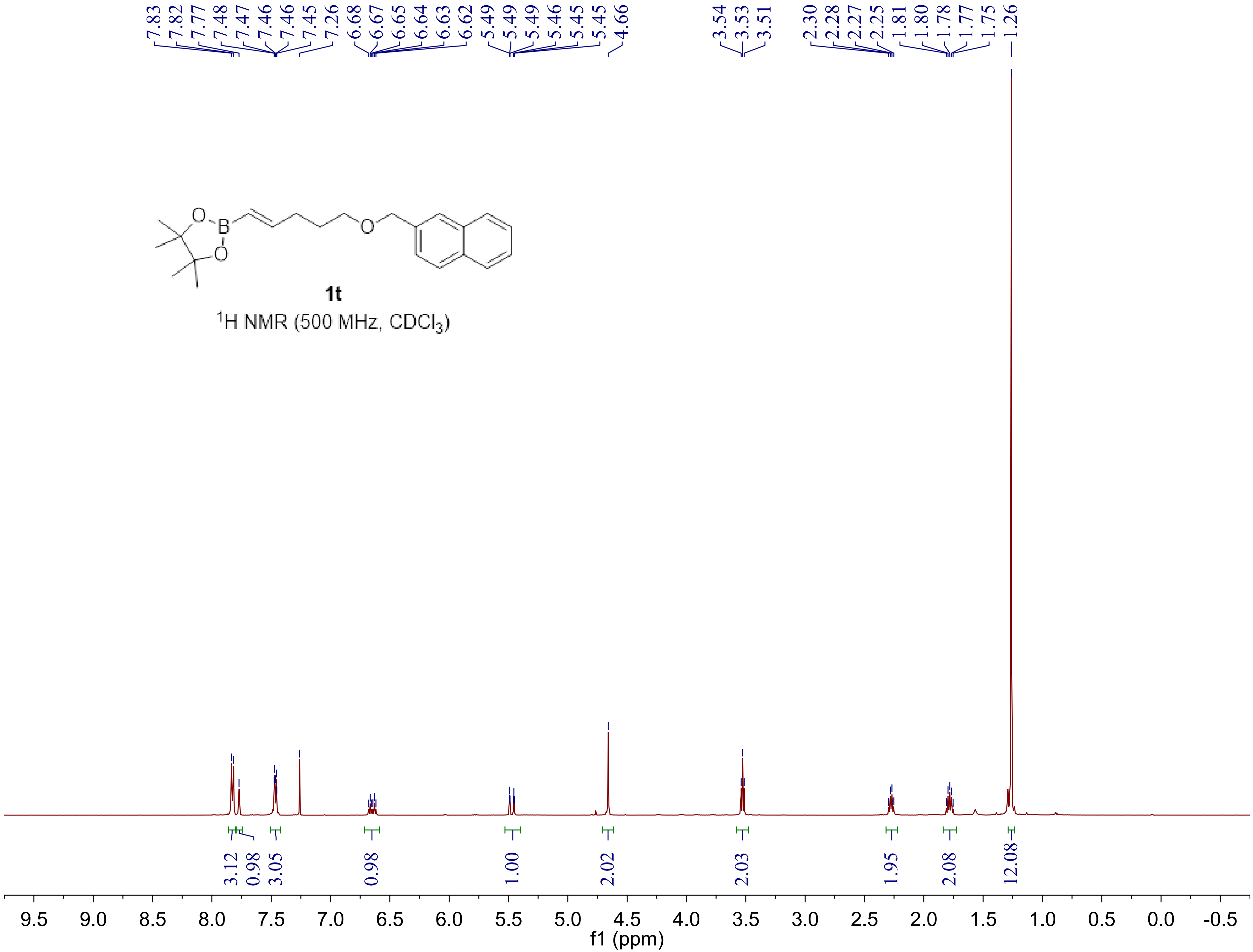

Supplementary Figure 162: ^{1}H NMR (500 MHz, $CDCl_3$) spectrum of **1t**.

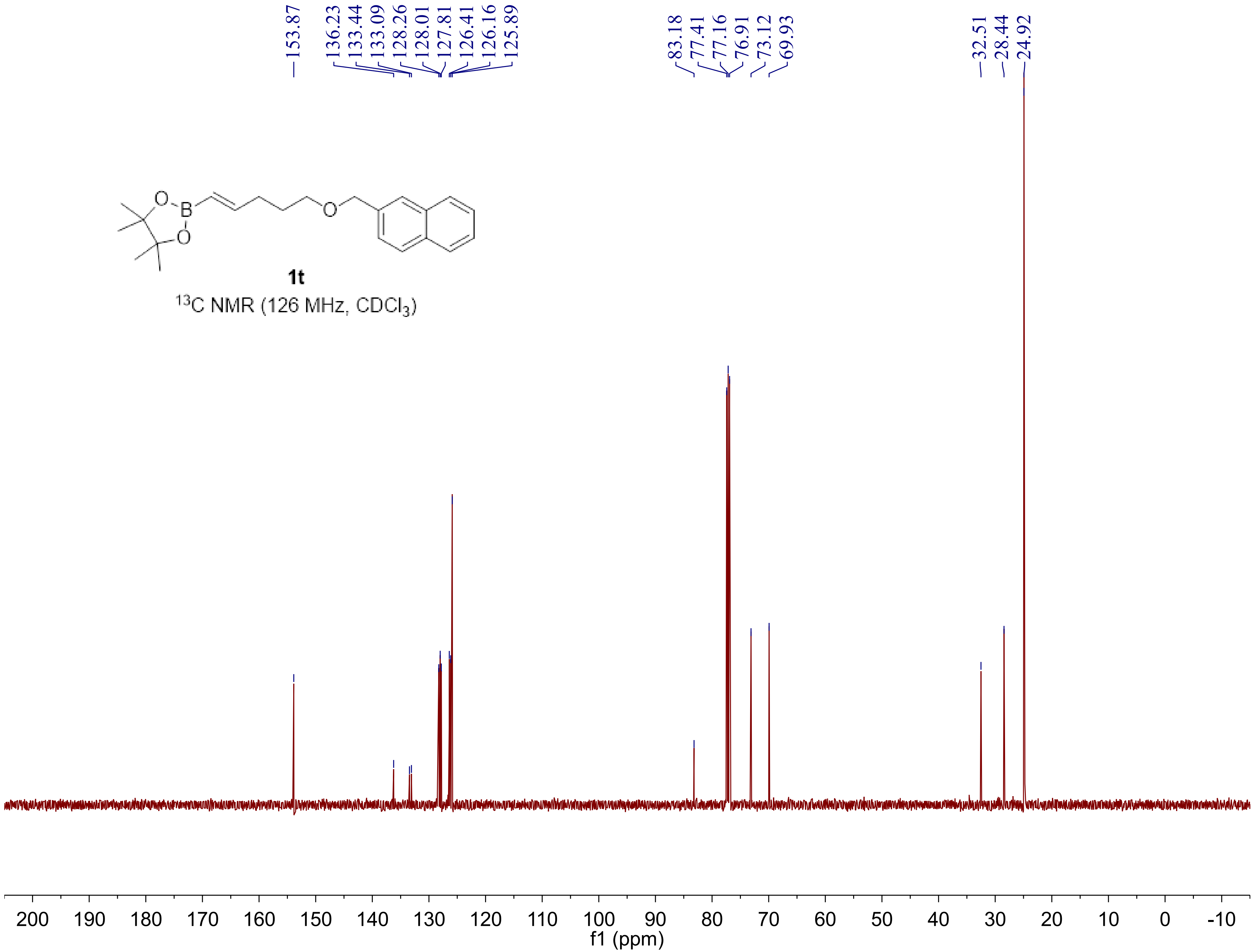

Supplementary Figure 163: ^{13}C NMR (126 MHz, $CDCl_3$) spectrum of **1t**.

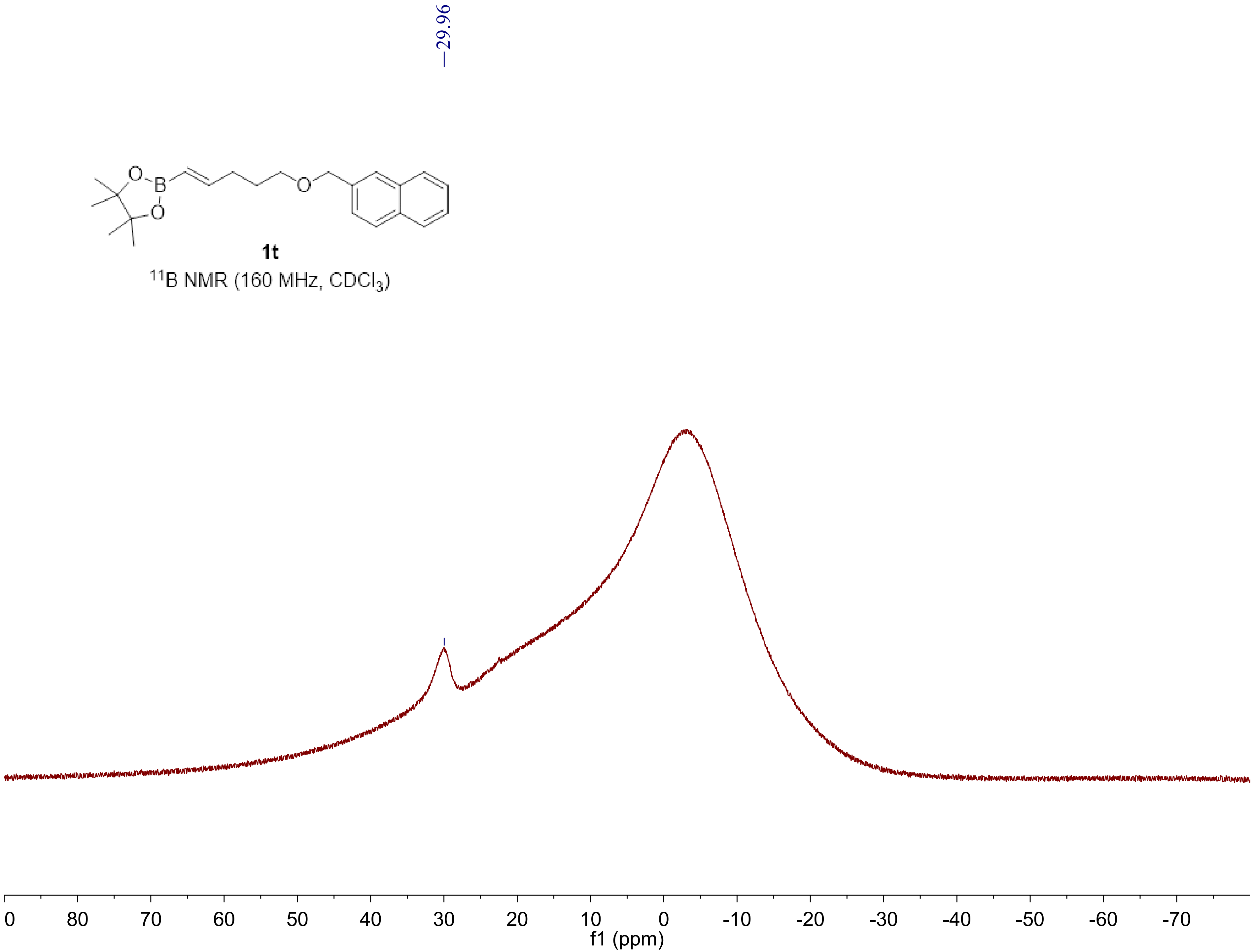

Supplementary Figure 164: ^{11}B NMR (160 MHz, $CDCl_3$) spectrum of **1t**.

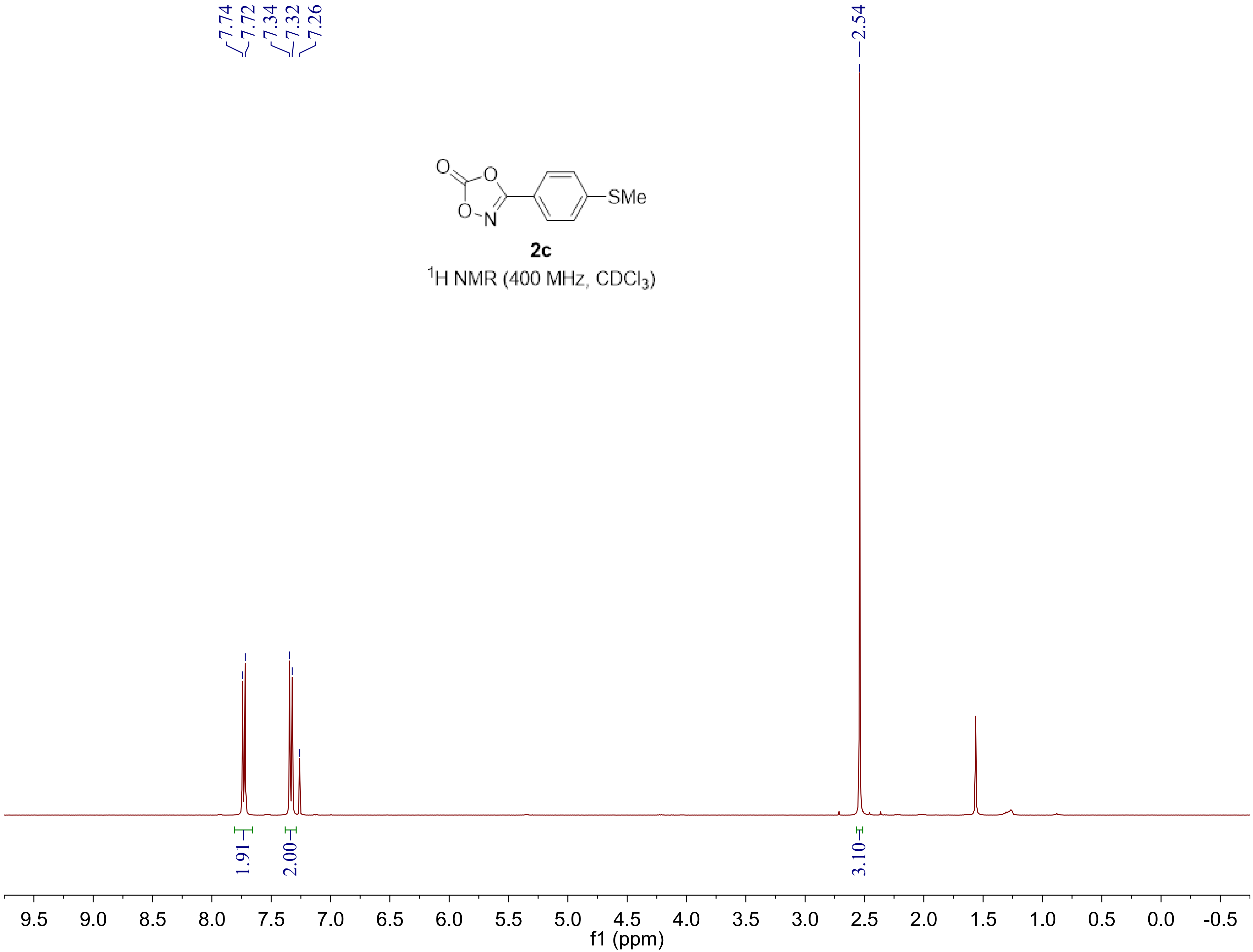

Supplementary Figure 165: ^{1}H NMR (400 MHz, $CDCl_3$) spectrum of **2c**.

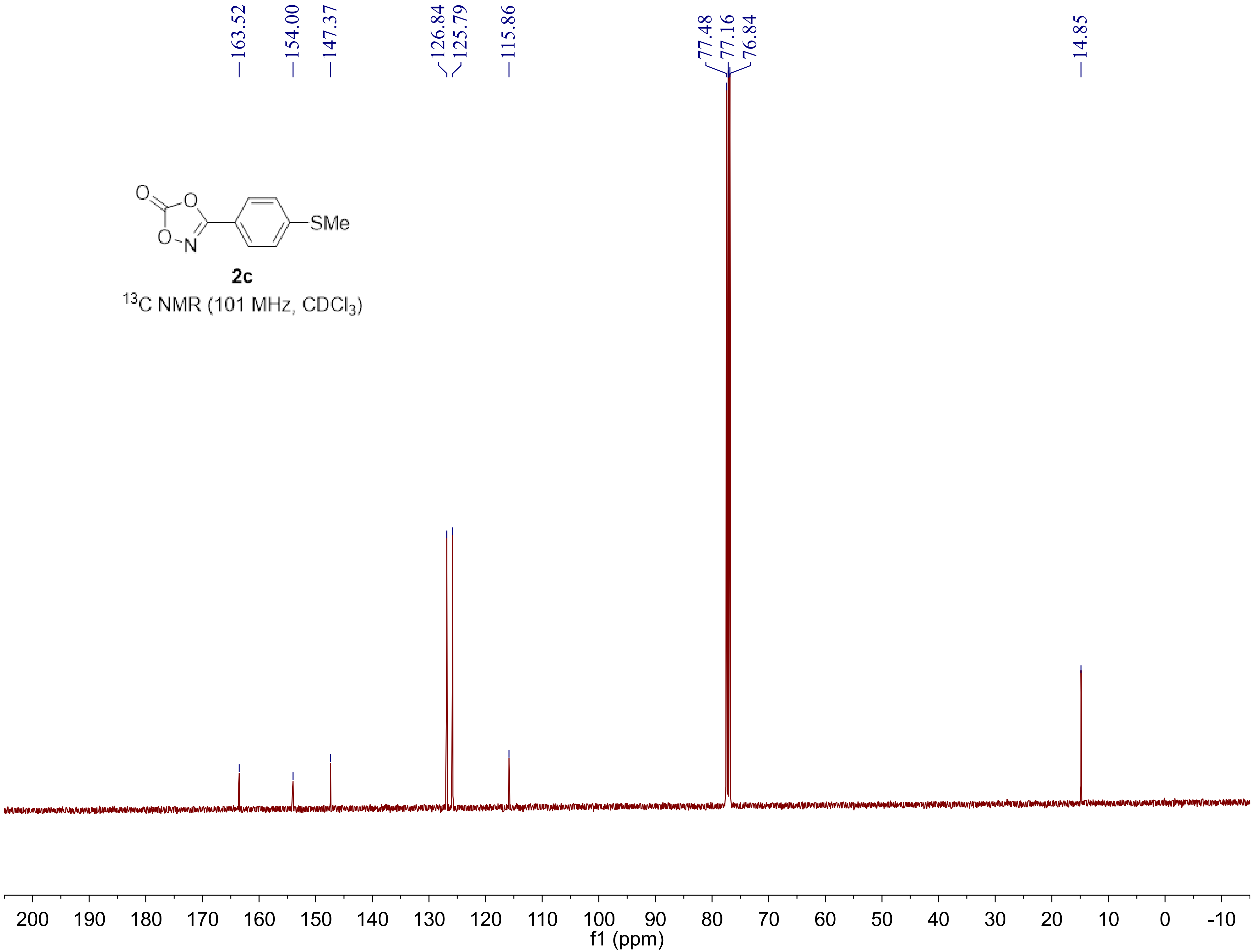

Supplementary Figure 166: ^{13}C NMR (101 MHz, $CDCl_3$) spectrum of **2c**.

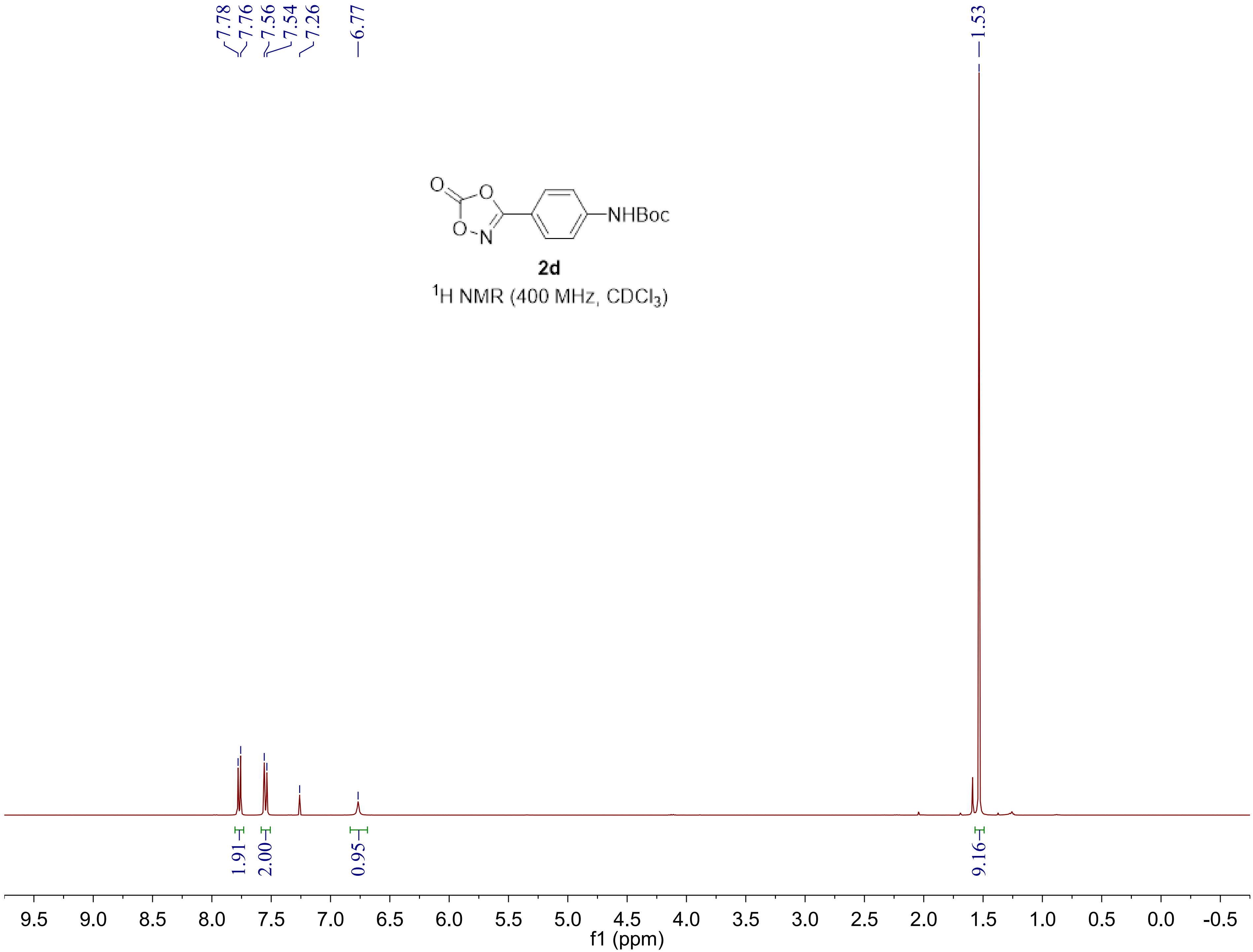

Supplementary Figure 167: ^{1}H NMR (400 MHz, $CDCl_3$) spectrum of **2d**.

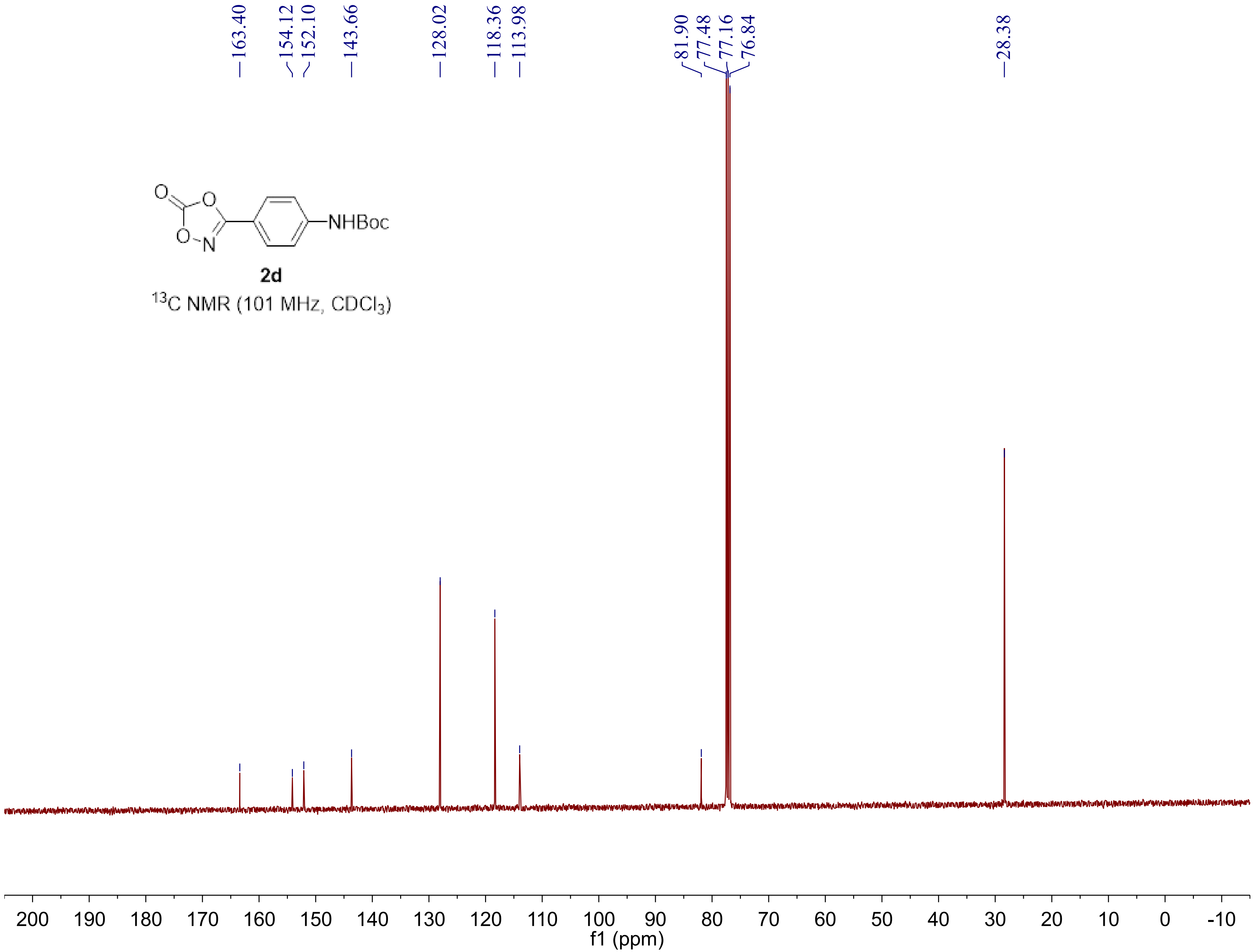

Supplementary Figure 168: ^{13}C NMR (101 MHz, $CDCl_3$) spectrum of **2d**.

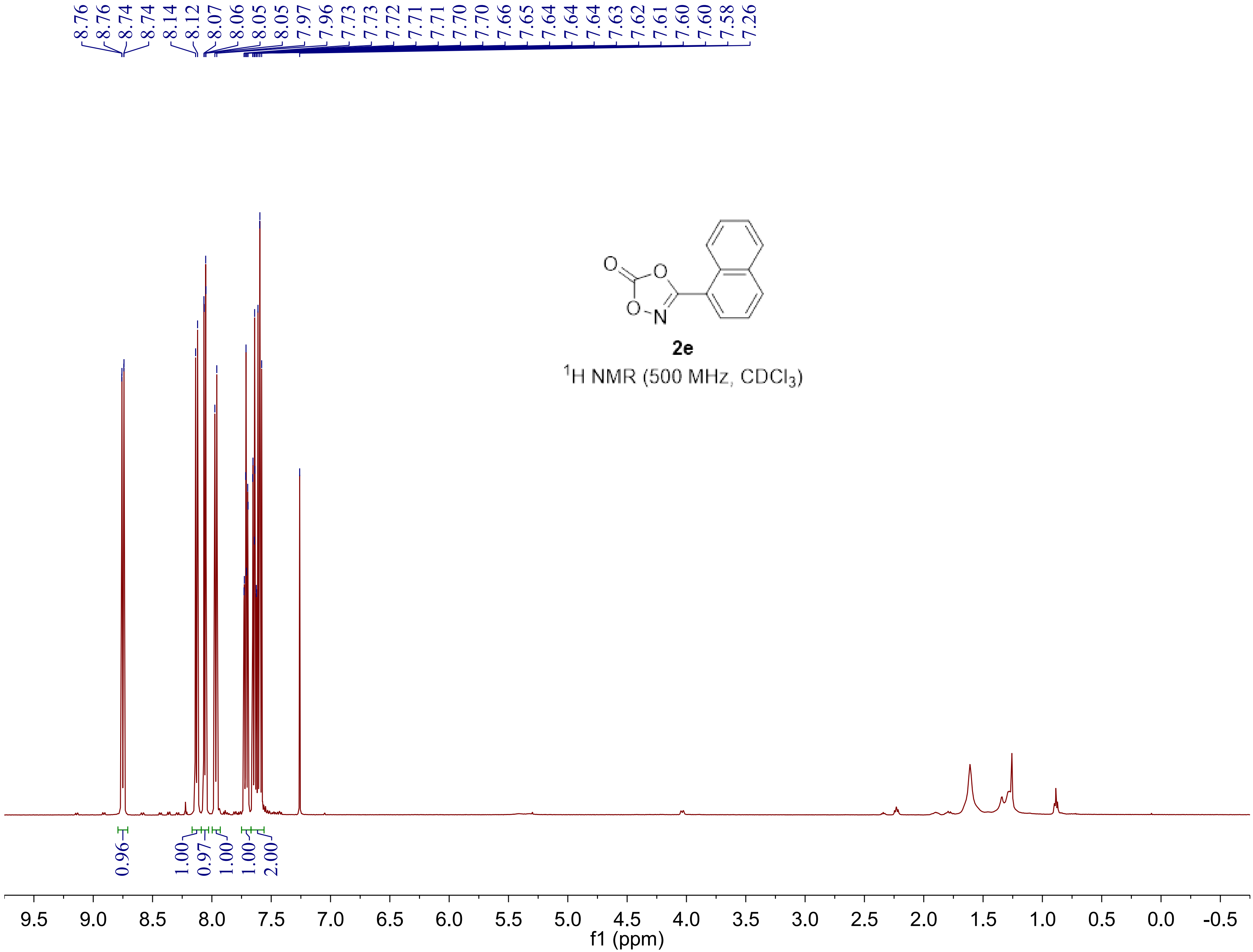

Supplementary Figure 169: ^{1}H NMR (500 MHz, $CDCl_3$) spectrum of **2e**.

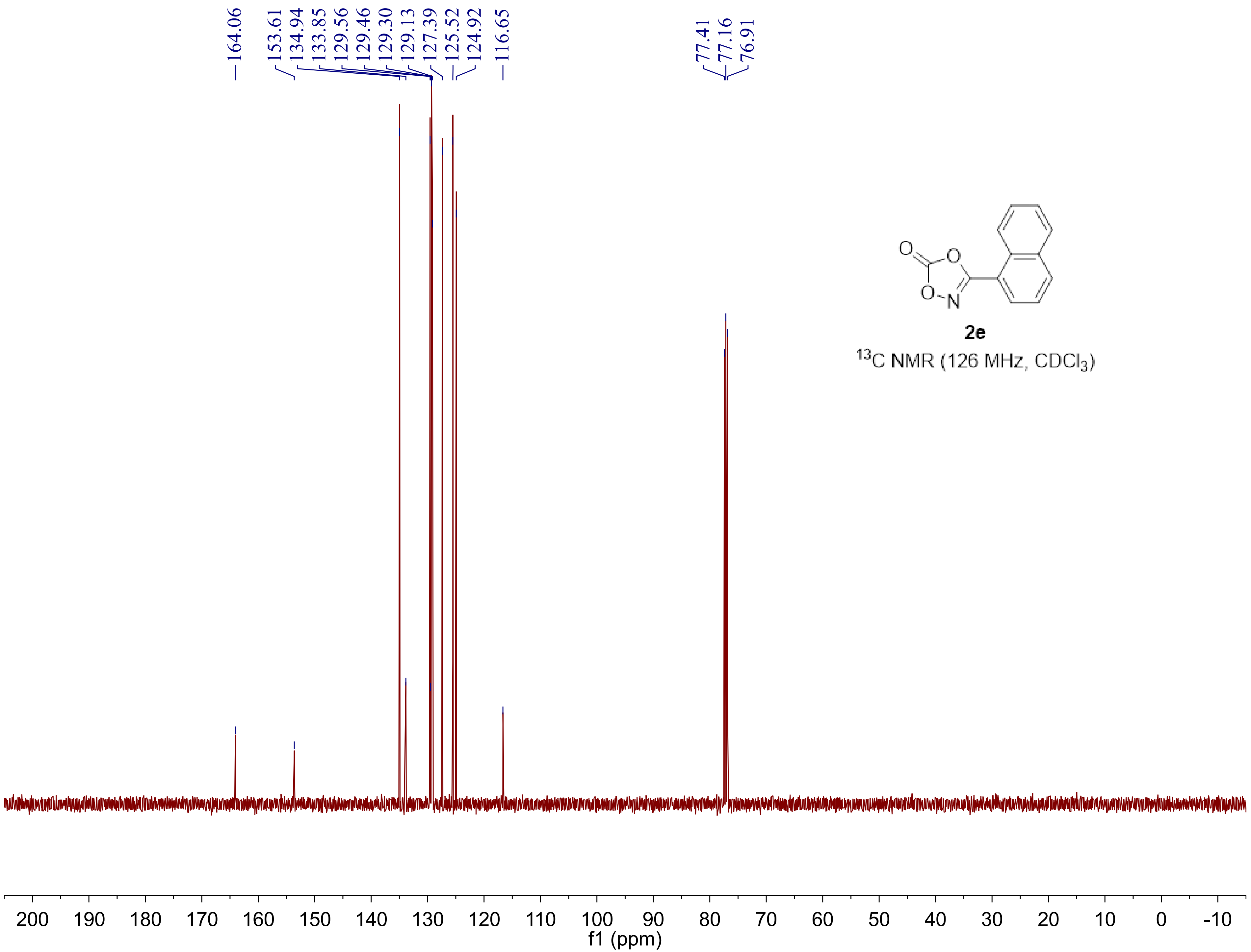

Supplementary Figure 170: ^{13}C NMR (126 MHz, $CDCl_3$) spectrum of **2e**.

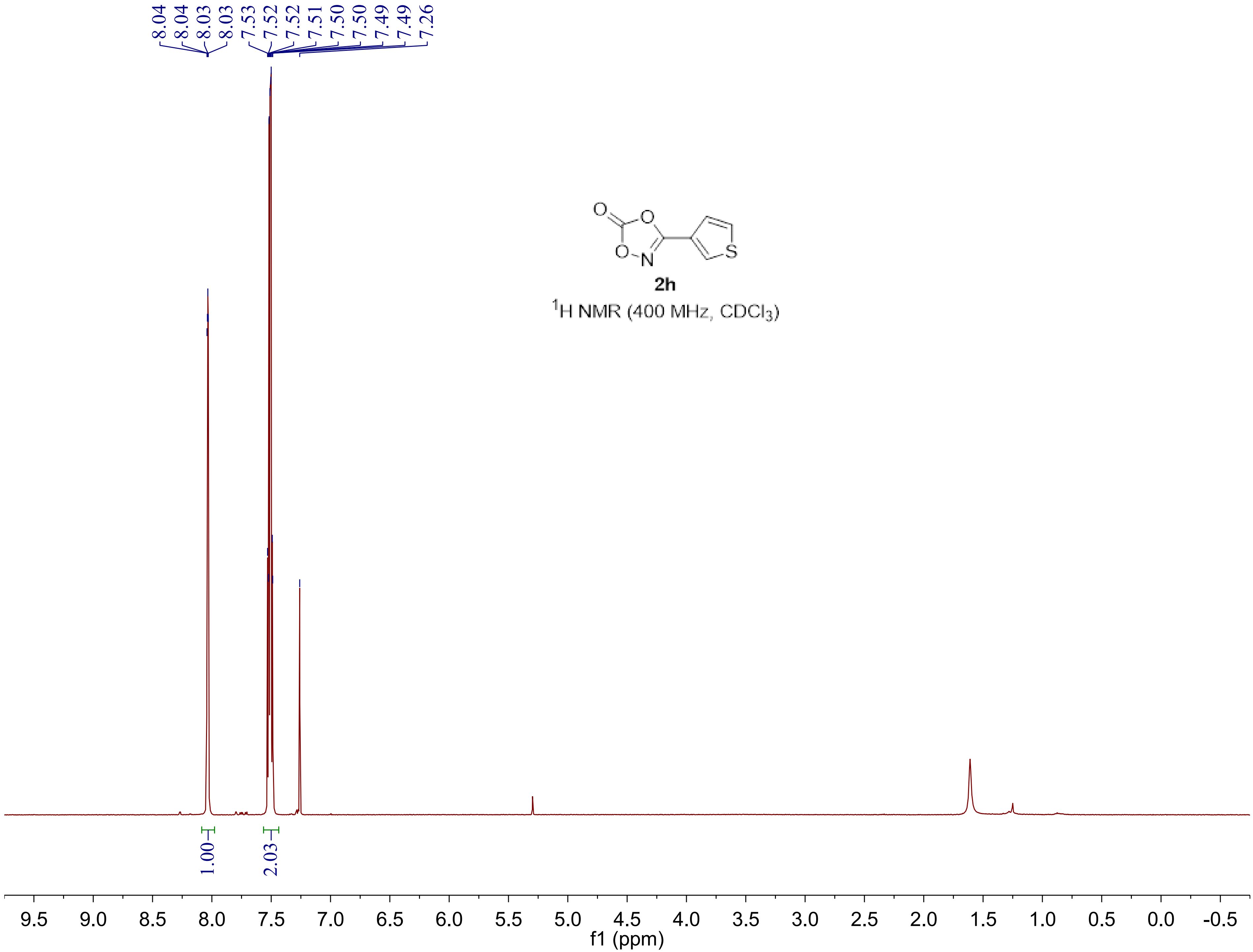

Supplementary Figure 171: ^{1}H NMR (400 MHz, $CDCl_3$) spectrum of **2h**.

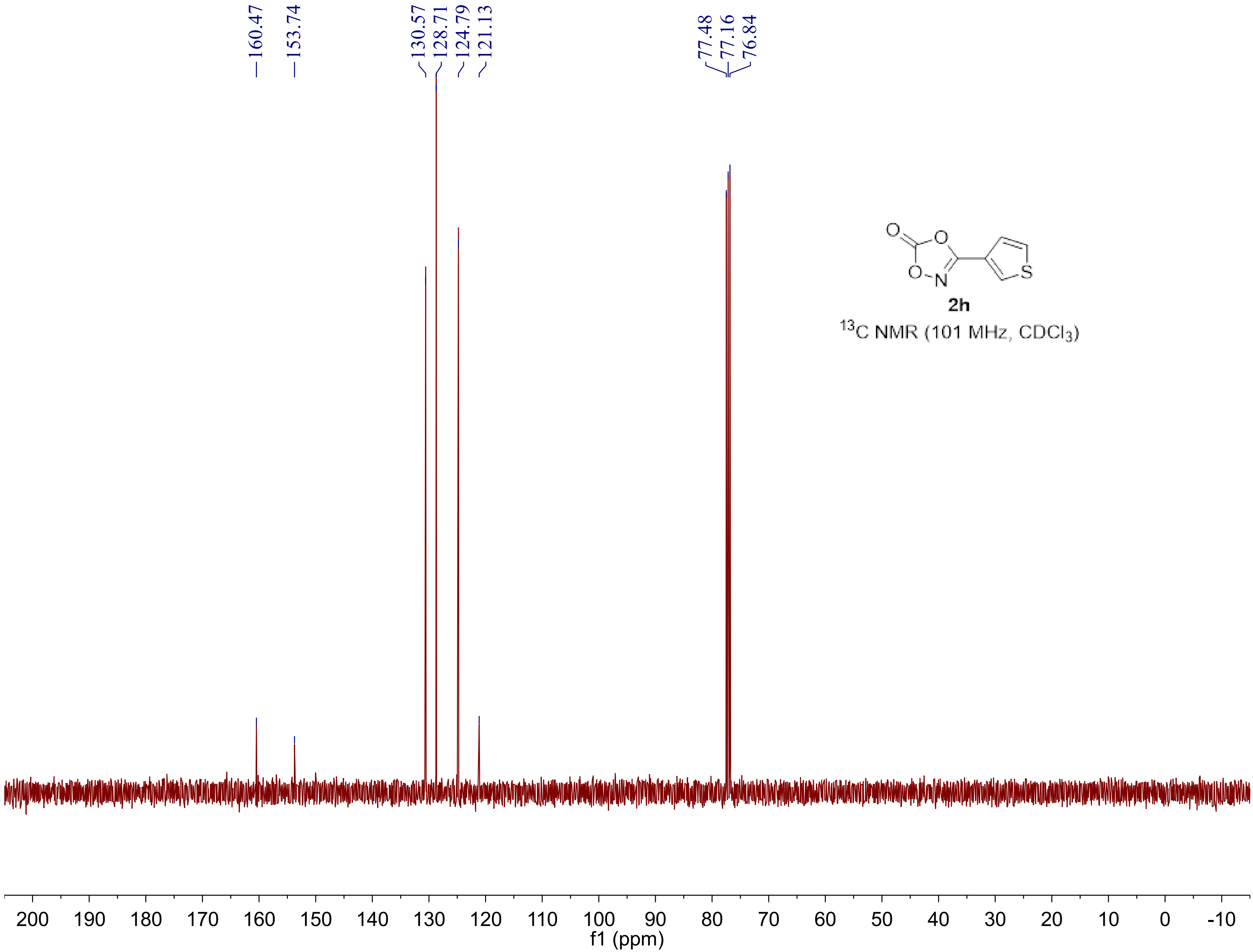

Supplementary Figure 172: ^{13}C NMR (101 MHz, $CDCl_3$) spectrum of **2h**.

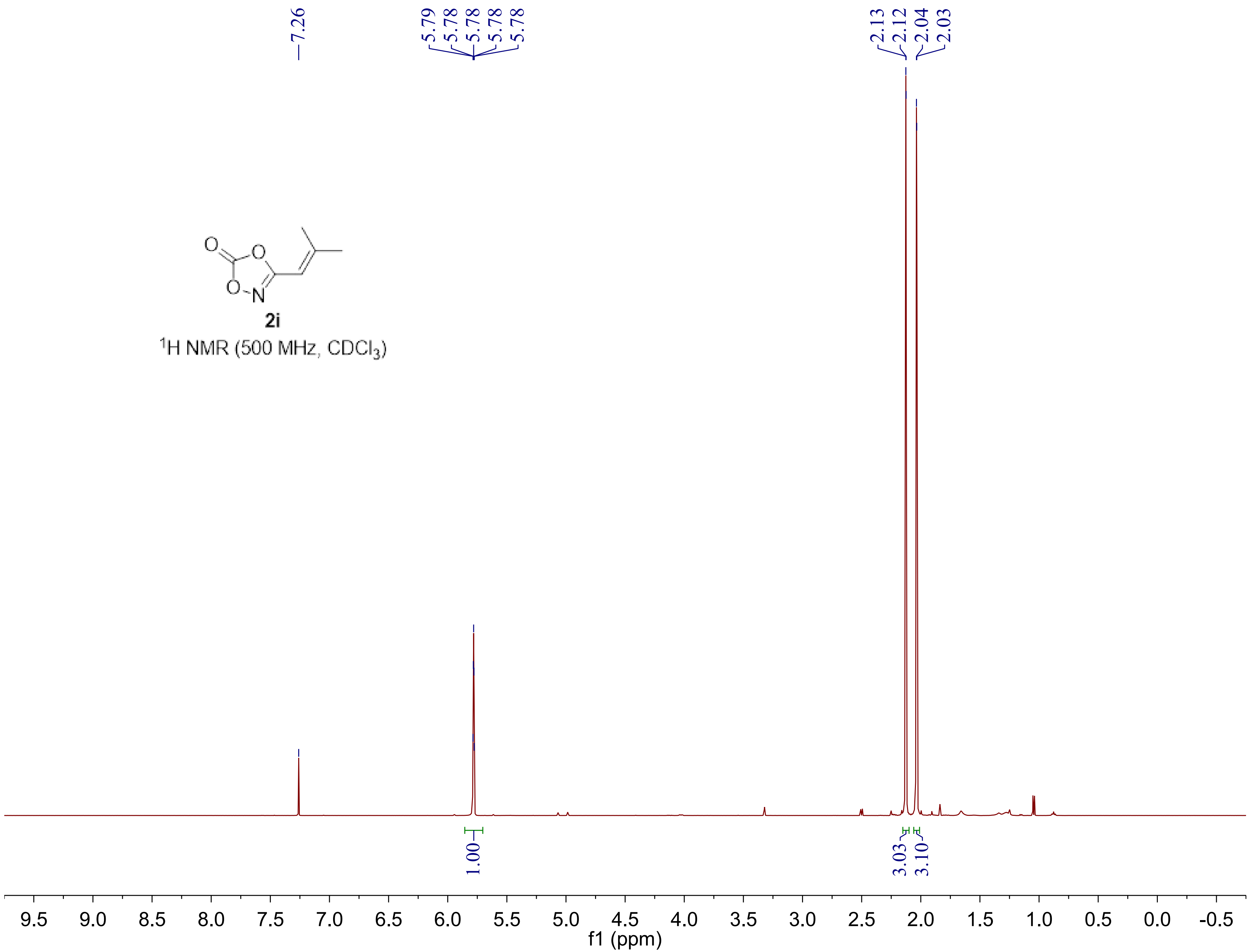

Supplementary Figure 173: ^{1}H NMR (500 MHz, $CDCl_3$) spectrum of **2i**.

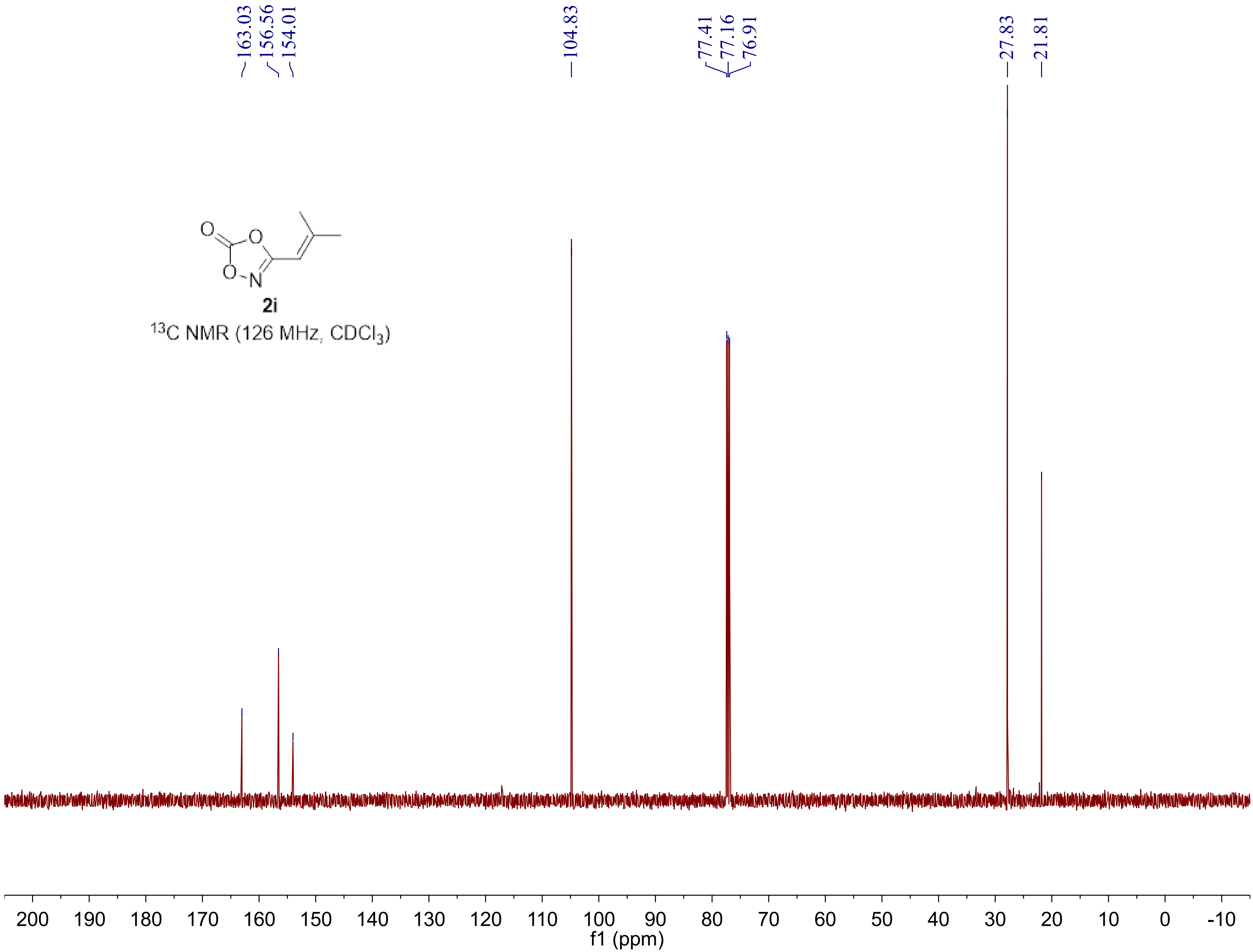

Supplementary Figure 174: ^{13}C NMR (126 MHz, $CDCl_3$) spectrum of **2i**.

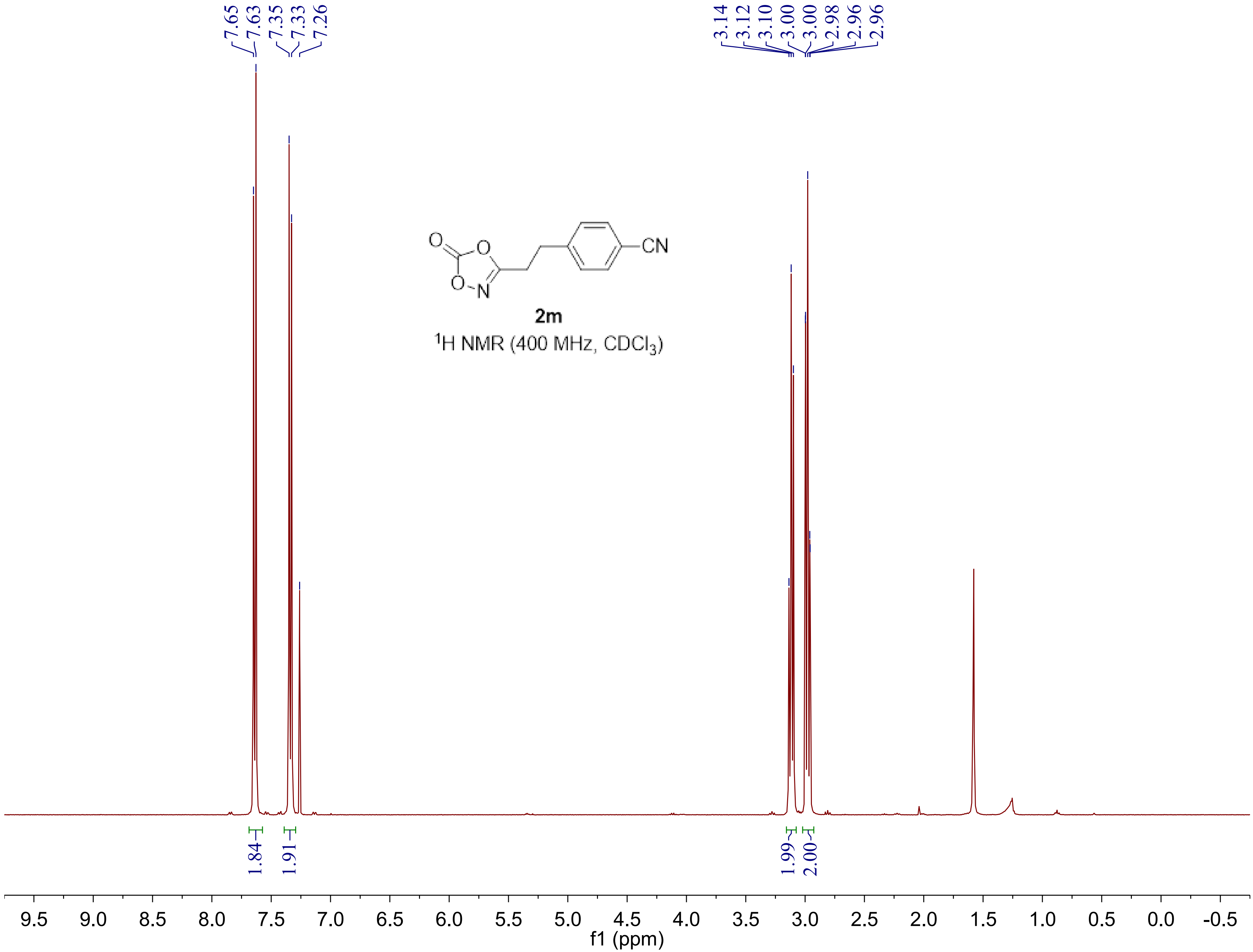

Supplementary Figure 175: ^{1}H NMR (400 MHz, $CDCl_3$) spectrum of **2m**.

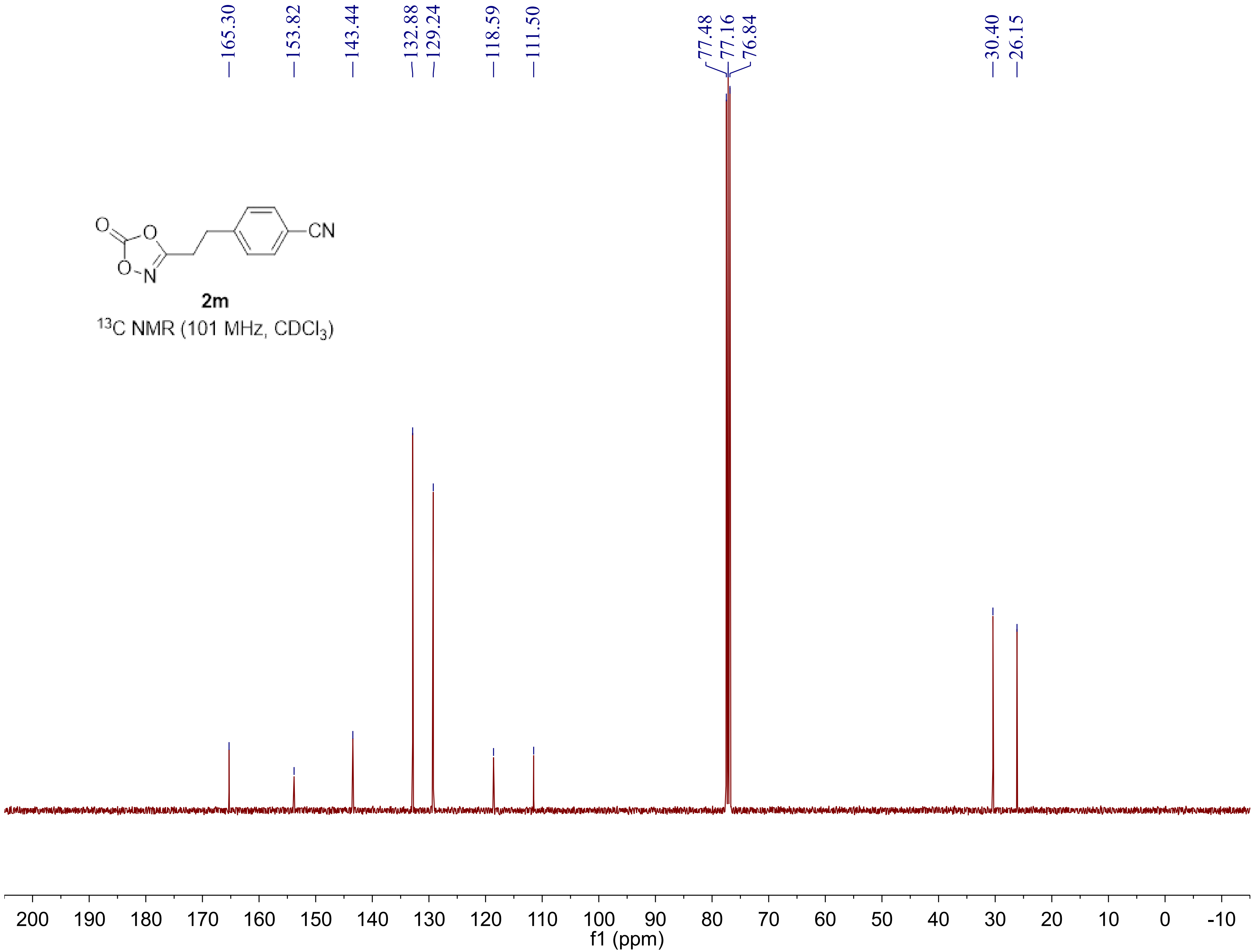

Supplementary Figure 176: ^{13}C NMR (101 MHz, $CDCl_3$) spectrum of **2m**.

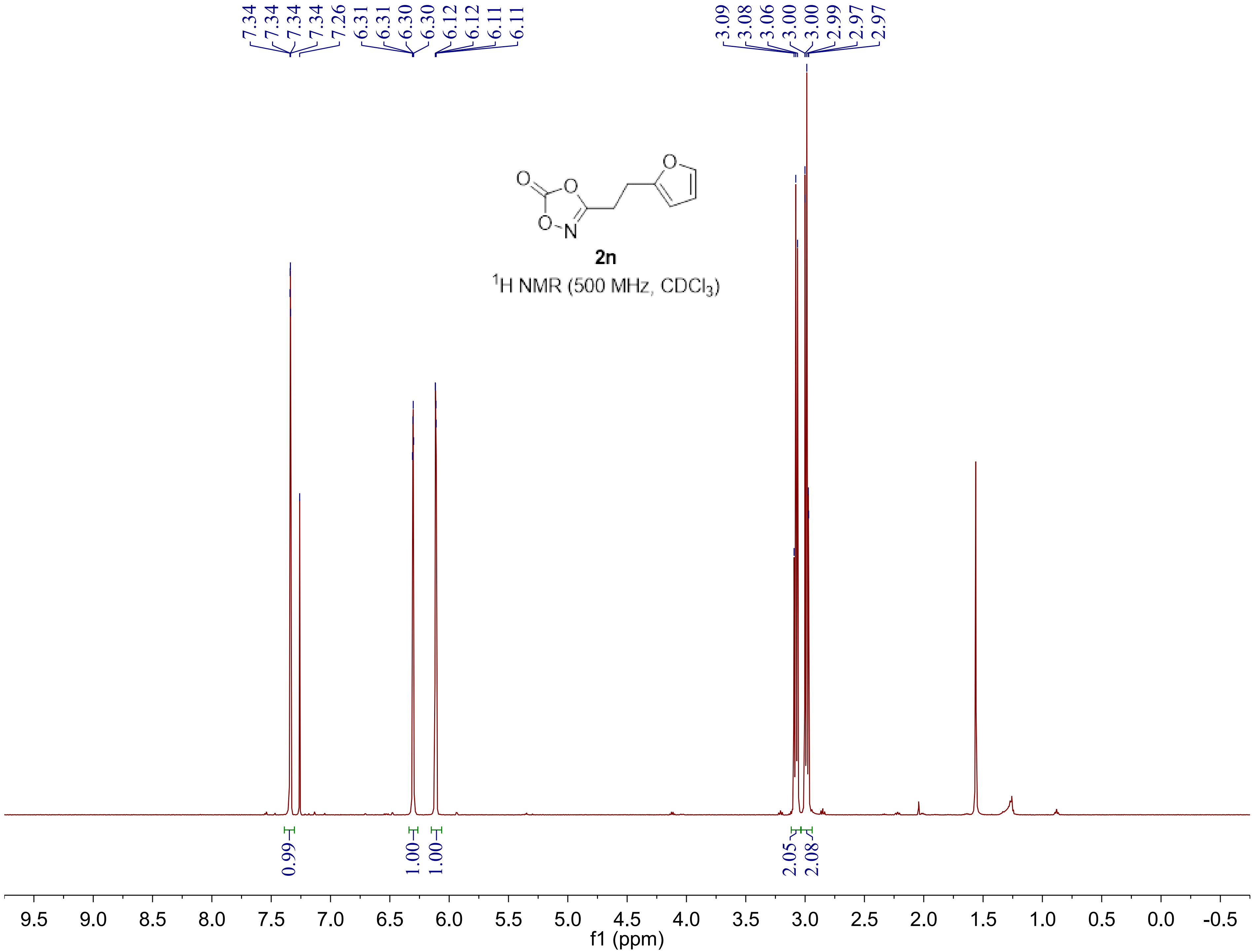

Supplementary Figure 177: ^{1}H NMR (500 MHz, $CDCl_3$) spectrum of **2n**.

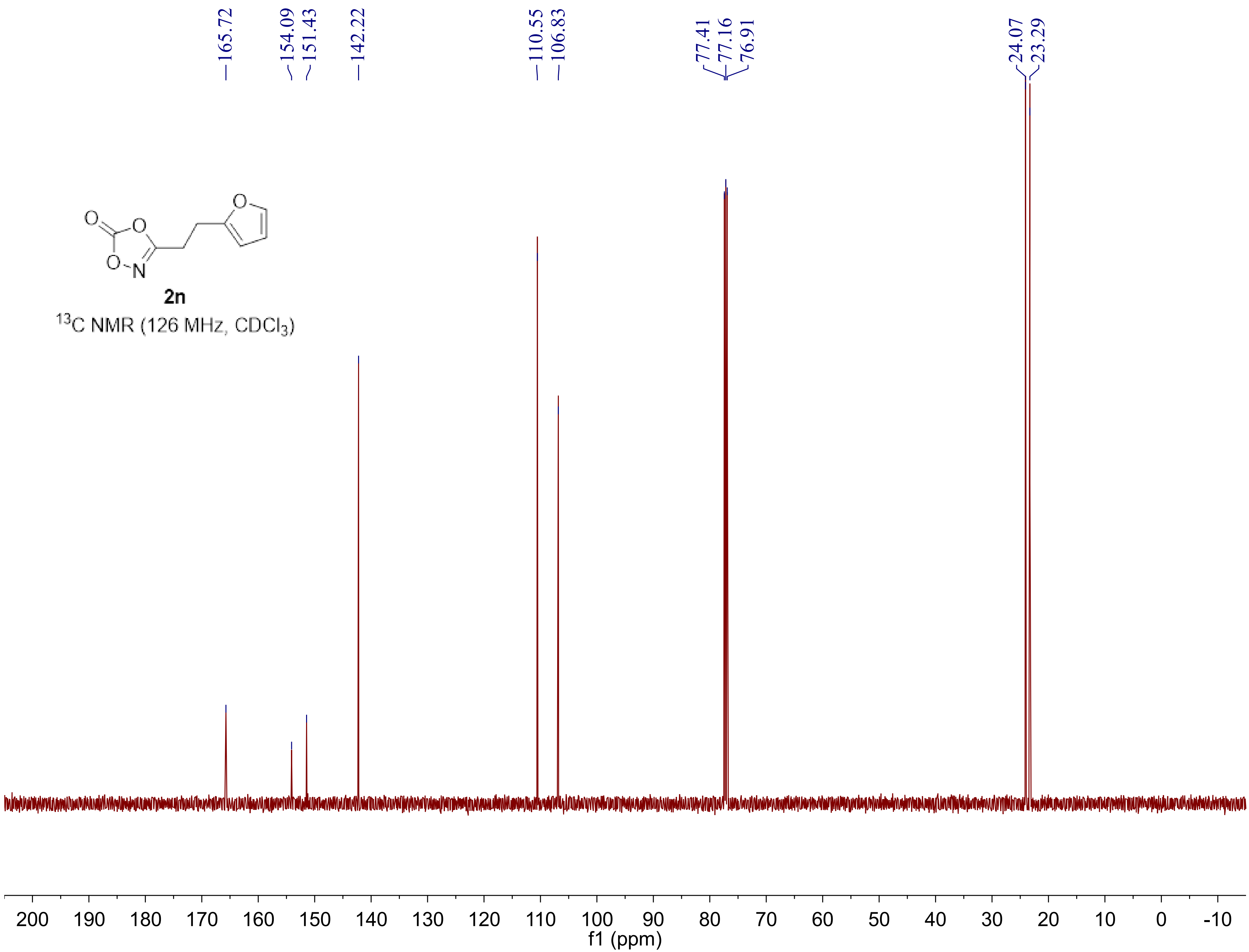

Supplementary Figure 178: ^{13}C NMR (126 MHz, $CDCl_3$) spectrum of **2n**.

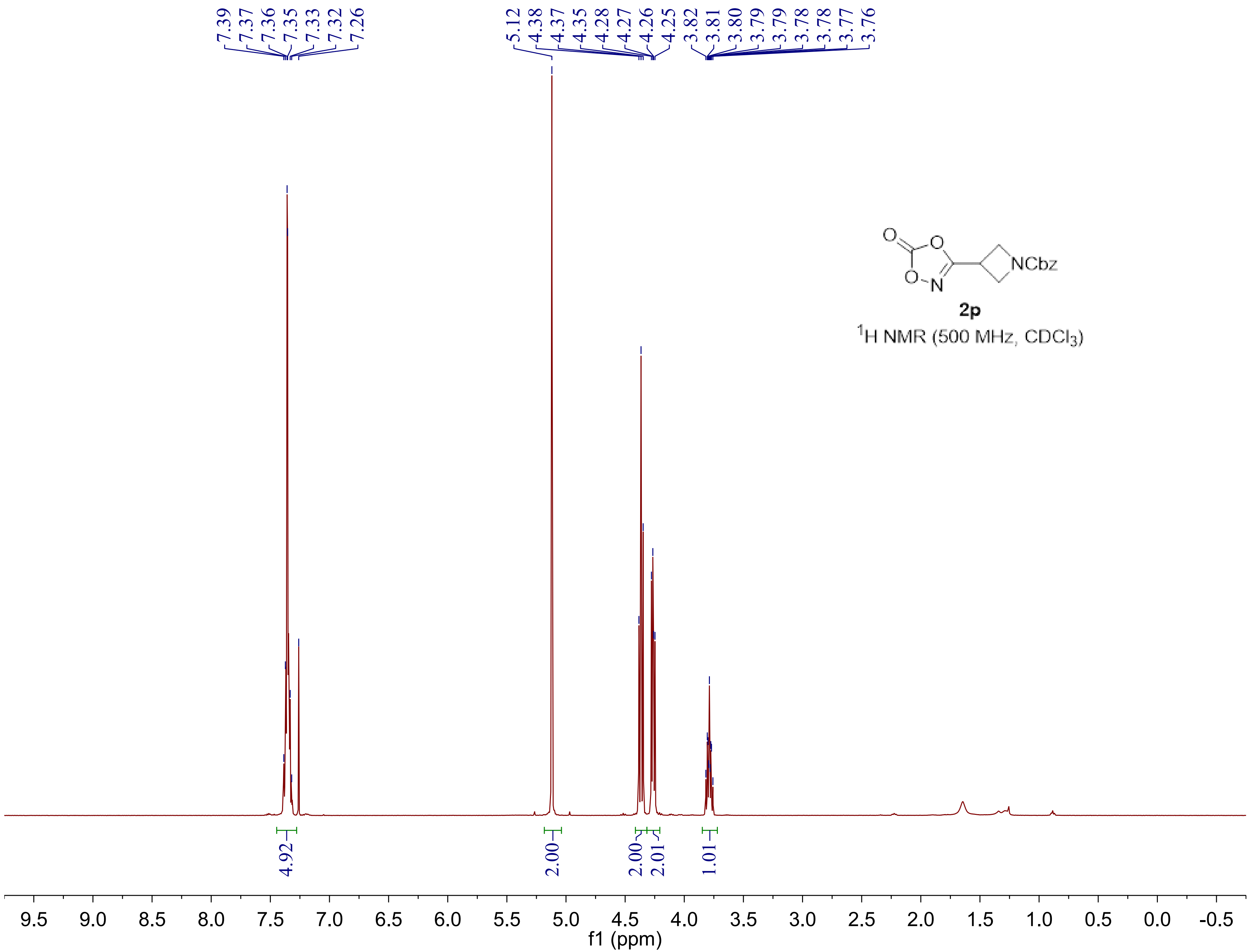

Supplementary Figure 179: ^{1}H NMR (500 MHz, $CDCl_3$) spectrum of **2p**.

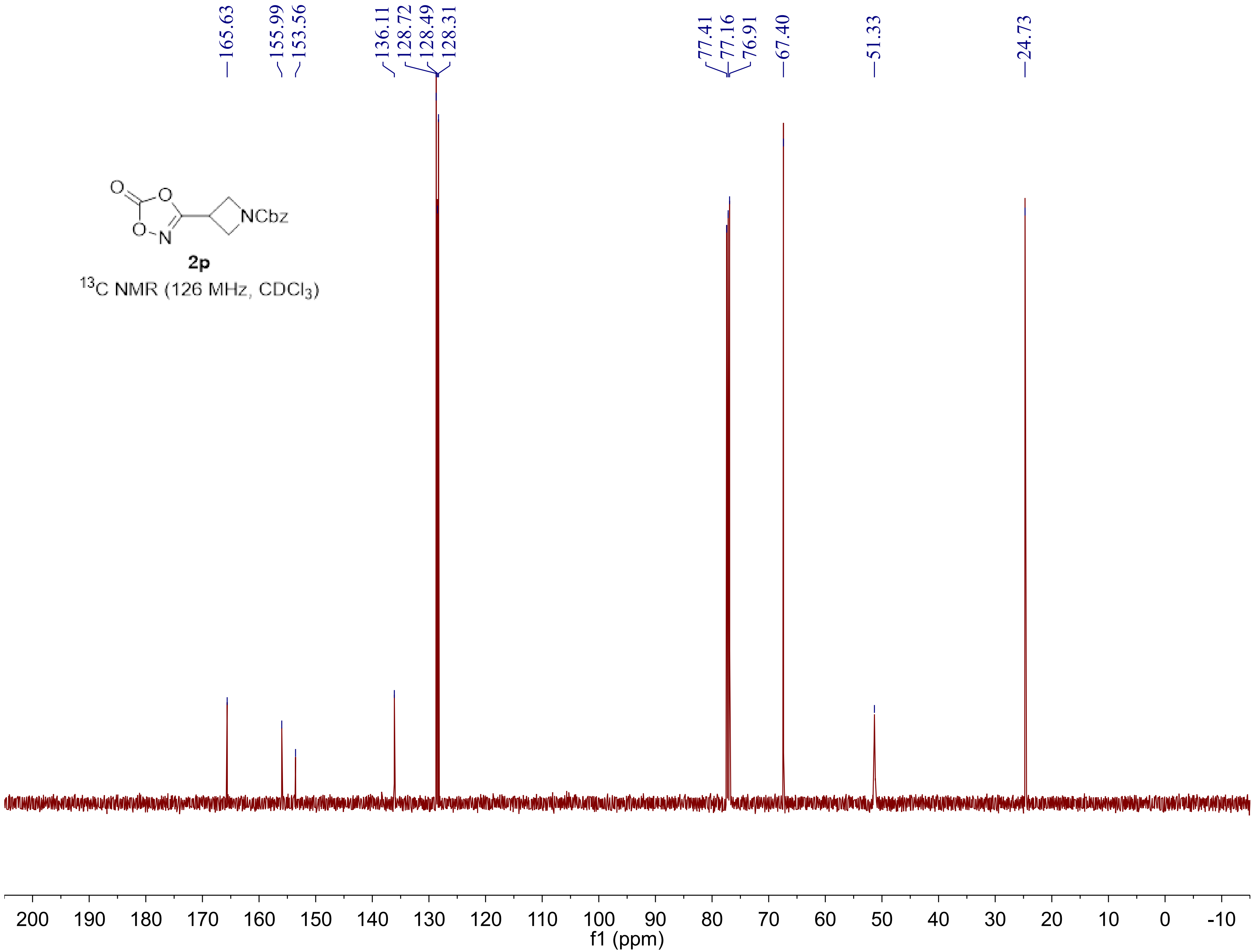

Supplementary Figure 180: ^{13}C NMR (126 MHz, $CDCl_3$) spectrum of **2p**.

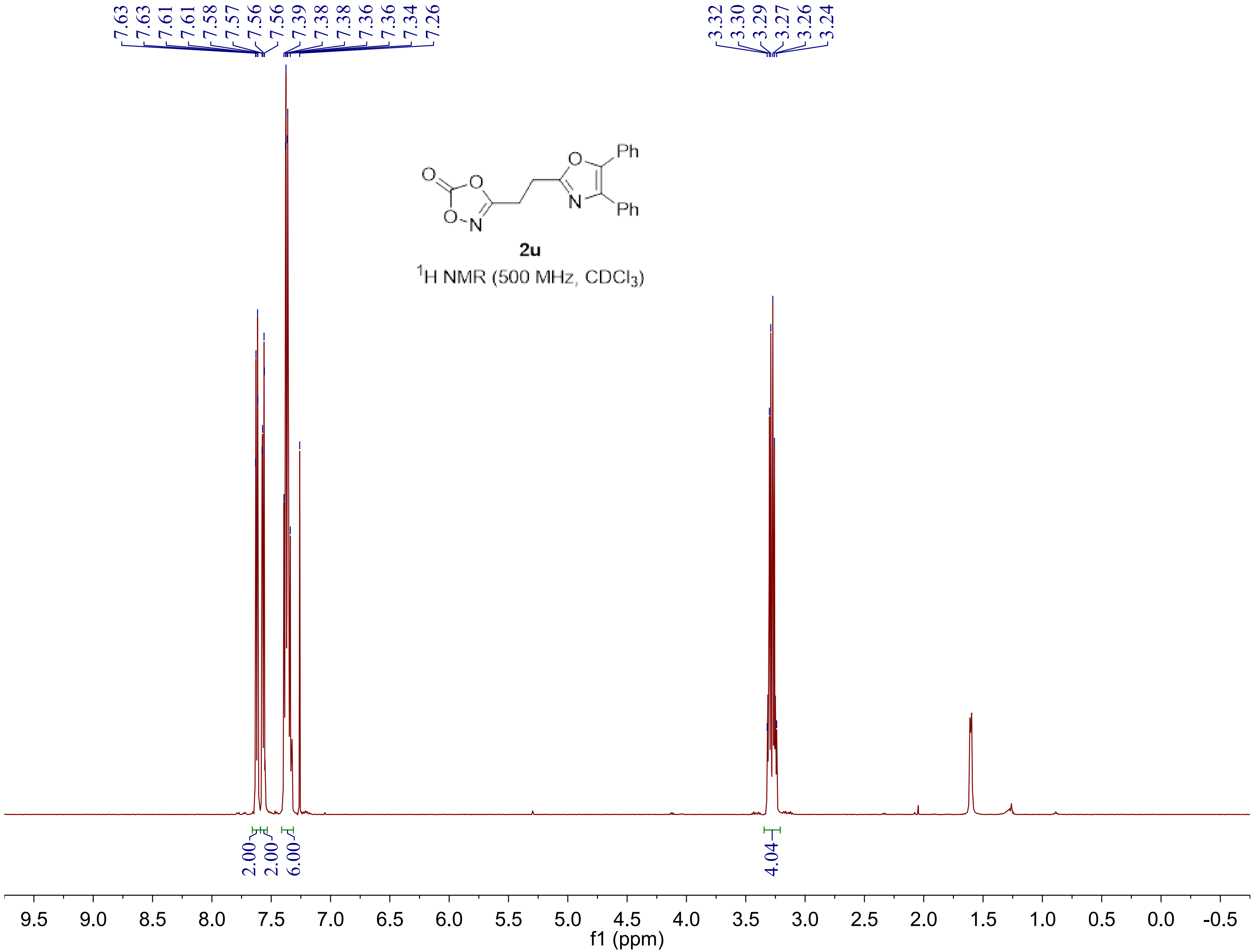

Supplementary Figure 181: ^{1}H NMR (500 MHz, $CDCl_3$) spectrum of **2u**.

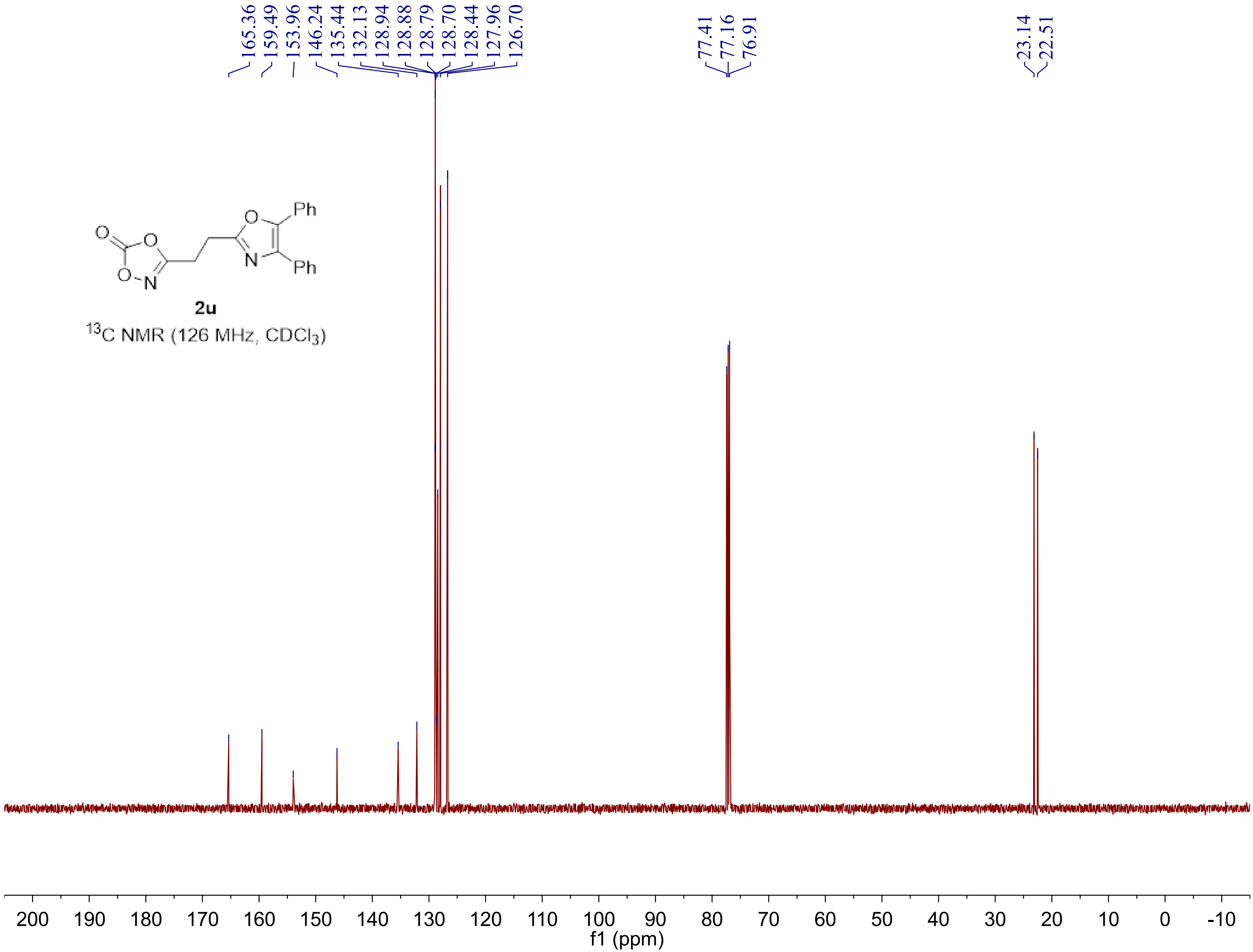

Supplementary Figure 182: ^{13}C NMR (126 MHz, $CDCl_3$) spectrum of **2u**.

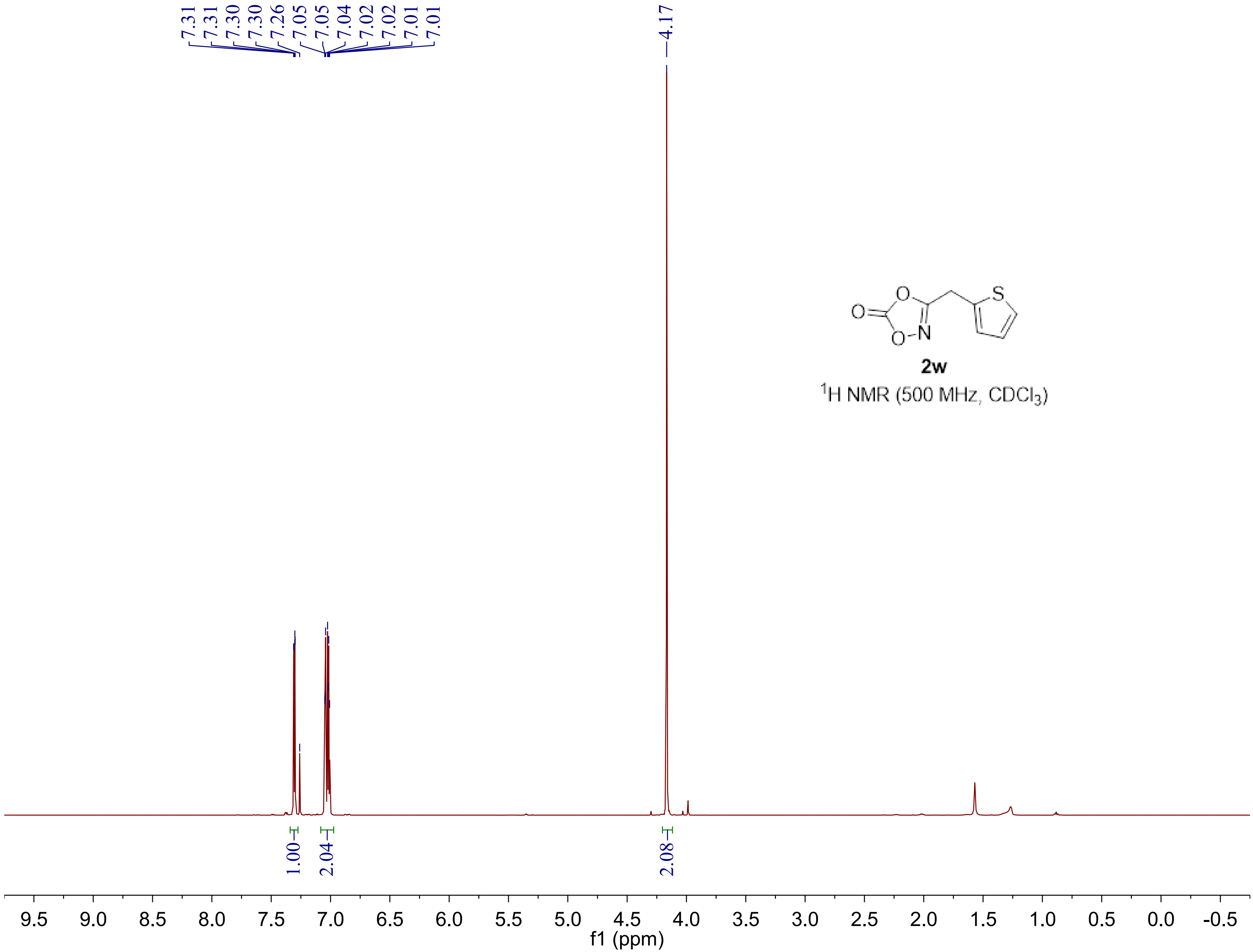

Supplementary Figure 183: ^{1}H NMR (500 MHz, $CDCl_3$) spectrum of **2w**.

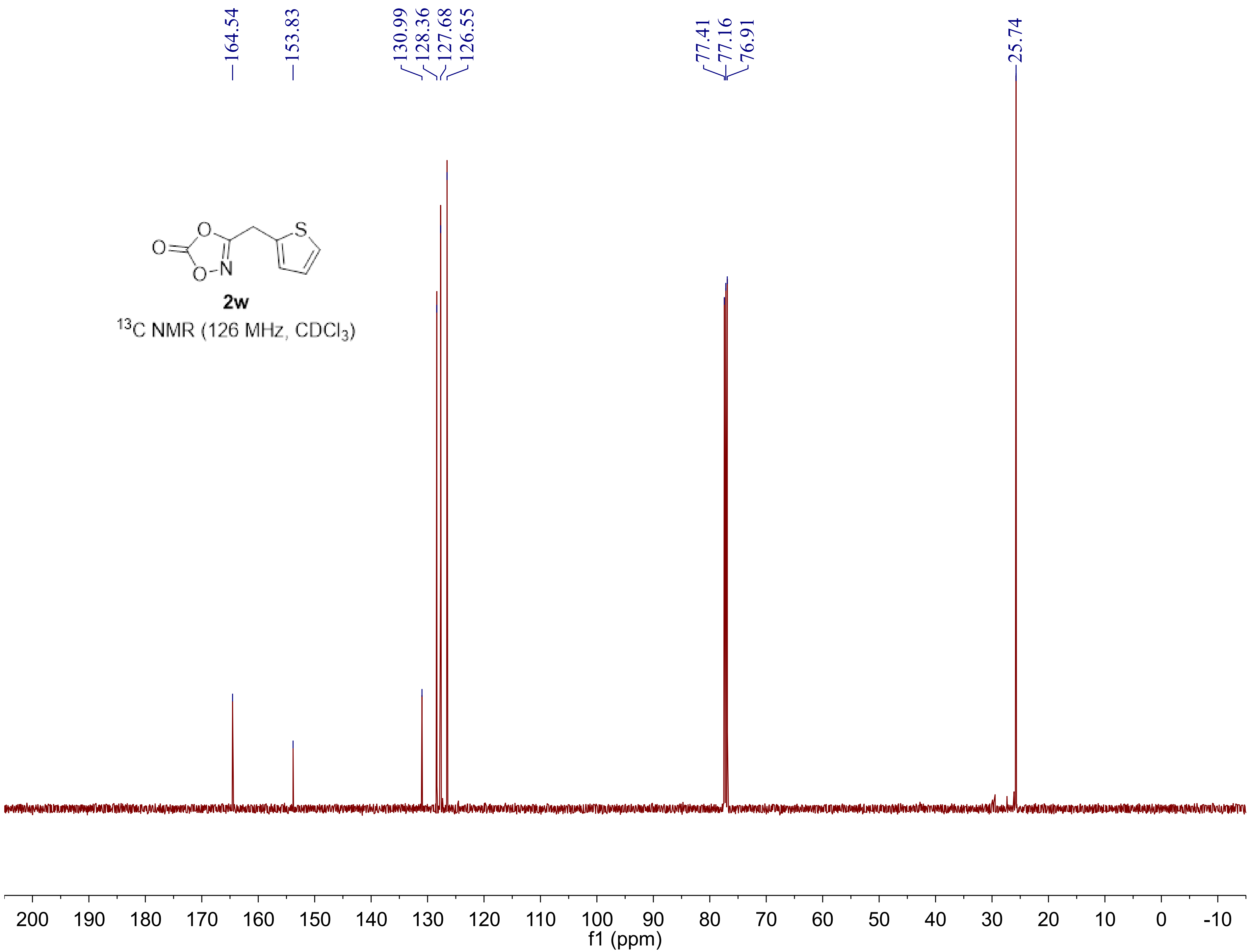

Supplementary Figure 184: ^{13}C NMR (126 MHz, $CDCl_3$) spectrum of **2w**.

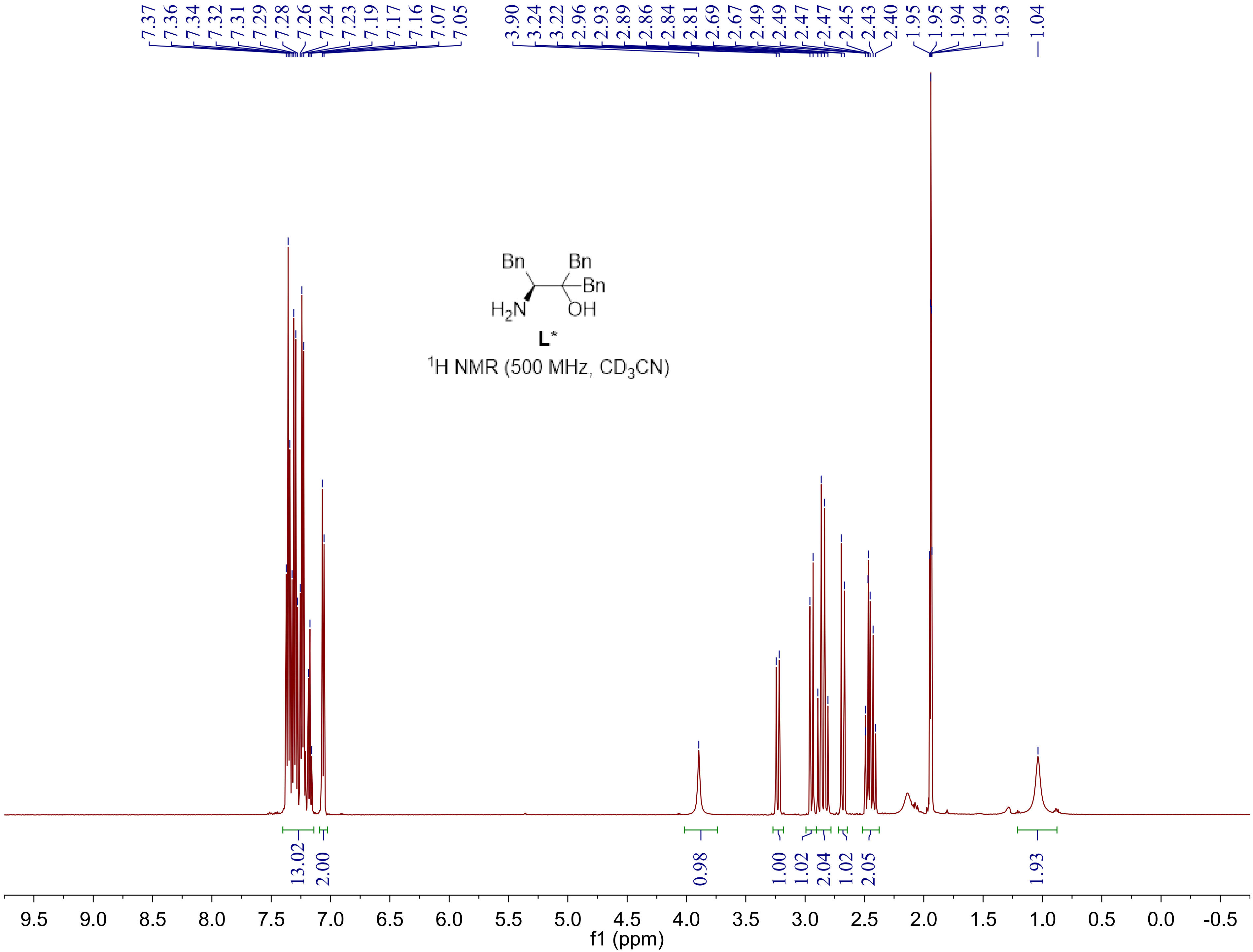

Supplementary Figure 185: ^{1}H NMR (500 MHz, CD_3CN) spectrum of **L***.

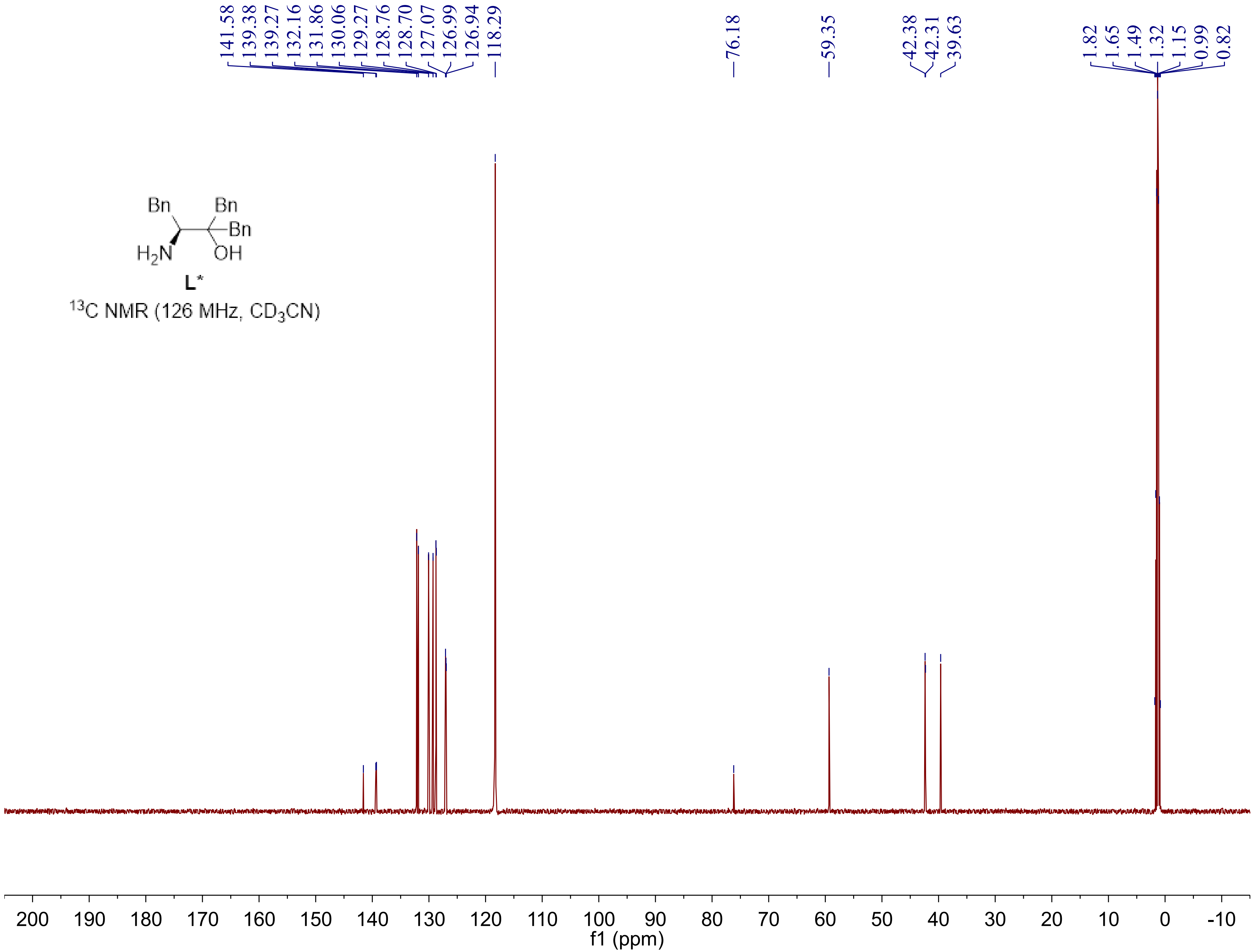

Supplementary Figure 186: ^{13}C NMR (126 MHz, CD_3CN) spectrum of **L***.

2. HPLC Trace

Data File H:\ORIGINAL DATA\ZY-09-146-10-RAC.D
Sample Name: ZY-09-146-10-IE

===
Acq. Operator : 系统 Seq. Line : 3
Acq. Instrument : HPLC-1260 Location : 61
Injection Date : 1/29/2022 5:06:07 PM Inj : 1
Inj Volume : 3.000 µl
Different Inj Volume from Sample Entry! Actual Inj Volume : 0.500 µl
Acq. Method : D:\zy\20220124\YH 2022-01-29 16-32-07\5EtOH10_10-1-6-240.M
Last changed : 1/29/2022 4:32:31 PM by 系统
Analysis Method : E:\DATA\20220317\LC 2022-03-28 16-47-04\3IPA_30_8_2.-SFT.M (Sequence Method)
Last changed : 3/28/2022 10:48:30 PM by SYSTEM
(modified after loading)

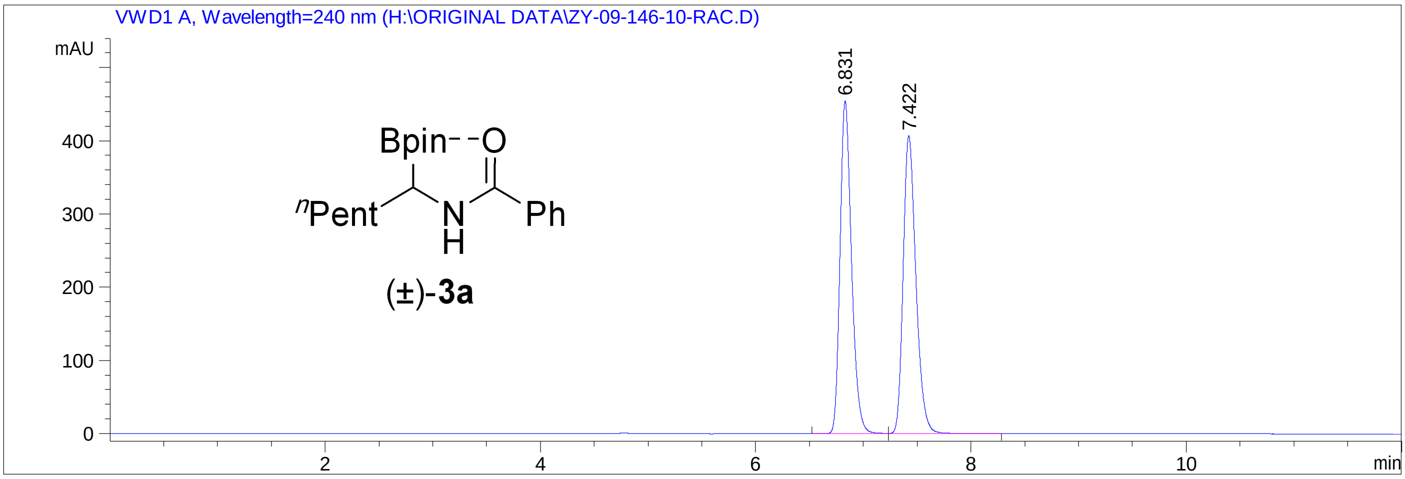

===
Area Percent Report
===

Sorted By : Signal
Multiplier : 1.0000
Dilution : 1.0000
Do not use Multiplier & Dilution Factor with ISTDs

Signal 1: VWD1 A, Wavelength=240 nm

Peak #	RetTime [min]	Type	Width [min]	Area [mAU*s]	Height [mAU]	Area %
1	6.831	VB R	0.1130	3366.24365	454.14883	50.1751
2	7.422	BB	0.1259	3342.75000	406.73416	49.8249

Totals : 6708.99365 860.88300

===
*** End of Report ***

Supplementary Figure 187: HPLC spectrum of (±)-**3a**.

Data File H:\ORIGINAL DATA\ZY-09-146-10-EE.D
Sample Name: ZY-09-146-10-EE

===
Acq. Operator   : 系统   Seq. Line :   2
Acq. Instrument : HPLC-1260   Location :   63
Injection Date : 1/29/2022 9:49:47 PM   Inj :   1
                                                Inj Volume : 3.000 µl
Different Inj Volume from Sample Entry! Actual Inj Volume : 0.300 µl
Acq. Method   : D:\zy\20220124\YH 2022-01-29 21-31-36\5EtOH10_10-1-6-240.M
Last changed   : 1/29/2022 9:31:43 PM by 系统
Analysis Method : E:\DATA\20220317\LC 2022-03-28 16-47-04\3IPA_30_8_2.-SFT.M (Sequence Method
)
Last changed   : 3/28/2022 10:47:29 PM by SYSTEM
                  (modified after loading)

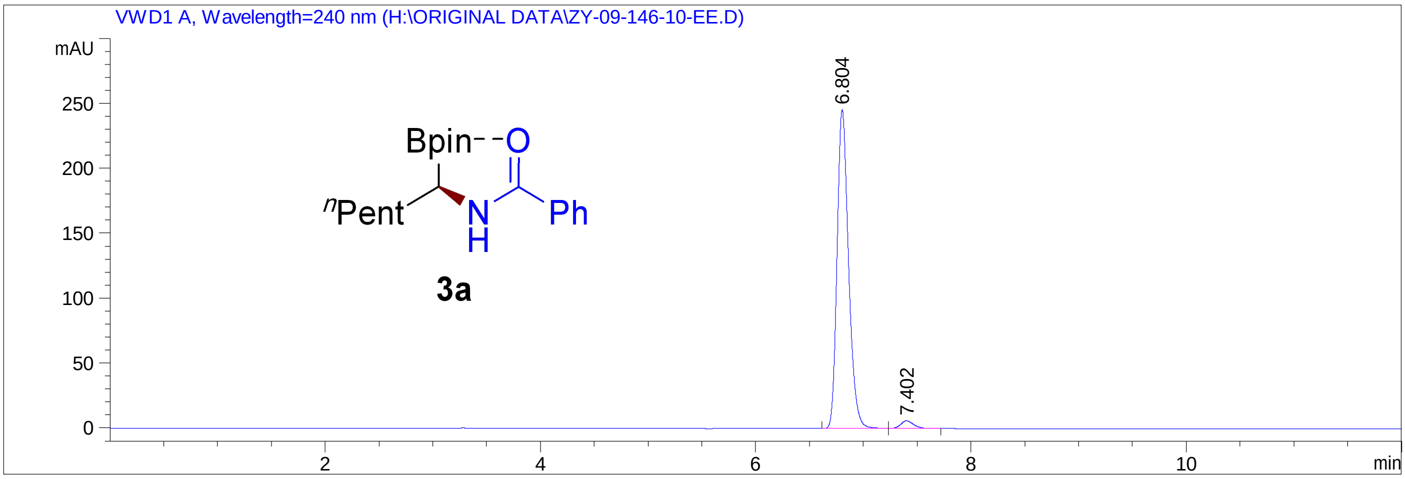

===
Area Percent Report
===

Sorted By   :   Signal
Multiplier   :   1.0000
Dilution   :   1.0000
Do not use Multiplier & Dilution Factor with ISTDs

Signal 1: VWD1 A, Wavelength=240 nm

Peak #	RetTime [min]	Type	Width [min]	Area [mAU*s]	Height [mAU]	Area %
1	6.804	BB	0.1133	1815.76379	245.36716	97.4591
2	7.402	BB	0.1244	47.33982	5.81724	2.5409

Totals :   1863.10361   251.18439

===
*** End of Report ***

Supplementary Figure 188: HPLC spectrum of **3a**.

Data File F:\ZY\AMIDE\ORIGINAL DATA\ZY-09-195-2-EE.D
Sample Name: ZY-09-195-2-EE

===

Acq. Operator : 系统 Seq. Line : 7
Acq. Instrument : HPLC-1260 Location : 84
Injection Date : 2/15/2022 10:02:32 AM Inj : 1
Inj Volume : 3.000 µl
Different Inj Volume from Sample Entry! Actual Inj Volume : 0.500 µl
Acq. Method : D:\zy\20220201\YH 2022-02-15 08-43-37\5EtOH15_10-1-2-240.M
Last changed : 2/15/2022 9:33:50 AM by 系统
Analysis Method : E:\DATA\20220316\LC 2022-03-16 22-19-08\10IPA-10-1.0-1.MZWJ.M (Sequence Method)
Last changed : 3/29/2022 9:51:28 AM by SYSTEM
(modified after loading)
Additional Info : Peak(s) manually integrated

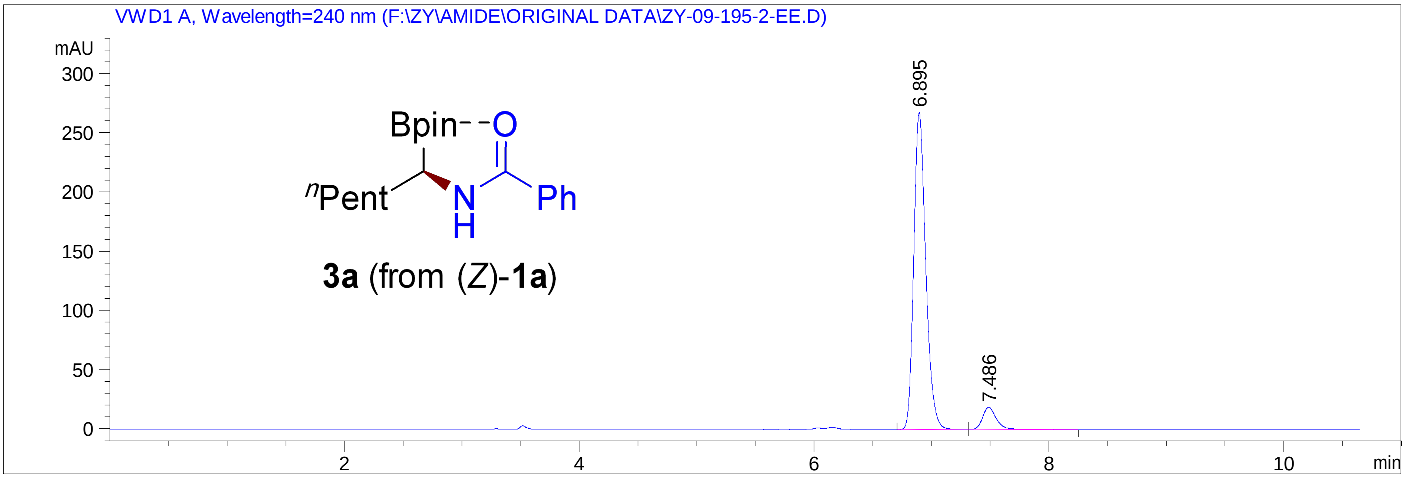

===

Area Percent Report

===

Sorted By : Signal
Multiplier : 1.0000
Dilution : 1.0000
Do not use Multiplier & Dilution Factor with ISTDs

Signal 1: VWD1 A, Wavelength=240 nm

Peak #	RetTime [min]	Type	Width [min]	Area [mAU*s]	Height [mAU]	Area %
1	6.895	BB	0.1081	1892.65186	267.47192	92.4615
2	7.486	BB	0.1246	154.30952	18.72402	7.5385

Totals : 2046.96138 286.19595

===

*** End of Report ***

Supplementary Figure 189: HPLC spectrum of **3a** (from (Z)-**1a**).

Data File E:\DATA\20220317\LC 2022-03-21 09-08-37\1FB-0401--006.D
Sample Name: ZY-10-57-1-RAC

===

Acq. Operator : SYSTEM   Seq. Line : 6
Acq. Instrument : HPLC1260   Location : P1-F2
Injection Date : 3/21/2022 10:24:39 AM   Inj : 1
Inj Volume : 3.000 µl
Different Inj Volume from Sample Entry! Actual Inj Volume : 0.300 µl
Acq. Method : E:\DATA\20220317\LC 2022-03-21 09-08-37\8IPA_15_0.5_1-254.M
Last changed : 3/21/2022 9:08:37 AM by SYSTEM
Analysis Method : E:\DATA\20220317\LC 2022-03-21 09-08-37\8IPA_15_0.5_1-254.M (Sequence Method)
Last changed : 3/21/2022 12:17:47 PM by SYSTEM
(modified after loading)
Additional Info : Peak(s) manually integrated

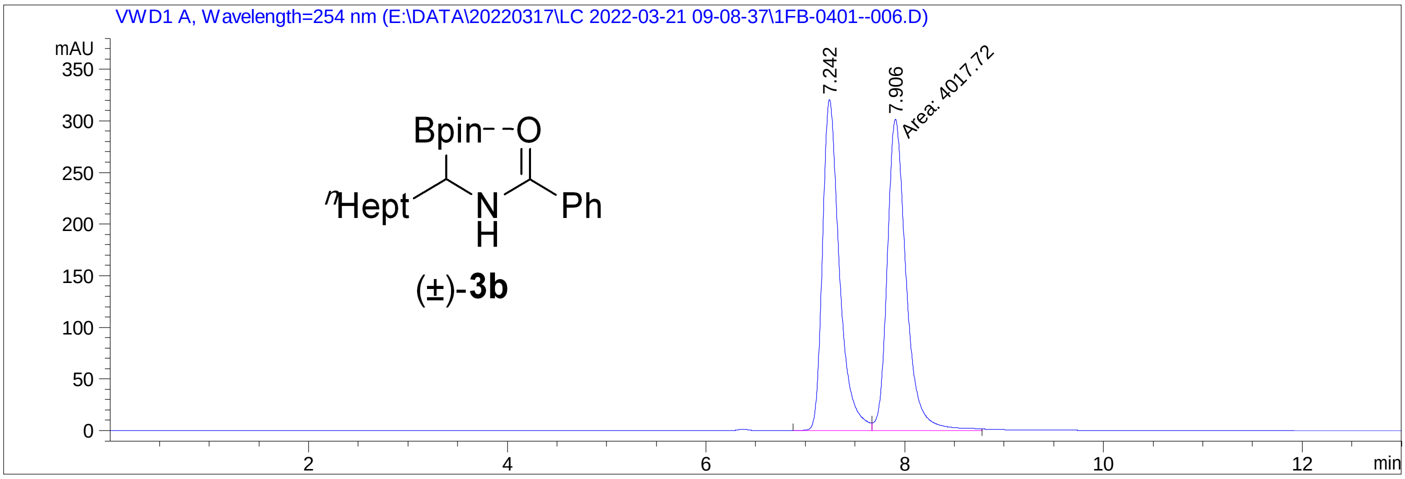

===

Area Percent Report

===

Sorted By : Signal
Multiplier : 1.0000
Dilution : 1.0000
Do not use Multiplier & Dilution Factor with ISTDs

Signal 1: VWD1 A, Wavelength=254 nm

Peak #	RetTime [min]	Type	Width [min]	Area [mAU*s]	Height [mAU]	Area %
1	7.242	BV	0.1790	3818.48267	320.39621	48.7287
2	7.906	MF	0.2222	4017.72388	301.33694	51.2713

Totals : 7836.20654 621.73315

===

*** End of Report ***

Supplementary Figure 190: HPLC spectrum of (±)-**3b**.

Data File E:\DATA\20220317\LC 2022-03-21 09-08-37\OnlineEdited--003.D
Sample Name: ZY-09-168-1-EE

===
Acq. Operator   : SYSTEM   Seq. Line :   3
Acq. Instrument : HPLC1260   Location :   P1-F3
Injection Date : 3/21/2022 9:37:16 AM   Inj :   1
                                                 Inj Volume : 3.000 µl
Different Inj Volume from Sample Entry! Actual Inj Volume : 0.500 µl
Acq. Method   : E:\DATA\20220317\LC 2022-03-21 09-08-37\8IPA_15_0.5_1-254.M
Last changed   : 3/21/2022 9:08:37 AM by SYSTEM
Analysis Method : E:\DATA\20220317\LC 2022-03-21 09-08-37\8IPA_15_0.5_1-254.M (Sequence Method)
Last changed   : 3/21/2022 12:17:47 PM by SYSTEM
(modified after loading)
Additional Info : Peak(s) manually integrated

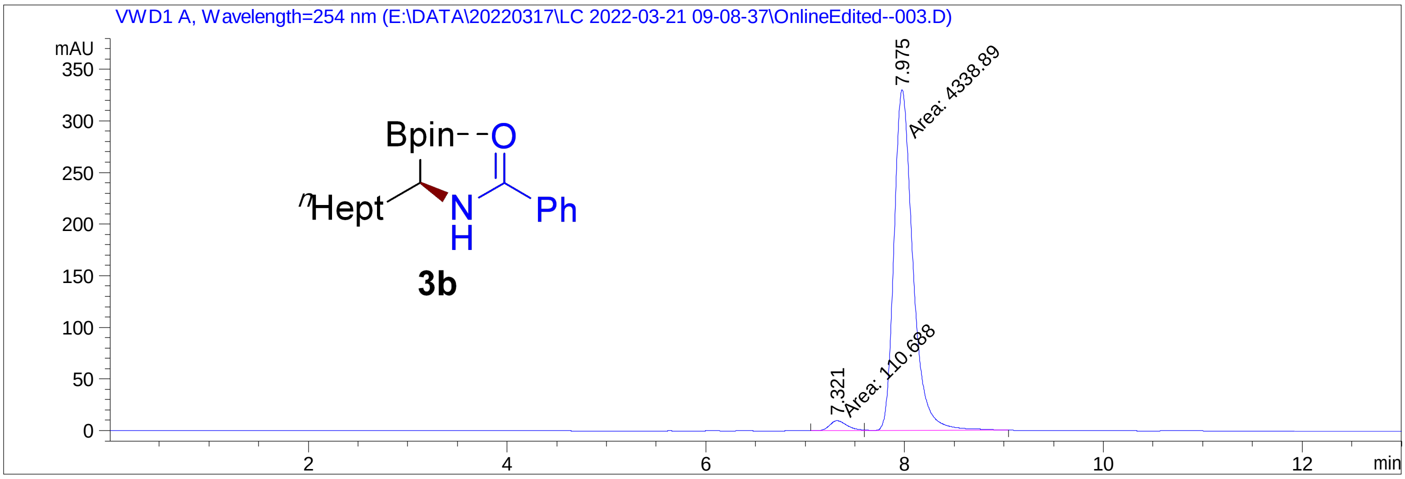

===
Area Percent Report
===

Sorted By   :   Signal
Multiplier   :   1.0000
Dilution   :   1.0000
Do not use Multiplier & Dilution Factor with ISTDs

Signal 1: VWD1 A, Wavelength=254 nm

Peak #	RetTime [min]	Type	Width [min]	Area [mAU*s]	Height [mAU]	Area %
1	7.321	MF	0.1973	110.68835	9.35163	2.4876
2	7.975	FM	0.2193	4338.88623	329.76462	97.5124

Totals :   4449.57458 339.11625

===
*** End of Report ***

Supplementary Figure 191: HPLC spectrum of **3b**.

Data File E:\DATA\20220317\LC 2022-03-21 09-08-37\1FA-0301--004.D
Sample Name: ZY-10-57-1-EE

===
Acq. Operator : SYSTEM Seq. Line : 4
Acq. Instrument : HPLC1260 Location : P1-F1
Injection Date : 3/21/2022 9:53:03 AM Inj : 1
Inj Volume : 3.000 µl
Different Inj Volume from Sample Entry! Actual Inj Volume : 2.000 µl
Acq. Method : E:\DATA\20220317\LC 2022-03-21 09-08-37\8IPA_15_0.5_1-254.M
Last changed : 3/21/2022 9:08:37 AM by SYSTEM
Analysis Method : E:\DATA\20220317\LC 2022-03-21 09-08-37\8IPA_15_0.5_1-254.M (Sequence Method)
Last changed : 3/21/2022 12:17:47 PM by SYSTEM
(modified after loading)

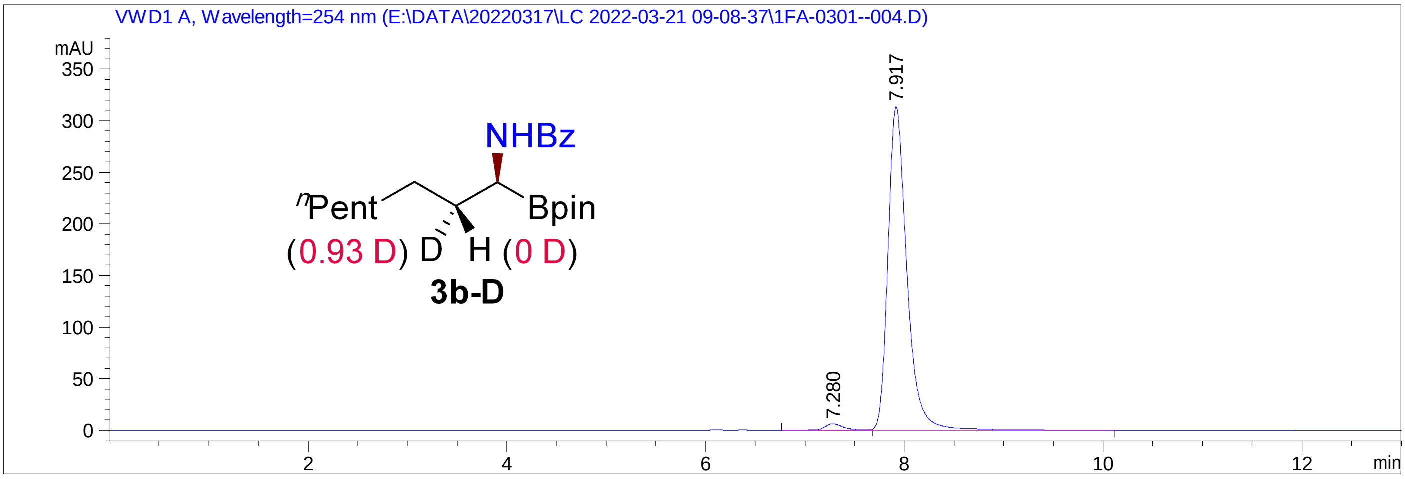

===
Area Percent Report
===

Sorted By : Signal
Multiplier : 1.0000
Dilution : 1.0000
Do not use Multiplier & Dilution Factor with ISTDs

Signal 1: VWD1 A, Wavelength=254 nm

Peak #	RetTime [min]	Type	Width [min]	Area [mAU*s]	Height [mAU]	Area %
1	7.280	BV E	0.1991	85.09237	6.39980	1.9985
2	7.917	VB R	0.2023	4172.81006	313.51718	98.0015

Totals : 4257.90243 319.91699

===
*** End of Report ***

Supplementary Figure 192: HPLC spectrum of **3b-D**.

Data File F:\ZY\AMIDE\ORIGINAL DATA\ZY-09-187-2-RAC.D
Sample Name: ZY-09-187-2-RAC

===

Acq. Operator : 系统 Seq. Line : 5
Acq. Instrument : HPLC-1260 Location : 44
Injection Date : 12/23/2021 10:10:24 AM Inj : 1
Inj Volume : 3.000 µl
Different Inj Volume from Sample Entry! Actual Inj Volume : 0.200 µl
Acq. Method : D:\zy\20211219\YH 2021-12-23 09-03-54\10EtOH-15-0.8-1-6-240.M
Last changed : 12/23/2021 9:03:56 AM by 系统
Analysis Method : E:\DATA\20220316\LC 2022-03-16 22-19-08\0.6IPA-15-0.8-1-210-JXL.M (Sequence Method)
Last changed : 3/29/2022 9:27:30 AM by SYSTEM
(modified after loading)
Additional Info : Peak(s) manually integrated

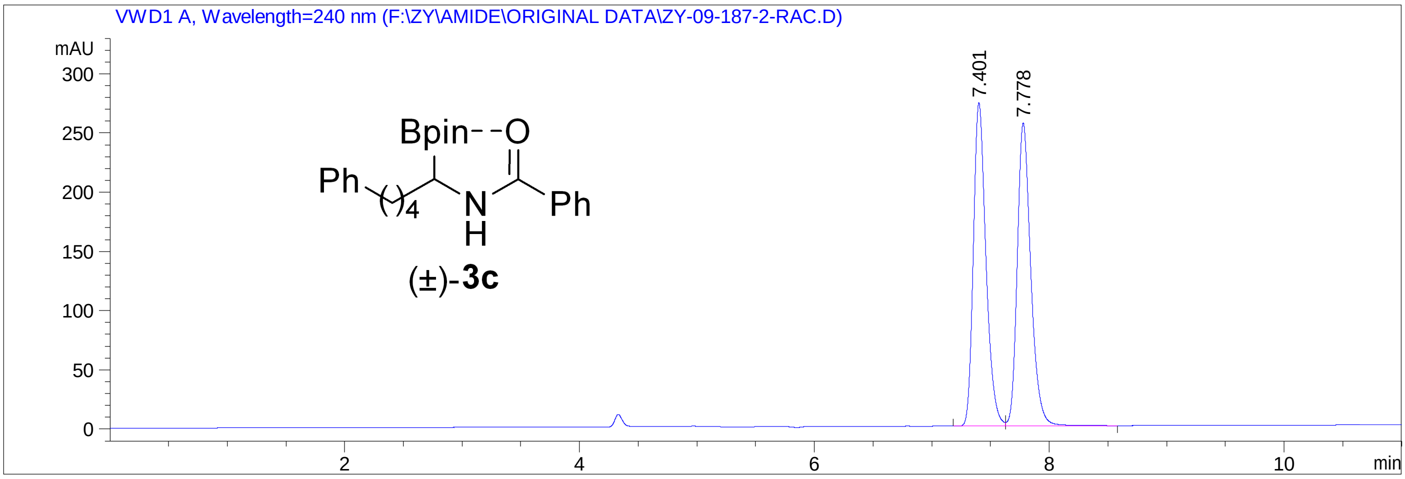

===

Area Percent Report

===

Sorted By : Signal
Multiplier : 1.0000
Dilution : 1.0000
Do not use Multiplier & Dilution Factor with ISTDs

Signal 1: VWD1 A, Wavelength=240 nm

Peak #	RetTime [min]	Type	Width [min]	Area [mAU*s]	Height [mAU]	Area %
1	7.401	BV	0.1128	2031.66479	273.12259	49.7983
2	7.778	VB	0.1209	2048.12427	255.81441	50.2017

Totals : 4079.78906 528.93700

===

*** End of Report ***

Supplementary Figure 193: HPLC spectrum of (±)-**3c**.

Data File F:\ZY\AMIDE\ORIGINAL DATA\ZY-09-187-2-EE.D
Sample Name: ZY-09-187-2-EE

===

Acq. Operator : 系统 Seq. Line : 4
Acq. Instrument : HPLC-1260 Location : 43
Injection Date : 12/23/2021 9:54:02 AM Inj : 1
Inj Volume : 3.000 µl
Different Inj Volume from Sample Entry! Actual Inj Volume : 0.400 µl
Acq. Method : D:\zy\20211219\YH 2021-12-23 09-03-54\10EtOH-15-0.8-1-6-240.M
Last changed : 12/23/2021 9:03:56 AM by 系统
Analysis Method : E:\DATA\20220316\LC 2022-03-16 22-19-08\0.6IPA-15-0.8-1-210-JXL.M (Sequence Method)
Last changed : 3/29/2022 9:27:03 AM by SYSTEM
(modified after loading)

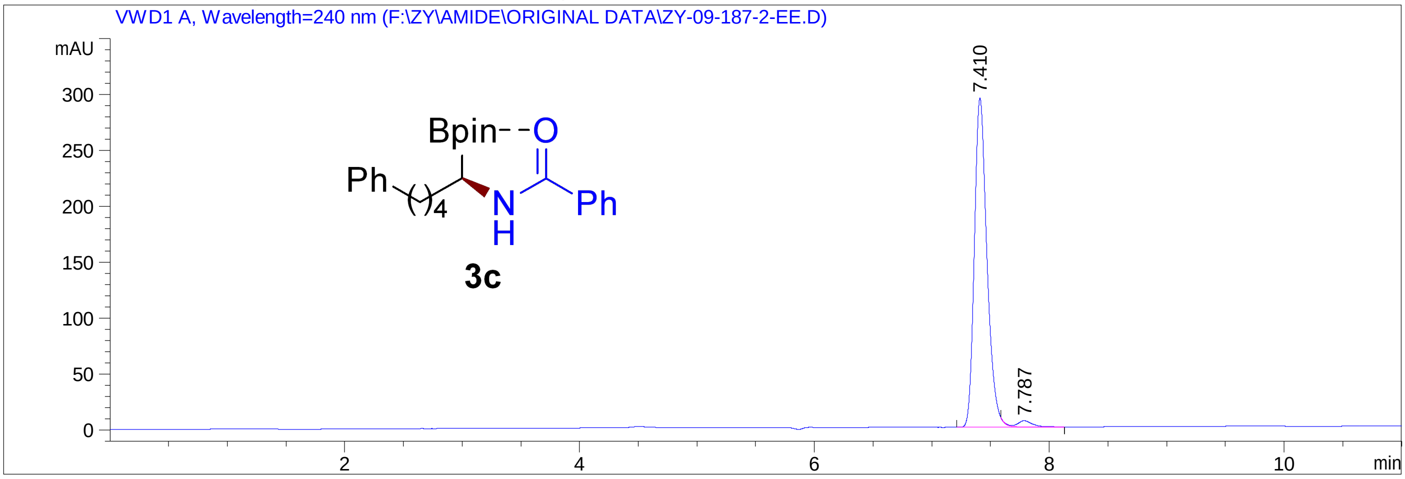

===

Area Percent Report

===

Sorted By : Signal
Multiplier : 1.0000
Dilution : 1.0000
Do not use Multiplier & Dilution Factor with ISTDs

Signal 1: VWD1 A, Wavelength=240 nm

Peak #	RetTime [min]	Type	Width [min]	Area [mAU*s]	Height [mAU]	Area %
1	7.410	BV R	0.1140	2207.72168	294.35645	97.8049
2	7.787	VB E	0.1295	49.54852	5.61053	2.1951

Totals : 2257.27020 299.96697

===

*** End of Report ***

Supplementary Figure 194: HPLC spectrum of **3c**.

Data File F:\ZY\AMIDE\ORIGINAL DATA\ZY-09-192-5-RAC.D
Sample Name: ZY-09-192-7-RAC

===
Acq. Operator : 系统 Seq. Line : 4
Acq. Instrument : HPLC-1260 Location : 82
Injection Date : 3/1/2022 2:24:02 PM Inj : 1
Inj Volume : 3.000 µl
Different Inj Volume from Sample Entry! Actual Inj Volume : 0.600 µl
Acq. Method : D:\zy\20220224\YH 2022-03-01 13-37-55\5IPA15_10-1-6-240.M
Last changed : 3/1/2022 1:37:55 PM by 系统
Analysis Method : E:\DATA\20220316\LC 2022-03-16 22-19-08\10IPA-10-1.0-1.MZWJ.M (Sequence Method)
Last changed : 3/29/2022 9:42:28 AM by SYSTEM
(modified after loading)

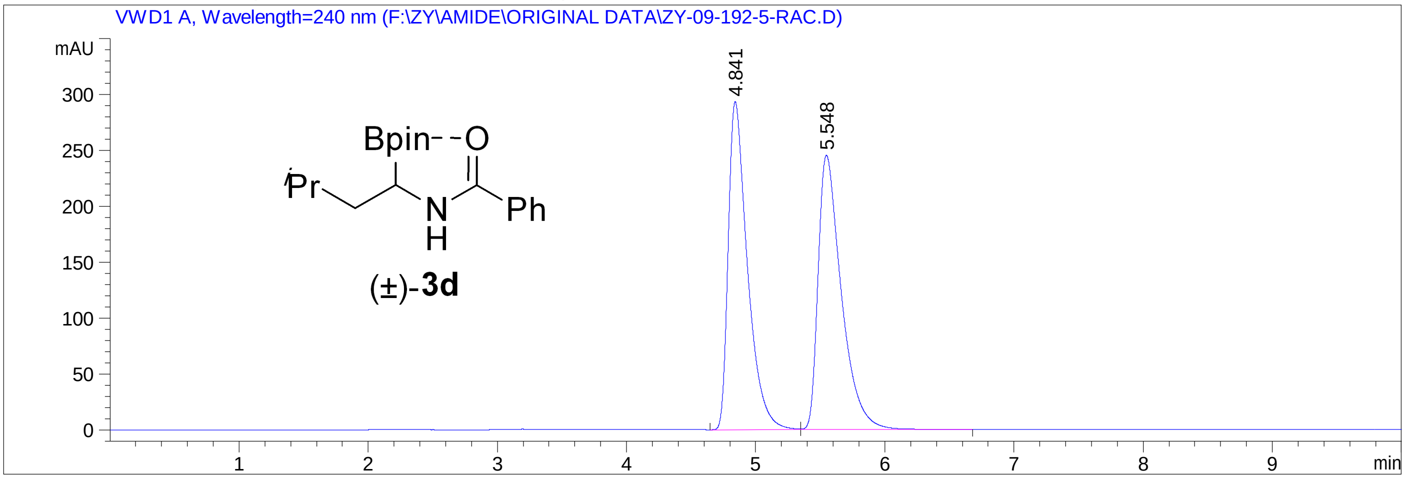

===
Area Percent Report
===

Sorted By : Signal
Multiplier : 1.0000
Dilution : 1.0000
Do not use Multiplier & Dilution Factor with ISTDs

Signal 1: VWD1 A, Wavelength=240 nm

Peak #	RetTime [min]	Type	Width [min]	Area [mAU*s]	Height [mAU]	Area %
1	4.841	BV	0.1531	3033.27930	293.32916	49.7162
2	5.548	VB	0.1874	3067.90430	245.37120	50.2838

Totals : 6101.18359 538.70036

===
*** End of Report ***

Supplementary Figure 195: HPLC spectrum of (±)-**3d**.

Data File F:\ZY\AMIDE\ORIGINAL DATA\ZY-09-192-5-EE.D
Sample Name: ZY-09-192-5-EE

===
Acq. Operator : 系统 Seq. Line : 4
Acq. Instrument : HPLC-1260 Location : 81
Injection Date : 3/1/2022 5:01:13 PM Inj : 1
Inj Volume : 3.000 µl
Different Inj Volume from Sample Entry! Actual Inj Volume : 1.200 µl
Acq. Method : D:\zy\20220224\YH 2022-03-01 16-20-30\5IPA15_10-1-6-240.M
Last changed : 3/1/2022 4:20:31 PM by 系统
Analysis Method : E:\DATA\20220316\LC 2022-03-16 22-19-08\10IPA-10-1.0-1.MZWJ.M (Sequence Method)
Last changed : 3/29/2022 9:41:21 AM by SYSTEM
(modified after loading)

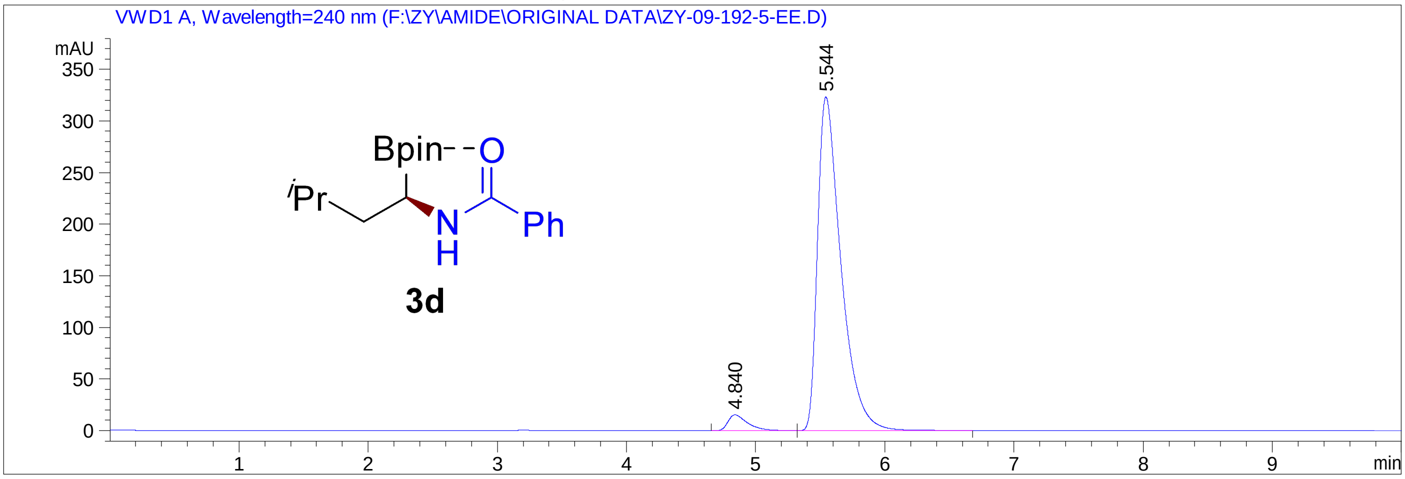

===
Area Percent Report
===

Sorted By : Signal
Multiplier : 1.0000
Dilution : 1.0000
Do not use Multiplier & Dilution Factor with ISTDs

Signal 1: VWD1 A, Wavelength=240 nm

Peak #	RetTime [min]	Type	Width [min]	Area [mAU*s]	Height [mAU]	Area %
1	4.840	BB	0.1574	163.37755	15.37929	3.8617
2	5.544	BB	0.1893	4067.31079	323.30203	96.1383

Totals : 4230.68834 338.68133

===
*** End of Report ***

Supplementary Figure 196: HPLC spectrum of **3d**.

Data File F:\ZY\AMIDE\ORIGINAL DATA\ZY-10-43-EE.D
Sample Name: ZY-10-43-EE

===

Acq. Operator : 系统 Seq. Line : 3
Acq. Instrument : HPLC-1260 Location : 81
Injection Date : 3/1/2022 2:07:38 PM Inj : 1
Inj Volume : 3.000 µl
Different Inj Volume from Sample Entry! Actual Inj Volume : 0.600 µl
Acq. Method : D:\zy\20220224\YH 2022-03-01 13-37-55\5IPA15_10-1-6-240.M
Last changed : 3/1/2022 1:37:55 PM by 系统
Analysis Method : C:\CHEM32\1\METHODS\0.2IPA-10-0.5-1-XYH-EQ.M
Last changed : 3/29/2022 10:09:24 AM by SYSTEM
(modified after loading)

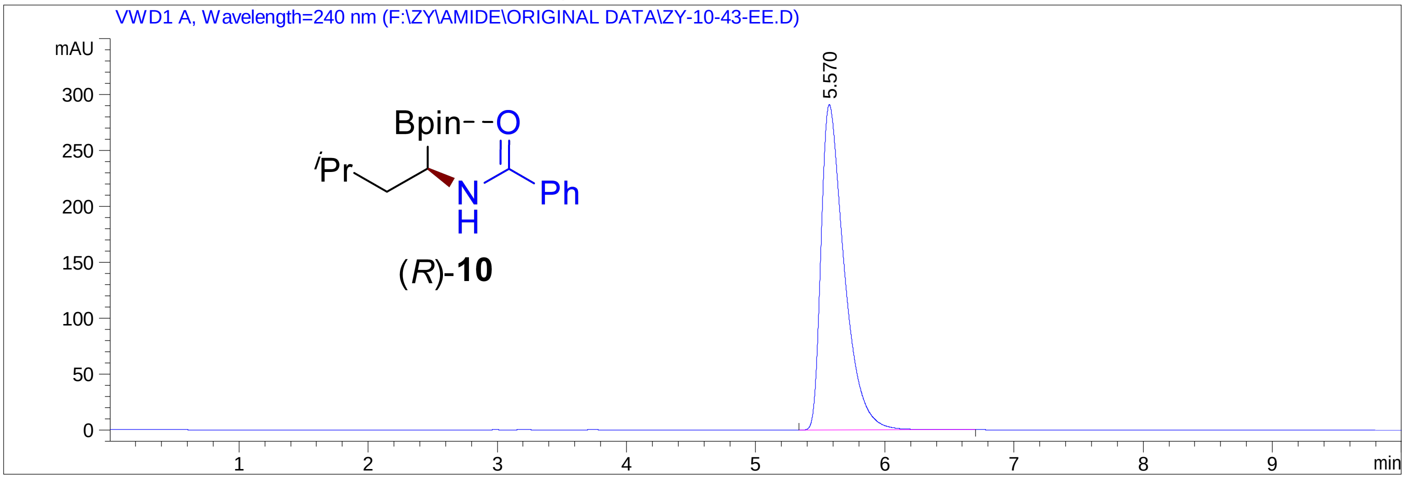

===

Area Percent Report

===

Sorted By : Signal
Multiplier : 1.0000
Dilution : 1.0000
Do not use Multiplier & Dilution Factor with ISTDs

Signal 1: VWD1 A, Wavelength=240 nm

Peak #	RetTime [min]	Type	Width [min]	Area [mAU*s]	Height [mAU]	Area %
1	5.570	BB	0.1852	3632.72119	290.84302	100.0000
Totals :				3632.72119	290.84302	

===

*** End of Report ***

Supplementary Figure 197: HPLC spectrum of (*R*)-**10**.

Data File F:\ZY\AMIDE\ORIGINAL DATA\ZY-09-186-2-RAC.D
Sample Name: ZY-09-186-2-RAC

===

Acq. Operator : 系统 Seq. Line : 10
Acq. Instrument : HPLC-1260 Location : 11
Injection Date : 12/15/2021 11:47:34 AM Inj : 1
Inj Volume : 3.000 µl
Different Inj Volume from Sample Entry! Actual Inj Volume : 0.800 µl
Acq. Method : D:\zy\20211129\YH 2021-12-15 09-26-25\5IPA-15-1.0-1-6-254-ZH.M
Last changed : 12/15/2021 11:14:11 AM by 系统
Analysis Method : E:\DATA\20220316\LC 2022-03-16 22-19-08\0.6IPA-15-0.8-1-210-JXL.M (Sequence Method)
Last changed : 3/29/2022 9:25:16 AM by SYSTEM
(modified after loading)
Additional Info : Peak(s) manually integrated

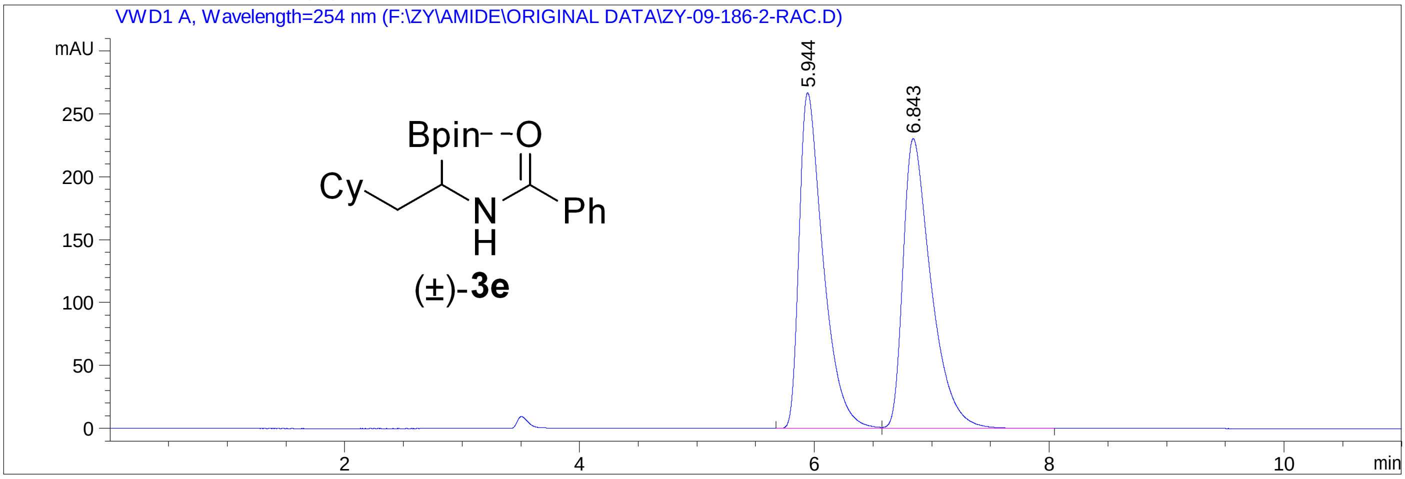

===

Area Percent Report

===

Sorted By : Signal
Multiplier : 1.0000
Dilution : 1.0000
Do not use Multiplier & Dilution Factor with ISTDs

Signal 1: VWD1 A, Wavelength=254 nm

Peak #	RetTime [min]	Type	Width [min]	Area [mAU*s]	Height [mAU]	Area %
1	5.944	BV	0.2142	3801.18140	266.80502	49.7416
2	6.843	VB	0.2460	3840.67212	230.42453	50.2584

Totals : 7641.85352 497.22955

===

*** End of Report ***

Supplementary Figure 198: HPLC spectrum of (±)-**3e**.

Data File F:\ZY\AMIDE\ORIGINAL DATA\ZY-09-186-2-EE.D
Sample Name: ZY-09-186-2-EE

===

Acq. Operator : 系统 Seq. Line : 9
Acq. Instrument : HPLC-1260 Location : 13
Injection Date : 12/15/2021 11:31:08 AM Inj : 1
Inj Volume : 3.000 µl
Different Inj Volume from Sample Entry! Actual Inj Volume : 1.200 µl
Acq. Method : D:\zy\20211129\YH 2021-12-15 09-26-25\5IPA-15-1.0-1-6-254-ZH.M
Last changed : 12/15/2021 11:14:11 AM by 系统
Analysis Method : E:\DATA\20220316\LC 2022-03-16 22-19-08\0.6IPA-15-0.8-1-210-JXL.M (Sequence Method)
Last changed : 3/29/2022 9:24:40 AM by SYSTEM
(modified after loading)
Additional Info : Peak(s) manually integrated

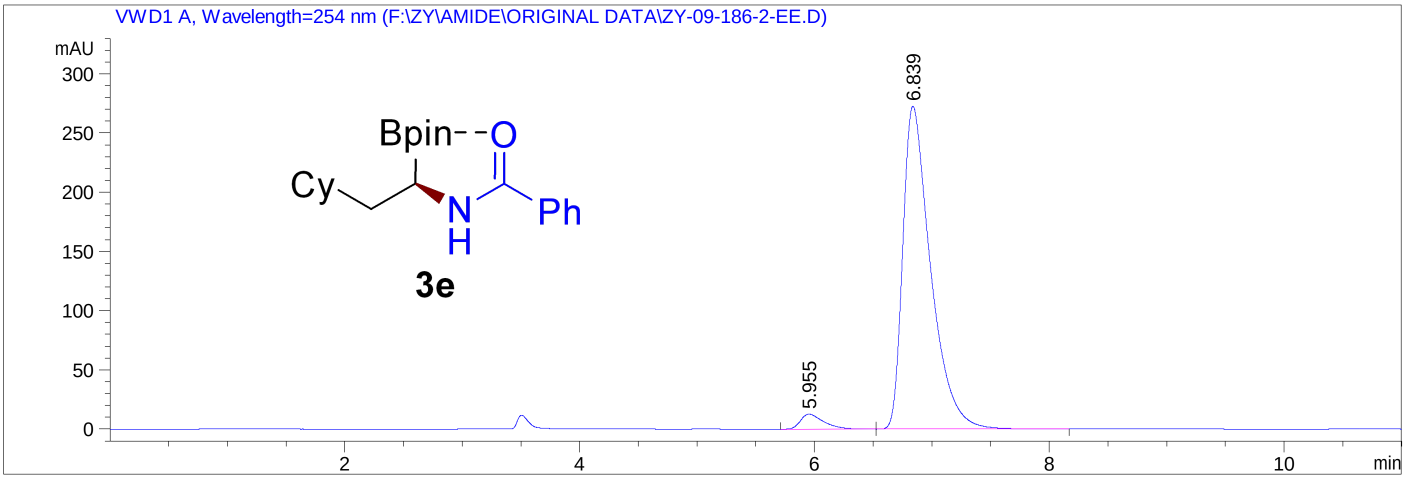

===

Area Percent Report

===

Sorted By : Signal
Multiplier : 1.0000
Dilution : 1.0000
Do not use Multiplier & Dilution Factor with ISTDs

Signal 1: VWD1 A, Wavelength=254 nm

Peak #	RetTime [min]	Type	Width [min]	Area [mAU*s]	Height [mAU]	Area %
1	5.955	BB	0.2016	174.05440	12.64594	3.7263
2	6.839	BB	0.2464	4496.94629	272.66776	96.2737

Totals : 4671.00069 285.31369

===

*** End of Report ***

Supplementary Figure 199: HPLC spectrum of **3e**.

Data File H:\DATA2\ZY-10-64-2-RAC.D
Sample Name: ZY-10-64-3-RAC

===

Acq. Operator : 系统 Seq. Line : 7
Acq. Instrument : HPLC-1260 Location : 41
Injection Date : 5/24/2022 7:49:56 PM Inj : 1
Inj Volume : 3.000 µl
Different Inj Volume from Sample Entry! Actual Inj Volume : 0.200 µl
Acq. Method : D:\zy\20220524\YH 2022-05-24 18-17-05\5EtOH15_10-1-2-240.M
Last changed : 5/24/2022 7:13:28 PM by 系统
Analysis Method : E:\DATA\20220317\LC 2022-06-04 08-49-06\0.3IPA-0.3-60-220-1-CCP.M (Sequence Method)
Last changed : 6/7/2022 10:11:57 AM by SYSTEM
(modified after loading)
Additional Info : Peak(s) manually integrated

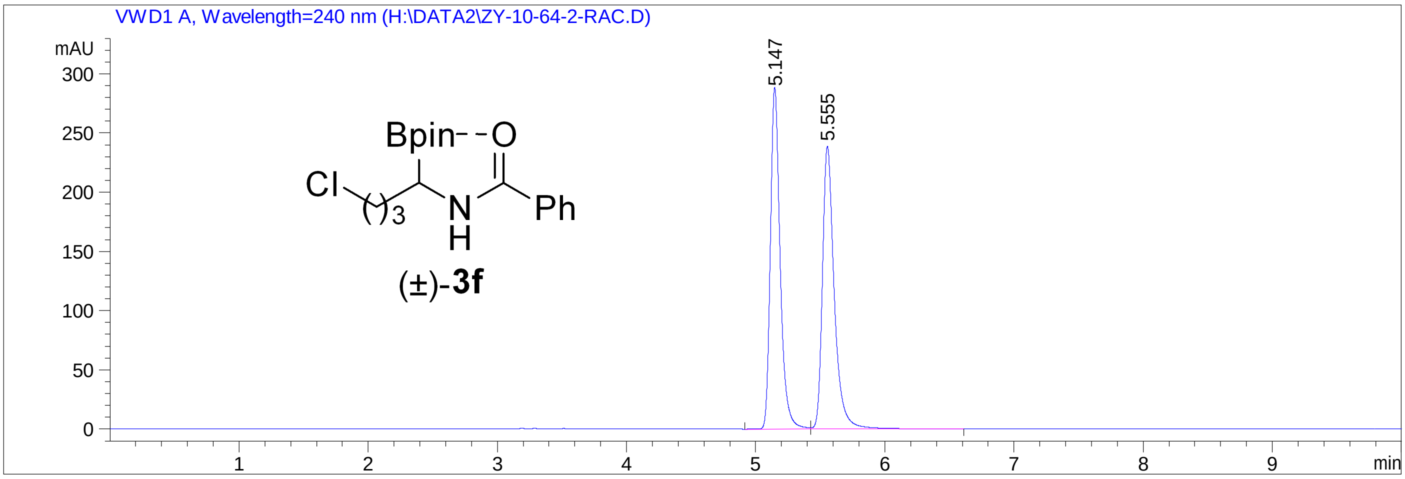

===

Area Percent Report

===

Sorted By : Signal
Multiplier : 1.0000
Dilution : 1.0000
Do not use Multiplier & Dilution Factor with ISTDs

Signal 1: VWD1 A, Wavelength=240 nm

Peak #	RetTime [min]	Type	Width [min]	Area [mAU*s]	Height [mAU]	Area %
1	5.147	VV R	0.0792	1514.91614	288.64532	49.8930
2	5.555	VB	0.0954	1521.41443	238.66467	50.1070

Totals : 3036.33057 527.31000

===

*** End of Report ***

Supplementary Figure 200: HPLC spectrum of (±)-**3f**.

Data File H:\DATA2\ZY-10-64-2-EE.D
Sample Name: ZY-10-64-2-EE

===

Acq. Operator : 系统 Seq. Line : 5
Acq. Instrument : HPLC-1260 Location : 42
Injection Date : 5/24/2022 7:15:09 PM Inj : 2
Inj Volume : 3.000 µl
Different Inj Volume from Sample Entry! Actual Inj Volume : 0.300 µl
Acq. Method : D:\zy\20220524\YH 2022-05-24 18-17-05\5EtOH15_10-1-2-240.M
Last changed : 5/24/2022 7:13:28 PM by 系统
Analysis Method : E:\DATA\20220317\LC 2022-06-04 08-49-06\0.3IPA-0.3-60-220-1-CCP.M (Sequence Method)
Last changed : 6/7/2022 10:10:23 AM by SYSTEM
(modified after loading)
Additional Info : Peak(s) manually integrated

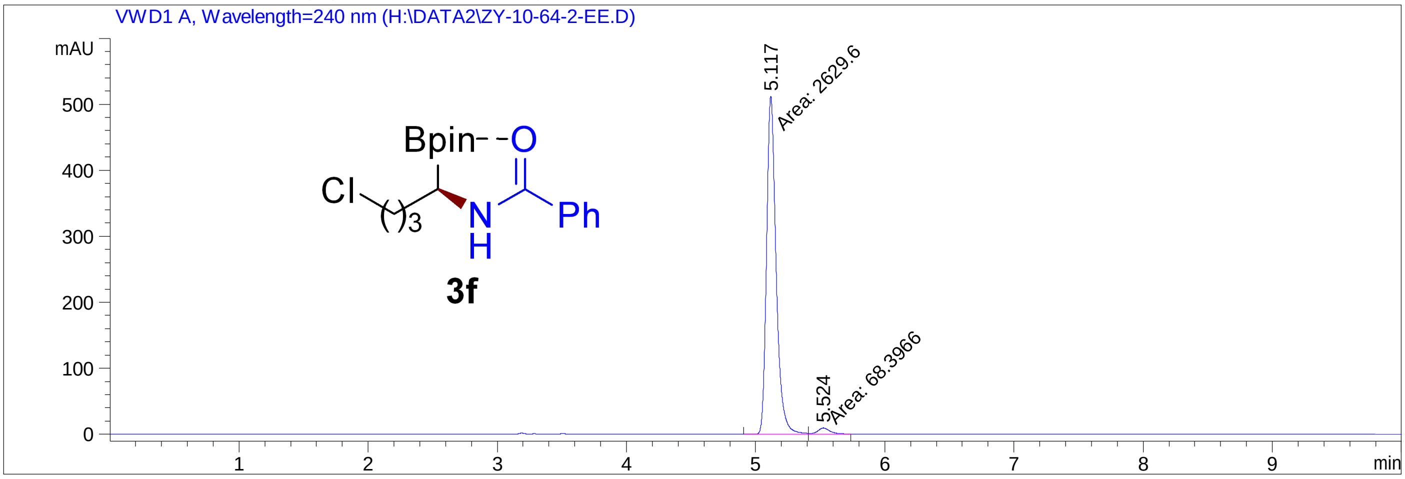

===

Area Percent Report

===

Sorted By : Signal
Multiplier : 1.0000
Dilution : 1.0000
Do not use Multiplier & Dilution Factor with ISTDs

Signal 1: VWD1 A, Wavelength=240 nm

Peak #	RetTime [min]	Type	Width [min]	Area [mAU*s]	Height [mAU]	Area %
1	5.117	MF	0.0856	2629.60010	512.06506	97.4649
2	5.524	MF	0.1180	68.39656	9.66246	2.5351

Totals : 2697.99666 521.72753

===

*** End of Report ***

Supplementary Figure 201: HPLC spectrum of **3f.**

Data File H:\1\ZY-09-186-6-RAC.D
Sample Name: ZY-09-186-6-RAC

===

Acq. Operator : 系统 Seq. Line : 32
Acq. Instrument : HPLC-1260 Location : 78
Injection Date : 8/20/2022 5:00:51 PM Inj : 1
Inj Volume : 3.000 µl
Different Inj Volume from Sample Entry! Actual Inj Volume : 1.600 µl
Acq. Method : D:\zy\20220803\YH 2022-08-20 08-41-06\5IPA20_10-1-2-240.M
Last changed : 8/20/2022 1:41:14 PM by 系统
Analysis Method : E:\DATA\20220317\LC 2022-08-22 08-37-45\5IPA-40-1.0-1-220-QDY.M (Sequence Method)
Last changed : 8/22/2022 7:02:33 PM by SYSTEM
(modified after loading)
Additional Info : Peak(s) manually integrated

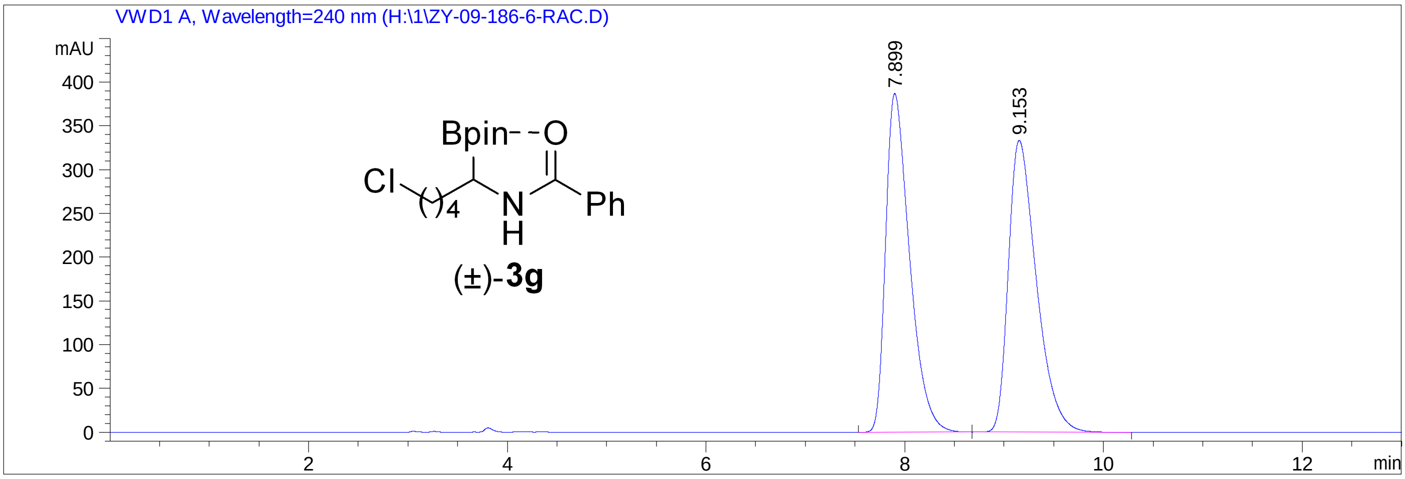

===

Area Percent Report

===

Sorted By : Signal
Multiplier : 1.0000
Dilution : 1.0000
Do not use Multiplier & Dilution Factor with ISTDs

Signal 1: VWD1 A, Wavelength=240 nm

Peak #	RetTime [min]	Type	Width [min]	Area [mAU*s]	Height [mAU]	Area %
1	7.899	BB	0.2586	6487.87744	387.00372	49.8193
2	9.153	BB	0.2952	6534.93945	333.38983	50.1807

Totals : 1.30228e4 720.39355

===

*** End of Report ***

Supplementary Figure 202: HPLC spectrum of (±)-**3g**.

Data File H:\1\ZY-09-186-6-EE.D
Sample Name: ZY-09-186-6-EE

===

Acq. Operator   : 系统   Seq. Line : 30
Acq. Instrument : HPLC-1260   Location :   80
Injection Date : 8/20/2022 4:28:28 PM   Inj :   1
                                               Inj Volume : 3.000 µl
Different Inj Volume from Sample Entry! Actual Inj Volume : 1.500 µl
Acq. Method   : D:\zy\20220803\YH 2022-08-20 08-41-06\5IPA20_10-1-2-240.M
Last changed   : 8/20/2022 1:41:14 PM by 系统
Analysis Method : E:\DATA\20220317\LC 2022-08-22 08-37-45\5IPA-40-1.0-1-220-QDY.M (Sequence Method)
Last changed   : 8/22/2022 7:01:09 PM by SYSTEM
                  (modified after loading)
Additional Info : Peak(s) manually integrated

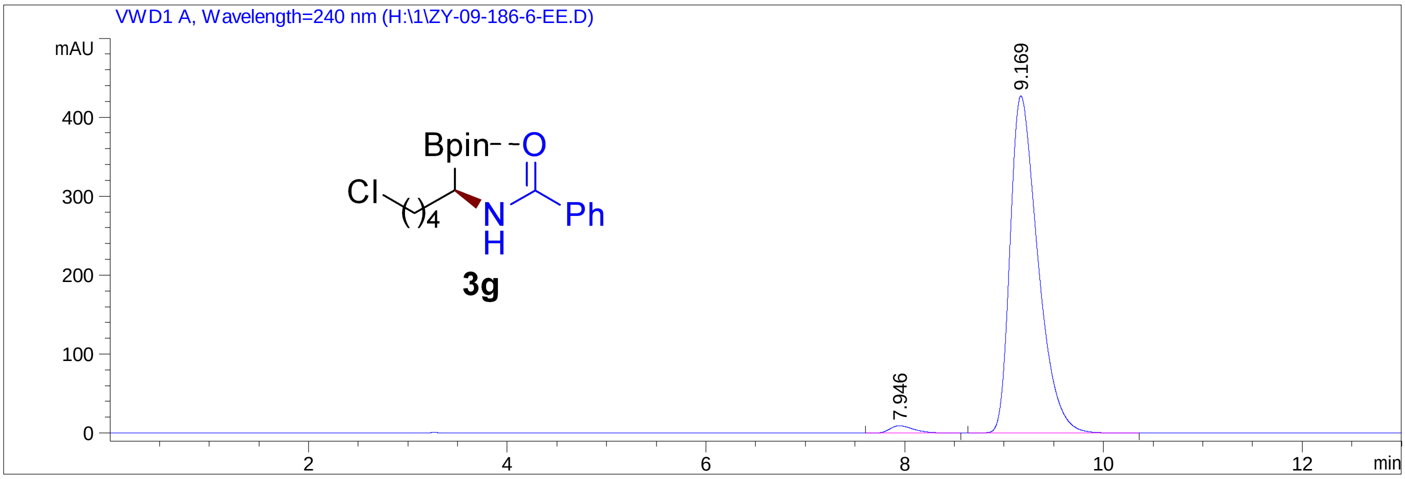

===

Area Percent Report

===

Sorted By   :   Signal
Multiplier   :   1.0000
Dilution   :   1.0000
Do not use Multiplier & Dilution Factor with ISTDs

Signal 1: VWD1 A, Wavelength=240 nm

Peak #	RetTime [min]	Type	Width [min]	Area [mAU*s]	Height [mAU]	Area %
1	7.946	BB	0.2433	155.65434	9.34727	1.8400
2	9.169	BB	0.2853	8304.04688	427.10986	98.1600

Totals :   8459.70122 436.45713

===

*** End of Report ***

Supplementary Figure 203: HPLC spectrum of **3g**.

Data File F:\ZY\AMIDE\ORIGINAL DATA\ZY-09-187-6-RAC.D
Sample Name: ZY-09-187-6-RAC

===

Acq. Operator : 系统 Seq. Line : 19
Acq. Instrument : HPLC-1260 Location : 62
Injection Date : 12/24/2021 3:31:27 PM Inj : 1
Inj Volume : 3.000 µl
Different Inj Volume from Sample Entry! Actual Inj Volume : 0.200 µl
Acq. Method : D:\zy\20211219\YH 2021-12-24 08-46-39\5EtOH15_8-1-2-240-.M
Last changed : 12/24/2021 12:39:49 PM by 系统
Analysis Method : E:\DATA\20220316\LC 2022-03-16 22-19-08\0.6IPA-15-0.8-1-210-JXL.M (Sequence Method)
Last changed : 3/29/2022 9:29:19 AM by SYSTEM
(modified after loading)
Additional Info : Peak(s) manually integrated

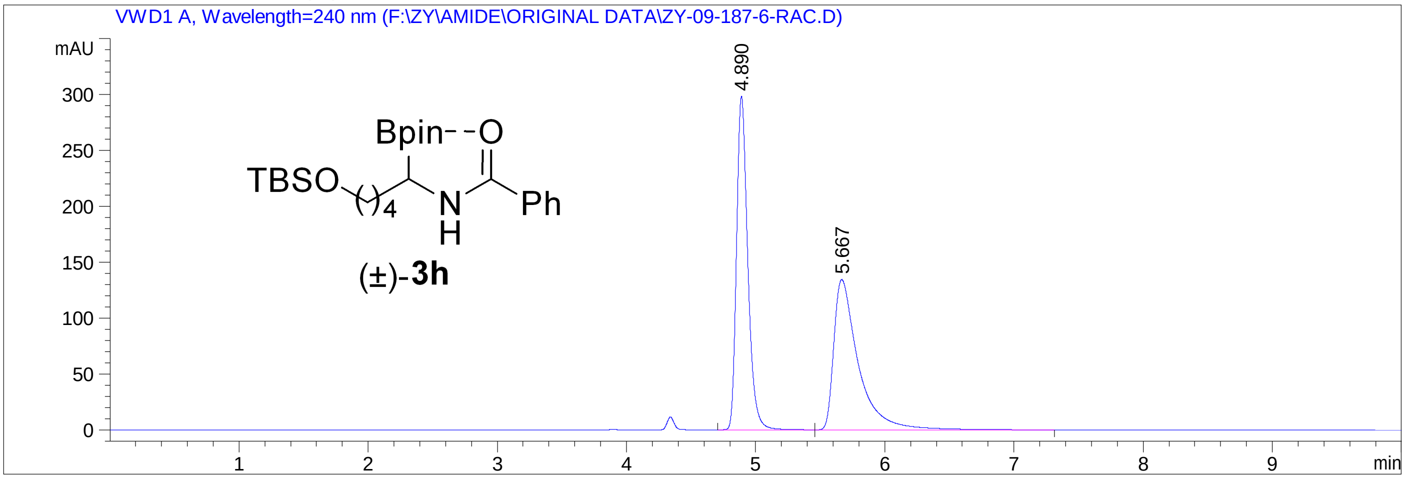

===

Area Percent Report

===

Sorted By : Signal
Multiplier : 1.0000
Dilution : 1.0000
Do not use Multiplier & Dilution Factor with ISTDs

Signal 1: VWD1 A, Wavelength=240 nm

Peak #	RetTime [min]	Type	Width [min]	Area [mAU*s]	Height [mAU]	Area %
1	4.890	BB	0.0919	1800.99927	298.45135	50.3802
2	5.667	BB	0.1930	1773.81934	134.34425	49.6198

Totals : 3574.81860 432.79561

===

*** End of Report ***

Supplementary Figure 204: HPLC spectrum of (±)-**3h**.

Data File F:\ZY\AMIDE\ORIGINAL DATA\ZY-09-187-6-EE.D
Sample Name: ZY-09-187-6-EE

===
Acq. Operator   : 系统   Seq. Line : 18
Acq. Instrument : HPLC-1260   Location :   61
Injection Date : 12/24/2021 3:15:06 PM   Inj :   1
                                              Inj Volume : 3.000 µl
Different Inj Volume from Sample Entry! Actual Inj Volume : 0.400 µl
Acq. Method   : D:\zy\20211219\YH 2021-12-24 08-46-39\5EtOH15_8-1-2-240-.M
Last changed   : 12/24/2021 12:39:49 PM by 系统
Analysis Method : E:\DATA\20220316\LC 2022-03-16 22-19-08\0.6IPA-15-0.8-1-210-JXL.M (Sequence Method)
Last changed   : 3/29/2022 9:28:52 AM by SYSTEM
                  (modified after loading)

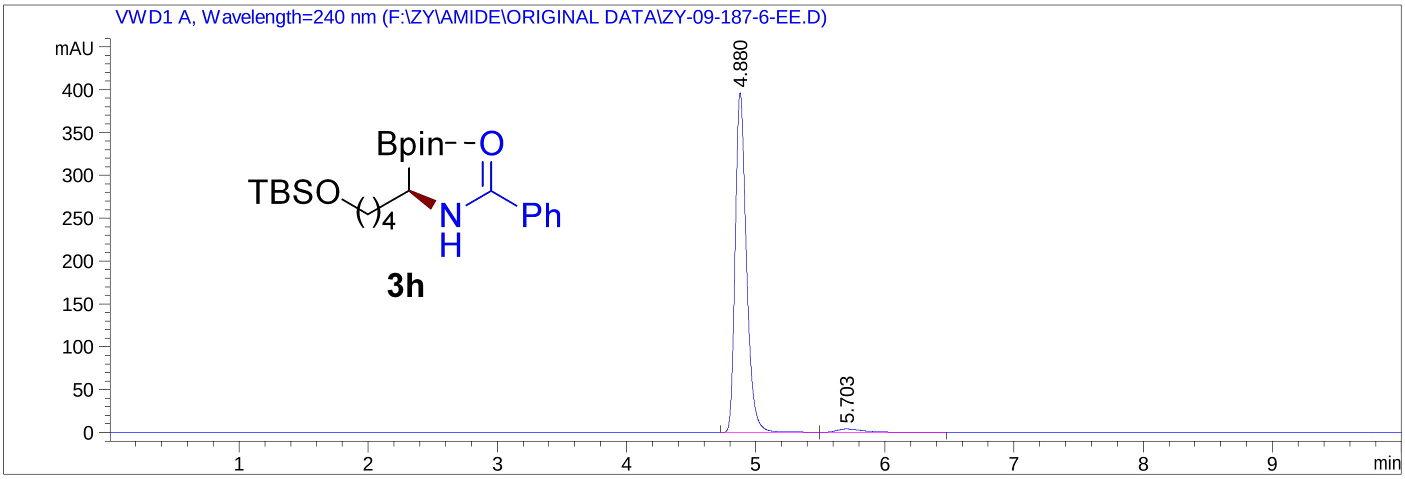

===
Area Percent Report
===

Sorted By : Signal
Multiplier : 1.0000
Dilution : 1.0000
Do not use Multiplier & Dilution Factor with ISTDs

Signal 1: VWD1 A, Wavelength=240 nm

Peak #	RetTime [min]	Type	Width [min]	Area [mAU*s]	Height [mAU]	Area %
1	4.880	BV R	0.0919	2393.51636	396.38632	97.5608
2	5.703	BB	0.2241	59.84169	3.93127	2.4392

Totals : 2453.35805 400.31759

===
*** End of Report ***

Supplementary Figure 205: HPLC spectrum of **3h**.

Data File E:\DATA\20210920\LC 2021-12-06 14-33-37\OnlineEdited--061.D
Sample Name: ZY-09-168-5-RAC

===
Acq. Operator   : SYSTEM   Seq. Line : 61
Acq. Instrument : HPLC1260   Location :   P1-E2
Injection Date : 12/7/2021 6:44:55 PM   Inj :   1
                                                Inj Volume : 3.000 µl
Different Inj Volume from Sample Entry! Actual Inj Volume : 0.500 µl
Acq. Method   : E:\DATA\20210920\LC 2021-12-06 14-33-37\5EtOH_15_10_4.M
Last changed   : 12/7/2021 5:06:46 PM by SYSTEM
Analysis Method : E:\DATA\20210920\LC 2021-12-06 14-33-37\5EtOH_15_10_4.M (Sequence Method)
Last changed   : 12/7/2021 9:56:54 PM by SYSTEM
                  (modified after loading)
Additional Info : Peak(s) manually integrated

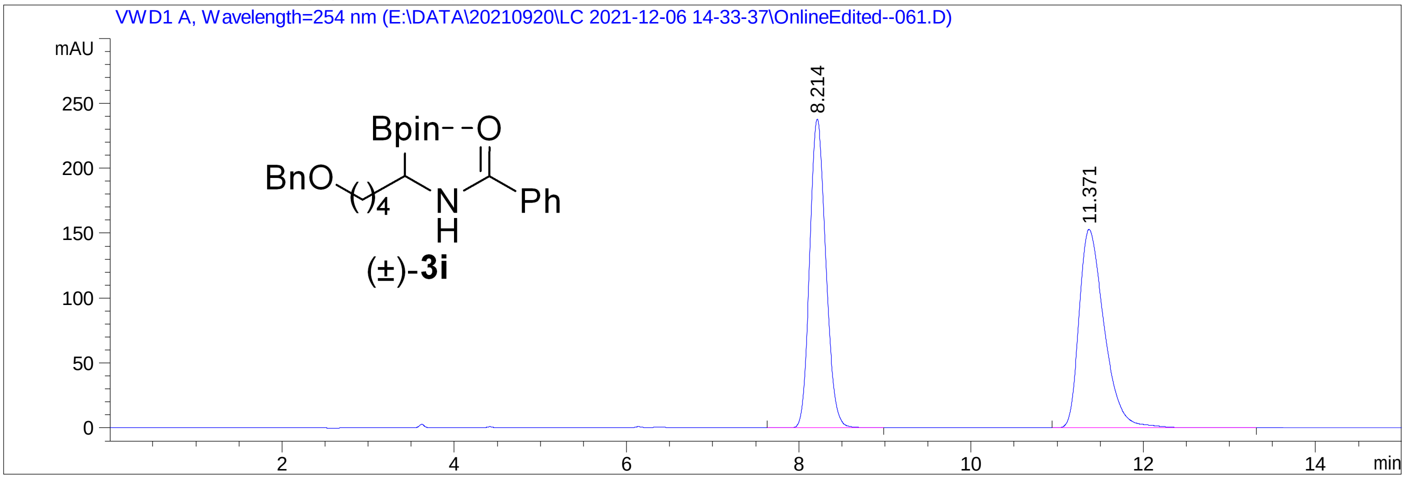

===
Area Percent Report
===

Sorted By   :   Signal
Multiplier   :   1.0000
Dilution   :   1.0000
Do not use Multiplier & Dilution Factor with ISTDs

Signal 1: VWD1 A, Wavelength=254 nm

Peak #	RetTime [min]	Type	Width [min]	Area [mAU*s]	Height [mAU]	Area %
1	8.214	BB	0.2025	3088.16382	237.84897	49.9227
2	11.371	BB	0.3116	3097.72485	152.74744	50.0773

Totals : 6185.88867 390.59641

===
*** End of Report ***

Supplementary Figure 206: HPLC spectrum of (±)-**3i**.

Data File E:\DATA\20210920\LC 2021-12-06 14-33-37\OnlineEdited--072.D
Sample Name: ZY-09-168-5-EE

===
Acq. Operator : SYSTEM Seq. Line : 72
Acq. Instrument : HPLC1260 Location : P1-E1
Injection Date : 12/7/2021 9:40:07 PM Inj : 1
Inj Volume : 3.000 µl
Different Inj Volume from Sample Entry! Actual Inj Volume : 0.800 µl
Acq. Method : E:\DATA\20210920\LC 2021-12-06 14-33-37\5EtOH_15_10_4.M
Last changed : 12/7/2021 9:19:59 PM by SYSTEM
Analysis Method : E:\DATA\20210920\LC 2021-12-06 14-33-37\5EtOH_15_10_4.M (Sequence Method)
Last changed : 12/7/2021 9:56:16 PM by SYSTEM
(modified after loading)

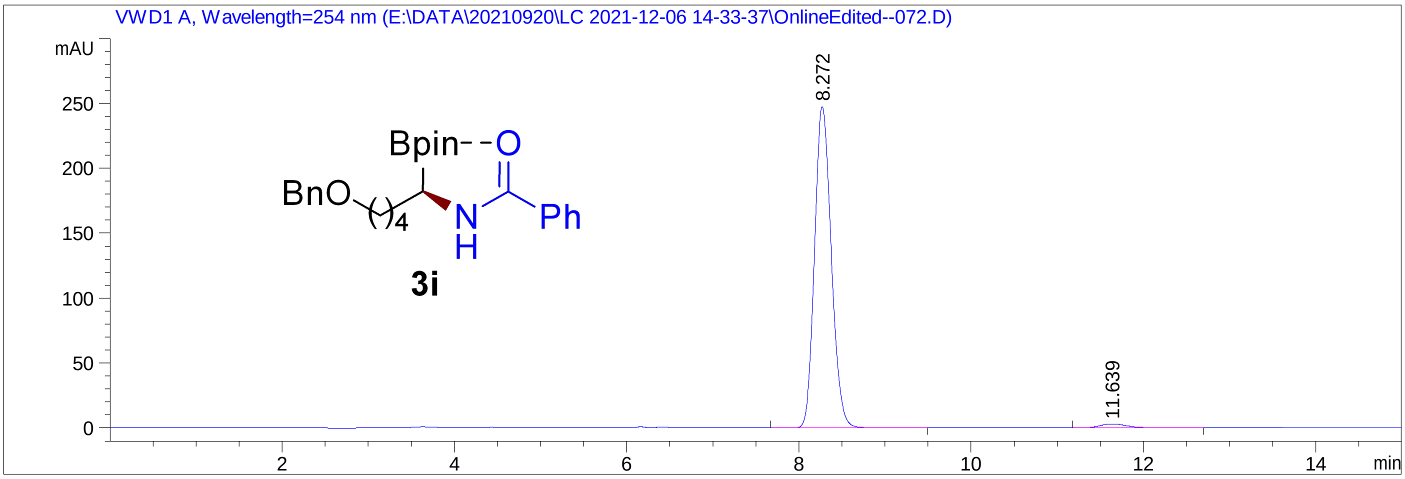

===
Area Percent Report
===

Sorted By : Signal
Multiplier : 1.0000
Dilution : 1.0000
Do not use Multiplier & Dilution Factor with ISTDs

Signal 1: VWD1 A, Wavelength=254 nm

Peak #	RetTime [min]	Type	Width [min]	Area [mAU*s]	Height [mAU]	Area %
1	8.272	BB	0.2106	3343.16821	247.41263	97.8705
2	11.639	BB	0.3530	72.74094	3.13104	2.1295

Totals : 3415.90915 250.54367

===
*** End of Report ***

Supplementary Figure 207: HPLC spectrum of **3i**.

Data File H:\1\ZY-09-192-2-RAC.D
Sample Name: ZY-09-192-2-RAC

===

Acq. Operator : 系统 Seq. Line : 39
Acq. Instrument : HPLC-1260 Location : 72
Injection Date : 8/20/2022 6:59:26 PM Inj : 1
Inj Volume : 3.000 µl
Different Inj Volume from Sample Entry! Actual Inj Volume : 0.800 µl
Acq. Method : D:\zy\20220803\YH 2022-08-20 08-41-06\10EtOH20_10-1-6-254.M
Last changed : 8/20/2022 1:58:22 PM by 系统
Analysis Method : E:\DATA\20220317\LC 2022-08-22 08-37-45\5IPA-40-1.0-1-220-QDY.M (Sequence Method)
Last changed : 8/22/2022 7:14:35 PM by SYSTEM
(modified after loading)
Additional Info : Peak(s) manually integrated

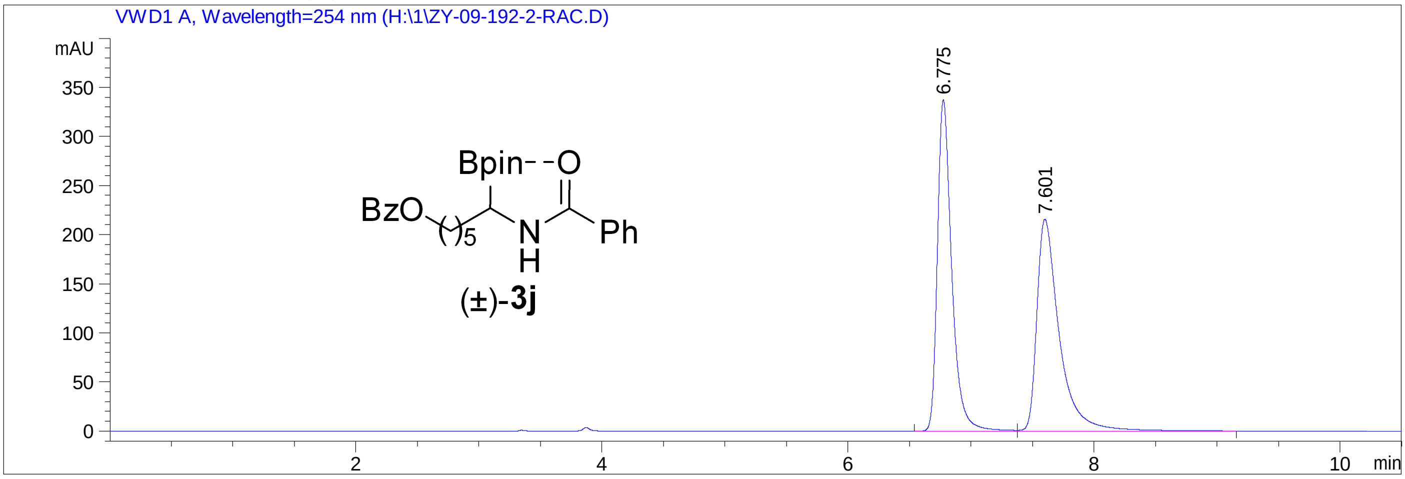

===

Area Percent Report

===

Sorted By : Signal
Multiplier : 1.0000
Dilution : 1.0000
Do not use Multiplier & Dilution Factor with ISTDs

Signal 1: VWD1 A, Wavelength=254 nm

Peak #	RetTime [min]	Type	Width [min]	Area [mAU*s]	Height [mAU]	Area %
1	6.775	BV	0.1214	2731.93823	337.47751	49.9922
2	7.601	VB	0.1882	2732.78638	215.87651	50.0078
Totals :				5464.72461	553.35402	

===

*** End of Report ***

Supplementary Figure 208: HPLC spectrum of (±)-**3j**.

Data File H:\1\ZY-09-192-2-EE.D
Sample Name: ZY-09-192-2-EE

===

Acq. Operator   : 系统   Seq. Line : 38
Acq. Instrument : HPLC-1260   Location :   71
Injection Date : 8/20/2022 6:37:59 PM   Inj :   1
                                               Inj Volume : 3.000 µl
Different Inj Volume from Sample Entry! Actual Inj Volume : 0.800 µl
Acq. Method   : D:\zy\20220803\YH 2022-08-20 08-41-06\10EtOH20_10-1-6-254.M
Last changed   : 8/20/2022 1:58:22 PM by 系统
Analysis Method : E:\DATA\20220317\LC 2022-08-22 08-37-45\5IPA-40-1.0-1-220-QDY.M (Sequence Method)
Last changed   : 8/22/2022 7:12:33 PM by SYSTEM
                  (modified after loading)
Additional Info : Peak(s) manually integrated

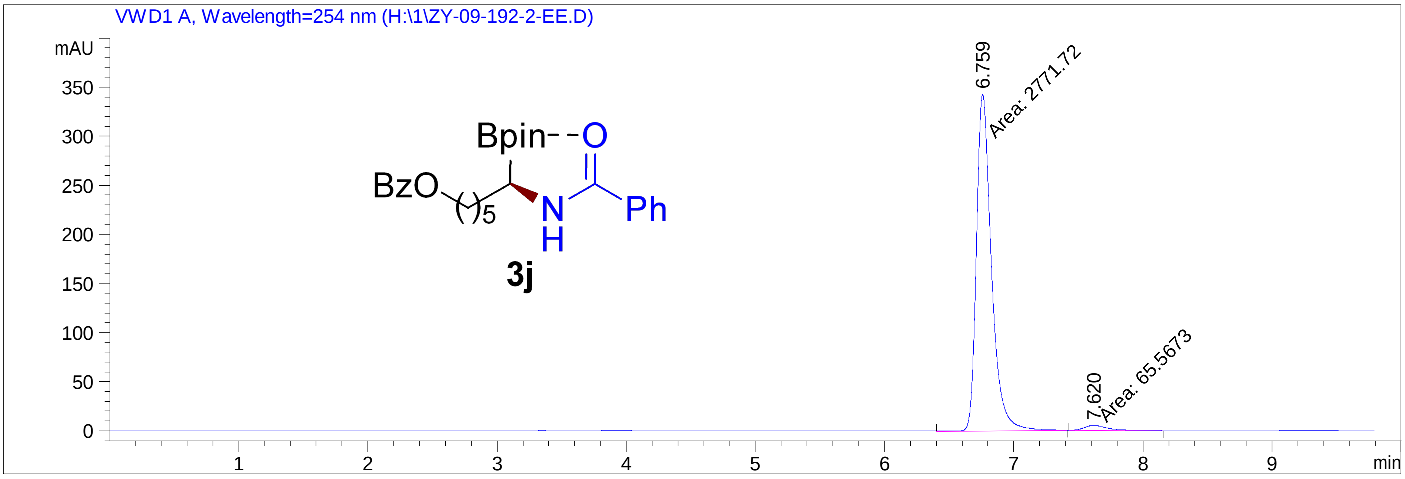

===

Area Percent Report

===

Sorted By   :   Signal
Multiplier   :   1.0000
Dilution   :   1.0000
Do not use Multiplier & Dilution Factor with ISTDs

Signal 1: VWD1 A, Wavelength=254 nm

Peak #	RetTime [min]	Type	Width [min]	Area [mAU*s]	Height [mAU]	Area %
1	6.759	MM	0.1347	2771.72437	342.95248	97.6891
2	7.620	MM	0.2110	65.56731	5.17876	2.3109

Totals :   2837.29167   348.13124

===

*** End of Report ***

Supplementary Figure 209: HPLC spectrum of **3j**.

Data File H:\1\ZY-10-115-5-RAC.D
Sample Name: ZY-10-115-6-12

===
Acq. Operator   : 系统   Seq. Line : 27
Acq. Instrument : HPLC-1260   Location :   54
Injection Date : 8/19/2022 10:13:31 PM   Inj :   1
                                               Inj Volume : 3.000 µl
Different Inj Volume from Sample Entry! Actual Inj Volume : 4.000 µl
Acq. Method   : D:\zy\20220803\YH 2022-08-19 14-14-07\20EtOH20_10-1-2-254.M
Last changed   : 8/19/2022 2:14:07 PM by 系统
Analysis Method : E:\DATA\20220317\LC 2022-08-22 08-37-45\5IPA-40-1.0-1-220-QDY.M (Sequence Method)
Last changed   : 8/22/2022 7:33:29 PM by SYSTEM
                  (modified after loading)
Additional Info : Peak(s) manually integrated

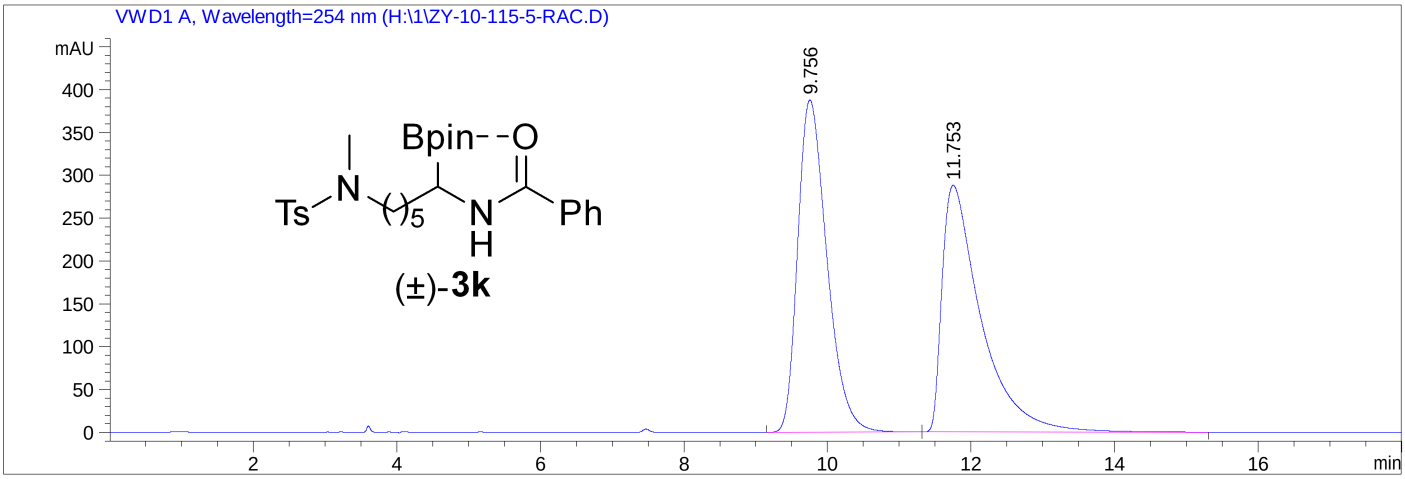

===
Area Percent Report
===

Sorted By : Signal
Multiplier : 1.0000
Dilution : 1.0000
Do not use Multiplier & Dilution Factor with ISTDs

Signal 1: VWD1 A, Wavelength=254 nm

Peak #	RetTime [min]	Type	Width [min]	Area [mAU*s]	Height [mAU]	Area %
1	9.756	BB	0.4317	1.09960e4	387.94690	50.3217
2	11.753	BB	0.5502	1.08554e4	288.05551	49.6783

Totals : 2.18514e4 676.00241

===
*** End of Report ***

Supplementary Figure 210: HPLC spectrum of (±)-**3k**.

Data File H:\OnlineEdited--024.D
Sample Name: ZY-10-115-5-13

===

Acq. Operator   : 系统   Seq. Line : 24
Acq. Instrument : HPLC-1260   Location :   75
Injection Date : 8/19/2022 9:19:43 PM   Inj :   1
                                            Inj Volume : 3.000 µl
Different Inj Volume from Sample Entry! Actual Inj Volume : 3.200 µl
Acq. Method   : D:\zy\20220803\YH 2022-08-19 14-14-07\20EtOH20_10-1-2-254.M
Last changed   : 8/19/2022 2:14:07 PM by 系统
Analysis Method : E:\DATA\20220317\LC 2022-08-19 14-05-28\10IPA_10_8_3.M (Sequence Method)
Last changed   : 8/19/2022 10:26:56 PM by SYSTEM
                  (modified after loading)
Additional Info : Peak(s) manually integrated

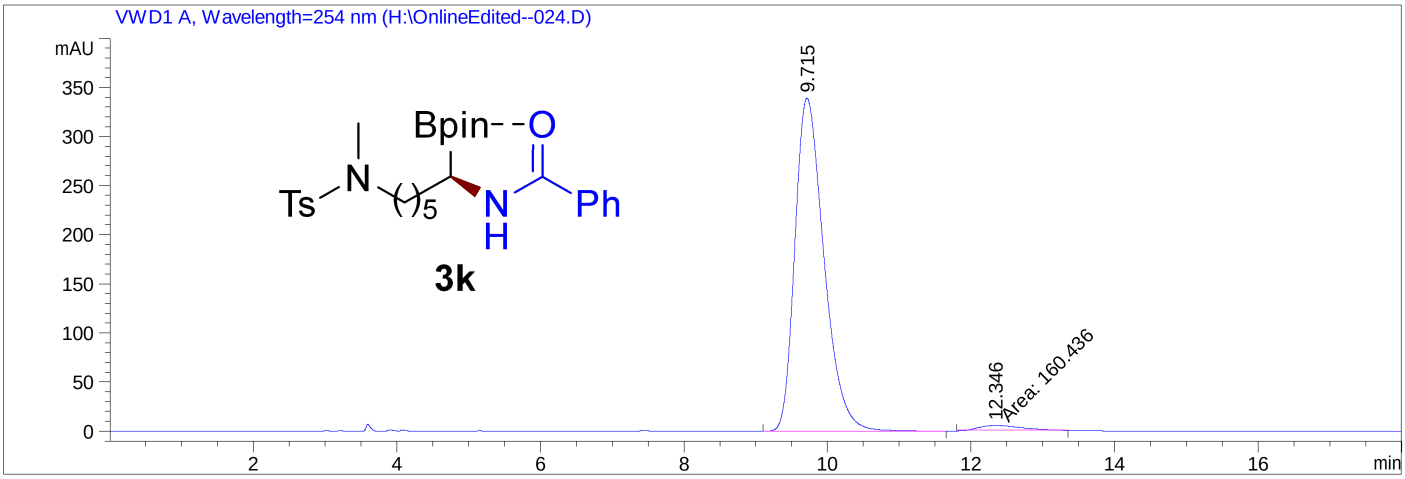

===

Area Percent Report

===

Sorted By   :   Signal
Multiplier   :   1.0000
Dilution   :   1.0000
Do not use Multiplier & Dilution Factor with ISTDs

Signal 1: VWD1 A, Wavelength=254 nm

Peak #	RetTime [min]	Type	Width [min]	Area [mAU*s]	Height [mAU]	Area %
1	9.715	BB	0.4357	9538.70508	339.05954	98.3459
2	12.346	MM	0.5951	160.43559	4.49287	1.6541

Totals :   9699.14067 343.55241

===

*** End of Report ***

Supplementary Figure 211: HPLC spectrum of **3k**.

Data File F:\ZY\AMIDE\ORIGINAL DATA\ZY-10-12-12-RAC.D
Sample Name: ZY-10-12-12-RAC

===

Acq. Operator : 系统 Seq. Line : 9
Acq. Instrument : HPLC-1260 Location : 81
Injection Date : 3/14/2022 8:10:38 PM Inj : 1
Inj Volume : 3.000 µl
Different Inj Volume from Sample Entry! Actual Inj Volume : 1.200 µl
Acq. Method : D:\zy\20220302\YH 2022-03-14 18-14-35\8IPA20_8-1-2-240.M
Last changed : 3/14/2022 7:50:44 PM by 系统
Analysis Method : E:\DATA\20220316\LC 2022-03-16 22-19-08\10IPA-10-1.0-1.MZWJ.M (Sequence Method)
Last changed : 3/29/2022 9:59:19 AM by SYSTEM
(modified after loading)
Additional Info : Peak(s) manually integrated

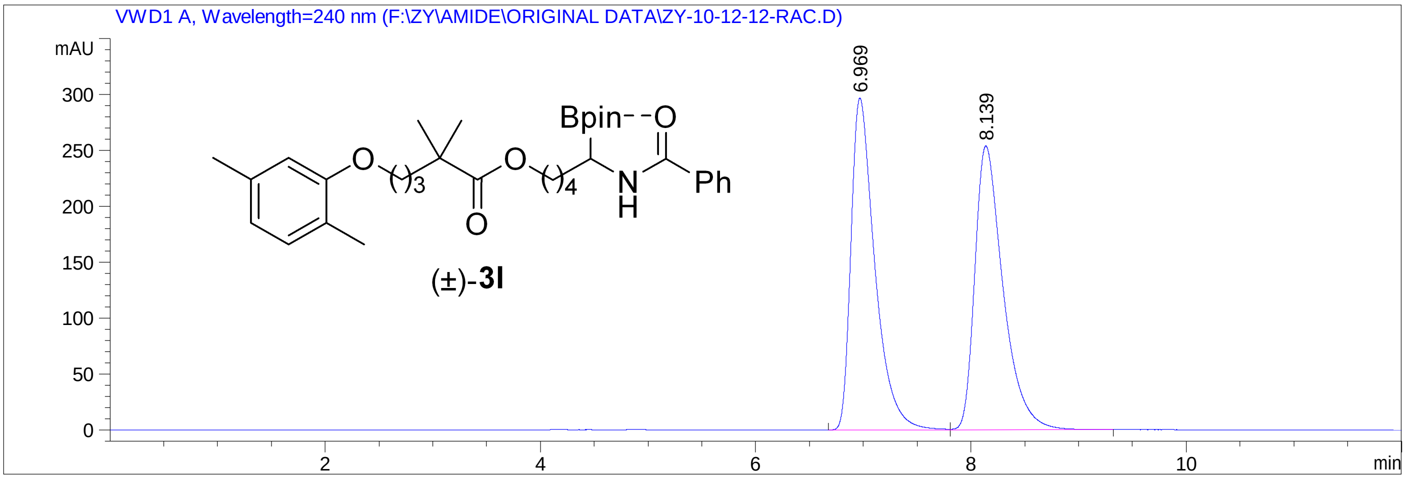

===

Area Percent Report

===

Sorted By : Signal
Multiplier : 1.0000
Dilution : 1.0000
Do not use Multiplier & Dilution Factor with ISTDs

Signal 1: VWD1 A, Wavelength=240 nm

Peak #	RetTime [min]	Type	Width [min]	Area [mAU*s]	Height [mAU]	Area %
1	6.969	BV	0.2315	4579.07910	296.74182	49.7372
2	8.139	VB	0.2682	4627.47461	253.77185	50.2628

Totals : 9206.55371 550.51367

===

*** End of Report ***

Supplementary Figure 212: HPLC spectrum of (±)-**3l**.

Data File F:\ZY\AMIDE\ORIGINAL DATA\ZY-10-12-12-EE.D
Sample Name: ZY-10-12-12-EE

===

Acq. Operator : 系统 Seq. Line : 8
Acq. Instrument : HPLC-1260 Location : 82
Injection Date : 3/14/2022 7:52:12 PM Inj : 1
Inj Volume : 3.000 µl
Different Inj Volume from Sample Entry! Actual Inj Volume : 0.600 µl
Acq. Method : D:\zy\20220302\YH 2022-03-14 18-14-35\8IPA20_8-1-2-240.M
Last changed : 3/14/2022 7:50:44 PM by 系统
Analysis Method : E:\DATA\20220316\LC 2022-03-16 22-19-08\10IPA-10-1.0-1.MZWJ.M (Sequence Method)
Last changed : 3/29/2022 9:59:57 AM by SYSTEM
(modified after loading)
Additional Info : Peak(s) manually integrated

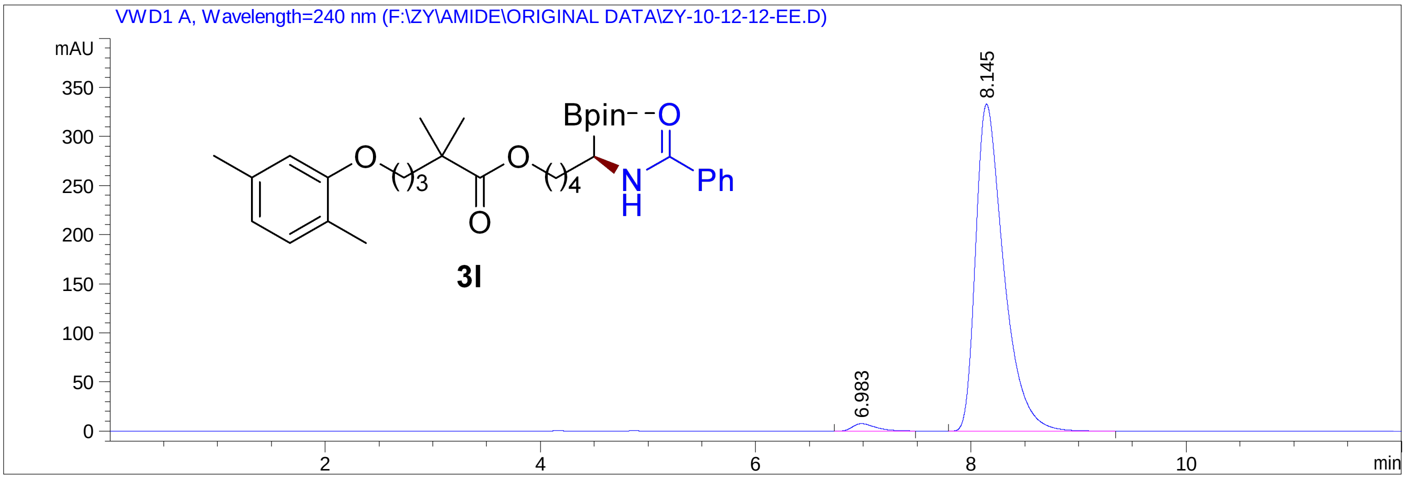

===

Area Percent Report

===

Sorted By : Signal
Multiplier : 1.0000
Dilution : 1.0000
Do not use Multiplier & Dilution Factor with ISTDs

Signal 1: VWD1 A, Wavelength=240 nm

Peak #	RetTime [min]	Type	Width [min]	Area [mAU*s]	Height [mAU]	Area %
1	6.983	BB	0.2180	115.32585	7.70988	1.8705
2	8.145	BB	0.2674	6050.16260	333.05563	98.1295

Totals : 6165.48845 340.76552

===

*** End of Report ***

Supplementary Figure 213: HPLC spectrum of **3l**.

Data File F:\ZY\AMIDE\ORIGINAL DATA\ZY-10-24-5-RAC.D
Sample Name: ZY-10-24-5-RAC

===

Acq. Operator : 系统 Seq. Line : 27
Acq. Instrument : HPLC-1260 Location : 71
Injection Date : 2/14/2022 10:13:51 PM Inj : 1
Inj Volume : 3.000 µl
Different Inj Volume from Sample Entry! Actual Inj Volume : 0.600 µl
Acq. Method : D:\zy\20220201\YH 2022-02-14 12-55-19\15EtOH30_10-1-2-254.M
Last changed : 2/14/2022 9:50:04 PM by 系统
Analysis Method : E:\DATA\20220316\LC 2022-03-16 22-19-08\10IPA-10-1.0-1.MZWJ.M (Sequence Method)
Last changed : 3/29/2022 10:02:54 AM by SYSTEM
(modified after loading)
Additional Info : Peak(s) manually integrated

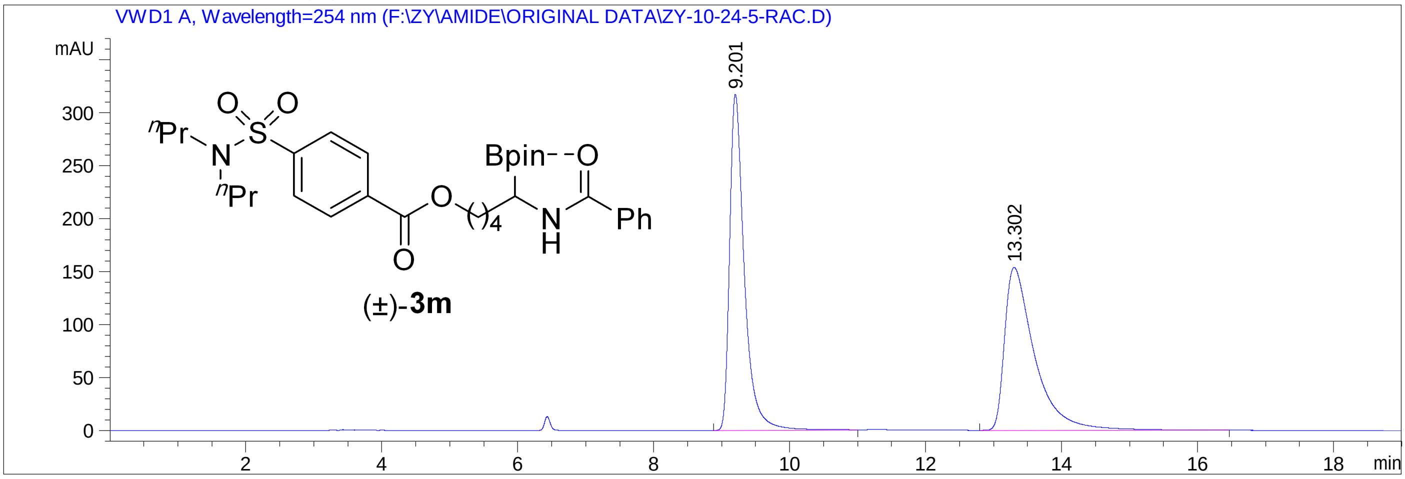

===

Area Percent Report

===

Sorted By : Signal
Multiplier : 1.0000
Dilution : 1.0000
Do not use Multiplier & Dilution Factor with ISTDs

Signal 1: VWD1 A, Wavelength=254 nm

Peak #	RetTime [min]	Type	Width [min]	Area [mAU*s]	Height [mAU]	Area %
1	9.201	BB	0.2237	4765.29834	317.25735	50.2247
2	13.302	BB	0.4520	4722.66553	153.67970	49.7753

Totals : 9487.96387 470.93706

===

*** End of Report ***

Supplementary Figure 214: HPLC spectrum of (±)-**3m**.

Data File F:\ZY\AMIDE\ORIGINAL DATA\ZY-10-24-5-EE.D
Sample Name: ZY-10-24-5-EE

===

Acq. Operator : 系统 Seq. Line : 26
Acq. Instrument : HPLC-1260 Location : 72
Injection Date : 2/14/2022 9:51:27 PM Inj : 1
Inj Volume : 3.000 µl
Different Inj Volume from Sample Entry! Actual Inj Volume : 0.600 µl
Acq. Method : D:\zy\20220201\YH 2022-02-14 12-55-19\15EtOH30_10-1-2-254.M
Last changed : 2/14/2022 9:50:04 PM by 系统
Analysis Method : E:\DATA\20220316\LC 2022-03-16 22-19-08\10IPA-10-1.0-1.MZWJ.M (Sequence Method)
Last changed : 3/29/2022 10:02:54 AM by SYSTEM
(modified after loading)
Additional Info : Peak(s) manually integrated

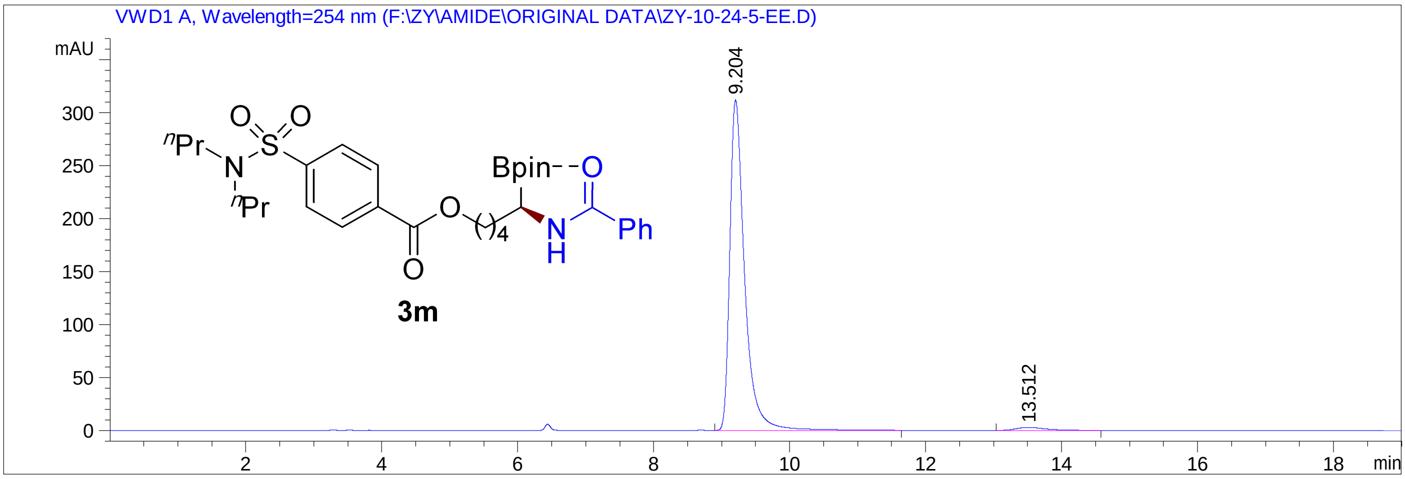

===
Area Percent Report
===

Sorted By : Signal
Multiplier : 1.0000
Dilution : 1.0000
Do not use Multiplier & Dilution Factor with ISTDs

Signal 1: VWD1 A, Wavelength=254 nm

Peak #	RetTime [min]	Type	Width [min]	Area [mAU*s]	Height [mAU]	Area %
1	9.204	BB	0.2267	4723.18945	311.85480	98.1437
2	13.512	BB	0.3615	89.33582	2.91907	1.8563

Totals : 4812.52527 314.77387

===
*** End of Report ***

Supplementary Figure 215: HPLC spectrum of **3m**.

Data File F:\ZY\AMIDE\ORIGINAL DATA\ZY-10-25-RAC.D
Sample Name: ZY-10-25-RAC

===

Acq. Operator : 系统 Seq. Line : 35
Acq. Instrument : HPLC-1260 Location : 81
Injection Date : 2/15/2022 12:45:00 AM Inj : 1
Inj Volume : 3.000 µl
Different Inj Volume from Sample Entry! Actual Inj Volume : 1.000 µl
Acq. Method : D:\zy\20220201\YH 2022-02-14 12-55-19\5IPA15_10-1-6-240.M
Last changed : 2/14/2022 9:25:27 PM by 系统
Analysis Method : E:\DATA\20220316\LC 2022-03-16 22-19-08\10IPA-10-1.0-1.MZWJ.M (Sequence Method)
Last changed : 3/29/2022 10:04:39 AM by SYSTEM
(modified after loading)

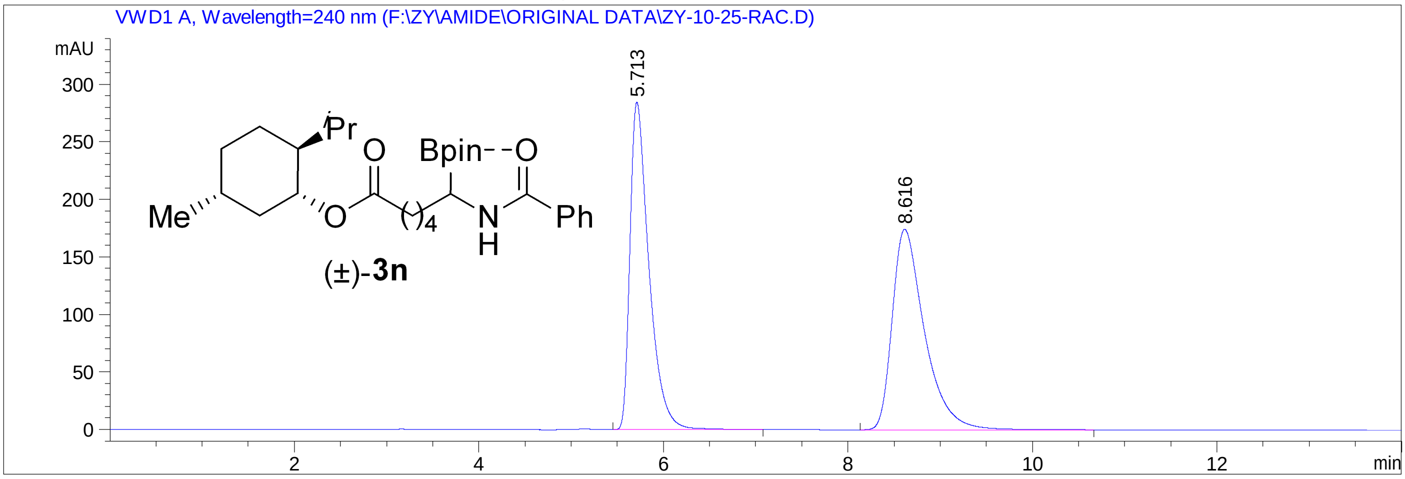

===

Area Percent Report

===

Sorted By : Signal
Multiplier : 1.0000
Dilution : 1.0000
Do not use Multiplier & Dilution Factor with ISTDs

Signal 1: VWD1 A, Wavelength=240 nm

Peak #	RetTime [min]	Type	Width [min]	Area [mAU*s]	Height [mAU]	Area %
1	5.713	BB	0.2128	4071.67749	284.85623	49.0033
2	8.616	BB	0.3652	4237.30811	174.26460	50.9967
Totals :				8308.98560	459.12083	

===

*** End of Report ***

Supplementary Figure 216: HPLC spectrum of (±)-**3n**.

Data File F:\ZY\AMIDE\ORIGINAL DATA\ZY-10-25-2-EE.D
Sample Name: ZY-10-25-2-EE

===

Acq. Operator : 系统 Seq. Line : 32
Acq. Instrument : HPLC-1260 Location : 83
Injection Date : 2/14/2022 11:55:42 PM Inj : 1
Inj Volume : 3.000 µl
Different Inj Volume from Sample Entry! Actual Inj Volume : 0.800 µl
Acq. Method : D:\zy\20220201\YH 2022-02-14 12-55-19\5IPA15_10-1-6-240.M
Last changed : 2/14/2022 9:25:27 PM by 系统
Analysis Method : E:\DATA\20220316\LC 2022-03-16 22-19-08\10IPA-10-1.0-1.MZWJ.M (Sequence Method)
Last changed : 3/29/2022 10:04:39 AM by SYSTEM
(modified after loading)

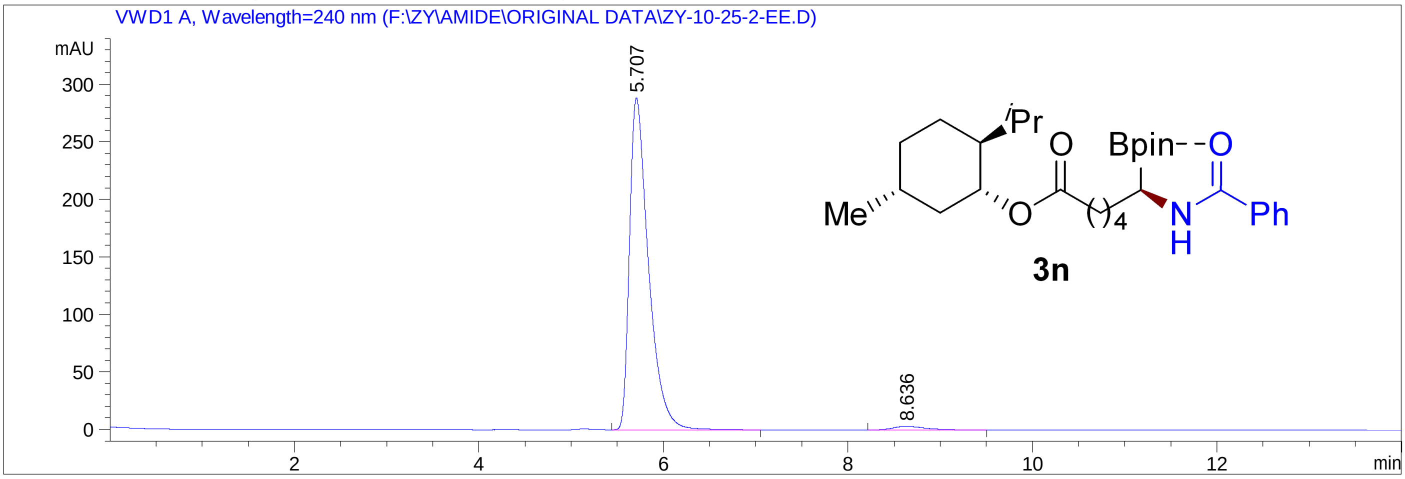

===

Area Percent Report

===

Sorted By : Signal
Multiplier : 1.0000
Dilution : 1.0000
Do not use Multiplier & Dilution Factor with ISTDs

Signal 1: VWD1 A, Wavelength=240 nm

Peak #	RetTime [min]	Type	Width [min]	Area [mAU*s]	Height [mAU]	Area %
1	5.707	BB	0.2122	4119.90674	288.39926	98.2076
2	8.636	BB	0.2825	75.19337	3.14431	1.7924

Totals : 4195.10011 291.54357

===

*** End of Report ***

Supplementary Figure 217: HPLC spectrum of **3n**.

Data File F:\ZY\AMIDE\ORIGINAL DATA\ZY-10-25-5-EE.D
Sample Name: ZY-10-25-5-EE

===

Acq. Operator : 系统 Seq. Line : 33
Acq. Instrument : HPLC-1260 Location : 82
Injection Date : 2/15/2022 12:12:05 AM Inj : 1
Inj Volume : 3.000 µl
Different Inj Volume from Sample Entry! Actual Inj Volume : 1.400 µl
Acq. Method : D:\zy\20220201\YH 2022-02-14 12-55-19\5IPA15_10-1-6-240.M
Last changed : 2/14/2022 9:25:27 PM by 系统
Analysis Method : E:\DATA\20220316\LC 2022-03-16 22-19-08\10IPA-10-1.0-1.MZWJ.M (Sequence Method)
Last changed : 3/29/2022 10:04:39 AM by SYSTEM
(modified after loading)

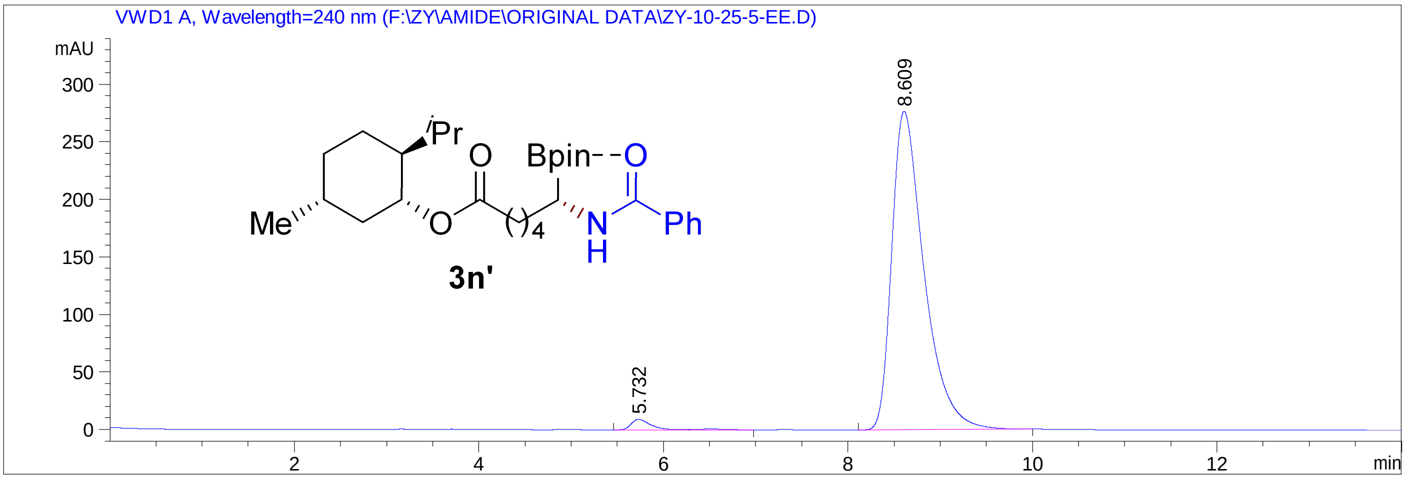

===

Area Percent Report

===

Sorted By : Signal
Multiplier : 1.0000
Dilution : 1.0000
Do not use Multiplier & Dilution Factor with ISTDs

Signal 1: VWD1 A, Wavelength=240 nm

Peak #	RetTime [min]	Type	Width [min]	Area [mAU*s]	Height [mAU]	Area %
1	5.732	BV R	0.2280	143.65533	9.10710	2.0901
2	8.609	BB	0.3617	6729.62012	276.75131	97.9099

Totals : 6873.27545 285.85841

===

*** End of Report ***

Supplementary Figure 218: HPLC spectrum of **3n'**.

Data File H:\A\ZY-10-91-3-RAC.D
Sample Name: ZY-10-91-3-RAC

===
Acq. Operator : 系统 Seq. Line : 5
Acq. Instrument : HPLC-1260 Location : 81
Injection Date : 6/26/2022 3:12:35 PM Inj : 1
Inj Volume : 3.000 µl
Different Inj Volume from Sample Entry! Actual Inj Volume : 0.600 µl
Acq. Method : D:\zy\20220624\YH 2022-06-26 14-05-25\12EtOH20_10-1-2-240.M
Last changed : 6/26/2022 2:05:25 PM by 系统
Analysis Method : E:\DATA\20220317\LC 2022-08-08 20-30-16\2IPA-50-0.8-3-210-JXL.M (Sequence Method)
Last changed : 8/9/2022 9:05:21 AM by SYSTEM
(modified after loading)

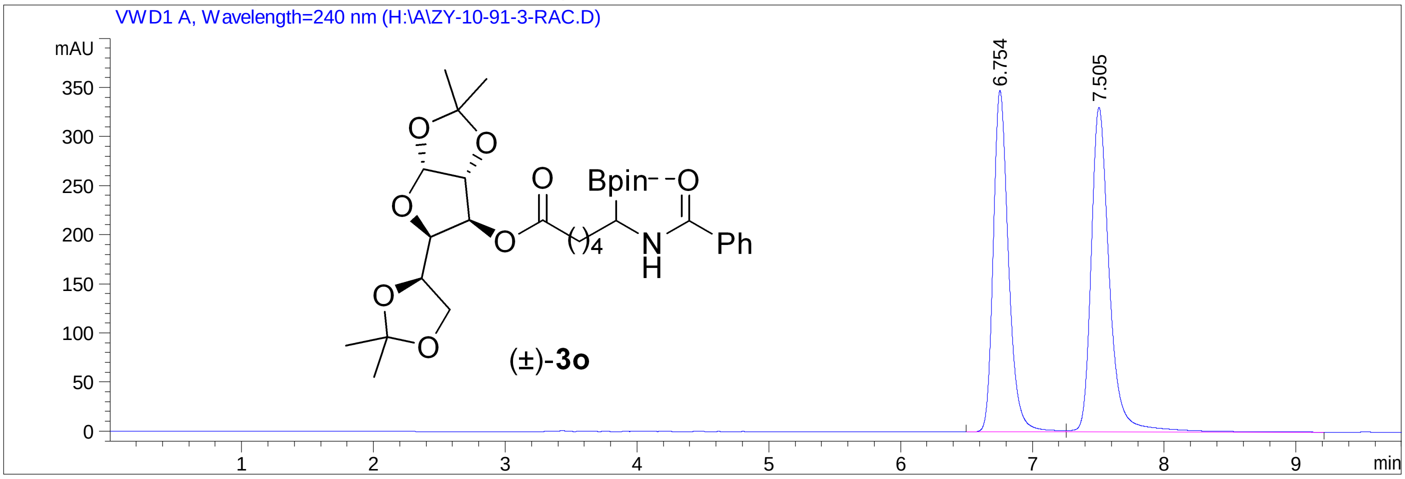

===
Area Percent Report
===

Sorted By : Signal
Multiplier : 1.0000
Dilution : 1.0000
Do not use Multiplier & Dilution Factor with ISTDs

Signal 1: VWD1 A, Wavelength=240 nm

Peak #	RetTime [min]	Type	Width [min]	Area [mAU*s]	Height [mAU]	Area %
1	6.754	BV	0.1230	2800.11133	347.39279	46.7353
2	7.505	VB	0.1456	3191.31055	330.62488	53.2647

Totals : 5991.42188 678.01767

===
*** End of Report ***

Supplementary Figure 219: HPLC spectrum of (±)-**3o**.

Data File H:\A\ZY-10-91-2-EE.D
Sample Name: ZY-10-91-2-EE

===

Acq. Operator : 系统 Seq. Line : 3
Acq. Instrument : HPLC-1260 Location : 82
Injection Date : 6/26/2022 2:29:40 PM Inj : 1
Inj Volume : 3.000 µl
Different Inj Volume from Sample Entry! Actual Inj Volume : 0.600 µl
Acq. Method : D:\zy\20220624\YH 2022-06-26 14-05-25\12EtOH20_10-1-2-240.M
Last changed : 6/26/2022 2:05:25 PM by 系统
Analysis Method : E:\DATA\20220317\LC 2022-08-08 20-30-16\2IPA-50-0.8-3-210-JXL.M (Sequence Method)
Last changed : 8/9/2022 9:03:39 AM by SYSTEM
(modified after loading)
Additional Info : Peak(s) manually integrated

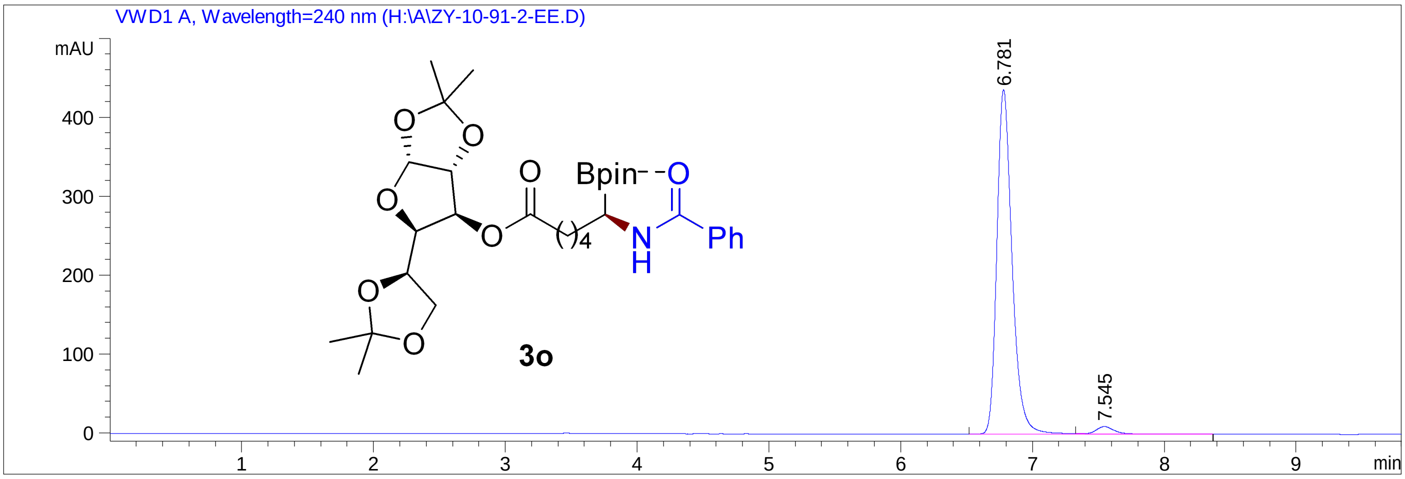

===

Area Percent Report

===

Sorted By : Signal
Multiplier : 1.0000
Dilution : 1.0000
Do not use Multiplier & Dilution Factor with ISTDs

Signal 1: VWD1 A, Wavelength=240 nm

Peak #	RetTime [min]	Type	Width [min]	Area [mAU*s]	Height [mAU]	Area %
1	6.781	BV R	0.1229	3513.69751	436.44815	97.4507
2	7.545	VB E	0.1465	91.91974	9.48676	2.5493

Totals : 3605.61725 445.93491

===

*** End of Report ***

Supplementary Figure 220: HPLC spectrum of **3o**.

Data File H:\A\ZY-10-91-5-EE.D
Sample Name: ZY-10-91-5-EE

```
=====================================================================
Acq. Operator   : 系统                          Seq. Line :    4
Acq. Instrument : HPLC-1260                      Location :   83
Injection Date  : 6/26/2022 2:51:11 PM                Inj :    1
                                               Inj Volume : 3.000 µl
Different Inj Volume from Sample Entry!  Actual Inj Volume : 0.600 µl
Acq. Method     : D:\zy\20220624\YH 2022-06-26 14-05-25\12EtOH20_10-1-2-240.M
Last changed    : 6/26/2022 2:05:25 PM by 系统
Analysis Method : E:\DATA\20220317\LC 2022-08-08 20-30-16\2IPA-50-0.8-3-210-JXL.M (Sequence
                  Method)
Last changed    : 8/9/2022 9:04:32 AM by SYSTEM
                  (modified after loading)
Additional Info : Peak(s) manually integrated
```

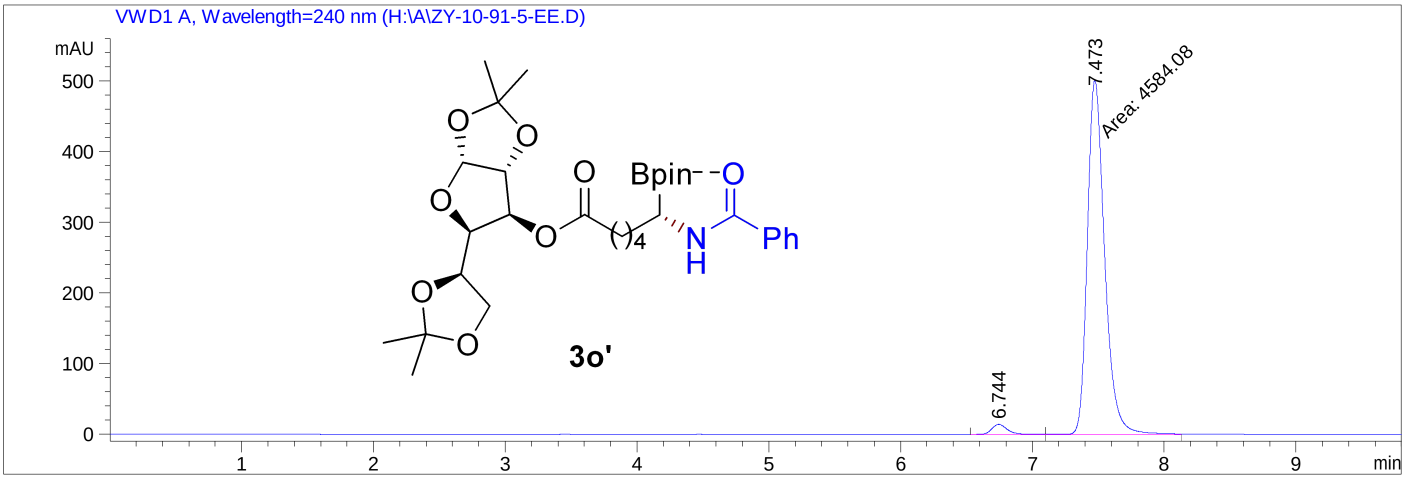


```
=====================================================================
                         Area Percent Report
=====================================================================

Sorted By             :      Signal
Multiplier            :      1.0000
Dilution              :      1.0000
Do not use Multiplier & Dilution Factor with ISTDs


Signal 1: VWD1 A, Wavelength=240 nm
```

Peak #	RetTime [min]	Type	Width [min]	Area [mAU*s]	Height [mAU]	Area %
1	6.744	BV	0.1267	118.68422	14.24024	2.5237
2	7.473	MF	0.1523	4584.07568	501.71143	97.4763

Totals : 4702.75990 515.95166

```
=====================================================================
                        *** End of Report ***
```

Supplementary Figure 221: HPLC spectrum of **3o'**.

Data File H:\A\ZY-10-101-3-RAC.D
Sample Name: ZY-10-101-3-RAC

===

Acq. Operator : 系统 Seq. Line : 33
Acq. Instrument : HPLC-1260 Location : 61
Injection Date : 7/5/2022 1:25:35 AM Inj : 1
Inj Volume : 3.000 µl
Different Inj Volume from Sample Entry! Actual Inj Volume : 0.600 µl
Acq. Method : D:\zy\20220624\YH 2022-07-04 14-04-41\10EtOH15_10-1-6-240.M
Last changed : 7/4/2022 7:42:02 PM by 系统
Analysis Method : E:\DATA\20220317\LC 2022-08-08 20-30-16\2IPA-50-0.8-3-210-JXL.M (Sequence Method)
Last changed : 8/9/2022 9:10:28 AM by SYSTEM
(modified after loading)

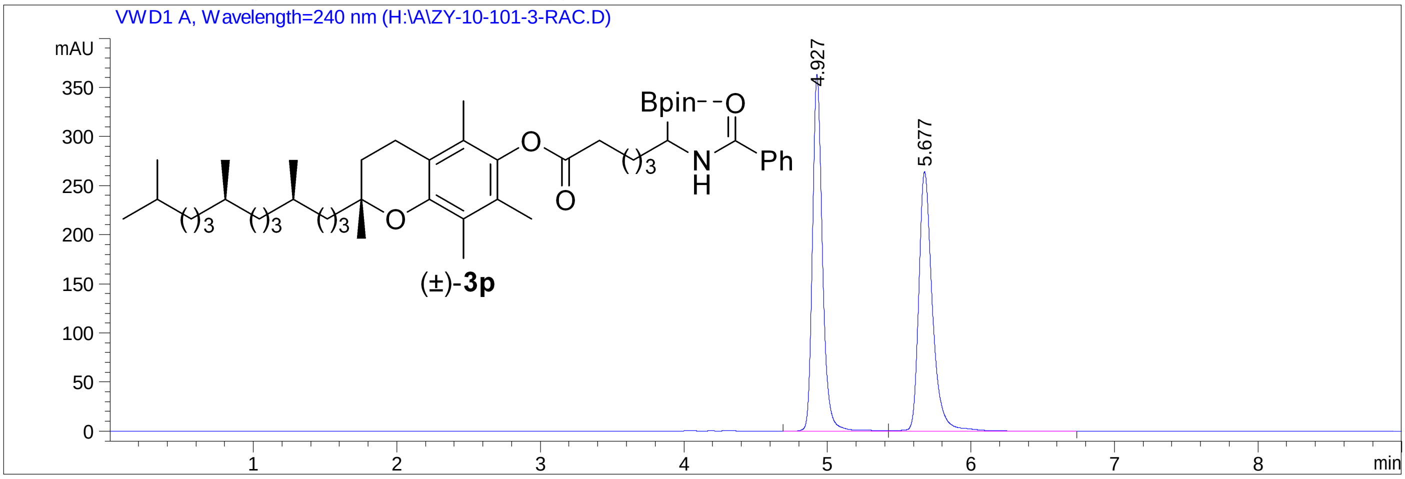

===

Area Percent Report

===

Sorted By : Signal
Multiplier : 1.0000
Dilution : 1.0000
Do not use Multiplier & Dilution Factor with ISTDs

Signal 1: VWD1 A, Wavelength=240 nm

Peak #	RetTime [min]	Type	Width [min]	Area [mAU*s]	Height [mAU]	Area %
1	4.927	BV R	0.0736	1760.48340	363.18640	50.2205
2	5.677	VB	0.0991	1745.02710	264.16177	49.7795
Totals :				3505.51050	627.34818	

===

*** End of Report ***

Supplementary Figure 222: HPLC spectrum of (±)-**3p**.

Data File H:\A\ZY-10-101-2-EE.D
Sample Name: ZY-10-101-2-EE

===

Acq. Operator : 系统 Seq. Line : 31
Acq. Instrument : HPLC-1260 Location : 62
Injection Date : 7/5/2022 12:52:46 AM Inj : 1
Inj Volume : 3.000 µl
Different Inj Volume from Sample Entry! Actual Inj Volume : 0.300 µl
Acq. Method : D:\zy\20220624\YH 2022-07-04 14-04-41\10EtOH15_10-1-6-240.M
Last changed : 7/4/2022 7:42:02 PM by 系统
Analysis Method : E:\DATA\20220317\LC 2022-08-08 20-30-16\2IPA-50-0.8-3-210-JXL.M (Sequence Method)
Last changed : 8/9/2022 9:09:56 AM by SYSTEM
(modified after loading)
Additional Info : Peak(s) manually integrated

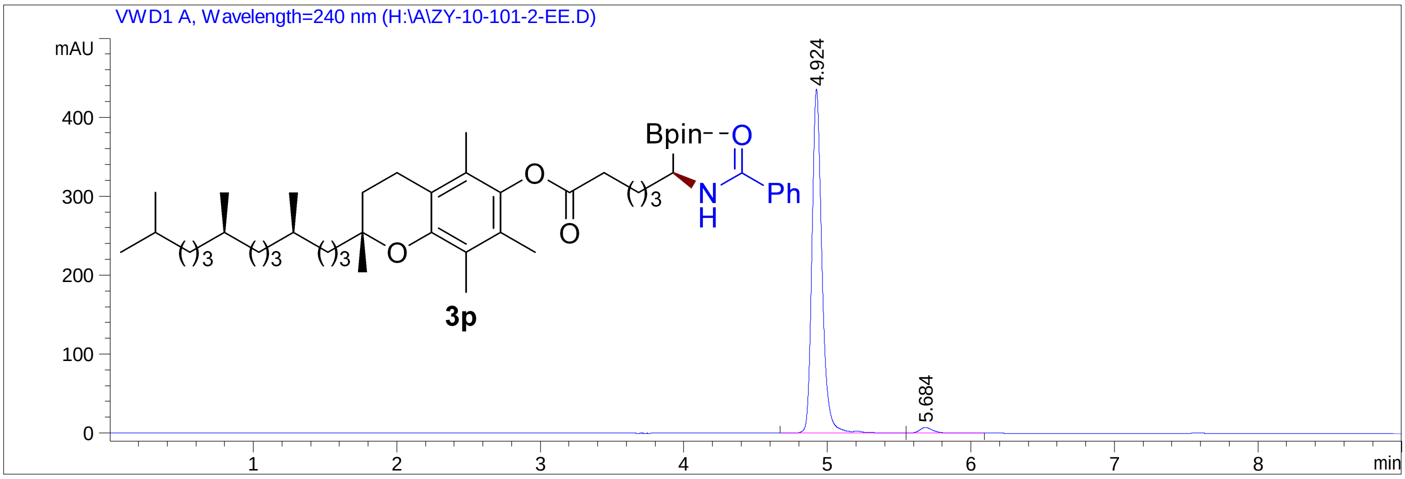

===

Area Percent Report

===

Sorted By : Signal
Multiplier : 1.0000
Dilution : 1.0000
Do not use Multiplier & Dilution Factor with ISTDs

Signal 1: VWD1 A, Wavelength=240 nm

Peak #	RetTime [min]	Type	Width [min]	Area [mAU*s]	Height [mAU]	Area %
1	4.924	BV R	0.0737	2114.95093	435.55344	97.8851
2	5.684	BB	0.0982	45.69511	7.04339	2.1149

Totals : 2160.64604 442.59682

===

*** End of Report ***

Supplementary Figure 223: HPLC spectrum of **3p**.

Data File H:\A\ZY-10-101-6-EE.D
Sample Name: ZY-10-101-6-EE

===
Acq. Operator   : 系统   Seq. Line : 32
Acq. Instrument : HPLC-1260   Location :   63
Injection Date : 7/5/2022 1:09:09 AM   Inj :   1
                                               Inj Volume : 3.000 µl
Different Inj Volume from Sample Entry! Actual Inj Volume : 0.300 µl
Acq. Method   : D:\zy\20220624\YH 2022-07-04 14-04-41\10EtOH15_10-1-6-240.M
Last changed   : 7/4/2022 7:42:02 PM by 系统
Analysis Method : E:\DATA\20220317\LC 2022-08-08 20-30-16\2IPA-50-0.8-3-210-JXL.M (Sequence Method)
Last changed   : 8/9/2022 9:09:12 AM by SYSTEM
                  (modified after loading)
Additional Info : Peak(s) manually integrated

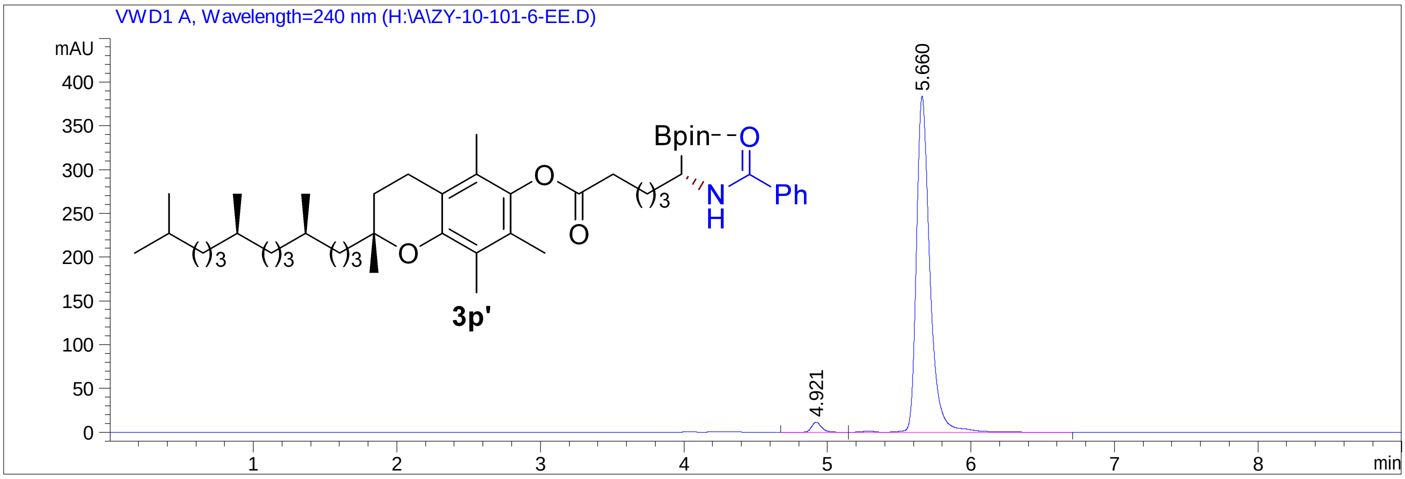

===
Area Percent Report
===

Sorted By   :   Signal
Multiplier   :   1.0000
Dilution   :   1.0000
Do not use Multiplier & Dilution Factor with ISTDs

Signal 1: VWD1 A, Wavelength=240 nm

Peak #	RetTime [min]	Type	Width [min]	Area [mAU*s]	Height [mAU]	Area %
1	4.921	BB	0.0754	58.30293	11.65547	2.2584
2	5.660	VB R	0.0991	2523.31274	384.16348	97.7416

Totals :   2581.61567   395.81895

===
*** End of Report ***

Supplementary Figure 224: HPLC spectrum of **3p'**.

Data File F:\ZY\AMIDE\ORIGINAL DATA\ZY-09-161-3-RAC.D
Sample Name: ZY-09-161-3-RAC

===

Acq. Operator : 系统 Seq. Line : 4
Acq. Instrument : HPLC-1260 Location : 71
Injection Date : 11/23/2021 9:59:34 AM Inj : 1
Inj Volume : 3.000 µl
Different Inj Volume from Sample Entry! Actual Inj Volume : 0.400 µl
Acq. Method : D:\zy\20211119\YH 2021-11-23 09-08-50\5EtOH15_10-1-2-254.M
Last changed : 11/23/2021 10:14:40 AM by 系统
(modified after loading)
Analysis Method : E:\DATA\20220316\LC 2022-03-16 22-19-08\10IPA-10-1.0-1.MZWJ.M (Sequence Method)
Last changed : 3/28/2022 11:08:25 PM by SYSTEM
(modified after loading)
Additional Info : Peak(s) manually integrated

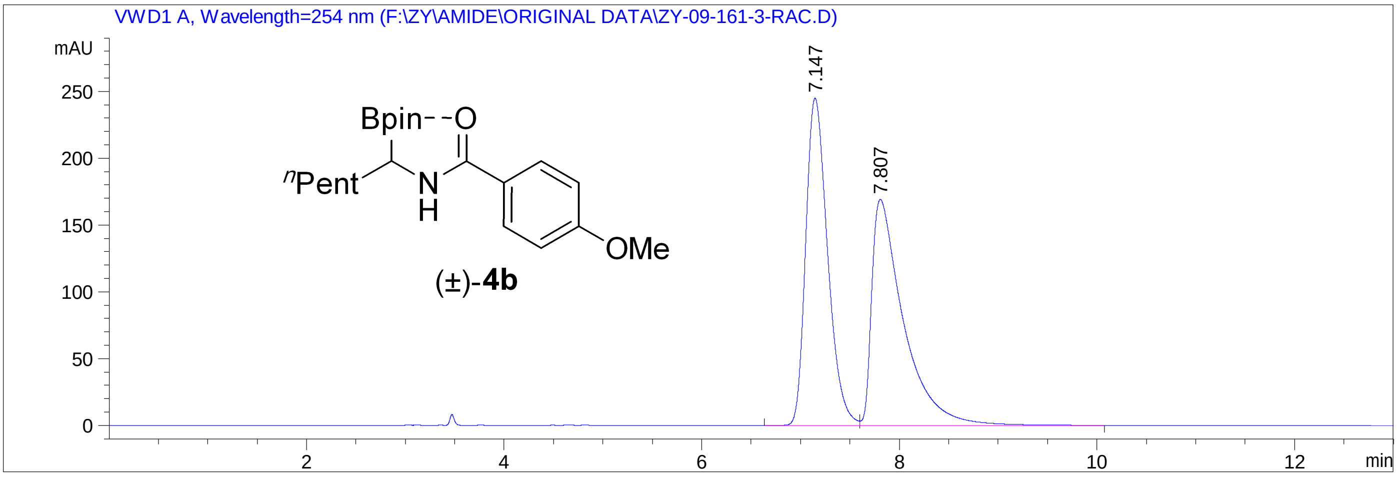

===

Area Percent Report

===

Sorted By : Signal
Multiplier : 1.0000
Dilution : 1.0000
Do not use Multiplier & Dilution Factor with ISTDs

Signal 1: VWD1 A, Wavelength=254 nm

Peak #	RetTime [min]	Type	Width [min]	Area [mAU*s]	Height [mAU]	Area %
1	7.147	BV	0.2367	3775.47705	245.07373	50.0009
2	7.807	VB	0.3262	3775.33398	169.27411	49.9991
Totals :				7550.81104	414.34784	

===

*** End of Report ***

Supplementary Figure 225: HPLC spectrum of (±)-**4b**.

Data File F:\ZY\AMIDE\ORIGINAL DATA\ZY-09-161-3-EE.D
Sample Name: ZY-09-161-3-EE

===

Acq. Operator : 系统 Seq. Line : 5
Acq. Instrument : HPLC-1260 Location : 72
Injection Date : 11/23/2021 10:16:12 AM Inj : 1
Inj Volume : 3.000 µl
Different Inj Volume from Sample Entry! Actual Inj Volume : 0.300 µl
Acq. Method : D:\zy\20211119\YH 2021-11-23 09-08-50\5EtOH15_10-1-2-254.M
Last changed : 11/23/2021 10:14:40 AM by 系统
Analysis Method : E:\DATA\20220316\LC 2022-03-16 22-19-08\10IPA-10-1.0-1.MZWJ.M (Sequence Method)
Last changed : 3/28/2022 11:07:48 PM by SYSTEM
(modified after loading)

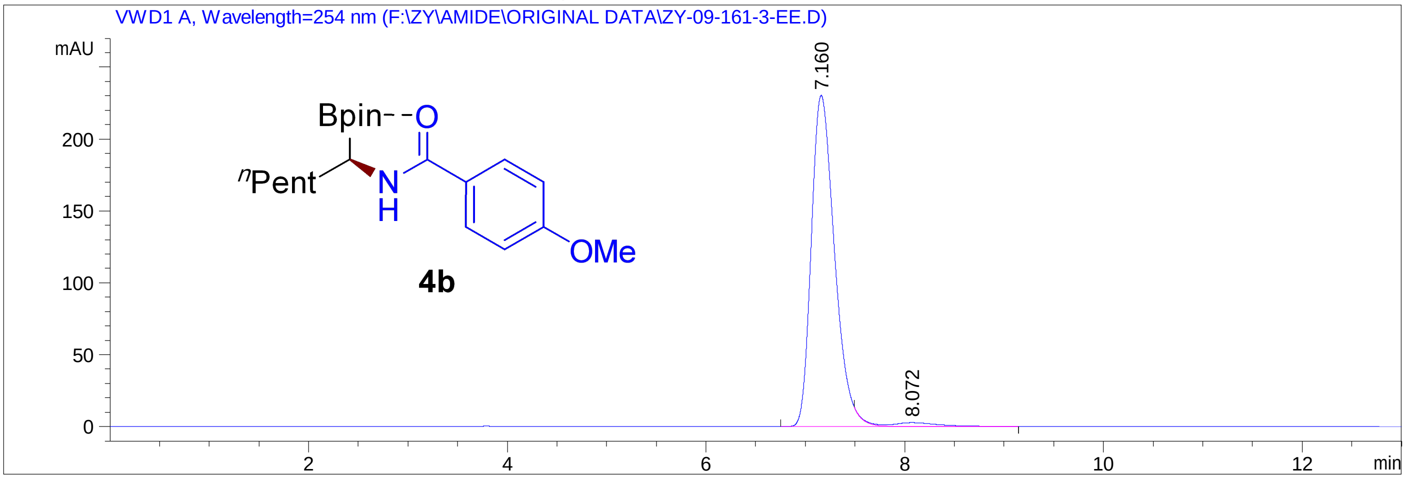

===

Area Percent Report

===

Sorted By : Signal
Multiplier : 1.0000
Dilution : 1.0000
Do not use Multiplier & Dilution Factor with ISTDs

Signal 1: VWD1 A, Wavelength=254 nm

Peak #	RetTime [min]	Type	Width [min]	Area [mAU*s]	Height [mAU]	Area %
1	7.160	BV R	0.2495	3752.35693	230.38501	97.6166
2	8.072	VB E	0.4021	91.61741	2.72957	2.3834

Totals : 3843.97434 233.11458

===

*** End of Report ***

Supplementary Figure 226: HPLC spectrum of **4b**.

Data File H:\1\ADD\ZY-09-189-2-RAC.D
Sample Name: ZY-09-189-2-RAC

===

Acq. Operator : 系统 Seq. Line : 22
Acq. Instrument : HPLC-1260 Location : 77
Injection Date : 8/20/2022 2:05:52 PM Inj : 1
Inj Volume : 3.000 µl
Different Inj Volume from Sample Entry! Actual Inj Volume : 1.000 µl
Acq. Method : D:\zy\20220803\YH 2022-08-20 08-41-06\5EtOH15_10-1-6-220.M
Last changed : 8/20/2022 2:00:54 PM by 系统
Analysis Method : C:\Chem32\1\Data\LC 2020-08-14 14-31-12\test2.M (Sequence Method)
Last changed : 8/23/2022 3:25:25 PM by SYSTEM
(modified after loading)
Additional Info : Peak(s) manually integrated

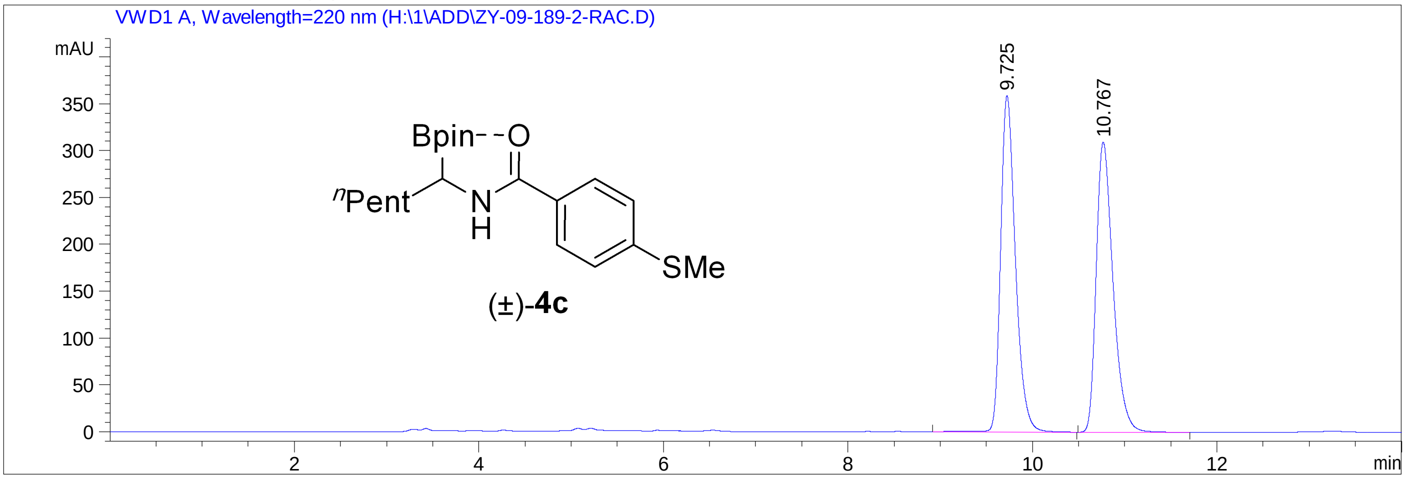

===

Area Percent Report

===

Sorted By : Signal
Multiplier : 1.0000
Dilution : 1.0000
Do not use Multiplier & Dilution Factor with ISTDs

Signal 1: VWD1 A, Wavelength=220 nm

Peak #	RetTime [min]	Type	Width [min]	Area [mAU*s]	Height [mAU]	Area %
1	9.725	VB R	0.1710	4004.30542	359.31268	50.4995
2	10.767	BB	0.1953	3925.08423	309.83737	49.5005

Totals : 7929.38965 669.15005

===

*** End of Report ***

Supplementary Figure 227: HPLC spectrum of **(±)-4c**.

Data File H:\1\ZY-09-189-2-EE.D
Sample Name: ZY-09-189-2-EE

===

Acq. Operator : 系统 Seq. Line : 19
Acq. Instrument : HPLC-1260 Location : 79
Injection Date : 8/20/2022 1:19:09 PM Inj : 1
Inj Volume : 3.000 µl
Different Inj Volume from Sample Entry! Actual Inj Volume : 0.600 µl
Acq. Method : D:\zy\20220803\YH 2022-08-20 08-41-06\5EtOH15_10-1-6-220.M
Last changed : 8/20/2022 9:59:08 AM by 系统
Analysis Method : E:\DATA\20220317\LC 2022-08-22 08-37-45\5IPA-40-1.0-1-220-QDY.M (Sequence Method)
Last changed : 8/22/2022 7:04:29 PM by SYSTEM
(modified after loading)
Additional Info : Peak(s) manually integrated

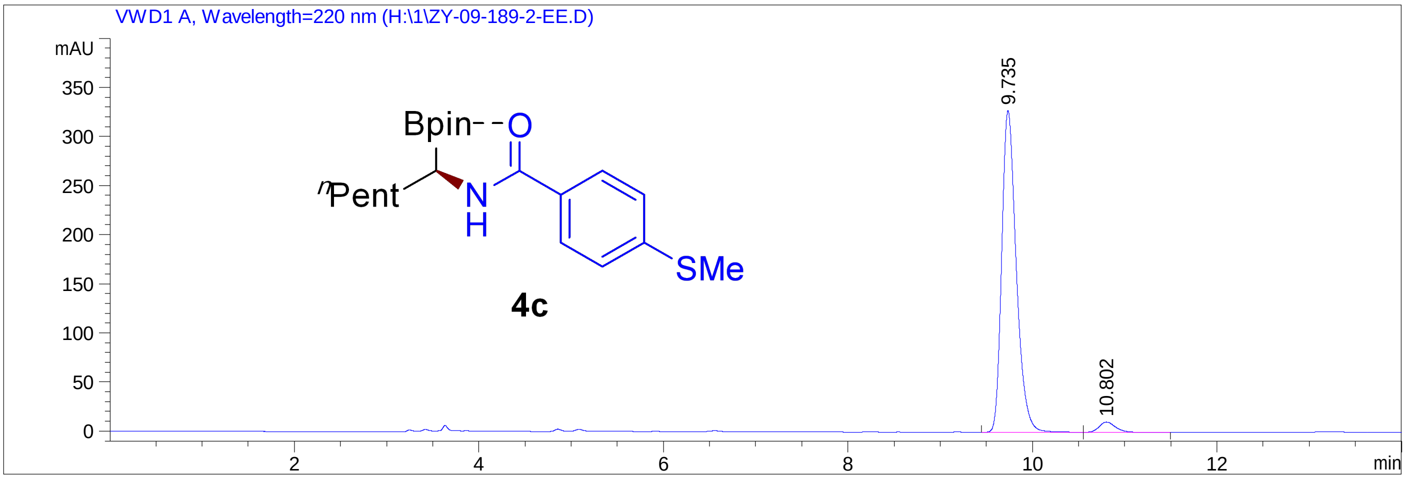

===

Area Percent Report

===

Sorted By : Signal
Multiplier : 1.0000
Dilution : 1.0000
Do not use Multiplier & Dilution Factor with ISTDs

Signal 1: VWD1 A, Wavelength=220 nm

Peak #	RetTime [min]	Type	Width [min]	Area [mAU*s]	Height [mAU]	Area %
1	9.735	BV	0.1678	3588.63062	327.61975	96.4551
2	10.802	VB	0.1891	131.88794	10.52918	3.5449

Totals : 3720.51855 338.14893

===

*** End of Report ***

Supplementary Figure 228: HPLC spectrum of **4c**.

Data File H:\ZY-09-189-6-RAC.D
Sample Name: ZY-09-189-6-RAC

===

Acq. Operator : 系统 Seq. Line : 31
Acq. Instrument : HPLC-1260 Location : 44
Injection Date : 12/21/2021 10:14:13 PM Inj : 1
Inj Volume : 3.000 µl
Different Inj Volume from Sample Entry! Actual Inj Volume : 0.300 µl
Acq. Method : D:\zy\20211219\YH 2021-12-21 13-59-57\5EtOH20_10-1-2-220.M
Last changed : 12/21/2021 9:45:37 PM by 系统
Analysis Method : E:\DATA\20220317\LC 2022-03-29 18-02-36\1.0IPA_15_0.8_1-254-SFT.M (Sequence Method)
Last changed : 3/29/2022 8:47:13 PM by SYSTEM
(modified after loading)
Additional Info : Peak(s) manually integrated

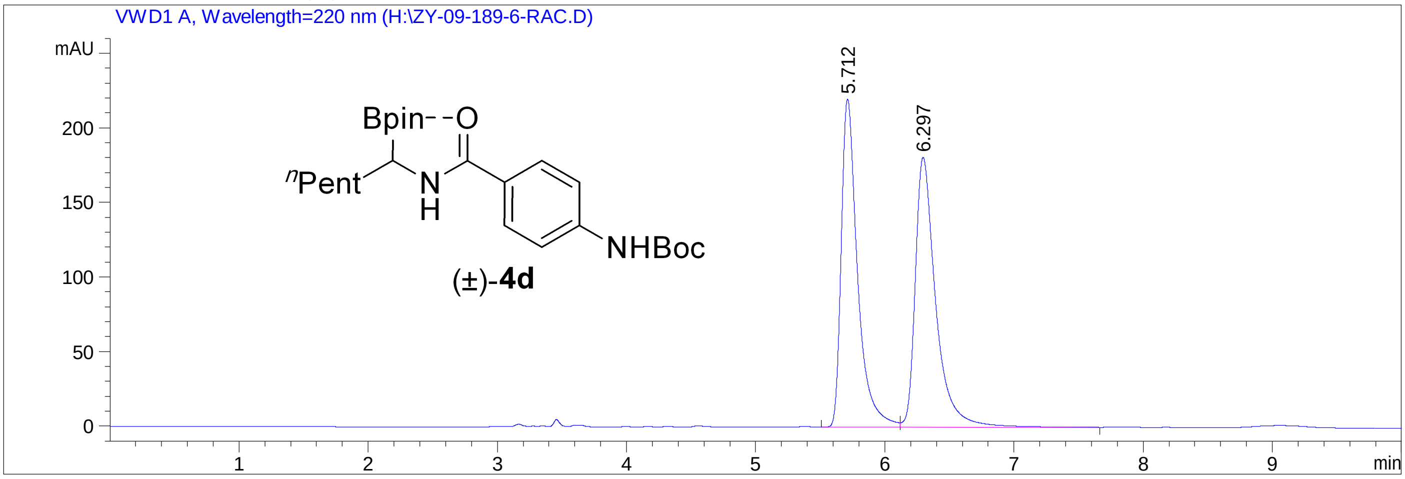

===
Area Percent Report
===

Sorted By : Signal
Multiplier : 1.0000
Dilution : 1.0000
Do not use Multiplier & Dilution Factor with ISTDs

Signal 1: VWD1 A, Wavelength=220 nm

Peak #	RetTime [min]	Type	Width [min]	Area [mAU*s]	Height [mAU]	Area %
1	5.712	BV	0.1316	1935.34534	220.14615	49.1952
2	6.297	VB	0.1642	1998.66370	181.10876	50.8048

Totals : 3934.00903 401.25491

===
*** End of Report ***

Supplementary Figure 229: HPLC spectrum of (±)-**4d**.

Data File H:\ZY-09-189-6-EE.D
Sample Name: ZY-09-189-6-EE

===
Acq. Operator : 系统 Seq. Line : 30
Acq. Instrument : HPLC-1260 Location : 43
Injection Date : 12/21/2021 9:57:49 PM Inj : 1
Inj Volume : 3.000 µl
Different Inj Volume from Sample Entry! Actual Inj Volume : 0.500 µl
Acq. Method : D:\zy\20211219\YH 2021-12-21 13-59-57\5EtOH20_10-1-2-220.M
Last changed : 12/21/2021 9:45:37 PM by 系统
Analysis Method : E:\DATA\20220317\LC 2022-03-29 18-02-36\1.0IPA_15_0.8_1-254-SFT.M (Sequence Method)
Last changed : 3/29/2022 8:46:33 PM by SYSTEM
(modified after loading)
Additional Info : Peak(s) manually integrated

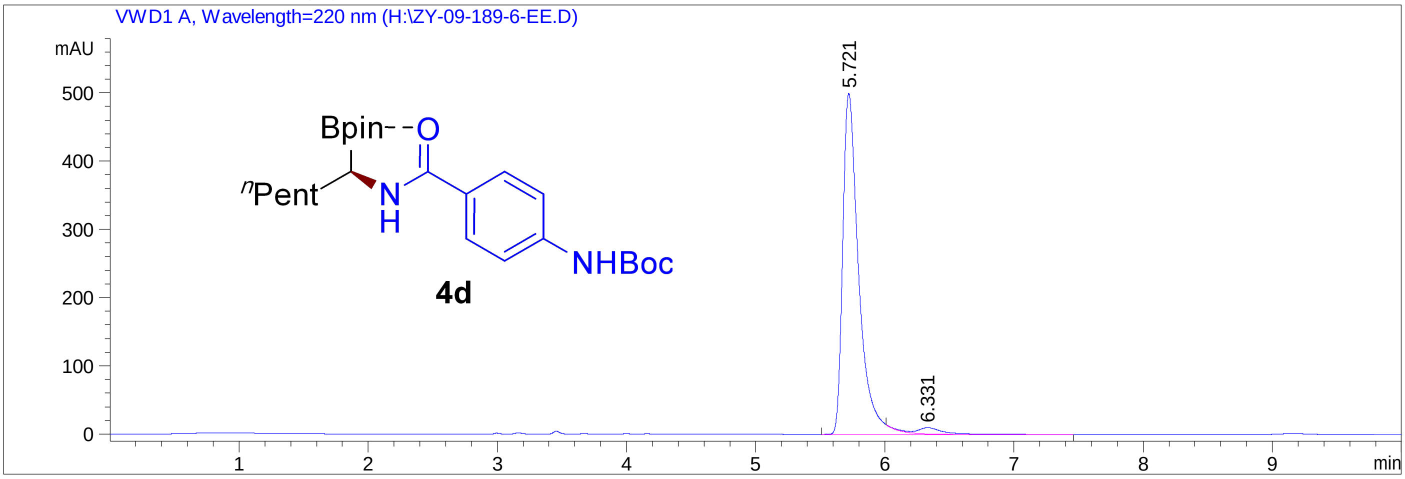

===
Area Percent Report
===

Sorted By : Signal
Multiplier : 1.0000
Dilution : 1.0000
Do not use Multiplier & Dilution Factor with ISTDs

Signal 1: VWD1 A, Wavelength=220 nm

Peak #	RetTime [min]	Type	Width [min]	Area [mAU*s]	Height [mAU]	Area %
1	5.721	BV R	0.1284	4325.95020	499.95084	96.8165
2	6.331	VB E	0.2160	142.24498	9.32273	3.1835

Totals : 4468.19518 509.27357

===
*** End of Report ***

Supplementary Figure 230: HPLC spectrum of **4d**.

Data File H:\DATA2\ZY-10-69-6-RAC.D
Sample Name: ZY-10-69-6-RAC

===

Acq. Operator : 系统 Seq. Line : 3
Acq. Instrument : HPLC-1260 Location : 41
Injection Date : 5/29/2022 8:39:19 PM Inj : 1
Inj Volume : 3.000 µl
Different Inj Volume from Sample Entry! Actual Inj Volume : 0.500 µl
Acq. Method : D:\zy\20220328\YH 2022-05-29 20-09-39\5EtOH15_10-1-2-240.M
Last changed : 5/29/2022 8:09:40 PM by 系统
Analysis Method : E:\DATA\20220317\LC 2022-06-04 08-49-06\0.3IPA-0.3-60-220-1-CCP.M (Sequence Method)
Last changed : 6/7/2022 10:14:44 AM by SYSTEM
(modified after loading)
Additional Info : Peak(s) manually integrated

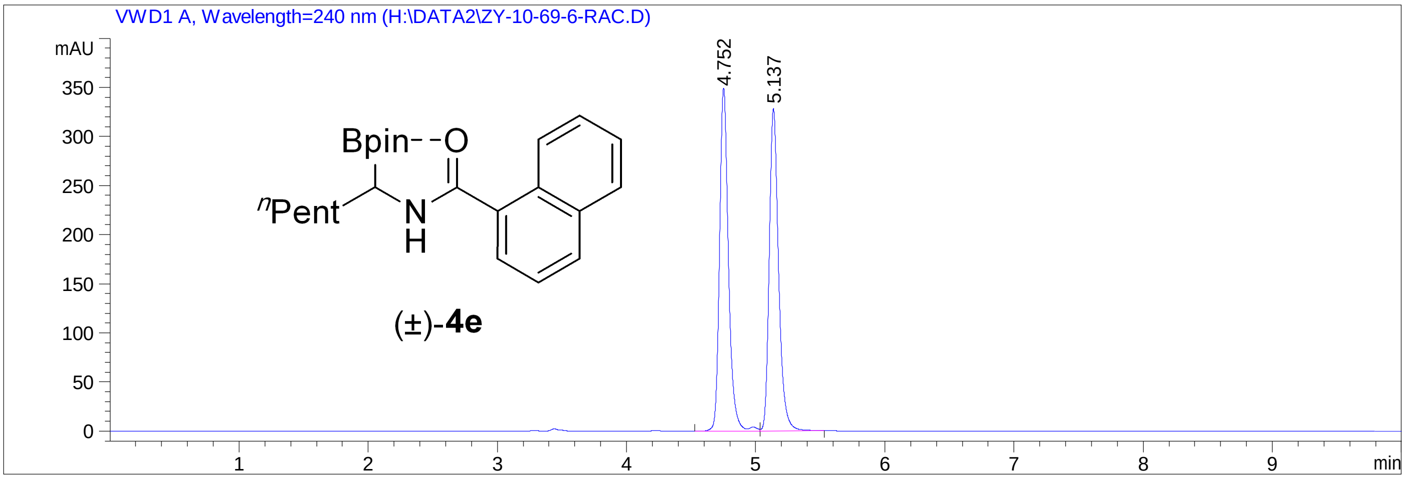

===

Area Percent Report

===

Sorted By : Signal
Multiplier : 1.0000
Dilution : 1.0000
Do not use Multiplier & Dilution Factor with ISTDs

Signal 1: VWD1 A, Wavelength=240 nm

Peak #	RetTime [min]	Type	Width [min]	Area [mAU*s]	Height [mAU]	Area %
1	4.752	BV R	0.0720	1679.71350	349.52957	50.4898
2	5.137	VB	0.0766	1647.12341	328.13077	49.5102

Totals : 3326.83691 677.66034

===

*** End of Report ***

Supplementary Figure 231: HPLC spectrum of (±)-**4e**.

Data File H:\DATA2\ZY-10-69-6-EE.D
Sample Name: ZY-10-69-6-EE

===
Acq. Operator : 系统 Seq. Line : 4
Acq. Instrument : HPLC-1260 Location : 42
Injection Date : 5/29/2022 8:55:45 PM Inj : 1
Inj Volume : 3.000 µl
Different Inj Volume from Sample Entry! Actual Inj Volume : 0.100 µl
Acq. Method : D:\zy\20220328\YH 2022-05-29 20-09-39\5EtOH15_10-1-2-240.M
Last changed : 5/29/2022 8:09:40 PM by 系统
Analysis Method : E:\DATA\20220317\LC 2022-06-04 08-49-06\0.3IPA-0.3-60-220-1-CCP.M (Sequence Method)
Last changed : 6/7/2022 10:13:51 AM by SYSTEM
(modified after loading)

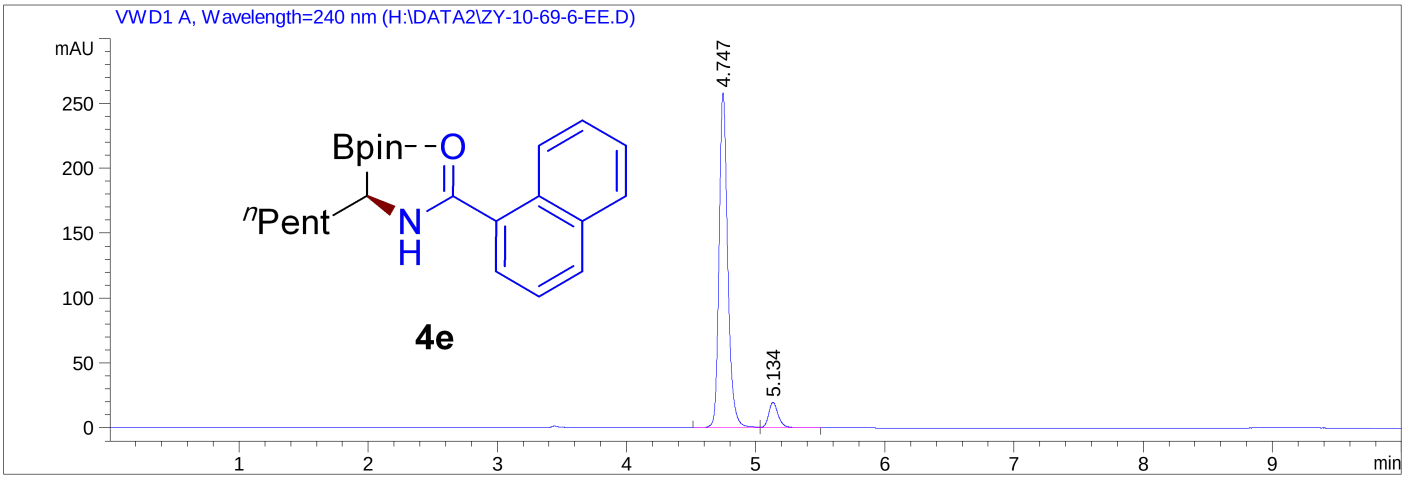

===
Area Percent Report
===

Sorted By : Signal
Multiplier : 1.0000
Dilution : 1.0000
Do not use Multiplier & Dilution Factor with ISTDs

Signal 1: VWD1 A, Wavelength=240 nm

Peak #	RetTime [min]	Type	Width [min]	Area [mAU*s]	Height [mAU]	Area %
1	4.747	BV	0.0710	1203.43774	257.80865	92.1441
2	5.134	VB	0.0789	102.60114	19.82286	7.8559

Totals : 1306.03889 277.63151

===
*** End of Report ***

Supplementary Figure 232: HPLC spectrum of **4e**.

Data File E:\DATA\20210920\LC 2021-11-30 09-32-52\OnlineEdited--053.D
Sample Name: ZY-09-164-5-RAC

===

Acq. Operator : SYSTEM Seq. Line : 53
Acq. Instrument : HPLC1260 Location : P1-E4
Injection Date : 12/1/2021 9:28:54 AM Inj : 1
Inj Volume : 3.000 µl
Different Inj Volume from Sample Entry! Actual Inj Volume : 0.300 µl
Acq. Method : E:\DATA\20210920\LC 2021-11-30 09-32-52\3IPA_20_0.4_3-254.M
Last changed : 12/1/2021 9:25:40 AM by SYSTEM
Analysis Method : E:\DATA\20210920\LC 2021-11-30 09-32-52\3IPA_20_0.4_3-254.M (Sequence Method)
Last changed : 12/1/2021 12:28:00 PM by SYSTEM
(modified after loading)
Additional Info : Peak(s) manually integrated

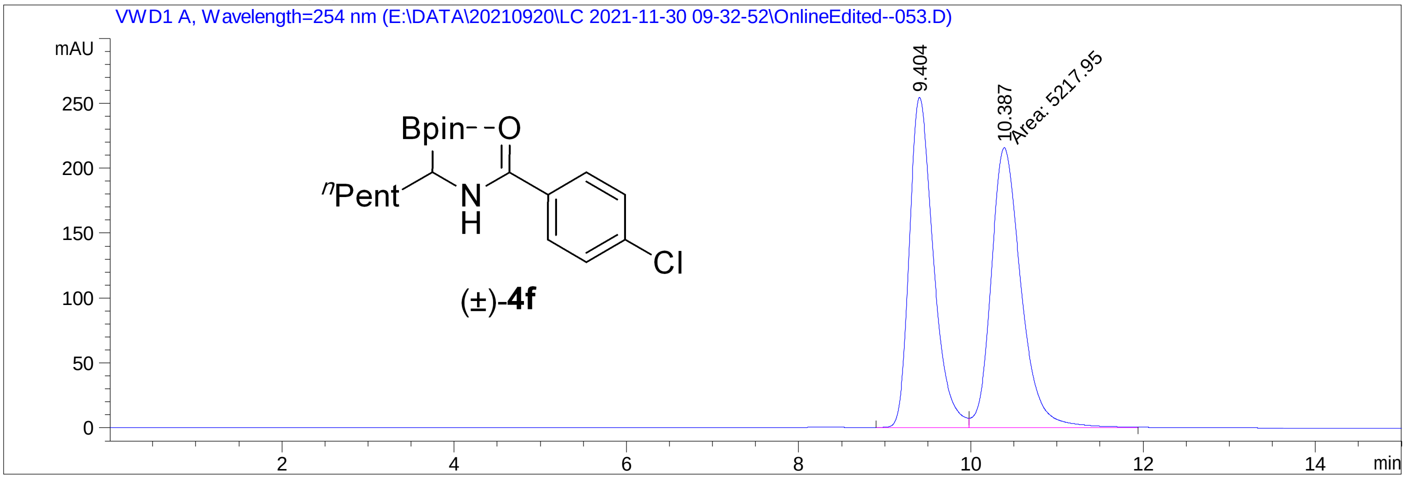

===

Area Percent Report

===

Sorted By : Signal
Multiplier : 1.0000
Dilution : 1.0000
Do not use Multiplier & Dilution Factor with ISTDs

Signal 1: VWD1 A, Wavelength=254 nm

Peak #	RetTime [min]	Type	Width [min]	Area [mAU*s]	Height [mAU]	Area %
1	9.404	BV	0.2968	4941.78369	254.15439	48.6409
2	10.387	MF	0.4034	5217.95313	215.57483	51.3591

Totals : 1.01597e4 469.72922

===

*** End of Report ***

Supplementary Figure 233: HPLC spectrum of (±)-**4f**.

Data File E:\DATA\20210920\LC 2021-11-30 09-32-52\OnlineEdited--051.D
Sample Name: ZY-09-164-5-EE

===

Acq. Operator : SYSTEM   Seq. Line : 51
Acq. Instrument : HPLC1260   Location : P1-E3
Injection Date : 12/1/2021 8:47:09 AM   Inj : 1
                                                    Inj Volume : 3.000 µl
Different Inj Volume from Sample Entry! Actual Inj Volume : 0.500 µl
Acq. Method : E:\DATA\20210920\LC 2021-11-30 09-32-52\3IPA_20_0.4_3-254.M
Last changed : 11/30/2021 11:50:22 PM by SYSTEM
Analysis Method : E:\DATA\20210920\LC 2021-11-30 09-32-52\3IPA_20_0.4_3-254.M (Sequence Method)
Last changed : 12/1/2021 12:28:54 PM by SYSTEM
(modified after loading)

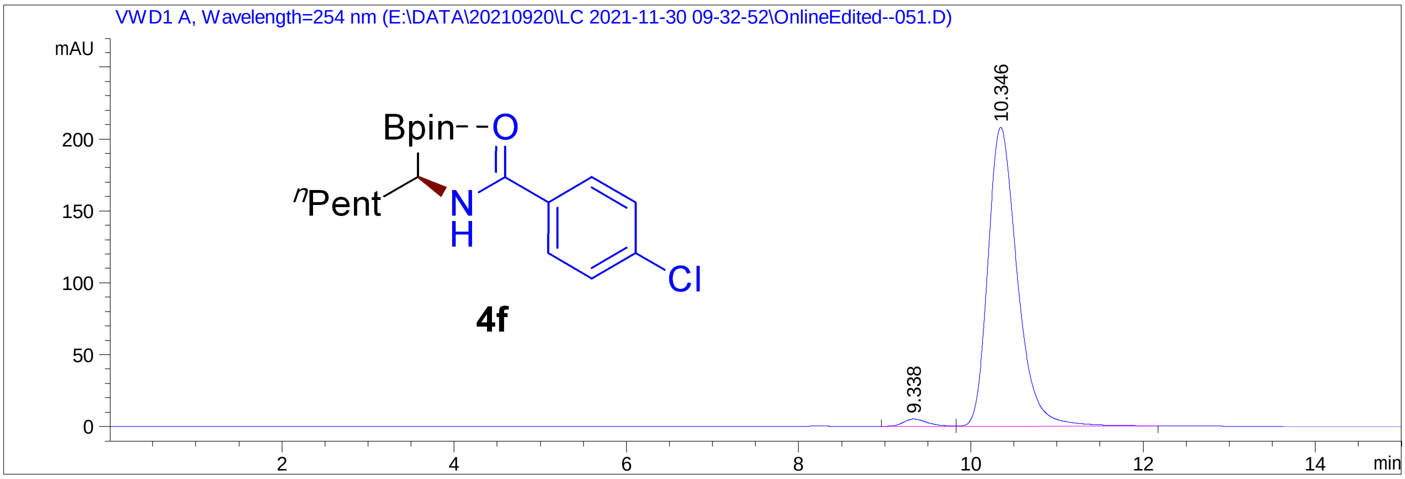

===

Area Percent Report

===

Sorted By : Signal
Multiplier : 1.0000
Dilution : 1.0000
Do not use Multiplier & Dilution Factor with ISTDs

Signal 1: VWD1 A, Wavelength=254 nm

Peak #	RetTime [min]	Type	Width [min]	Area [mAU*s]	Height [mAU]	Area %
1	9.338	BV	0.2937	96.96291	5.03491	1.9338
2	10.346	VB	0.3610	4917.21289	207.83859	98.0662

Totals : 5014.17580 212.87350

===

*** End of Report ***

Supplementary Figure 234: HPLC spectrum of **4f**.

Data File F:\ZY\AMIDE\ORIGINAL DATA\ZY-09-161-8-RAC.D
Sample Name: ZY-09-161-8-RAC

===

Acq. Operator : 系统 Seq. Line : 7
Acq. Instrument : HPLC-1260 Location : 21
Injection Date : 11/24/2021 12:11:30 AM Inj : 1
Inj Volume : 3.000 µl
Different Inj Volume from Sample Entry! Actual Inj Volume : 1.500 µl
Acq. Method : D:\zy\20211119\YH 2021-11-23 21-47-26\3IPA-30-0.8-1-2-254.M
Last changed : 11/23/2021 9:47:27 PM by 系统
Analysis Method : E:\DATA\20220316\LC 2022-03-16 22-19-08\10IPA-10-1.0-1.MZWJ.M (Sequence Method)
Last changed : 3/28/2022 11:10:10 PM by SYSTEM
(modified after loading)
Additional Info : Peak(s) manually integrated

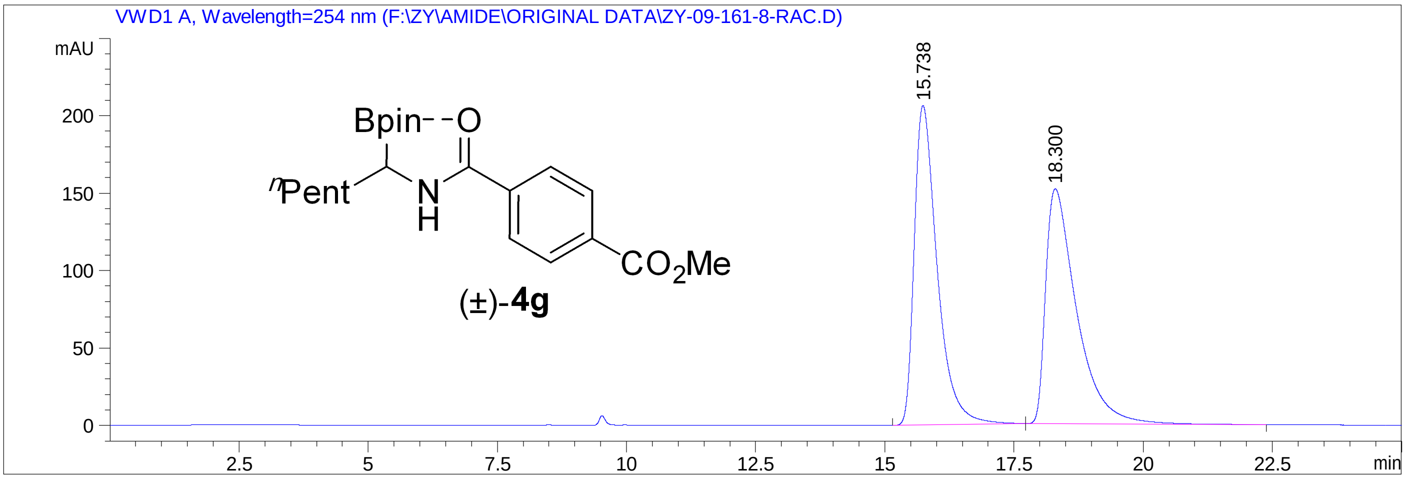

===

Area Percent Report

===

Sorted By : Signal
Multiplier : 1.0000
Dilution : 1.0000
Do not use Multiplier & Dilution Factor with ISTDs

Signal 1: VWD1 A, Wavelength=254 nm

Peak #	RetTime [min]	Type	Width [min]	Area [mAU*s]	Height [mAU]	Area %
1	15.738	BB	0.4680	6377.62256	206.38446	50.1471
2	18.300	BB	0.6098	6340.20410	151.79956	49.8529
Totals :				1.27178e4	358.18402	

===

*** End of Report ***

Supplementary Figure 235: HPLC spectrum of (±)-**4g**.

Data File F:\ZY\AMIDE\ORIGINAL DATA\ZY-09-161-8-EE.D
Sample Name: ZY-09-161-8-EE

===

Acq. Operator : 系统 Seq. Line : 6
Acq. Instrument : HPLC-1260 Location : 22
Injection Date : 11/23/2021 11:40:07 PM Inj : 1
Inj Volume : 3.000 µl
Different Inj Volume from Sample Entry! Actual Inj Volume : 0.700 µl
Acq. Method : D:\zy\20211119\YH 2021-11-23 21-47-26\3IPA-30-0.8-1-2-254.M
Last changed : 11/23/2021 9:47:27 PM by 系统
Analysis Method : E:\DATA\20220316\LC 2022-03-16 22-19-08\10IPA-10-1.0-1.MZWJ.M (Sequence Method)
Last changed : 3/28/2022 11:09:37 PM by SYSTEM
(modified after loading)

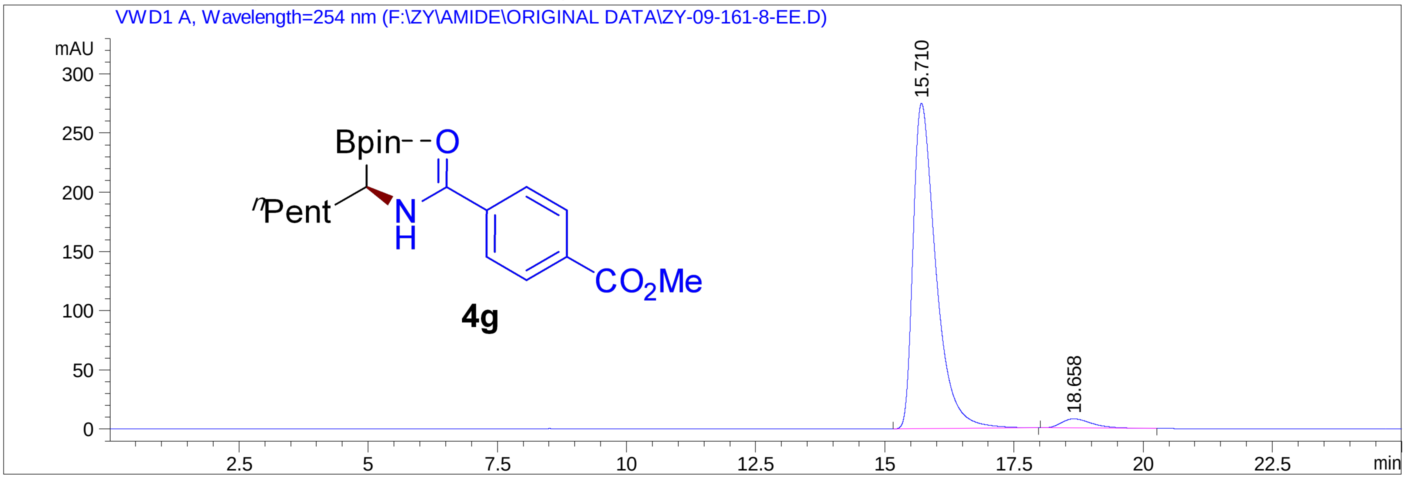

===

Area Percent Report

===

Sorted By : Signal
Multiplier : 1.0000
Dilution : 1.0000
Do not use Multiplier & Dilution Factor with ISTDs

Signal 1: VWD1 A, Wavelength=254 nm

Peak #	RetTime [min]	Type	Width [min]	Area [mAU*s]	Height [mAU]	Area %
1	15.710	BB	0.4667	8486.95020	274.90009	96.3010
2	18.658	BB	0.5090	325.99210	7.82603	3.6990

Totals : 8812.94229 282.72612

===

*** End of Report ***

Supplementary Figure 236: HPLC spectrum of **4g**.

Data File E:\DATA\20210920\LC 2021-11-30 09-32-52\OnlineEdited--056.D
Sample Name: ZY-09-165-2-RAC

===

Acq. Operator : SYSTEM Seq. Line : 56
Acq. Instrument : HPLC1260 Location : P1-E2
Injection Date : 12/1/2021 10:26:45 AM Inj : 1
Inj Volume : 3.000 µl
Different Inj Volume from Sample Entry! Actual Inj Volume : 1.600 µl
Acq. Method : E:\DATA\20210920\LC 2021-11-30 09-32-52\2IPA_20_1.0_2-254.M
Last changed : 12/1/2021 9:31:40 AM by SYSTEM
Analysis Method : E:\DATA\20210920\LC 2021-11-30 09-32-52\2IPA_20_1.0_2-254.M (Sequence Method)
Last changed : 12/1/2021 12:30:19 PM by SYSTEM
(modified after loading)
Additional Info : Peak(s) manually integrated

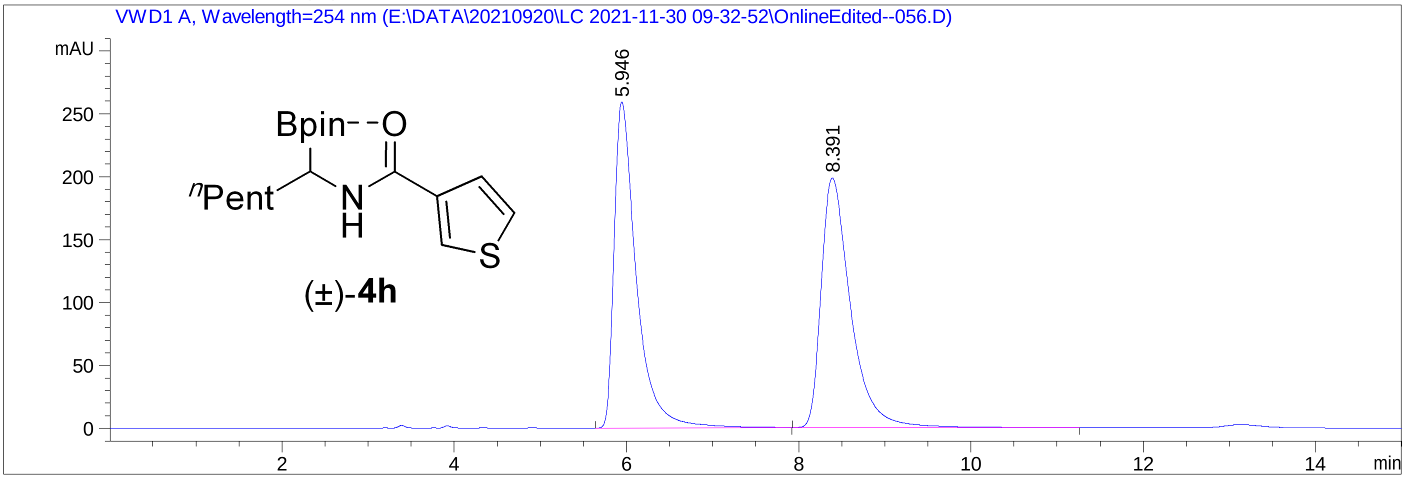

===

Area Percent Report

===

Sorted By : Signal
Multiplier : 1.0000
Dilution : 1.0000
Do not use Multiplier & Dilution Factor with ISTDs

Signal 1: VWD1 A, Wavelength=254 nm

Peak #	RetTime [min]	Type	Width [min]	Area [mAU*s]	Height [mAU]	Area %
1	5.946	BB	0.2665	4652.44141	259.13269	49.6479
2	8.391	BB	0.3575	4718.43799	198.40140	50.3521

Totals : 9370.87939 457.53409

===

*** End of Report ***

Supplementary Figure 237: HPLC spectrum of (±)-**4h**.

Data File E:\DATA\20210920\LC 2021-11-30 09-32-52\OnlineEdited--055.D
Sample Name: ZY-09-165-2-EE

===

Acq. Operator : SYSTEM Seq. Line : 55
Acq. Instrument : HPLC1260 Location : P1-E1
Injection Date : 12/1/2021 10:05:47 AM Inj : 1
Inj Volume : 3.000 µl
Different Inj Volume from Sample Entry! Actual Inj Volume : 1.500 µl
Acq. Method : E:\DATA\20210920\LC 2021-11-30 09-32-52\2IPA_20_1.0_2-254.M
Last changed : 12/1/2021 9:31:40 AM by SYSTEM
Analysis Method : E:\DATA\20210920\LC 2021-11-30 09-32-52\2IPA_20_1.0_2-254.M (Sequence Method)
Last changed : 12/1/2021 12:30:52 PM by SYSTEM
(modified after loading)

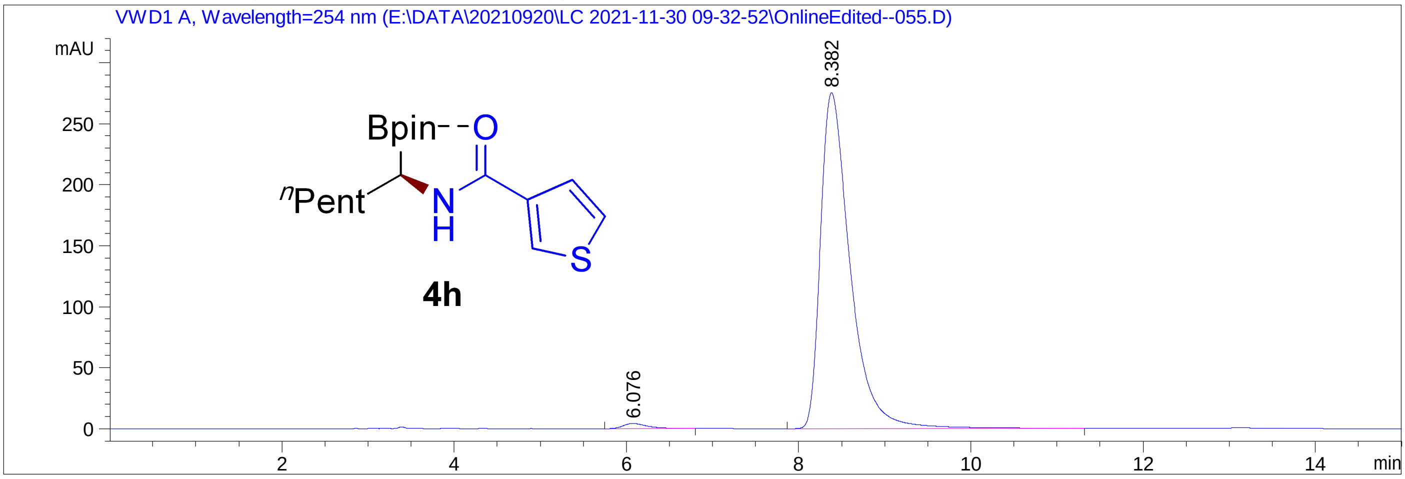

===

Area Percent Report

===

Sorted By : Signal
Multiplier : 1.0000
Dilution : 1.0000
Do not use Multiplier & Dilution Factor with ISTDs

Signal 1: VWD1 A, Wavelength=254 nm

Peak #	RetTime [min]	Type	Width [min]	Area [mAU*s]	Height [mAU]	Area %
1	6.076	BB	0.2913	81.13868	4.18332	1.2148
2	8.382	BB	0.3615	6597.98486	275.45410	98.7852

Totals : 6679.12354 279.63742

===

*** End of Report ***

Supplementary Figure 238: HPLC spectrum of **4h**.

Data File E:\DATA\20211222\LC 2021-12-22 14-06-06\1CB-0401.D
Sample Name: ZY-09-167-6-RAC

===

Acq. Operator : SYSTEM Seq. Line : 4
Acq. Instrument : HPLC1260 Location : P1-C2
Injection Date : 12/22/2021 2:55:43 PM Inj : 1
Inj Volume : 3.000 µl
Different Inj Volume from Sample Entry! Actual Inj Volume : 0.500 µl
Acq. Method : E:\DATA\20211222\LC 2021-12-22 14-06-06\5IPA15_10_3-240.M
Last changed : 12/22/2021 2:06:06 PM by SYSTEM
Analysis Method : E:\DATA\20211222\LC 2021-12-22 14-06-06\5IPA15_10_3-240.M (Sequence Method)
Last changed : 12/22/2021 3:19:21 PM by SYSTEM
(modified after loading)
Additional Info : Peak(s) manually integrated

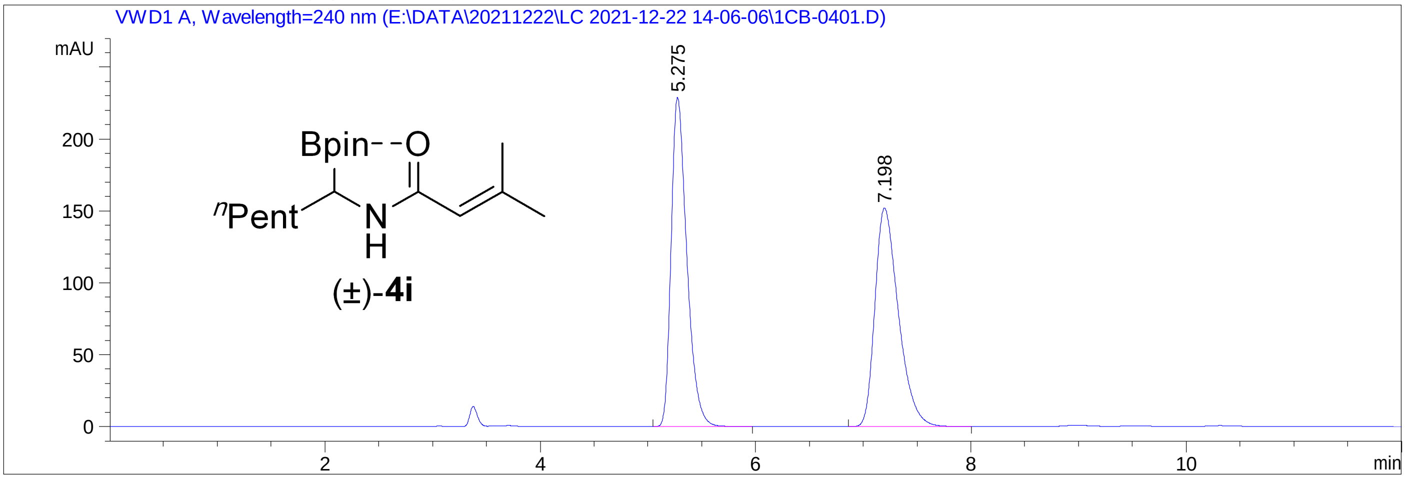

===

Area Percent Report

===

Sorted By : Signal
Multiplier : 1.0000
Dilution : 1.0000
Do not use Multiplier & Dilution Factor with ISTDs

Signal 1: VWD1 A, Wavelength=240 nm

Peak #	RetTime [min]	Type	Width [min]	Area [mAU*s]	Height [mAU]	Area %
1	5.275	BB	0.1542	2320.10425	228.95290	50.1665
2	7.198	BB	0.2309	2304.70605	152.02585	49.8335

Totals : 4624.81030 380.97874

===

*** End of Report ***

Supplementary Figure 239: HPLC spectrum of (±)-**4i**.

Data File E:\DATA\20211222\LC 2021-12-22 14-06-06\1CA-0301.D
Sample Name: ZY-09-167-6-EE

===

Acq. Operator : SYSTEM Seq. Line : 3
Acq. Instrument : HPLC1260 Location : P1-C1
Injection Date : 12/22/2021 2:39:59 PM Inj : 1
Inj Volume : 3.000 µl
Different Inj Volume from Sample Entry! Actual Inj Volume : 0.500 µl
Acq. Method : E:\DATA\20211222\LC 2021-12-22 14-06-06\5IPA15_10_3-240.M
Last changed : 12/22/2021 2:06:06 PM by SYSTEM
Analysis Method : E:\DATA\20211222\LC 2021-12-22 14-06-06\5IPA15_10_3-240.M (Sequence Method)
Last changed : 12/22/2021 3:19:21 PM by SYSTEM
(modified after loading)

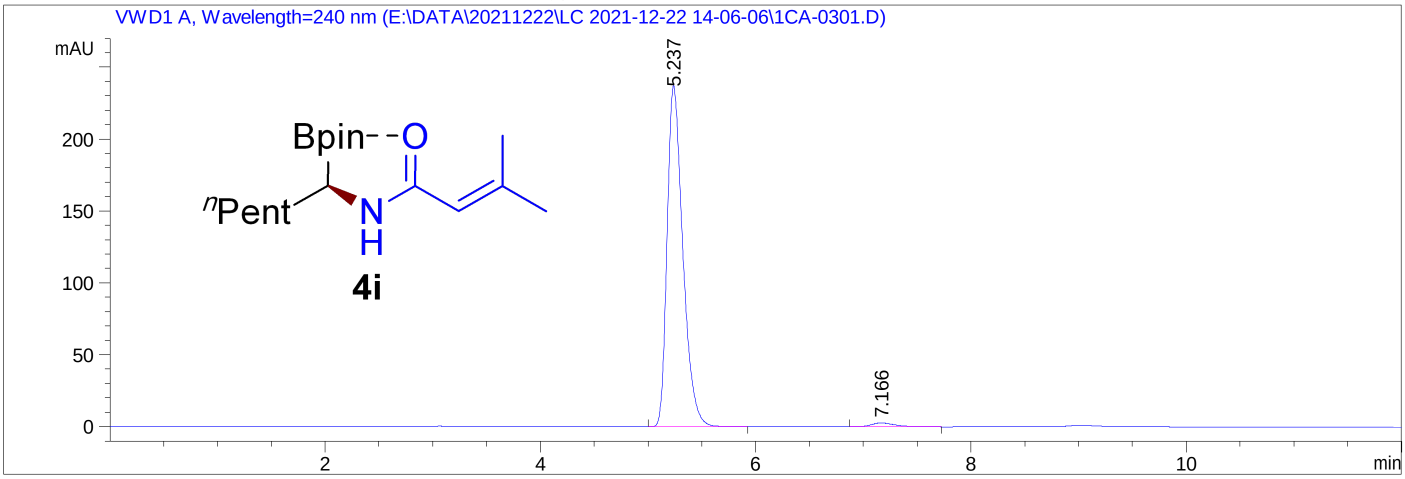

===

Area Percent Report

===

Sorted By : Signal
Multiplier : 1.0000
Dilution : 1.0000
Do not use Multiplier & Dilution Factor with ISTDs

Signal 1: VWD1 A, Wavelength=240 nm

Peak #	RetTime [min]	Type	Width [min]	Area [mAU*s]	Height [mAU]	Area %
1	5.237	BB	0.1545	2407.39844	236.98210	98.3816
2	7.166	BB	0.2291	39.60282	2.63921	1.6184

Totals : 2447.00126 239.62132

===

*** End of Report ***

Supplementary Figure 240: HPLC spectrum of **4i**.

Data File H:\ORIGINAL DATA\ZY-09-160-1-RAC.D
Sample Name: ZY-09-160-1-RAC

===

Acq. Operator : 系统 Seq. Line : 8
Acq. Instrument : HPLC-1260 Location : 31
Injection Date : 11/20/2021 11:50:54 AM Inj : 1
Inj Volume : 3.000 µl
Different Inj Volume from Sample Entry! Actual Inj Volume : 5.000 µl
Acq. Method : D:\zy\20211023\YH 2021-11-20 09-04-54\5IPA20_10-1-6-254.M
Last changed : 11/20/2021 9:33:00 AM by 系统
Analysis Method : E:\DATA\20220317\LC 2022-03-28 16-47-04\0.2IPA-40-0.5-1-XYH.M (Sequence Method)
Last changed : 3/28/2022 11:00:12 PM by SYSTEM
(modified after loading)
Additional Info : Peak(s) manually integrated

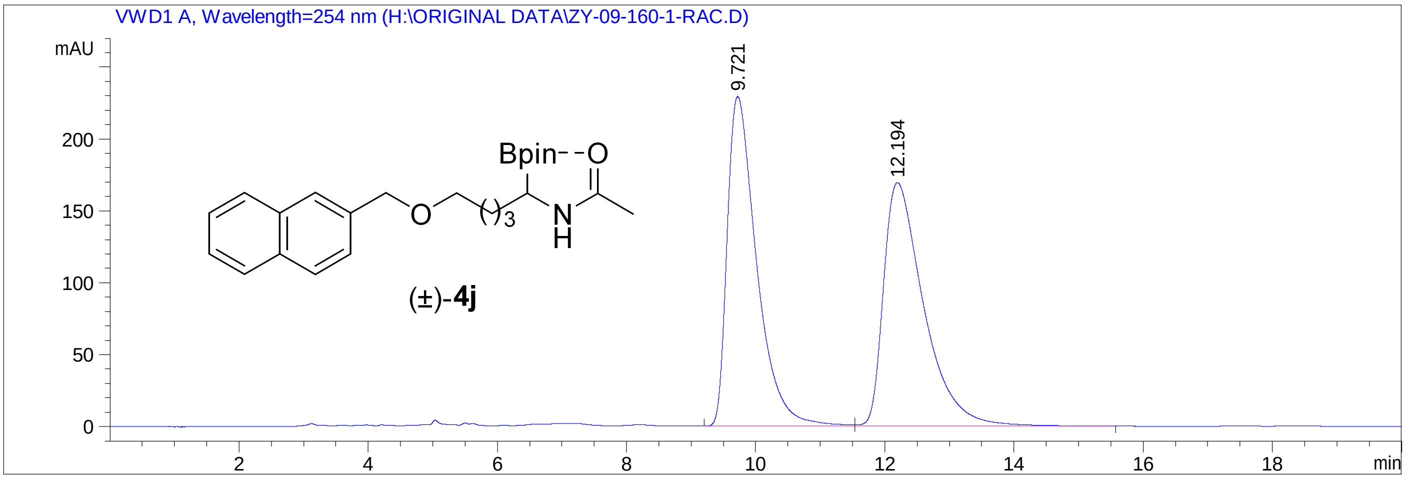

===

Area Percent Report

===

Sorted By : Signal
Multiplier : 1.0000
Dilution : 1.0000
Do not use Multiplier & Dilution Factor with ISTDs

Signal 1: VWD1 A, Wavelength=254 nm

Peak #	RetTime [min]	Type	Width [min]	Area [mAU*s]	Height [mAU]	Area %
1	9.721	BV	0.4782	7264.92676	229.26585	49.8054
2	12.194	VB	0.6462	7321.70605	169.41502	50.1946

Totals : 1.45866e4 398.68088

===

*** End of Report ***

Supplementary Figure 241: HPLC spectrum of (±)-**4j**.

Data File H:\ORIGINAL DATA\ZY-09-160-2-EE.D
Sample Name: ZY-09-160-2-EE

===

Acq. Operator : 系统 Seq. Line : 7
Acq. Instrument : HPLC-1260 Location : 32
Injection Date : 11/20/2021 11:29:33 AM Inj : 1
Inj Volume : 3.000 µl
Different Inj Volume from Sample Entry! Actual Inj Volume : 5.000 µl
Acq. Method : D:\zy\20211023\YH 2021-11-20 09-04-54\5IPA20_10-1-6-254.M
Last changed : 11/20/2021 9:33:00 AM by 系统
Analysis Method : E:\DATA\20220317\LC 2022-03-28 16-47-04\0.2IPA-40-0.5-1-XYH.M (Sequence Method)
Last changed : 3/28/2022 10:59:28 PM by SYSTEM
(modified after loading)
Additional Info : Peak(s) manually integrated

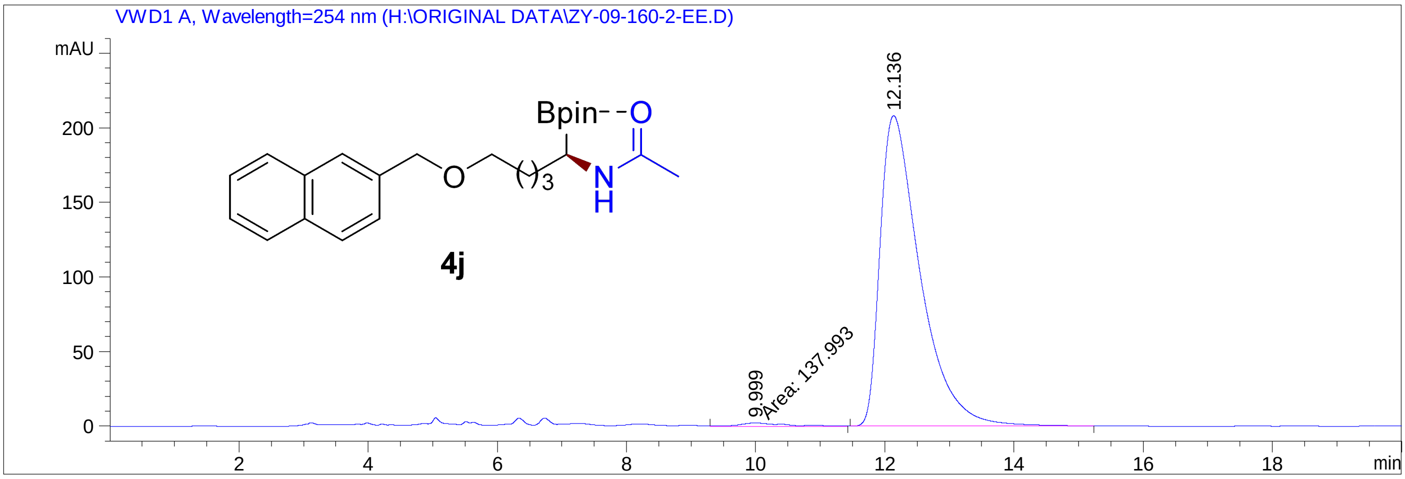

===

Area Percent Report

===

Sorted By : Signal
Multiplier : 1.0000
Dilution : 1.0000
Do not use Multiplier & Dilution Factor with ISTDs

Signal 1: VWD1 A, Wavelength=254 nm

Peak #	RetTime [min]	Type	Width [min]	Area [mAU*s]	Height [mAU]	Area %
1	9.999	MM	0.9422	137.99254	2.44099	1.5152
2	12.136	BB	0.6388	8969.10059	208.12076	98.4848

Totals : 9107.09312 210.56175

===

*** End of Report ***

Supplementary Figure 242: HPLC spectrum of **4j**.

Data File F:\ZY\AMIDE\ORIGINAL DATA\ZY-09-166-5-RAC.D
Sample Name: ZY-09-166-7-OD

===

Acq. Operator : 系统 Seq. Line : 13
Acq. Instrument : HPLC-1260 Location : 31
Injection Date : 12/4/2021 12:57:37 PM Inj : 1
Inj Volume : 3.000 µl
Different Inj Volume from Sample Entry! Actual Inj Volume : 0.500 µl
Acq. Method : D:\zy\20211129\YH 2021-12-04 09-05-16\8IPA20_5-1-2-210.M
Last changed : 12/4/2021 9:13:44 AM by 系统
Analysis Method : E:\DATA\20220316\LC 2022-03-16 22-19-08\10IPA-10-1.0-1.MZWJ.M (Sequence Method)
Last changed : 3/28/2022 11:23:29 PM by SYSTEM
(modified after loading)
Additional Info : Peak(s) manually integrated

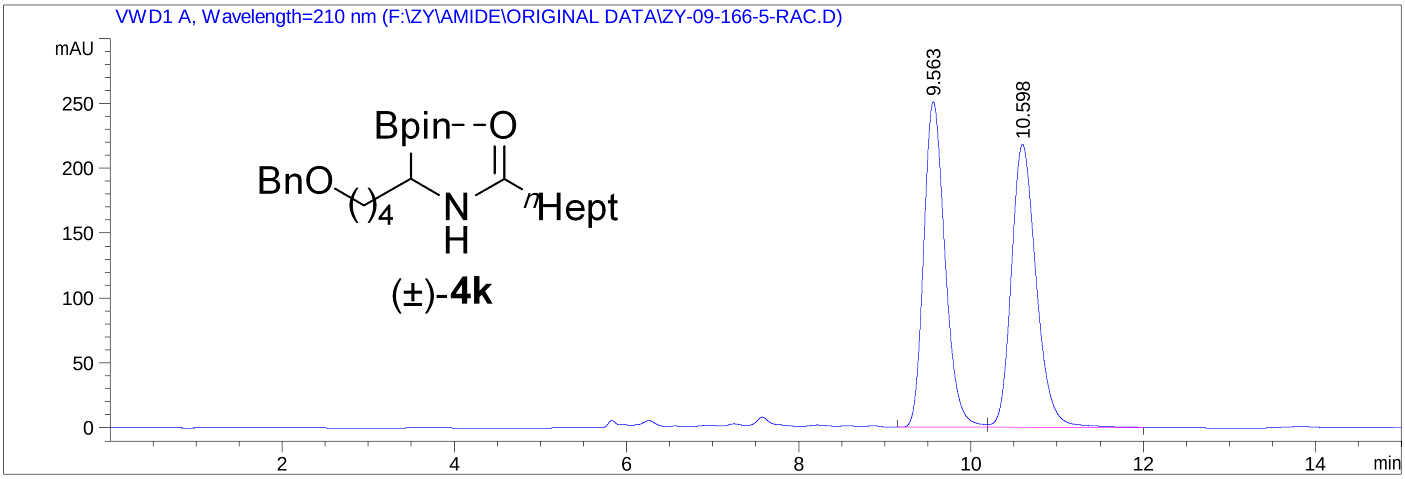

===
Area Percent Report
===

Sorted By : Signal
Multiplier : 1.0000
Dilution : 1.0000
Do not use Multiplier & Dilution Factor with ISTDs

Signal 1: VWD1 A, Wavelength=210 nm

Peak #	RetTime [min]	Type	Width [min]	Area [mAU*s]	Height [mAU]	Area %
1	9.563	BV	0.2638	4313.62402	250.64847	49.6492
2	10.598	VB	0.3083	4374.57373	217.81007	50.3508

Totals : 8688.19775 468.45854

===
*** End of Report ***

Supplementary Figure 243: HPLC spectrum of (±)-**4k**.

Data File F:\ZY\AMIDE\ORIGINAL DATA\ZY-09-166-5-EE.D
Sample Name: ZY-09-166-5-EE

===

Acq. Operator : 系统 Seq. Line : 14
Acq. Instrument : HPLC-1260 Location : 35
Injection Date : 12/4/2021 1:18:58 PM Inj : 1
Inj Volume : 3.000 µl
Different Inj Volume from Sample Entry! Actual Inj Volume : 0.400 µl
Acq. Method : D:\zy\20211129\YH 2021-12-04 09-05-16\8IPA20_5-1-2-210.M
Last changed : 12/4/2021 9:13:44 AM by 系统
Analysis Method : E:\DATA\20220316\LC 2022-03-16 22-19-08\10IPA-10-1.0-1.MZWJ.M (Sequence Method)
Last changed : 3/28/2022 11:22:39 PM by SYSTEM
(modified after loading)
Additional Info : Peak(s) manually integrated

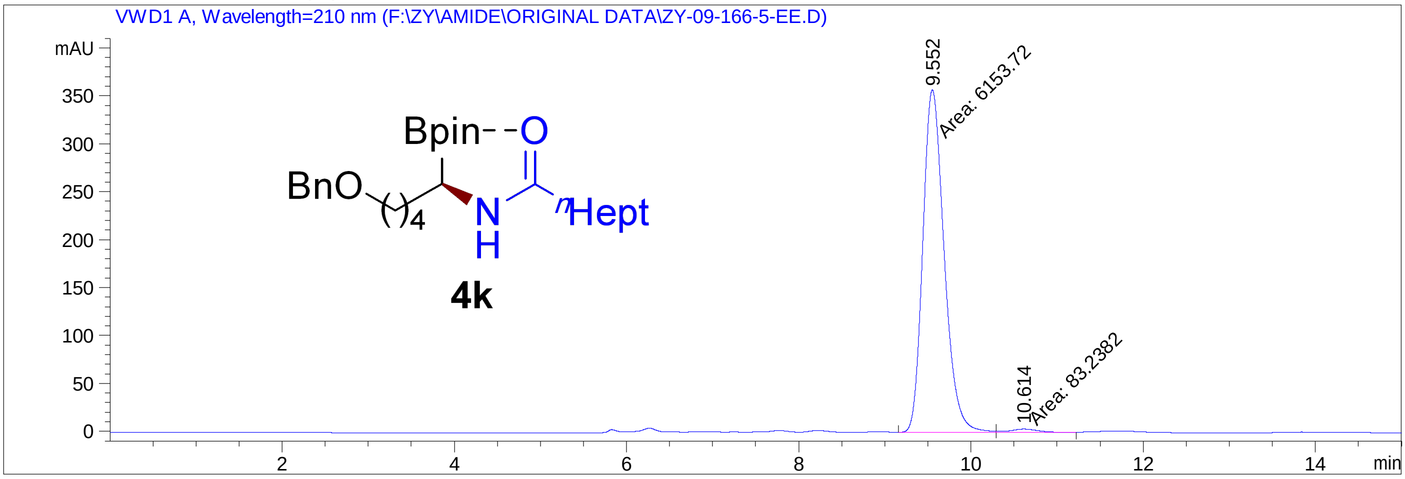

===

Area Percent Report

===

Sorted By : Signal
Multiplier : 1.0000
Dilution : 1.0000
Do not use Multiplier & Dilution Factor with ISTDs

Signal 1: VWD1 A, Wavelength=210 nm

Peak #	RetTime [min]	Type	Width [min]	Area [mAU*s]	Height [mAU]	Area %
1	9.552	MF	0.2872	6153.71680	357.14899	98.6654
2	10.614	FM	0.3918	83.23817	3.54073	1.3346

Totals : 6236.95496 360.68971

===

*** End of Report ***

Supplementary Figure 244: HPLC spectrum of **4k**.

Data File H:\1\ZY-10-115-1-RAC.D
Sample Name: ZY-10-115-2-9

===
Acq. Operator   : 系统   Seq. Line : 45
Acq. Instrument : HPLC-1260   Location :   52
Injection Date : 8/20/2022 8:27:49 PM   Inj :   1
                                               Inj Volume : 3.000 µl
Different Inj Volume from Sample Entry! Actual Inj Volume : 2.000 µl
Acq. Method   : D:\zy\20220803\YH 2022-08-20 08-41-06\5IPA16_10-1-2-210.M
Last changed   : 8/20/2022 3:56:20 PM by 系统
Analysis Method : E:\DATA\20220317\LC 2022-08-22 08-37-45\5IPA-40-1.0-1-220-QDY.M (Sequence Method)
Last changed   : 8/22/2022 7:20:24 PM by SYSTEM
                  (modified after loading)
Additional Info : Peak(s) manually integrated

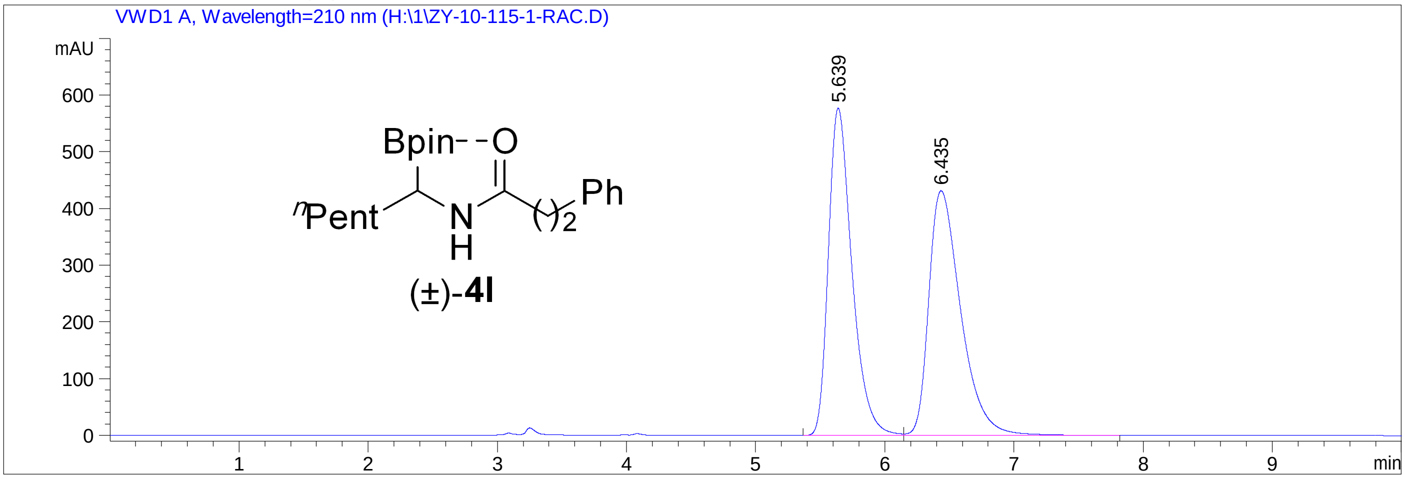

===
Area Percent Report
===

Sorted By : Signal
Multiplier : 1.0000
Dilution : 1.0000
Do not use Multiplier & Dilution Factor with ISTDs

Signal 1: VWD1 A, Wavelength=210 nm

Peak #	RetTime [min]	Type	Width [min]	Area [mAU*s]	Height [mAU]	Area %
1	5.639	BV	0.1913	7313.53271	577.17682	49.6464
2	6.435	VV R	0.2596	7417.70801	431.49738	50.3536

Totals : 1.47312e4 1008.67419

===
*** End of Report ***

Supplementary Figure 245: HPLCspectrum of (±)-**4l**.

Data File H:\1\ZY-10-115-1-EE.D
Sample Name: ZY-10-115-1-EE1

===
Acq. Operator : 系统 Seq. Line : 50
Acq. Instrument : HPLC-1260 Location : 58
Injection Date : 8/20/2022 9:53:47 PM Inj : 1
Inj Volume : 3.000 µl
Different Inj Volume from Sample Entry! Actual Inj Volume : 1.000 µl
Acq. Method : D:\zy\20220803\YH 2022-08-20 08-41-06\5IPA16_10-1-2-210.M
Last changed : 8/20/2022 9:47:09 PM by 系统
Analysis Method : E:\DATA\20220317\LC 2022-08-22 08-37-45\5IPA-40-1.0-1-220-QDY.M (Sequence Method)
Last changed : 8/22/2022 7:19:25 PM by SYSTEM
(modified after loading)
Additional Info : Peak(s) manually integrated

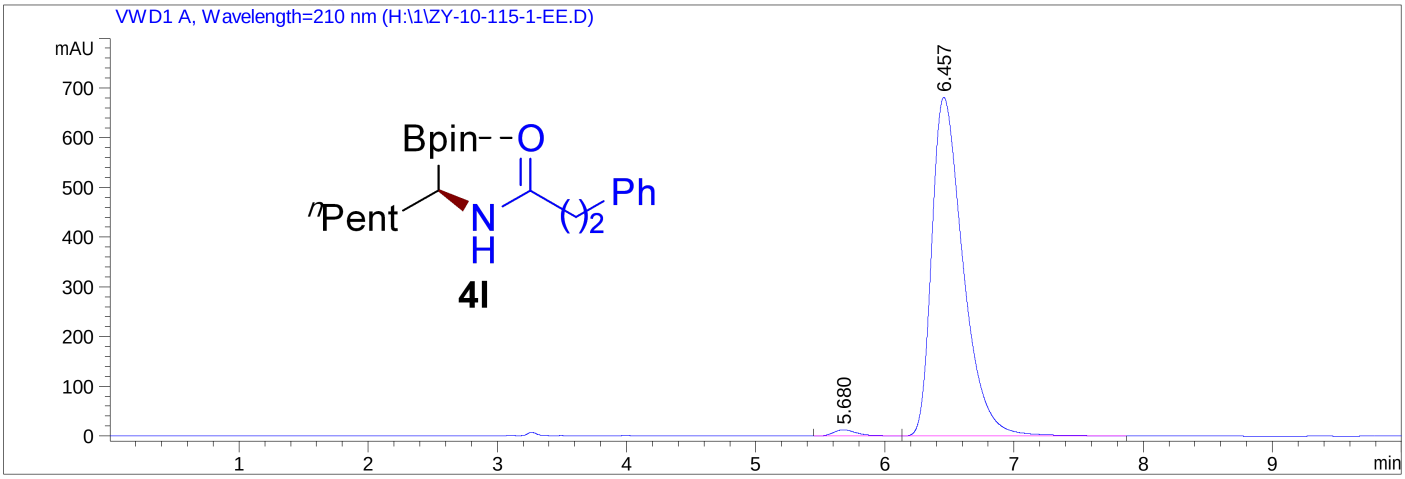

===
Area Percent Report
===

Sorted By : Signal
Multiplier : 1.0000
Dilution : 1.0000
Do not use Multiplier & Dilution Factor with ISTDs

Signal 1: VWD1 A, Wavelength=210 nm

Peak #	RetTime [min]	Type	Width [min]	Area [mAU*s]	Height [mAU]	Area %
1	5.680	BV	0.1935	161.75188	12.53654	1.3883
2	6.457	VB	0.2532	1.14890e4	681.26971	98.6117

Totals : 1.16508e4 693.80625

===
*** End of Report ***

Supplementary Figure 246: HPLC spectrum of **4l**.

Data File F:\ZY\AMIDE\ORIGINAL DATA\ZY-09-188-1-RAC.D
Sample Name: ZY-09-188-1-RAC

===

Acq. Operator   : 系统   Seq. Line :   8
Acq. Instrument : HPLC-1260   Location :   52
Injection Date : 12/19/2021 5:05:45 PM   Inj :   1
                                               Inj Volume : 3.000 µl
Different Inj Volume from Sample Entry! Actual Inj Volume : 0.500 µl
Acq. Method   : D:\zy\20211219\YH 2021-12-19 15-11-05\10IPA15_10-1-6-220-FZ.M
Last changed   : 12/19/2021 3:11:05 PM by 系统
Analysis Method : E:\DATA\20220316\LC 2022-03-16 22-19-08\0.6IPA-15-0.8-1-210-JXL.M (Sequence Method)
Last changed   : 3/29/2022 9:32:27 AM by SYSTEM
                  (modified after loading)
Additional Info : Peak(s) manually integrated

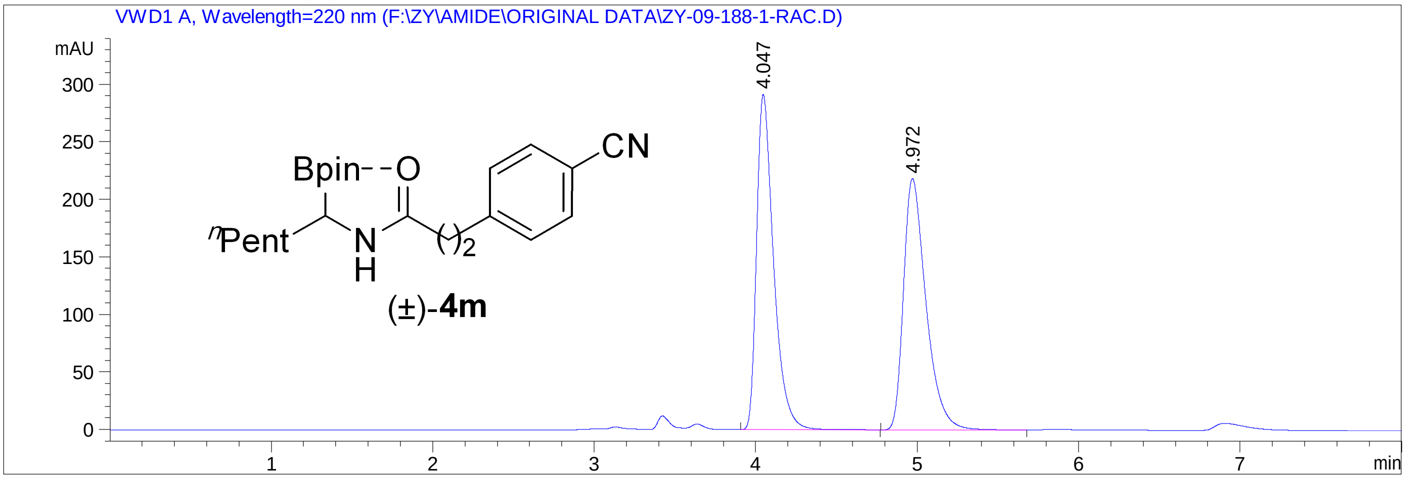

===

Area Percent Report

===

Sorted By : Signal
Multiplier : 1.0000
Dilution : 1.0000
Do not use Multiplier & Dilution Factor with ISTDs

Signal 1: VWD1 A, Wavelength=220 nm

Peak #	RetTime [min]	Type	Width [min]	Area [mAU*s]	Height [mAU]	Area %
1	4.047	BV R	0.1112	2139.70337	291.44876	50.2747
2	4.972	BB	0.1464	2116.31763	218.64890	49.7253

Totals : 4256.02100 510.09766

===

*** End of Report ***

Supplementary Figure 247: HPLC spectrum of (±)-**4m**.

Data File F:\ZY\AMIDE\ORIGINAL DATA\ZY-09-188-1-EE.D
Sample Name: ZY-09-188-1-EE

===
Acq. Operator   : 系统   Seq. Line :   6
Acq. Instrument : HPLC-1260   Location :   51
Injection Date : 12/19/2021 4:33:36 PM   Inj :   1
                                                   Inj Volume : 3.000 µl
Different Inj Volume from Sample Entry! Actual Inj Volume : 0.400 µl
Acq. Method   : D:\zy\20211219\YH 2021-12-19 15-11-05\10IPA15_10-1-6-220-FZ.M
Last changed   : 12/19/2021 3:11:05 PM by 系统
Analysis Method : E:\DATA\20220316\LC 2022-03-16 22-19-08\0.6IPA-15-0.8-1-210-JXL.M (Sequence Method)
Last changed   : 3/29/2022 9:31:28 AM by SYSTEM
                  (modified after loading)
Additional Info : Peak(s) manually integrated

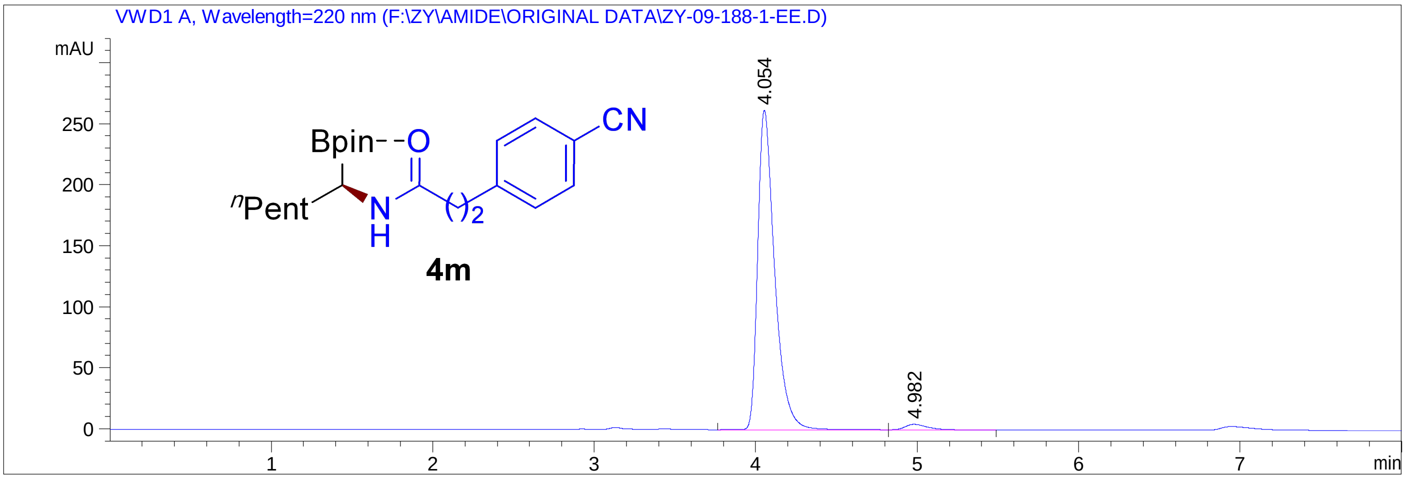

===
Area Percent Report
===

Sorted By : Signal
Multiplier : 1.0000
Dilution : 1.0000
Do not use Multiplier & Dilution Factor with ISTDs

Signal 1: VWD1 A, Wavelength=220 nm

Peak #	RetTime [min]	Type	Width [min]	Area [mAU*s]	Height [mAU]	Area %
1	4.054	VV R	0.1098	1915.36646	261.94006	97.7296
2	4.982	VB	0.1468	44.49722	4.54354	2.2704

Totals : 1959.86367 266.48360

===
*** End of Report ***

Supplementary Figure 248: HPLC spectrum of **4m**.

Data File F:\ZY\AMIDE\ORIGINAL DATA\ZY-09-188-6-RAC.D
Sample Name: ZY-09-188-6-RAC

===

Acq. Operator : 系统 Seq. Line : 43
Acq. Instrument : HPLC-1260 Location : 52
Injection Date : 12/19/2021 3:00:44 AM Inj : 1
Inj Volume : 3.000 µl
Different Inj Volume from Sample Entry! Actual Inj Volume : 0.400 µl
Acq. Method : D:\zy\20211129\YH 2021-12-18 15-56-07\5IPA15_8-1-6-220.M
Last changed : 12/18/2021 7:59:12 PM by 系统
Analysis Method : E:\DATA\20220316\LC 2022-03-16 22-19-08\0.6IPA-15-0.8-1-210-JXL.M (Sequence Method)
Last changed : 3/29/2022 9:35:09 AM by SYSTEM
(modified after loading)
Additional Info : Peak(s) manually integrated

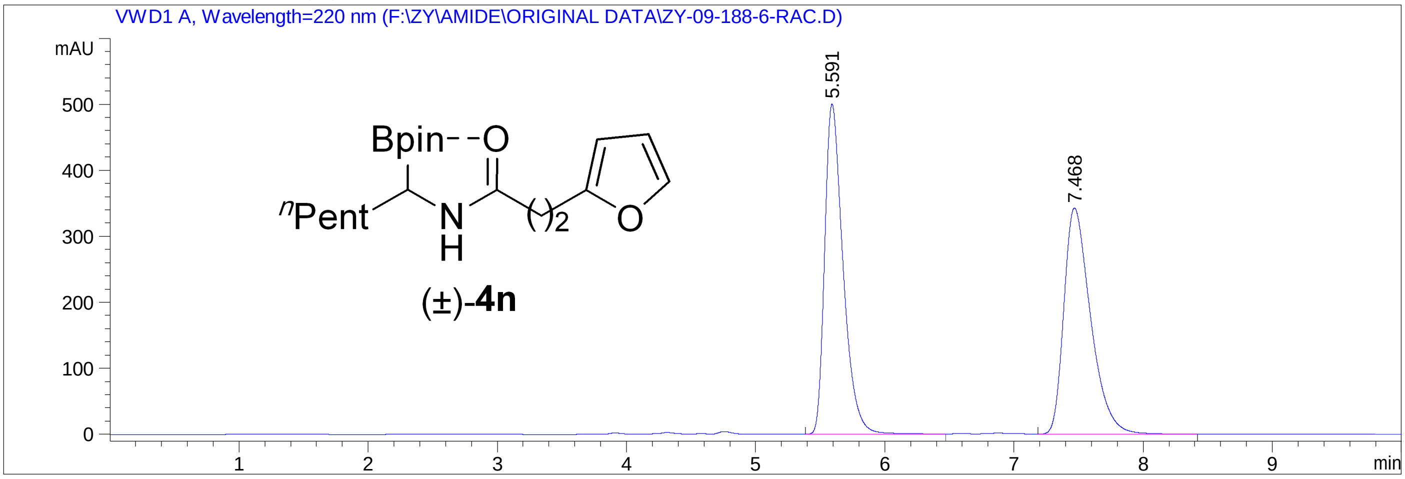

===

Area Percent Report

===

Sorted By : Signal
Multiplier : 1.0000
Dilution : 1.0000
Do not use Multiplier & Dilution Factor with ISTDs

Signal 1: VWD1 A, Wavelength=220 nm

Peak #	RetTime [min]	Type	Width [min]	Area [mAU*s]	Height [mAU]	Area %
1	5.591	BV R	0.1505	4951.50684	499.95935	50.0940
2	7.468	BB	0.2189	4932.93262	342.67126	49.9060

Totals : 9884.43945 842.63062

===

*** End of Report ***

Supplementary Figure 249: HPLC spectrum of (±)-**4n**.

Data File F:\ZY\AMIDE\ORIGINAL DATA\ZY-09-188-6-EE.D
Sample Name: ZY-09-188-6-EE

===

Acq. Operator : 系统 Seq. Line : 40
Acq. Instrument : HPLC-1260 Location : 51
Injection Date : 12/19/2021 2:12:39 AM Inj : 1
Inj Volume : 3.000 µl
Different Inj Volume from Sample Entry! Actual Inj Volume : 0.400 µl
Acq. Method : D:\zy\20211129\YH 2021-12-18 15-56-07\5IPA15_8-1-6-220.M
Last changed : 12/18/2021 7:59:12 PM by 系统
Analysis Method : E:\DATA\20220316\LC 2022-03-16 22-19-08\0.6IPA-15-0.8-1-210-JXL.M (Sequence Method)
Last changed : 3/29/2022 9:34:29 AM by SYSTEM
(modified after loading)
Additional Info : Peak(s) manually integrated

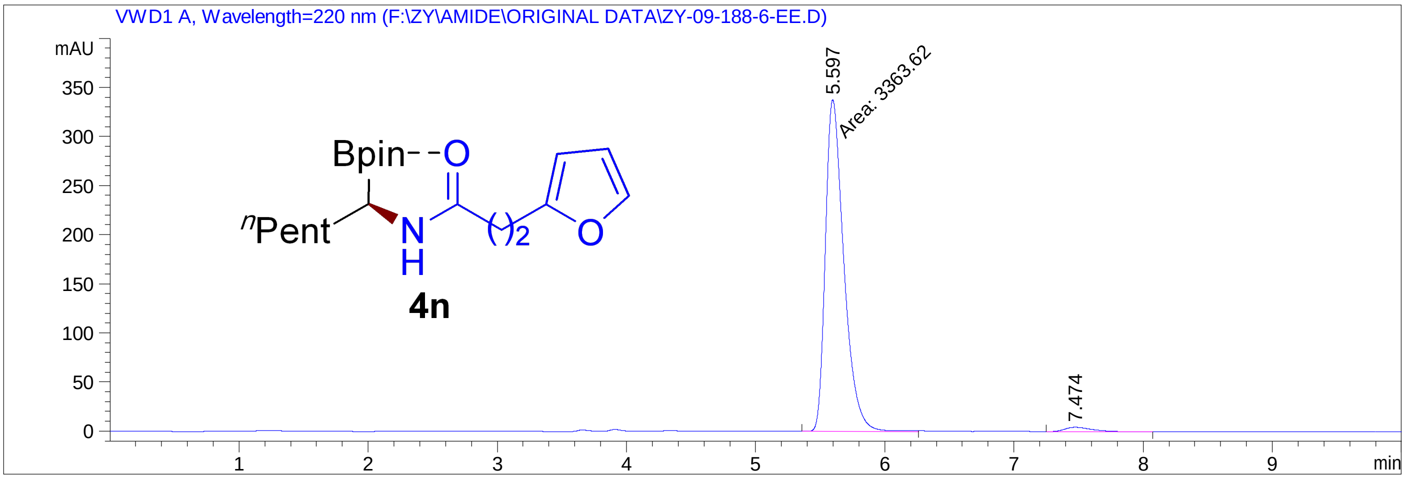

===

Area Percent Report

===

Sorted By : Signal
Multiplier : 1.0000
Dilution : 1.0000
Do not use Multiplier & Dilution Factor with ISTDs

Signal 1: VWD1 A, Wavelength=220 nm

Peak #	RetTime [min]	Type	Width [min]	Area [mAU*s]	Height [mAU]	Area %
1	5.597	MF	0.1660	3363.61768	337.73022	98.0657
2	7.474	BB	0.2115	66.34515	4.51200	1.9343

Totals : 3429.96283 342.24223

===

*** End of Report ***

Supplementary Figure 250: HPLC spectrum of **4n**.

Data File F:\ZY\AMIDE\ORIGINAL DATA\ZY-09-195-5-RAC.D
Sample Name: ZY-09-195-5-RAC

===

Acq. Operator : 系统 Seq. Line : 8
Acq. Instrument : HPLC-1260 Location : 62
Injection Date : 12/31/2021 6:45:20 PM Inj : 1
Inj Volume : 3.000 µl
Different Inj Volume from Sample Entry! Actual Inj Volume : 1.000 µl
Acq. Method : D:\zy\20211226\YH 2021-12-31 16-20-46\5IPA-15-1.0-1-2-220.M
Last changed : 12/31/2021 5:46:49 PM by 系统
Analysis Method : E:\DATA\20220316\LC 2022-03-16 22-19-08\10IPA-10-1.0-1.MZWJ.M (Sequence Method)
Last changed : 3/29/2022 9:53:44 AM by SYSTEM
(modified after loading)
Additional Info : Peak(s) manually integrated

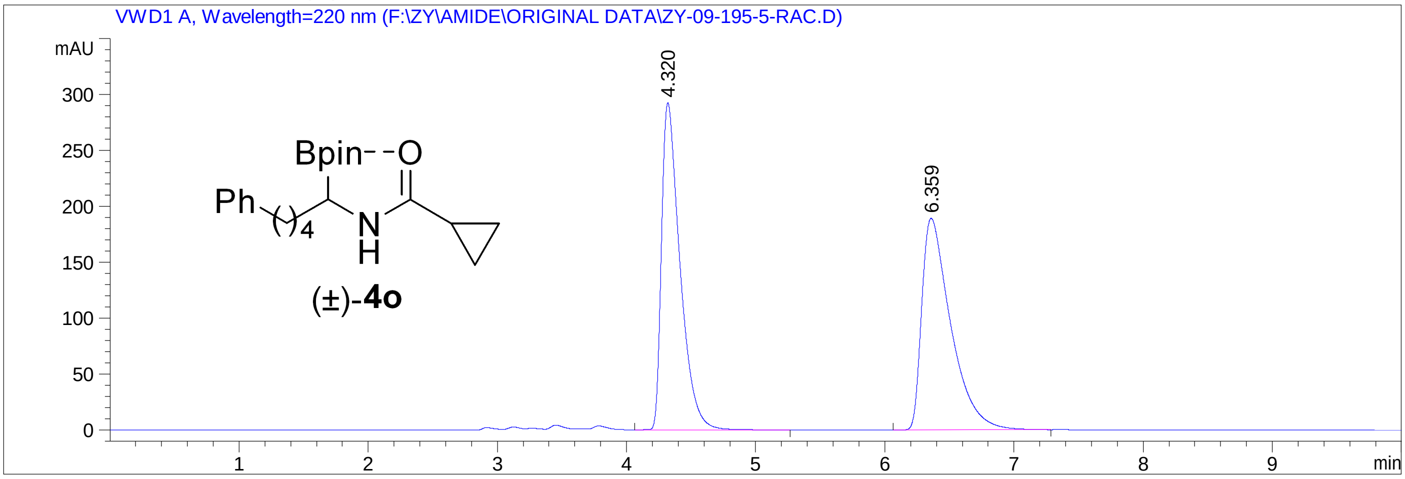

===

Area Percent Report

===

Sorted By : Signal
Multiplier : 1.0000
Dilution : 1.0000
Do not use Multiplier & Dilution Factor with ISTDs

Signal 1: VWD1 A, Wavelength=220 nm

Peak #	RetTime [min]	Type	Width [min]	Area [mAU*s]	Height [mAU]	Area %
1	4.320	BB	0.1479	2904.71558	292.34595	49.6171
2	6.359	BB	0.2328	2949.54614	189.23438	50.3829

Totals : 5854.26172 481.58032

===

*** End of Report ***

Supplementary Figure 251: HPLC spectrum of (±)-**4o**.

Data File F:\ZY\AMIDE\ORIGINAL DATA\ZY-09-195-5-EE.D
Sample Name: ZY-09-195-5-EE

===

Acq. Operator : 系统 Seq. Line : 9
Acq. Instrument : HPLC-1260 Location : 61
Injection Date : 12/31/2021 7:01:41 PM Inj : 1
Inj Volume : 3.000 µl
Different Inj Volume from Sample Entry! Actual Inj Volume : 1.800 µl
Acq. Method : D:\zy\20211226\YH 2021-12-31 16-20-46\5IPA-15-1.0-1-2-220.M
Last changed : 12/31/2021 5:46:49 PM by 系统
Analysis Method : E:\DATA\20220316\LC 2022-03-16 22-19-08\10IPA-10-1.0-1.MZWJ.M (Sequence Method)
Last changed : 3/29/2022 9:54:16 AM by SYSTEM
(modified after loading)
Additional Info : Peak(s) manually integrated

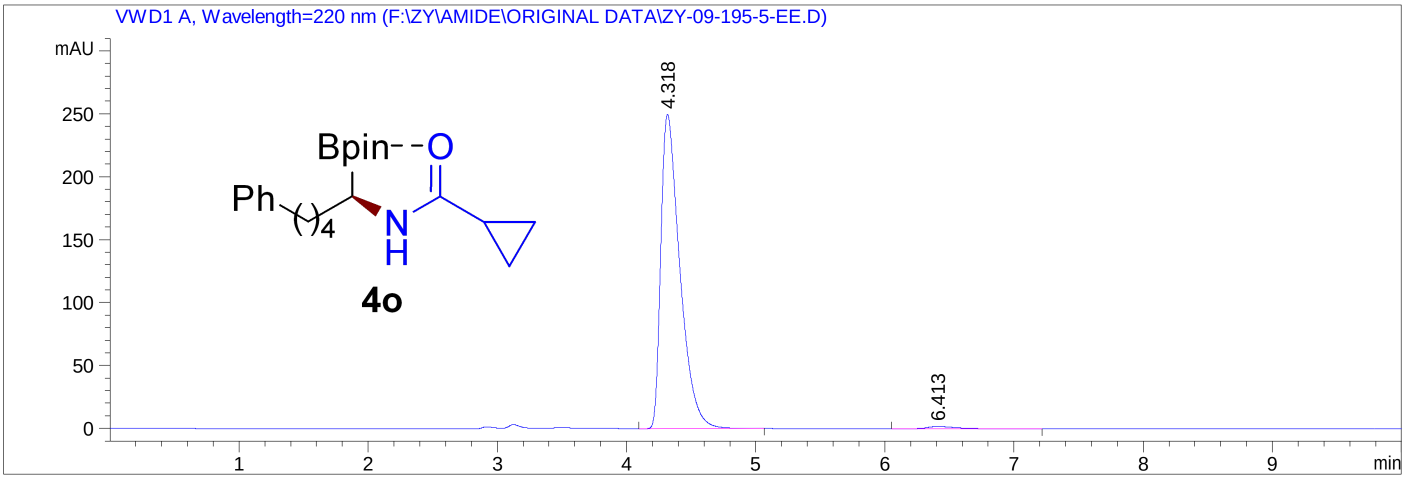

===

Area Percent Report

===

Sorted By : Signal
Multiplier : 1.0000
Dilution : 1.0000
Do not use Multiplier & Dilution Factor with ISTDs

Signal 1: VWD1 A, Wavelength=220 nm

Peak #	RetTime [min]	Type	Width [min]	Area [mAU*s]	Height [mAU]	Area %
1	4.318	BB	0.1538	2553.71533	249.79950	98.7791
2	6.413	BB	0.2204	31.56495	1.86559	1.2209

Totals : 2585.28028 251.66509

===

*** End of Report ***

Supplementary Figure 252: HPLC spectrum of **4o**.

Data File F:\ZY\AMIDE\ORIGINAL DATA\ZY-10-24-2-RAC.D
Sample Name: ZY-10-24-2-RAC

===

Acq. Operator : 系统 Seq. Line : 19
Acq. Instrument : HPLC-1260 Location : 82
Injection Date : 2/15/2022 8:34:49 PM Inj : 1
Inj Volume : 3.000 µl
Different Inj Volume from Sample Entry! Actual Inj Volume : 2.600 µl
Acq. Method : D:\zy\20220201\YH 2022-02-15 14-53-54\5IPA20_10-1-2-210.M
Last changed : 2/15/2022 7:02:39 PM by 系统
Analysis Method : E:\DATA\20220316\LC 2022-03-16 22-19-08\10IPA-10-1.0-1.MZWJ.M (Sequence Method)
Last changed : 3/29/2022 10:00:58 AM by SYSTEM
(modified after loading)
Additional Info : Peak(s) manually integrated

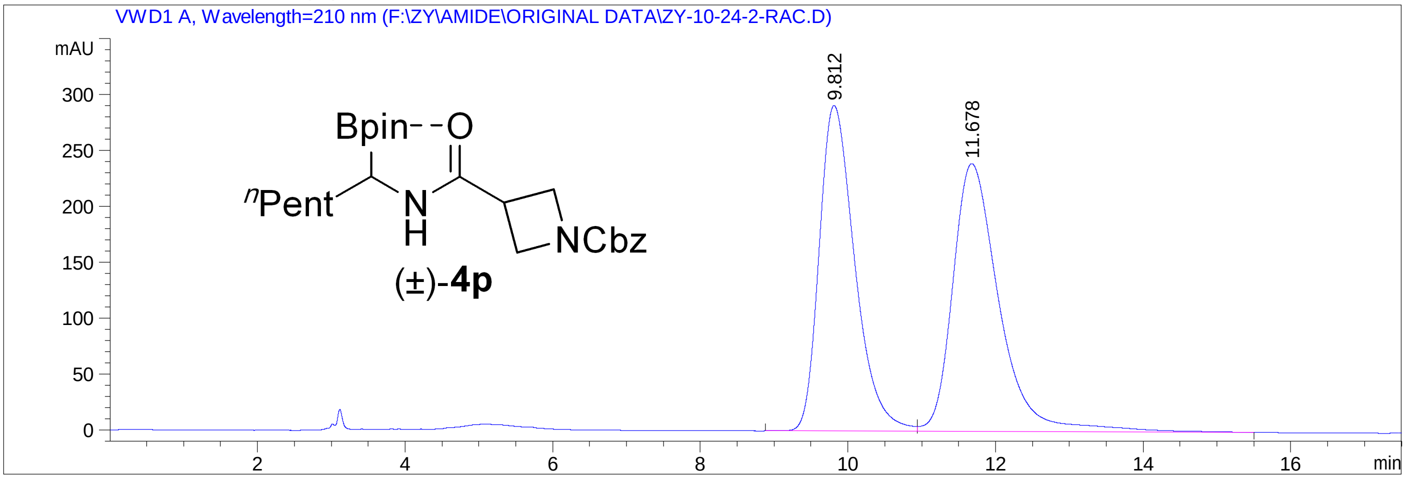

===

Area Percent Report

===

Sorted By : Signal
Multiplier : 1.0000
Dilution : 1.0000
Do not use Multiplier & Dilution Factor with ISTDs

Signal 1: VWD1 A, Wavelength=210 nm

Peak #	RetTime [min]	Type	Width [min]	Area [mAU*s]	Height [mAU]	Area %
1	9.812	BV	0.5117	9773.50684	291.12683	48.2130
2	11.678	VB	0.6469	1.04980e4	239.47452	51.7870

Totals : 2.02715e4 530.60135

===

*** End of Report ***

Supplementary Figure 253: HPLC spectrum of (±)-**4p**.

Data File F:\ZY\AMIDE\ORIGINAL DATA\ZY-10-24-2-EE.D
Sample Name: ZY-10-24-2-EE

===

Acq. Operator : 系统 Seq. Line : 18
Acq. Instrument : HPLC-1260 Location : 81
Injection Date : 2/15/2022 8:13:24 PM Inj : 1
Inj Volume : 3.000 µl
Different Inj Volume from Sample Entry! Actual Inj Volume : 2.800 µl
Acq. Method : D:\zy\20220201\YH 2022-02-15 14-53-54\5IPA20_10-1-2-210.M
Last changed : 2/15/2022 7:02:39 PM by 系统
Analysis Method : E:\DATA\20220316\LC 2022-03-16 22-19-08\10IPA-10-1.0-1.MZWJ.M (Sequence Method)
Last changed : 3/29/2022 10:00:58 AM by SYSTEM
(modified after loading)
Additional Info : Peak(s) manually integrated

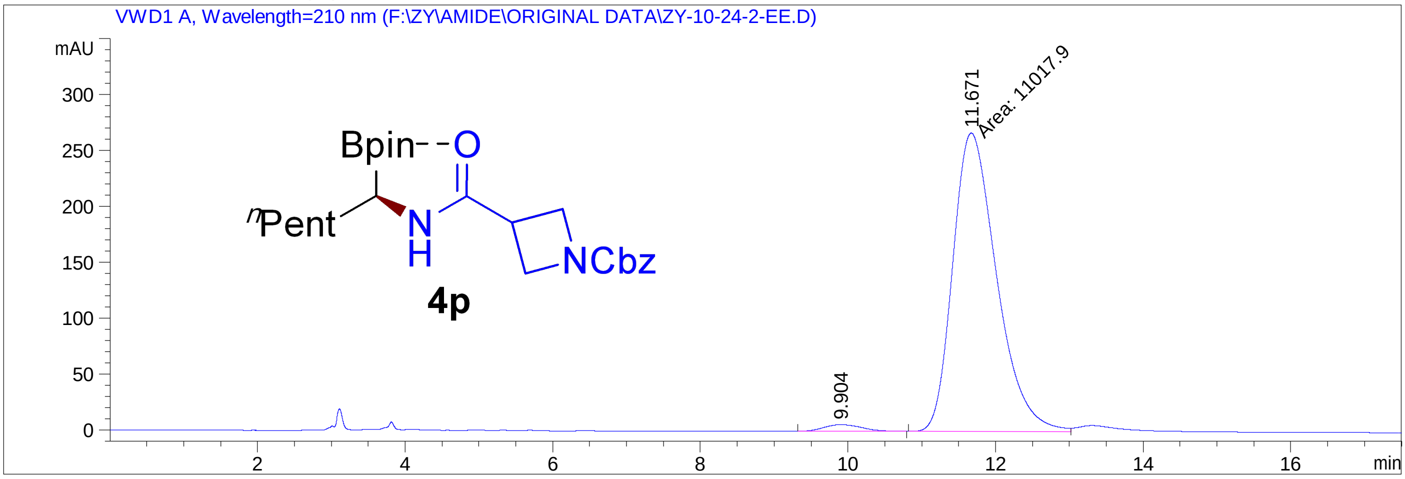

===

Area Percent Report

===

Sorted By : Signal
Multiplier : 1.0000
Dilution : 1.0000
Do not use Multiplier & Dilution Factor with ISTDs

Signal 1: VWD1 A, Wavelength=210 nm

Peak #	RetTime [min]	Type	Width [min]	Area [mAU*s]	Height [mAU]	Area %
1	9.904	BV R	0.4181	211.22409	5.92163	1.8810
2	11.671	MF	0.6879	1.10179e4	266.93561	98.1190

Totals : 1.12291e4 272.85724

===

*** End of Report ***

Supplementary Figure 254: HPLC spectrum of **4p**.

Data File F:\ZY\AMIDE\ORIGINAL DATA\ZY-09-166-1-RAC.D
Sample Name: ZY-09-166-1-RAC

===

Acq. Operator : 系统 Seq. Line : 10
Acq. Instrument : HPLC-1260 Location : 1
Injection Date : 12/3/2021 12:10:23 PM Inj : 1
Inj Volume : 3.000 µl
Different Inj Volume from Sample Entry! Actual Inj Volume : 1.800 µl
Acq. Method : D:\zy\20211203\YH 2021-12-03 09-47-06\5IPA-20-1.0-1-6-220-JXL.M
Last changed : 12/3/2021 11:14:35 AM by 系统
Analysis Method : E:\DATA\20220316\LC 2022-03-16 22-19-08\10IPA-10-1.0-1.MZWJ.M (Sequence Method)
Last changed : 3/28/2022 11:19:50 PM by SYSTEM
(modified after loading)
Additional Info : Peak(s) manually integrated

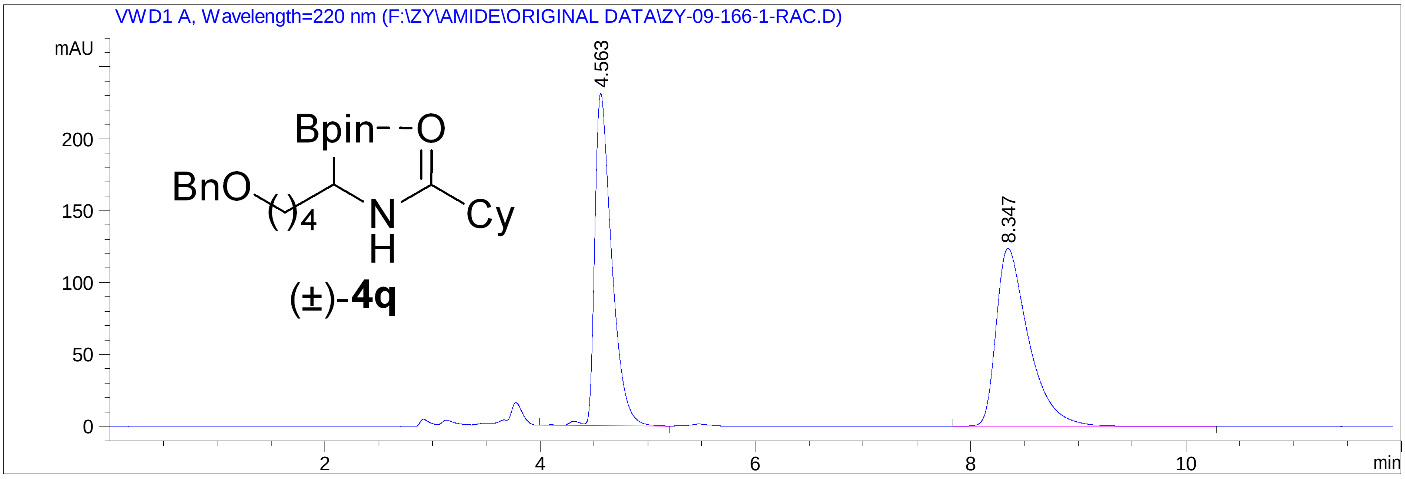

===

Area Percent Report

===

Sorted By : Signal
Multiplier : 1.0000
Dilution : 1.0000
Do not use Multiplier & Dilution Factor with ISTDs

Signal 1: VWD1 A, Wavelength=220 nm

Peak #	RetTime [min]	Type	Width [min]	Area [mAU*s]	Height [mAU]	Area %
1	4.563	VB R	0.1617	2529.77661	231.13509	49.2039
2	8.347	BB	0.3129	2611.63428	123.91994	50.7961

Totals : 5141.41089 355.05503

===

*** End of Report ***

Supplementary Figure 255: HPLC spectrum of (±)-**4q**.

Data File F:\ZY\AMIDE\ORIGINAL DATA\ZY-09-166-1-EE.D
Sample Name: ZY-09-166-1-EE

===

Acq. Operator : 系统 Seq. Line : 9
Acq. Instrument : HPLC-1260 Location : 2
Injection Date : 12/3/2021 11:53:59 AM Inj : 1
Inj Volume : 3.000 µl
Different Inj Volume from Sample Entry! Actual Inj Volume : 1.800 µl
Acq. Method : D:\zy\20211203\YH 2021-12-03 09-47-06\5IPA-20-1.0-1-6-220-JXL.M
Last changed : 12/3/2021 11:14:35 AM by 系统
Analysis Method : E:\DATA\20220316\LC 2022-03-16 22-19-08\10IPA-10-1.0-1.MZWJ.M (Sequence Method)
Last changed : 3/28/2022 11:19:09 PM by SYSTEM
(modified after loading)
Additional Info : Peak(s) manually integrated

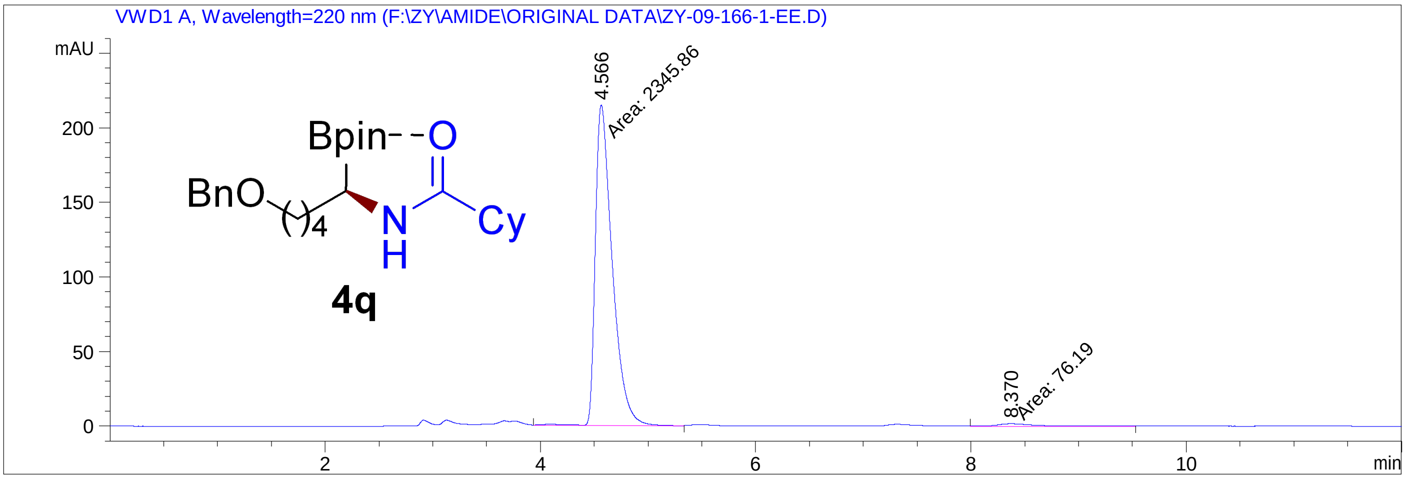

===

Area Percent Report

===

Sorted By : Signal
Multiplier : 1.0000
Dilution : 1.0000
Do not use Multiplier & Dilution Factor with ISTDs

Signal 1: VWD1 A, Wavelength=220 nm

Peak #	RetTime [min]	Type	Width [min]	Area [mAU*s]	Height [mAU]	Area %
1	4.566	MF	0.1819	2345.85986	214.88185	96.8543
2	8.370	MM	0.6441	76.19001	1.97147	3.1457

Totals : 2422.04987 216.85332

===

*** End of Report ***

Supplementary Figure 256: HPLC spectrum of **4q**.

Data File F:\ZY\AMIDE\ORIGINAL DATA\ZY-09-167-1-RAC.D
Sample Name: ZY-09-167-1-RAC

===

Acq. Operator : 系统 Seq. Line : 4
Acq. Instrument : HPLC-1260 Location : 31
Injection Date : 12/5/2021 10:10:44 PM Inj : 1
Inj Volume : 3.000 µl
Different Inj Volume from Sample Entry! Actual Inj Volume : 1.800 µl
Acq. Method : D:\zy\20211129\YH 2021-12-05 21-28-00\5IPA-15-1-1-2-220-JXL.M
Last changed : 12/5/2021 9:53:06 PM by 系统
Analysis Method : E:\DATA\20220316\LC 2022-03-16 22-19-08\10IPA-10-1.0-1.MZWJ.M (Sequence Method)
Last changed : 3/28/2022 11:26:24 PM by SYSTEM
(modified after loading)
Additional Info : Peak(s) manually integrated

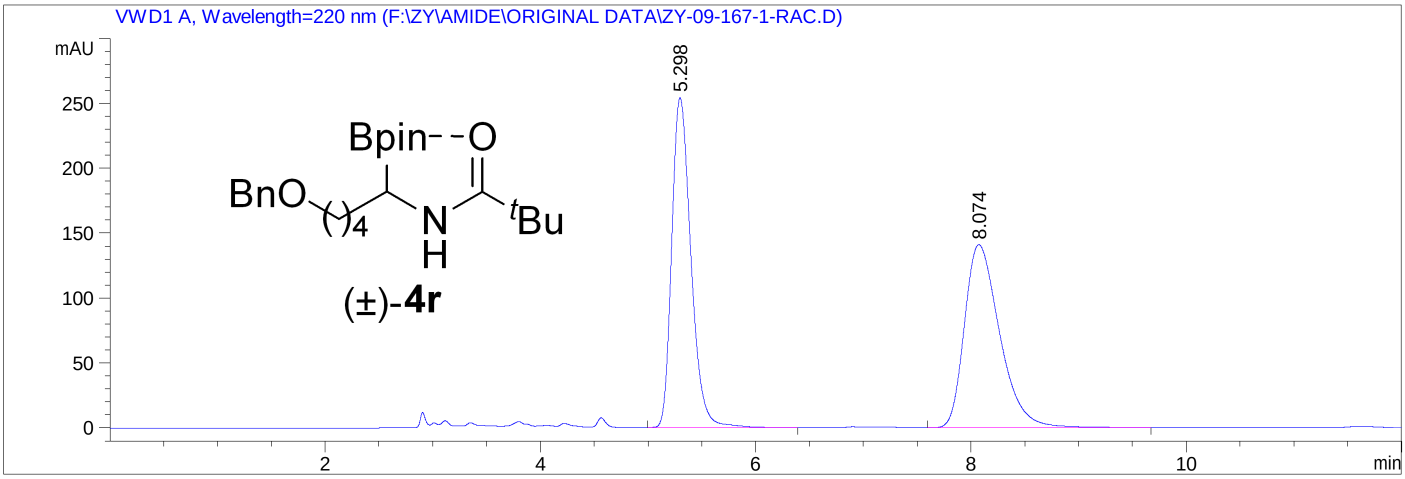

===

Area Percent Report

===

Sorted By : Signal
Multiplier : 1.0000
Dilution : 1.0000
Do not use Multiplier & Dilution Factor with ISTDs

Signal 1: VWD1 A, Wavelength=220 nm

Peak #	RetTime [min]	Type	Width [min]	Area [mAU*s]	Height [mAU]	Area %
1	5.298	BB	0.1920	3145.98950	253.95166	49.5685
2	8.074	BB	0.3471	3200.76025	140.88438	50.4315

Totals : 6346.74976 394.83604

===

*** End of Report ***

Supplementary Figure 257: HPLC spectrum of (±)-**4r**.

Data File F:\ZY\AMIDE\ORIGINAL DATA\ZY-09-167-1-EE.D
Sample Name: ZY-09-167-1-EE

===

Acq. Operator : 系统 Seq. Line : 3
Acq. Instrument : HPLC-1260 Location : 32
Injection Date : 12/5/2021 9:53:10 PM Inj : 1
Inj Volume : 3.000 µl
Different Inj Volume from Sample Entry! Actual Inj Volume : 1.600 µl
Acq. Method : D:\zy\20211129\YH 2021-12-05 21-28-00\5IPA-15-1-1-2-220-JXL.M
Last changed : 12/5/2021 9:53:06 PM by 系统
(modified after loading)
Analysis Method : E:\DATA\20220316\LC 2022-03-16 22-19-08\10IPA-10-1.0-1.MZWJ.M (Sequence Method)
Last changed : 3/28/2022 11:25:31 PM by SYSTEM
(modified after loading)
Additional Info : Peak(s) manually integrated

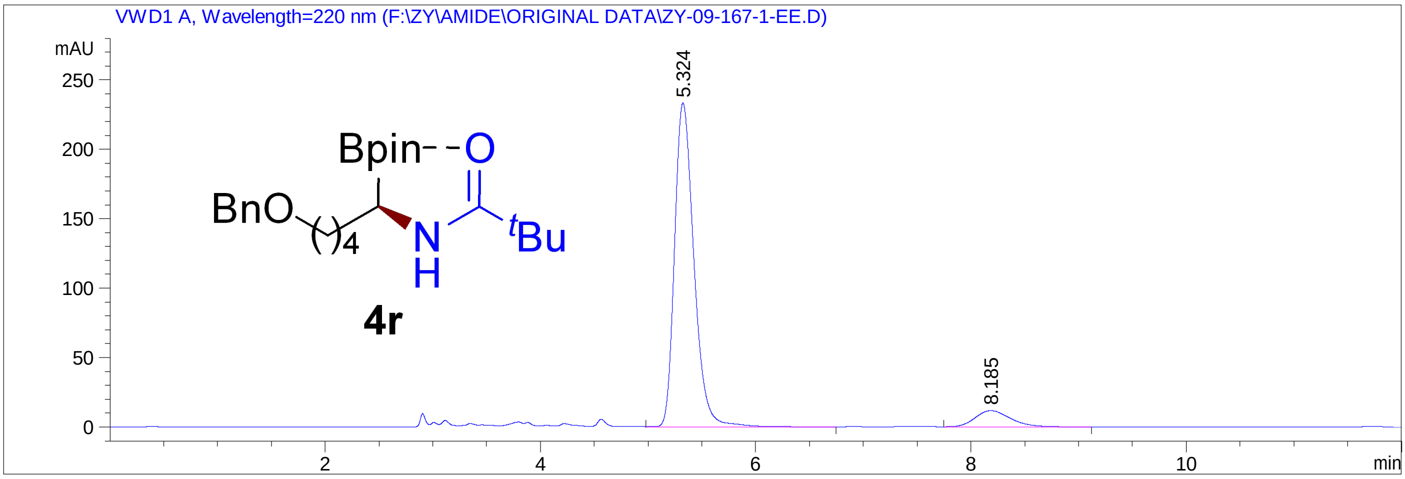

===

Area Percent Report

===

Sorted By : Signal
Multiplier : 1.0000
Dilution : 1.0000
Do not use Multiplier & Dilution Factor with ISTDs

Signal 1: VWD1 A, Wavelength=220 nm

Peak #	RetTime [min]	Type	Width [min]	Area [mAU*s]	Height [mAU]	Area %
1	5.324	BB	0.1931	2913.18408	233.36481	91.6417
2	8.185	BB	0.3437	265.69937	11.66675	8.3583

Totals : 3178.88345 245.03156

===

*** End of Report ***

Supplementary Figure 258: HPLC spectrum of **4r**.

HPLC1260 3/28/2022 11:25:33 PM SYSTEM Page 1 of 1

Data File F:\ZY\AMIDE\ORIGINAL DATA\ZY-09-194-5-RAC.D
Sample Name: ZY-09-194-5-RAC

===

Acq. Operator : 系统 Seq. Line : 7
Acq. Instrument : HPLC-1260 Location : 22
Injection Date : 1/3/2022 10:53:04 AM Inj : 1
Inj Volume : 3.000 µl
Different Inj Volume from Sample Entry! Actual Inj Volume : 1.600 µl
Acq. Method : D:\zy\20211226\YH 2022-01-03 09-22-34\5IPA-15-1.0-1-2-220.M
Last changed : 1/3/2022 10:03:22 AM by 系统
Analysis Method : E:\DATA\20220316\LC 2022-03-16 22-19-08\10IPA-10-1.0-1.MZWJ.M (Sequence Method)
Last changed : 3/29/2022 9:49:15 AM by SYSTEM
(modified after loading)
Additional Info : Peak(s) manually integrated

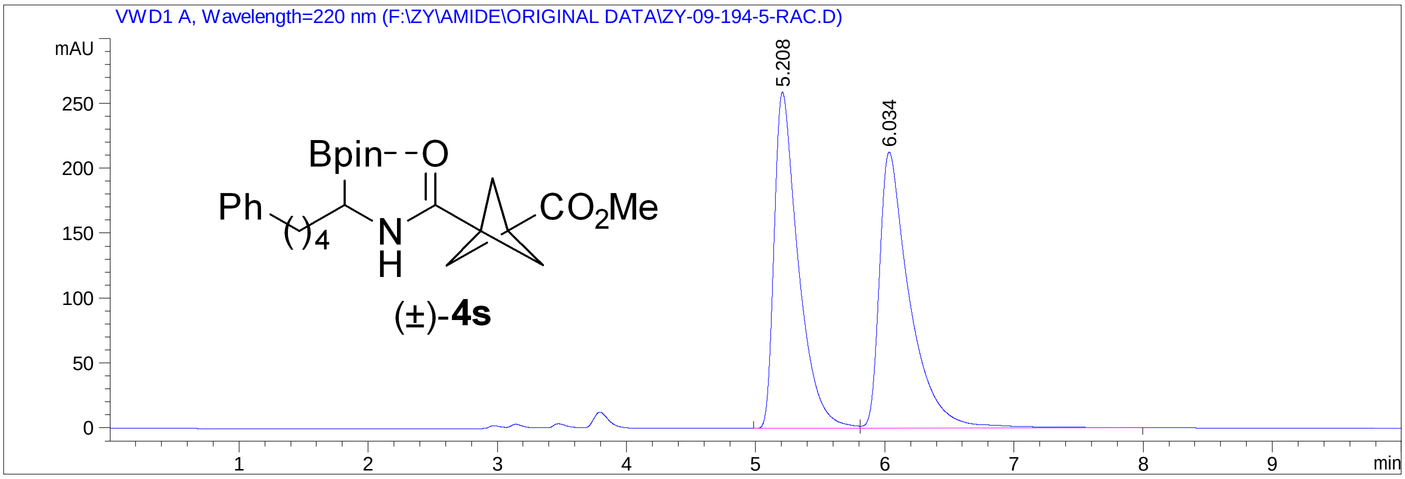

===

Area Percent Report

===

Sorted By : Signal
Multiplier : 1.0000
Dilution : 1.0000
Do not use Multiplier & Dilution Factor with ISTDs

Signal 1: VWD1 A, Wavelength=220 nm

Peak #	RetTime [min]	Type	Width [min]	Area [mAU*s]	Height [mAU]	Area %
1	5.208	BV	0.1897	3366.52661	258.92990	49.2252
2	6.034	VV R	0.2345	3472.50415	212.65123	50.7748

Totals : 6839.03076 471.58113

===

*** End of Report ***

Supplementary Figure 259: HPLC spectrum of (±)-**4s**.

Data File F:\ZY\AMIDE\ORIGINAL DATA\ZY-09-194-5-EE.D
Sample Name: ZY-09-194-5-EE

===
Acq. Operator : 系统 Seq. Line : 6
Acq. Instrument : HPLC-1260 Location : 21
Injection Date : 1/3/2022 10:36:44 AM Inj : 1
Inj Volume : 3.000 µl
Different Inj Volume from Sample Entry! Actual Inj Volume : 0.800 µl
Acq. Method : D:\zy\20211226\YH 2022-01-03 09-22-34\5IPA-15-1.0-1-2-220.M
Last changed : 1/3/2022 10:03:22 AM by 系统
Analysis Method : E:\DATA\20220316\LC 2022-03-16 22-19-08\10IPA-10-1.0-1.MZWJ.M (Sequence Method)
Last changed : 3/29/2022 9:49:15 AM by SYSTEM
(modified after loading)
Additional Info : Peak(s) manually integrated

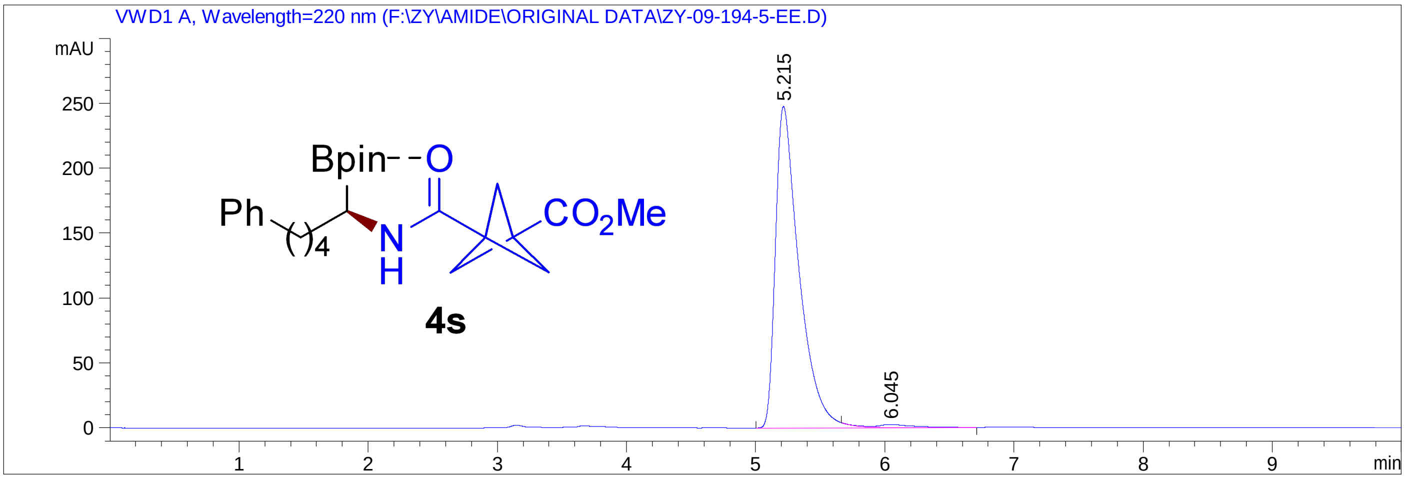

===
Area Percent Report
===

Sorted By : Signal
Multiplier : 1.0000
Dilution : 1.0000
Do not use Multiplier & Dilution Factor with ISTDs

Signal 1: VWD1 A, Wavelength=220 nm

Peak #	RetTime [min]	Type	Width [min]	Area [mAU*s]	Height [mAU]	Area %
1	5.215	BV R	0.1852	3155.59595	247.50763	98.5435
2	6.045	VB E	0.2622	46.64185	2.39526	1.4565

Totals : 3202.23780 249.90289

===
*** End of Report ***

Supplementary Figure 260: HPLC spectrum of **4s**.

Data File F:\ZY\AMIDE\ORIGINAL DATA\ZY-09-165-5-RAC.D
Sample Name: ZY-09-165-5-RAC

===

Acq. Operator : 系统 Seq. Line : 3
Acq. Instrument : HPLC-1260 Location : 52
Injection Date : 12/27/2021 9:31:34 AM Inj : 1
Inj Volume : 3.000 µl
Different Inj Volume from Sample Entry! Actual Inj Volume : 1.000 µl
Acq. Method : D:\zy\20211226\YH 2021-12-27 08-56-44\10IPA15_1-1-2-240.M
Last changed : 12/27/2021 8:56:44 AM by 系统
Analysis Method : E:\DATA\20220316\LC 2022-03-16 22-19-08\10IPA-10-1.0-1.MZWJ.M (Sequence Method)
Last changed : 3/28/2022 11:11:46 PM by SYSTEM
(modified after loading)
Additional Info : Peak(s) manually integrated

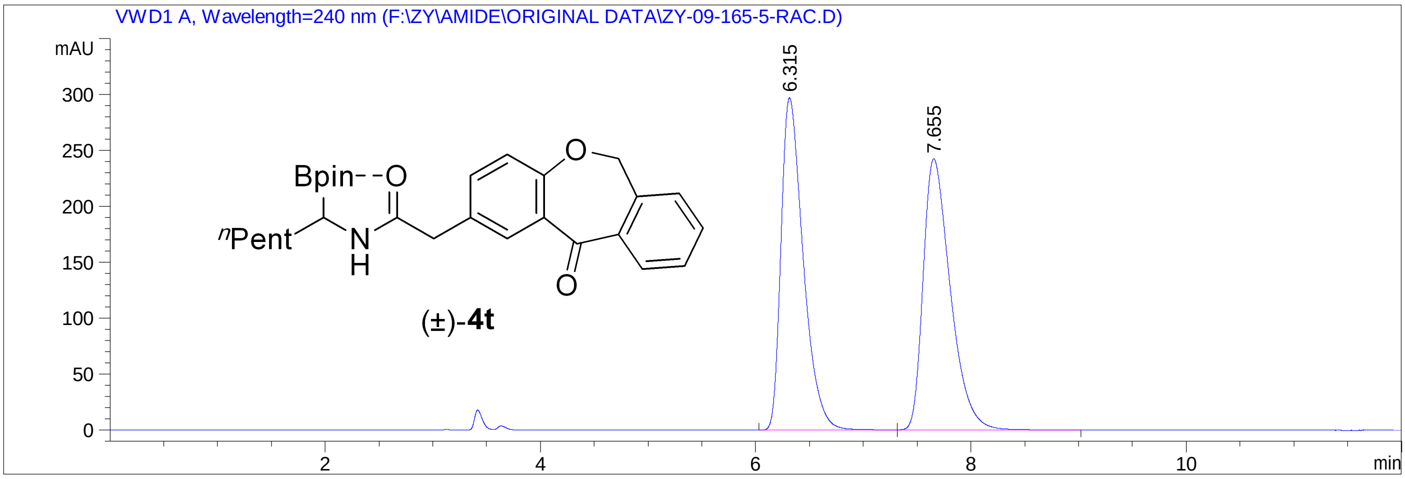

===

Area Percent Report

===

Sorted By : Signal
Multiplier : 1.0000
Dilution : 1.0000
Do not use Multiplier & Dilution Factor with ISTDs

Signal 1: VWD1 A, Wavelength=240 nm

Peak #	RetTime [min]	Type	Width [min]	Area [mAU*s]	Height [mAU]	Area %
1	6.315	BB	0.2177	4259.83740	297.23294	50.0306
2	7.655	BB	0.2678	4254.63379	242.33784	49.9694

Totals : 8514.47119 539.57079

===

*** End of Report ***

Supplementary Figure 261: HPLC spectrum of (±)-**4t**.

Data File F:\ZY\AMIDE\ORIGINAL DATA\ZY-09-165-5-EE.D
Sample Name: ZY-09-165-5-EE

===

Acq. Operator : 系统 Seq. Line : 9
Acq. Instrument : HPLC-1260 Location : 51
Injection Date : 12/27/2021 10:58:49 AM Inj : 1
Inj Volume : 3.000 µl
Different Inj Volume from Sample Entry! Actual Inj Volume : 1.300 µl
Acq. Method : D:\zy\20211226\YH 2021-12-27 08-56-44\10IPA15_1-1-2-240.M
Last changed : 12/27/2021 8:56:44 AM by 系统
Analysis Method : E:\DATA\20220316\LC 2022-03-16 22-19-08\10IPA-10-1.0-1.MZWJ.M (Sequence Method)
Last changed : 3/28/2022 11:11:10 PM by SYSTEM
(modified after loading)

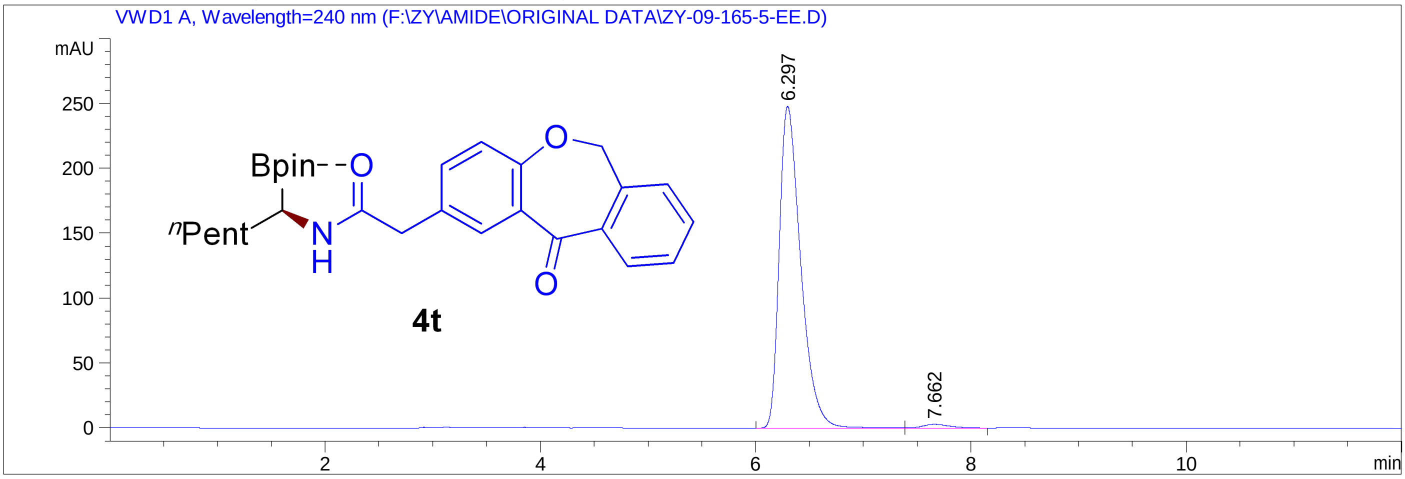

===

Area Percent Report

===

Sorted By : Signal
Multiplier : 1.0000
Dilution : 1.0000
Do not use Multiplier & Dilution Factor with ISTDs

Signal 1: VWD1 A, Wavelength=240 nm

Peak #	RetTime [min]	Type	Width [min]	Area [mAU*s]	Height [mAU]	Area %
1	6.297	BV	0.2116	3430.65576	247.72098	98.5969
2	7.662	VB	0.2450	48.82105	2.81994	1.4031

Totals : 3479.47681 250.54092

===

*** End of Report ***

Supplementary Figure 262: HPLC spectrum of **4t**.

Data File F:\ZY\AMIDE\ORIGINAL DATA\ZY-09-196-5-RAC.D
Sample Name: ZY-09-196-5-RAC

===

Acq. Operator : 系统 Seq. Line : 7
Acq. Instrument : HPLC-1260 Location : 22
Injection Date : 1/2/2022 7:10:31 PM Inj : 1
Inj Volume : 3.000 µl
Different Inj Volume from Sample Entry! Actual Inj Volume : 0.600 µl
Acq. Method : D:\zy\20211226\YH 2022-01-02 17-33-48\5IPA20_8-1-2-240.M
Last changed : 1/2/2022 6:35:59 PM by 系统
Analysis Method : E:\DATA\20220316\LC 2022-03-16 22-19-08\10IPA-10-1.0-1.MZWJ.M (Sequence Method)
Last changed : 3/29/2022 9:57:32 AM by SYSTEM
(modified after loading)
Additional Info : Peak(s) manually integrated

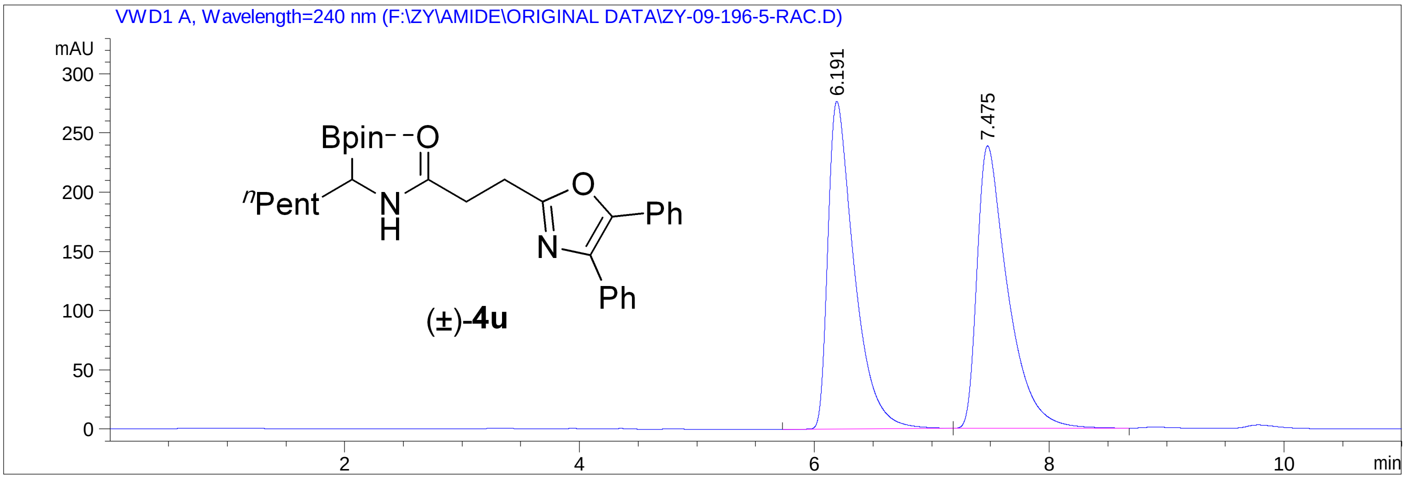

===

Area Percent Report

===

Sorted By : Signal
Multiplier : 1.0000
Dilution : 1.0000
Do not use Multiplier & Dilution Factor with ISTDs

Signal 1: VWD1 A, Wavelength=240 nm

Peak #	RetTime [min]	Type	Width [min]	Area [mAU*s]	Height [mAU]	Area %
1	6.191	BB	0.2315	4319.26270	276.83588	49.8534
2	7.475	BB	0.2675	4344.66309	238.48940	50.1466

Totals : 8663.92578 515.32527

===

*** End of Report ***

Supplementary Figure 263: HPLC spectrum of (±)-**4u**.

Data File F:\ZY\AMIDE\ORIGINAL DATA\ZY-09-196-5-EE.D
Sample Name: ZY-09-196-5-EE

===

Acq. Operator : 系统 Seq. Line : 6
Acq. Instrument : HPLC-1260 Location : 21
Injection Date : 1/2/2022 6:49:09 PM Inj : 1
Inj Volume : 3.000 µl
Different Inj Volume from Sample Entry! Actual Inj Volume : 0.800 µl
Acq. Method : D:\zy\20211226\YH 2022-01-02 17-33-48\5IPA20_8-1-2-240.M
Last changed : 1/2/2022 6:35:59 PM by 系统
Analysis Method : E:\DATA\20220316\LC 2022-03-16 22-19-08\10IPA-10-1.0-1.MZWJ.M (Sequence Method)
Last changed : 3/29/2022 9:58:14 AM by SYSTEM
(modified after loading)
Additional Info : Peak(s) manually integrated

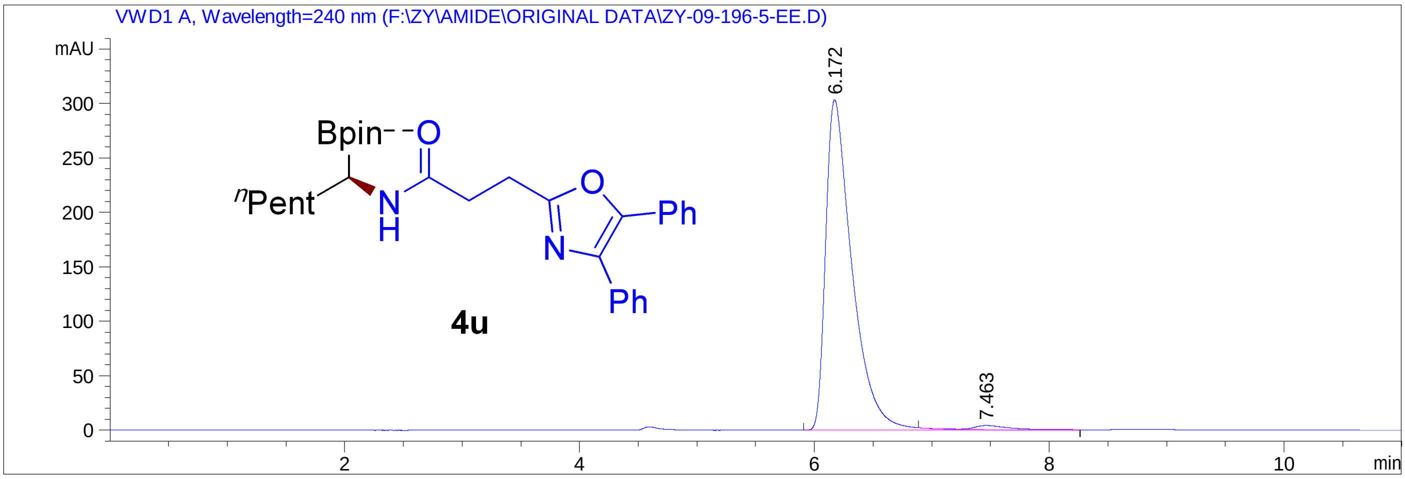

===
Area Percent Report
===

Sorted By : Signal
Multiplier : 1.0000
Dilution : 1.0000
Do not use Multiplier & Dilution Factor with ISTDs

Signal 1: VWD1 A, Wavelength=240 nm

Peak #	RetTime [min]	Type	Width [min]	Area [mAU*s]	Height [mAU]	Area %
1	6.172	BV R	0.2321	4751.24121	303.63879	98.3375
2	7.463	VB E	0.2815	80.32556	3.88019	1.6625

Totals : 4831.56677 307.51899

===
*** End of Report ***

Supplementary Figure 264: HPLC spectrum of **4u**.

Data File F:\ZY\AMIDE\ORIGINAL DATA\ZY-09-196-1-RAC.D
Sample Name: ZY-09-196-1-RAC

===

Acq. Operator : 系统 Seq. Line : 4
Acq. Instrument : HPLC-1260 Location : 62
Injection Date : 12/31/2021 2:05:31 PM Inj : 1
Inj Volume : 3.000 µl
Different Inj Volume from Sample Entry! Actual Inj Volume : 1.000 µl
Acq. Method : D:\zy\20211226\YH 2021-12-31 13-15-03\10IPA15_1-1-2-240.M
Last changed : 12/31/2021 1:15:03 PM by 系统
Analysis Method : E:\DATA\20220316\LC 2022-03-16 22-19-08\10IPA-10-1.0-1.MZWJ.M (Sequence Method)
Last changed : 3/29/2022 9:56:17 AM by SYSTEM
(modified after loading)
Additional Info : Peak(s) manually integrated

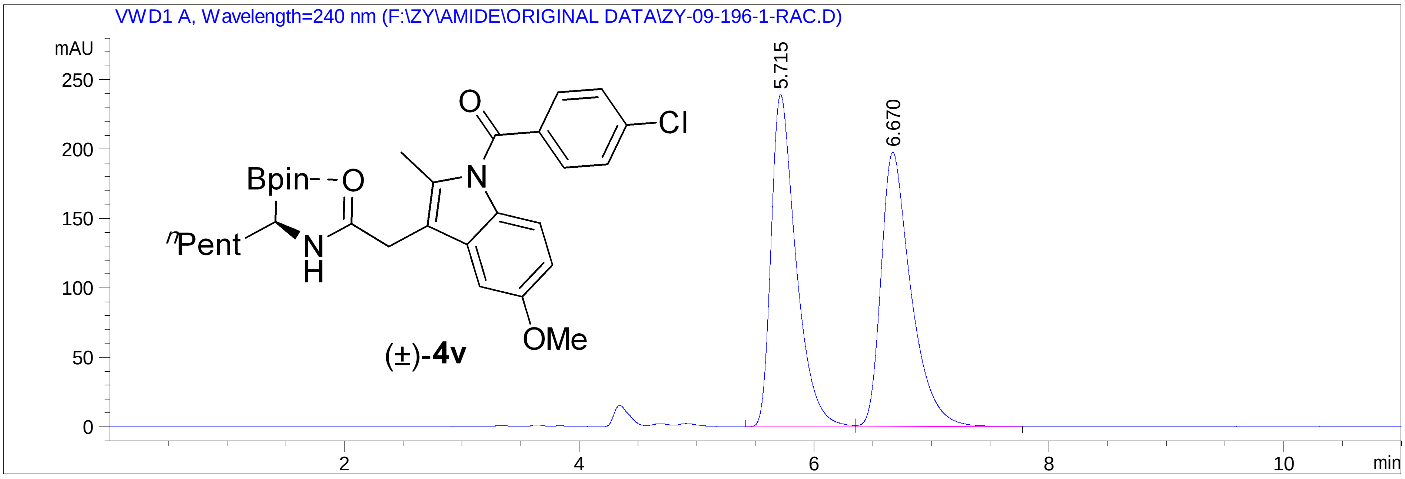

===

Area Percent Report

===

Sorted By : Signal
Multiplier : 1.0000
Dilution : 1.0000
Do not use Multiplier & Dilution Factor with ISTDs

Signal 1: VWD1 A, Wavelength=240 nm

Peak #	RetTime [min]	Type	Width [min]	Area [mAU*s]	Height [mAU]	Area %
1	5.715	BV	0.2207	3531.01099	239.23489	50.1104
2	6.670	VB	0.2635	3515.44824	197.65958	49.8896

Totals : 7046.45923 436.89447

===

*** End of Report ***

Supplementary Figure 265: HPLC spectrum of (±)-**4v**.

Data File F:\ZY\AMIDE\ORIGINAL DATA\ZY-09-196-1-EE.D
Sample Name: ZY-09-196-1-EE

===

Acq. Operator : 系统 Seq. Line : 3
Acq. Instrument : HPLC-1260 Location : 61
Injection Date : 12/31/2021 1:49:06 PM Inj : 1
Inj Volume : 3.000 µl
Different Inj Volume from Sample Entry! Actual Inj Volume : 1.000 µl
Acq. Method : D:\zy\20211226\YH 2021-12-31 13-15-03\10IPA15_1-1-2-240.M
Last changed : 12/31/2021 1:15:03 PM by 系统
Analysis Method : E:\DATA\20220316\LC 2022-03-16 22-19-08\10IPA-10-1.0-1.MZWJ.M (Sequence Method)
Last changed : 3/29/2022 9:56:17 AM by SYSTEM
(modified after loading)

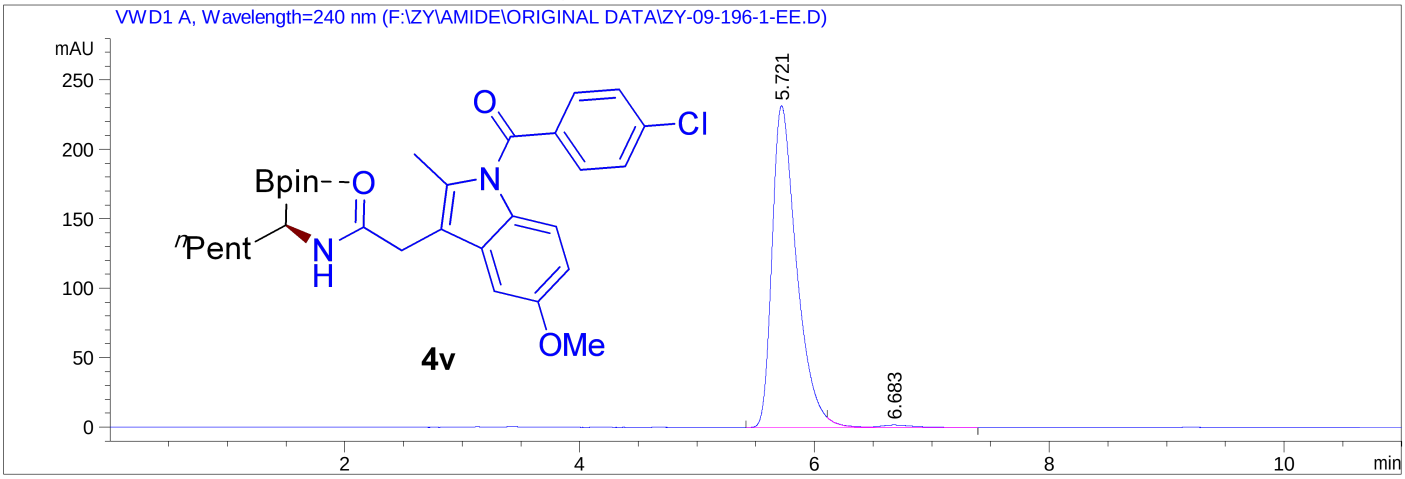

===

Area Percent Report

===

Sorted By : Signal
Multiplier : 1.0000
Dilution : 1.0000
Do not use Multiplier & Dilution Factor with ISTDs

Signal 1: VWD1 A, Wavelength=240 nm

Peak #	RetTime [min]	Type	Width [min]	Area [mAU*s]	Height [mAU]	Area %
1	5.721	BV R	0.2181	3378.15308	231.75551	98.8486
2	6.683	VB E	0.2638	39.34804	1.82285	1.1514

Totals : 3417.50111 233.57836

===

*** End of Report ***

Supplementary Figure 266: HPLC spectrum of **4v**.

Data File F:\ZY\AMIDE\ORIGINAL DATA\ZY-10-41-RAC.D
Sample Name: ZY-10-41-RAC

===

Acq. Operator : 系统 Seq. Line : 6
Acq. Instrument : HPLC-1260 Location : 83
Injection Date : 3/2/2022 9:59:13 PM Inj : 1
Inj Volume : 3.000 µl
Different Inj Volume from Sample Entry! Actual Inj Volume : 2.800 µl
Acq. Method : D:\zy\20220302\YH 2022-03-02 20-49-54\0.5IPA-15-1.0-1-2-220-SFT.M
Last changed : 3/2/2022 9:25:44 PM by 系统
Analysis Method : E:\DATA\20220316\LC 2022-03-16 22-19-08\10IPA-10-1.0-1.MZWJ.M (Sequence Method)
Last changed : 3/29/2022 10:06:28 AM by SYSTEM
(modified after loading)
Additional Info : Peak(s) manually integrated

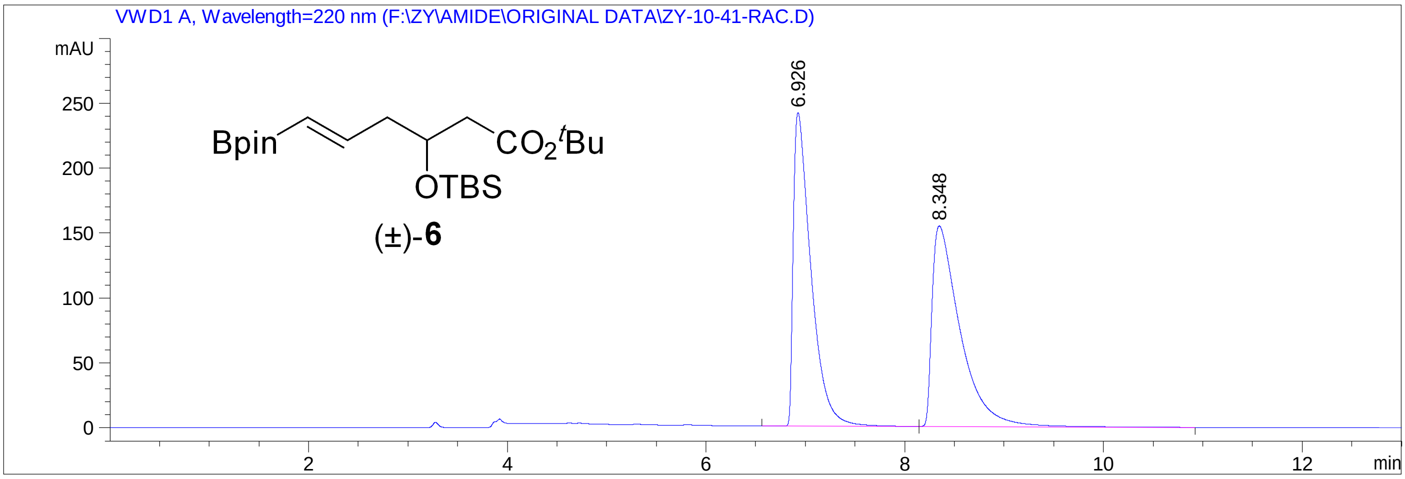

===

Area Percent Report

===

Sorted By : Signal
Multiplier : 1.0000
Dilution : 1.0000
Do not use Multiplier & Dilution Factor with ISTDs

Signal 1: VWD1 A, Wavelength=220 nm

Peak #	RetTime [min]	Type	Width [min]	Area [mAU*s]	Height [mAU]	Area %
1	6.926	VB R	0.1845	2990.16821	241.46602	49.5881
2	8.348	BB	0.2910	3039.84058	154.54143	50.4119

Totals : 6030.00879 396.00745

===

*** End of Report ***

Supplementary Figure 267: HPLC spectrum of (±)-**6**.

Data File F:\ZY\AMIDE\ORIGINAL DATA\ZY-10-41-EE.D
Sample Name: ZY-10-41-EE

===

Acq. Operator : 系统 Seq. Line : 5
Acq. Instrument : HPLC-1260 Location : 82
Injection Date : 3/2/2022 9:44:50 PM Inj : 1
Inj Volume : 3.000 μl
Different Inj Volume from Sample Entry! Actual Inj Volume : 1.500 μl
Acq. Method : D:\zy\20220302\YH 2022-03-02 20-49-54\0.5IPA-15-1.0-1-2-220-SFT.M
Last changed : 3/2/2022 9:25:44 PM by 系统
Analysis Method : E:\DATA\20220316\LC 2022-03-16 22-19-08\10IPA-10-1.0-1.MZWJ.M (Sequence Method)
Last changed : 3/29/2022 10:06:28 AM by SYSTEM
(modified after loading)
Additional Info : Peak(s) manually integrated

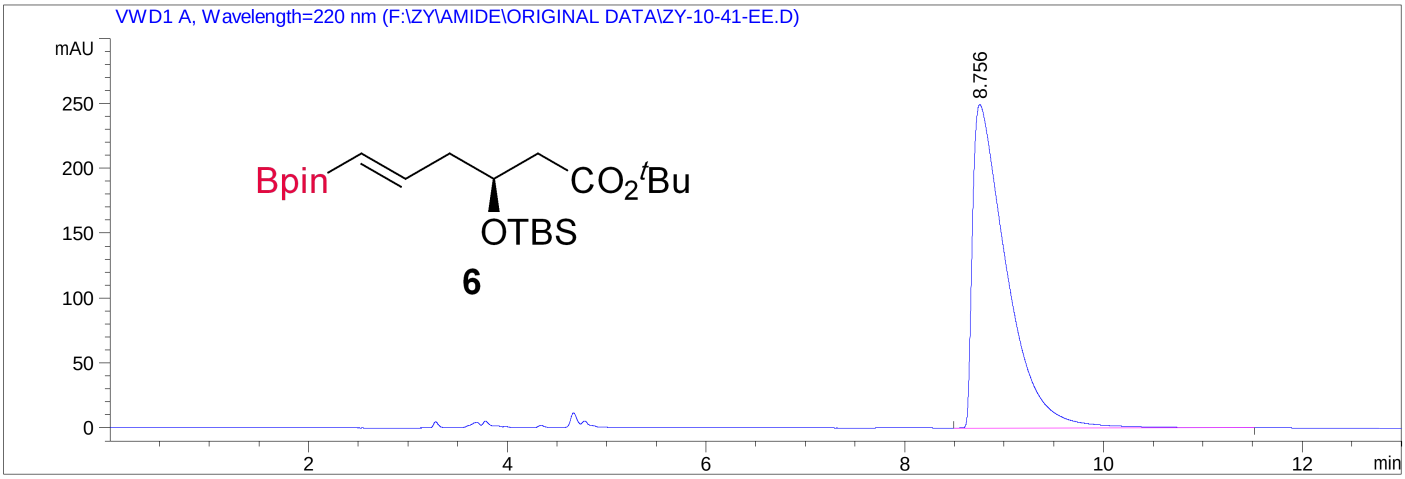

===

Area Percent Report

===

Sorted By : Signal
Multiplier : 1.0000
Dilution : 1.0000
Do not use Multiplier & Dilution Factor with ISTDs

Signal 1: VWD1 A, Wavelength=220 nm

Peak #	RetTime [min]	Type	Width [min]	Area [mAU*s]	Height [mAU]	Area %
1	8.756	BB	0.3500	5931.49121	248.95393	100.0000

Totals : 5931.49121 248.95393

===

*** End of Report ***

Supplementary Figure 268: HPLC spectrum of **6**.

Data File F:\ZY\AMIDE\ORIGINAL DATA\ZY-10-58-2-RAC.D
Sample Name: ZY-10-58-2-RAC

===

Acq. Operator : 系统 Seq. Line : 18
Acq. Instrument : HPLC-1260 Location : 81
Injection Date : 3/23/2022 7:12:12 PM Inj : 1
Inj Volume : 3.000 µl
Different Inj Volume from Sample Entry! Actual Inj Volume : 1.500 µl
Acq. Method : D:\zy\20220302\YH 2022-03-23 15-01-36\5IPA-15-1.0-1-6-240.M
Last changed : 3/23/2022 6:43:30 PM by 系统
Analysis Method : C:\CHEM32\1\METHODS\0.2IPA-10-0.5-1-XYH-EQ.M
Last changed : 3/29/2022 10:11:05 AM by SYSTEM
(modified after loading)
Additional Info : Peak(s) manually integrated

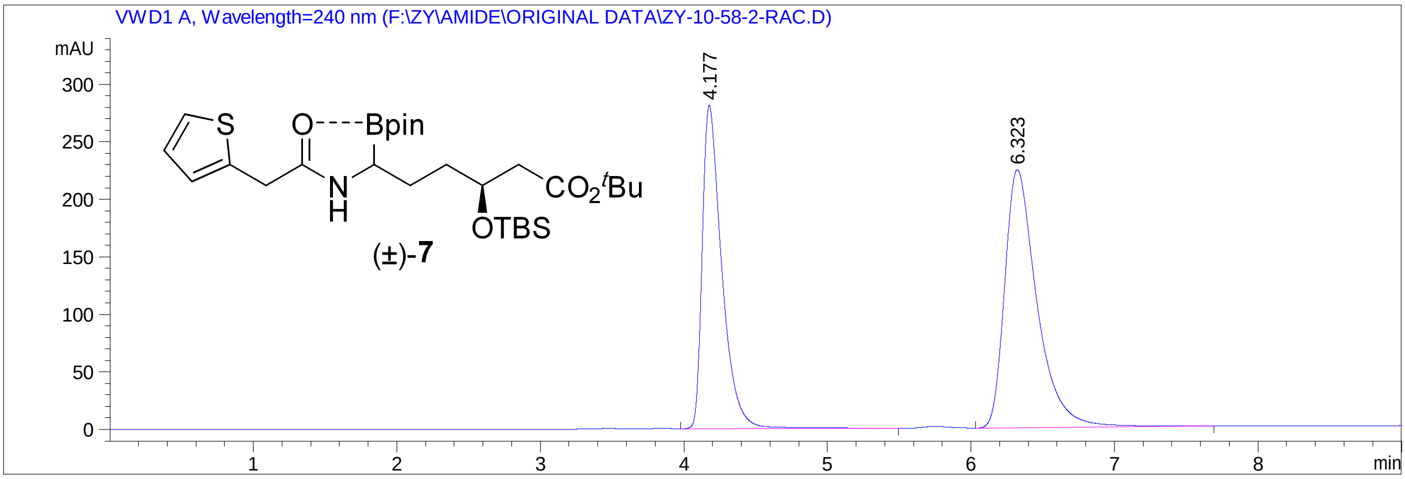

===

Area Percent Report

===

Sorted By : Signal
Multiplier : 1.0000
Dilution : 1.0000
Do not use Multiplier & Dilution Factor with ISTDs

Signal 1: VWD1 A, Wavelength=240 nm

Peak #	RetTime [min]	Type	Width [min]	Area [mAU*s]	Height [mAU]	Area %
1	4.177	BV R	0.1429	2674.33887	281.27936	43.2703
2	6.323	BB	0.2293	3506.19946	224.38475	56.7297

Totals : 6180.53833 505.66411

===

*** End of Report ***

Supplementary Figure 269: HPLC spectrum of (±)-**7**.

Data File F:\ZY\AMIDE\ORIGINAL DATA\ZY-10-58-2-EE.D
Sample Name: ZY-10-58-2

===
Acq. Operator   : 系统   Seq. Line :   7
Acq. Instrument : HPLC-1260   Location :   87
Injection Date : 3/28/2022 11:27:04 AM   Inj :   1
                                              Inj Volume : 3.000 µl
Different Inj Volume from Sample Entry! Actual Inj Volume : 4.500 µl
Acq. Method   : D:\zy\20220328\YH 2022-03-28 10-02-43\5IPA15_10-1-2-240.M
Last changed   : 3/28/2022 10:02:43 AM by 系统
Analysis Method : C:\CHEM32\1\METHODS\0.2IPA-10-0.5-1-XYH-EQ.M
Last changed   : 3/29/2022 10:10:25 AM by SYSTEM
                  (modified after loading)
Additional Info : Peak(s) manually integrated

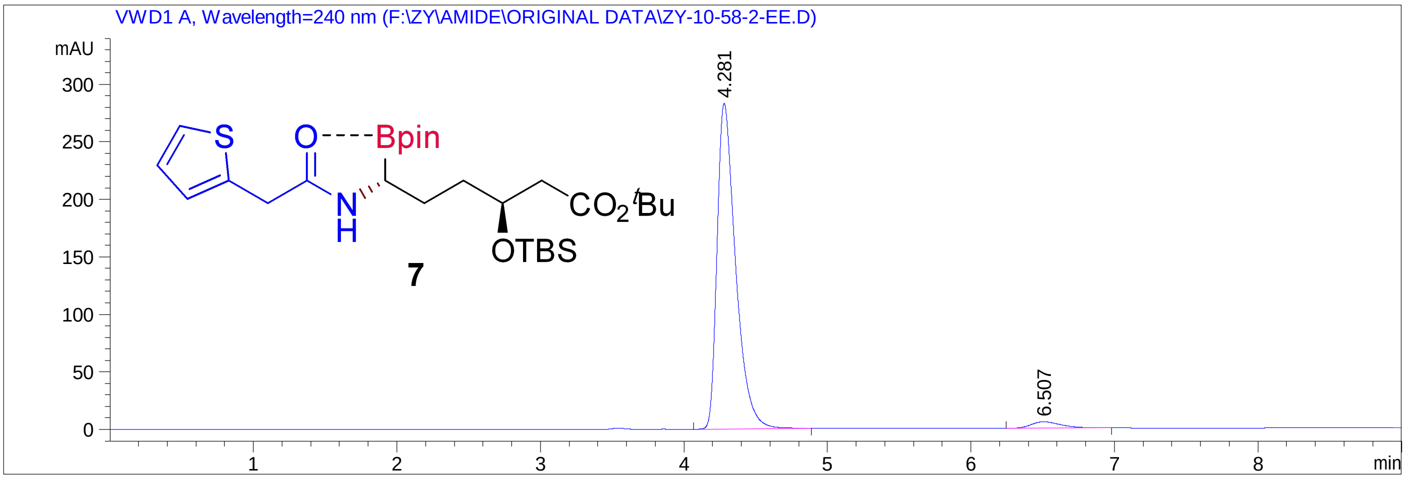

===
Area Percent Report
===

Sorted By : Signal
Multiplier : 1.0000
Dilution : 1.0000
Do not use Multiplier & Dilution Factor with ISTDs

Signal 1: VWD1 A, Wavelength=240 nm

Peak #	RetTime [min]	Type	Width [min]	Area [mAU*s]	Height [mAU]	Area %
1	4.281	BB	0.1348	2543.26392	283.05804	96.8887
2	6.507	BB	0.2216	81.67083	5.60107	3.1113

Totals : 2624.93475 288.65911

===
*** End of Report ***

Supplementary Figure 270: HPLC spectrum of **7**.

III. Supplementary References

1. Berk, S. C. & Buchwald, S. L. An air-stable catalyst system for the conversion of esters to alcohols. *J. Org. Chem.* **57**, 3751–3753 (1992).
2. Bhowmik, A., Mulay, S. V. & Fernandes, R. A. A step-economic synthesis of (*S*)-(–)-Juglomycin C and (*S*)-(–)-NHAB by Dötz benzannulation and convergent deprotections. *Asian J. Org. Chem.* **8**, 1534–1538 (2019).
3. Hecker, S. J., Reddy, K. R., Totrov, M., Hirst, G. C., Lomovskaya, O., Griffith, D. C., King, P., Tsivkovski, R., Sun, D., Sabet, M., Tarazi, Z., Clifton, M. C., Atkins, K., Raymond, A., Potts, K. T., Abendroth, J., Boyer, S. H., Loutit, J. S., Morgan, E. E., Durso, S. & Dudley, M. N. Discovery of a cyclic boronic acid β-lactamase inhibitor (RPX7009) with utility vs class a serine carbapenemases. *J. Med. Chem.* **58**, 3682–3692 (2015).
4. Shimizu, H., Igarashi, T., Miura, T. & Murakami, M. Rhodium-catalyzed reaction of 1-alkenylboronates with aldehydes leading to allylation products. *Angew. Chem. Int. Ed.* **50**, 11465–11469 (2011).
5. Yang, C., Gao, Y., Bai, S., Jiang, C. & Qi, X. Chemoselective cross-coupling of *gem*-borazirconocene alkanes with aryl halides. *J. Am. Chem. Soc.* **142**, 11506–11513 (2020).
6. Kontokosta, D., Mueller, D. S., Wang, H.-Y. & Anderson, L. L. Preparation of α-imino aldehydes by [1,3]-rearrangements of O-alkenyl oximes. *Org. Lett.* **15**, 4830–4833 (2013).
7. Bera, S., Mao, R. & Hu, X. Enantioselective C(sp^3)–C(sp^3) cross-coupling of non-activated alkyl electrophiles via nickel hydride catalysis. *Nat. Chem.* **13**, 270–277 (2021).
8. Coombs, J. R., Zhang, L. & Morken, J. P. Enantiomerically enriched tris(boronates): readily accessible conjunctive reagents for asymmetric synthesis. *J. Am. Chem. Soc.* **136**, 16140–16143 (2014).
9. Fujita, M., Lee, H. J. & Okuyama, T. Stereochemical inversion in the vinylic substitution of boronic esters to give iodonium salts: participation of the internal

oxy group. *Org. Lett.* **8**, 1399–1401 (2006).

10. Zhang, Y., Ma, J., Chen, J., Meng, L., Liang, Y. & Zhu, S. A relay catalysis strategy for enantioselective nickel-catalyzed migratory hydroarylation forming chiral α-aryl alkylboronates. *Chem* **7**, 3171–3188 (2021).
11. Bera, S. & Hu, X. Nickel-catalyzed regioselective hydroalkylation and hydroarylation of alkenyl boronic esters. *Angew. Chem. Int. Ed.* **58**, 13854–13859 (2019).
12. Du, B., Ouyang, Y., Chen, Q. & Yu, W.-Y. Thioether-directed NiH-catalyzed remote γ-C(sp^3)−H hydroamidation of alkenes by 1,4,2-dioxazol-5-ones. *J. Am. Chem. Soc.* **143**, 14962−14968 (2021).
13. Lei, H. & Rovis, T. Ir-catalyzed intermolecular branch-selective allylic C–H amidation of unactivated terminal olefins. *J. Am. Chem. Soc.* **141**, 2268–2273 (2019).
14. Wang, H., Jung, H., Song, F., Zhu, S., Bai, Z., Chen, D., He, G., Chang, S. & Chen, G. Nitrene-mediated intermolecular *N–N* coupling for efficient synthesis of hydrazides. *Nat. Chem.* **13**, 378–385 (2021).
15. Jung, H. J., Park, S., Lee, H. S., Shin, H. G., Yoo, Y., Baral, E. R., Lee, J. H., Kwak, J. & Kim, J. G. Chemical upcycling of waste poly(bisphenol a carbonate) to 1,4,2-dioxazol-5-ones and one-pot C−H amidation. *ChemSusChem* **14**, 4301–4306 (2021).
